# *Fusarium*: more than a node or a foot-shaped basal cell

**DOI:** 10.1016/j.simyco.2021.100116

**Published:** 2021-08-17

**Authors:** P.W. Crous, L. Lombard, M. Sandoval-Denis, K.A. Seifert, H.-J. Schroers, P. Chaverri, J. Gené, J. Guarro, Y. Hirooka, K. Bensch, G.H.J. Kema, S.C. Lamprecht, L. Cai, A.Y. Rossman, M. Stadler, R.C. Summerbell, J.W. Taylor, S. Ploch, C.M. Visagie, N. Yilmaz, J.C. Frisvad, A.M. Abdel-Azeem, J. Abdollahzadeh, A. Abdolrasouli, A. Akulov, J.F. Alberts, J.P.M. Araújo, H.A. Ariyawansa, M. Bakhshi, M. Bendiksby, A. Ben Hadj Amor, J.D.P. Bezerra, T. Boekhout, M.P.S. Câmara, M. Carbia, G. Cardinali, R.F. Castañeda-Ruiz, A. Celis, V. Chaturvedi, J. Collemare, D. Croll, U. Damm, C.A. Decock, R.P. de Vries, C.N. Ezekiel, X.L. Fan, N.B. Fernández, E. Gaya, C.D. González, D. Gramaje, J.Z. Groenewald, M. Grube, M. Guevara-Suarez, V.K. Gupta, V. Guarnaccia, A. Haddaji, F. Hagen, D. Haelewaters, K. Hansen, A. Hashimoto, M. Hernández-Restrepo, J. Houbraken, V. Hubka, K.D. Hyde, T. Iturriaga, R. Jeewon, P.R. Johnston, Ž. Jurjević, İ. Karalti, L. Korsten, E.E. Kuramae, I. Kušan, R. Labuda, D.P. Lawrence, H.B. Lee, C. Lechat, H.Y. Li, Y.A. Litovka, S.S.N. Maharachchikumbura, Y. Marin-Felix, B. Matio Kemkuignou, N. Matočec, A.R. McTaggart, P. Mlčoch, L. Mugnai, C. Nakashima, R.H. Nilsson, S.R. Noumeur, I.N. Pavlov, M.P. Peralta, A.J.L. Phillips, J.I. Pitt, G. Polizzi, W. Quaedvlieg, K.C. Rajeshkumar, S. Restrepo, A. Rhaiem, J. Robert, V. Robert, A.M. Rodrigues, C. Salgado-Salazar, R.A. Samson, A.C.S. Santos, R.G. Shivas, C.M. Souza-Motta, G.Y. Sun, W.J. Swart, S. Szoke, Y.P. Tan, J.E. Taylor, P.W.J. Taylor, P.V. Tiago, K.Z. Váczy, N. van de Wiele, N.A. van der Merwe, G.J.M. Verkley, W.A.S. Vieira, A. Vizzini, B.S. Weir, N.N. Wijayawardene, J.W. Xia, M.J. Yáñez-Morales, A. Yurkov, J.C. Zamora, R. Zare, C.L. Zhang, M. Thines

**Affiliations:** 1 Westerdijk Fungal Biodiversity Institute, 3508 AD, Utrecht, the Netherlands; 2 Wageningen University and Research Centre (WUR), Laboratory of Phytopathology, Droevendaalsesteeg 1, 6708 PB, Wageningen, the Netherlands; 3 Netherlands Institute of Ecology (NIOO-KNAW), Department of Microbial Ecology, Droevendaalsesteeg 10, 6708 PB, Wageningen, the Netherlands; 4 Department of Biology, Carleton University, 1125 Colonel By Drive, Ottawa, Ontario, K1S 5B6, Canada; 5 Plant Protection Department, Agricultural Institute of Slovenia, Hacquetova ulica 17, 1000, Ljubljana, Slovenia; 6 Department of Plant Science and Landscape Architecture, University of Maryland, College Park, MD, USA; 7 Escuela de Biología and Centro de Investigaciones en Productos Naturales, Universidad de Costa Rica, San Pedro, Costa Rica; 8 Unitat de Micologia, Facultat de Medicina i Ciències de la Salut i Institut d’Investigació Sanitària Pere Virgili (IISPV), Universitat Rovira i Virgili, 43201, Reus, Spain; 9 Department of Clinical Plant Science, Faculty of Bioscience, Hosei University, 3-7-2 Kajino-cho, Koganei, Tokyo, 184-8584, Japan; 10 ARC-Plant Health and Protection, Private Bag X5017, Stellenbosch, 7599, Western Cape, South Africa; 11 State Key Laboratory of Mycology, Institute of Microbiology, Chinese Academy of Sciences, Beijing, 100101, China; 12 University of Chinese Academy of Sciences, Beijing, 100049, China; 13 Department of Botany & Plant Pathology, Oregon State University, Corvallis, OR, 97330, USA; 14 Department of Microbial Drugs, Helmholtz Centre for Infection Research GmbH (HZI), Inhoffenstrasse 7, 38124 Braunschweig, Germany; 15 Sporometrics, Toronto, ON, Canada; 16 Dalla Lana School of Public Health, University of Toronto, Toronto, ON, Canada; 17 Plant and Microbial Biology, 111 Koshland Hall, University of California, Berkeley, CA, 94720-3102, USA; 18 Senckenberg Biodiversity and Climate Research Center, Senckenberganlage 25, D-60325, Frankfurt am Main, Germany; 19 Department of Biochemistry, Genetics and Microbiology, Forestry and Agricultural Biotechnology Institute (FABI), Faculty of Natural and Agricultural Sciences, University of Pretoria, P. Bag X20, Hatfield, 0028, Pretoria, South Africa; 20 Department of Biotechnology and Biomedicine, DTU-Bioengineering, Technical University of Denmark, 2800, Kongens Lyngby, Denmark; 21 Systematic Mycology Lab., Botany and Microbiology Department, Faculty of Science, Suez Canal University, Ismailia, 41522, Egypt; 22 Department of Plant Protection, Faculty of Agriculture, University of Kurdistan, P.O. Box 416, Sanandaj, Iran; 23 Department of Medical Microbiology, King's College Hospital, London, UK; 24 Department of Infectious Diseases, Imperial College London, London, UK; 25 Department of Mycology and Plant Resistance, V. N. Karazin Kharkiv National University, Maidan Svobody 4, 61022, Kharkiv, Ukraine; 26 Department of Food Science and Technology, Cape Peninsula University of Technology, P.O. Box 1906, Bellville, 7535, South Africa; 27 School of Forest Resources and Conservation, University of Florida, Gainesville, FL, USA; 28 Department of Plant Pathology and Microbiology, College of Bio-Resources and Agriculture, National Taiwan University, No.1, Sec.4, Roosevelt Road, Taipei, 106, Taiwan, ROC; 29 Iranian Research Institute of Plant Protection, Agricultural Research, Education and Extension Organization (AREEO), P.O. Box 19395-1454, Tehran, Iran; 30 Natural History Museum, University of Oslo, Norway; 31 Department of Natural History, NTNU University Museum, Trondheim, Norway; 32 Setor de Micologia/Departamento de Biociências e Tecnologia, Instituto de Patologia Tropical e Saúde Pública, Rua 235 - s/n – Setor Universitário - CEP: 74605-050, Universidade Federal de Goiás/Federal University of Goiás, Goiânia, Brazil; 33 Departamento de Agronomia, Universidade Federal Rural de Pernambuco, Recife, 52171-900, PE, Brazil; 34 Departamento de Parasitología y Micología, Instituto de Higiene, Facultad de Medicina – Universidad de la República, Av. A. Navarro 3051, Montevideo, Uruguay; 35 Department of Pharmaceutical Science, University of Perugia, Via Borgo 20 Giugno, 74 Perugia, Italy; 36 Instituto de Investigaciones Fundamentales en Agricultura Tropical Alejandro de Humboldt (INIFAT), Académico Titular de la Academia de Ciencias de, Cuba; 37 Grupo de Investigación Celular y Molecular de Microorganismos Patógenos (CeMoP), Departamento de Ciencias Biológicas, Universidad de Los Andes, Bogotá, 111711, Colombia; 38 Mycology Laboratory, New York State Department of Health Wadsworth Center, Albany, NY, USA; 39 Laboratory of Evolutionary Genetics, Institute of Biology, University of Neuchatel, CH-2000, Neuchatel, Switzerland; 40 Senckenberg Museum of Natural History Görlitz, PF 300 154, 02806, Görlitz, Germany; 41 Mycothèque de l'Université catholique de Louvain (MUCL, BCCMTM), Earth and Life Institute – ELIM – Mycology, Université catholique de Louvain, Croix du Sud 2 bte L7.05.06, B-1348, Louvain-la-Neuve, Belgium; 42 Department of Microbiology, Babcock University, Ilishan Remo, Ogun State, Nigeria; 43 The Key Laboratory for Silviculture and Conservation of Ministry of Education, Beijing Forestry University, Beijing, 100083, China; 44 Laboratorio de Micología Clínica, Hospital de Clínicas, Universidad de Buenos Aires, Buenos Aires, Argentina; 45 Facultad de Farmacia y Bioquímica, Universidad de Buenos Aires, Buenos Aires, Argentina; 46 Royal Botanic Gardens, Kew, Richmond, Surrey, TW9 3DS, UK; 47 Laboratorio de Salud de Bosques y Ecosistemas, Instituto de Conservación, Biodiversidad y Territorio, Facultad de Ciencias Forestales y Recursos Naturales, Universidad Austral de Chile, casilla 567, Valdivia, Chile; 48 Institute of Grapevine and Wine Sciences (ICVV), Spanish National Research Council (CSIC)-University of La Rioja-Government of La Rioja, Logroño, 26007, Spain; 49 Institut für Biologie, Karl-Franzens-Universität Graz, Holteigasse 6, 8010, Graz, Austria; 50 Applied genomics research group, Universidad de los Andes, Cr 1 # 18 a 12, Bogotá, Colombia; 51 Center for Safe and Improved Food, Scotland's Rural College (SRUC), Kings Buildings, West Mains Road, Edinburgh, EH9 3JG, UK; 52 Biorefining and Advanced Materials Research Center, Scotland's Rural College (SRUC), Kings Buildings, West Mains Road, Edinburgh, EH9 3JG, UK; 53 Department of Agricultural, Forestry and Food Sciences (DISAFA), University of Torino, Largo P. Braccini 2, 10095, Grugliasco, TO, Italy; 54 BioAware, Hannut, Belgium; 55 Research Group Mycology, Department of Biology, Ghent University, 35 K.L. Ledeganckstraat, 9000, Ghent, Belgium; 56 Faculty of Science, University of South Bohemia, Branišovská 31, 370 05, České Budějovice, Czech Republic; 57 Department of Botany, Swedish Museum of Natural History, P.O. Box 50007, SE-104 05, Stockholm, Sweden; 58 Microbe Division/Japan Collection of Microorganisms RIKEN BioResource Research Center, 3-1-1 Koyadai, Tsukuba, Ibaraki, 305-0074, Japan; 59 Department of Botany, Charles University in Prague, Prague, Czech Republic; 60 Center of Excellence in Fungal Research, Mae Fah Luang University, Chaing Rai, 57100, Thailand; 61 Cornell University, 334 Plant Science Building, Ithaca, NY, 14850, USA; 62 Department of Health Sciences, Faculty of Medicine and Health Sciences, University of Mauritius, Reduit, Mauritius; 63 Manaaki Whenua Landcare Research, Private Bag 92170, Auckland, 1142, New Zealand; 64 EMSL Analytical, Inc., 200 Route 130 North, Cinnaminson, NJ, 08077, USA; 65 Department of Nutrition and Dietetics, Faculty of Health Sciences, Yeditepe University, Turkey; 66 Department of Plant and Soil Sciences, University of Pretoria, P. Bag X20 Hatfield, Pretoria, 0002, South Africa; 67 Institute of Environmental Biology, Ecology and Biodiversity, Utrecht University, 3584 CH, Utrecht, the Netherlands; 68 Laboratory for Biological Diversity, Ruđer Bošković Institute, Bijenička cesta 54, HR-10000, Zagreb, Croatia; 69 University of Veterinary Medicine, Vienna (VetMed), Institute of Food Safety, Food Technology and Veterinary Public Health, Veterinaerplatz 1, 1210 Vienna and BiMM – Bioactive Microbial Metabolites group, 3430 Tulln a.d. Donau, Austria; 70 University of California, Davis, One Shields Ave., Davis, CA, 95616, USA; 71 Department of Agricultural Biological Chemistry, College of Agriculture & Life Sciences, Chonnam National University, Yongbong-Dong 300, Buk-Gu, Gwangju, 61186, South Korea; 72 Ascofrance, 64 route de Chizé, 79360, Villiers-en-Bois, France; 73 The Key Laboratory of Molecular Biology of Crop Pathogens and Insects of Ministry of Agriculture, The Key Laboratory of Biology of Crop Pathogens and Insects of Zhejiang Province, Institute of Biotechnology, Zhejiang University, 866 Yuhangtang Road, Hangzhou, 310058, China; 74 V.N. Sukachev Institute of Forest SB RAS, Laboratory of Reforestation, Mycology and Plant Pathology, Krasnoyarsk, 660036, Russia; 75 Reshetnev Siberian State University of Science and Technology, Department of Chemical Technology of Wood and Biotechnology, Krasnoyarsk, 660037, Russia; 76 School of Life Science and Technology, University of Electronic Science and Technology of China, Chengdu, 611731, China; 77 Queensland Alliance for Agriculture and Food Innovation, The University of Queensland, Ecosciences Precinct, G.P.O. Box 267, Brisbane, 4001, Australia; 78 Department of Botany, Faculty of Science, Palacký University Olomouc, Šlechtitelů 27, CZ-783 71, Olomouc, Czech Republic; 79 Department of Agricultural, Food, Environmental and Forestry Science and Technology (DAGRI), Plant Pathology and Entomology section, University of Florence, P.le delle Cascine 28, 50144, Firenze, Italy; 80 Graduate school of Bioresources, Mie University, Kurima-machiya 1577, Tsu, Mie, 514-8507, Japan; 81 Gothenburg Global Biodiversity Center at the Department of Biological and Environmental Sciences, University of Gothenburg, Box 461, 405 30, Gothenburg, Sweden; 82 Department of Microbiology and Biochemistry, Faculty of Natural and Life Sciences, University of Batna 2, Batna, 05000, Algeria; 83 Laboratorio de Micodiversidad y Micoprospección, PROIMI-CONICET, Av. Belgrano y Pje. Caseros, Argentina; 84 Universidade de Lisboa, Faculdade de Ciências, Biosystems and Integrative Sciences Institute (BioISI), Campo Grande, 1749-016, Lisbon, Portugal; 85 Microbial Screening Technologies, 28 Percival Rd, Smithfield, NSW, 2164, Australia; 86 Dipartimento di Agricoltura, Alimentazione e Ambiente, sez. Patologia vegetale, University of Catania, Via S. Sofia 100, 95123 Catania, Italy; 87 Phytopathology, Van Zanten Breeding B.V., Lavendelweg 15, 1435 EW, Rijsenhout, the Netherlands; 88 National Fungal Culture Collection of India (NFCCI), Biodiversity and Palaeobiology (Fungi) Group, Agharkar Research Institute, Pune, Maharashtra, 411 004, India; 89 Laboratory of Mycology and Phytopathology – (LAMFU), Department of Chemical and Food Engineering, Universidad de los Andes, Cr 1 # 18 a 12, Bogotá, Colombia; 90 Plant Pathology and Population Genetics, Laboratory of Microorganisms, National Gene Bank, Tunisia; 91 Laboratory of Emerging Fungal Pathogens, Department of Microbiology, Immunology, and Parasitology, Discipline of Cellular Biology, Federal University of São Paulo (UNIFESP), São Paulo, 04023062, Brazil; 92 USDA-ARS Mycology & Nematology Genetic Diversity & Biology Laboratory, Bldg. 010A, Rm. 212, BARC-West, 10300 Baltimore Ave, Beltsville, MD, 20705, USA; 93 Departamento de Micologia Prof. Chaves Batista, Universidade Federal de Pernambuco, Centro de Biociências, Cidade Universitária, Av. Prof. Moraes Rego, s/n, Recife, PE, CEP: 50670-901, Brazil; 94 Centre for Crop Health, University of Southern Queensland, Toowoomba, 4350, Queensland, Australia; 95 College of Plant Protection, Northwest A&F University, Yangling, Shaanxi, China; 96 Faculty of Natural and Agricultural Sciences, Department of Plant Sciences, University of the Free State, P.O. Box 339, Bloemfontein, 9300, South Africa; 97 Queensland Plant Pathology Herbarium, Department of Agriculture and Fisheries, Dutton Park, Queensland, 4102, Australia; 98 Royal Botanic Garden Edinburgh, 20A Inverleith Row, Edinburgh, EH3 5LR, United Kingdom; 99 Faculty of Veterinary and Agricultural Sciences, The University of Melbourne, Parkville, VIC, 3010, Australia; 100 Food and Wine Research Institute, Eszterházy Károly University, 6 Leányka Street, H-3300, Eger, Hungary; 101 Department of Life Sciences and Systems Biology, University of Torino and Institute for Sustainable Plant Protection (IPSP-SS Turin), C.N.R, Viale P.A. Mattioli, 25, I-10125, Torino, Italy; 102 Center for Yunnan Plateau Biological Resources Protection and Utilization, College of Biological Resource and Food Engineering, Qujing Normal University, Qujing, Yunnan, 655011, China; 103 Shandong Provincial Key Laboratory for Biology of Vegetable Diseases and Insect Pests, College of Plant Protection, Shandong Agricultural University, Taian, 271018, China; 104 Fitosanidad, Colegio de Postgraduados-Campus Montecillo, Montecillo-Texcoco, 56230 Edo. de Mexico, Mexico; 105 Leibniz Institute DSMZ-German Collection of Microorganisms and Cell Cultures GmbH, Inhoffenstrasse 7 B, 38124, Braunschweig, Germany; 106 Museum of Evolution, Uppsala University, Norbyvägen 16, SE-752 36, Uppsala, Sweden; 107 Ministry of Agriculture Key Laboratory of Molecular Biology of Crop Pathogens and Insects, Institute of Biotechnology, College of Agriculture and Biotechnology, Zhejiang University, No. 866 Yuhangtang Road, Hangzhou, 310058, China; 108 Goethe-University Frankfurt am Main, Department of Biological Sciences, Institute of Ecology, Evolution and Diversity, Max-von-Laue Str. 13, D-60438, Frankfurt am Main, Germany; 109 LOEWE Centre for Translational Biodiversity Genomics, Georg-Voigt-Str. 14-16, D-60325, Frankfurt am Main, Germany

**Keywords:** Multi-gene phylogeny, Mycotoxins, *Nectriaceae*, *Neocosmospora*, Novel taxa, Pathogen, Taxonomy, *Luteonectria* Sand.-Den., L. Lombard, Schroers & Rossman, *Nothofusarium* Crous, Sand.-Den. & L. Lombard, *Scolecofusarium* L. Lombard, Sand.-Den. & Crous, *Setofusarium* (Nirenberg & Samuels) Crous & Sand.-Den., *Fusarium echinatum* Sand.-Den. & G.J. Marais, *Fusarium lyarnte* J.L. Walsh, Sangal., L.W. Burgess, E.C.Y. Liew & Summerell, *Fusarium palustre* W.H. Elmer & Marra, *Fusarium prieskaense* G.J. Marais & Sand.-Den., *Fusarium werrikimbe* J.L. Walsh, L.W. Burgess, E.C.Y. Liew & B.A. Summerell, *Fusicolla quarantenae* J.D.P. Bezerra, Sand.-Den., Crous & Souza-Motta, *Fusicolla meniscoidea* L. Lombard & Sand.-Den., *Fusicolla sporellula* Sand.-Den. & L. Lombard, *Macroconia bulbipes* Crous & Sand.-Den., *Macroconia phlogioides* Sand.-Den. & Crous, *Neocosmospora epipeda* Quaedvl. & Sand.-Den., *Neocosmospora merkxiana* Quaedvl. & Sand.-Den., *Neocosmospora neerlandica* Crous & Sand.-Den., *Neocosmospora nelsonii* Crous & Sand.-Den., *Neocosmospora pseudopisi* Sand.-Den. & L. Lombard, *Nothofusarium devonianum* L. Lombard, Crous & Sand.-Den., *Stylonectria corniculata* Gräfenhan, Crous & Sand.-Den., *Stylonectria hetmanica* Akulov, Crous & Sand.-Den., *Apiognomonia platani* (Lév.) L. Lombard, *Calloria tremelloides* (Grev.) L. Lombard, *Cosmosporella cavisperma* (Corda) Sand.-Den., L. Lombard & Crous, *Cylindrodendrum orthosporum* (Sacc. & P. Syd.) L. Lombard, *Dialonectria volutella* (Ellis & Everh.) L. Lombard & Sand.-Den., *Fusarium armeniacum* (G.A. Forbes *et al.*) L.W. Burgess & Summerell, *Hymenella aurea* (Corda) L. Lombard, *Hymenella spermogoniopsis* (Jul. Müll.) L. Lombard & Sand.-Den., *Luteonectria albida* (Rossman) Sand.-Den. & L. Lombard, *Luteonectria nematophila* (Nirenberg & Hagedorn) Sand.-Den. & L. Lombard, *Neocosmospora floridana* (T. Aoki *et al.*) L. Lombard & Sand.-Den., *Neocosmospora obliquiseptata* (T. Aoki *et al.*) L. Lombard & Sand.-Den., *Neocosmospora rekana* (Lynn & Marinc.) L. Lombard & Sand.-Den., *Neocosmospora tuaranensis* (T. Aoki *et al.*) L. Lombard & Sand.-Den., *Scolecofusarium ciliatum* (Link) L. Lombard, Sand.-Den. & Crous, *Setofusarium setosum* (Samuels & Nirenberg) Sand.-Den. & Crous., *Fusarium buharicum* Jacz. ex Babajan & Teterevn.-Babajan, *Fusarium cavispermum* Corda, *Fusarium flocciferum* Corda, *Fusarium graminearum* Schwabe, *Fusarium heterosporum* Nees & T. Nees, *Fusarium redolens* Wollenw., *Fusarium reticulatum* Mont., *Fusarium scirpi* Lambotte & Fautrey, *Fusarium stilboides* Wollenw., *Fusarium xylarioides* Steyaert, *Fusisporium culmorum* Wm.G. Sm., *Fusisporium incarnatum* Roberge ex Desm., *Selenosporium equiseti* Corda, *Sphaeria sanguinea* var. *cicatricum* Berk., *Sporotrichum poae* Peck., *Atractium pallidum* Bonord., *Cephalosporium sacchari* E.J. Butler, *Fusarium aeruginosum* Delacr., *Fusarium agaricorum* Sarrazin, *Fusarium albidoviolaceum* Dasz., *Fusarium aleyrodis* Petch, *Fusarium amentorum* Lacroix, *Fusarium annuum* Leonian, *Fusarium arcuatum* Berk. & M.A. Curtis, *Fusarium aridum* O.A. Pratt, *Fusarium arthrosporioides* Sherb., *Fusarium asparagi* Delacr., *Fusarium batatas* Wollenw., *Fusarium biforme* Sherb., *Fusarium cactacearum* Pasin. & Buzz.-Trav., *Fusarium cacti-maxonii* Pasin. & Buzz.-Trav., *Fusarium caudatum* Wollenw., *Fusarium cavispermum* Corda, *Fusarium cepae* Hanzawa, *Fusarium cesatii* Rabenh., *Fusarium citriforme* Jamal., *Fusarium citrinum* Wollenw., *Fusarium citrulli* Taubenh., *Fusarium clavatum* Sherb., *Fusarium coccinellum* Kalchbr., *Fusarium cromyophthoron* Sideris, *Fusarium cucurbitae* Taubenh., *Fusarium cuneiforme* Sherb., *Fusarium delacroixii* Sacc., *Fusarium dimerum* var. *nectrioides* Wollenw., *Fusarium epicoccum* McAlpine, *Fusarium eucheliae* Sartory, R. Sartory & J. Mey., *Fusarium fissum* Peyl, *Fusarium flocciferum* Corda, *Fusarium gemmiperda* Aderh., *Fusarium genevense* Dasz., *Fusarium graminearum* Schwabe, *Fusarium graminum* Corda, *Fusarium heterosporioides* Fautrey, *Fusarium heterosporum* Nees & T. Nees, *Fusarium idahoanum* O.A. Pratt, *Fusarium juruanum* Henn., *Fusarium lanceolatum* O.A. Pratt, *Fusarium lateritium* Nees, *Fusarium loncheceras* Sideris, *Fusarium malvacearum* Taubenh., *Fusarium martii* f. *phaseoli* Burkh., *Fusarium muentzii* Delacr., *Fusarium nigrum* O.A. Pratt, *Fusarium oxysporum* var. *asclerotium* Sherb., *Fusarium palczewskii* Jacz., *Fusarium polymorphum* Matr., *Fusarium poolense* Taubenh., *Fusarium prunorum* McAlpine, *Fusarium pusillum* Wollenw., *Fusarium putrefaciens* Osterw., *Fusarium redolens* Wollenw., *Fusarium reticulatum* Mont., *Fusarium rhizochromatistes* Sideris, *Fusarium rhizophilum* Corda, *Fusarium rhodellum* McAlpine, *Fusarium roesleri* Thüm., *Fusarium rostratum* Appel & Wollenw., *Fusarium rubiginosum* Appel & Wollenw., *Fusarium rubrum* Parav., *Fusarium samoense* Gehrm., *Fusarium scirpi* Lambotte & Fautrey, *Fusarium secalis* Jacz., *Fusarium spinaciae* Hungerf., *Fusarium sporotrichioides* Sherb., *Fusarium stercoris* Fuckel, *Fusarium stilboides* Wollenw., *Fusarium stillatum* De Not. ex Sacc., *Fusarium sublunatum* Reinking, *Fusarium succisae* Schröt. ex Sacc., *Fusarium tabacivorum* Delacr., *Fusarium trichothecioides* Wollenw., *Fusarium tritici* Liebman, *Fusarium tuberivorum* Wilcox & G.K. Link, *Fusarium tumidum* var. *humi* Reinking, *Fusarium ustilaginis* Kellerm. & Swingle, *Fusarium viticola* Thüm., *Fusarium willkommii* Lindau, *Fusarium xylarioides* Steyaert, *Fusarium zygopetali* Delacr., *Fusisporium andropogonis* Cooke ex Thüm., *Fusisporium anthophilum* A. Braun, *Fusisporium arundinis* Corda, *Fusisporium clypeaster* Corda, *Fusisporium culmorum* Wm.G. Sm., *Fusisporium didymum* Harting, *Fusisporium elasticae* Thüm., *Fusisporium episphaericum* Cooke & Ellis, *Fusisporium flavidum* Bonord., *Fusisporium hordei* Wm.G. Sm., *Fusisporium incarnatum* Roberge ex Desm., *Fusisporium lolii* Wm.G. Sm., *Fusisporium pandani* Corda, *Gibberella phyllostachydicola* W. Yamam., *Menispora penicillata* Harz, *Selenosporium equiseti* Corda, *Selenosporium hippocastani* Corda, *Selenosporium urticearum* Corda., *Sphaeria sanguinea* var. *cicatricum* Berk., *Atractium ciliatum* Link, *Fusarium longipes* Wollenw. & Reinking, *Fusisporium avenaceum* Fr., *Selenosporium sarcochroum* Desm

## Abstract

Recent publications have argued that there are potentially serious consequences for researchers in recognising distinct genera in the terminal fusarioid clade of the family *Nectriaceae*. Thus, an alternate hypothesis, namely a very broad concept of the genus *Fusarium* was proposed. In doing so, however, a significant body of data that supports distinct genera in *Nectriaceae* based on morphology, biology, and phylogeny is disregarded. A DNA phylogeny based on 19 orthologous protein-coding genes was presented to support a very broad concept of *Fusarium* at the F1 node in *Nectriaceae*. Here, we demonstrate that re-analyses of this dataset show that all 19 genes support the F3 node that represents *Fusarium sensu stricto* as defined by *F. sambucinum* (sexual morph synonym *Gibberella pulicaris*). The backbone of the phylogeny is resolved by the concatenated alignment, but only six of the 19 genes fully support the F1 node, representing the broad circumscription of *Fusarium.* Furthermore, a re-analysis of the concatenated dataset revealed alternate topologies in different phylogenetic algorithms, highlighting the deep divergence and unresolved placement of various *Nectriaceae* lineages proposed as members of *Fusarium*. Species of *Fusarium s. str.* are characterised by *Gibberella* sexual morphs, asexual morphs with thin- or thick-walled macroconidia that have variously shaped apical and basal cells, and trichothecene mycotoxin production, which separates them from other fusarioid genera. Here we show that the Wollenweber concept of *Fusarium* presently accounts for 20 segregate genera with clear-cut synapomorphic traits, and that fusarioid macroconidia represent a character that has been gained or lost multiple times throughout *Nectriaceae*. Thus, the very broad circumscription of *Fusarium* is blurry and without apparent synapomorphies, and does not include all genera with fusarium-like macroconidia, which are spread throughout *Nectriaceae* (*e.g.*, *Cosmosporella*, *Macroconia*, *Microcera*). In this study four new genera are introduced, along with 18 new species and 16 new combinations. These names convey information about relationships, morphology, and ecological preference that would otherwise be lost in a broader definition of *Fusarium*. To assist users to correctly identify fusarioid genera and species, we introduce a new online identification database, Fusarioid-ID, accessible at www.fusarium.org. The database comprises partial sequences from multiple genes commonly used to identify fusarioid taxa (*act1*, *CaM*, *his3*, *rpb1*, *rpb2*, *tef1*, *tub2*, ITS, and LSU). In this paper, we also present a nomenclator of names that have been introduced in *Fusarium* up to January 2021 as well as their current status, types, and diagnostic DNA barcode data. In this study, researchers from 46 countries, representing taxonomists, plant pathologists, medical mycologists, quarantine officials, regulatory agencies, and students, strongly support the application and use of a more precisely delimited *Fusarium* (= *Gibberella*) concept to accommodate taxa from the robust monophyletic node F3 on the basis of a well-defined and unique combination of morphological and biochemical features. This F3 node includes, among others, species of the *F. fujikuroi, F. incarnatum-equiseti, F. oxysporum,* and *F. sambucinum* species complexes, but not species of *Bisifusarium* [*F. dimerum* species complex (SC)], *Cyanonectria* (*F. buxicola* SC), *Geejayessia* (*F. staphyleae* SC), *Neocosmospora* (*F. solani* SC) or *Rectifusarium* (*F. ventricosum* SC). The present study represents the first step to generating a new online monograph of *Fusarium* and allied fusarioid genera (www.fusarium.org).

## Introduction

**Fig. 1:**
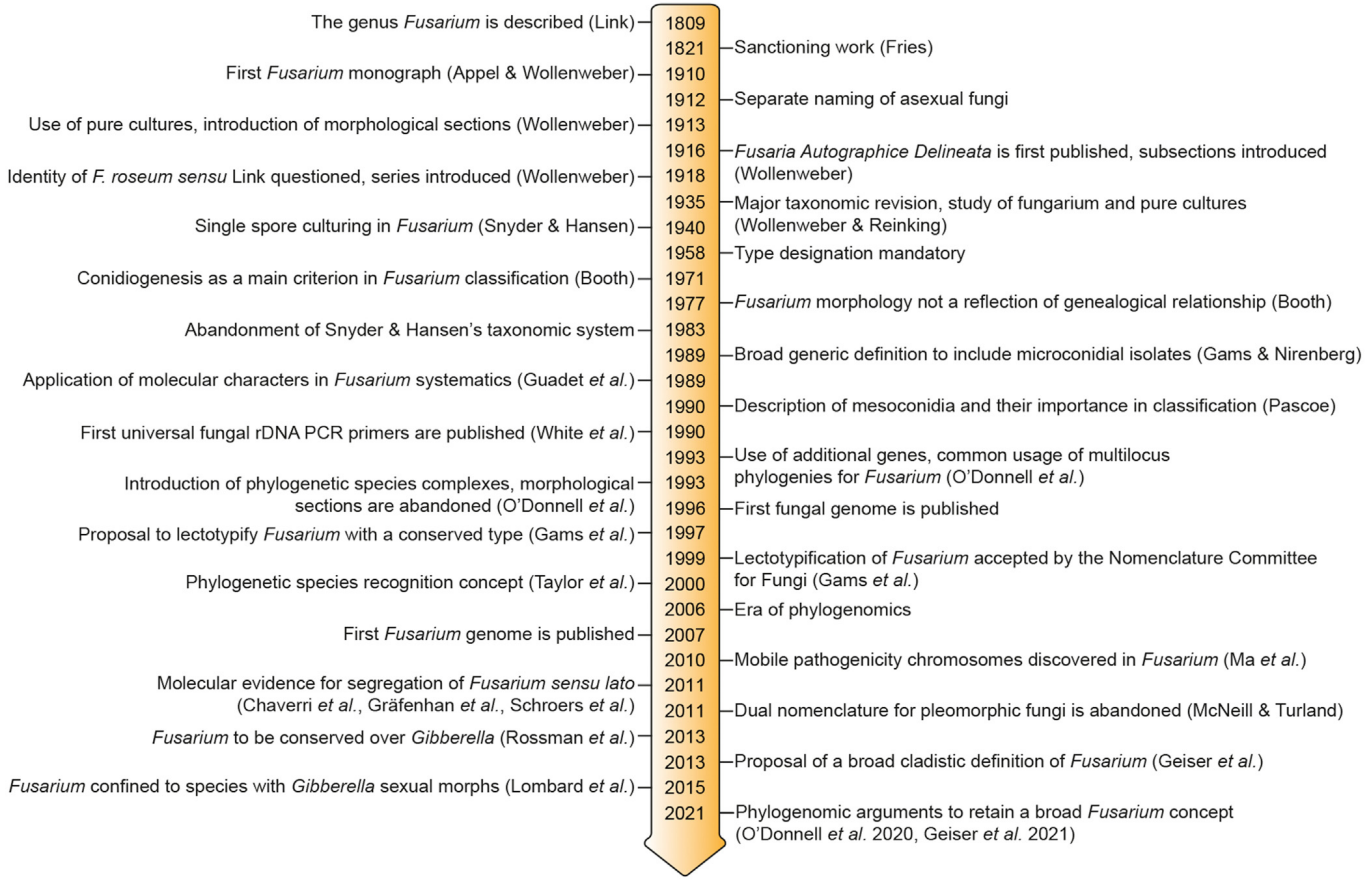
Timeline summarising important events in the taxonomy and nomenclature of *Fusarium* and related taxa.

The relevance and impact of *Fusarium* (*Ascomycota*, *Hypocreales*, *Nectriaceae*) to humankind is substantial. Over the past 100 years, it has attracted considerable attention from scientists as the extent of species diversity and the impact on agriculture and human health became clear. After an initial period of discovery and cataloguing by 19^th^ century naturalists, its taxonomy became the target of research from a broad range of scientists, that resulted in the emergence of distinct “schools” that promoted different taxonomic approaches to fusarium-like organisms. With the advent of an objective and reproducible framework for phylogenetic relationships inferred from molecular phylogenetics, it might have been expected that controversies would melt away, and a stable, universally accepted taxonomy of *Fusarium* and its species would emerge, but this does not yet appear to be the case (Fig. 1). However, all scientists working with *Fusarium* desire a stable taxonomic system, and all agree that taxonomic changes should be made with the aim of promoting stability.

Recently, Geiser *et al.* (2021), largely in response to papers published by Gräfenhan *et al.* (2011), Schroers *et al.* (2011), Lombard *et al.* (2015), and Sandoval-Denis *et al.* (2019), proposed a cladistic solution to redelimit a generic concept for *Fusarium*. The generic treatment of *Fusarium* by Geiser *et al.* (2013, 2021), produced an ill-delimited genus without clear synapomorphies, as fusarium-like macroconidia are strongly polyphyletic within *Nectriaceae* and also occur outside their very broadly circumscribed *Fusarium* concept. We argue that a narrower concept of genera with a clear, unique combination of features is needed for the majority of fusarioid species.

### Dual nomenclature and consensus on the use of the generic name *Fusarium*

In accordance with the single-name system for fungi, that was adopted at the International Botanical Congress, Melbourne (IBCM) in 2011, we are in full agreement with Geiser *et al.* (2013, 2021) and O'Donnell *et al.* (2020) that the name *Fusarium* applies to any genus with a delimitation that includes the conserved lectotype of the type species, *F. sambucinum* (sexual morph synonym *Gibberella pulicaris*), as stated by Rossman *et al.* (2013). Unfortunately, a single joint paper explaining the choice of this name supported by the entire *Fusarium* community was planned but failed because of the insistence of a subset of authors to adopt a broad generic concept.

Taxonomy and nomenclature are different concepts, although they are frequently confused, leading to misinterpretations. Support for dual nomenclature ended at the IBCM in August 2011. The significance of 1 January 2013 was to ensure the formal nomenclatural validity of newly proposed *dual* names (new species or new combinations) that were in press or part of studies about to be submitted for publication. These dates have no significance for names proposed in a single name system, which can be done at any time. Despite these technicalities, virtually all members of the *Fusarium* community accept that *Fusarium* must be used over the sexually-typified name *Gibberella* in the single name system, a recommendation included in the proposed list of Protected Names submitted to the Nomenclature Committee for Fungi, the body with the authority to recommend its formal acceptance (Kirk *et al.* 2013). However, statements in Geiser *et al.* (2013) seem to reflect a confusion about how the nomenclatural decision affected taxonomic concepts.

The name *Fusarium* has never been at risk during the nomenclatural transition, and the community support for its use in a single name system is unanimous. We fully agree with Geiser *et al.* (2013, 2021) and Rossman *et al.* (2013) that *Fusarium* equals *Gibberella*. *Fusarium* will always be applied to the clade that includes the type species of *Fusarium, F. sambucinum*, which is the same fungus that also typifies *Gibberella.* In this study, we show that the clade defined as *Fusarium s. str.* (O'Donnell *et al.* 2013*,* as *Gibberella;* Geiser *et al.* 2013, as Clade B) combines monophyly, morphology of sexual and asexual morphs, and biochemical data in a coherent way that can logically be recognised at the generic rank. Expanding the concept of *Fusarium* to node F1 *sensu* Geiser *et al.* (2013, 2021) results in the combination of several distinct genera and does not resolve the issue of fusarium-like macroconidia in genera outside their broad circumscription of *Fusarium.*

### Phylogenetic structure and distribution of fusarioid asexual morphs in *Nectriaceae* (*Hypocreales*)

Gräfenhan *et al.* (2011) and Schroers *et al.* (2011) presented a phylogenetic overview of selected *Nectriaceae* based on combined analyses of two different genes, namely the commonly employed and phylogenetically informative RNA polymerase II second largest subunit (*rpb2*) and exon regions of the larger subunit of ATP citrate lyase (*acl1*). The two papers were the first to apply a single name system to fusarioid fungi (*i.e.,* genera with fusarium-like macroconidia), and were written along with others (see Rossman & Seifert 2011) to promote discussions that eventually led to changes to the International Code of Nomenclature for algae, fungi, and plants (ICNafp) (Turland *et al.* 2018).

The main focus of the Gräfenhan *et al.* (2011) paper was to deal with extraneous elements that had long been included in *Fusarium.* These fungi had distinct phenotypic characters, such as thin, collapsing perithecial walls, slow growing agar colonies lacking aerial mycelium, or sparsely septate macroconidia. Users of the Gerlach & Nirenberg (1982) and Nelson *et al.* (1983) identification manuals may be familiar with some of these species, then called *Fusarium aquaeductuum, F. coccophilum* and *F. merismoides*. There was evidence in the first papers on the molecular phylogeny of *Fusarium* that these species did not belong to *Fusarium* (*e.g.,* see O'Donnell 1993). It was not until the study by Gräfenhan *et al.* (2011) that other genera in the family, such as members of the *Cylindrocarpon* generic complex (Chaverri *et al.* 2011), *Calonectria* (Liu *et al.* 2020), *Tubercularia* (Hirooka *et al. 2*012), and minor genera such as *Mariannaea, Pseudonectria*, and *Volutella* (also see Lombard *et al.* 2015) were adequately sampled to yield generic-level resolution*.* The phylograms showed the division of fusarioid taxa into two large groups, which Gräfenhan *et al.* (2011) called the Terminal Fusarium Clade (abbreviated TFC by Geiser *et al.* 2013) and the ill-delineated Basal Fusarium Clade (BFC) that contained several of the genera noted above. A single-genus recognition for the BFC was not feasible because of the great morphological, genetic, and ecological divergence among the sampled species. The BFC included seven genera, each with their monophyly strongly supported and more or less ecologically coherent. Species with fusarioid conidia were reclassified in the phylogenetically redefined but previously described genera *Atractium*, *Cosmospora*, *Dialonectria*, *Fusicolla*, *Macroconia*, *Microcera*, and *Stylonectria* (Gräfenhan *et al.* 2011, Schroers *et al.* 2011)*.* Geiser *et al.* (2013) accepted these segregate genera in the BFC as distinct from the TFC, while correctly pointing out the weak support values obtained for the phylogenetic backbone of the tree. One consequence of the widespread occurrence of macroconidia in the taxon sampling (fusarioid genera, cylindrocarpon-like genera, and *Calonectria*) was the suggestion that especially the fusarioid macroconidium is a plesiomorphic character (that is, an ancestral character) and had been lost in some lineages in *Nectriaceae* (Gräfenhan *et al.* 2011).

The second paper by Schroers *et al.* (2011) recovered similar phylogenies as Gräfenhan *et al.* (2011), but focused on the TFC, supplementing this with a five-gene analysis of a particular subclade within the TFC intended to delimit phylogenetic genera and a few species. This demonstrated the monophyly of the treated genera and resulted in the acceptance of the previously described *Cyanonectria* (Samuels *et al.* 2009), as well as the description of the genus *Geejayessia*. Again, Geiser *et al.* (2013) correctly criticised the weakness of the backbone of the tree, especially in the BFC. About 75 % of the phylogenetic signal in the analysis came from one gene, *rpb2*. Schroers *et al.* (2011) did not discuss the taxonomic fate of *Neocosmospora* (the *Fusarium solani* species complex, FSSC), which was represented by only two species in their analysis, but was excluded from *Fusarium s. str*.

The call for more genetic markers and even genome analysis by Geiser *et al.* (2013), to better resolve the phylogenetic backbone of the TFC was justified, but the increased number of markers should have been matched by increased taxon sampling of all known genera of *Nectriaceae*, as taxon sampling is equally important for inferring robust and meaningful phylogenies (Zwickl & Hillis 2002, Heath *et al.* 2008).

Lombard *et al.* (2015) greatly expanded both the number of genetic markers and the taxon sampling in order to explore the generic boundaries across the *Nectriaceae*, including all genera known from culture and many genera for which no DNA data was previously available. A 10-gene phylogeny was inferred including all the markers previously used by Gräfenhan *et al.* (2011), Schroers *et al.* (2011), Geiser *et al.* (2013), and O'Donnell *et al.* (2013), plus nrDNA sequences and other markers of known phylogenetic utility, namely actin (*act1*), beta-tubulin (*tub2*), calmodulin (*CaM*), histone (*his3*), and the translation elongation factor 1-α (*tef1*). From this, a phylogeny of the TFC overall congruent to that presented by Gräfenhan *et al.* (2011) and Geiser *et al.* (2013) was obtained. Importantly, the monophyly of *Albonectria, Cyanonectria, Geejayessia, Fusarium*, and *Neocosmospora* was reaffirmed and a few early diverging lineages previously included in the TFC were segregated into new fusarioid genera *i.e., Bisifusarium* (formerly the *F. dimerum s*pecies complex) and *Rectifusarium* (formerly the *F. ventricosum* species complex) (Lombard *et al.* 2015).

After nearly a hundred years of quandary, a modern revision was published for *Neocosmospora* (Sandoval-Denis *et al.* 2019), In this study, many unnamed phylogenetic species were morphologically characterised and given Latin binomials, while old names were resurrected, epitypified, and linked to DNA barcodes.

Two recent publications by O'Donnell *et al.* (2020) and Geiser *et al.* (2021) argued for the broad *Fusarium* concept of Geiser *et al.* (2013). Both papers present very similar phylogenetic analyses, relying on 19 genes, including 12 newly sampled markers, namely: cytochrome P450 reductase (*cpr1*), ATP-dependent DNA helicase II (*ku70*), sphinganine palmitoyl transferase subunit 2 (*lcb2*), DNA replication licensing factor (*mcm7*), phosphoglycerate kinase (*pgk1*), topoisomerase (*top1*), two subunits each of the DNA polymerase (*dpa1* and *dpe1*), the fatty acid synthase (*fas1, fas2*), alpha-tubulin (*tub1*), and *tub2*. The previously employed marker *his3* was not included, nor were nrDNA markers. The results are in essence the same as those of the previously published phylogenies, but with stronger support for the backbone in the combined analyses (see Cummings & Meyer 2005). Geiser *et al.* (2021) claimed that the F1 node was supported by 12, and the F2 node by 14 of the individual genes, but did not mention that all 19 genes supported the F3 node (*Fusarium s. str*. = the *Gibberella* clade).

In this study we re-investigated the Geiser *et al.* (2021) dataset using several different high-resolution phylogenetic approaches, and we found that their evaluations of concordance were based on an inadequate interpretation of Ultra-Fast bootstrap results (only values ≥ 95 % are to be deemed significant, see Minh *et al.* 2013, Hoang *et al.* 2018). In addition to the topological incongruences among six genes (*act1*, *CaM*, DNA polymerase epsilon subunit *dpe1*, *ku70*, *pgk1*, *tef1*, and *tub2*), only six and 11 genes actually support the F1 and F2 nodes, respectively, while all 19 genes support the F3 node. The low internode certainty (IC) and IC All (ICA) values obtained for F1 (0.19 and 0.33, respectively) were misinterpreted by Geiser *et al.* (2021) as IC values close to 0 indicate conflict between the partitions (Salichos *et al.* 2014). The F3 node was well supported with IC and ICA values at 1 (Geiser *et al.* 2021, Supplementary Table. S1), which indicates the absence of conflict.

While the effort by O'Donnell *et al.* (2020) and Geiser *et al.* (2021) to include a high diversity of DNA markers is commendable, it is undermined by an imbalanced selection of taxa for their analyses. Specifically, there is a marked overrepresentation of node F1 species, while sampling and taxon selection across the *Nectriaceae* is almost absent. Excluding any of the major genus-level clades, especially those relevant to the recognition of *Bisifusarium*, *Neocosmospora* and *Rectifusarium,* introduces taxon sampling biases in a way that reduce the reliability of phylogenetic inferences and support values with respect to the backbone of the *Nectriaceae*. Furthermore, neither O'Donnell *et al.* (2020) nor Geiser *et al.* (2021) give full consideration to morphological and ecological evidence. In principle, a genus should always be delimited as monophyletic, supported by derived traits. In addition, its circumscription should depend on the systematic (phylogenetic and biological) structure of the family it belongs to, in this case, the *Nectriaceae*.

Phylogenetics has rapidly advanced from a powerful adjunct tool for understanding evolutionary relationships to the dominant principle for classification, especially for delimitation of taxa at all ranks. However, the resulting analyses and phylogenies are compromised if they are not reconciled with other biological data. The call for additional genomic data in the *Fusarium* clade (Geiser *et al.* 2013, Aoki *et al.* 2019) may improve backbone node support values, but the phylogenetic structure is unlikely to change; it is the translation of that data into practicable taxonomy. The broad *Fusarium* concept of Aoki *et al.* (2019), O'Donnell *et al.* (2020) and Geiser *et al.* (2021) is phylogenetically possible, but it does not offer a generic definition based on a combination of available genetic, morphological, biochemical and ecological data. It is, thus, impractical in that it is so broad that the genus would not have any synapomorphies when compared to other genera of the *Nectriaceae* outside their broad circumscription of *Fusarium*.

The arguments presented by Aoki *et al.* (2019), O'Donnell *et al.* (2020) and Geiser *et al.* (2021) are centred around the phylogenetic support of some nodes, which have never been a key subject of the discussion, as the made observations generally match the interpretations made by many authors. While the very broad circumscription of *Fusarium* reflects as a monophyletic group in DNA phylogenetic analyses, the TFC is a conglomerate of several monophyletic genera that has a common ancestor (node F1 in Geiser *et al.* 2013). Each of these genera has a distinctive combination of morphological features. An analogous situation was observed in the monophyletic sister clade that was originally classified as *Cylindrocarpon s. lat.*, but that is currently viewed as composed of several monophyletic genera *i.e*., *Cinnamomeonectria*, *Corinectria*, *Cylindrodendrum*, *Dactylonectria*, *Ilyonectria*, *Macronectria*, *Neonectria*, *Pleiocarpon*, *Rugonectria*, *Thelonectria* and *Tumenectria* (Chaverri *et al.* 2011, Gräfenhan *et al.* 2011, Lombard *et al.* 2014, Salgado-Salazar *et al.* 2016, González & Chaverri 2017).

### What is a genus?

Taxonomically, a genus is a group that is defined by a type species, and that often includes additional species considered to belong to the same group (Vellinga *et al.* 2015). The observations or category of data involved in delineating genera have varied over time, and in many cases, the characters used to delimit well accepted genera have proven to be homoplasious and the genera polyphyletic (Crous *et al.* 2009). However, it is a fundamental principle that taxonomic entities should reflect evolutionary relationships.

This has led to inevitable splitting of well-known fungal taxa, both genera and species, into smaller groups, but sometimes also genera were merged with others based on the reappraisal or discovery of derived characters (*e.g.*, Voglmayr & Thines 2007). This proceeds with each technological revolution providing ever deeper insight into the biological/evolutionary relationships of organisms, and has accelerated again since molecular phylogenetics came into widespread use. There is a prevailing notion that nature made species, but that humans made all other taxonomic ranks for their own convenience. However, it is increasingly recognised that all taxonomic ranks, including the species level, do not have solid boundaries but are more like a steam cloud with fuzzy margins. At the genus level, these boundaries are often even more obscure, but is a genus just an arbitrary (but statistically well-supported) monophyletic convenience, a consensus accepted by a self-appointed committee? Or is a genus a meaningful, definable unit resulting from evolutionary processes, which can be recognised by patterns of biological structure, biochemistry, behaviour, and adaptation to specific niches? We believe that the latter should be the case. While we recognise that generic delimitations will always depend on a subjective choice, we believe that generic concepts should always be guided in a phylogenetic context by morphological, biochemical, or ecological characters that can both be used for practical recognition and convey evolutionary information.

The generic concept for *Fusarium* proposed by Geiser *et al.* (2013, 2021) is a rejection of this concept, as it merges lineages with divergent characters that were accepted and applied not only throughout the family *Nectriaceae* for the delimitation of genera but also in other fungal families and orders. The very broad genus *Fusarium* that it gives rise to does not have clear-cut features, as the diversity of characters shared with the rest of the *Nectriaceae* is so high that it could be extended almost arbitrarily to the entire family. It would, in fact be as if the concept of cryptic species was expanded to genera, that is, genera that can only be recognised as a well-supported node on a phylogram, which is, in our view, in disagreement with fundamental principles of practical classification. The node F1 selected by Geiser *et al.* (2013, 2021) for defining *Fusarium* is devoid of phenotypic support and includes several genera with distinct evolutionary traits. Indeed, the Geiser *et al.* (2013, 2021) concept of *Fusarium* is strictly phylogenetically defined and essentially amounts to a list of the species bound within a selected clade. Their morphological circumscription does not admit the existence of synapomorphies (*i.e.,* unique diagnostic characters possessed by all included species), and it extends beyond their chosen node to other groups in *Nectriaceae*. In this very wide definition of *Fusarium*, phenotypic characters and ecological patterns that correlate with well-supported monophyletic groups within the larger, poorly supported TFC are disregarded as basis for generic delineation.

Admittedly, phenotypic characters in the TFC are tricky to interpret. The fusarioid macroconidium with or without a well-developed foot-shaped basal cell (*i.e.,* basal conidial cell showing an asymmetrical papillum, delimited from the rest of the cell and forming a distinct notch) occurs in the majority but not all of the species in the traditional generic concept, but is also a feature present in a significant proportion of other members of the *Nectriaceae*, or even of the unrelated genus *Microdochium* (*Amphisphaeriaceae*). It is, therefore, not a unique feature for generic delineation (Gräfenhan *et al.* 2011).

Perithecial pigmentation has been used to delimit genera in *Nectriaceae*. The orange/red perithecium is an ancestral character in the family and common also to members of the BFC and early diverging lineages of the TFC, including all *Neocosmospora* species known to reproduce sexually, *Setofusarium*, and some species of *Cyanonectria* and *Geejayessia*. These structures are easily distinguished from the homogeneously bluish/black perithecia of true *Fusarium s. str.* species in the *Gibberella* clade *sensu* O'Donnell *et al.* (2013). Contrary to what was suggested by Geiser *et al.* (2021), it is not *Neocosmospora* which represents an interesting but morphologically aberrant lineage*,* since neither its type nor the members of its modern morphological circumscription (Nalim *et al.* 2011) exhibit aberrant characteristics. It is the dark-coloured perithecia typical of *Fusarium s. str.* (= *Gibberella* clade) that are aberrant and unusual within *Nectriaceae*.

The dark purple to black perithecium formerly used to characterise *Fusarium s. str*. (= *Gibberella*), represents a synapomorphic state. Ascomata with similar colours have evolved independently in some, but not all, species of *Geejayessia*, while heterogeneously coloured bluish black or bicoloured perithecia can be observed in several species of *Cyanonectria*, which often appears as a sister genus to *Fusarium*. However, *Cyanonectria* and *Geejayessia* differ from *Fusarium* and *Neocosmospora* by their typically well-developed stromata as well as their thinner and smooth perithecial walls. Notably, pale yellowish perithecia occur in several clades and are a derived character as well, and one genus that we accept, *Albonectria*, was initially defined by white perithecia (Rossman *et al.* 1999). Also, in terms of its ascospores, *Fusarium* shows a derived state. With the exception of *Albonectria*, which includes species with hyaline, ellipsoidal to fusoid, 3-septate, smooth to finely striated ascospores, the genera mentioned above present mostly pale yellow-brown ascospores. Ascospores of *Fusarium s. str.* are more often subhyaline, ellipsoidal to fusoid, 1–3-septate, and smooth-walled when viewed with light microscopy. Ascospores of *Neocosmospora* are easily distinguished from those of *Fusarium* by being ovoid to ellipsoidal, (0–)1-septate, pigmented, conspicuously striate or more rarely cerebriform or spinulose. It is worth noting that most of the above-mentioned characters and differences are the same applied to define genera across the whole *Nectriaceae* (Rossman *et al.* 1999, Lombard *et al.* 2015), where they correlate well with phylogenetic inferences. Ascospores showing similarly many septa as in *Fusarium s. str.* have independently evolved in *Nectria diploa* (now *Microcera*), as well as in *N. glabra*, and *N. decora* (now *Flammocladiella*). The fact that none of these species is a member of the TFC supports the interpretation that multiseptate ascospores might be apomorphic for *Fusarium s. str.*, separating it clearly from other phylogenetically related genera.

Behaviour and other adaptations, determine how an organism operates and survives in nature and are the ultimate determinants and products of natural selection. They may be difficult to translate into nodes and other results of phylogenetic analyses such as phylogenetic distance. Despite this, similarities in adaptive traits are frequently used to calibrate phylogenetic delimitations of genera. For example, all known species of *Microcera* are pathogens of scale insects. It is easy to understand the hypothesis that the ancestor of this clade jumped to these hosts, followed by subsequent radiation and speciation (Thines 2019). This resulted in considerable micromorphological diversity, while a core of adaptation resulting from the parasitic life style remained conserved. Similarly, several of the genus-level clades include mostly mycoparasitic species or pathogens of plants. If we apply this kind of thinking to the well-supported clades of the TFC, as noted by Schroers *et al.* (2011), species of *Cyanonectria* and *Geejayessia* occur only on woody hosts (mostly species of *Buxus, Celtis* and *Staphylea*) and would typically not occur as soil-borne plant pathogens or pathogens of grasses. They are also not known to produce trichothecene mycotoxins. This is in stark contrast with the prevailing ecological concept of *Fusarium s. str.* as a genus of primarily soil-borne fungi, of which many are in a firm biological association with grasses and herbs. Importantly, the vast majority of *Fusarium s. str*. species produce trichothecene mycotoxins as a chemical synapomorphy. Most of the strongly supported clades within the TFC can be supported by these kinds of morphological, chemical, and biological traits, allowing the possibility of non-arbitrary recognition of biologically meaningful genera. One such clade is *Neocosmospora*.

### Arguments for and the practicality of recognising *Neocosmospora* (the *F. solani* species complex) as a genus

In the days of dual nomenclature, the distinction between the red perithecia of *Neocosmospora*, as amended by Nalim *et al.* (2011), and the typically purple or blackish perithecia of the trichothecene-producing *Gibberella* species was generally accepted by *Fusarium* taxonomists. The ecological distinctiveness of *Neocosmospora* as a group of soil fungi, often associated with roots and causing root rot and vascular wilt diseases, was also generally acknowledged. In addition to the dissimilar sexual characters mentioned above, the asexual morphs of this group are also distinctive. The macroconidia are usually thick-walled, with blunt, rounded apical cells, and they usually have inconspicuous foot-shaped basal cells. Microconidia are produced on very long, narrow phialides. Cultures of a vast majority of species of this group can easily be recognised morphologically, even with a dissecting microscope.

The ecological similarities of the members of *Neocosmospora* with *F. oxysporum* have to be acknowledged, as noted by Geiser *et al.* (2013, 2021). However, these two groups of species are morphologically distinct, even as asexual morphs. *Fusarium oxysporum* produces macroconidia with acutely pointed apical cells, and microconidia from phialides that are usually 5–10 times shorter than those of *Neocosmospora* species.

Geiser *et al.* (2013, 2021) have pointed out that microchromosomes or conditionally dispensable chromosomes occur in *Neocosmospora* and members of their F3 clade, namely *F. oxysporum*. Microchromosomes have been observed, however, also in phylogenetically distinct taxa such as *Magnaporthe oryzae* (Yoshida *et al.* 2009, now *Pyricularia oryzae*), *Mycosphaerella graminicola* (Stukenbrock *et al.* 2010, now *Zymoseptoria tritici*), and *Alternaria arborescens* (Hu *et al.* 2012) and might occur sporadically as a result of horizontal gene transfer. They are thought to increase the ability of a pathogen to adapt to the host's defence mechanisms. The ability to acquire conditionally dispensable chromosomes might thus be seen as a general genetic tool allowing organisms to gain ecologically advantageous genes. Similarly, they could present a general driving force in co-evolutionary processes, but the per se occurrence of conditionally dispensable chromosomes in two taxa can hardly be used as a criterion for drawing conclusions on or imply generic relatedness.

In the Nelson *et al.* (1983) manual and in one of the last vestiges of the ultra-reductionist Snyder & Hansen (1941) system, *F. solani* was recognised as the only species of section *Martiella*, even though the existence of several distinct mating populations was known. The European system (exemplified by Gerlach & Nirenberg 1982) accepted several more species, derived from the classic Wollenweber & Reinking (1935) treatment. When molecular phylogenetic studies of this group began in earnest, *Neocosmospora* included three major clades and many species (O'Donnell 1993, 2000, O'Donnell *et al.* 2008a). To date, 86 species are formally described in this group (Aoki *et al.* 2019, Sandoval-Denis *et al.* 2019, Guarnaccia *et al.* 2021), but additional novel phylogenetic lineages are recognised and await formal description.

Thus, in *Neocosmospora* we have a group of species that can easily be recognised morphologically by both sexual and asexual morphs, exhibit generally consistent ecological behaviour, lack trichothecene mycotoxins, and form a strongly supported monophyletic group. This sounds like a biologically meaningful calibration of a genus, but what about the practicality of doing this? Presently, the data supporting the recognition of *Neocosmospora* (and equally, also *Fusarium s. str.*, the F3 clade) is stronger than the data supporting either of the nodes favoured for designating a broader concept of *Fusarium*. If there are 100 plus species in *Neocosmospora*, and hundreds of species in the trichothecene-producing, *Poaceae*-loving *Fusarium s. str.* clade, it will be useful for students, plant pathologists, clinical microbiologists, and other scientists to have different generic names for each group. Those names will convey information about relationships and behaviour that are lost in a broader definition of *Fusarium* with much greater diversity of ecological and biochemical behaviours. Geiser *et al.* (2013) raised concerns that grant evaluators, government regulators and medical practitioners who now believe they know what *Fusarium* means will be confused by the segregation of these fusarioid fungi into different genera, and that confusion could lead to unpredictable consequences. However, in our experience these end users continuously familiarise themselves with up-to-date, informative taxonomic and nomenclatural concepts for socio-economically important fungal groups, thus allowing them to predict the possible real-world effects of reliably identified fungi with increased precision. To them, the segregation of a heterogeneous concept of *Fusarium* into biologically and biochemically predictive genera will be helpful.

With *Neocosmospora* accepted as a different genus, *Albonectria, Cyanonectria*, and *Geejayessia,* as defined by Schroers *et al.* (2011), as well as *Bisifusarium* and *Rectifusarium* as defined in Lombard *et al.* (2015) must also be accepted as separate genera. As previously said, these are all monophyletic groups, also characterised by distinctive ecological and morphological traits.

The end consequence of our strategy is a series of phylogenetically well-supported genera, each with a recognisable suite of morphological characters, and ecological, pathological, and biochemical behaviour. Indeed, the results of such splitting activities applied to what we called the Wollenweber concept of *Fusarium s. lat.* accounts for 20 segregate genera. Most importantly, both *Fusarium* and *Neocosmospora* will have generic names to indicate their important but distinct significance. The extraneous species, with different ecology and generally much lower economic or agricultural significance can now justifiably be classified elsewhere, where they can be appreciated for their own features without the need for the uncertainty inherent in a broad concept of the generic name *Fusarium.*

The generic concept of *Fusarium* proposed by Geiser *et al.* (2013, 2021) functions well as a phylogenetic concept only if taxonomists turn their eyes away from all other kinds of data and observations applied to the family *Nectriaceae*. It is a political generic concept, meant to assuage the concerns of plant pathologists and other applied scientists, many of whom are already upset by the proliferation of cryptic phylogenetic species. Ironically, this late-blooming alleged pragmatism seems to betray the cladistic ideals that many of its authors profess to adhere to (Taylor 2014).

All authors agree on the use of the single name *Fusarium*, have a common understanding of a phylogenetic structure of the family *Nectriaceae*, and agree that removing *Neocosmospora* from the main *Fusarium* core is the critical point of discussion. Sequencing additional markers may lead to increased phylogenetic support, but it is a false comfort if the taxon sampling does not include as many genera of *Nectriaceae* as possible. Expanded representation of the TFC in the dataset will not solve the controversy, and the resulting phylogenies will remain unbalanced. The segregation of *Neocosmospora* from *Fusarium* certainly needs to be done efficiently by those who have the most comprehensive expertise on the relevant species, which include several of the co-authors of the Geiser *et al.* (2013, 2021) and O'Donnell *et al.* (2020) papers as well as the present one.

*Fusarium* taxonomy has long been confused because of the nine-species system of Snyder & Hansen (1940, 1941), the misleading overlaps caused by convergent evolution and character loss, the difficulty in characterising perithecia, the phenomenon of cultural degeneration, and rigid opinions of the taxonomists and plant pathologists who have worked on them. To arrive at a stable taxonomy for *Fusarium*, the generic concept needs to be fixed in a practical and evolutionary reasonable manner so that future technologies and applications will not disrupt it.

## Secondary metabolites of fusarioid genera

**Fig. 2:**
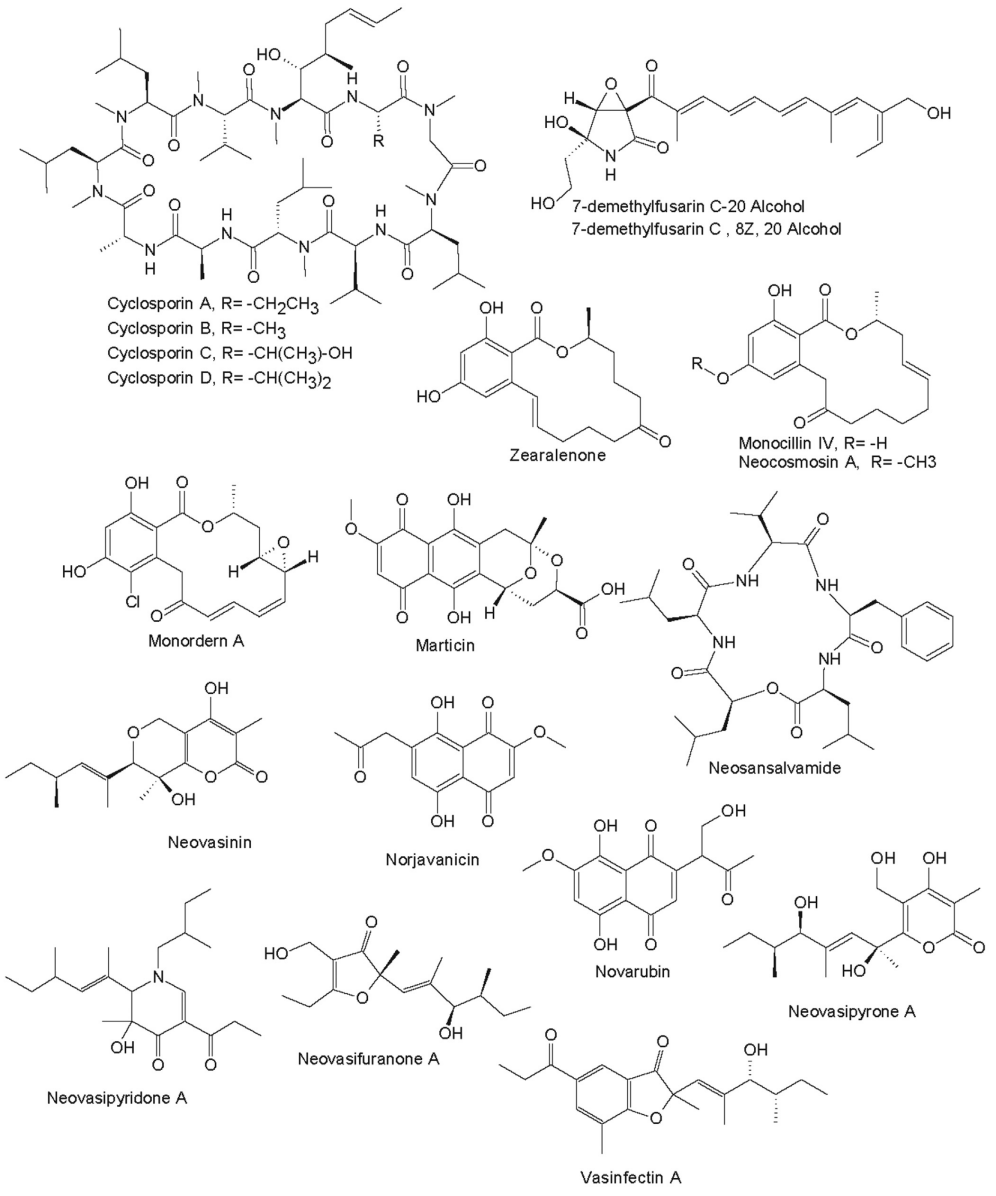
Secondary metabolites from *Fusarium* spp. / *Neocosmospora* spp.

**Fig. 3:**
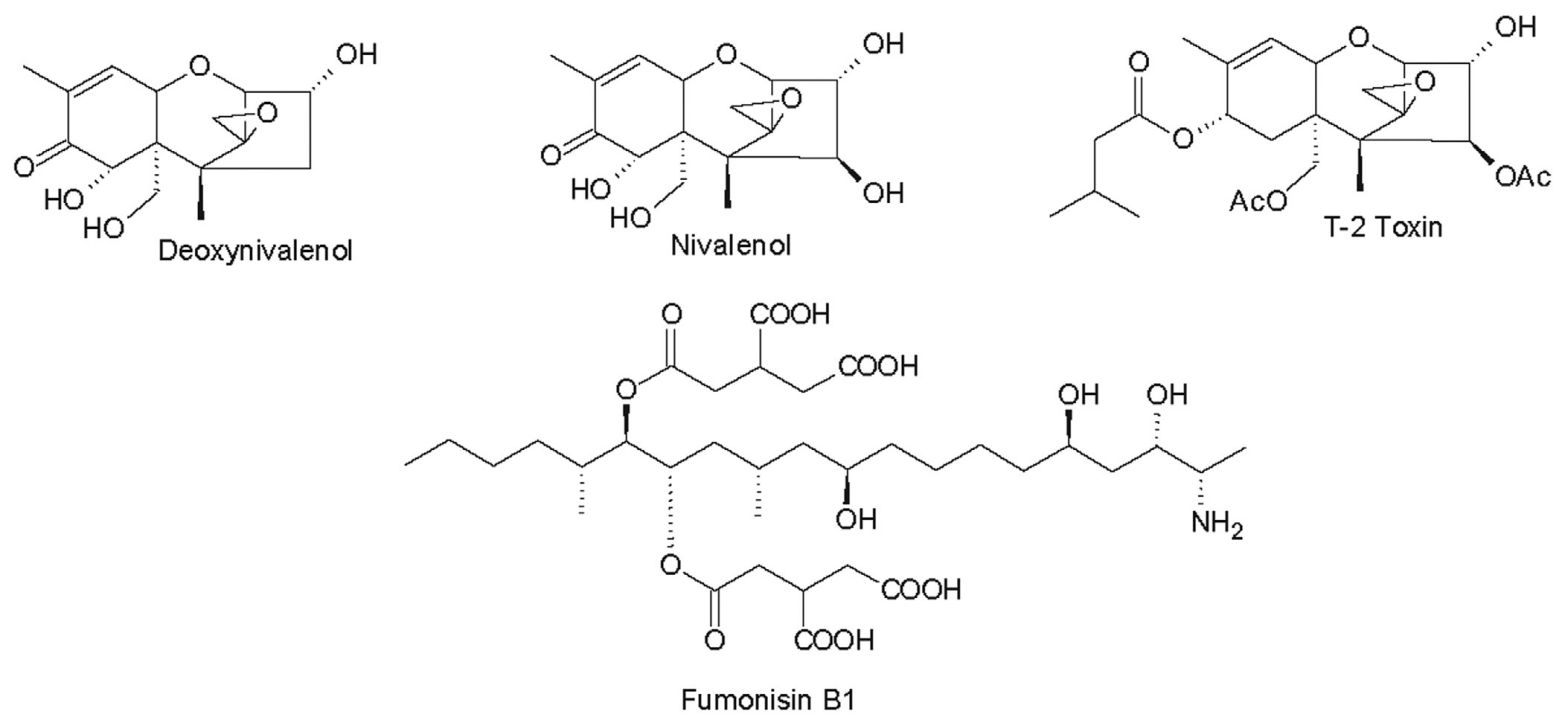
Some of the most important mycotoxins from *Fusarium* spp.

**Fig. 4:**
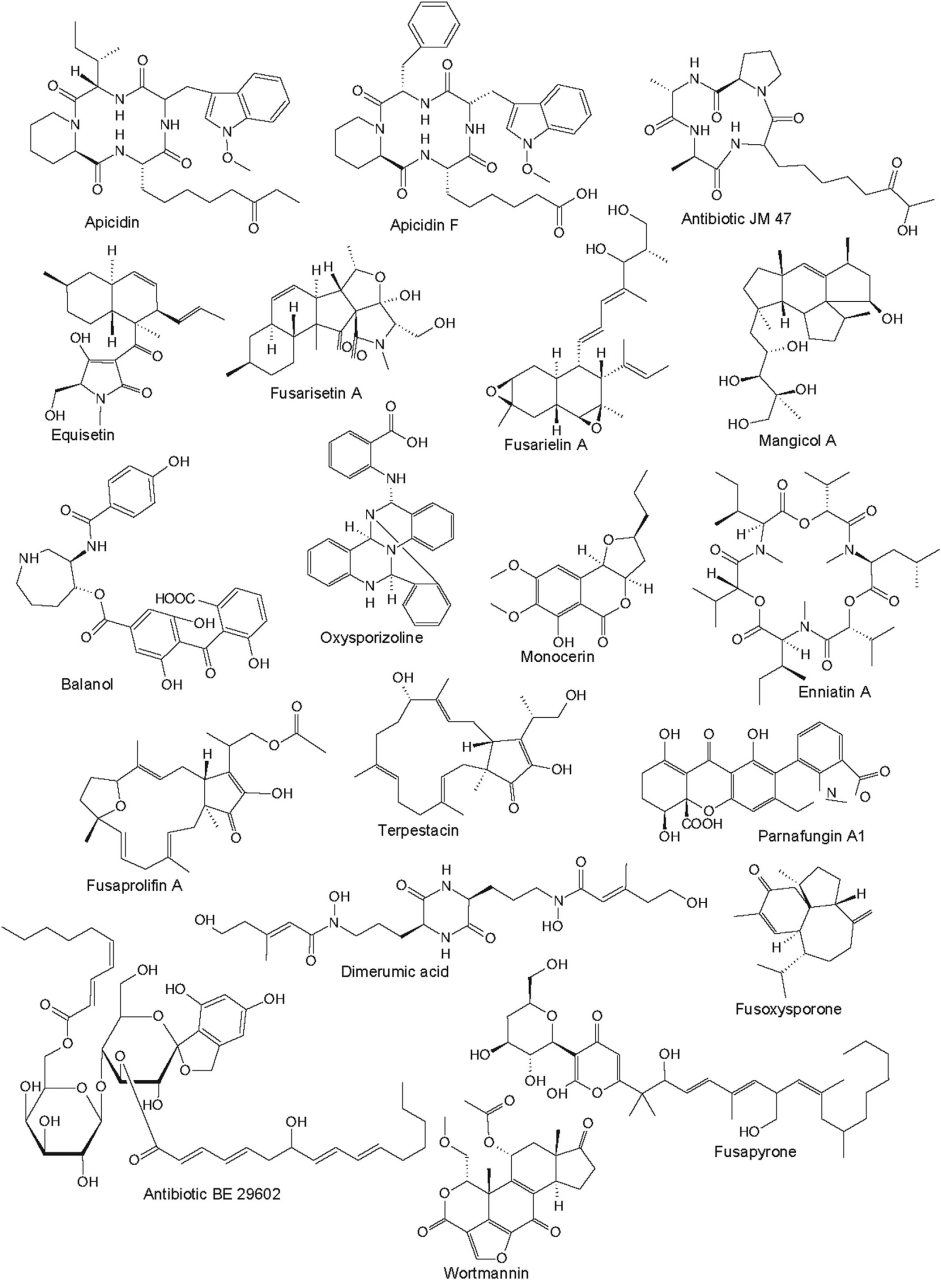
Secondary metabolites from fusarioid *Hypocreales*.

The phylogenetic distribution of the fusarioid genera presented here is further corroborated by their ability to produce genus-specific secondary metabolites. The commercial database Dictionary of Natural Products (DNP; http://dnp.chemnetbase.com), was used to search for secondary metabolites produced by the genera and species treated here. The database contained (as of March 6, 2021) over 720 entries on metabolites from *Fusarium s. lat.*, even though some plant metabolites, discovered during studies on the elicitation of phytoalexins by challenging plant cells with a *Fusarium* strain, are included. The number of metabolites from *Fusarium s. lat.* is therefore estimated to be around 680, which is still behind *Aspergillus s. lat.* (over 3 000 entries) and *Penicillium s. lat.* (over 2 700 entries). Hits that were retrieved were confirmed by consulting the original literature. The reported structures were corroborated, with a selection of these compounds presented here (Fig. 2, Fig. 3, Fig. 4).

It remains uncertain if the reported taxonomy is reliable, since the producer strains may have been misidentified or determined using one of many outdated taxonomic concepts. However, several compound classes have been encountered multiple times from the same species or species complex, and in some instances, the strains were identified by experts and/or sequenced later in phylogenetic studies (O'Donnell *et al.* 2018). The situation is further complicated by the fact that certain secondary metabolites have been given similar names, but represent different molecules. The name solaniol has been given to both a trichothecene (*Fusarium s. str*.) and a naphthoquinone (*Neocosmospora*), and the fusariumins represent four different secondary metabolites.

### Typical metabolites of *Fusarium s. str.*

*Fusarium sambucinum*, the type species of the genus, has not been studied in much detail, but among the 20 metabolites known from this species, several metabolites are ranked in the classes trichothecenes and enniatins. The trichothecenes represents a well-known and notoriously dangerous class of mycotoxins belonging to the scirpene terpenoid type. These compounds are widely distributed within the genus *Fusarium s. str*., including familiar plant pathogenic species such as, *F. culmorum*, *F. graminearum*, *F. sporotrichioides* and *F. tricinctum* (Bamburg *et al.* 1968, Tatsuno *et al.* 1968, Yoshizawa & Morooka, 1973, Jiménez *et al.* 1997). The enniatins, known from 17 *Fusarium s. str*. species (Munkvold 2017, O'Donnell *et al.* 2018), are cyclic depsipeptides that have strong antibiotic activities (Plattner *et al.* 1948, German-Fattal 2001, Bills & Gloer 2017). Similar to trichothecenes, they are only known from *Fusarium s. str.* in the current taxonomic concept, although *Trichoderma* and *Beauveria*, which belong to different families of the *Hypocreales*, also produce trichothecenes or enniatin-like beauvericins, respectively. However, trichothecenes have not been reported from *Neocosmospora* or “*F. solani*” except from two isolates misidentified as “*F. solani”* (Ueno *et al.* 1972, Sugimoto *et al.* 2002) (Supplementary Table S2)

Two other well-known classes of mycotoxins, the fumonisins (Bezuidenhout *et al.* 1988) and zearalenone (Urry *et al.* 1966), are also found frequently among species of *Fusarium s. str*. Similarly, equisetin, also considered a “mycotoxin” and originally found from a *Fusarium* sp. strain (NRRL 5537) in the FIESC (Vesonder *et al.* 1979, Xia *et al.* 2019) is actually a strong antibiotic. A more complex derivative known as fusarisetin A was reported from an unidentified *Fusarium* sp. (Jang *et al.* 2011). Some rather unique compounds only known from *Fusarium s. str.*, include wortmannin (Abbas & Mirocha, 1988) and oxysporizoline (Nenkep *et al.* 2016), which have interesting biological activities and may be species or even strain-specific.

Among the compounds that are not regarded as mycotoxins, the antimicrobial sesquiterpenes of the fusarielin type (Sørensen *et al.* 2013) and the antiparasitic and cytostatic cyclopeptides of the apicidin type (Jiang e*t al*. 2002, Von Bargen *et al.* 2013) have been respectively isolated from *Fusarium s. str*. Additionally, aurofusarin (Munkvold 2017, O'Donnell *et al.* 2018), chlamydosporol (Munkvold 2017, O'Donnell *et al.* 2018), fusapyrone (Evidente *et al.* 1994), fusaric acid (Munkvold 2017, O'Donnell *et al.* 2018), fusoxysporone (Abraham & Hannsen 1992), fusaproliferin, moniliformin (Munkvold 2017, O'Donnell *et al.* 2018) and the terpestacins (Liu *et al.* 2013) are other examples of secondary metabolites found only in *Fusarium s. str*. Thus far, only one report has indicated that a *Neocosmospora* species can produce fusaric acid (Zhou *et al.* 2019). Both aurofusarin and bikaverin produced by *Fusarium s. str*. and other bis-naphthoquinone and bis-naphthopyrone pigments protect fungi from predation (Xu *et al.*, 2019), while *Neocosmospora* species produce other naphthoquinones such as javanicin (Arnstein & Cook 1947, Kimura *et al.* 1981) as potential predator protectors. Some unique compounds have been reported from marine strains of certain *Fusarium* species, which include the mangicols, rare sesterterpenes produced by a strain tentatively classified as *F. heterosporum* (Renner *et al.* 2000).

### Typical metabolites of *Neocosmospora* and other fusarioid genera

*Neocosmospora* species and other fusarioid genera apparently have a different secondary metabolism, or have not been intensively studied in the past. A striking example are the cyclosporins, which are immunosuppressive peptides. Originally, these were obtained from *Tolypocladium inflatum*, but later also found to be produced by species of *Neocosmospora* (Sawai *et al.* 1981, Nakajima *et al.* 1989). However, they have not been reported from *Fusarium s. str*. Other unique compounds only known from *Neocosmospora* species, include dihydrofusarin (Kurobane *et al.* 1980, Kyekyeku *et al.* 2017), the polyketides neovasipyrones (Furumoto *et al.* 1995, Nakajima *et al.* 1995) and vasinfectin A (Furumoto *et al.* 1997). The rare cyclopeptides of the neosansalvamide type (Lee & Lee 2012) and the resorcylic acid lactones of the monorden/monocillin type (Cutler *et al.* 1987, Gao *et al.* 2013) are also known from *Neocosmospora* and other fungi, but not from *Fusarium s. str.,* even though the latter compounds bear a high structural resemblance to zearalenone. Several *Neoscomospora* species produce a range of naphthoquinones that are members of a widespread class of polyketides (Roos 1977).

The fusarioid genus *Bisifusarium* is known to produce the PKS/NRPS hybrid siderophore, dimerumic acid (= dimerum acid) (Diekmann 1970), and indole acetic acid (Reddy & Reddy 1992, Kulkarni *et al.* 2011, 2013). The parnafungins, which are under development as antimycotics, are only known from *Microcera larvarum* (Parish *et al.* 2008)*.* Additionally, *Microcera larvarum* is also known to produce monocerin and fusarentins, which are not known from any other fungi (Grove & Pople 1979), except a *Colletotrichum* species (Tianpanich *et al.* 2011). The anticancer agent balanol (azepinostatin) (Ohshima *et al.* 1994) is known to be produced by two *Fusicolla* species, which might be applied as a taxonomic marker for this genus, although it has also been found in species of the *Ophiocordycipitaceae*. Unfortunately, there is no available information on secondary metabolites for the other fusarioid genera treated here. However, secondary metabolite studies of these missing genera will facilitate for the discovery of novel molecules and help to elucidate the functional biodiversity of these fungi.

## Recommended methods for the identification and characterisation of *Fusarium* and allied genera

The following part of this study presents an overview of the morphological and phylogenetic characters of *Fusarium* and related genera as well as an account of recommended methods for the identification and characterisation of these taxa. In addition, novel genera and species are described and, in view of the recent taxonomic data, a list of names that are applied to the genus *Fusarium s. lat.* with their current scientific names is presented.

### Morphology

**Fig. 5:**
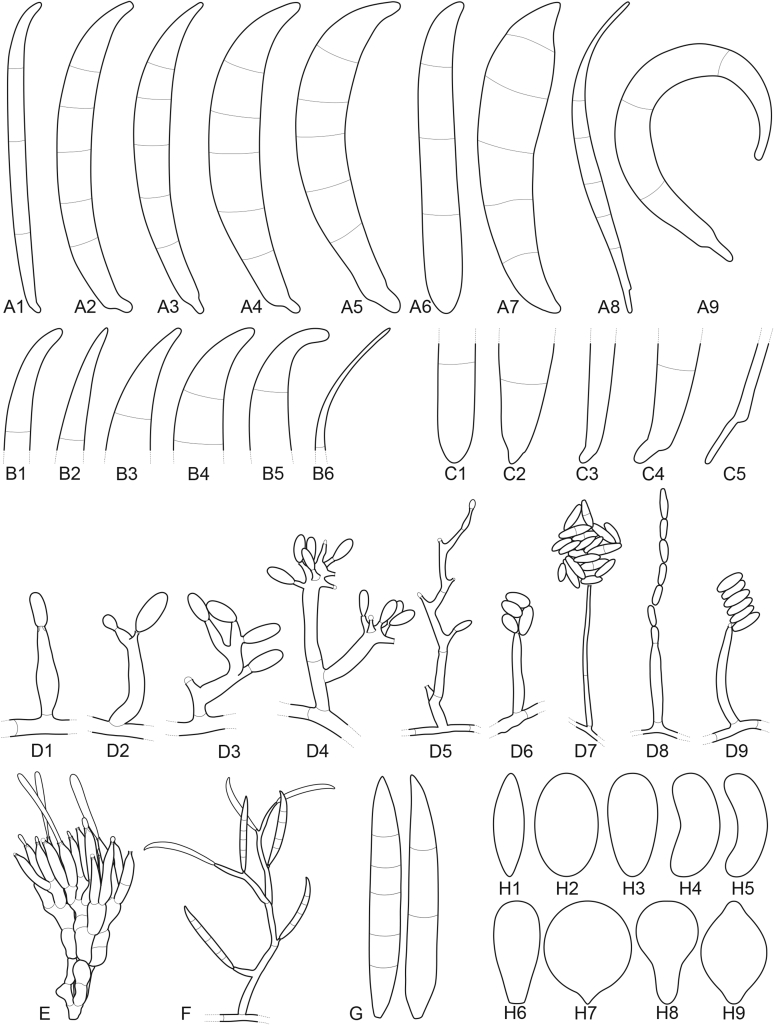
Basic morphological features of fusarioid fungi. **A.** Macroconidial shapes. A1. Slender with no significant curvature. A2. Curved with parallel walls. A3. Unequally curved. A4. Widest at the middle portion. A5. Widest at the apical third, wedge-shaped. A6. Widest at the basal portion. A7. Irregularly clavate and swollen. A8. Elongate, whip-like. A9. Distinctly curved. **B.** Macroconidial apex. B1. Curved. B2. Long and tapered. B3. Pointed. B4. Blunt. B5. Hooked. B6. Elongated. **C.** Macroconidial base. C1. Obtuse, non foot-shaped. C2. Papillate, non foot-shaped. C3. Poorly developed, foot-shaped. C4. Well-developed, foot-shaped. C5. Elongate, foot-shaped. **D.** Aerial phialides and microconidial organization. D1. Monophialide. D2–D5. Polyphialides. D2. Simple polyphialide. D3–D4. Polyphialides with multiple conidiogenous loci. D5. Sympodially proliferating polyphialides. D6, D7. Microconidia forming false heads. D8, D9. Microconidia in chains (D8. Dry chain. D9. Palisade). **E.** Sporodochial conidiophore and conidiogenous cells. **F.** Aerial conidiophore bearing mesoconidia. **G.** Mesoconidia. **H.** Microconidial shapes. H1. Fusiform. H2. Oval. H3. Obovoid. H4. Reniform. H5. Allantoid. H6. Clavate. H7. Napiform. H8. Pyriform. H9. Limoniform.

Current *Fusarium* taxonomy is dominated by molecular phylogenetic studies. Nonetheless, morphology is a fundamental component of the generic and species concepts of fungi and must not be overlooked. Key morphological features for generic circumscription include characteristics of sexual morphs such as perithecial colour, wall thickness and anatomy, surface structures and the presence and nature of a basal stroma, ascospore shape, septation, colour and surface ornamentation (Rossman *et al.* 1999). Classification of taxa solely based on their asexual morphs can be trickier than integrated systems using sexual and asexual characters. However, the general shapes, different types and combinations of conidiogenous structures and conidia present in culture can be sufficient to allow a preliminary identification (Fig. 5), especially if host data are also available (Leslie & Summerell 2006). For species-level characterisation, a number of morphological traits must be carefully studied, particularly those of the asexual morph, while sexual morphs are generally less suitable, especially as they are typically not produced in culture. Diagnostic characters for species identification include colony characters such as colony morphology, pigmentation, and type of aerial mycelium. Also included are the dimensions and characteristics of aerial conidiophores and conidiogenous cells (mono- *vs* polyphialides), presence/absence and characteristics of sporodochia, the types of conidia produced, *e.g.,* aerial microconidia, mesoconidia, and aerial and sporodochial macroconidia. In examining conidia themselves, consideration is given to the overall shape, septation and curvature of the macroconidia, as well as characteristics of their apical and basal cells; with aerial microconidia, their dimensions, shape, septation and spatial organisation (forming slimy heads, chains or a combination of both) are noted. Finally, the presence or absence of chlamydospores may be important.

### Culture media and incubation

**Fig. 6:**
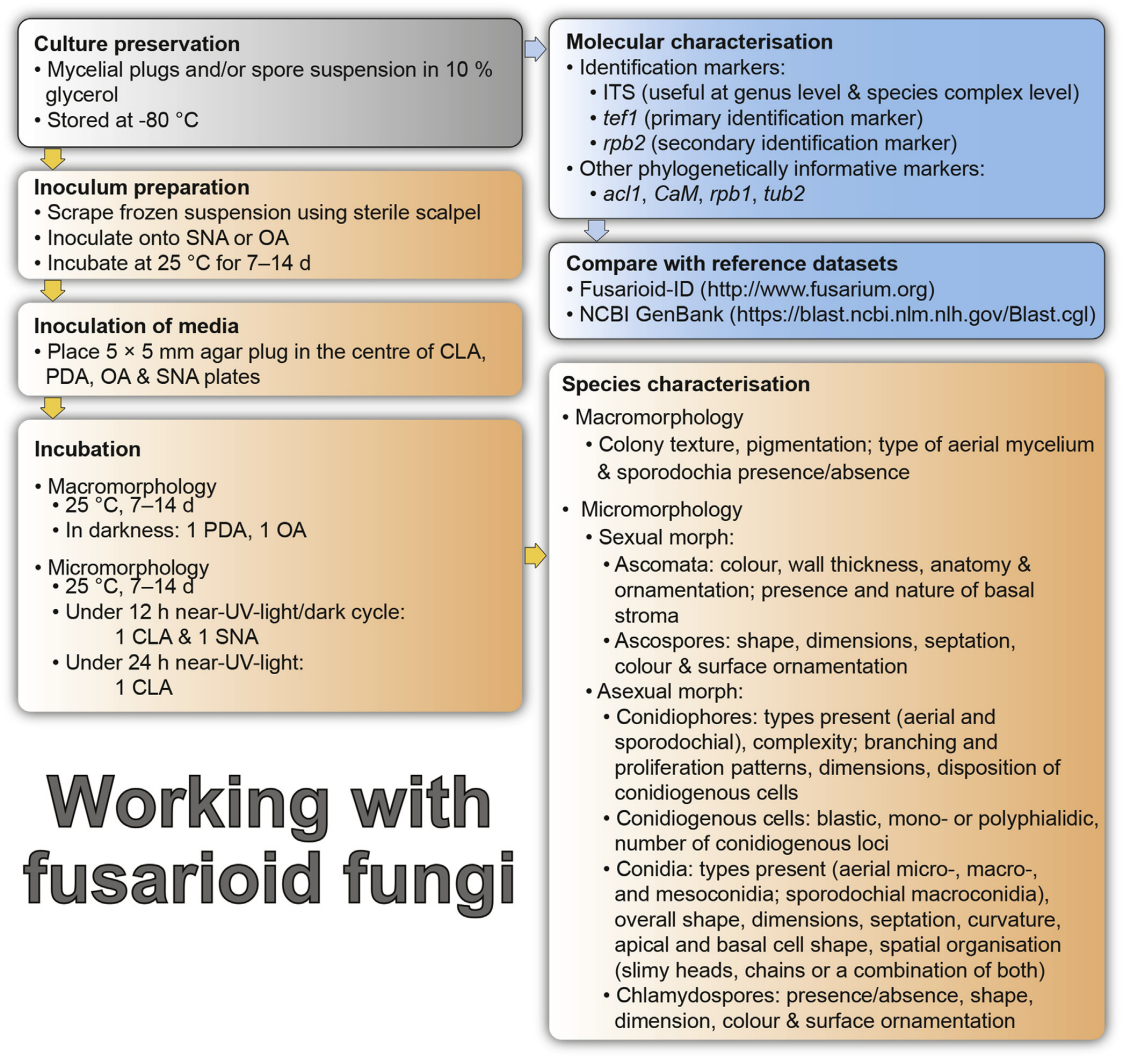
Flow diagram summarising recommended methods for the preservation, identification, and characterisation of fusarioid fungi.

Vigorous growth, sporulation, and pigment production of fusarioid fungi can be achieved on numerous agar formulations. The morphology of fungal structures will vary dramatically depending on the selection of media and growth conditions which may compromise the identification process. In addition, it is also common for fusaria to degenerate and lose viability in culture, particularly when they are grown on nutrient-rich media (Nelson *et al.* 1983, Nirenberg 1990, Summerell *et al.* 2003, Leslie & Summerell 2006). Culture conditions and media have been extensively summarised in the literature (Booth 1971, Nirenberg 1990, Nelson *et al.* 1994, Summerell *et al.* 2003, Leslie & Summerell 2006). Consequently, we recommend the agar formulations listed in Table 1 to be employed for the isolation and description of fusaria. A summary of the procedures and conditions suitable for work with fusarioid fungi is shown in Fig. 6.

**Table 1:** Recommended agar media formulations for the isolation and cultivation of fusaria.

Agar media	Components^1^		Preparation^2^	Incubation^3^	Application	Reference
Carnation leaf agar (CLA)	Sterilised carnation leaves		Carnation leaves are cut into approximately 5 × 5 mm pieces and dried at 60 °C for 24 h; sterilise by gamma radiation or autoclave; place 3–5 pieces on nearly solid 2 % WA surface.	25 °C;	Micro-morphological characterisation: formation of sporodochia; sporodochial macroconidia	Fisher *et al.* (1982), Crous *et al.* (2019a)
	WA			7–14 d under 12 h near-UV-light/dark cycle; 7–14 d under 24 h near-UV-light		
Selective Fusarium Agar (SFA)	Glucose (Dextrose)	20 g	Add all components, except antibiotics, to water and autoclave; cool to 45–50 °C and add antibiotics. Dichloran can be replaced by PCNB (0.75 g).	25 °C; 7–14 d in dark	Selective isolation of fusaria from soil	Tio *et al.* (1977), Leslie & Summerell (2006)
	KH_2_PO_4_	1 g				
	NaNO_3_	2 g				
	MgSO_4_·7H_2_O	0.5 g				
	Yeast Extract	1 g				
	1 % FeSO_4_·7H_2_O (aquous)	1 ml				
	Streptomycin	(5 % w/v) 20 mL				
	Neomycin	(1 % w/v) 12 mL				
	Dichloran	(50 % w/v in ethanol) 13 mL				
	Agar	20 g				
	Water	1 000 mL				
Komada's Medium	D-Galactose	20 g	Add all components, except antibiotics, oxgall and borax; to water and autoclave; cool to 45–50 °C and add the reamining components. Adjust pH to 3.8 ± 0.2 prior to autoclaving.	25 °C; 7–14 d in dark	Selective isolation of fusaria from soil, particularly those belonging to the Fusarium oxysporum species complex. Other fusaria can be inhibited by this medium	Komada (1975), Leslie & Summerell (2006)
	L-Asparagine	2 g				
	KH_2_PO_4_	1 g				
	KCl	0.5 g				
	MgSO_4_·7H_2_O	0.5 g				
	PCNB	0.75 g				
	Fe3Na EDTA	0.01 g				
	Streptomycin	(5 % w/v) 6 mL				
	Oxgall stock solution	0.5 g				
	Na_2_B_4_O_7_·10H_2_O (borax)	0.5 g				
	Agar	15–20 g				
	Water	1 000 mL				
Malachite Green Agar (MGA)	Peptone	15 g	Add all components, except antibiotics, to water and autoclave; cool to 45–50 °C and add antibiotics. Penicillin can be also replaced by chloramphenicol (5 % w/v) or neomycin (1 % w/v).	25 °C; 7–14 d in dark	Selective isolation of fusaria from soil and plant material, with improved inhibition of non-fusarioid contaminants	Castellá *et al.* (1997), Leslie & Summerell (2006)
	KH_2_PO_4_	1 g				
	MgSO_4_·7H_2_O	0.5 g				
	Malachite green oxalate	2.5 mg				
	Streptomycin	(5 % w/v) 20 mL				
	Penicillin	(5 % w/v) 20 mL				
	Agar	20 g				
	Water	1 000 mL				
Oatmeal agar (OA)	Oatmeal extract	1 000 mL	Oatmeal flakes (30 g/L) are wrapped in cloth and simmered in water for 2 h; liquid is squeezed and filtered through cloth.	25 °C; 7–14 d in dark	Macro-morphological characterisation, colony characteristics	Crous *et al.* (2019a)
	Agar	15–20 g				
	
Potato dextrose agar (PDA)	Potato extract	230 mL	Potatoes (5 kg; peeled and sliced) are minced; soak in water (300 mL/100 g potato) overnight at 4 °C; filter through cloth; adjust pH to 6.6.	25 °C; 7–14 d in dark; 5–40 °C (5 °C increments for growth curves)	Inoculum preparation, macro-morphological characterisation: colony characteristics; growth curve	Crous *et al.* (2019a)
	Agar	15–20 g				
	Water	770 mL				
Peptone Pentachloronitrobenzene (PCNB) agar (PPA)	Peptone	15 g	Add all components, except antibiotics, to water and autoclave; cool to 45–50 °C and add antibiotics. Penicillin can be also replaced by chloramphenicol (5 % w/v) or neomycin (1 % w/v).	25 °C; 7–14 d in dark	Selective isolation of fusaria from soil and plant material	Nash & Snyder (1962), Booth (1971), Leslie & Summerell (2006)
	KH_2_PO_4_	1 g				
	MgSO_4_·7H_2_O	0.5 g				
	PCNB	0.75 g				
	Streptomycin	(5 % w/v) 20 mL				
	Penicillin	(5 % w/v) 20 mL				
	Agar	20 g				
	Water	1 000 mL				
Rose Bengal-Glycerine-Urea Medium (RbGU)	Glycerol	10 g	Add all components, except antibiotics, to water and autoclave; cool to 45–50 °C and add antibiotics.	25 °C; 7–14 d in dark	Isolation of fusaria from soil and plant material	van Wyk *et al.* (1986), Leslie & Summerell (2006)
	Urea	1 g				
	L-Alaninw	0.5 g				
	PCNB	1 g				
	Rose Bengal	0.5 g				
	Streptomycin	(5 % w/v) 20 mL				
	Agar	15 g				
	Water	1 000 mL				
Synthetic nutrient-poor agar (SNA)	KH_2_PO_4_	1 g	Add all components to water and autoclave.	25 °C; 7–14 d under 12 h near-UV-light/dark cycle	Inoculum preparation, micro-morphological characterisation: aerial conidiophores and micro- & macroconidia; chlamydospore formation	Nirenberg (1976), Crous *et al.* (2019a)
	KNO_3_	1 g				
	MgSO_4_·7H_2_O	0.5 g				
	KCl	0.5 g				
	Glucose	0.2 g				
	Saccharose	0.2 g				
	Water	1 000 mL				
Water agar (WA)	Agar	15–20 g		25 °C; 7–14 d in dark	Inoculum preparation, base agar for CLA	Crous *et al.* (2019a)
	Water	1 000 mL				

1 Unless specified differently, antibiotic stock solutions are prepared in distilled water.

2 Water refers to distilled water; autoclave = 121 °C for 15 min.

3 Near-UV = near ultraviolet spectrum (wavelength 320–400 nm).

An important condition that must be stressed is that the identification must always be made on the basis of a monosporic culture (a culture produced from a single sporulating conidium, ascospore, or hyphal tip), as multiple species are commonly found to co-occur in the same substrate tissue. A freshly isolated fusarioid strain should be sub-cultured onto at least two different culture media, a relatively rich one suitable for examination of gross morphology, and a nutrient-poor one for micromorphological examination and for further culture propagation. The standard culture setup for initial assessment of growth rates and colony characters *i.e*., colony pigmentation, diffusible pigments, and colour of sporodochia, is to use potato dextrose agar (PDA) incubated for 1–2 wk. *Fusarium* and related genera will also grow and sporulate well on malt extract agar (MEA, recipe in Crous *et al.* 2019a), which can be a suitable alternative for initial isolation and monosporic cultivation. However, MEA should not be used to assess colony or morphological characters. Standard incubation is commonly made in total darkness; however, exposure to light will normally result in a faster and more intense pigmentation. We have observed better colour formation using in-house prepared media rather than commercial formulae. While colony colour cannot be employed as a primary criterion for species identification, it can provide useful means to grossly distinguish related groups and to direct the identification process towards determining genera or species complexes. The high nutrient content of these agar media strongly affects sporulation, commonly resulting in the development of atypical structures. Therefore, we strongly discourage the use of PDA for micromorphological assessment or culture propagation of *Fusarium* spp. (Nelson *et al.* 1994, Summerell *et al.* 2003). Oatmeal agar (OA) is a suitable alternative for strain sub-culturing, allowing for good sporulation with reduced strain degeneration; however, it is not recommended for micromorphological studies.

Carnation leaf agar (CLA), synthetic nutrient-poor agar (SNA), and water agar (WA) are the standard culture media for micromorphological analyses. Also, by reducing culture degeneration, they allow for prolonged storage of actively growing cultures (Nirenberg 1976, Nelson *et al.* 1983, Leslie & Summerell 2006). Subcultures on CLA will normally produce abundant sporodochia and macroconidia on the surface or around the carnation leaf pieces with consistent morphological features. Incubation at room temperature (20–25 °C) for 1–2 wk under a 12/12 h near-UV light (wavelength 320–400 nm)/dark or near-UV light/cool fluorescent light cycles results in stronger sporulation and good development of sporodochial pigmentation (Nirenberg 1990, Seifert 1996, Summerell *et al.* 2003, Leslie & Summerell 2006). The use of continuous near-UV light (also commonly termed "blacklight" or UV-A light) is also suitable although it often results in the formation of unusually long macroconidia (Nirenberg 1990), and it can suppress the development of useful morphological characters such as the globose microconidia of *Fusarium globosum*. Nevertheless, incubation under near-UV light is fundamental since isolates of some species such as *Fusarium poae* and *F. sacchari* are known to lack macroconidia or to produce them in only small quantities unless they are stimulated by incubation under a near-UV light source (Leslie *et al.* 2005, Leslie & Summerell 2006). *Fusarium* cultures also need adequate aeration to produce conidia reliably and to attain stable growth rates, and hence we discourage the incubation of sealed plates. Carnation leaf agar, SNA, and WA are also suitable for the observation of conidiophore disposition and microconidial arrangements such as the formation of false heads, chains or both. These structures can easily be examined under a dissecting microscope or at low magnification under a compound light microscope (Leslie & Summerell 2006). Examination of micromorphological characters must be carried out using slide preparations mounted in water. Lactic acid, lactophenol and Shear's mounting media can cause considerable shrinking of the structures and can alter the appearance of the cell surface; hence we advise against the use of these mountants for examination of morphological characters in *Fusarium* and related genera.

Additional culture media, incubation conditions, and protocols are available for induction of sexual characters in *Fusarium* and related genera (Klittich & Leslie 1988, Leslie & Summerell 2006, Guo *et al.* 2018, Kim *et al.* 2019, Santos *et al.* 2019). Carrot agar (CA) and half-strength CA are the most commonly used media. The crossing procedures are often variations from the protocol of Klittich & Leslie (1988), in which strains of opposite mating types are paired in all possible combinations as male and female parents, together with crosses made against tester strains from known mating populations (Leslie & Summerell 2006). The process can be shortened by reducing the number of combinations to be crossed by first determining the *MAT* gene alleles carried by each strain by means of specific mating type idiomorph PCR primers (Kerényi *et al.* 1999, 2004, Steenkamp *et al.* 2000).

### Molecular studies

Several genes, primer combinations and PCR conditions have been listed in the *Fusarium* literature (O'Donnell *et al.* 1998a, b, 2000a, b, O'Donnell et al., 2007, O'Donnell et al., 2010, O'Donnell et al., 2013, Gräfenhan *et al.* 2011, Lombard *et al.* 2015, 2019a, b), including whole-genome sequencing to mine for the desired genes (O'Donnell *et al.* 2020, Geiser *et al.* 2021). Here we detail those DNA markers that have shown the best results in routine diagnosis (Table 2, Fig. 6).

**Table 2:** Recommended PCR primers for DNA amplification of *Fusarium* and related genera.

Gene/DNA region		Primer			
Name	Abbreviation	Name	Direction	Sequence (5'→3')	Reference
28S large subunit of the nrDNA	LSU	LR0R	Forward	ACCCGCTGAACTTAAGC	Vilgalys & Sun (1994)
		LR5	Reverse	ATCCTGAGGGAAACTTC	Vilgalys & Hester (1990)
		NL4^2^	Reverse	GGTCCGTGTTTCAAGACGG	Kurtzman & Robnett (1997)
ATP citrate lyase	*acl1*	230up	Forward	AGCCCGATCAGCTCATCAAG	Gräfenhan *et al.* (2011)
		1220low	Reverse	CCTGGCAGCAAGATCVAGGAAGT	Gräfenhan *et al.* (2011)
Beta-tubulin	*tub2*	T1	Forward	AACATGCGTGAGATTGTAAGT	O'Donnell & Cigelnik (1997)
		TUB-2Fd^2^	Forward	GTBCACCTYCARACCGGYCARTG	Woudenberg *et al.* (2009)
		TUB4RD	Reverse	CCRGAYTGRCCRAARACRAAGTTGTC	Woudenberg *et al.* (2009)
Calmodulin	*CaM*	CAL-228f	Forward	GAGTTCAAGGAGGCCTTCTCCC	Carbone & Kohn (1999)
		CAL-CL1^2^	Forward	GARTWCAAGGAGGCCTTCTC	O'Donnell *et al.* (2000b)
		CAL-CL2A^2^	Reverse	TTTTTGCATCATGAGTTGGAC	O'Donnell *et al.* (2000b)
		CAL-2Rd	Reverse	TGRTCNGCCTCDCGGATCATCTC	Quaedvlieg *et al.* (2011)
Internal transcribed spacer region of the nrDNA	ITS	ITS5	Forward	GGAAGTAAAAGTCGTAACAAGG	White *et al.* (1990)
		V9G^2^	Forward	TTACGTCCCTGCCCTTTGTA	de Hoog & van den Ende (1998)
		ITS4	Reverse	TCCTCCGCTTATTGATATGC	White *et al.* (1990)
RNA polymerase largest subunit	*rpb1*	Fa	Forward	CAYAARGARTCYATGATGGGWC	O'Donnell *et al.* (2010)
		F7	Forward	CRACACAGAAGAGTTTGAAGG	O'Donnell *et al.* (2010)
		F8^1^	Forward	TTCTTCCACGCCATGGCTGGTCG	O'Donnell *et al.* (2010)
		F6^1^	Forward	CTGCTGGTGGTATCATTCACG	O'Donnell *et al.* (2010)
		R8	Reverse	CAATGAGACCTTCTCGACCAGC	O'Donnell *et al.* (2010)
		R9	Reverse	TCARGCCCATGCGAGAGTTGTC	O'Donnell *et al.* (2010)
		G2R^1^	Reverse	GTCATYTGDGTDGCDGGYTCDCC	O'Donnell *et al.* (2010)
RNA polymerase second largest subunit	*rpb2*	RPB2-5f2	Forward	GGGGWGAYCAGAAGAAGGC	Reeb *et al.* (2004)
		fRPB2-7cf	Forward	ATGGGYAARCAAGCYATGGG	Liu *et al.* (1999)
		fRPB2-7cr	Reverse	CCCATRGCTTGYTTRCCCAT	Liu *et al.* (1999)
		RPB2-11ar	Reverse	GCRTGGATCTTRTCRTCSACC	Liu *et al.* (1999)
Translation elongation factor 1-alpha	*tef1*	EF-1	Forward	ATGGGTAAGGARGACAAGAC	O'Donnell *et al.* (1998b)
		EF-2	Reverse	GGARGTACCAGTSATCATG	O'Donnell *et al.* (1998b)

1 Used only for sequencing reactions.

2 Alternative primer, not used in this study.

Nuclear ribosomal DNA (nrDNA), including the internal transcribed spacer region cistron (ITS) and the 28S large subunit nrDNA (LSU), are nearly useless for species recognition in *Fusarium* and related genera. Nevertheless, given the ease of amplification and the extensive data available for comparison in public databases (Schoch *et al.* 2012), these markers are useful in the discrimination between the multiple species complexes of *Fusarium,* and for obtaining a confident genus-level identification for *Fusarium* and related genera, allowing further DNA markers to be incorporated in the analyses. The ITS region can still provide valuable information at species level for related genera containing species formerly included in *Fusarium* (*Bisifusarium*, *Cosmosporella*, *Fusicolla*, *Macroconia*, *Microcera*, and *Stylonectria*).

Many protein-coding genes have been explored for identification and taxonomic purposes in *Fusarium* and fusarioid fungi. The two main genes used for identification are *tef1* and *rpb2*. Both offer high discriminatory power and are well represented in public databases. Translation elongation factor 1-α is commonly the first-choice identification marker as it has very good resolution power for most species in all the genera treated here, while *rpb2* allows for enhanced discrimination between closely related species. For example, some species in the *Fusarium fujikuroi* species complex (FFSC) and in *Neocosmospora* that are not easily separated by using *tef1* alone (O'Donnell 2000, Nalim *et al.* 2011, Herron *et al.* 2015), can be resolved with *rpb2.* On the other hand, PCR amplification and sequencing success are often better for *tef1* than for *rpb2*. When used for phylogenetic analyses, sequence alignments of *rpb2* sequences are much more robust and less ambiguous than *tef1* data, given the former gene's advantageously low proportion of introns. An analogous situation has been shown in *Aspergillus* (Samson *et al.* 2014) and *Penicillium* (Visagie *et al.* 2014).

Additional genetic markers, often employed in association with the previously mentioned genes in multigene phylogenetic analyses include *acl1*, *tub2*, *CaM*, and *rpb1*. These markers have variable resolution or applicability depending on the genus or species complex. For example, use of *CaM* data may yield conflicting clade resolutions in the FFSC (O'Donnell 2000, Al-Hatmi *et al.* 2019), while paralogous or xenologous gene copies have been demonstrated for *tub2* in the *F. chlamydosporum* and *F. incarnatum-equiseti* species complexes (O'Donnell *et al.* 2009) as well as in *Neocosmospora* (O'Donnell 2000, O'Donnell *et al.* 2008a).

The most widely used algorithm for fungal identification by means of DNA markers is the Basic Local Alignment Search Tool (BLAST), available at the NCBI's GenBank website. This is a quick and useful method that can convey a great deal of information, but its results must be analysed with care given the presence of a high proportion of misidentified strains and low-quality sequences that must be filtered out (Vilgalys 2003, Nilsson *et al.* 2012). Sequences from type material are present in the GenBank nucleotide database for most fusarioid species known from culture, especially for *rpb2* and *tef1* barcodes, but the ex-type status of these sequences is not always explicitly mentioned. In many cases the names listed do not reflect the current taxonomy, even for sequences derived from ex-type cultures.

Some sequences used in past phylogenetic analyses of O'Donnell *et al.* (2020) and Geiser *et al.* (2021) appear to be linked to incorrect *Fusarium* names, likely due to errors in the database used. For this reason, we recommend the use of our curated database: Fusarioid-ID (https://www.fusarium.org). It can also be used for sequence similarity-based analysis of routine isolations and for identifications within several related genera.

## MALDI-TOF

A number of studies have thus far demonstrated the utility of mass spectrometry (MS) for species determination of subgroups of *Fusarium*, particularly members of the FFSC (Al-Hatmi *et al.* 2015, 2016, Wigmann *et al.* 2019). It is also useful for clinically relevant subgroups within several *Fusarium* species complexes (Marinach-Patrice *et al.* 2009, Triest *et al.* 2015, Sleiman *et al.* 2016, Paziani *et al.* 2020) and clinically relevant *Bisifusarium* (Triest *et al.* 2015, Paziani *et al.* 2020) and *Neocosmospora* species (Marinach-Patrice *et al.* 2009, Triest *et al.* 2015, Sleiman *et al.* 2016, Paziani *et al.* 2020). These techniques show highly accurate discriminative power, comparable to what has been shown with bacteria and yeasts. Only a limited number of taxa have thus far been evaluated, and a genus-wide evaluation of applicability of MALDI-TOF to *Fusarium* and related taxa is pending. The main limiting factor is, as usual, the current lack of representation of these taxa in commercial spectrum databases, a matter that can be resolved by constructing in-house, curated reference databases of spectra. Online availability and comparison of MS spectra of *Fusarium* has been proposed by Triest *et al.* (2015).

## Materials and methods

### Isolates and fungarium specimens

Fungal strains were obtained from the Westerdijk Fungal Biodiversity Institute (WI) collection (CBS), the Belgian Coordinated Collections of Microorganisms (IHEM), the International Mycological Institute (IMI), and the personal collection of Pedro W. Crous (CPC) housed at WI. For the list of names applied to the genus *Fusarium* and related fungarium specimens, the following fungaria were approached for holotype specimens: B, BM, BO, BP, BPI, BR, BRA, C, CBS, CO, DAOM, E, FH, H, HAL, IMI, K(M), L, LEP, M, MASS, MPA, NY, PC, PAD, PARMA, PAV, PH, PRM, ROVP, SIENA, STR, UPS, VPRI, W, and WIR.

### DNA amplification and phylogeny

Total genomic DNA was extracted from isolates grown for 7 d on PDA or MEA (recipes in Crous *et al.* 2019a; Table 1) incubated at 24 °C under a 12/12 h photoperiod using the Wizard® Genomic DNA purification Kit (Promega Corporation, Madison, WI, USA), following the manufacturer's instructions. Partial gene sequences were determined for eight DNA markers, *i.e*., *acl1*, *CaM*, ITS, LSU, *rpb1, rpb2*, *tef1*, and *tub2* using PCR protocols described elsewhere (O'Donnell *et al.* 1998b, 2007, 2010, Lombard *et al.* 2015). Primer pairs used for amplification and sequencing of the respective gene regions are summarised in Table 2. Consensus sequences for each marker were assembled in Geneious R11 (Kearse *et al.* 2012) or SeqMan Pro v. 15.3.0 (DNASTAR, Madison, WI, USA). All sequences generated in this study were deposited in GenBank (Table 3; also see Diagnostic DNA Barcodes in list of *Fusarium* names). The multiple sequence alignments and phylogenetic trees were deposited in TreeBASE (study ID 28093).

**Table 3:** Details of strains included in the phylogenetic analyses.

Species name	Strain^1^	Substrate	Country	GenBank accession number^2^							
				*acl1*	*CaM*	ITS	LSU	*rpb1*	*rpb2*	*tef1*	*tub2*
*Albonectria albosuccinea*	NRRL 20459	Unidentified tree	Venezuela	—	—	JAADYS010000048.1∗	JAADYS010000048.1∗	JX171471	JX171585	JAADYS010002360.1∗	—
*A. rigidiuscula*	CBS 133754	*Bauhinia longicupsis*	French Guiana	—	—	**MW827602**	**MW827641**	**MW834177**	**MW833995**	**MW834269**	—
*Atractium crassum*	CBS 180.31^T^ = NRRL 20894	Water tap	Germany	—	—	KM231790	MH866623	**MW834178**	HQ897722	KM231919	—
*At. stilbaster*	DAOM 215627	Cut stump	Canada	—	—	—	HQ843769	—	HQ897748	—	—
*Bisifusarium delphinoides*	CBS 110140 = FRC E-0073 = NRRL 36160	Human eye	USA	—	—	**MW827603**	**MW827642**	JX171535	HM347219	EU926302	—
*B. dimerum*	CBS 108944^ET^ = NRRL 36140	Human blood	Netherlands	—	—	JQ434586	JQ434514	—	HM347218	KR673912	—
*B. nectrioides*	CBS 176.31^T^ = NRRL 20689	Humus	Honduras	—	—	EU926245	EU926245	JX171477	JX171591	EU926312	—
*B. penzigii*	CBS 116508 = ATCC 15621 = NRRL 20711	Human eye	Sri Lanka	—	—	EU926256	EU926256	JX171482	HM347217	EU926323	—
*Corinectria fuckeliana*	CBS 239.29 = IMI 039700	*Picea sitchensis*	Scotland	—	—	**MW827604**	**MW827643**	**MW834179**	**MW833996**	DQ789728	—
*Co. tsugae*	CBS 788.69^T^	*Tsuga heterophylla*	Canada	—	—	KM231763	KM231763	—	KM231763	**MW834270**	—
*Cosmospora butyri*	CBS 301.38^T^ = MUCL 9950	Butter	Denmark	—	—	**MW827605**	**MW827644**	**MW834180**	HQ897729	—	—
*Cs. coccinea*	CBS 341.70	*Inonotus nodulosus* on *Fagus sylvatica*	Germany	—	—	MH859703	KM231692	**MW834181**	HQ897777	KM231947	—
*Cs. khandalensis*	CBS 356.65^IT^ = ATCC 16091 = IMI 112790 = MUCL 7974	*Bambusa* sp.	India	—	—	MH858608	NG_069711	—	**MW833997**	—	—
*Cs. lavitskiae*	CBS 530.68^T^ = ATCC 18666 = IMI 133984	Plant debris	Ukraine	—	—	KU563624	HQ231997	—	**MW833998**	**MW834271**	—
*Cs. viridescens*	CBS 102433	*Tilia* sp.	Czech Republic	—	—	KJ676148	KJ676185	**MW834182**	**MW833999**	KJ676343	—
*Cosmosporella cavisperma*	CBS 172.31^ET^ = NRRL 13996	*Pinus sylvestris*	Norway	—	—	**MW827606**	**MW827645**	JX171465	**MW834000**	—	—
*Cyanonectria buxi*	CBS 125551^ET^	Dead terminal branches connected with alive *Buxus sempervirens* var. *elegantissima*	Slovenia	—	—	NR_145049	MH875034	**MW834183**	**MW834001**	KM231939	—
*C. cyanostoma*	CBS 101734^ET^ = CBS 115512 = GJS 98-127	*Buxus sempervirens*	France	—	—	FJ474076	MH874353	**MW834184**	**MW834002**	HM626647	—
*Dialonectria episphaeria*	CBS 125494	Old ascomycete ascomata	Canada	—	—	MH863609	MH875085	**MW834185**	HQ897756	KM231953	—
*D. ullevolea*	CBS 125493	Ascomycete on *Fagus americana*	USA	—	—	KM231821	KM231696	—	HQ897782	KM231952	—
*Fusarium acutatum*	CBS 402.97^T^ = BBA 69580 = FRC O-1117 = NRRL 13309	Unknown	India	—	MW402459	—	—	MW402653	MW402768	MW402125	MW402323
*F. agapanthi*	NRRL 54463^T^	*Agapanthus* sp.	Australia	—	KU900611	—	—	KU900620	KU900625	KU900630	KU900635
*F. ananatum*	CBS 118516^T^ = CMW 18685 = MRC 8165	*Ananas comosus fruit*	South Africa	—	LT996175	—	—	LT996188	LT996137	LT996091	LT996112
*F. andiyazi*	CBS 119857^T^ = NRRL 31727	*Sorghum bicolor soil debris*	South Africa	—	LT996176	—	—	LT996189	LT996138	LT996092	LT996113
*F. anthophilum*	CBS 737.97 = DAOM 225119 = FRC M-1355 = IMI 375325 = NRRL 13602	*Hippeastrum* sp.	Germany	—	LT996177	—	—	LT996190	LT996139	LT996093	LT996114
*F. bactridioides*	NRRL 20476	*Cronartium conigenum*	USA	—	AF158343	—	—	Not public	Not public	AF160290	U34434
*F. begoniae*	CBS 403.97^T^ = BBA 67781 = DAOM 225116 = IMI 375315 = NRRL 25300	*Begonia elatior* hybrid	Germany	—	AF158346	—	—	LT996191	LT996140	AF160293	U61543
*F. beomiforme*	CBS 740.97 = BBA 65829 = DAOM 225123 = IMI 375328 = NRRL 25174	Soil	New Caledonia	—	—	U61674	U61648	JX171506	JX171619	PVQB02000800∗	—
*F. brevicatenulatum*	CBS 404.97^T^ = BBA 69197 = DAOM 225122 = IMI 375329 = NRRL 25446	*Striga asiatica*	Madagascar	—	**MW834108**	—	—	—	MN534295	MN533995	MN534063
*F. buharicum*	CBS 796.70 = ATCC 24135 = BBA 11122 = DSM 62165 = FRC R-4955 = IMI 141195 = NRRL 13371	*Hibiscus cannabinus*	Iran	—	—	U34581	U34552	JX171449	JX171563	—	—
*F. bulbicola*	CBS 220.76^T^ = BBA 12293 = BBA 63628 = DAOM 225114 = IMI 202877 = IMI 375322 = NRRL 13618	*Nerine bowdenii*	Germany	—	KF466327	—	—	KF466394	KF466404	KF466415	KF466437
*F. circinatum*	CBS 405.97^T^ = BBA 69720 = DAOM 225113 = IMI 375321 = MRC 7541 = NRRL 25331	*Pinus radiata*	USA	—	KM231393	—	—	JX171510	HM068354	KM231943	KM232080
*F. coicis*	NRRL 66233^T^ = RBG 5368	*Coix gasteenii*	Australia	—	LT996178	—	—	KP083269	KP083274	KP083251	LT996115
*F. compactum*	NRRL 13829	River sediments	Japan	—	—	—	—	JX171460	JX171574	—	—
*F. concentricum*	CBS 450.97^T^ = BBA 64354 = CBS 833.85 = DAOM 225146 = IMI 375352 = NRRL 25181	*Musa sapientum*	Costa Rica	—	AF158335	—	—	LT996192	JF741086	AF160282	U61548
*F. cugenangense*	CBS 130308 = NRRL 25387 = ATCC 26225	Human toe nail	New Zealand	—	—	**MW827607**	**MW827646**	JX171512	JX171625	MH485011	—
*F. curvatum*	CBS 744.97 = IMI 375335 = NRRL 22902	*Pseudotsuga menziesii*	USA	—	AF158365	—	—	LT996203	LT575065	AF160312	U34424
*F. denticulatum*	CBS 735.97 = NRRL 25302	*Ipomoea batatas*	USA	—	AF158322	—	—	LT996195	LT996143	AF160269	U61550
*F. dlaminii*	CBS 119860^T^ = BBA 69859 = FRC M-1637 = MRC 3032 = NRRL 13164	Soil debris in cornfield	South Africa	—	AF158330	—	—	KU171681	KU171701	AF160277	U34430
*F. echinatum*	CBS 146496 = CPC 30814	Unidentified tree	South Africa	—	**MW834109**	—	—	**MW834186**	**MW834003**	**MW834272**	**MW834300**
	CBS 146497^T^ = CPC 30815	Unidentified tree	South Africa	—	**MW834110**	—	—	**MW834187**	**MW834004**	**MW834273**	**MW834301**
*F. equiseti*	CBS 245.61 = NRRL 20697	*Beta vulgaris*	Chile	—	—	MH858038	MH869603	JX171481	JX171595	—	—
*F. flocciferum*	CBS 831.85 = BBA 64346 = NRRL 25473	*Triticum aestivum*	Germany	—	—	—	**MW827647**	JX171514	JX171627	—	—
*F. fracticaudum*	CBS 137234^PT^ = CMW 25237	*Pinus maximonoii*	Colombia	—	LT996179	—	—	LT996196	LT996144	KJ541059	KJ541051
*F fractiflexum*	NRRL 28852^T^	*Cymbidium* sp.	Japan	—	AF158341	—	—	Not public	LT575064	AF160288	AF160315
*F. fredkrugeri*	CBS 144209^T^ = CPC 33747	*Melhania acuminata* rhizosphere	South Africa	—	LT996181	—	—	LT996199	LT996147	LT996097	LT996117
*F. fujikuroi*	CBS 221.76^T^ = BBA 12428 = BBA 63630 = IHEM 3821 = IMI 196086 = IMI 202879 = NRRL 13620 = NRRL 13998 = NRRL 22174	*Oryza sativa*	Taiwan	—	—	**MW827608**	**MW827648**	**MW834188**	**MW834005**	AF160279	—
	NRRL 13566 = ATCC 38941 = DAOM 225143 = IMI 300793 = IMI 375349 = NRRL 5538 = NRRL A-26483	*Oryza sativa*	China	—	AF158332	—	—	JX171456	JX171570	AF160279	U34415
*F. globosum*	CBS 428.97^T^ = DAOM 214966 = FRC M-8014 = IMI 375330 = MRC 6647 = NRRL 26131 = PREM 51878	*Zea mays*	South Africa	—	KF466329	—	—	KF466396	KF466406	KF466417	KF466439
*F. graminearum*	CBS 123657 = NRRL 31084	*Zea mays*	USA	—	—	DQ459823	DQ459823	JX171531	JX171644	AY452957	—
*F. heterosporum*	CBS 720.79 = NRRL 20693	*Claviceps purpurea* on *Lolium perenne*	Netherlands	—	—	**MW827609**	**MW827649**	JX171480	JX171594	JAAGWP010000622.1∗	—
*F. inflexum*	CBS 716.74^T^ = ATCC 32213 = BBA 63203 = DAOM 225130 = DSM 63203 = IMI 375336 = NRRL 20433	*Vicia faba*	Germany	—	AF158366	—	—	JX171469	JX171583	AF008479	U34435
*F. konzum*	CBS 119849^T^ = MRC 8427	*Sorghastrum nuttans*	USA	—	LT996182	—	—	LT996200	LT996148	LT996098	LT996118
*F. lactis*	CBS 411.97^ET^ = BBA 68590 = DAOM 225145 = IMI 375351 = NRRL 25200	*Ficus carica*	USA	—	AF158325	—	—	LT996201	LT996149	AF160272	U61551
*F. lateritium*	NRRL 13622 = NRRL A-26433	*Ulmus* sp.	USA	—	—	—	—	JX171457	JX171571	—	—
*F. longipes*	NRRL 20723 = IMI 265540	Unknown	England	—	—	—	—	JX171483	JX171596	—	—
*F. mangiferae*	NRRL 25226 = BBA 69662 = DAOM 225155 = IMI 304063 = IMI 375361	*Mangifera indica*	Israel	—	AF158334	—	—	JX171509	HM068353	AF160281	U61561
*"F." melanochlorum*	CBS 202.65 = ATCC 16069 = BBA 9831 = DSM 62248 = NRRL 36353	*Fagus sylvatica*	Austria	—	—	MH858541	MH870179	JX171537	JX171649	—	—
*F. mexicanum*	NRRL 47473	*Mangifera indica*	Mexico	—	GU737389	—	—	LR792579	LR792615	GU737416	GU737308
*F. napiforme*	CBS 748.97^T^ = BBA 69861 = DAOM 225147 = FRC M-3563 = IMI 375353 = MRC 4144 = NRRL 13604	*Pennisetum typhoides*	Namibia	—	AF158319	—	—	HM347136	EF470117	AF160266	U34428
*F. nurragi*	CBS 392.96 = NRRL 36452	Soil	Australia	—	—	**MW827610**	**MW827650**	JX171538	JX171650	JAALXI010000436.1∗	—
*F. nygamai*	CBS 749.97^T^ = ATCC 58555 = BBA 69862 = DAOM 225148 = FRC M-1375 = IMI 375354 = NRRL 13448	*Sorghum bicolor*	Australia	—	AF158326	—	—	LT996202	EF470114	AF160273	U34426
*F. parvisorum*	CBS 137236^T^	*Pinus patula*	Colombia	—	LT996183	—	—	—	LT996150	KJ541060	KJ541055
*F. phyllophilum*	CBS 216.76^T^ = BBA 11730 = BBA 63625 = DAOM 225132 = IMI 202874 = IMI 375338 = NRRL 13617	*Dracaena deremensis*	Italy	—	KF466333	—	—	KF466399	KF466410	KF466421	KF466443
*F. poae*	NRRL 13714 = FRC T-503 = MRC 2181	Overwintered wheat	Canada	—	—	—	—	JX171458	JX171572	—	—
*F. prieskaense*	CPC 30825	*Aloidendron dichotomum*	South Africa	—	**MW834111**	—	—	**MW834189**	**MW834006**	**MW834274**	**MW834302**
	CBS 146498^T^ = CPC 30826	*Prunus spinosa*	South Africa	—	**MW834112**	—	—	**MW834190**	**MW834007**	**MW834275**	**MW834303**
	CBS 146499 = CPC 30827	*Prunus spinosa*	South Africa	—	**MW834113**	—	—	**MW834191**	**MW834008**	**MW834276**	**MW834304**
*F. phyllophilum*	CBS 217.76 = BBA 11341 = BBA 63624 = DAOM 225133 = IMI 202873 = IMI 375339 = NRRL 22944	*Cattleya* sp.	Germany	—	KF466333	U34558	U34529	JX171504	JX171617	AF160280	KF466443
*F. pseudocircinatum*	CBS 449.97^T^ = ATCC 24379 = BBA 69636 = CBS 126.73 = IMI 105384 = NRRL 22946	*Solanum* sp.	Ghana	—	AF158324	—	—	LT996204	LT996151	AF160271	U34427
*F. pseudograminearum*	CBS 109956^T^ = NRRL 28062	*Hordeum vulgare*	Australia	—	—	DQ459871	DQ459871	JX171524	JX171637	AF212468	—
*F. pseudonygamai*	CBS 417.97^T^ = BBA 69552 = FRC M-1166 = IMI 375342 = NRRL 13592	*Pennisetum typhoides*	Nigeria	—	AF158316	—	—	LT996205	LT996152	AF160263	U34421
*F. ramigenum*	CBS 418.98^T^ = BBA 68592 = DAOM 225137 = IMI 375343 = NRRL 25208	*Ficus carica*	USA	—	KF466335	—	—	KF466401	KF466412	KF466423	KF466445
*F. redolens*	CBS 743.97 = DAOM 225128 = IMI 375334 = NRRL 22901	*Pseudotsuga menziesii*	Canada	—	—	U34565	U34536	JX171503	JX171616	MT409452	—
*F. sacchari*	CBS 223.76^ET^ = BBA 63340 = DAOM 225138 = IMI 202881 = NRRL 13999	*Saccharum officinarum*	India	—	AF158331	—	—	JX171466	JX171580	AF160278	U34414
*F. sambucinum*	CBS 146.95 = BBA 64226 = NRRL 22187 = NRRL 20727	*Solanum tuberosum*	England	—	—	—	—	JX171493	JX171606	**MW834277**	—
*F. sarcochroum*	CBS 745.79 = BBA 63714 = NRRL 20472	*Viscum album*	Switzerland	—	—	**MW827611**	**MW827651**	JX171472	JX171586	**MW834278**	—
*F. scirpi*	NRRL 13402	Soil	Australia	—	—	GQ505681	GQ505681	JX171452	JX171566	GQ505592	—
*F. sororula*	CBS 137242^T^ = CMW 40578	*Pinus patula*	Colombia	—	LT996184	—	—	LT996206	LT996153	KJ541067	KJ541057
*Fusarium sp.*	CBS 102163 = GJS 84-426	Bamboo	Venezuela	—	—	KM231812	KM231681	**MW834193**	**MW834009**	KM231940	—
*F. sterilihyposum*	NRRL 25623	Mango	South Africa	—	AF158353	—	—	MW402713	MN193897	AF160300	AF160316
*F. stilboides*	NRRL 20429 = ATCC 15662	*Coffea* sp.	Nyasaland	—	—	—	—	JX171468	JX171582	—	—
*F. subglutinans*	CBS 747.97^ET^ = BBA 62451 = DAOM 225141 = FRC M-36 = MRC 8554 = NRRL 22016 = NRRL 22114	*Zea mays*	USA	—	AF158342	—	—	JX171486	JX171599	AF160289	U34417
*F. sublunatum*	CBS 189.34^T^ = BBA 62431 = DSM 62431 = NRRL 20840 = NRRL 13384	Soil	Costa Rica	—	—	HQ897830	KM231680	JX171451	JX171565	—	—
*F. succisae*	CBS 219.76^ET^ = BBA 12287 = BBA 63627 = DAOM 225142 = IMI 202876 = IMI 375347 = NRRL 13613	*Succisa pratensis*	Germany	—	AF158344	—	—	LT996207	LT996154	AF160291	U34419
*F. sudanense*	CBS 454.97^T^ = BBA 65862 = NRRL 25451 = NRRL 26793	*Striga hermonthica*	Sudan	—	LT996185	—	—	LT996208	LT996155	KU711697	KU603909
*F. temperatum*	NRRL 25622 = NRRL 26616	*Zea mays*	South Africa	—	AF158354	—	—	Not public	Not public	AF160301	AF160317
*F. terricola*	CBS 483.94^T^ = FRC M-1650	Soil	Australia	—	KU603951	—	—	LT996209	LT996156	KU711698	KU603908
*F. thapsinum*	CBS 733.97 = DAOM 225109 = IMI 375317 = MRC 6002 = NRRL 22045	*Sorghum bicolor*	South Africa	—	LT996186	—	—	JX171487	JX171600	AF160270	U34418
*F. tjaetaba*	CBS 144400^T^ = NRRL 66243 = RBG 5361	*Sorghum interjectum*	Australia	—	LT996187	—	—	**MW834192**	KP083275	KP083263	GU737296
*F. torreyae*	CBS 133858^T^ = NRRL 54151	*Torreya* sp.	USA	—	—	HM068344	**MW827652**	JX171548	JX171660	HM068337	—
*F. tricinctum*	CBS 393.93^ET^ = BBA 64485 = NRRL 25481	Winter wheat culm base	Germany	—	—	HM068317	HM068317	JX171516	JX171629	AB674263	—
*F. tupiense*	NRRL 53984	*Mangifera indica*	Brazil	—	GU737377	—	—	LR792583	LR792619	GU737404	GU737296
*F. udum*	CBS 178.32 = BBA 1813 = DAOM 225111 = IMI 375319 = NRRL 22949	*Lactarius pubescens*	Germany	—	AF158328	—	—	LT996220	LT996172	AF160275	U34433
*F. venenatum*	NRRL 22196 = BBA 65031	*Zea mays*	Germany	—	—	—	—	JX171494	JX171607	—	—
*F. verticillioides*	CBS 734.97 = BBA 62264 = IMI 375318 = NRRL 22172	*Zea mays*	Germany	—	AF158315	—	—	LT996221	EF470122	AF160262	U34413
*F. xylarioides*	CBS 258.52^ET^ = NRRL 25486	*Coffea* sp.	Ivory Coast	—	—	—	—	JX171517	HM068355	AY707136	AY707118
*Fusicolla acetilerea*	BBA 63789^T^ = IMI 181488 = NRRL 20827	Polluted soil	Japan	—	—	HQ897790	U88108	—	HQ897701	—	—
	BBA 63789 = IMI 181488 = NRRL 20827	Polluted soil	Japan	HQ897839	—	HQ897790	U88108	—	HQ897701	—	—
*Fu. aquaeductuum*	CBS 734.79 = BBA 63669 = NRRL 20686	Drinking water	Germany	—	—	**MW827612**	**MW827653**	JX171476	HQ897742	**MW847905**	—
	CBS 268.53	Rubber tubing	Netherlands	—	—	MH857190	MH868728	—	—	—	—
	CBS 837.85^ET^ =BBA 64559 = NRRL 20865 = NRRL 37595	Plug in water tap	Germany	—	—	KM231823	KM231699	—	—	—	KM232094
*Fu. betae*	BBA 64317^ET^	*Triticum aestivum*	Germany	HQ897917	—	MH855265	MH866717	—	HQ897781	—	—
*Fu. bharatavarshae*	NFCCI 4423^T^	*Avicennia marina*	India	—	—	MK152510	MK152511	—	MK157022	—	MK376462
*Fu. cassiae-fistulae*	MFLUCC 19-0318^T^	*Cassia fistula*	Thailand	—	—	MT215497	MT215549	—	—	—	—
*Fu. epistroma*	BBA 62201^ET^ = ATCC 24369 = IMI 85601 = NRRL 20439 = NRRL 20461	*Diatrypella* sp., on *Betula* sp.	England	HQ897901	—	—	AF228352	—	HQ897765	—	—
*Fu. gigantispora*	HKAS 101990	*Bruguiera* sp.	Thailand	—	—	MN047106	MN017870	—	—	—	—
	MFLU 161206^T^	*Avicennia marina*	Thailand	—	—	MN047105	MN017876	—	—	—	—
*Fu. matuoi*	CBS 581.78 = ATCC 18694 = MAFF 238445 = NRRL 20427	*Albizzia julibrissin*	Japan	HQ897858	—	KM231822	KM231698	**MW834194**	HQ897720	KM231954	KM232093
*Fu. melogrammae*	CBS 141092^T^	*Melogramma campylosporum* on *Carpinus* sp.	England	—	—	KX897140	KY092489	—	HQ897720	—	**MW834305**
*Fu. meniscoidea*	CBS 110189 = FRC E-0086	Soil	Australia	**MW834043**	—	**MW827613**	**MW827654**	—	**MW834010**	**MW834279**	**MW834306**
*Fu. merismoides*	CBS 186.34 = BBA 1867a = NRRL 20895	*Acer* sp.	Germany	—	—	MH855482	MH866963	—	—	—	—
*Fu. ossicola*	CBS 140161^T^	Bone of wild boar	Belgium	—	—	MF628022	MF628021	—	**MW834011**	**MW834280**	**MW834307**
*Fu. quarantenae*	URM 8367^T^ = CBS 141541	*Melocactus zehntneri*	Brazil	—	—	**MW553789**	**MW553788**	—	**MW556626**	**MW556625**	**MW556624**
*Fu. septimanifiniscientiae*	CBS 144935^T^	Soil	Netherlands	—	—	MK069422	MK069418	—	—	MK077808	MK069408
*Fu. siamensis*	MFLUCC 17-2577^T^	*Cassia fistula*	Thailand	—	—	MT215498	MT215550	—	—	—	—
*Fu. sporellula*	CBS 110191 = FRC E-0139	Soil	South Africa	**MW834044**	—	**MW827614**	**MW827655**	—	**MW834012**	**MW834281**	**MW834308**
*Fu. violacea*	CBS 634.76^T^ = BBA 62461 = NRRL 20896	*Quadraspidiotus perniciosus*	Iran	—	—	KM231824	U88112	**MW834195**	HQ897696	KM231956	KM232095
*Geejayessia atrofusca*	CBS 125482 = DAOM 238117	*Staphylea trifolia*	Canada	—	—	MH863592	MH875066	**MW834196**	HQ897775	**MW834282**	—
	NRRL 22316	*Staphylea trifolia*	USA	—	—	AF178423	AF178392	JX171496	EU329502	AF178361	—
*G. celtidicola*	CBS 125502^T^	*Celtis occidentalis*	Canada	HM626625	—	HM626657	HM626669	**MW834197**	**MW834013**	HM626638	KM232074
*G. cicatricum*	CBS 125550	Dead twig connected with alive *Buxus sempervirens* var. *elegantissima*	Slovenia	—	—	HM626654	HM626666	**MW834198**	HQ897697	HM626642	—
	CBS 125552	Dead twig	Slovenia	HQ728171	—	HQ728145	MH875038	—	HQ728153	HM626644	—
*Ilyonectria capensis*	CBS 132815^T^	*Protea* sp.	South Africa	—	—	NR_152887	NG_070049	**MW834199**	**MW834014**	JX231119	—
*I. destructans*	CBS 264.65	*Cyclamen persicum*	Sweden	—	—	MH858563	KM515927	—	**MW834015**	JF735695	—
*Luteonectria albida*	CBS 102683 = GJS 99-73 = GJS 8522A	Tree bark	Costa Rica	—	—	**MW827615**	MH874402	**MW834200**	**MW834016**	**MW834283**	—
	NRRL 22152^T^ = NRRL 13950	Woody stem bark	Jamaica	—	—	JABFEP010000142.1∗	JABFEP010000142.1∗	JX171492	JX171605	JABFEP010002685.1∗	—
*L. nematophila*	NRRL 54600	Unknown	Germany	—	—	JABFFA010000104.1∗	JABFFA010000104.1∗	JX171552	JX171664	JABFFA010003988.1∗	—
*Macroconia bulbipes*	CBS 146678 = CPC 37137	*Erica* sp. associated with *Dimerosporiopsis engleriana*	South Africa	**MW834045**	**MW834114**	**MW827616**	**MW827656**	**MW834201**	**MW834017**	—	**MW834309**
	CBS 146679^T^ = CPC 37138	*Erica* sp. associated with *Dimerosporiopsis engleriana*	South Africa	**MW834046**	**MW834115**	**MW827617**	**MW827657**	**MW834202**	**MW834018**	—	**MW834310**
*Ma. cupularis*	HMAS 173240^T^	*Stylodothis* sp. on unidentified tree	China	—	—	EF121864	EF121870	—	—	—	—
*Ma. gigas*	HMAS 173239^T^	Rotten stem of bamboo associated with other fungi	China	—	—	EF121853	EF121869	—	—	—	—
*Ma. leptosphaeriae*	CBS 100001	*Leptosphaeria* on dead stem of *Urtica dioica*	Netherlands	HQ897891	**MW834116**	HQ897810	HQ897755	**MW834203**	HQ728164	KM231959	KM232097
*Ma. papillionacearum*	CBS 125495	*Ascomycete* on *Fabaceae*	USA	HQ897912	**MW834117**	HQ897826	MH875086	**MW834204**	HQ897776	—	KM232096
*Ma. phlogioides*	CBS 125496	*Quercus* sp., branch in stream	USA	HQ897868	**MW834118**	**MW827618**	**MW827658**	**MW834205**	HQ897732	**MW834284**	**MW834311**
	CBS 146500 = CPC 35388	*Encephalartos* sp. leaf	South Africa	**MW834047**	**MW834119**	**MW827619**	**MW827659**	**MW834206**	**MW834019**	—	**MW834312**
	CBS 146501^T^ = CPC 35389	*Encephalartos* sp. leaf	South Africa	**MW834048**	**MW834120**	**MW827620**	**MW827660**	**MW834207**	**MW834020**	—	**MW834313**
*Ma. sphaeriae*	CBS 717.74	*Pyrenomycete* on *Coronilla emerus*	France	**MW834049**	**MW834121**	**MW827621**	**MW827661**	—	KM232390	—	KM232099
	CBS 112770	*Cucurbitaria laburni* on *Laburnum anagyroides*	Austria	KM231061	KM231413	**MW827622**	**MW827662**	**MW834208**	**MW834021**	—	KM232098
*Mariannaea elegans*	DAOM 226709	*Betula* sp.	Canada	—	—	—	HQ843768	—	HQ897747	—	—
*M. samuelsii*	CBS 125515^T^ = DAOM 235814	Soil	Guatemala	—	—	NR_137767	NG_060269	—	HQ897752	—	—
*Microcera coccophila*	CBS 310.34 = NRRL 13962	Scale insect	Italy	—	—	MH855540	KM231703	JX171462	JX171576	—	—
*Mi. diploa*	CBS 735.79 = BBA 61173 = NRRL 36545	*Quadraspidiotus perniciosus*	Iran	—	—	**MW827623**	**MW827663**	JX171463	JX171577	—	—
*Mi. larvarum*	CBS 738.79 = BBA 62239 = DSM 62239 = MUCL 19033 = NRRL 20473	*Quadraspidiotus perniciosus*	Iran	—	—	KM231825	KM231701	JX171473	JX171587	KM231957	—
*Mi. rubra*	CBS 638.76^IT^ = BBA 62460 = NRRL 20475; NRRL 22111; NRRL 22170	*Quadraspidiotus perniciosus* on *Prunus domestica*	Iran	HQ897903	KM231409	MH861019	MH872790	—	HQ897767	—	**MW834314**
*Microcera sp.*	NRRL 26790	*Parmelia rudecta*	USA	—	—	—	—	JX171523	JX171636	—	—
*Nectria cinnabarina*	CBS 125165^ET^	*Aesculus* sp.	France	KM231074	—	HM484548	HM484562	—	KM232402	HM484527	—
*"Nt." flavoviridis*	CBS 124353 = BBA 65542 = NRRL 22093	Decorticated wood	USA	—	—	HQ897791	**MW827664**	**MW834209**	HQ897702	—	—
*Neocosmospora acutispora*	CBS 145461^T^ = NRRL 22574 = BBA 62213	*Coffea arabica*	Guatemala	**MW834050**	**MW834122**	LR583700	LR583908	**MW834210**	LR583814	LR583593	—
*N. addoensis*	CBS 146509 = CPC 37127	*Citrus sinensis*	South Africa	MW218004	MW218051	MW173041	MW173032	MW218097	MW446574	MW248740	—
	CBS 146510^T^ = CPC 37128	*Citrus sinensis*	South Africa	MW218005	MW218052	MW173042	MW173033	MW218098	MW446575	MW248741	—
*N. ambrosia*	CBS 571.94^ET^ = NRRL 22346 = BBA 65390 = MAFF 246287	*Euwallacea fornicatus*	India	—	—	EU329669	EU329669	**MW834211**	EU329503	FJ240350	—
	NRRL 20438 = IMI 296597	*Xyleborus fornicatus*	India	—	—	AF178397	AF178366	JX171470	JX171584	NIZV01000014.1∗	—
*N. ampla*	CBS 202.32^T^ = BBA 4170	*Coffea* sp.	German East Africa	**MW834051**	**MW834123**	LR583701	LR583909	**MW834212**	LR583815	LR583594	—
*N. bataticola*	CBS 144397 = NRRL 22400 = BBA 64683	*Ipomoea batatas*	USA	MW218006	MW218053	AF178407	AF178376	MW218099	EU329509	AF178343	—
	CBS 144398^T^ = NRRL 22402 = BBA 64954 = FRC S-0567	*Ipomoea batatas*	USA	MW218007	MW218054	AF178408	AF178377	MW218100	FJ240381	AF178344	—
*N. borneensis*	CBS 145462^ET^ = NRRL 22579 = BBA 65095 = GJS 85-197	Bark or recently dead tree	Indonesia	**MW834052**	**MW834124**	AF178415	AF178384	**MW834213**	EU329515	AF178352	—
*N. bostrycoides*	CBS 144.25^NT^	Soil	Honduras	MW218008	MW218055	LR583704	LR583912	MW218101	LR583818	LR583597	—
	CBS 392.66 = NRRL 25325 = BBA 69595	*Bertholletia excelsa*	Unknown	MW218009	MW218056	LR583705	LR583913	MW218102	LR583819	LR583598	—
*N. brevicona*	CBS 204.31^ET^ = NRRL 22659 = BBA 2123	*Gladiolus* sp.	Indonesia	MW218010	MW218057	LR583707	LR583915	MW218103	LR583821	LR583600	—
*N. brevis*	CBS 130326 = NRRL 28009 = CDC B-5543	Human eye	USA	**MW834053**	**MW834125**	DQ094351	DQ236393	**MW834214**	EF470136	DQ246869	—
*N. catenata*	CBS 143228 = NRRL 54992 = UTHSC 09-1008	*Stegostoma fasciatum*	USA	MW218011	MW218058	KC808255	KC808255	MW218104	KC808354	KC808213	—
	CBS 143229^T^ = NRRL 54993 = UTHSC 09-1009	*Stegostoma fasciatum*	USA	MW218012	MW218059	KC808256	KC808256	MW218105	KC808355	KC808214	—
*N. citricola*	CBS 146512 = CPC 37130	*Citrus sinensis*	South Africa	MW218014	MW218061	MW173047	MW173035	MW218107	MW446580	MW248746	—
	CBS 146513^T^ = CPC 37131	*Citrus sinensis*	South Africa	MW218015	MW218062	MW173048	MW173036	MW218108	MW446581	MW248747	—
*N. crassa*	CBS 144386^T^ = MUCL 11420	Unknown	France	MW218016	MW218063	LR583709	LR583917	MW218109	LR583823	LR583604	—
*N. cryptoseptata*	CBS 145463^T^ = NRRL 22412 = BBA 65024	Bark	French Guiana	**MW834054**	**MW834126**	AF178414	AF178383	**MW834215**	EU329510	AF178351	—
*N. cucurbitae*	CBS 410.62 = NRRL 22658 = CECT 2864	*Cucurbita viciifolia*	Netherlands	**MW834055**	**MW834127**	LR583710	LR583918	**MW834216**	LR583824	DQ247640	—
	CBS 616.66^T^ = NRRL 22399 = BBA 64411	*Cucurbita viciifolia*	Netherlands	**MW834056**	**MW834128**	LR583711	LR583919	**MW834217**	LR583825	DQ247592	—
*N. cyanescens*	CBS 518.82^T^	Human foot	Netherlands	MW218017	MW218064	AB190389	LR583920	MW218110	LR583826	LR583605	—
	CBS 637.82	Human foot	Netherlands	MW218018	MW218065	LR583712	LR583921	MW218111	LR583827	LR583606	—
*N. diminuta*	CBS 144390^T^ = MUCL 18798	*Coelocaryon preusii*	Unknown	**MW834057**	**MW834129**	LR583713	LR583922	**MW834218**	LR583828	LR583607	—
*N. elegans*	CBS 144395 = NRRL 22163 = MAFF 238540 = ATCC 18690	*Xanthoxylum piperitum*	Japan	MW218019	MW218066	AF178394	AF178363	MW218112	EU329496	AF178328	—
	CBS 144396^ET^ = NRRL 22277 = MAFF 238541 = ATCC 42366	*Xanthoxylum piperitum*	Japan	MW218020	MW218067	AF178401	AF178370	MW218113	FJ240380	AF178336	—
*N. epipeda*	CBS 146523^T^ = CPC 38310	*Bouvardia* sp. imported from Uganda	Netherlands	**MW834058**	**MW834130**	**MW827624**	**MW827665**	**MW834219**	**MW834022**	**MW834285**	—
	CBS 146524 = CPC 38311	*Bouvardia* sp. imported from Uganda	Netherlands	**MW834059**	**MW834131**	**MW827625**	**MW827666**	**MW834220**	**MW834023**	**MW834286**	—
*N. euwallaceae*	CBS 135854^T^ = NRRL 54722	*Euwallacea* sp.	Israel	—	—	JQ038014	JQ038014	JQ038021	JQ038028	JQ038007	—
*N. falciformis*	CBS 475.67^T^ = IMI 268681	Human mycetoma	Puerto Rico	MW218021	MW218068	MG189935	MG189915	MW218114	LT960558	LT906669	—
	CBS 121450	Declined grape vine	Syria	MW218022	MW218069	JX435211	JX435211	MW218115	JX435261	JX435161	—
	NRRL 43529 = CDC 2006743575	Human cornea	USA	—	—	EF453117	EF453117	JX171541	JX171653	EF452965	—
*N. ferruginea*	CBS 109028^T^ = NRRL 32437	Human subcutaneous nodule	Switzerland	**MW834060**	**MW834132**	DQ094446	DQ236488	**MW834221**	EU329581	DQ246979	—
	CPC 28194	*Citrus sinensis*	Italy	**MW834061**	**MW834133**	LT746276	LT746276	**MW834222**	LT746341	LR583602	—
*N. floridana*	NRRL 62628^T^ = MAFF 246849	*Euwallacea interjectus*	USA	—	—	KC691563	KC691563	KC691593	KC691624, KC691653	KC691535	—
*N. gamsii*	CBS 143207^T^ = NRRL 32323 = UTHSC 99-205	Human bronchoalveolar lavage fluid	USA	**MW834062**	**MW834134**	DQ094420	DQ236462	**MW834223**	EU329622	DQ247103	—
	CBS 143211 = NRRL 32794 = FRC S-1152	Humidifier coolant	USA	**MW834063**	**MW834135**	DQ094563	DQ236605	**MW834224**	EU329576	DQ246951	—
*N. gamtoosensis*	CBS 146502^T^ = VG16 = CPC 37120	*Citrus sinensis*	South Africa	MW218023	MW218070	MW173063	MW173038	MW218116	MW446611	MW248762	—
*N. haematococca*	CBS 119600^ET^ = FRC S-1832	Dying tree	Sri Lanka	**MW834064**	**MW834136**	KM231797	KM231664	—	LT960561	DQ247510	—
*N. hypothenemi*	CBS 145464^T^ = NRRL 52782 = ARSEF 5878	*Hypothenemus hampei*	Benin	MW218024	—	LR583715	LR583923	MW218117	JF741176	JF740850	—
	CBS 145466 = NRRL 52783 = ARSEF 5879	*Hypothenemus hampei*	Uganda	MW218025	MW218071	**MW827626**	**MW827667**	MW218118	**MW834024**	**MW834287**	—
*N. illudens*	CBS 147303 = NRRL 22090 = BBA 67606 = GJS 82-98	*Beilschmiedia tawa*	New Zealand	**MW834065**	**MW834137**	AF178393	AF178362	JX171488	JX171601	AF178326	—
*N. ipomoeae*	CBS 353.87 = NRRL 22657	*Gerbera* sp.	Netherlands	MW218026	MW218072	LR583717	LR583925	MW218119	LR583831	DQ247639	—
	CBS 833.97	*Rosa* sp.	Netherlands	MW218027	MW218073	LR583719	LR583927	MW218120	LR583833	LR583611	—
*N. keleraja*	CBS 125720^PT^ = FRC S-1837 = GJS 02-114	Branch of unidentified tree	Sri Lanka	**MW834066**	**MW834138**	LR583720	LR583928	**MW834225**	LR583834	LR583612	—
	CBS 125722^PT^ = FRC S-1836 = GJS 02-114	Branch of unidentified tree	Sri Lanka	**MW834067**	**MW834139**	JF433039	JF433039	**MW834226**	LR583835	DQ247515	—
*N. keratoplastica*	CBS 490.63^T^	Human	Japan	MW218028	MW218074	LR583721	LR583929	MW218121	LT960562	LT906670	—
	CBS 144389 = MUCL 18301	Greenhouse humic soil	Belgium	MW218029	MW218075	LR583722	LR583930	MW218122	LR583836	LR583613	—
*N. kuroshio*	CBS 142642^T^	*Euwallacea* sp.	USA	**MW834068**	**MW834140**	LR583723	LR583931	**MW834227**	LR583837	KX262216	—
*N. kurunegalensis*	CBS 119599^T^ = GJS 02-94	Recently cut tree	Sri Lanka	**MW834069**	**MW834141**	JF433036	JF433036	**MW834228**	LR583838	DQ247511	—
*N. lerouxii*	CBS 146514^T^ = CPC 37132	*Citrus sinensis*	South Africa	MW218030	MW218076	MW173069	MW173039	MW218123	MW446617	MW248768	—
*N. lichenicola*	CBS 509.63 = MUCL 8050 = IMUR 410	Air	Brazil	**MW834070**	**MW834142**	LR583728	LR583936	**MW834229**	LR583843	LR583618	—
	CBS 623.92^ET^	Human	Germany	**MW834071**	**MW834143**	LR583730	LR583938	—	LR583845	LR583620	—
*N. liriodendri*	CBS 117481^T^ = NRRL 22389 = BBA 67587 = GJS 91-148	*Liriodendron tulipifera*	USA	MW218031	MW218077	AF178404	AF178373	MW218124	EU329506	AF178340	—
*N. longissima*	CBS 126407^T^ = GJS 85-72	Tree bark	New Zealand	**MW834072**	**MW834144**	LR583731	LR583939	**MW834230**	LR583846	LR583621	—
*N. macrospora*	CBS 142424^T^ = CPC 28191	*Citrus sinensis*	Italy	MW218032	MW218078	LT746266	LT746281	MW218125	LT746331	LT746218	—
	CPC 28193	*Citrus sinensis*	Italy	MW218033	MW218079	LT746268	LT746283	MW218126	LT746333	LT746220	—
*N. mahasenii*	CBS 119594^T^	Dead branch on live tree	Sri Lanka	**MW834073**	**MW834145**	JF433045	JF433045	**MW834231**	LT960563	DQ247513	—
*N. martii*	CBS 115659^ET^ = FRC S-0679 = MRC 2198	*Solanum tuberosum*	Germany	**MW834074**	**MW834146**	JX435206	JX435206	**MW834232**	JX435256	JX435156	—
*N. merkxiana*	CBS 146525^T^	*Chrysanthemum* sp. imported from Uganda	Netherlands	**MW834075**	**MW834147**	**MW827627**	**MW827668**	**MW834233**	**MW834025**	**MW834288**	—
	CBS 146526	*Chrysanthemum* sp. imported from Uganda	Netherlands	**MW834076**	**MW834148**	**MW827628**	**MW827669**	**MW834234**	**MW834026**	**MW834289**	—
*N. metavorans*	CBS 135789^T^	Human pleural effusion	Greece	MW218034	MW218080	LR583738	LR583946	MW218127	LR583849	LR583627	—
	CBS 143219 = NRRL 46708 = FMR 8634	Human foot	Spain	MW218035	MW218081	LR583744	LR583948	MW218128	LR583851	LR583629	—
*N. mori*	CBS 145467^T^ = NRRL 22230 = MAFF 238539	*Morus alba*	Japan	**MW834077**	**MW834149**	DQ094305	DQ236347	**MW834235**	EU329499	AF178358	—
	CBS 145468 = NRRL 22157 = MAFF 238538	*Morus alba*	Japan	**MW834078**	**MW834150**	DQ094306	DQ236348	**MW834236**	EU329493	AF178359	—
*N. neerlandica*	CBS 232.34^T^	*Pisum sativum*	Netherlands	**MW834079**	**MW834151**	**MW827629**	**MW827670**	**MW834237**	**MW847903**	**MW847906**	—
*N. nelsonii*	CBS 309.75^T^	*Pisum sativum*	Unknown	**MW834080**	**MW834152**	**MW827630**	**MW827671**	**MW834238**	**MW847904**	**MW847907**	—
*N. nirenbergiana*	CBS 145469^T^ = NRRL 22387 = BBA 65023 = GJS 87-127	Bark	French Guiana	**MW834081**	**MW834153**	AF178403	AF178372	—	EU329505	AF178339	—
*N. noneumartii*	CBS 115658^T^ = FRC S-0661	*Solanum tuberosum*	Israel	MW218036	MW218082	LR583745	LR583949	MW218129	MW446618	LR583630	—
*N. obliquiseptata*	NRRL 62611 = MAFF 246845	*Euwallacea* sp.	Australia	—	—	KC691576	KC691576	KC691606	KC691637, KC691666	KC691548	—
*N. oblonga*	CBS 130325^T^ = NRRL 28008 = CDC B-4701	Human eye	USA	**MW834082**	**MW834154**	LR583746	LR583950	**MW834239**	LR583853	LR583631	—
*N. oligoseptata*	CBS 143241^T^ = NRRL 62579 = FRC S-2581 = MAFF 246283	*Euwallacea validus*	USA	**MW834083**	**MW834155**	KC691566	KC691566	KC691596	LR583854	KC691538	—
*N. paraeumartii*	CBS 487.76^T^ = NRRL 13997 = BBA 62215	*Solanum tuberosum*	Argentina	**MW834084**	**MW834156**	LR583747	LR583951	**MW834240**	LR583855	DQ247549	—
*N. parceramosa*	CBS 115695^T^	Soil	South Africa	MW218037	MW218083	JX435199	JX435199	—	JX435249	JX435149	—
*N. perseae*	CBS 144142^T^ = CPC 26829	*Persea americana*	Italy	MW218038	MW218084	LT991940	LT991947	MW218130	LT991909	LT991902	—
*N. petroliphila*	CBS 203.32 = NRRL 13952	*Pelargonium* sp.	South Africa	MW218039	MW218085	DQ094320	DQ236362	MW218131	LR583857	DQ246835	—
	CBS 224.34 = NRRL 28579	Human toenail	Cuba	MW218040	MW218086	DQ094383	DQ236425	MW218132	LR583858	DQ246910	—
*N. phaseoli*	CBS 265.50	*Phaseolus* sp.	USA	**MW834085**	**MW834157**	LR583750	LR583954	—	KJ511278	FJ919464	—
	NRRL 22276 = ATCC 38466	*Phaseolus vulgaris*	USA	—	—	EU329668	EU329668	JX171495	JX171608	AY220186	—
*N. piperis*	CBS 145470^T^ = NRRL 22570 = GJS 89-14 = CML 1888	*Piper nigrum*	Brazil	**MW834086**	**MW834158**	AF178422	AF178391	**MW834241**	EU329513	AF178360	—
*N. pisi*	CBS 123669^ET^ = NRRL 45880 = ATCC MYA-4622	Progeny of parentals from *Pisum sativum* and soil	USA	**MW834087**	**MW834159**	LR583753	LR583957	**MW834242**	LR583862	LR583636	—
	CBS 142372	*Trifolium subterraneum*	Germany	**MW834088**	**MW834160**	LR583755	LR583959	**MW834243**	LR583864	KY556454	—
*N. plagianthi*	NRRL 22632 = GJS 83-146	*Hoheria glabrata*	New Zealand	—	—	AF178417	AF178386	JX171501	JX171614	AF178354	—
*N. protoensiformis*	CBS 145471^T^ = NRRL 22178 = GJS 90-168	Dicot tree	Venezuela	**MW834089**	**MW834161**	AF178399	AF178368	**MW834244**	EU329498	AF178334	—
*N. pseudensiformis*	CBS 130.78 = NRRL 22575 = NRRL 22653	*Cocos nucifera*	Indonesia	**MW834090**	**MW834162**	LR583759	LR583963	**MW834245**	LR583868	DQ247635	—
*N. pseudopisi*	CBS 266.50	*Pisum sativum*	Unknown	**MW834091**	**MW834163**	**MW827631**	**MW827672**	**MW834246**	**MW834027**	**MW834290**	—
*N. pseudoradicicola*	CBS 145472^T^ = NRRL 25137 = ARSEF 2313	Diseased cocoa pods	Papua New Guinea	MW218041	MW218087	JF740899	JF740899	MW218133	JF741084	JF740757	—
*N. quercicola*	CBS 141.90^T^ = NRRL 22652	*Quercus cerris*	Italy	**MW834092**	**MW834164**	LR583760	LR583964	**MW834247**	LR583869	DQ247634	—
*N. rectiphora*	CBS 125726 = FRC S-1842	Dead tree	Sri Lanka	**MW834093**	**MW834165**	JF433043	JF433043	**MW834248**	**MW834028**	JF433026	—
	CBS 125727^T^ = GJS 02-89 = FRC S-1831	Dead tree	Sri Lanka	**MW834094**	**MW834166**	JF433034	JF433034	**MW834249**	LR583871	DQ247509	—
*N. regularis*	CBS 190.35	*Phaseolus* sp.	USA	**MW834095**	**MW834167**	LR583762	LR583966	**MW834250**	LR583872	LR583642	—
	CBS 230.34^T^	*Pisum sativum*	Netherlands	**MW834096**	**MW834168**	LR583763	LR583967	—	**MW834029**	LR583643	—
*N. rekana*	CMW 52862^T^	*Euwallacea perbrevis*	Indonesia	—	—	MN249094	—	—	MN249137, MN249108	MN249151	—
*N. robusta*	CBS 145473^T^ = NRRL 22395 = BBA 65682	Bark	Venezuela	—	**MW834169**	AF178405	LR583968	**MW834251**	EU329507	AF178341	—
*N. samuelsii*	CBS 114067^T^ = GJS 89-70	Bark	Guyana	**MW834097**	**MW834170**	LR583764	LR583969	**MW834252**	LR583874	LR583644	—
*N. silvicola*	CBS 119601 = GJS 98-135	*Populus nigra*	France	**MW834098**	**MW834171**	LR583765	LR583970	**MW834253**	LR583875	LR583645	—
	CBS 123846^T^ = GJS 04-147	*Liriodendron tulipifera*	USA	**MW834099**	**MW834172**	LR583766	LR583971	**MW834254**	LR583876	LR583646	—
*N. solani*	CBS 140079^ET^ = NRRL 66304 = GJS 09-1466 = FRC S-2364	*Solanum tuberosum*	Slovenia	MW218042	MW218088	KT313633	KT313633	MW218134	KT313623	KT313611	—
*N. spathulata*	CBS 145474^T^ = NRRL 28541 = UTHSC 98-1305	Human synovial fluid	USA	MW218045	MW218091	EU329674	EU329674	MW218137	EU329542	DQ246882	—
*N. stercicola*	CBS 142481^T^ = DSM 106211	Compost yard debris	Germany	**MW834100**	**MW834173**	LR583779	LR583984	**MW834255**	LR583887	LR583658	—
	CBS 144388 = MUCL 18299	Greenhouse humic soil	Belgium	**MW834101**	**MW834174**	LR583780	LR583985	**MW834256**	LR583888	LR583659	—
*N. suttoniana*	CBS 143214^T^ = NRRL 32858	Human wound	USA	MW218046	MW218092	DQ094617	DQ236659	MW218138	EU329630	DQ247163	—
	CBS 143224 = NRRL 54972	Equine eye	USA	MW218047	MW218093	MG189940	MG189925	MW218139	KC808336	KC808197	—
*N. tonkinensis*	CBS 115.40^T^	*Musa sapientum*	Vietnam	MW218048	MW218094	MG189941	MG189926	MW218140	LT960564	LT906672	—
	CBS 118931	*Solanum lycopersicum*	UK	MW218049	MW218095	LR583784	LR583989	MW218141	LR583891	LR583662	—
*N. tuaranensis*	NRRL 22231^T^ = ATCC 16563 = MAFF 246842	*Hevea brasiliensis* damaged by unknown ambrosia beetle	Malaysia	—	—	KC691570	KC691570	KC691600	KC691631, KC691660	KC691542	—
*N. vasinfecta*	CBS 325.54 = ATCC 16238 = IFO 7591 = IMI 251386 = NRRL 22436	Soil	South Africa	—	—	AF178412	AF178381	JX171497	JX171610	AF178348	—
	CBS 446.93 = IMI 316967 = NHL 2919	Soil	Japan	**MW834102**	**MW834175**	LR583791	LR583996	**MW834257**	LR583898	LR583670	—
	CBS 533.65 = IMI 302625	Unknown	India	**MW834103**	**MW834176**	LR583792	LR583997	**MW834258**	LR583899	LR583671	—
*Neonectria coccinea*	CBS 125484	*Fagus sylvatica*	Germany	—	—	HQ897832	MH875068	**MW834259**	HQ897785	—	—
*Ne. ditissima*	CBS 125486	*Fagus americana*	Canada	—	—	HQ897824	MH877864	—	HQ897774	—	—
*Nothofusarium devonianum*	CBS 147304^T^ = NRRL 22134	*Ruscus aculeatus*	United Kingdom	—	—	**MW827632**	**MW827673**	JX171490	JX171603	**MW834291**	—
*Pseudofusicolla belgica*	CBS 147300 = IHEM 5322	Recycled water from air-conditioning humidifier	Belgium	—	—	KJ125590	KJ126478	—	KP835473	KJ126182	—
	CBS 147301^T^ = IHEM 2413	Recycled water, spray humidifier in air-conditioned building	Belgium	—	—	KJ125588	KJ126476	—	KP835474	KJ126180	—
	CBS 147302 = IHEM 2440	Humidifier water from air-conditioning	Belgium	—	—	KJ125589	KJ126477	—	KP835475	KJ126181	—
	IHEM 2105	Recycled humidifier water from airconditioning	Belgium	—	—	KP835478	KP835480	—	KP835476	KP835484	—
*Rectifusarium robinianum*	CBS 430.91^T^ = NRRL 25729	*Robinia pseudoacacia*	Germany	—	—	KM231794	NG_058096	JX171520	JX171633	KM231923	—
*R. ventricosum*	CBS 748.79^T^ = BBA 62452 = NRRL 20846 = NRRL 22113	Wheat field soil	Germany	—	—	HQ897816	KM231658	JX171484	JX171597	KM231924	—
*Rugonectria castaneicola*	CBS 128360	Bark	China	—	—	MH864901	MH876352	**MW834260**	**MW834030**	**MW834292**	—
*Ru. neobalansae*	CBS 125120 = GJS 85-219	Dead tree	Indonesia	—	—	KM231750	HM364322	—	**MW834031**	KM231874	—
*Ru. rugulosa*	CBS 126565 = GJS 09-1245	Dead wood	Venezuela	—	—	KM231749	MH877897	**MW834261**	**MW834032**	KM231873	—
*Setofusarium setosum*	CBS 574.94 = BBA 65063	Unknown	French Guiana	—	—	**MW827633**	**MW827674**	**MW834262**	**MW834033**	**MW834293**	—
	CBS 635.92^ET^ = GJS 88-12 = NRRL 36526	Tree bark	French Guiana	—	—	**MW827634**	**MW827675**	JX171539	JX171651	**MW834294**	—
*Scolecofusarium ciliatum*	CBS 155.86 = NRRL 22284	*Hordeum vulgare* mouldy grain, associated with scale insects	Denmark	—	—	**MW827635**	**MW827676**	**MW834263**	**MW834034**	**MW834295**	—
	CBS 191.65^NT^ = ATCC 16068 = ATCC 24137 = BBA 9661 = DSM 62172 = IMI 112499 = NRRL 20431	*Fagus sylvatica*	Germany	—	—	**MW827636**	**MW827677**	**MW834264**	**MW834035**	**MW834296**	—
	CBS 144385 = IHEM 2989	*Fagus sylvatica*	Belgium	—	—	KJ125591	KJ126479	**MW834265**	KP835472	**MW834297**	—
*Stylonectria applanata*	CBS 125489	Unidentified ascomycete on *Betula* sp.	Canada	HQ897875	—	HQ897805	KM231689	—	HQ897739	KM231944	—
*St. carpini*	DAOM 235819	*Melanconis spodiaea* on *Carpinus betulus*	Austria	HQ897909	—	HQ897823	—	—	HQ897773	—	—
*St. corniculata*	CBS 125491^T^	Unidentified ascomycete on *Carpinus* sp.	Germany	HQ897915	—	HQ897829	KM231691	—	HQ897779	KM231946	—
*St. hetmanica*	CBS 147305^T^ = CPC 38725	*Diaporthe* sp. on *Frangula alnus*	Ukraine	**MW834104**	—	**MW827637**	—	—	**MW834036**	—	—
	CBS 147306 = CPC 38848	*Dothiorella sarmentorum* on *Acer platanoides*	Ukraine	**MW834105**	—	**MW827638**	—	—	**MW834037**	—	—
*St. norvegica*	CBS 139239^T^	Dead sporodochia of fusarium state on pyrenomycete (presumably *Amphiporthe* sp.)	Norway	**MW834106**	—	KR605485	—	—	**MW834038**	—	—
	CBS 139242	On sporodochia of fusarium-like on unidentified pyrenomycete	Norway	**MW834107**	—	**MW827639**	—	—	**MW834039**	—	—
*St. purtonii*	DAOM 235818	*Picea abies*	Germany	HQ897919	—	HQ897831	—	—	HQ897783	—	—
*St. qilianshanensis*	HMAS 255803^T^	Unknown ascomycete on *Picea asperata*	China	MT087289	—	—	—	—	MT087288	—	—
*St. wegelianiana*	CBS 125490	*Hapalycystis bicaudata* on *Ulmus glabra*	Austria	HQ897890	—	KM231817	KM231690	—	HQ897754	KM231945	—
*Thelonectria discophora*	CBS 125487	*Aesculus hippocastanum*	Germany	—	—	HQ897789	**MW827678**	**MW834266**	HQ897700	**MW834298**	—
*T. olida*	CBS 215.67^NT^ = ATCC 16548 = DSM 62520 = IMI 116873	*Asparagus officinalis*	Germany	—	—	**MW827640**	**MW827679**	**MW834267**	**MW834040**	**MW834299**	—
*Tumenectria laetidisca*	CBS 100284	Bamboo	Japan	—	—	KJ022017	KJ022066	—	**MW834041**	KJ022400	—
	CBS 101909^ET^	Bamboo	Jamaica	—	—	KJ022018	KJ022067	**MW834268**	**MW834042**	KJ022401	—

1 ARSEF: Collection of entomopathogenic fungal cultures, US Department of Agriculture (USDA), Agricultural Research Service (ARS), Ithaca, NY, USA; ATCC: American Type Culture Collection, Manassas, VA, USA; BBA: Biologische Bundesanstalt für Land- und Forstwirtschaft, Institut für Mikrobiologie, Berlin, Germany; CBS: Westerdijk Fungal Biodiverity Institute (WI), Utrecht, The Netherlands; CDC: Centers for Disease Control and Prevention, Atlanta, GA, USA; CECT: Spanish Type Culture Collection, Universidad de Valencia, Burjassot, Spain; CML: Coleção Micológica de Lavras, Universidade Federal de Lavras, Minas Gerais, Brazil; CMW: Culture collection at the FABI, University of Pretoria, South Africa; CPC: Collection of P.W. Crous, held at WI; DAOM: Canadian National Mycological Herbarium and Culture Collection, AAFC, Ottawa, Ontario, Canada; DSM: DSMZ-Deutsche Sammlung von Mikroorganismen und Zellkulturen GmbH, Braunschweig, Germany; FMR: Facultat de Medicina i Ciències de la Salut, Reus, Spain; FRC: Fusarium Research Center, Pennsylvannia State University, PA, USA; GJS: Collection of G.J. Samuels, USDA-ARS, USA; HKAS: Herbarium of Cryptogams, Kunming Institute of Botany, Kunming, China; HMAS: Herbarium Mycologicum Academiae Sinicae, Chinese Academy of Sciences, Beijing, China; IFO: Institute for Fermentation, Osaka, Yodogawa-ku, Osaka, Japan; IHEM: Biomedical Fungi and Yeasts Collection, Scientific Institute of Public Health, Belgium; IMI: CABI Bioscience, Egham, UK; IMUR: Institute of Mycology, University of Recife, Recife, Brazil; MAFF: Ministry of Agriculture, Forestry and Fisheries, Tsukuba, Ibaraki, Japan; MFLU: Mae Fah Luang University herbarium, Chiang Rai, Thailand; MRC: Microbial Culture Collection, South African Medical Research Council, Tygerberg, South Africa; MUCL: Mycothèque de ĺUniversité Catholique de Louvain, Louvain-la-Neuve, Belgium; NHL: National Institute of Hygienic Sciences, Tokyo, Japan; NRRL: Agricultural Research Service Culture Collection, National Center for Agricultural Utilization Research, USDA, Peoria, IL, USA; RBG: Royal Botanic Gardens Trust, Sydney, New South Wales, Australia; URM: Micoteca do Departmento de Micologia, Universidade Federal de Pernambuco, Recife, Brazil; UTHSC: Fungus Testing Laboratory, Department of Pathology, University of Texas Health Science Center, San Antonio, USA. ET: Ex-epitype; IT: Ex-isotype; NT: Ex-neotype; PT: Ex-paratype; T: Ex-type.

2 *acl1* = ATP citrate lyase; *CaM* = Calmodulin; ITS = Internal transcribed spacer region of the nrDNA; LSU = 28S large subunit of the nrDNA; *rpb1* = RNA polymerase largest subunit; *rpb2* = RNA polymerase second largest subunit; *tef1* = translation elongation factor 1-alpha; *tub2* = Beta-tubulin. Sequences generated in this study are shown in bold; Not public = sequences not available at GenBank, obtained from K. O’Donnell's alignment datasets; ∗ = Whole genome sequence contig accession numbers.

Sequences of the individual markers, including introns, were aligned using MAFFT v. 7.110 (Katoh *et al.* 2019) using default parameters and manually corrected where necessary. Seven multimarker datasets (Table 4) were assembled and analysed using Maximum Likelihood (ML) and Bayesian Inference (BI). For the ML analyses, concatenated phylogenies, where each marker was treated as a separate partition, were determined using IQ-TREE v. 2.1.2 (Nguyen *et al.* 2015, Minh *et al.* 2020b) with ultrafast bootstrapping (UFBoot2; Hoang *et al.* 2018) for estimation of branch support. The most suitable evolutionary model for each partition was estimated using ModelFinder (Kalyaanamoorthy *et al.* 2017; Minh *et al.* 2020b) as implemented in IQ-TREE. To assess whether the individual markers were compatible, genealogical concordance factors (gCF) were calculated using IQ-TREE (Minh *et al.* 2020a, b). Additional ML analyses were performed using RAxML v. 8.2.12 (randomised accelerated (sic) maximum likelihood for high performance computing; Stamatakis 2014) with the system's default modelling options. The robustness of the analysis was evaluated by bootstrap support (BS) with the number of bootstrap replicates automatically determined by the software. The BI analyses were carried out through the CIPRES website (http://www.phylo.org) using MrBayes v. 3.2.7a (Ronquist & Huelsenbeck 2003) incorporating the best evolutionary models for each marker as determined by MrModeltest v. 2.3 (Nylander 2004). Two parallel Markov Chain Monte Carlo (MCMC) runs of four incrementally heated chains (temp parameter = 0.2) were run starting from a random tree topology. The MCMC analyses lasted for 5M generations, and convergence of the runs was checked by average standard deviation of split frequencies below 0.01. Trees were saved every 1 000 generations and the first 25 % of saved trees were discarded as the “burn-in” phase. Posterior probabilities (PP) were determined from the remaining trees. Proper mixing of the MCMC runs was further confirmed by checking that all chains converged (minimum and average Estimated Sampled Size [ESS >200], Potential Scale Reduction Factor [PSRF = 1.0]) and by plotting and analysing trace file results using Tracer v.1.7.1 (Rambaut *et al.* 2018).

**Table 4:** Summary of phylogenetic information generated in this study.

Analysis	Nuclear region	Length + gap	PI	Var.	BI unique site patterns	Model (AIC)	Model (BIC)	ML -InL (IQ)
Generic delimitation	ITS	626	249	310	378	GTR+I+G	TIMe+I+G4	-3099.276
	LSU	435	90	109	118	GTR+I+G	TIM2+F+I+G4	-15223.682
	*rpb1*	1 371	705	755	823	GTR+I+G	TIM3e+I+G4	-27263.487
	*rpb2*	1 761	834	892	989	GTR+I+G	GTR+F+I+G4	-8493.378
	*tef1*	699	448	489	551	GTR+I+G	TIM2e+I+G4	-40875.16
	Combined	4 892	2 326	2 555	2 859	n/d	n/d	-94954.982
Ex-type strains	*rpb1*	1 724	980	550	1 358	GTR+I+G	TIM3e+R4	-37377.092
	*rpb2*	1 789	788	916	1 056	GTR+I+G	TIM2e+R6	-44286.314
	*tef1*	859	463	301	700	GTR+I+G	GTR+F+I+G4	-25546.628
	Combined	4 372	2 231	1 767	3 114	n/d	n/d	-113450.62
*Fusarium fujikuroi* species complex	*CaM*	545	76	131	150	SYM+G	G4TNe+G4	-4032.663
	*rpb1*	1 534	201	340	344	SYM+G	TIM2e+G4	-5669.761
	*rpb2*	1 541	241	362	365	GTR+I+G	TNe+G4	-7415.729
	*tef1*	676	137	243	305	GTR+I+G	TNe+I+G4	-2062.906
	*tub2*	488	76	150	182	SYM+G	TNe+G4	-1930.688
	Combined	4 794	731	1 226	1 346	n/d	n/d	-22043.423
*Fusicolla*	*acl1*	908	153	346	241	GTR+G	TNe+I	-3238.214
	ITS	518	54	111	128	GTR+I+G	TIM2e+I+G4	-1704.698
	LSU	476	34	69	72	K80+I	K80+R2	-1229.69
	*rpb2*	1 702	258	447	359	SYM+I+G	TIM2e+G4	-5692.247
	*tef1*	476	109	216	202	SYM+I	TIM2+F+G4	-2051.471
	*tub2*	484	83	162	159	GTR+G	K80+G4	-1780.157
	Combined	4 564	691	1 351	1 161	n/d	n/d	-16092.82
*Macroconia*	*acl1*	801	207	332	205	SYM+I	K80+I	-1241.031
	*CaM*	551	150	223	159	K80+I	K80+I	-2092.487
	ITS	540	36	64	94	GTR+I	TNe+G4	-2259.518
	LSU	694	21	37	3	GTR+I	TNe+I	-3097.338
	*rpb1*	814	116	182	96	SYM+G	TNe+G4	-2620.526
	*rpb2*	778	160	618	151	SYM+I	TNe+G4	-1784.381
	*tub2*	519	101	168	142	SYM+G	TNe+G4	-1205.535
	Combined	4 697	791	1 624	850	n/d	n/d	-14388.257
*Neocosmospora*	*acl1*	630	173	271	297	K80+I+G	TIM3e+I+G4	-13572.514
	*CaM*	586	171	231	280	HKY+I+G	TIM2e+R3	-5595.928
	ITS	476	119	357	211	GTR+I+G	TNe+G4	-4164.678
	LSU	482	36	63	76	GTR+I+G	TIM3e+I+G4	-10056.777
	*rpb1*	1 492	390	506	636	GTR+I+G	TIM2e+R3	-2888.743
	*rpb2*	1 613	449	564	621	GTR+I+G	TIM2e+I+G4	-1496.116
	*tef1*	688	230	323	370	GTR+I+G	K80+G4	-4087.046
	Combined	5 967	1 568	2 315	2 491	n/d	n/d	-46528.083
*Stylonectria*	*acl1*	897	254	426	416	GTR+G	K80+I	-1022.317
	ITS	544	21	39	47	HKY+I	TNe+G4	-5181.494
	*rpb2*	1 631	183	442	299	GTR+G	TNe+G4	-4061.543
	Combined	3 072	458	907	762	n/d	n/d	-10441.718

PI = parsimony informative characters; Var. = variable characters; BI = Bayesian inference; Model (AIC) = evolutionary model selected by MrModeltest; Model (BIC) = evolutionary model selected by ModelFinder in IQ-TREE; ML -InL (R) = best tree score determined using RAxML; ML -InL(IQ) = best tree score determined in IQ-TREE. F = Empirical base frequencies; G = Rate of discrete Gamma categories; GTR = General time reversible model; HKY = Unequal transition/transversion rates and unequal base frequencies; I = Proportion of invariable sites; K80 = Unequal transition/transversion rates and equal base frequencies; R = FreeRate model; SYM = Symmetric model; TIM2 = Transition model, AC = AT, CG = GT and unequal base frequencies; TIM2e = TIM2 with equal base frequencies; TIM3e = Transition model, AC = CG, AT = GT with equal base frequencies; TNe = Unequal transition/transversion rates with unequal purine/pyrimidine rates and equal base frequencies; TPM2 = AC = AT, AG = CT, CG = GT and equal base frequencies.

The phylogenetic re-analysis of the dataset presented by Geiser *et al.* (2021) was first made according to the original exons-only alignment file and procedures as indicated in Geiser *et al.* (2021) (Supplementary Table S1). Additionally, the dataset was split into the 19 genes according to the original partitioning file, and every gene was realigned using the MAFFT webserver (v. 7, Katoh *et al.* 2019) applying the G-INS-i algorithm. All other parameters were set to default. Six of the 19 genes exhibited a diverging alignment length. No subsequent changes were done to the alignments. The sequences were merged using BioEdit (v. 7.2.5, Hall 1999), and the phylogenetic trees were calculated using Minimum evolution (ME) and ML algorithms, and BI. The ME tree was calculated using FastTree 2 (Price *et al.* 2010) using standard settings and 1 000 bootstraps (Felsenstein 1985). The ML analysis was done using RAxML (v. 8.2.12, Stamatakis 2014) with the generalized time-reversible (GTR) model and applying the partitioning option, which estimates the Gamma-shape parameter and the proportion of invariable sites for every gene separately. Again 1 000 bootstraps were calculated to estimate branch support. Bayesian inference was conducted using MrBayes v. 3.2.7 (Ronquist & Huelsenbeck 2003) with the partitioned dataset. The Gamma-shape parameter and proportion of invariable sites were estimated independently for each partition. MrBayes was run for 5 M generations with every 500^th^ tree sampled and a burn-in of 30 % of the sampled trees to ensure sampling from the stationary phase. All other parameters were set to default.

### Morphology

Morphological characterisation followed standard procedures as described by Leslie & Summerell (2006) using PDA, SNA (Nirenberg 1976), and CLA (Fisher *et al.* 1982). Colony morphology and pigmentation were evaluated on PDA after 7 to 14 d at 25 °C in darkness. Colour notation was based on the colour charts of Rayner (1970). Fungarium specimens were rehydrated in 3 % aqueous KOH for a few minutes and then rinsed by replacing the KOH solution with sterile distilled water or 100 % lactic acid (Samuels 1976a, b, Samuels *et al.* 1990). Unless otherwise mentioned, micromorphological characters were examined using water as mounting medium on a Zeiss Axioskop 2 plus or a Nikon Eclipse 80i, both equipped with Differential Interference Contrast (DIC) optics and a Nikon AZ100 dissecting microscope all fitted with Nikon DS-Ri2 high-definition colour digital cameras to photo-document fungal structures. Measurements were taken using the Nikon software NIS-elements D v. 4.50. The dimensions of at least 30 randomly selected elements were recorded for every fungal structure. Average, standard deviation, and maximum–minimum values were determined for elements using five or more individual measurements. To facilitate the comparison of relevant micro- and macroconidial features, composite photo plates were assembled from separate photo micrographs using Adobe Photoshop CC.

## Results

### DNA phylogeny

The results of DNA evolutionary model selection, alignment length, and composition as well as tree statistics for all the multimarker datasets included in this study are summarised in Table 4.

*Re-analysis of the dataset of**Geiser et al. (2021)**:* A re-analysis of the dataset of Geiser *et al.* (2021) revealed no major differences in the ML analysis. However, in ME analysis (Supplementary Fig. S3), we found that the backbone architecture is less solid than previously thought and a large monophyletic clade containing *Neocosmospora*, *Albonectria*, and several other genera formed as sister group to *Fusarium s. str.* with strong support.

**Fig. 7:**
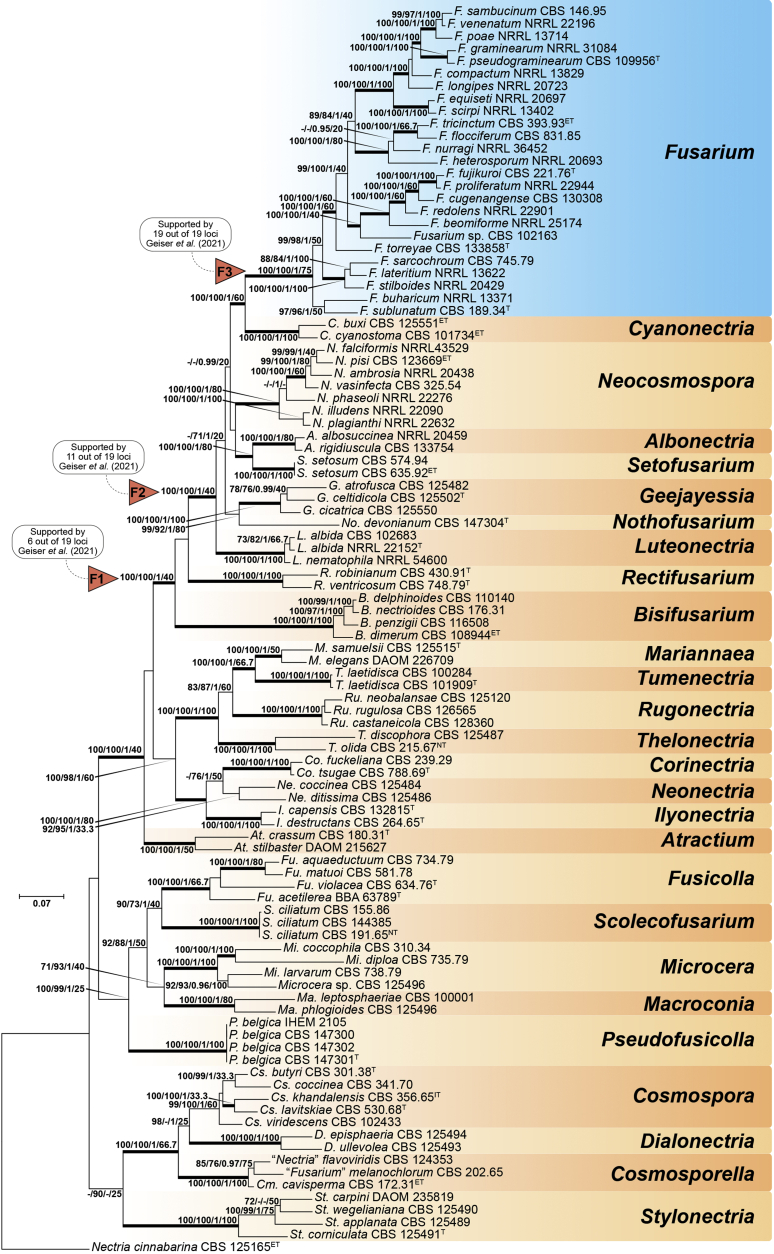
Maximum-Likelihood (IQ-TREE-ML) consensus tree inferred from the combined ITS, LSU, *rpb1*, *rpb2* and *tef1* multiple sequence alignment of members of *Nectriaceae*. Numbers at the branches indicate support values (RAxML-BS / UFboot2-BS / BI-PP / gCF) above 70 % / 0.95 with thickened branches indicating full support (RAxML-BS / UFboot2-BS / gCF = 100 %; BI-PP = 1). The scale bar indicates expected changes per site. The tree is rooted to *Nectria cinnabarina* (CBS 125165). Arrows “F1”, “F2” and “F3” indicate the three alternative *Fusarium* hypotheses *sensu*Geiser *et al.* (2013). Ex-epitype, ex-isotype, ex-neotype and ex-type strains are indicated with ET, IT, NT, and T, respectively.

*Generic delimitation of fusarioid taxa in* Nectriaceae: The analyses included nectriaceous taxa historically ascribed to *Fusarium s. lat.*, including several recently segregated fusarioid genera (Gräfenhan *et al.* 2011, Schroers *et al.* 2011, Lombard *et al.* 2015), cylindrocarpon-like taxa (Chaverri *et al.* 2011), and the closely related – although morphologically distinct – phylogenetic relatives *Cosmospora* and *Mariannaea*. Analyses using ML and BI of the individual genes and combined datasets resulted in phylogenies with congruent topologies. Therefore, only IQ-TREE-ML topologies are presented with RAxML-BS, UFboot2-BS, BI-PP and gCF support values superimposed (Fig. 7).

The combined alignment of ITS, LSU, *rpb1*, *rpb2* and *tef1* comprised 100 strains representing 92 species, including the outgroup *Nectria cinnabarina* (CBS 125165). Phylogenetic analyses resolved 27 monophyletic genera, of which 19 contain taxa with fusarioid asexual morphs and nectria- or cosmospora-like sexual morphs. Of these, 15 clades represent currently described genera, namely *Albonectria*, *Atractium*, *Bisifusarium*, *Cosmosporella*, *Cyanonectria*, *Dialonectria*, *Fusarium*, *Fusicolla*, *Geejayessia*, *Macroconia*, *Microcera*, *Neocosmospora*, *Pseudofusicolla*, *Rectifusarium*, and *Stylonectria*. The fusarioid genera *Cosmosporella* and *Dialonectria*, both of which have cosmospora-like sexual morphs, clustered as sister clades to *Cosmospora*; the latter, however, differ by having acremonium-like asexual morphs. The remaining four clades with fusarioid morphology represent undescribed taxa, formally described here as the new genera *Luteonectria*, *Nothofusarium*, *Scolecofusarium*, and *Setofusarium*. A strongly supported clade comprising six cylindrocarpon-like genera (*Corinectria*, *Ilyonectria*, *Neonectria*, *Rugonectria*, *Thelonectria*, and *Tumenectria*) and the genus *Mariannaea* resolved as successive sister groups to the F1 node.

Twenty-four out of the 27 genera included in the analysis resolved as fully supported clades, including all but one (*Nothofusarium* with RAxML-BS = 99 % / UFboot-BS = 92 % / PP = 1) of the fusarioid genera (Fig. 7). The two remaining clades (*Cosmospora* and *Neonectria*), however, received high statistical support (RAxML-BS = 99 % / UFboot-BS = 100 % / PP = 1 and RAxML-BS = 92 % / UFboot-BS = 95 % / PP = 1, respectively). Similarly, the combined phylogeny resolved most of the internal nodes with high to full bootstrap and Bayesian PP support including the nodes F1, F2, and F3 *sensu* Geiser *et al.* (2013, 2021) and O'Donnell *et al.* (2013, 2020). Nevertheless, only F3 was resolved with confidence by all the individual marker phylogenies (Supplementary Fig. S4). Node F2 was resolved with high statistical support in the ITS, *rpb1*, and *tef1* phylogenies, but unsupported in the LSU and *rpb2* trees, while node F1 resolved without bootstrap and PP support in the ITS, *rpb1*, *rpb2*, and *tef1* phylogenies and was not recovered in the LSU tree.

**Fig. 8:**
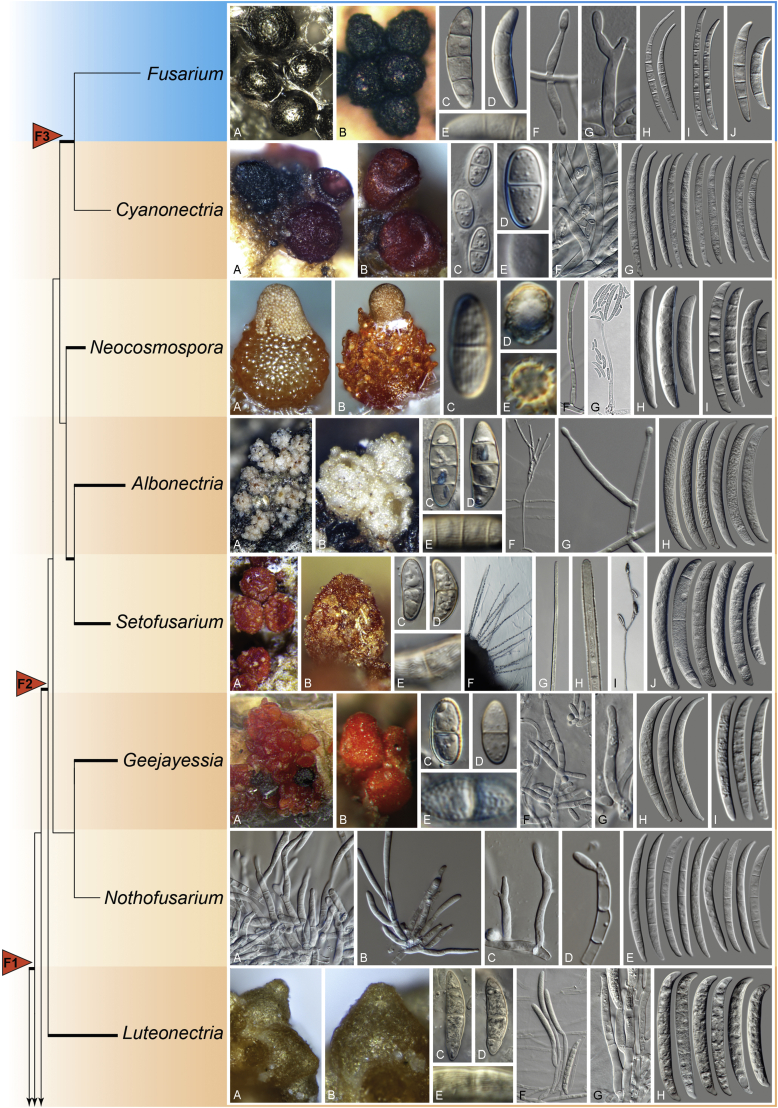

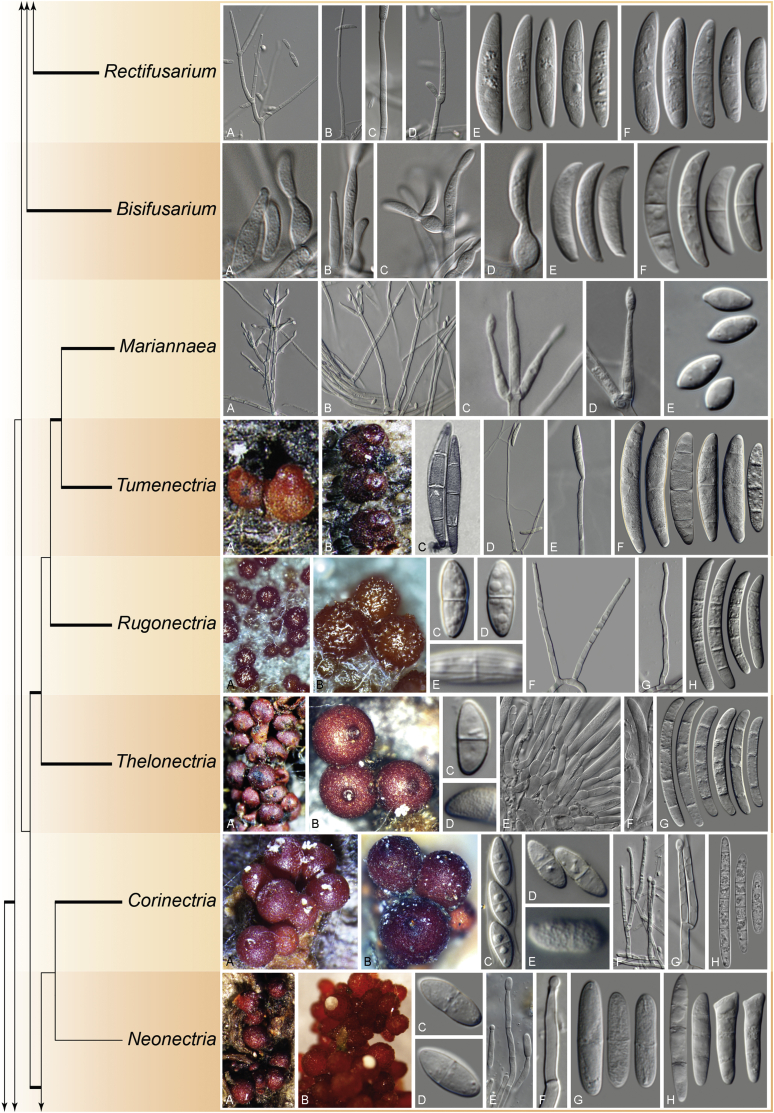

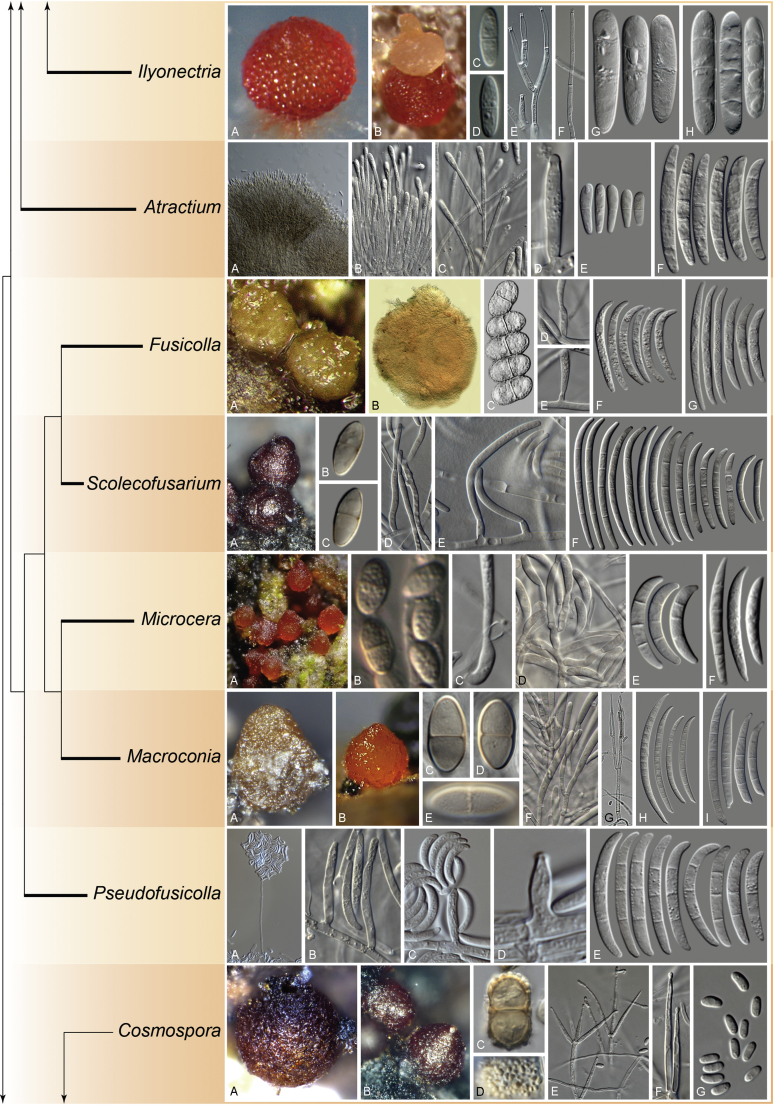

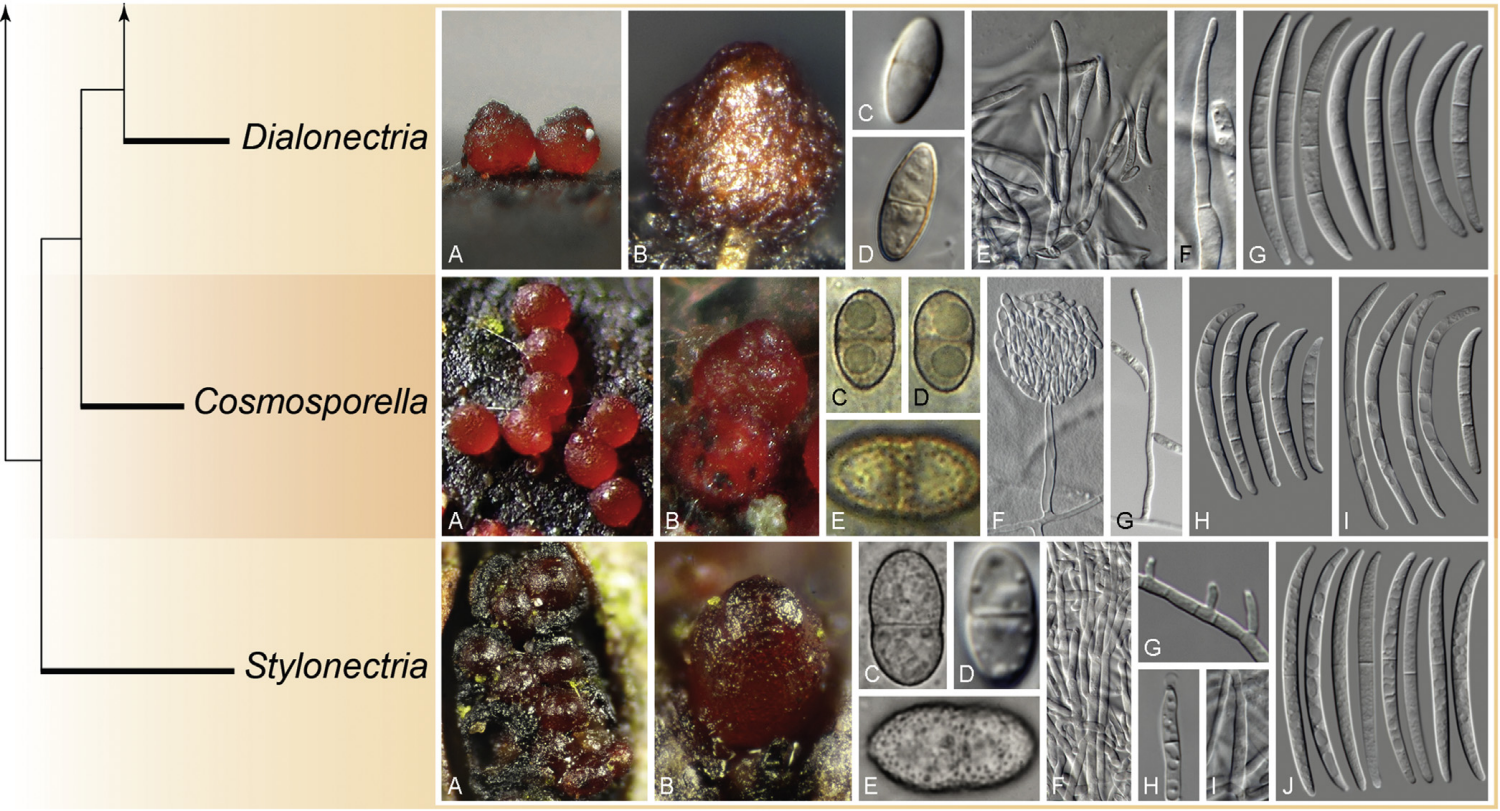
Morphological features and phylogenetic affinities of fusarioid genera of *Nectriaceae* and close relatives. The tree was delineated based on the phylogeny presented in Fig. 7 and does not indicate phylogenetic distances. Fully supported branches are indicated in **bold**. The genus *Fusarium* is indicated in blue. Arrows “F1”, “F2” and “F3” indicate the three alternative *Fusarium* hypotheses *sensu*Geiser *et al.* (2013). ***Fusarium*.** A, B. Ascomata. C–E. Ascospores. F, G. Conidiogenous cells. H–J. Macroconidia. (B. Adapted from Schroers *et al.* 2011). ***Cyanonectria*.** A, B. Ascomata. C–E. Ascospores. F. Conidiogenous cells. G. Macroconidia. ***Neocosmospora*.** A, B. Ascomata. C–E. Ascospores. F, G. Conidiogenous cells. H, I. Macroconidia. [A. Adapted from Sandoval-Denis & Crous (2018). G. Adapted from Sandoval-Denis *et al.* (2019)]. ***Albonectria*.** A, B. Ascomata. C–E. Ascospores. F, G. Conidiophores and conidiogenous cells. H. Macroconidia. ***Setofusarium*.** A, B. Ascomata. C–E. Ascospores. F–H. Setae formed on sporodochia. I. Conidiophore. J. Conidia. ***Geejayessia*.** A, B. Ascomata. C–E. Ascospores. F, G. Conidiophores and conidiogenous cells. H, I. Macroconidia. [A. Adapted from Schroers *et al.* (2011)]. ***Nothofusarium*.** A–D. Conidiophores and conidiogenous cells. E. Conidia. ***Luteonectria*.** A, B. Ascomata. C–D. Ascospores. F, G. Conidiophores and conidiogenous cells. H. Conidia. ***Rectifusarium*.** A–D. Conidiophores and conidiogenous cells. E, F. Conidia. ***Bisifusarium*.** A–D. Conidiophores and conidiogenous cells. E, F. Conidia. ***Mariannaea*.** A, B. Conidiophores. C, D. Conidiogenous cells. E. Conidia. ***Tumenectria*.** A, B. Ascomata. C. Ascospores. D, E. Conidiophores and conidiogenous cells. F. Conidia. [A–C. Adapted from Salgado-Salazar *et al.* (2016)]. ***Rugonectria*.** A, B. Ascomata. C–E. Ascospores. F, G. Conidiophores and conidiogenous cells. H. Conidia. ***Thelonectria*.** A, B. Ascomata. C, D. Ascospores. E, F. Conidiophores and conidiogenous cells. G. Conidia. ***Corinectria*.** A, B. Ascomata. C–E. Ascospores. F, G. Conidiophores and conidiogenous cells. H. Conidia. (H. Picture by C. González). ***Neonectria*.** A, B. Ascomata. C, D. Ascospores. E, F. Conidiophores and conidiogenous cells. G, H. Conidia. [A. Adapted from Chaverri *et al.* (2011)]. ***Ilyonectria*.** A, B. Ascomata. C, D. Ascospores. E, F. Conidiophores and conidiogenous cells. G, H. Conidia. ***Atractium*.** A, B. Conidiophores. C, D. Conidiogenous cells. E, F. Conidia. ***Fusicolla*.** A, B. Ascomata. C. Ascospores. D, E. Conidiogenous cells. F, G. Conidia. (A–C. Pictures by C. Lechat). ***Scolecofusarium*.** A. Ascomata. B, C. Ascospores. D, E. Conidiophores and conidiogenous cells. F. Conidia. ***Microcera*.** A. Ascomata. B. Ascospores. C, D. Conidiogenous cells. E, F. Conidia. (A, B. Pictures by N. Aplin, Fungi of Great Britain and Ireland). ***Macroconia*.** A, B. Ascomata. C–E. Ascospores. F, G. Conidiophores and conidiogenous cells. H, I. Conidia. (B. Picture by P. Mlčoch). ***Pseudofusicolla*.** A, B. Conidiophores and conidiogenous cells. C, D. Conidia. [A–D. Adapted from Triest *et al.* (2016)]. ***Cosmospora*.** A, B. Ascomata. C, D. Ascospores. E, F. Conidiophores and conidiogenous cells. G. Conidia. ***Dialonectria*.** A, B. Ascomata. C–E. Ascospores. F, G. Conidiophores and conidiogenous cells. H. Conidia. (A. Picture by P. Mlčoch). ***Cosmosporella*.** A, B. Ascomata. C–E. Ascospores. F, G. Conidiophores and conidiogenous cells. H, I. Conidia. (A–E. Pictures by P. Mlčoch). ***Stylonectria*.** A, B. Ascomata. C–E. Ascospores. F–I. Conidiophores and conidiogenous cells. J. Conidia. (A–C, E. Pictures by B. Wergen).

To illustrate shared and differential morphological characters among the different genera recognised here, a tree was constructed based on the phylogeny presented in Fig. 7, and the main morphological features were plotted for each clade/genus (Fig. 8). In addition to the genera recognised above, the recently described aquatic fusarioid genus *Varicosporella* (Lechat & Fournier 2015) is not included in the phylogenetic analyses due to lack of available sequences; however, is accepted here based on its distinct morphology. Non-molecular character variation supports the phylogenetic relationship of fusarioid taxa in *Nectriaceae*. The 20 fusarioid genera in *Nectriaceae* are characterised by phialidic asexual morphs with variously septate, falcate conidia with diverse degrees of foot-shaped basal cell development, formed on aerial or sporodochial conidiophores, with or without additional production of microconidia. Characteristic macroconidial foot-shaped basal cells are found most of the time, but not always (*e.g*., *Fusarium caeruleum*) in clade F1, *i.e., Albonectria*, *Bisifusarium*, *Cyanonectria*, *Fusarium*, *Geejayessia*, *Luteonectria*, *Neocosmospora*, *Nothofusarium*, *Rectifusarium*, and *Setofusarium,* but are also present in distantly related genera such as *Cosmosporella*, *Dialonectria*, *Macroconia*, and *Microcera*. *Setofusarium* is clearly recognisable by the formation of thick-walled, slightly rugose setae on its sporodochia.

With the exception of *Atractium*, *Bisifusarium*, *Nothofusarium*, and *Pseudofusicolla*, most fusarioid genera have sexual morphs, usually seen as nectria-like or cosmospora-like perithecial ascomata. The ascomata show various colour reactions or no reaction in KOH; the colour reaction correlates with the phylogenetic distribution. Apart from *Albonectria*, with white to pale yellow perithecia, *Luteonectria*, with white to buff coloured perithecia and *Fusarium*, with dark blue-violet to black perithecia, *Fusicolla*, with yellow-orange perithecia and *Varicosporella*, with yellow perithecia, the rest of fusarioid genera all present orange to red perithecial ascomata. Going beyond this prototypical group, perithecia of *Cyanonectria* species are often unequally red to dark blue, while those of *Geejayessia* can be bright red or black. Anatomically, two types of perithecial walls can be distinguished among the known fusarioid genera, based on wall thickness: thin-walled perithecia, in which a single region can be identified, and thick-walled perithecia, on which distinctive inner and outer regions can be recognised (but see Schroers *et al.* 2011 for differing interpretations). The former is seen in *Cosmosporella*, *Cyanonectria*, *Dialonectria*, *Fusicolla*, *Geejayessia*, *Luteonectria*, *Macroconia*, *Microcera*, *Scolecofusarium*, and *Varicosporella*; and the latter is found in *Albonectria*, *Fusarium*, *Neocosmospora*, *Rectifusarium*, *Setofusarium* and *Stylonectria*. With the exception of *Rectifusarium* and *Stylonectria*, the perithecial surface of the thick-walled genera is typically warted; nevertheless, those of *Setofusarium* often present additional scaly protrusions, while smooth perithecia can be rarely found in *Neocosmospora* (*i.e.*, *N. vasinfecta*). Additionally, both *Cyanonectria* and *Geejayessia* most commonly have smooth perithecial walls. The remaining genera, that is *Cosmosporella*, *Dialonectria*, *Fusicolla*, *Luteonectria*, *Macroconia*, *Microcera*, *Rectifusarium*, *Scolecofusarium*, *Stylonectria*, and *Varicosporella*, all form smooth-walled perithecia.

Significant variation also exists among fusarioid genera regarding ascospore characteristics. Most genera consistently form 1-septate ascospores. These are seen in *Cosmosporella*, *Cyanonectria*, *Dialonectria*, *Fusicolla*, *Geejayessia*, *Macroconia*, *Microcera*, *Rectifusarium*, *Scolecofusarium*, *Setofusarium*, *Stylonectria*, and *Varicosporella*. Except for *Cyanonectria*, in which the ascospores remain hyaline and smooth; *Setofusarium*, in which the ascospores surface is finely striated, and *Varicosporella*, in which the ascospore surface is ribbed, ascospores of the above-mentioned genera are often pale yellow to pale brown and smooth at first, becoming finely spinulose or tuberculate. The genus *Neocosmospora* forms (0–)1-septate, yellow-brown ascospores, which are often markedly striate, or more rarely cerebriform (*i.e*., *N. vasinfecta*) or spiny (*i.e*., *N. spinulosa*). *Albonectria* and *Luteonectria* form characteristic 3-septate, pale yellow-brown, faintly striate ascospores, while *Fusarium* produces 1–3-septate, hyaline to pale yellow-brown and smooth ascospores.

**Fig. 9:**
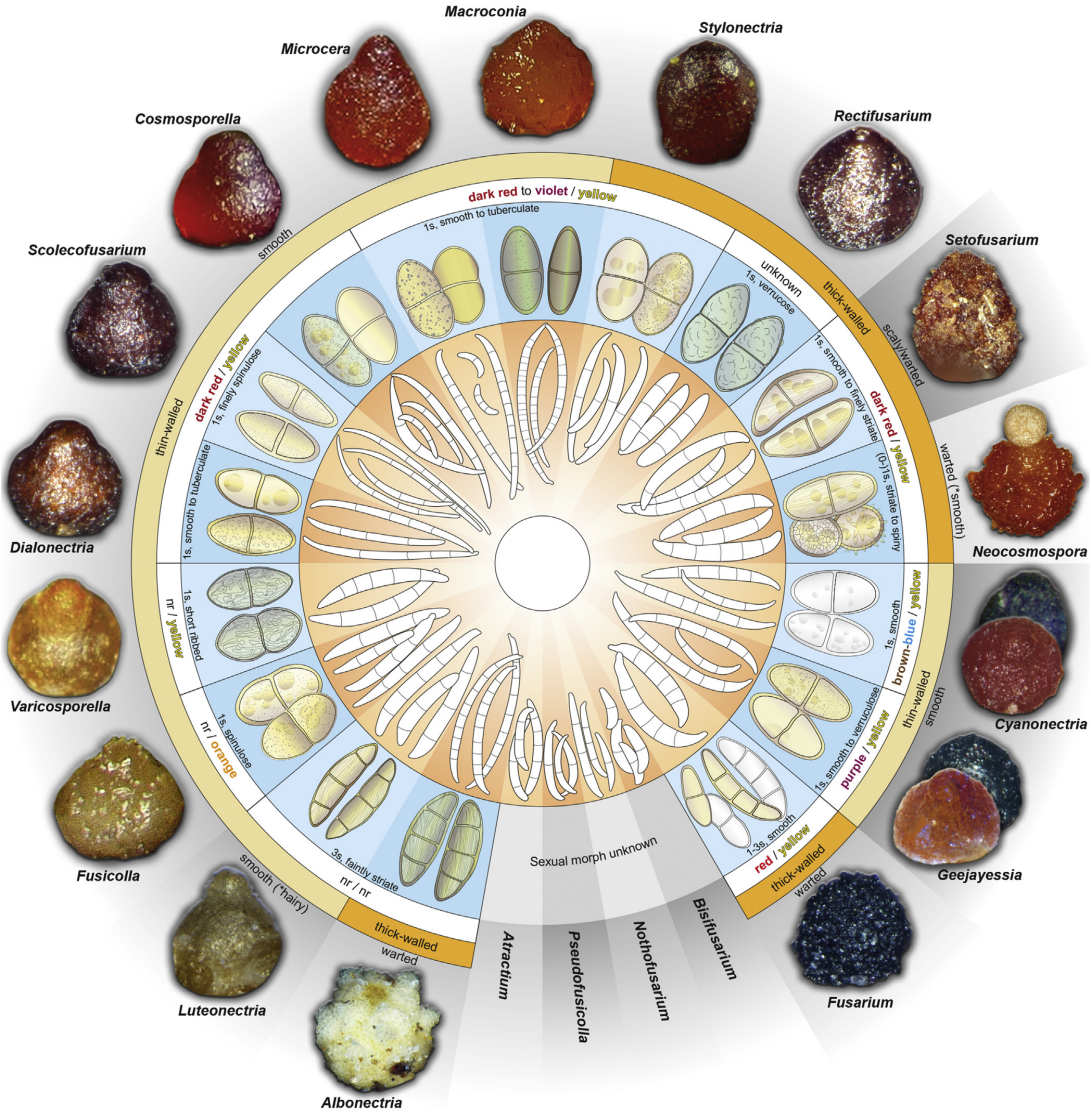
Characters for morphological identification of fusarioid genera in *Nectriaceae*. The rings show, from inside to outside: conidial morphology; ascospore morphology, septation and surface; colour reaction of ascomata in 3 % KOH/lactic acid (nr = no reaction); ascomata wall thickness; and general colour, appearance and wall surface of ascomata.

Based on the morphological variation observed in these taxa, an identification scheme is presented for fusarioid genera of the *Nectriaceae* (Fig. 9)*.*

**Fig. 10:**
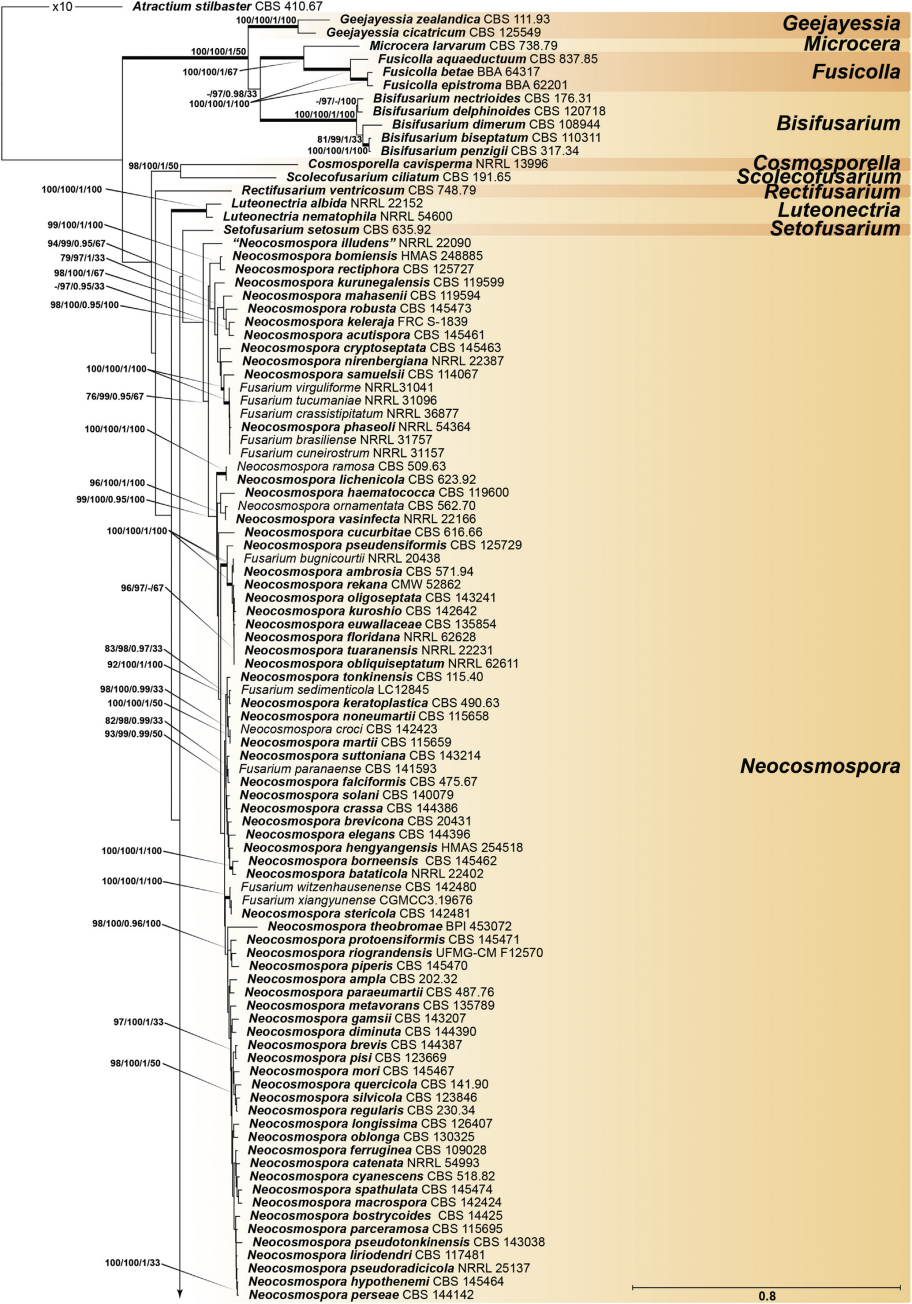

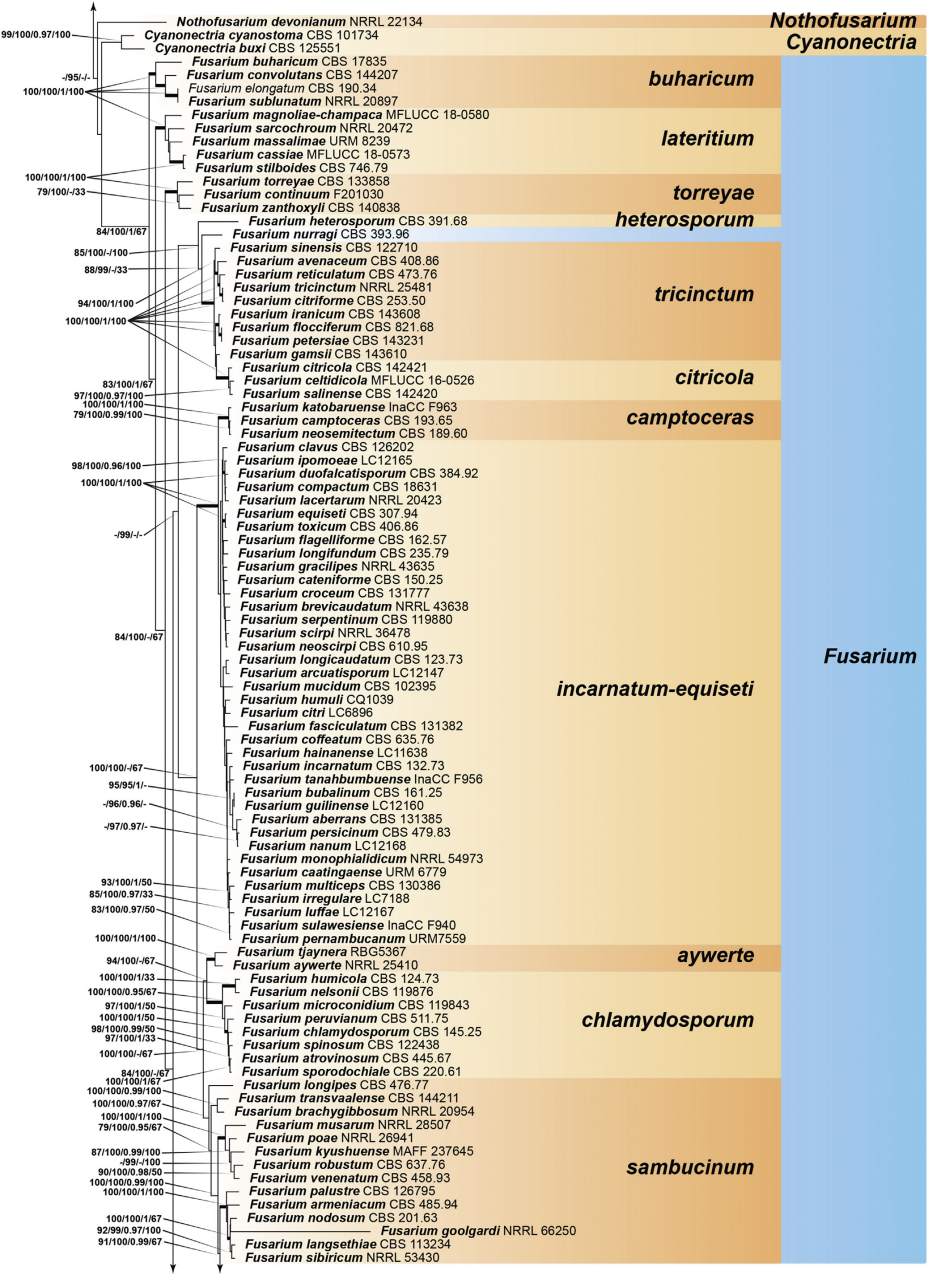

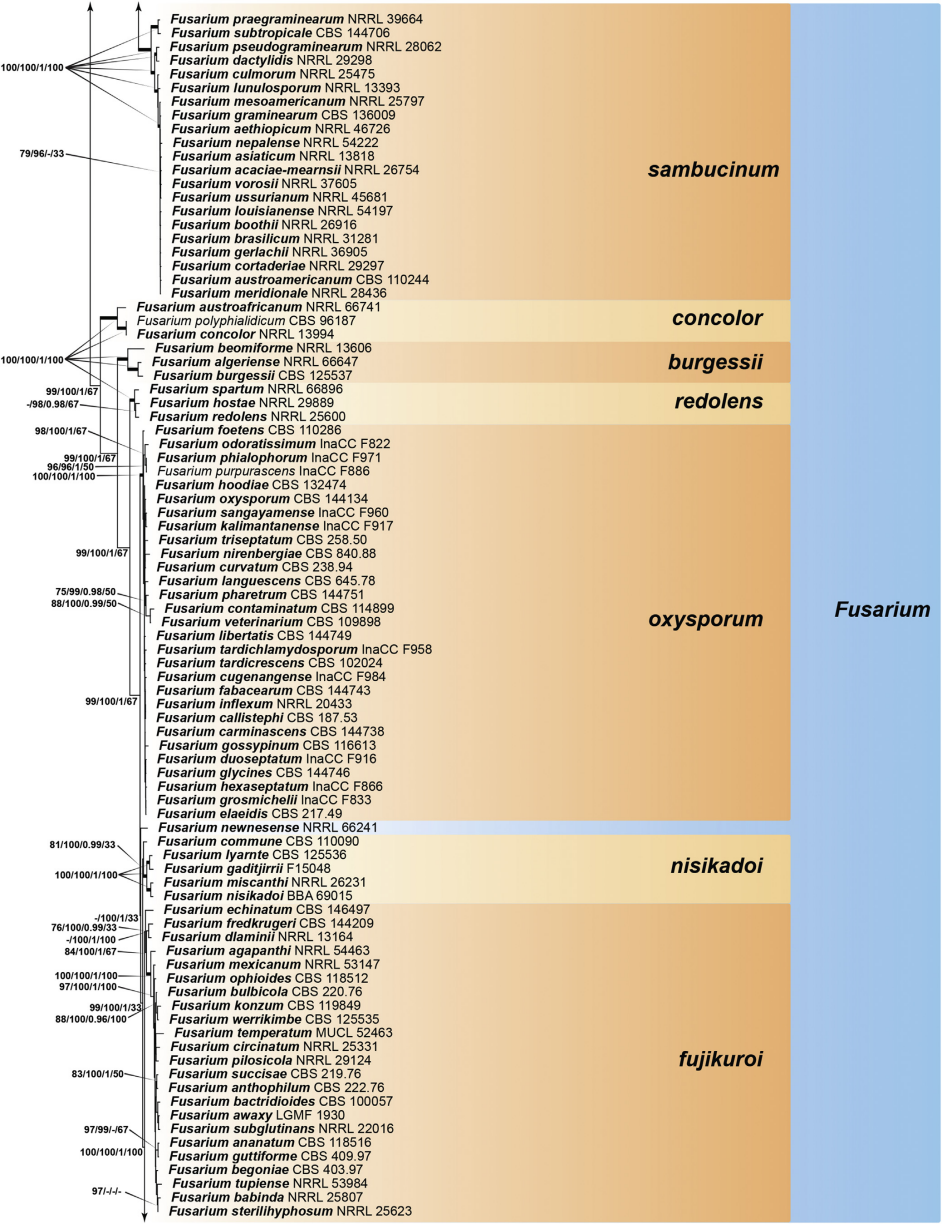

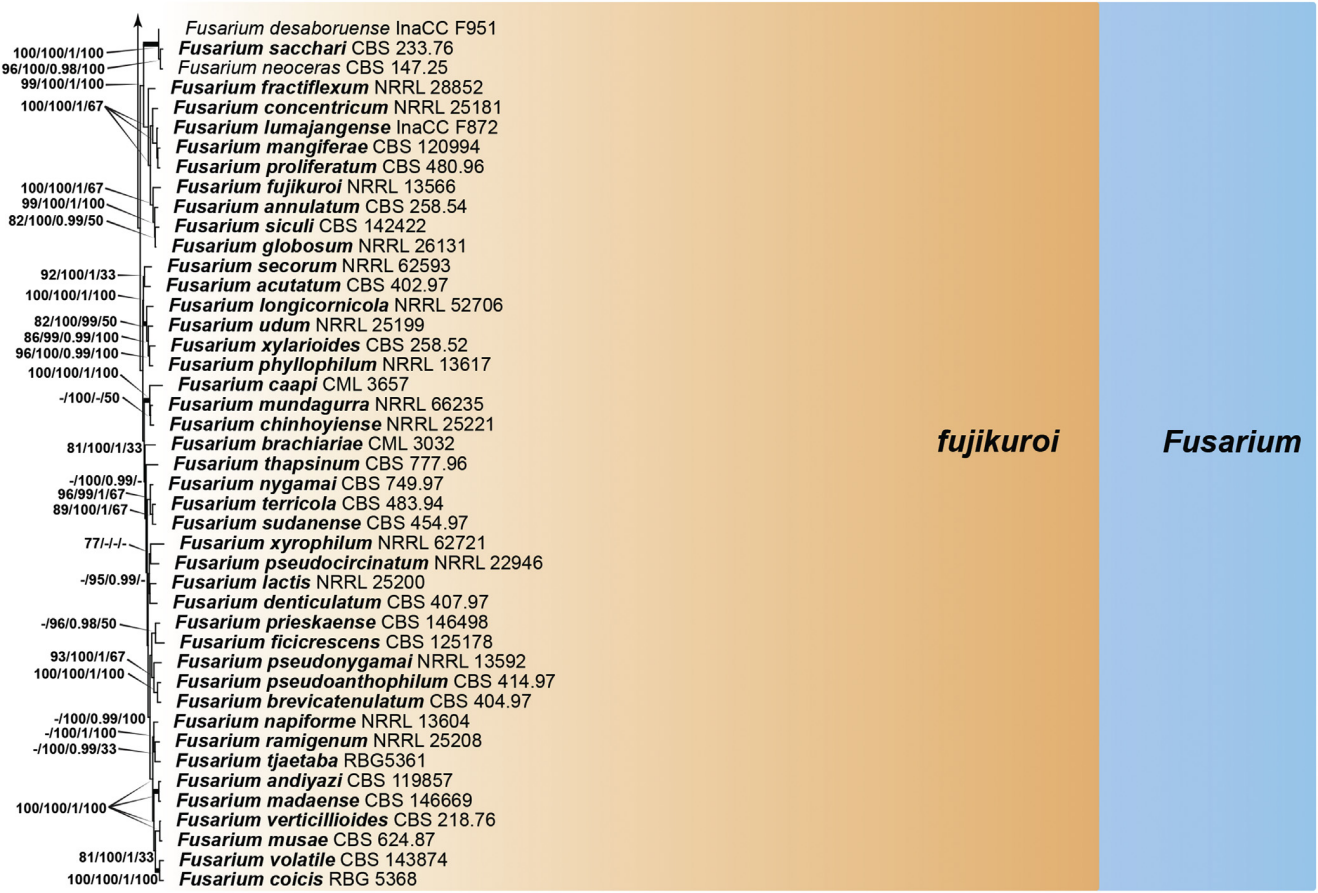
Maximum-Likelihood (IQ-TREE-ML) consensus tree inferred from the combined *rpb1*, *rpb2* and *tef1* sequence alignment of the living type strains as indicated in the nomenclator list. Numbers at the branches indicate support values (RAxML-BS / UFboot2-BS / BI-PP) above 70 % / 0.95 with thickened branches indicating full support (RAxML-BS / UFboot2-BS = 100 %; BI-PP = 1). The scale bar indicates expected changes per site. The tree is rooted to *Atractium stilbaster* (CBS 410.67). Names indicated in **bold** are in current use. Subdivision of the *Fusarium* clade (blue block) represent the recognised species complexes.

*Ex-type strain phylogeny*: The analyses included partial *rpb1*, *rpb2* and *tef1* sequences of only the ex-, epi- and neotype strains as indicated in the nomenclator list of all the names that have been introduced in *Fusarium*. The analyses used both ML inferences and BI of the individual genes and combined datasets, and they resulted in phylogenies with congruent topologies. Therefore, the RAxML topology is presented with RAxML-BS, UFboot2-BS, BI-PP and gCF support values superimposed (Fig. 10).

The combined alignment comprised 325 strains from 309 species of 14 fusarioid genera including *Atractium stilbaster* (CBS 410.67) as the outgroup. A total of 14 fusarioid genera were resolved of which six (*Cosmosporella*, *Microcera*, *Nothofusarium*, *Rectifusarium*, *Scolecofusarium*, and *Setofusarium*) were represented by single lineages, mostly due to a lack of living isolates directly linked to type material available for other species recognised within these genera at present. The genera *Fusarium* (224 strains; 220 accepted species) and *Neocosmospora* (83 strains; 71 accepted species) both represented the largest sampling of living isolates directly linked to type material available. The remaining five genera were represented by two or more strains and include *Bisifusarium* (five species and strains), *Cyanonectria* (two species and strains), *Fusicolla* (three species and strains), *Geejayessia* (two species and strains), and *Luteonectria* (two species and strains).

In order to describe novel species found for the genera treated in this study, additional phylogenies were constructed for the *Fusarium fujikuroi* species complex (FFSC), *Fusicolla*, *Macroconia*, *Neocosmospora*, and *Stylonectria*.

**Fig. 11:**
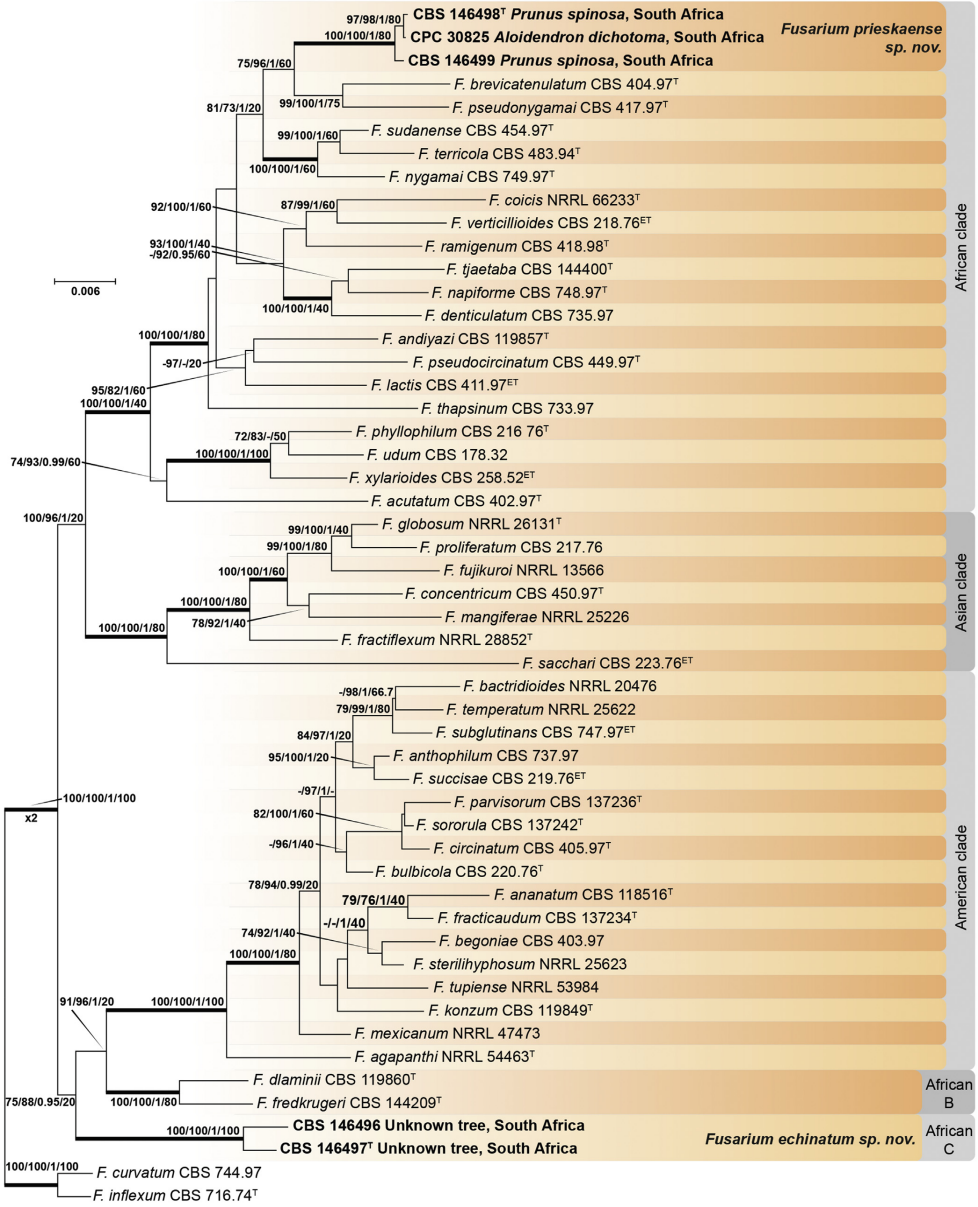
Maximum-Likelihood (IQ-TREE-ML) consensus tree inferred from the combined *CaM*, *rpb1*, *rpb2*, *tef1*, and *tub2* sequence alignment of members of the *Fusarium fujikuroi* species complex. Numbers at the branches indicate support values (RAxML-BS / UFboot2-BS / BI-PP) above 70 % / 0.95 with thickened branches indicating full support (RAxML-BS / UFboot2-BS = 100 %; BI-PP = 1). Novel taxa are indicated in **bold**. The scale bar indicates expected changes per site. The tree is rooted to *Fusarium curvatum* CBS 744.97 and *Fusarium inflexum* CBS 716.74. Ex-epitype, ex-neotype and ex-type strains are indicated with ET, NT, and T, respectively.

Fusarium fujikuroi *SC phylogeny*: The analyses included partial sequences of five genes (*CaM*, *rpb1*, *rpb2*, *tef1* and *tub2*) from 52 strains representing 46 species of the FFSC, and two outgroup taxa (*F. curvatum* CBS 744.97 and *F. inflexum* CBS 716.74) (Fig. 11). The analysis of the combined dataset fully supported five main clades corresponding to the African, American and Asian clades *sensu* O'Donnell *et al.* (2000b), plus the African B-clade (Sandoval-Denis *et al.* 2018b, Yilmaz *et al.* 2021) and a fifth, monotypic clade, which formed the sister clade to the joint American and African B clades and which is here termed African C. The latter clade included two strains showing a clear genealogical and morphological separation from their closest phylogenetic relatives; both came from an unknown tree species in South Africa. This clade is here described as the novel species *F. echinatum*. Another fully supported novel monophyletic group was found within the main African clade, related to but distinct from *F. brevicatenulatum* and *F. pseudonygamai*. This novel group, represented by isolates of South African origin isolated from *Prunus spinosa* and from the South African indigenous species *Aloidendron dichotomum*, is here recognised as the novel species *F. prieskaense*.

**Fig. 12:**
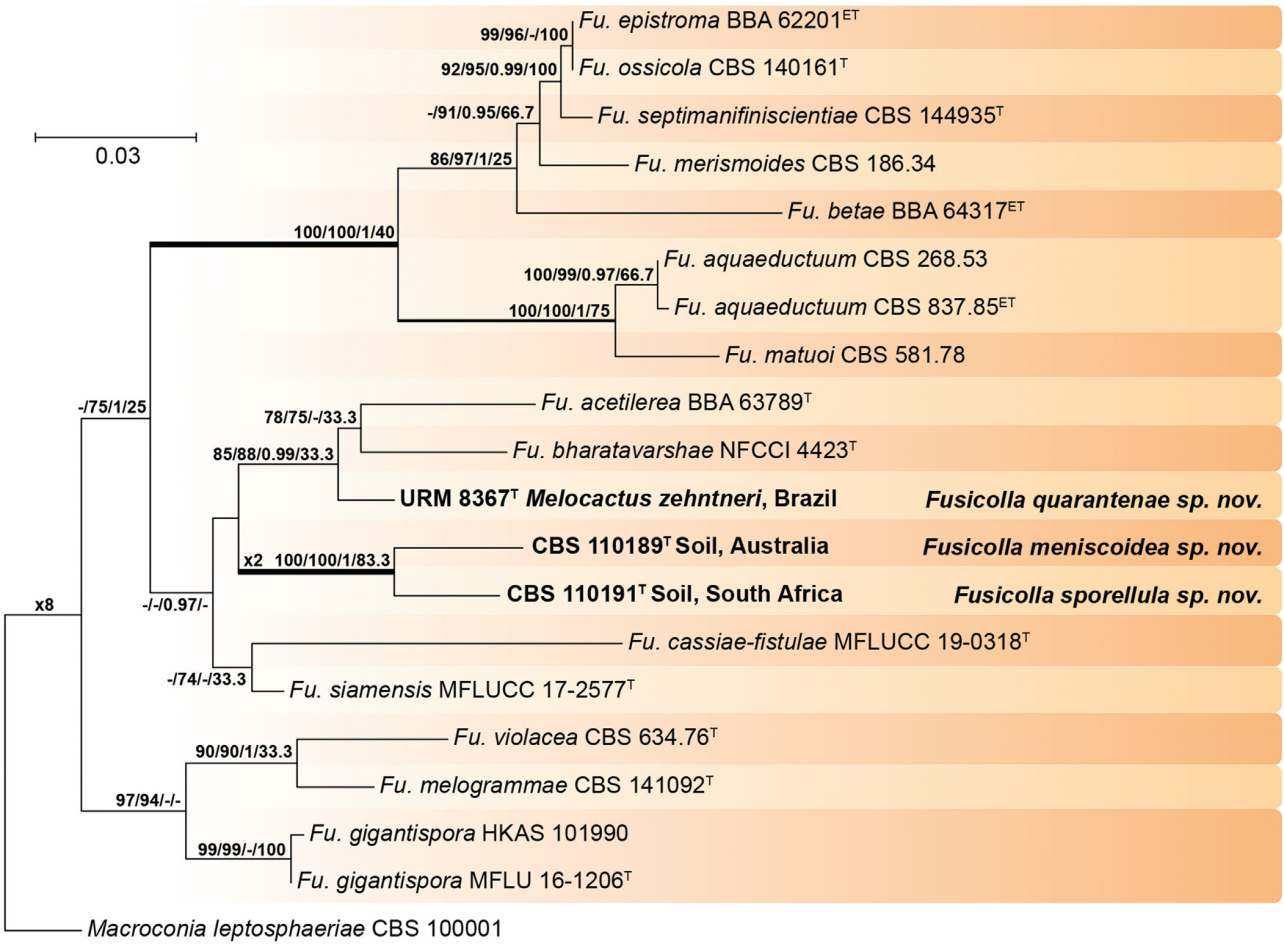
Maximum-Likelihood (IQ-TREE-ML) consensus tree inferred from the combined *acl1*, ITS, LSU, *rpb2*, *tef1*, and *tub2* sequence alignment of members of the genus *Fusicolla*. Numbers at the branches indicate support values (RAxML-BS / UFboot2-BS / BI-PP) above 70 % / 0.95 with thickened branches indicating full support (RAxML-BS / UFboot2-BS = 100 %; BI-PP = 1). Novel taxa are indicated in **bold**. The scale bar indicates expected changes per site. The tree is rooted to *Macroconia leptosphaeriae* CBS 100001. Ex-epitype and ex-type strains are indicated with ET, and T, respectively.

Fusicolla *phylogeny*: The alignment consisted of partial *acl1*, ITS, LSU, *rpb2*, *tef1*, and *tub2* sequences from 20 type or reference strains, representing 17 species of *Fusicolla* (*Fu.*) plus one outgroup taxon (*Macroconia leptosphaeriae* CBS 100001). The analysis confidently resolved 11 ingroup taxa (Fig. 12), including three novel monotypic lineages, represented by strains URM 8367, CBS 110189, and CBS 110191, described here as the new species *Fu. quarantenae*, *Fu. meniscoidea* and *Fu. sporellula*. Due to a partial lack of sequence data, six species could not be clearly resolved. *Fusicolla cassiae-fistulae* and *Fu. siamensis* did not receive statistical support in the combined analysis but are well-resolved using nrDNA sequence data (data not shown). *Fusicolla acetilerea* and *Fu. bharatavarshae*, while well-delimited in the individual ITS, LSU and *rpb2* analyses (data not shown), were ill-supported in the 6-marker combined analysis. Similarly, *Fu. epistroma* and *Fu. ossicola* were not differentiated in either the multimarker analysis, or in the individual *rpb2* analysis. The lack of sequences available to allow comparison with *Fu. epistroma*, for which only LSU and *rpb2* sequences are available, prevented further analysis, as did a similar problem with *Fu. bharatavarshae*, for which only nrDNA and *rpb2* are available.

**Fig. 13:**
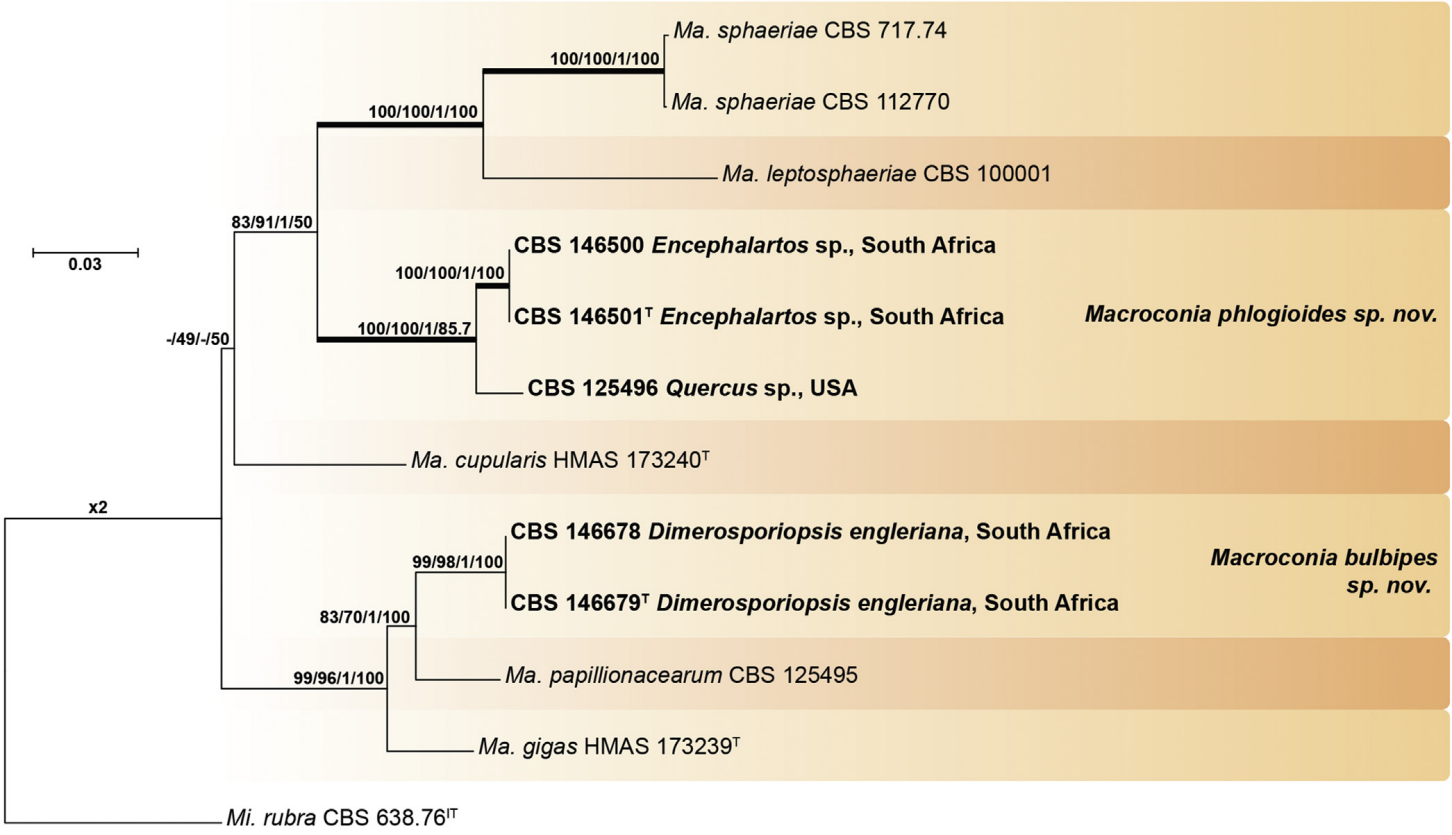
Maximum-Likelihood (ML) consensus tree inferred from the combined *acl1*, *CaM*, ITS, LSU, *rpb1*, *rpb2*, and *tub2* sequence alignment of members of the genus *Macroconia*. Numbers at the branches indicate support values (RAxML-BS / UFboot2-BS / BI-PP / gCF) above 70 % / 0.95 with thickened branches indicating full support (RAxML-BS / UFboot2-BS = 100 %; BI-PP = 1). Novel taxa are indicated in **bold**. The scale bar indicates expected changes per site. The tree is rooted to *Microcera rubra* CBS 638.76. Ex-type and ex-isotype strains are indicated with T, and IT, respectively.

Macroconia *phylogeny*: The analysis consisted of partial *acl1*, *CaM*, ITS, LSU, *rpb1*, *rpb2*, and *tub2* sequences from 12 strains representing seven lineages of *Macroconia* (*Ma*.) plus one outgroup taxon (*Microcera rubra* CBS 638.76) (Fig. 13). Four out of the five *Macroconia* spp. previously known from culture, *Ma. gigas*, *Ma. leptosphaeriae*, *Ma. papilionacearum*, and *Ma. sphaeriae*, resolved as highly to fully-supported lineages. The poorly resolved position of the ex-type isolate of *Ma. cupularis* (HMAS 173240) should be interpreted in light of the fact that only nrDNA sequences were available for analysis. However, separate ITS and LSU comparisons demonstrated it as distinct (data not shown). Two distinct and highly supported novel lineages of South African origin were determined and are described here as the novel species, *Ma. bulbipes* and *Ma. phlogioides*.

**Fig. 14:**
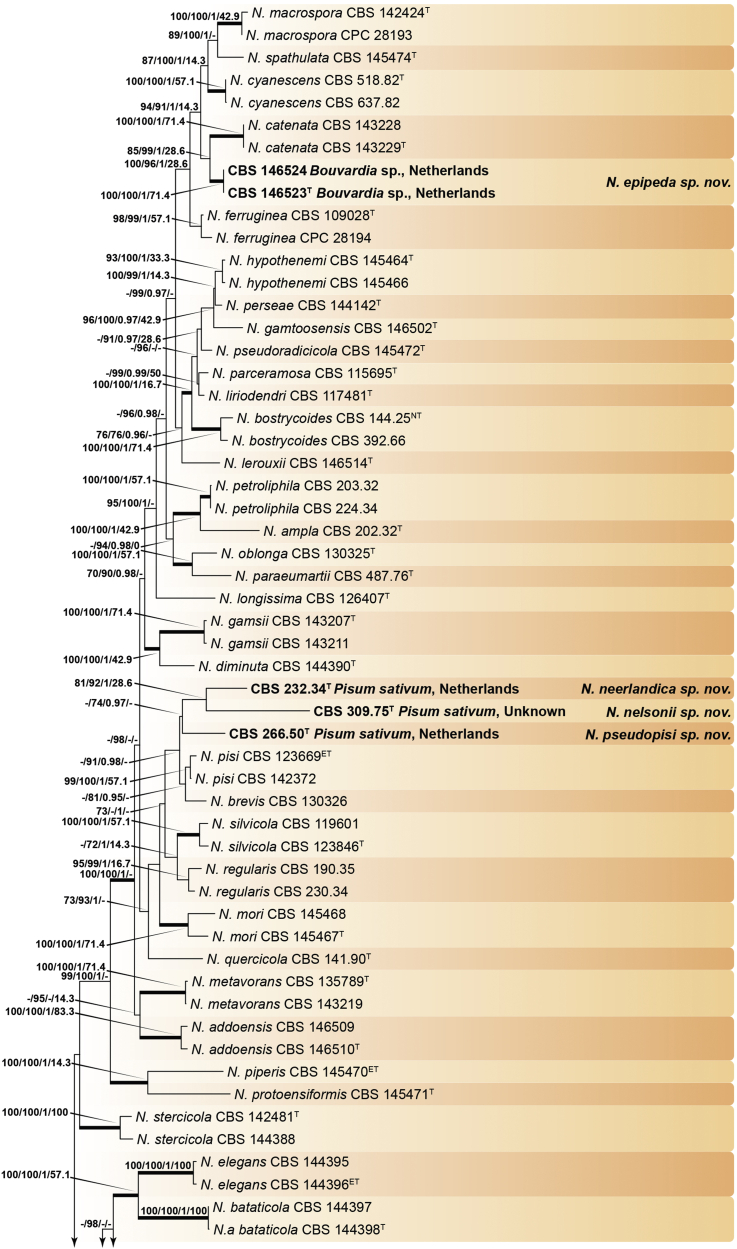

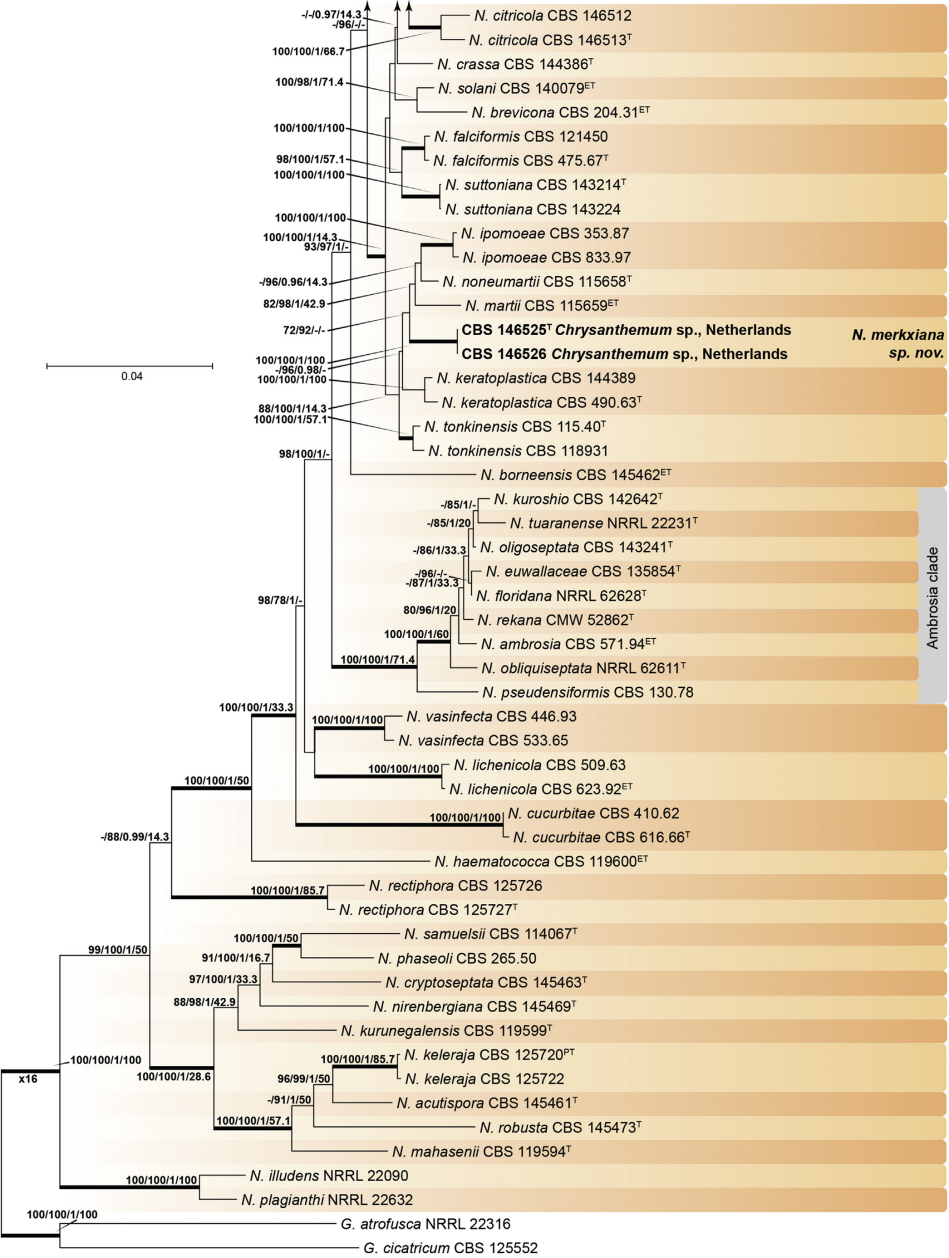
Maximum-Likelihood (IQ-TREE-ML) consensus tree inferred from the combined *acl1*, *CaM*, ITS, LSU, *rpb1*, *rpb2*, and *tef1* sequence alignment of members of the genus *Neocosmospora*. Numbers at the branches indicate support values (RAxML-BS / UFboot2-BS / I-PP) above 70 % / 0.95 with thickened branches indicating full support (RAxML-BS / UFboot2-BS = 100 %; BI-PP = 1). Novel taxa are indicated in **bold**. The scale bar indicates expected changes per site. The tree is rooted to *Geejayessia atrofusca* NRRL 22316 and *G. cicatricum* CBS 125552. Ex-epitype, ex-neotype, ex-paratype and ex-type strains are indicated with ET, NT, PT, and T, respectively.

Neocosmospora *phylogeny*: The alignment consisted of partial *acl1*, *CaM*, ITS, LSU, *rpb1*, *rpb2*, and *tef1* sequences of 107 ex-type and reference strains, including two outgroup taxa (*Geejayessia atrofusca* NRRL 22316 and *G. cicatricum* CBS 125552). The analysis resolved 76 terminal clades, of which 71 correspond to known species of *Neocosmospora* (Fig. 14). Seventy of these clades resolved with high support from two or more independent algorithms (RAxML, IQ-TREE-ML, and BI). The position of the ex-type of *N. crassa* (CBS 144386) is poorly resolved and only partially supported by BI. Similarly, except for the types of *N. ambrosia* (CBS 571.94), *N. obliquiseptata* (NRRL 62611), *N. rekana* (CMW 52862), and the reference strain of *N. pseudensiformis* (CBS 130.78), the position of most members of the well-delimited Ambrosia-clade of *Neocosmospora* were only partially supported by the individual analyses (only BI in *N. kuroshio*, *N. oligoseptata*, and *N. tuaranensis*, and only IQ-TREE-ML-BS for *N. euwallaceae* and *N. floridana*). All these lineages were represented by single isolates in these analyses. Of the five unnamed phylogenetic clades, one corresponded to a species previously known from phylogenetic analyses (FSSC 41, Cardoso 2015), for which a Latin binomial is lacking; this species is here formally described as *N. merkxiana*. The four additional novel lineages discovered here are proposed as the novel species *N. neerlandica*, *N. nelsonii*, *N. pseudopisi*, and *N. epipeda*.

**Fig. 15:**
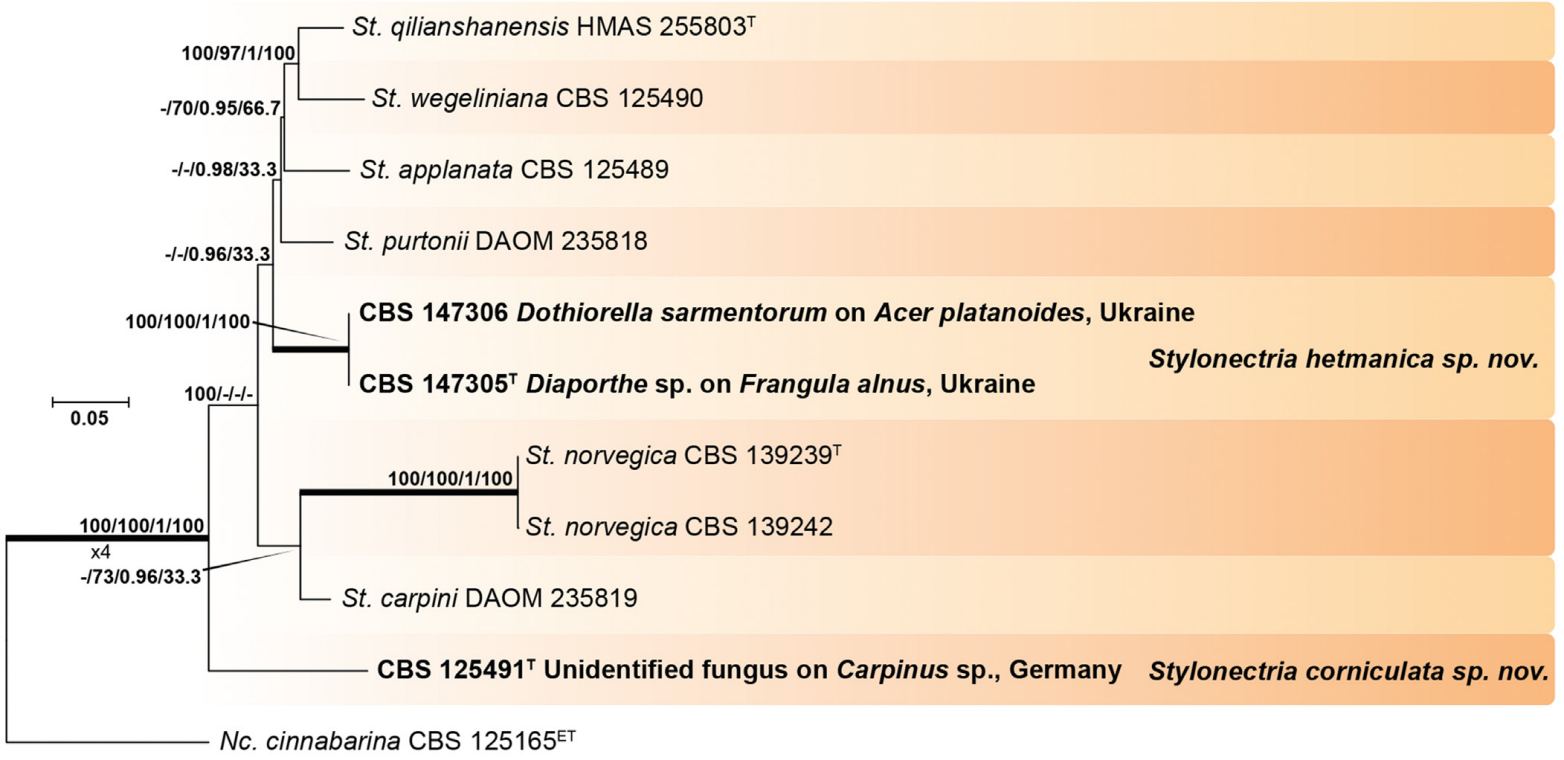
Maximum-Likelihood (IQ-TREE-ML) consensus tree inferred from the combined *acl1*, ITS, and *rpb2* sequence alignment of members of the genus *Stylonectria*. Numbers at the branches indicate support values (RAxML-BS / UFboot2-BS / BI-PP) above 70 % / 0.95 with thickened branches indicating full support (RAxML-BS / UFboot2-BS = 100 %; BI-PP = 1). Novel taxa are indicated in **bold**. The scale bar indicates expected changes per site. The tree is rooted to *Macroconia leptosphaeriae* CBS 100001. Ex-epitype and ex-type strains are indicated with ET and T, respectively.

Stylonectria *phylogeny*: The alignment consisted of partial *acl1*, ITS and *rpb2* sequences of 11 strains, including the outgroup (*Nectria cinnabarina* CBS 125165). The analyses (Fig. 15) identified eight species-level clades, of which six represented previously known species of the genus: *St. applanata*, *St. carpini*, *St. norvegica*, *St. purtonii*, *St. qilianshanensis*, and *St. wegeliniana.* One strain, CBS 125491, isolated from an unknown ascomycetous host, corresponded to a previously known unnamed and fully supported monophyletic lineage, which is formally described here as *St. corniculata*. In addition, a fully supported clade formed by two strains, CBS 147305 from *Diaporthe* sp. and CBS 147306 from *Dothiorella sarmentorum*, is here recognised as the novel species *St. hetmanica*.

### Taxonomy

**Fig. 16:**
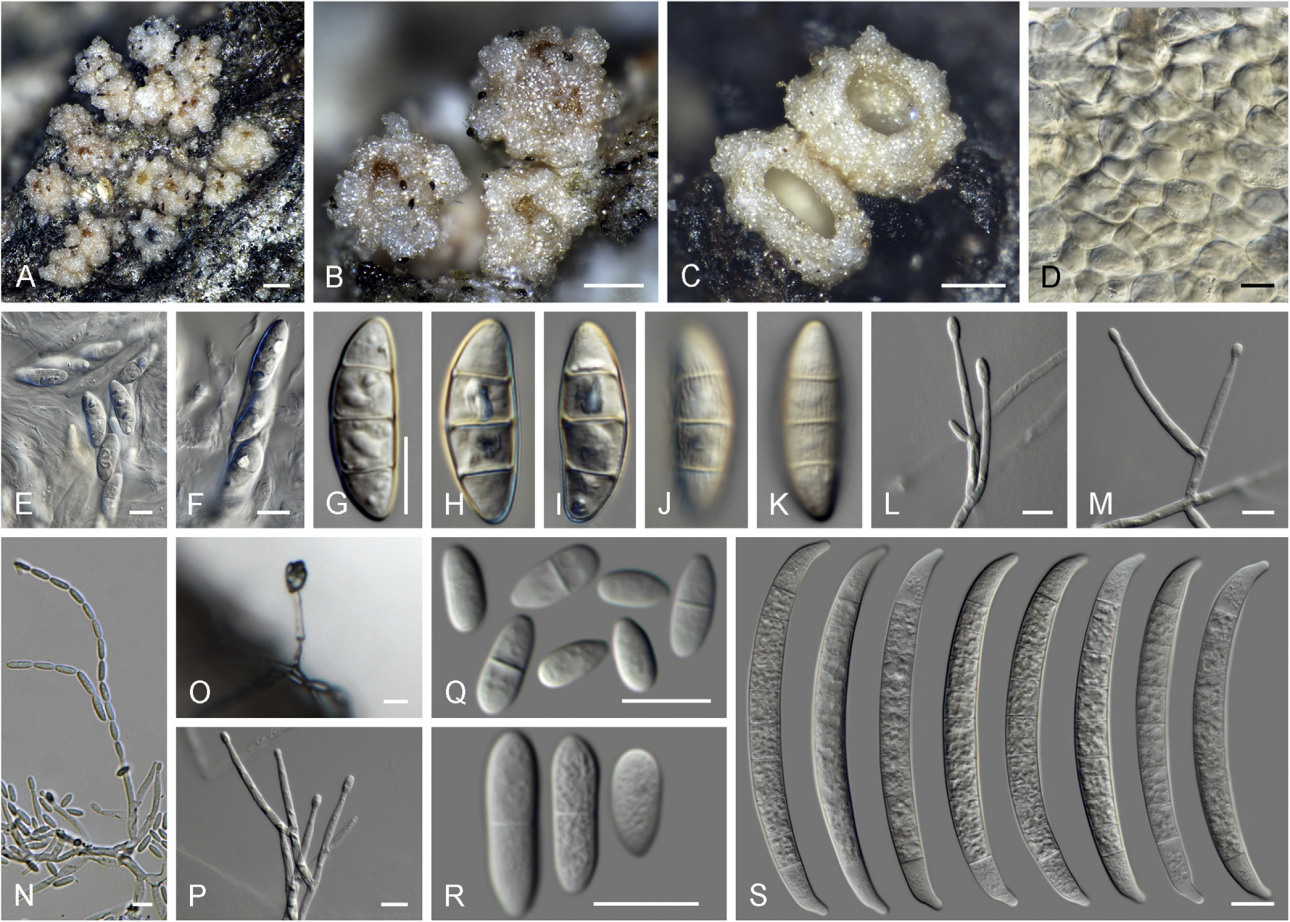
*Albonectria* spp. **A–C.** Ascomata on natural substrate. **D.** Surface view of perithecial wall in 2 % KOH. **E–K.** Asci and ascospores (J, K. Surface view). **L–P.** Conidiophores and conidiogenous cells. **Q, R.** Microconidia. **S.** Macroconidia. A, C–F, H–J. *Albonectria rigidiuscula* (BPI 553050). B, G, K. *Albonectria rigidiuscula* (BPI 1104484). L, M, P–S. *Albonectria rigidiuscula* (CBS 122570). N, O. *Albonectria rigidiuscula* (CBS 133.25). Scale bars: A–C = 100 μm; all others = 10 μm (G applies to H–K).

***Albonectria*** Rossman & Samuels, Stud. Mycol. 42: 105. 1999. Figs 8, 16.

*Type species*: *Albonectria rigidiuscula* (Berk. & Broome) Rossman & Samuels, Stud. Mycol. 42: 105. 1999.

(See *F. colorans* in List section for synonyms)  

*Ascomata* perithecial, solitary or gregarious, superficial on a sparse to well-developed, pseudoparenchymatous stroma, globose to subglobose to ellipsoidal or ovoid to obovoid, not collapsing or laterally pinched when dry, off-white to pale yellow to pale ochraceous, not changing in KOH, strongly tuberculate and thick-walled, with or without a small, pointed papilla, lacking hairs or appendages. *Ascomatal wall* of three regions: outer region of thick-walled, *textura angularis* to *textura globulosa*; middle region of elongate thick-walled cells; inner region with thin-walled, hyaline elongated cells. *Asci* narrowly to broadly clavate or ellipsoidal, 4–8-spored, ascospores obliquely uniseriate or biseriate. *Ascospores* ellipsoidal to long-ellipsoidal or fusoid to long-fusoid, 3- to multiseptate, hyaline to yellow-brown, smooth to striate, not to slightly constricted at the septum. *Conidiophores* mononematous (aerial conidiophores) or grouped on sporodochia; *aerial conidiophores* unbranched or irregularly branched, bearing terminal or lateral phialides, often reduced to single phialides; *conidiogenouhs cells* monophialidic, cylindrical to subcylindrical, smooth- and thin-walled, with periclinal thickening inconspicuous or absent, producing arial micro- and macroconidia. *Microconidia* hyaline, thin-walled, 0- or 1-septate, ovoid to obovoid, with or without a flattened basal papilla, borne in dry chains or small slimy heads. *Macroconidia* falcate, multiseptate, thick-walled, with a blunt to hooked apical cell and well-developed foot-shaped basal cell or distinctly beaked at both ends. *Sporodochia* cream to yellow; *sporodochial conidiophores* verticillately branched and densely packed, consisting of short, smooth- and thin-walled stipes bearing apical whorls of 2–4 monophialides; *sporodochial conidiogenous cells* monophialidic, cylindrical to subulate, smooth- and thin-walled, with reduced or flared collarette. *Sporodochial macroconidia* formed in off-white or creamy slimy masses, falcate, 5–9-septate, thick-walled, gently curved to straight, with a blunt to hooked apical cell and distinct well-developed foot-shaped basal cell. *Chlamydospores* absent.

[Description adapted from Rossman *et al.* (1999), Booth (1971) and Lombard *et al.* (2015)].  

*Diagnostic features*: Off-white to pale yellow to pale ochraceous perithecia producing narrowly or broadly clavate to ellipsoidal asci containing (long) ellipsoidal to fusoid, 3- to multiseptate ascospores; fusarioid asexual morph characterised by monophialides producing distinctly long, robust, slightly curved to straight multiseptate macroconidia and dry chains or small slimy heads of ovoid microconidia. Chlamydospores absent.  

**Fig. 17:**
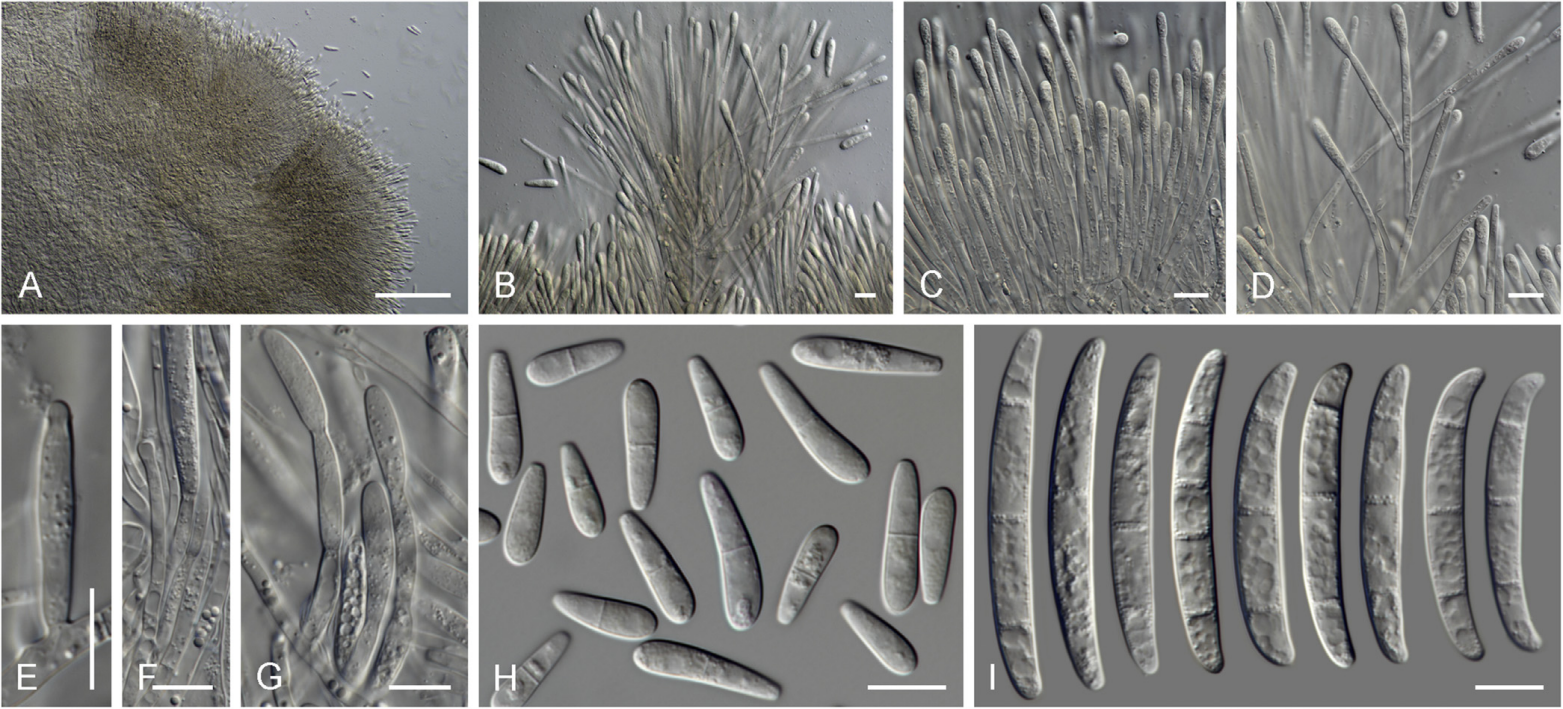
*Atractium* spp. **A, B.** Synnemata. **C–G.** Conidiophores and conidiogenous cells. **H.** Microconidia. **I.** Macroconidia. A–D, H. *Atractium stilbaster* (CBS 410.67). E–G, I. *Atractium crassum* (CBS 180.31). Scale bars: A = 100 μm; all others = 10 μm.

***Atractium*** Link, Mag. Ges. Naturf. Freunde Berlin 3: 10. 1809 (Fries, Syst. Mycol. 1: XLI. 1821, *nom. sanct.*). Figs 8, 17.

*Type species*: *Atractium stilbaster* Link, Mag. Ges. Naturf. Freunde Berlin 3: 10. 1809.  

*Ascomata* unknown. *Conidiophores* aggregated into sporodochia or synnemata, non-stromatic; *synnemata* determinate, pale brown, composed of a stipe of parallel hyphae and a divergent capitulum of conidiophores giving rise to a slimy conidial mass; conidiophore branching once or twice monochasial, 2-level verticillate, monoverticillate or irregularly biverticillate. *Conidiogenous cells* monophialidic, hyaline, subulate with conspicuous periclinal thickening, producing micro- and macroconidia. *Microconidia* hyaline, thin-walled, 0- or 1-septate, ellipsoidal, allantoid, broadly lunate to reniform, straight or slightly curved, tapering towards both apices with rounded base. *Macroconidia* 3–5-septate, falcate, gently curved, with a rounded to blunt apical cell, and obtuse, non foot-shaped basal cell, forming yellow to orange masses.

[Description adapted from Gräfenhan *et al.* (2011)].  

*Diagnostic features*: Synnematous asexual morph characterised by fusarioidal macroconidia lacking foot-shaped basal cells.  

**Fig. 18:**
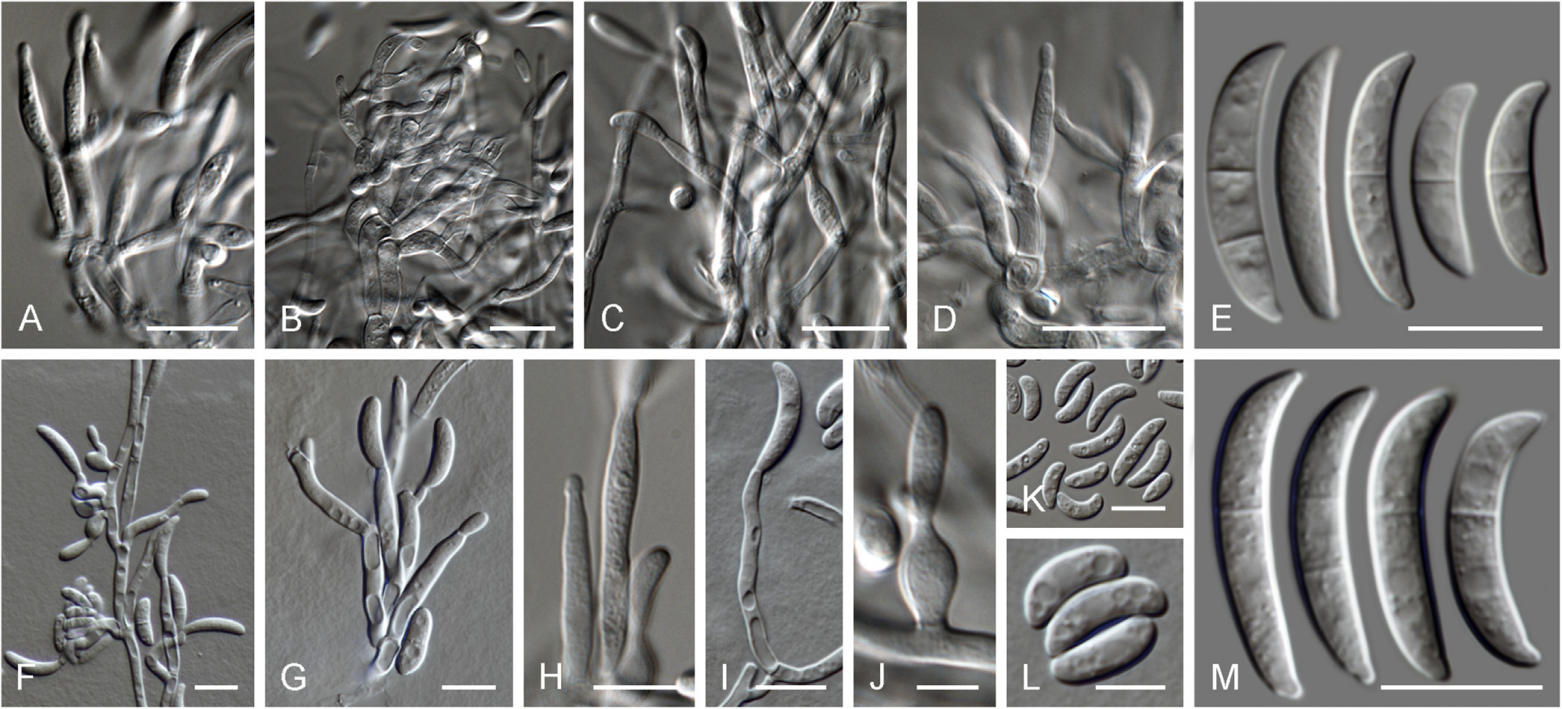
*Bisifusarium* spp. **A–D, F–J.** Conidiophores and conidiogenous cells. **K, L.** Microconidia. **E, M.** Macroconidia. A–E. *Bisifusarium dimerum* (CBS 108944). F–M. *Bisifusarium delphinoides* (CBS 120718). Scale bars: H, J = 5 μm; all others = 10 μm.

***Bisifusarium*** L. Lombard *et al.*, Stud. Mycol. 80: 223. 2015. Figs 8, 18.

*Type species*: *Bisifusarium dimerum* (Penz.) L. Lombard & Crous, Stud. Mycol. 80: 225. 2015.

(See *F. dimerum* in List section for synonyms)  

*Ascomata* unknown. *Conidiophores* mononematous (aerial conidiophores) or grouped on sporodochia; *aerial conidiophores* simple, unbranched or irregularly branched, mostly reduced to terminal or single lateral conidiogenous cells. *Conidiogenous cells* often formed as (i) lateral phialidic pegs arising from superficial or submerged intercalary hyphal cells or, (ii) cylindrical and slightly tapering towards apex or ampulliform, smooth- and thin-walled monophialides, rarely polyphialides, with inconspicuous or absent periclinal thickening, solitary or aggregated to represent a poorly developed pionnotal sporodochial-like structure, producing micro- and macroconidia. *Microconidia* hyaline, thin-walled, 0- or 1-septate, ellipsoidal, allantoid, broadly lunate to reniform, straight or curved, tapering towards both ends. *Macroconidia* falcate, (0–)1–2(–3)-septate, thick-walled, curved to lunate, with a blunt to hooked apical cell and obtuse to poorly developed, foot-shaped basal cell, typically formed on sporodochia. *Sporodochia* pale yellow to orange; *sporodochial conidiophores* verticillately branched and densely packed, consisting of short, smooth- and thin-walled stipes bearing an apical whorl of 2–3 monophialides; *sporodochial conidiogenous cells* monophialidic, cylindrical to subulate, smooth- and thin-walled, with reduced or flared collarette. *Chlamydospores*, if present, globose to subglobose to ellipsoidal, solitary or in chains, sometimes aggregated in sclerotia.

[Description adapted from Schroers *et al.* (2009) and Lombard *et al.* (2015)].  

*Diagnostic features*: Fusarioid asexual morph characterised by lateral phialidic pegs arising from superficial or submerged intercalary hyphal cells or solitarily formed monophialides producing microconidia; distinctly short (< 30 μm long), curved to lunate, (0–)1–2(–3)-septate macroconidia typically formed on sporodochia on plant tissue such as carnation leaf pieces.  

***Corinectria*** C. González & P. Chaverri, Mycol. Progr. 16: 1021. 2017. Fig. 8.  

*Type species*: *Corinectria fuckeliana* (C. Booth) C. González & P. Chaverri, Mycol. Progr. 16: 1023. 2017.

*Basionym*: *Nectria fuckeliana* C. Booth, Mycol. Pap. 73: 56. 1959.

*Synonym*: *Neonectria fuckeliana* (C. Booth) Castl. & Rossman, Canad. J. Bot. 84: 1428. 2006.  

*Ascomata* perithecial, gregarious, seated on an erumpent stroma, superficial, globose to subglobose, orange, red to dark red darkening around ostiolar region, turning black in KOH, pigment dissolving in lactic acid, not collapsing when dry, slightly papillate to papillate, smooth-walled, lacking hairs or appendages. *Ascomatal wall* of 2–3 regions: outer region of thick-walled, pigmented cells forming a *textura epidermoidea*; middle and inner regions of globose to elongate, hyaline, thin-walled cells, becoming thinner toward the centrum. *Asci* cylindrical, 8-spored, with an apical ring, uniseriate. *Ascospores* ellipsoidal to fusoid, 1-septate, hyaline, smooth. *Conidiophores* mononematous, hyaline, septate, unbranched or sparsely branched, terminating in 1–2 phialides or reduced to lateral phialides. *Conidiogenous cells* monophialidic, cylindrical, tapering towards the apex, with inconspicuous periclinal thickening and collarettes. *Sporodochia* not formed. *Microconidia* ellipsoidal to obovoid, hyaline, aseptate, sometimes forming false heads on phialides. *Macroconidia* cylindrical, mostly straight, (3–)5–7-septate, with rounded ends. *Chlamydospores* unknown.

[Description adapted from González & Chaverri (2017)].  

*Diagnostic features*: Orange to dark red, smooth-walled perithecia with papilla producing cylindrical asci bearing ellipsoidal to fusoid, 1-septate ascospores and cylindrocarpon-like asexual morph characterised by (3–)5–7-septate macroconidia.  

***Cosmospora*** Rabenh., Hedwigia 2: 59. 1862. Fig. 8.

*Synonyms*: *Crysogluten* Briosi & Farneti, Atti Ist. Bot. Univ. Lab. Crittog. Pavia 8: 117. 1904.

*Botryocrea* Petr., Sydowia 3: 140. 1949.  

*Type species*: *Cosmospora coccinea* Rabenh., Hedwigia 2: 59. 1862 [non *Nectria coccinea* (Pers.) Fr. 1849].

*Synonyms*: *Nectria cosmariospora* Ces. & De Not., Comment. Soc. Crittog. Ital. 1: 195. 1863.

*Dialonectria cosmariospora* (Ces. & De Not.) Cooke, Grevillea 12: 110. 1884.

*Cucurbitaria cosmariospora* (Ces. & De Not.) Kuntze, Revis. Gen. Pl. 3: 461. 1898.

*Dialonectria cosmariospora* (Ces. & De Not.) Z. Moravec, Ceská Mykol. 8: 92. 1954, an isonym, Art. 6.3, Note 2.

*Verticillium olivaceum* W. Gams, Cephalosporium-artige Schimmelpilze: 129. 1971.  

*Ascomata* perithecial, solitary or gregarious, with inconspicuous or absent stroma, obpyriform with an acute or papillate apex, orange red or bright red, turning dark red in KOH, smooth walled. *Asci* narrowly clavate to cylindrical, with an apical ring, 8-spored. *Ascospores* initially hyaline, becoming yellow brown to reddish brown, 1-septate, becoming tuberculate when mature. *Conidiophores* acremonium-like, consisting of lateral phialides on somatic hyphae, or with one or two levels of monochasial branching, or verticillate, hyaline. *Conidiogenous cells* monophialidic, cylindrical to subulate to subclavate, hyaline. *Microconidia* ellipsoidal, oblong or clavate or slightly allantoid, aseptate, hyaline, forming slimy heads. *Macroconidia* absent or rare, subcylindrical, curved, slightly narrowing towards each end, apical cell often slightly hooked with a more or less pointed apex, basal cell obtuse to poorly developed, foot-shaped, 3–5-septate, hyaline.

[Description adapted from Rossman *et al.* (1999) and Gräfenhan *et al.* (2011)].  

*Diagnostic features*: Orange-red to bright red perithecia with an acute or papillate apex producing cylindrical to narrowly clavate asci, yellow brown to reddish brown, 1-septate, tuberculate ascospores and acremonium-like asexual morph.  

**Fig. 19:**
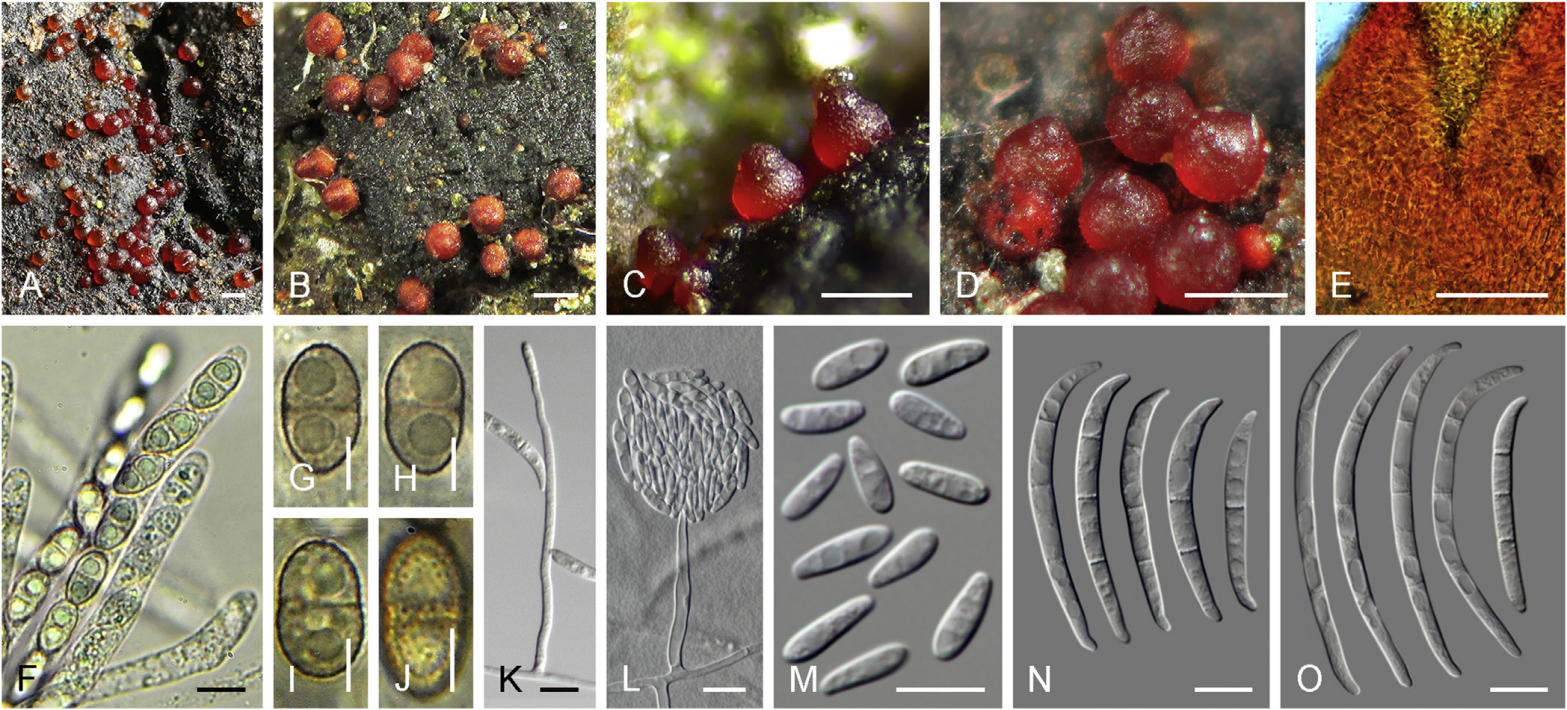
*Cosmosporella* spp. **A–D.** Ascomata on natural substrate. **E.** Surface view of perithecial wall. **F.** Asci. **G–J.** Ascospores. **K, L.** Conidiophores. **M.** Microconidia. **N, O.** Macroconidia. A–J. “*Cosmospora” flavoviridis* (photos P. Mlčoch). K–N. “*Cosmospora” flavoviridis* (CBS 124353). O. *Cosmosporella cavisperma* (CBS 172.31). Scale bars: A–D = 300 μm; E = 50 μm; G–J = 5 μm; all others = 10 μm.

***Cosmosporella*** S.K. Huang *et al.*, Cryptog. Mycol. 39: 179. 2018. Figs 8, 19.

*Type species*: *Cosmosporella olivacea* S.K. Huang *et al.*, Cryptog. Mycol. 39: 181. 2018.  

*Ascomata* perithecial, solitary to gregarious, superficial, on immersed to erumpent stroma, ovoid, globose to obpyriform, collapsing laterally when dry, orange red, red to pale yellow, not reacting in KOH, with a central ostiole, with hyaline periphyses. *Ascomatal wall* membranous, composed of orange to hyaline cells of *textura angularis*, with septate paraphyses. *Asci* cylindrical to slightly clavate, apically rounded, with evanescent wall, pedicel combined with paraphyses, 8-spored, unitunicate. *Ascospores* hyaline to pale brown, ellipsoidal to ovoid, 0- or 1-septate. *Conidiophores* acremonium-like, mononematous, hyaline, septate, consisting of lateral phialides on somatic hyphae, or with one or two levels of monochasial branching, or irregularly branched. *Conidiogenous cells* monophialidic, cylindrical, producing micro- and macroconidia. *Microconidia* ellipsoidal to obovoid, 0- or 1-septate, hyaline, forming a false head on phialides. *Macroconidia* falcate, almost straight to curved, 1–3-septate, with a blunt to hooked apical cell and poorly to well-developed foot-shaped basal cell. *Chlamydospores* unknown.

[Description adapted from Huang *et al.* (2018)].  

*Diagnostic features*: Pale yellow to orange-red perithecia lacking a papilla producing cylindrical to narrowly clavate asci, pale brown, 1-septate ascospores and fusarioid asexual morph characterised by overly long, 1–3-septate macroconidia.  

***Cosmosporella cavisperma*** (Corda) Sand.-Den., L. Lombard & Crous, ***comb. nov*.** MycoBank MB 838659.

*Basionym*: *Fusarium cavispermum* Corda, Icon. Fung. 1: 3. 1837.

*Synonyms*: *Fusarium aquaeductuum* var. *cavispermum* (Corda) Raillo, *Fungi of the Genus Fusarium*: 280. 1950.

*Fusarium oxydendri* Ellis & Everh., Bull. Torrey Bot. Club 24: 477. 1897.

*Fusarium cavispermum* var. *minus* Wollenw., Fusaria Autogr. Delin. 3: 848. 1930.  

*Lectotypus*: **Czech Republic**, near Carlsstein, on pine resin, A.K.J. Corda, Icon. Fung. 1: tab. I, fig. 58 (MBT 10001322 *hic designatus*). **Epitype** of *Fusarium cavispermum* (CBS 172.31, MBT 10000645 *hic designatus,* a metabolically inactive culture). **Norway**, from *Pinus sylvestris*, 1929, H.W. Wollenweber, culture ex-epitype CBS 172.31 = NRRL 13996.  

*Notes*: The genus *Cosmosporella* was erected by Huang *et al.* (2018) to accommodate *Cm. olivacea* and the superfluous taxon *Cm. obscura*, shown to cluster within a subset of taxa pertaining to *Cosmospora s. lat*. (Rossman *et al.* 1999), former members of the *Nectria episphaeria* group *sensu* Booth (1959) and *Nectria* subgenus *Dialonectria* (Samuels *et al.* 1991) characterised by cosmospora-like sexual morphs and fusarioid asexual morphs. More recently, this monophyletic clade had been ascribed to the *Fusarium cavispermum* species complex (O'Donnell *et al.* 2013) and, separated from any of the polyphyletic taxa formerly classified in *Fusarium* section *Eupionnotes* (O'Donnell 1993, Schroers *et al.* 2009, Gräfenhan *et al.* 2011). “*Fusarium” melanochlorum*, its purposed sexual morph “*Nectria*” *flavoviridis* (Gerlach & Nirenberg 1982), and “*Fusarium*” *cavispermum* have also been resolved as members of this clade (Gräfenhan *et al.* 2011, O'Donnell *et al.* 2013, Huang *et al.* 2018, and Fig. 7 in this paper). Here, the new combination *Cm. cavisperma* is proposed, lectotypified, and an epitype is designated to stabilise the application of the name based on material studied by Wollenweber [number 849 in Wollenweber (1916–1935) and Gerlach & Nirenberg (1982)]. The suggested conspecificity of “*F”. melanochlorum* and “*N*”. *flavoviridis*, however, is questioned given the large phylogenetic distance between the currently available strains. Fresh isolations and a thorough phylogenetic revision of the entire group including additional *Cosmospora s. lat.* taxa having fusarioid asexual morphs are necessary.  

**Fig. 20:**
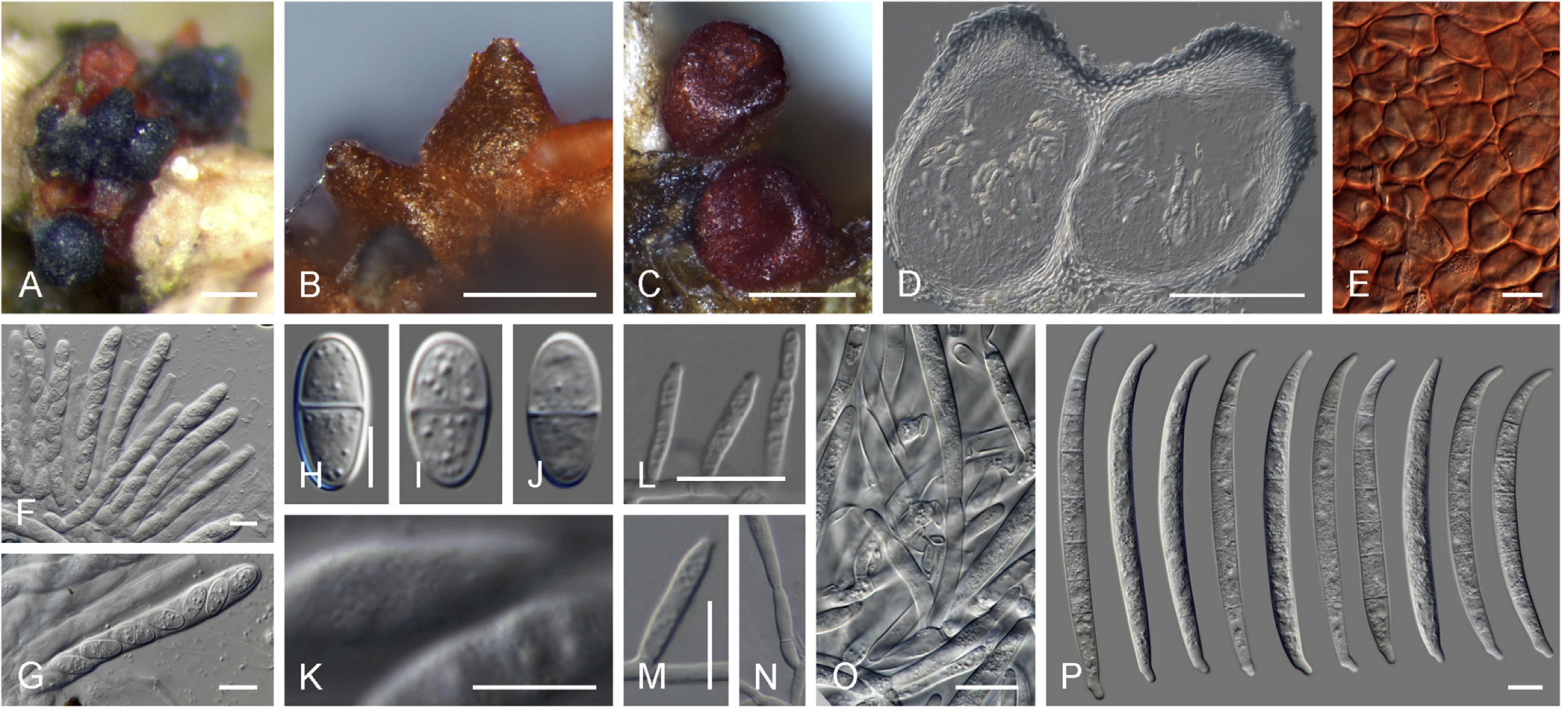
*Cyanonectria* spp. **A–C.** Ascomata on natural substrate. **D.** Longitudinal section through perithecium in Shears. **E.** Surface view of perithecial wall in 2 % KOH. **F, G.** Asci. **H–K.** Ascospores (K. Surface view). **L–O.** Conidiogenous cells. **P.** Macroconidia. A–C, E–J. *Cyanonectria buxi* (CBS H-20380). D, K. *Cyanonectria buxi* (CBS H-20379). L–N. *Cyanonectria buxi* (CBS 130.97). O, P. *Cyanonectria buxi* (CBS 125551). [A, D, L. adapted from Schroers *et al.* (2011).] Scale bars: A–D = 100 μm; H–K = 5 μm (H applies to I and J); all others = 10 μm.

***Cyanonectria*** Samuels & Chaverri, Mycol. Progr. 8: 56. 2009. Figs 8, 20.

*Type species*: *Cyanonectria cyanostoma* (Sacc. & Flageolet) Samuels & Chaverri, Mycol. Progr. 8: 56. 2009.

*Basionym*: *Nectria cyanostoma* Sacc. & Flageolet, Rendiconti Congr. Bot. Palermo 1902: 53. 1902.

*Synonym*: *Fusarium cyanostomum* (Sacc. & Flageolet) O'Donnell & Geiser, Phytopathology 103: 404. 2013.  

*Ascomata* perithecial, gregarious or caespitose, with a reduced or well-developed prosenchymatous stroma, smooth- and thin-walled, ampulliform to obpyriform to pyriform, apex dark bluish purple to bluish black and body less intensely dark bluish or red or reddish brown, turning darker in KOH, pigment dissolving in lactic acid to become red to yellow, non-papillate, lacking hairs or appendages. *Ascomatal wall* consisting of a single region, comprising several layers of morphologically similar cells. *Asci* cylindrical to narrowly clavate, with rounded to flattened thickened apex, with or without refractive ring, 8-spored, ascospores overlapping uniseriate or biseriate above and uniseriate below. *Ascospores* ellipsoidal, 1-septate, not or slightly constricted at septum, pale yellow-brown, smooth-walled or finely verrucose. *Conidiophores* mononematous (aerial conidiophores) or grouped on sporodochia; *aerial conidiophores* unbranched or rarely branched, bearing terminal or lateral phialides, often reduced to single phialides. *Conidiogenous cells* monophialidic, cylindrical to subcylindrical, smooth- and thin-walled, with periclinal thickening inconspicuous or absent. *Sporodochia* white to bluish; *sporodochial conidiophores* verticillately branched and densely packed, consisting of short, smooth- and thin-walled stipes bearing apical whorls of 2–3 monophialides; *sporodochial conidiogenous cells* monophialidic, cylindrical to subulate, smooth- and thin-walled, with reduced or flared collarette. *Macroconidia* formed in off-white or creamy or greyish blue slimy masses, falcate, straight to gently curved, with inequilateral fusoid or hooked apical cell and well-developed, foot-shaped basal cell. *Microconidia* unknown. *Chlamydospores* absent or rarely formed from cells of the macroconidia, subglobose.

[Description adapted from Samuels *et al.* (2009) and Schroers *et al.* (2011)].  

*Diagnostic features*: Bicoloured or dark bluish purple to bluish black perithecia producing cylindrical to narrowly clavate asci containing ellipsoidal, 1-septate ascospores and fusarioid asexual morph characterised by monophialides producing long, narrow, almost straight macroconidia, lacking microconidia and hyphal-borne chlamydospores.  

**Fig. 21:**
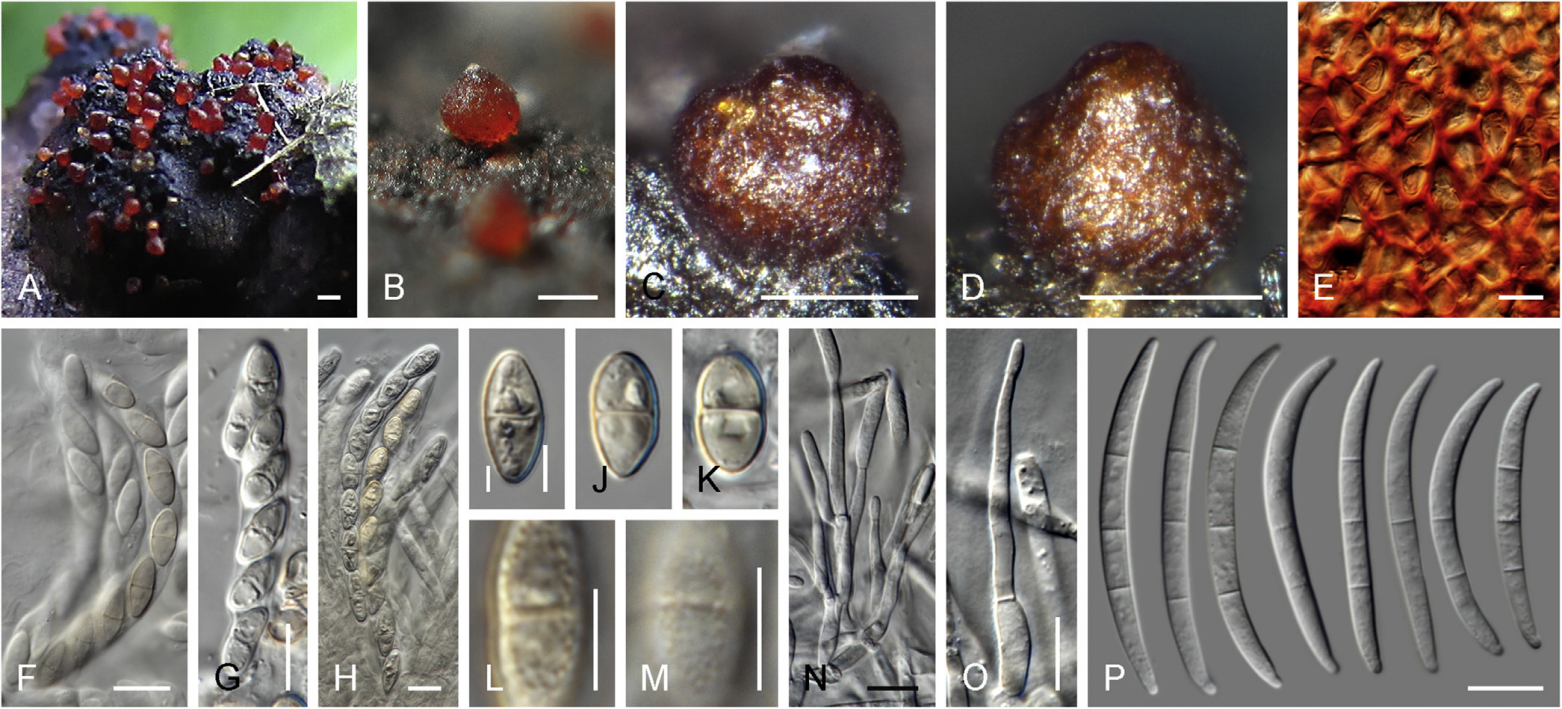
*Dialonectria* spp. **A–D.** Ascomata on natural substrate. **E.** Surface view of perithecial wall in 2 % KOH. **F–H.** Asci. **I–M.** Ascospores (L, M. Surface view). **N, O.** Conidiophores and conidiogenous cells. **P.** Macroconidia. A, B. *Dialonectria episphaeria* (photos P. Mlčoch). C, D, F, M. *Dialonectria episphaeria* (CBS H-19716). E, G, K. *Dialonectria sanguinea* (CBS H-2127). H–J, L. *Dialonectria episphaeria* (CBS H-2662). N–P. *Dialonectria episphaeria* (CBS 125494). Scale bars: A–D = 100 μm; I, L, M = 5 μm (I applies to J and K); all others = 10 μm.

***Dialonectria*** (Sacc.) Cooke, Grevillea 12: 77, 109. 1884. Figs 8, 21.

*Basionym*: *Nectria* subgen. *Dialonectria* Sacc., Syll. Fung. 2: 490. 1883.  

*Type species*: *Dialonectria episphaeria* (Tode) Cooke (as “*episphaerica*”), *Grevillea* 12: 82. 1884.

*Basionym*: *Sphaeria episphaeria* Tode, Fung. mecklenb. sel. 2: 21. 1791.  

*Ascomata* perithecial, solitary or gregarious, with inconspicuous or absent stroma, obpyriform with an acute or round papilla, orange red to carmine red, turning dark red in KOH, smooth-walled. *Asci* narrowly clavate to cylindrical, with an apical ring, 8-spored, uniseriate. *Ascospores* initially hyaline, becoming pale brown, 1-septate, becoming tuberculate when mature. *Conidiophores* mononematous, initially as lateral phialides on somatic hyphae, sometimes verticillate, hyaline. *Conidiogenous cells* monophialidic, subulate to subclavate, hyaline. *Microconidia* ellipsoidal to clavate, aseptate, hyaline, abundant. *Macroconidia* if present subcylindrical, moderately curved, slightly narrowing towards each end, apical cell often slightly hooked with a more or less pointed tip, basal cell obtuse to poorly developed, foot-shaped, predominantly 3–5-septate, hyaline. *Chlamydospores* unknown.

[Description adapted from Rossman *et al.* (1999) and Gräfenhan *et al.* (2011)].  

*Diagnostic features*: Orange-red to carmine-red perithecia with an acute or round papilla producing cylindrical to narrowly clavate asci, pale brown, 1-septate, tuberculate ascospores and asexual morph that rarely produces macroconidia.  

**Fig. 22:**
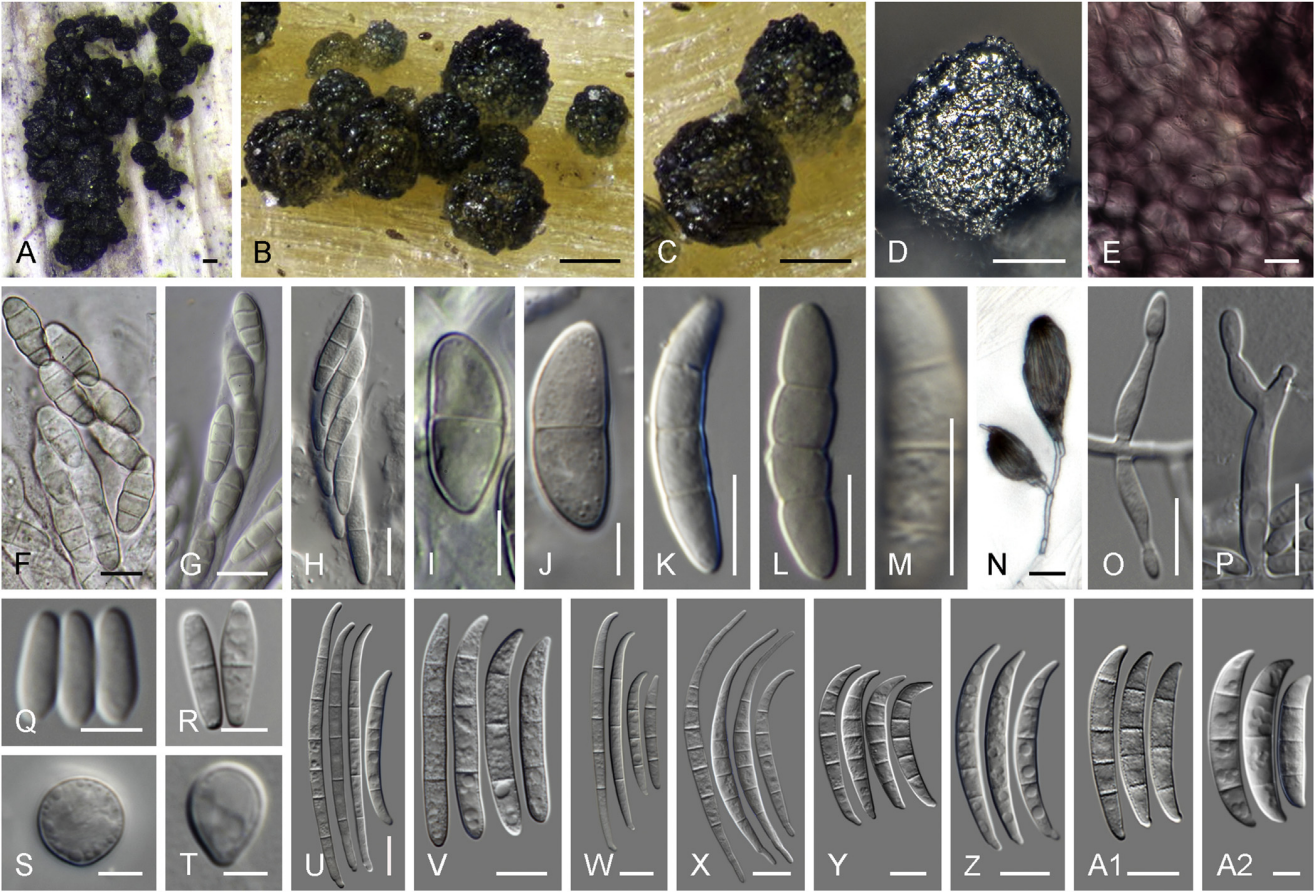
*Fusarium* spp. **A–D.** Ascomata on natural substrate. **E.** Surface view of perithecial wall in 2 % KOH. **F–H.** Asci. **I–M.** Ascospores (M. Surface view). **N–P.** Conidiophores and conidiogenous cells. **Q–T.** Microconidia. **U–A2.** Macroconidia. A. *Fusarium graminearum* (photo P. Cannon). B, C, F. *Fusarium sambucinum* [adapted from Wergen (2018)]. D. *Fusarium* sp. (HPC 2244). E. *Fusarium cf*. *tricinctum* (CBS H-12819). G, I. *Fusarium lateritium* (photo P. Cannon). H, K. *Fusarium equiseti* (CBS H-12817). J. *Fusarium sambucinum* (BPI 632307). L, M. *Fusarium sambucinum* (CBS H-12818). N. *Fusarium avenaceum* (CPC 30660). O, Q. *Fusarium fredkrugerii* (CBS 144209). P, W. *Fusarium prieskaense* (CBS 146498). R. *Fusarium madaense* (CBS 146669). S. *Fusarium globosum* (CBS 428.97). T. *Fusarium echinatum* (CBS 146497). U. *Fusarium avenaceum* (CBS 408.86). V. *Fusarium caeruleum* (CBS 146590). X. *Fusarium longicaudatum* [CBS 123.73, adapted from Xia *et al.* (2019)]. Y. *Fusarium transvaalense* [CBS 144211, adapted from Sandoval-Denis *et al.* (2018b)]. Z. *Fusarium gamsii* (CBS 143610). A1. *Fusarium oxysporum* [CBS 144134, adapted from Lombard et al., 2019b, Lombard et al., 2019a]. A2. *Fusarium convolutans* [CBS 144207, adapted from Sandoval-Denis *et al.* (2018b)]. Scale bars: A–D = 100 μm; I–M, Q–T = 5 μm; all others = 10 μm.

***Fusarium*** Link, Mag. Ges. Naturf. Freunde Berlin 3: 10. 1809. Figs 8, 22.

*Synonyms*: *Fusisporium* Link, Mag. Ges. Naturf. Freunde Berlin 3: 19. 1809.

*Selenosporium* Corda, Icon. Fung. 1: 7. 1837.

*Gibberella* Sacc., Michelia 1: 43. 1877.

*Lisea* Sacc., Michelia 1: 43. 1877.

*Sporotrichella* P. Karst., Meddel. Soc. Fauna Fl. Fenn. 14: 96. 1887.

*Gibberella* subgen. *Lisiella* Cooke & Massee, Grevillea 16: 5. 1887.

*Lisiella* (Cooke & Massee) Sacc., Syll. Fung. 9: 945. 1891.

*Septorella* Allesch., Hedwigia 36: 241. 1897.

*Ustilaginoidella* Essed, Ann. Bot. 25: 351. 1911.

*Rachisia* Linder, Deutsche Essigind. 17: 467. 1913.

*Stagonostroma* Died., Krypt.-Fl. Mark Brandenb. 9: 561. 1914.

*Fusidomus* Grove, J. Bot. 67: 201. 1929.

*Pseudofusarium* Matsush., Microfungi Solomon Isl. Papua-New Guinea: 46. 1971.  

*Type species*: *Fusarium sambucinum* Fuckel, Fungi Rhen. Exs., Fasc. 3, no. 211. 1863, *nom. cons.*

(See List section for synonyms)  

*Ascomata* perithecial, mostly gregarious, non-stromatic or on a thin stroma erumpent through the epidermis, superficial, subglobose to globose to broadly pyriform, not collapsing or laterally pinched when dry, bluish purple to black, turning dark purple in KOH, pigment dissolving in lactic acid, non-papillate, slightly rugose to tuberculate, lacking hairs or appendages. *Ascomatal wall* of two regions: outer region of thick-walled, pigmented cells forming a *textura angularis* or *textura globulosa*; inner region of elongate, hyaline, thin-walled cells, becoming thinner towards the centrum. *Asci* clavate, apex simple, 8-spored often with an apical ring, biseriate to pluriseriate. *Ascospores* ellipsoidal to cylindrical, 1–3-septate, not or slightly constricted at the septa, pale tan, smooth-walled. *Conidiophores* mononematous (aerial conidiophores) or grouped on sporodochia; *aerial conidiophores*, if consistenly formed, unbranched, sympodial or irregularly branched, bearing terminal or lateral phialides, often reduced to single phialides. *Conidiogenous cells* mono- or polyphialidic, subulate to subcylindrical, smooth- and thin-walled, sometimes proliferating percurrently, with periclinal thickening inconspicuous or absent. *Aerial conidia* hyaline, smooth- and thin-walled, of three types: *microconidia* ellipsoidal to fusoid to ovoid to obovoid to reniform to allantoid to clavate to napiform to pyriform to limoniform, 0–5-septate, borne in false heads or chains on the phialides; *mesoconidia* (occurring in some species or species complexes) falcate, slender with no significant curvature to curved with parallel walls, 1–5-septate, tapering towards both ends, with a pointed to blunt apical cell and obtuse to flattened basal cell; *macroconidia*, typically formed on sporodochia, falcate, slightly to strongly curved dorsiventrally, 1-septate to multiseptate, with a curved, long and tapering, pointed, blunt, hooked or elongated apical cell and obtuse, poorly developed, well-developed, to elongate, foot-shaped basal cell. *Sporodochia* cream to pale tan to orange to saffron to blue; *sporodochial conidiophores* verticillately branched and densely packed, consisting of short, smooth- and thin-walled stipes bearing an apical whorl of 2–4 monophialides; *sporodochial conidiogenous cells* subulate to subcylindrical, smooth- and thin-walled, with reduced or flared collarette; *sporodochial (macro)conidia* falcate, smooth- and thin-walled, distinctly curved to curved with parallel walls to unequally curved, tapering towards both ends, with pointed, blunt, papillate, hooked, or elongate apical cell and obtuse, poorly developed, well-developed, to elongate, foot-shaped basal cell. *Chlamydospores* globose to subglobose to ovoid to obovoid, hyaline to subhyaline, smooth-walled to slightly verrucose, terminal or intercalary, solitary or in pairs or forming chains or aggregating to form microsclerotia.

[Description adapted from Rossman *et al.* (1999) and Lombard *et al.* (2015)].  

*Diagnostic features*: Dark blue to black perithecia producing clavate asci bearing ellipsoidal to cylindrical 1- to multiseptate ascospores and asexual morphs producing micro- and macroconidia, and sometimes mesoconidia on aerial conidiophores with mono- and/or polyphialides or only macroconidia in sporodochia. Chlamydospores form in hyphae, rarely in macroconidia.  

**Fig. 23:**
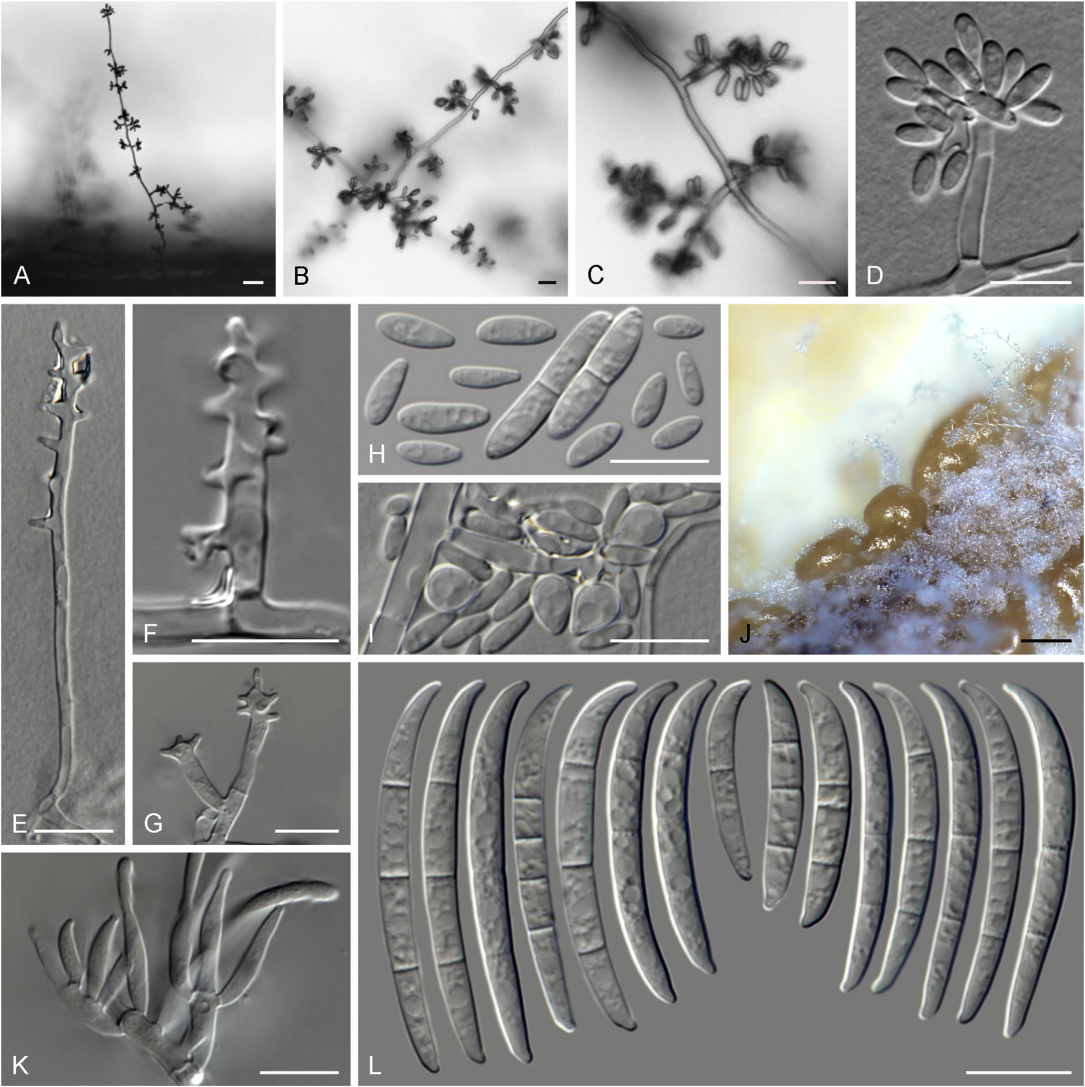
*Fusarium echinatum* (CBS 146497). **A–D.** Aerial conidiophores. **E–G.** Conidiogenous cells on aerial conidiophores. **H, I.** Microconidia. **J.** Sporodochia formed on the surface of carnation leaves. **K.** Sporodochial conidiophores and conidiogenous cells. **L.** Macroconidia. Scale bars: A = 20 μm; J = 100 μm; all others = 10 μm.

***Fusarium echinatum*** Sand.-Den. & G.J. Marais, ***sp. nov*.** MycoBank MB 838660. Fig. 23.

*Etymology:* From the Latin *echinatus*, prickly, referring to the spiny appearance of its multiloculate, often swollen and rather deformed conidiogenous cells.  

*Typus*: **South Africa**, unidentified tree species, 2010, A. Lubben (**holotype** CBS H-24658, culture ex-type CBS 146497 = CPC 30815 = CAMS 000733).  

*Conidiophores* on aerial mycelium 10–120 μm tall, unbranched or irregularly laterally branched, bearing lateral and terminal single phialides; *aerial conidiogenous cells* polyphialidic, subulate, subcylindrical or more commonly irregularly shaped, curved, swollen and distorted due to abundant conidiogenous loci, smooth- and thin-walled, 6.5–36.5 × 2–3.5 μm, polyphialides with 2–3 or more commonly 10–18 conidiogenous openings, with inconspicuous to absent periclinal thickening and collarettes. *Aerial microconidia* forming small false heads on tips of phialides, hyaline, smooth, and thin-walled, commonly ovoid to ellipsoidal, 0- or 1-septate, 4–11(–19) × 2–3.5(–4.5) μm (av. 7.5 × 2.7 μm), and more rarely napiform, smooth and thin-walled, 0-septate, (5–)5.5–7 × (3.5–)4.5–5.5 μm (av. 6.4 × 4.5 μm). *Sporodochial conidiophores* 28.5–60(–68.5) μm tall, irregularly branched, bearing terminal solitary monophialides or whorls of up to three monophialides. *Sporodochial conidiogenous cells* monophialidic*,* subulate to subcylindrical, smooth- and thin-walled, (8.5–)11.5–16(–17.5) × (1.5–)2.5–3.5 μm. *Sporodochial macroconidia* moderately curved to wedge-shaped, slender, tapering towards the basal part, apical cell of equal size than the adjacent cell, blunt to slightly hooked; basal cell poorly to well-developed, foot-shaped, (1–)2–3(–4)-septate, hyaline, thin- and smooth-walled: 1-septate conidia: (16.5–)19.5–32.5(–36) × 2.5–3.5 μm (av. 26.1 × 2.9 μm); 2-septate conidia: (19.5–)25–36(–37.5) × 2.5–3.5 μm (av. 30.5 × 3.1 μm); 3-septate conidia: (20.5–)28.5–36(–40) × (2.5–)3–3.5(–4.5) μm (av. 32.5 × 3.2 μm); 4-septate conidia: (27–)30.5–39(–40.5) × 3–4 μm (av. 35.4 × 3.6 μm); overall: (19.5–)28.6–36.5(–40.5) × (2.5–)3–3.5(–4.5) μm (av. 32.4 × 3.2 μm). *Chlamydospores* not observed.  

*Culture characteristics*: Colonies on PDA reaching 31–63 mm diam at 25 °C after 7 d. Surface white, pale luteus to sulphur yellow, flat, woolly to cottony with radial patches of white aerial mycelium, margin regular and filiform. Reverse white, sulphur yellow to pure yellow at centre. On OA pale luteus to sulphur yellow, flat, membranous at first, quickly becoming velvety to dusty, margin regular. Reverse sulphur yellow.  

*Additional material examined*: **South Africa**, unidentified tree species, 2010, A. Lubben, culture CBS 146496 = CPC 30814 = CAMS 000730.  

*Notes:*Yilmaz *et al.* (2021) recently revised the FFSC, including formal descriptions for several species, while fixing the typification of relevant plant pathogenic and toxigenic species. Species in this complex have been traditionally organised according to their biogeographic patterns, which roughly match their phylogenetic distribution. Apart from the monophyletic American and Asian clades, the complex contains a non-monophyletic African clade, which is currently known to cluster into two distinct clades: the speciose core African clade and the African “B” clade encompassing *F. dlaminii* and *F. fredkrugeri* (O'Donnell *et al.* 2000b, Herron *et al.* 2015, Sandoval-Denis *et al.* 2018b, Yilmaz *et al.* 2021). The novel South African species *F. echinatum*, however, formed a fully-supported single lineage that did not belong to any of the currently known biogeographically defined clades (Fig. 11). The most noticeable morphological feature that distinguishes *F. echinatum* is the presence of well-developed polyphialides bearing multiple conidiogenous openings that are often concentrated in large numbers and that cause a deformation of the apical region. Somewhat similar, conspicuous polyphialides can be found in *Fusarium chlamydosporum* and *F. concolor* (syn. *F. polyphialidicum*); however, these species are not directly related, in that they belong to two different species complexes, the *F. chlamydosporum* and *F. concolor* species complexes, respectively (Fig. 10). The polyphialides formed by these two species do not show as many conidiogenous loci as do those of *F. echinatum*.  

**Fig. 24:**
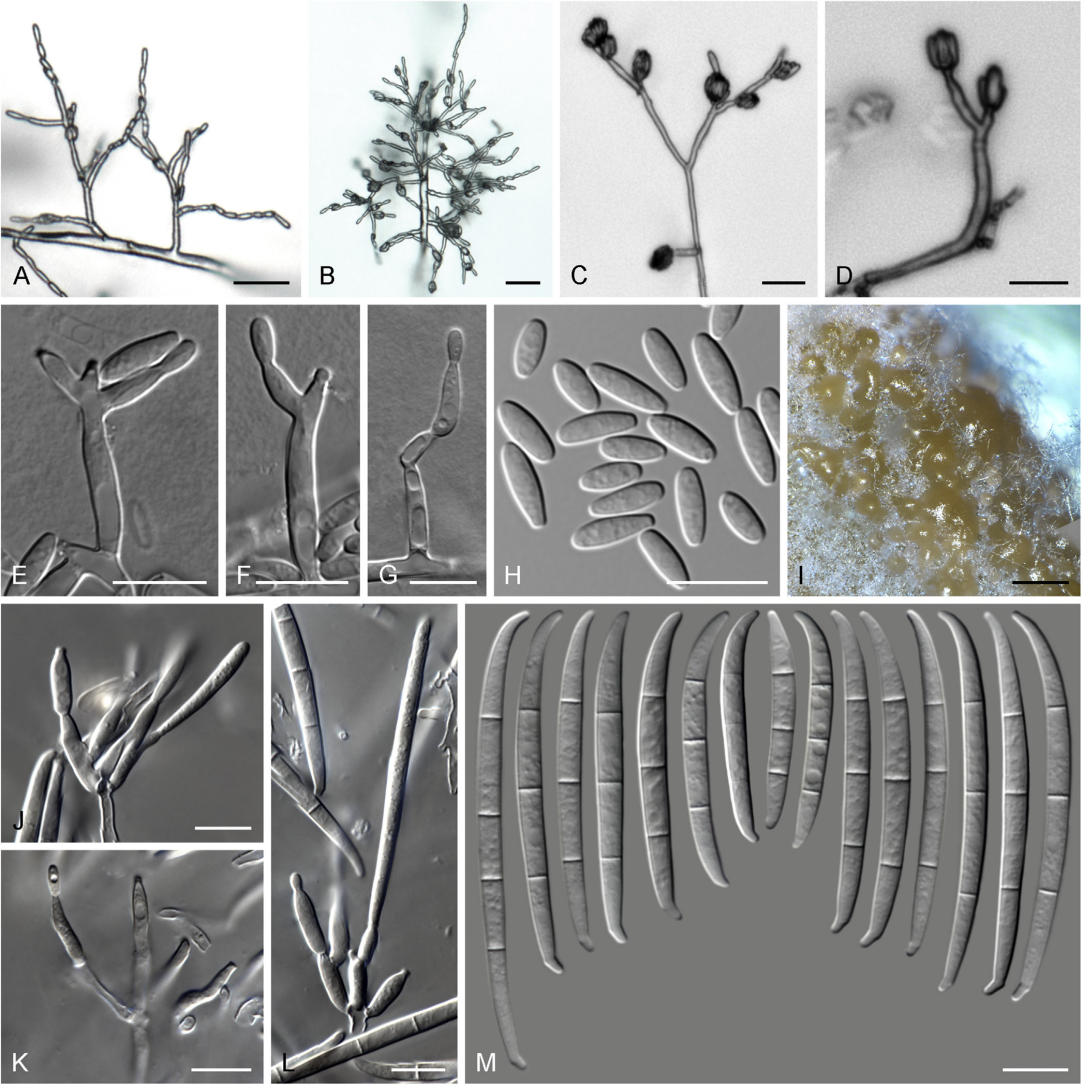
*Fusarium prieskaense* (CBS 146498). **A–D.** Aerial conidiophores. **E–G.** Conidiogenous cells on aerial conidiophores. **H.** Microconidia. **I.** Sporodochia formed on the surface of carnation leaves. **J–L.** Sporodochial conidiophores and conidiogenous cells. **M.** Macroconidia. Scale bars: A, B = 20 μm; I = 100 μm; all others = 10 μm.

***Fusarium prieskaense*** G.J. Marais & Sand.-Den., ***sp. nov*.** MycoBank MB 838661. Fig. 24.

*Etymology:* Referring to Prieska, a town in Northern Cape Province, South Africa, where the type was collected.  

*Typus*: **South Africa**, Northern Cape Province, Prieska, on *Prunus spinosa*, 2010, F.J.J. van der Walt & G.J. Marais (**holotype** CBS H-24660, culture ex-type CBS 146498 = CPC 30826 = CAMS 001176).  

*Conidiophores* on aerial mycelium 12.5–43.5 μm tall, unbranched or rarely irregularly or sympodially branched and proliferating, bearing terminal single phialides or whorls of 2–3 phialides, commonly reduced to solitary conidiogenous cells borne laterally on hyphae; *aerial conidiogenous cells* mono- and polyphialides, subulate to subcylindrical, smooth- and thin-walled, 8–29.5 × 2–5 μm, polyphialides often with 2–3 conidiogenous openings, periclinal thickening and collarettes often inconspicuous or absent. *Aerial microconidia* forming small false heads and short chains on phialide tips, hyaline, obovoid to short clavate, smooth and thin-walled, 0-septate, (4.5–)6–9(–13) × 2–3(–4) μm (av. 7.4 × 2.6 ìm). *Sporodochial conidiophores* 24.5–39(–45) μm tall, irregularly branched, bearing terminal solitary or whorls of 2–3 phialides. *Sporodochial conidiogenous cells* monophialidic, doliiform, subulate to subcylindrical, smooth- and thin-walled, (8.5–)10–14(–15) × 2–4.5 μm. *Sporodochial conidia* straight to moderately curved and slender, tapering towards the basal part, apical cell more or less equally sized as the adjacent cell, blunt to slightly hooked; basal cell well-developed, foot-shaped, rarely papillate, (1–)3–4-septate, hyaline, thin- and smooth-walled: 1-septate conidia: 23.5 × 3.5 μm; 3-septate conidia: (33.5–)44.5–58(–68.5) × (3–)3.5–4.5(–5) μm (av. 51.1 × 4 μm); 4-septate conidia: (52.5–)55.5–67.5(–71) × 3.5–4.5 μm (av. 61.3 × 4.1 μm); overall: (23–)44–59(–71) × 3–4(–5) μm (av. 51.3 × 4 μm). *Chlamydospores* not observed.  

*Culture characteristics:* Colonies on PDA reaching 42–68 mm diam at 25 °C after 7 d. Surface pale luteous, luteous to pale sienna, flat, velvety to felty, sometimes with small white patches of aerial mycelium, margin filiform and regular. Reverse sulphur yellow to amber, pale orange at centre. On OA, sienna to pale umber, flat, membranous to dusty, margin entire and regular; reverse sienna to pale umber.  

*Additional material examined*: **South Africa**, Northern Cape Province, Prieska, on *Prunus spinosa*, 2010, F.J.J. van der Walt & G.J. Marais, culture CBS 146499 = CPC 30827 = CAMS 001177; on *Aloidendron dichotomum*, 2010, F.J.J. van der Walt & G.J. Marais, culture CPC 30825 = CAMS 001175.  

*Notes: Fusarium prieskaense* is nested within the core African clade of the FFSC (Fig. 11). Similar to most members of this clade, this species is characterised by forming mostly monophialides and occasional to frequent polyphialides, sometimes proliferating and producing aerial conidia typically organised in a combination of false heads and short to long chains. *Fusarium prieskaense* is morphologically and phylogenetically related to *Fusarium brevicatenulatum* and *F. pseudonygamai* from which it can be differentiated by its pale luteus to yellow colony pigmentation on PDA, versus the orange to dark blue or violet pigments produced by the two latter species (Leslie & Summerell 2006). Additionally, sporodochia and macroconidia are commonly and abundantly produced by *F. prieskaense*, whereas these structures are relatively rare in the two aforementioned species. Moreover, the obovoid to short clavate microconidia of *F. prieskaense* also distinguishes this species from *F. brevicatenulatum*, which is characterised by long oval to obovoid microconidia (Nirenberg *et al.* 1998).  

**Fig. 25:**
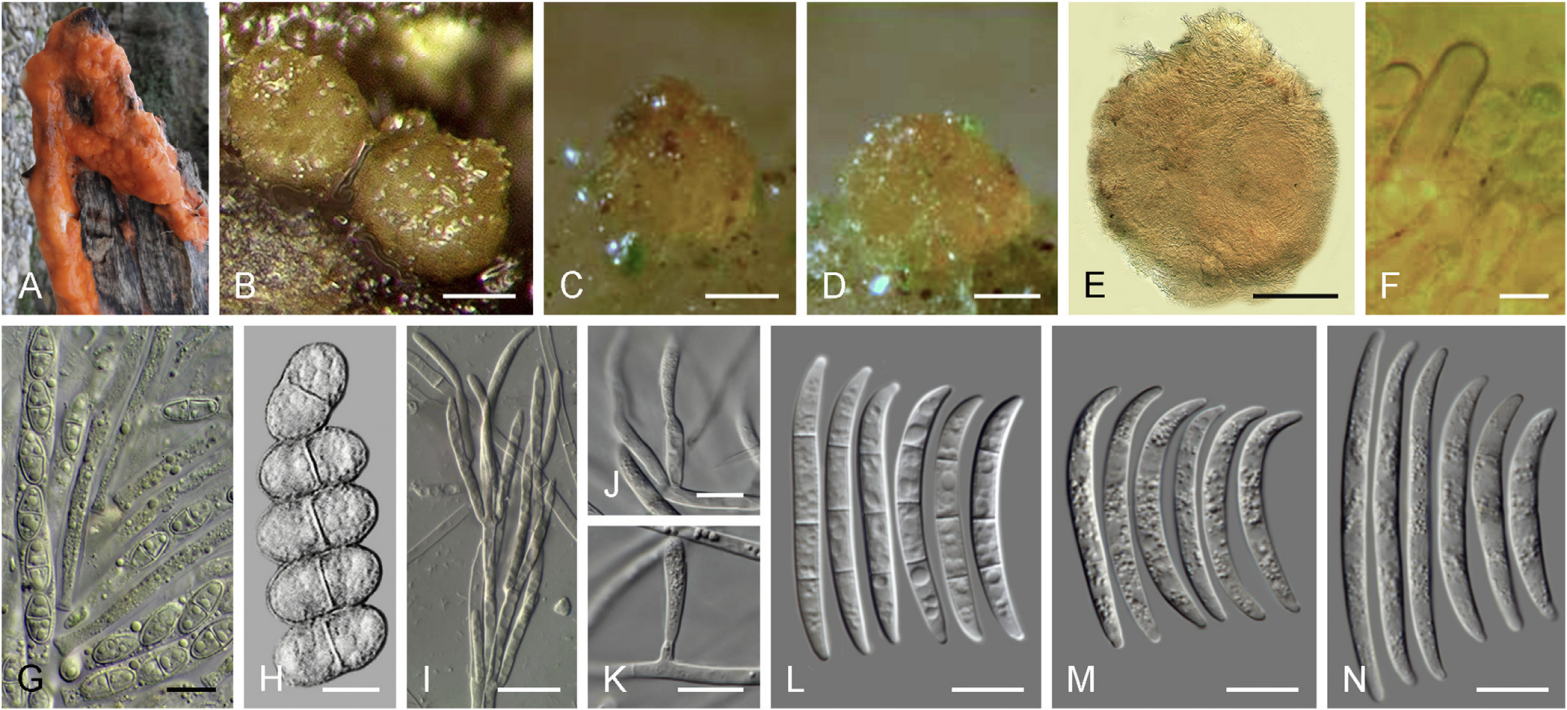
*Fusicolla* spp. **A.** Slimy macroscopic growth on natural substrate. **B–E.** Ascomata on natural substrate. **F.** Ostiolar hairs. **G.** Asci. **H.** Ascospores. **I–K.** Conidiophores and conidiogenous cells. **L–N.** Macroconidia. A. *Fusicolla merismoides* (photo J. Cunningham). B. *Fusicolla melogrammae* [CLL 16006, adapted from Crous *et al.* (2016)]. C–H. *Fusicolla ossicola* (photos N. Aplin and P. Cannon). I. *Fusicolla merismoides* (photo P. Cannon). J, K, M. *Fusicolla aquaeductuum* (CBS 734.79). L. *Fusicolla violacea* (CPC 38810). N. *Fusicolla matuoi* (CBS 581.78). Scale bars: B–E = 100 μm; F, H. 5 μm; all others = 10 μm.

***Fusicolla*** Bonord., Handb. Allg. Mykol.: 150. 1851. Figs 8, 25.

*Type species*: *Fusicolla betae* (Desm.) Bonord., Handb. Allg. Mykol.: 150. 1851.

(See *F. betae* in List section for synonyms)  

*Ascomata* perithecial, solitary, rarely gregarious, with erumpent stroma, fully or partially immersed in a slimy, pale orange sheet of hyphae over the substrate, globose to pyriform with a short acute or disk-like papilla, not collapsing when dry, yellow, pale buff to orange, not changing colour in KOH, smooth-walled, rarely tuberculate, generally lacking hairs or with short, thick-walled hyphae-like structures. *Asci* cylindrical to narrowly clavate, with an apical ring, 8-spored. *Ascospores* broadly ellipsoidal, 1-septate, slightly constricted at the septum, verrucose, hyaline to pale brown. *Conidiophores* initially as lateral phialides on somatic hyphae, sometimes monochasial, verticillate or penicillate, hyaline. *Conidiogenous cells* monophialidic, cylindrical to subulate, hyaline. *Microconidia* absent or sparse, ellipsoidal to allantoid, aseptate, hyaline. *Macroconidia* falcate, straight to curved, narrowing towards the ends, apical cell often hooked with a pointed tip, basal cell poorly developed, foot-shaped, 1–3-septate or 3–5-septate or up to 10-septate, hyaline. *Chlamydospores* absent to abundant, globose, single, in pairs or chains, sometimes formed in macroconidia.

[Description adapted from Gerlach & Nirenberg (1982) and Gräfenhan *et al.* (2011)].  

*Diagnostic features*: Yellow to orange, mostly smooth-walled perithecia with a short acute or disk-like papilla producing cylindrical to narrowly clavate asci bearing broadly ellipsoidal, 1-septate, verrucose ascospores and fusarioid asexual conidia.  

**Fig. 26:**
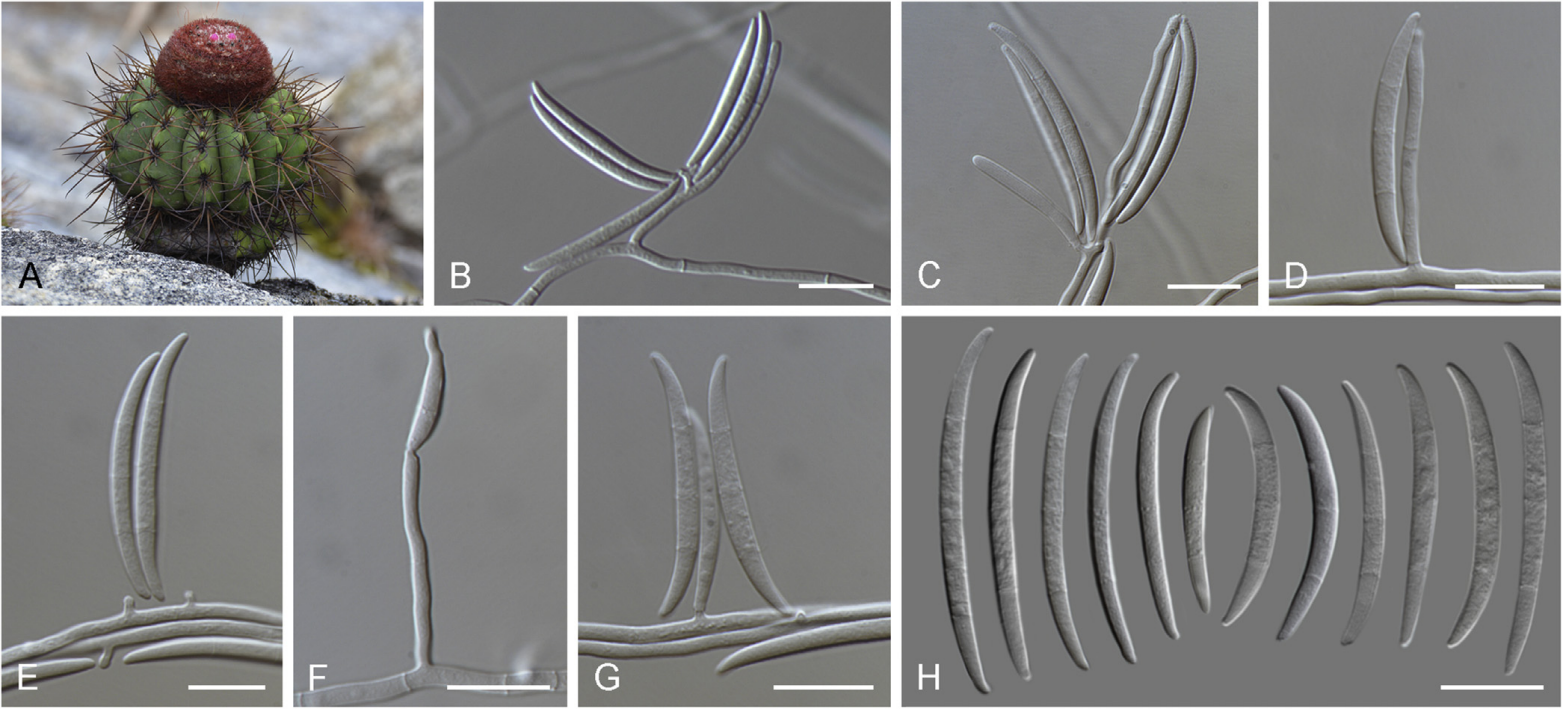
*Fusicolla quarantenae* (URM 8367). **A.** Host. **B–G.** Conidiophores, conidiogenous cells and conidia. **H.** Macroconidia. Scale bars = 10 μm.

***Fusicolla quarantenae*** J.D.P. Bezerra, Sand.-Den., Crous & Souza-Motta, ***sp. nov*.** MycoBank MB 838692. Fig. 26.

*Etymology:* The epithet refers to the quarantine period during the 2020–2021 coronavirus pandemic, which killed thousands of people on five continents, and during which this species was described.  

*Typus*: **Brazil**, Pernambuco state, Itaíba municipality, Curral Velho Farm, 9º08.895 S 37º12.069 W, on cladodes of *Melocactus zehntneri*, Sep. 2013, J.D.P. Bezerra (**holotype** URM 94407, culture ex-type URM 8367 = CBS 141541).  

*Conidiophores* arising laterally from somatic hyphae, simple, straight, hyaline, thin- and smooth-walled, septate, 25–116 × 1.5–2.5 μm, or reduced to solitary conidiogenous cells. *Conidiogenous cells* monophialidic, arising laterally from hyphae, cylindrical to subulate, straight, hyaline, thin- and smooth-walled, 1–22 × 0.5–2 μm, or as short lateral pegs. *Macroconidia* falcate, more or less straight, slightly narrowing towards the ends, apical cell often hooked with a more or less pointed tip, basal cell poorly developed, foot-shaped, hyaline, thin- and smooth-walled, 3-septate, (21–)27–35(–38.5) × 2–2.5(–3) μm (av. 29.5 × 2.5 μm, n = 30). *Microconidia*, *chlamydospores* and *sexual morph* not observed.  

*Culture characteristics*: Colonies on PDA reaching 15 mm diam after at 25 °C after 7 d. Surface yellow to apricot in centre, peach to brick in middle, and salmon at margin, flat, aerial mycelium absent, slimy, with entire margin; reverse yellow to brick.  

*Notes*: *Fusicolla quarantenae*, an endophyte of *Melocactus zehntneri*, is morphologically reminiscent of *Fu. betae*, *Fu. epistroma*, and *Fu. septimanifiniscientiae*, all of which produce mainly 3-septate macroconidia. *Fusicolla betae* and *Fu. epistroma* differ by having larger conidia (50–60 μm and 19–45 μm long, respectively, Gerlach & Nirenberg 1982). The absence of chlamydospores in *Fu. quarantenae* further differentiates this species from *Fu. epistroma* and *Fu. septimanifiniscientiae* (Gerlach & Nirenberg 1982, Crous *et al.* 2018).  

**Fig. 27:**
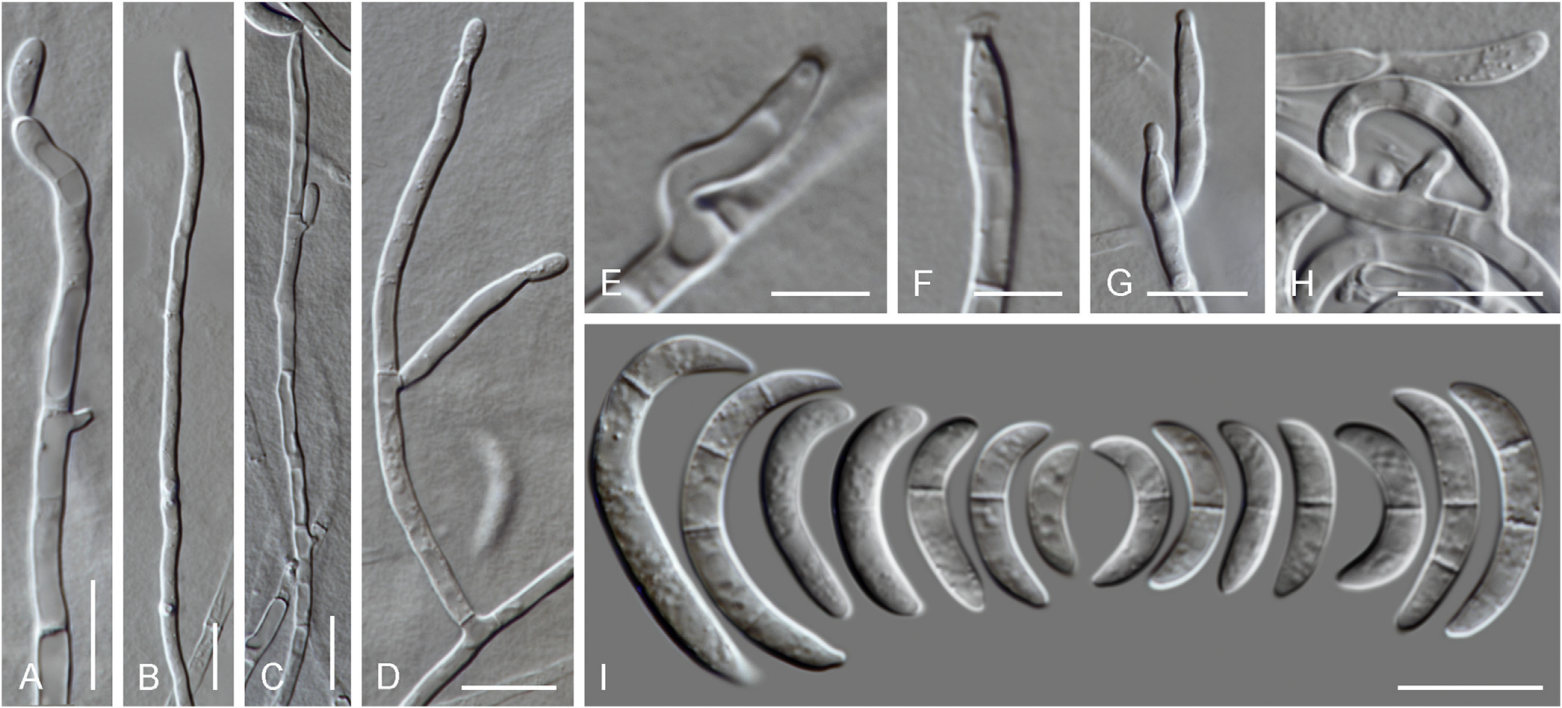
*Fusicolla meniscoidea* (CBS 110189). **A–D.** Conidiophores. **E–H.** Conidiogenous cells. **I.** Macroconidia. Scale bars: A–D, G–I = 10 μm; E, F = 5 μm.

***Fusicolla meniscoidea*** L. Lombard & Sand.-Den., ***sp. nov*.** MycoBank MB 838662. Fig. 27.

*Etymology*: From Greek *mēniskos*, crescent, in reference to the shape of its conidia.  

*Typus*: **Australia**, from soil, unknown collection date (before 1978), unknown collector (**holotype** CBS H-24662, culture ex-type CBS 110189 = FRC E-0086).  1

*Conidiophores* arising laterally or terminally from somatic hyphae 50–70 μm long, simple or sparingly branched laterally, straight, hyaline, smooth- and thin-walled, bearing terminal and lateral conidiogenous cells, or more commonly reduced to single conidiogenous cells borne laterally on the substrate and aerial hyphae. *Conidiogenous cells* monophialidic, subcylindrical, cylindrical to slightly subulate, 10.5–35 × 2–3.5 μm, smooth- and thin-walled, without noticeable periclinal thickening, a minute apical collarette can be present. *Macroconidia* falcate, tapering gently towards both ends, apical cell often hooked with a blunt to pointy apex, basal cell obtuse to poorly developed, foot-shaped, 0–2(–3)-septate, predominantly 1-septate, hyaline, smooth- and thin-walled; 0-septate (8–)9–13(–15) × 2–3.5 μm (av. 11.1 × 2.9 μm); 1-septate, (9–)11.5–15(–17.5) × 2.5–3.5 μm (av. 13.1 × 2.9 μm); 2-septate, 13–17.5(–18) × 2.5–4 μm (av. 15.4 × 3 μm); 3-septate, 20–24.5(–25.5) × 3–3.5 μm (av. 22.6 × 3.3 μm). *Microconidia*, *chlamydospores* and *sexual morph* not observed.  

*Culture characteristics*: Colonies on PDA reaching 21–30 mm diam at 25 °C after 7 d. Surface white to pale luteus at periphery, centre salmon to pale orange, flat to slightly radially folded, membranous to slimy, margin entire to slightly undulate; reverse luteous to pale salmon at centre. On OA, pale luteous to pale salmon, flat, membranous, margin entire; reverse pale luteous.  

*Notes*: *Fusicolla meniscoidea* is here introduced based on an isolate originally misidentified as *Bisifusarium dimerum*. Despite the great genetic differences and phylogenetic distance, the two taxa share similar morphological traits, particularly regarding macroscopic aspects of colonial growth, and the shape and size of conidiophores and conidia. However, unlike in *B. dimerum*, conidia of *Fu. meniscoidea* present a much more pronounced curvature involving both conidial planes (somewhat parallel walls), while foot-shaped basal cells are less evident or absent. *Fusicolla aqueductuum*, *Fu. betae*, *Fu. quarantenae*, and *Fu. violacea* are all morphologically related to *Fu. meniscoidea* by showing similar conidial septation ranges and lacking chlamydospores. Conidial size in *Fu. meniscoidea* is, however, markedly reduced and often closer to the lower limits of the conidial size of all the aforementioned species. Another species also described here, *Fusicolla sporellula*, lacks chlamydospores but has similar, although smaller, conidia with a reduced range of septa (0- or 1-septate). It furthermore differs from *Fu. meniscoidea* by its shorter and doliiform conidiogenous cells.  

**Fig. 28:**
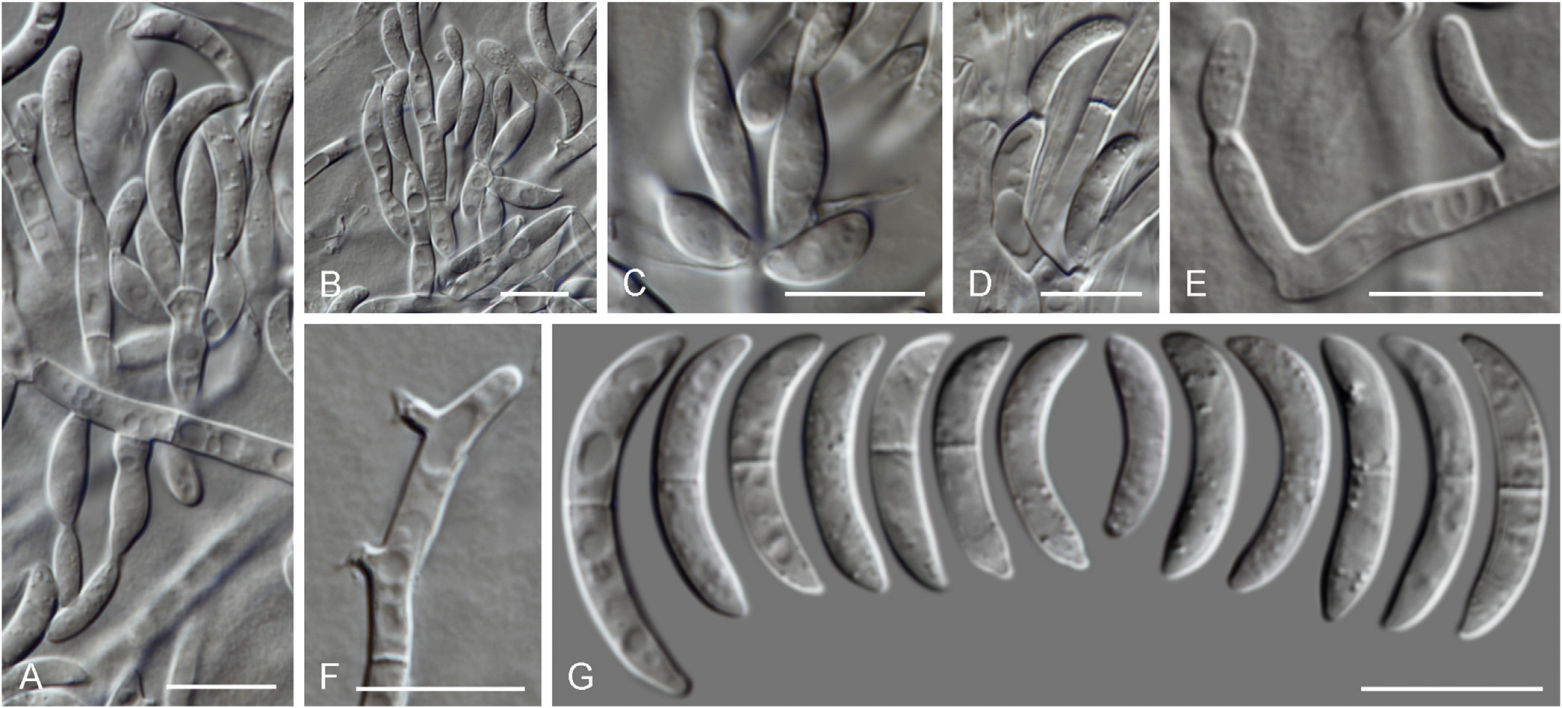
*Fusicolla sporellula* (CBS 110191). **A–C.** Conidiophores. **D–F.** Conidiogenous cells. **G.** Macroconidia. Scale bars = 10 μm.

***Fusicolla sporellula*** Sand.-Den. & L. Lombard, ***sp. nov*.** MycoBank MB 838663. Fig. 28.

*Etymology:* From Latin, very small spores, in reference to its very small conidia.  

*Typus*: **South Africa**, Transkei, from soil, unknown collection date (before 1983), unknown collector (**holotype** CBS H-24663, culture ex-type CBS 110191 = FRC E-0139).  

*Conidiophores* arising laterally from substrate and aerial hyphae 14–35 μm long, simple or laterally and verticillately branched, straight, hyaline, smooth- and thin-walled, or reduced to single conidiogenous cells. *Conidiogenous cells* monophialidic, doliiform, short lageniform to subulate 7.5–20 × 2.5–4 μm, smooth- and thin-walled, with or without inconspicuous periclinal thickening, collarettes absent; or reduced to short phialidic pegs emerging laterally from hyphae, 1–5 × 1–2.5 μm, smooth- and thin-walled, with inconspicuous periclinal thickening and an often conspicuously flared collarette. *Macroconidia* lunate to falcate, moderately to strongly dorsiventrally curved, slightly narrowing towards both ends, apical cell blunt, more or less hooked, basal cell obtuse to poorly developed, foot-shaped, hyaline, thin- and smooth-walled, 0- or 1-septate, predominantly 1-septate, 0-septate: (11–)12–14(–15) × 2–3(–3.5) μm (av. 13.2 × 2.7 μm), 1-septate: (11.5–)13–16.5(–20) × 2.5–3.5 μm (av. 14.6 × 2.8 μm). *Microconidia, chlamydospores*, and *sexual morph* not observed.  

*Culture characteristics*: Colonies on PDA reaching 24–31 mm diam at 25 °C after 7 d. Surface white, luteous to orange, flat to slightly radially folded, membranous to slimy, margin entire; reverse pale luteous to saffron, peach at centre. On OA, pale luteous to peach, flat, membranous with filiform to undulate margins; reverse pale peach to saffron.  

*Notes*: *Fusicolla sporellula* presents the smallest conidia described to date for any species in this genus. This taxon is phylogenetically and morphologically related to *Fu. meniscoidea,* from which it can be differentiated by its smaller and less septate conidia, and by the characteristic doliiform shape of its conidiogenous cells.  

**Fig. 29:**
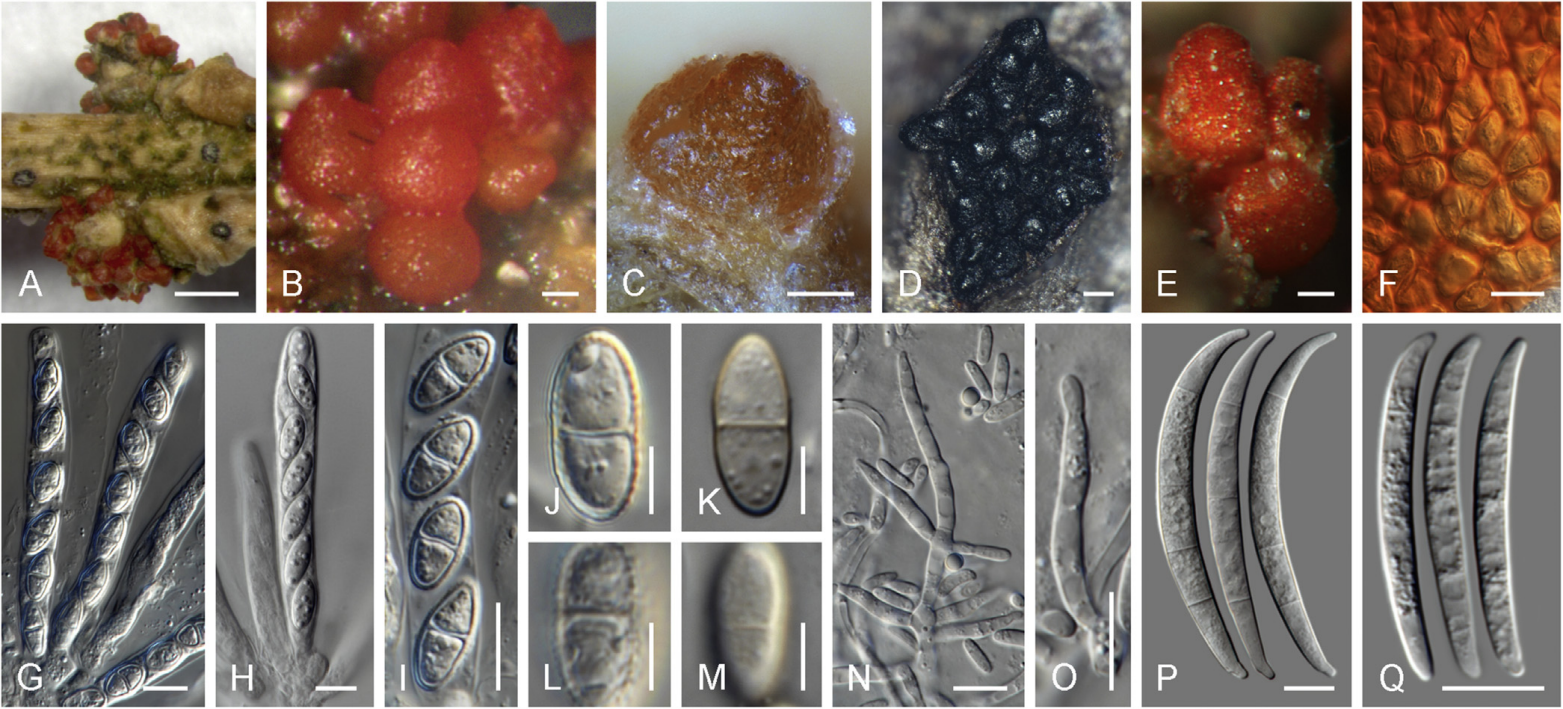
*Geejayessia* spp. **A–E.** Ascomata on natural substrate. **F.** Surface view of perithecial wall in 2 % KOH. **G–I.** Asci. **J–M.** Ascospores. **N, O.** Conidiophores and conidiogenous cells. **P, Q.** Macroconidia. A, C. *Geejayessia cicatricum* [CBS H-20375, adapted from Schroers *et al.* (2011)]. C. *Geejayessia cicatricum* (CBS H-20374). D, H, K, M. *Geejayessia atrofusca* (CBS H-20381). E–G, I, J, L. *Geejayessia desmazieri* (CBS H-20372). N, O, Q. *Geejayessia atrofusca* (CBS 502.94). P. *Geejayessia cicatricum* (CBS 125549). Scale bars: A = 500 μm; B, D, E = 200 μm; C = 100 μm; J–M = 5 μm; all others = 10 μm.

***Geejayessia*** Schroers *et al.*, Stud. Mycol. 68: 124. 2011. Figs 8, 29.

*Type species*: *Geejayessia cicatricum* (Berk.) Schroers, Stud. Mycol. 68: 124. 2011.

(See *F. cicatricum* in List section for synonyms)  

*Ascomata* perithecial, caespitose, with erumpent, byssoid or densely prosenchymatous stroma, superficial, broadly ampulliform with short ostiolar neck to broadly ellipsoidal, not collapsing when dry, pale orange, brownish to reddish orange, bright, reddish black or black, changing colour in KOH if not black and becoming purple in lactic acid, mostly smooth-walled, lacking hairs or appendages. *Ascomatal wall* consists of a single region, comprising several layers of morphologically similar cells. *Asci* cylindrical to clavate, with a broadly rounded or flattened apex, with or without a minute refractive ring, 8-spored, mostly overlapping, uniseriate or biseriate above and uniseriate below. *Ascospores* broadly ellipsoidal to ellipsoidal, 1-septate, slightly constricted at the septum, verrucose, hyaline to pale brown. *Conidiophores* mononematous (aerial conidiophores) or grouped on sporodochia. *Aerial conidiophores* unbranched, sympodial or irregularly branched, bearing terminal or lateral phialides, often reduced to single phialides. *Conidiogenous cells* monophialidic, subcylindrical to cylindrical, smooth- and thin-walled, with periclinal thickening inconspicuous or absent. *Aerial conidia* hyaline, smooth- and thin-walled, of two types: *microconidia*, present in some species, ellipsoidal to fusoid, 0- or 1-septate, with rounded ends, straight to slightly curved; *macroconidia* typically formed on sporodochia, falcate, straight to gently curved dorsiventrally, 3–8-septate, with a blunt apical cell and well-developed foot-shaped basal cell. *Sporodochia* cream to pale yellow; *sporodochial conidiophores* verticillately branched and densely packed, consisting of short, smooth- and thin-walled stipes bearing an apical whorl of 2–3 monophialides; *sporodochial conidiogenous cells* monophialidic, cylindrical to subcylindrical, smooth- and thin-walled, with reduced or flared collarette. *Chlamydospores* unknown.

[Description adapted from Schroers *et al.* (2011) and Lombard *et al.* (2015)].  

*Diagnostic features*: Pale orange, brownish to reddish orange, bright red, reddish black to black, mostly smooth-walled perithecia with short ostiolar neck producing clavate to cylindrical asci bearing ellipsoidal, 1-septate, verrucose ascospores and asexual morphs producing only macroconidia on sporodochia or micro- and macroconidia on elongate subulate to subcylindrical aerial conidiophores with monophialides. *Chlamydospores* absent.  

***Ilyonectria*** P. Chaverri & C. Salgado, Stud. Mycol. 68: 69. 2011. Fig. 8.  

*Type species*: *Ilyonectria destructans* (Zinssm.) Rossman *et al.*, Stud. Mycol. 80: 217. 2015.

(See *F. aderholdii* in List section for synonyms)  

*Ascomata* perithecial, solitary or gregarious, non-stromatic, superficial, globose to subglobose or ovoid to obpyriform, red, turning purple to dark purple in KOH, pigment dissolving in lactic acid, not collapsing when dry, with broadly conical papilla or flattened apex, smooth to slightly rugulose, lacking hairs or appendages. *Ascomatal wall* of two regions: outer region of thick-walled, pigmented cells forming a *textura globosa*; inner region of compressed, flattened cells, becoming thinner towards the centrum. *Asci* narrowly clavate to cylindrical, 8-spored, apex subtruncate, with inconspicuous apical ring, uniseriate. *Ascospores* ellipsoidal, 1-septate, hyaline, smooth. *Conidiophores* simple or complex or sporodochial; *simple conidiophores* arising laterally or terminally from aerial mycelium, solitary or loosely aggregated, unbranched or sparsely branched, bearing up to three phialides; *complex conidiophores* solitary or aggregated in small sporodochia, repeatedly and irregularly branched. *Conidiogenous cells* monophialidic, cylindrical, tapering towards the apex. *Microconidia* 0- or 1-septate, ovoid to fusoid to ellipsoidal, with a minutely or clearly laterally displaced hilum, formed in heads on solitary conidiophores or as masses on sporodochia. *Macroconidia* straight, cylindrical, 1–3(–4)-septate, with both ends obtusely rounded, base sometimes with a visible, centrally located to laterally displaced hilum, forming flat domes of slimy masses. *Chlamydospores* globose to subglobose, thick-walled, intercalary or solitary, initially hyaline, becoming brown with age.

[Description adapted from Chaverri *et al.* (2011)].  

*Diagnostic features*: Red, mostly smooth-walled perithecia with conical papilla or flattened apex producing cylindrical asci bearing ellipsoidal, 1-septate ascospores and cylindrocarpon-like asexual morph characterised by 1–3(–4)-septate macroconidia with centrally located to laterally displaced hilum.  

**Fig. 30:**
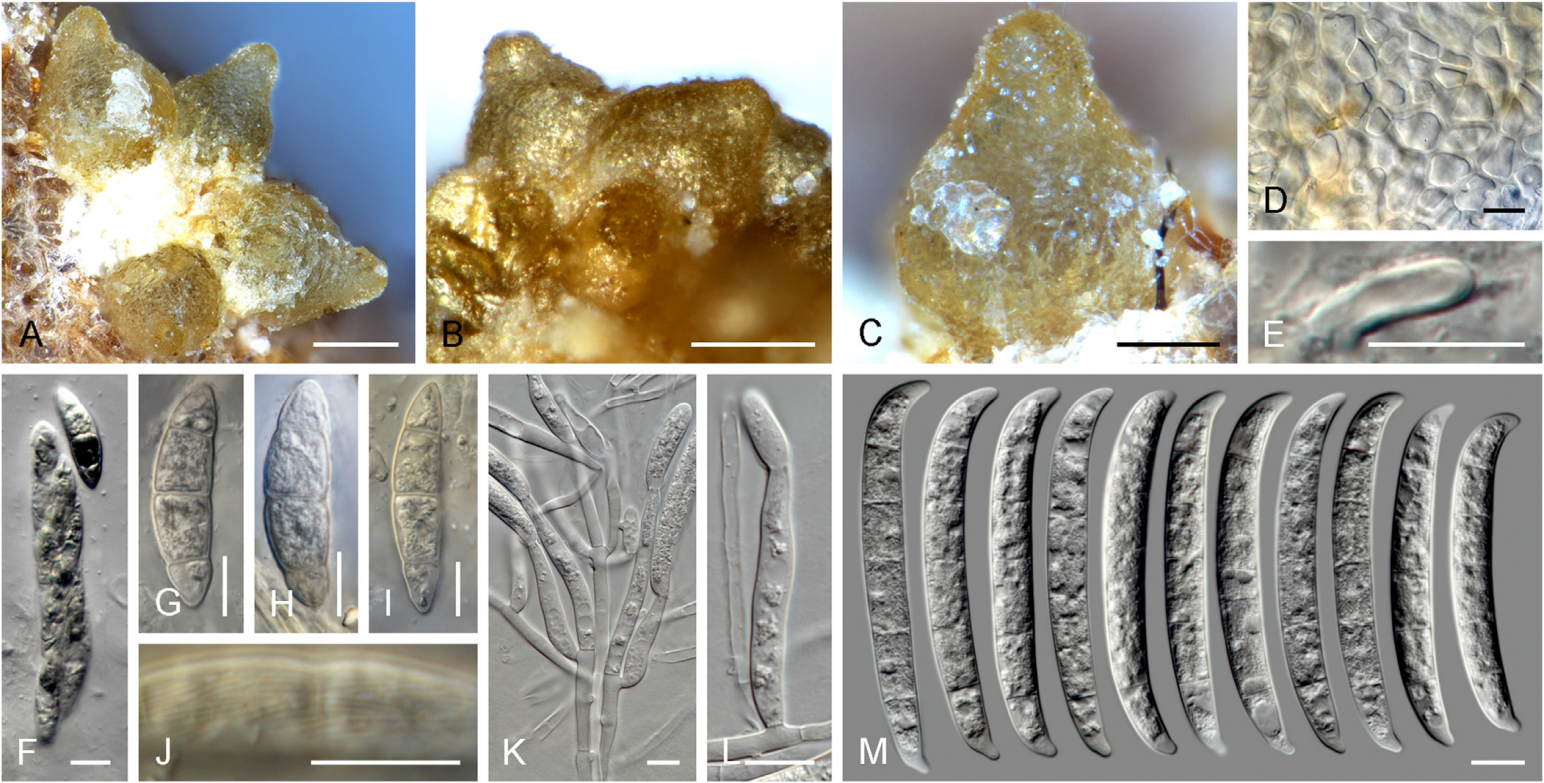
*Luteonectria albida*. **A–C.** Ascomata on natural substrate. **D.** Surface view of perithecial wall in lactic acid. **E.** Detail of ascomata hair. **F.** Asci. **G–J.** Ascospores (J. Surface view). **K, L.** Conidiophores and conidiogenous cells. **M.** Macroconidia. A, C. BPI 550103. B. BPI 1108874. D–J. BPI 1108875. K–M. CBS 102683. Scale bars: A, B = 100 μm; C = 50 μm; all others = 10 μm.

***Luteonectria*** Sand.-Den., L. Lombard, Schroers & Rossman, ***gen. nov*.** MycoBank MB 838664. Figs 8, 30.

*Etymology*: Name refers to the luteous coloured, nectria-like ascomata characteristic of these fungi.  

*Type species*: *Luteonectria albida* (Rossman) Sand.-Den. & L. Lombard  

*Ascomata* perithecial, gregarious on a well-developed stroma composed of pseudoparenchymatous cells, covered with loose, white hyphae, smooth and thin-walled, globose to pyriform, off-white to pale luteous, becoming ochraceous when dry, with a broadly rounded and papillate apical region, not changing colour in KOH or lactic acid, short setae-like hairs sometimes emerging from perithecial wall. *Asci* clavate with simple apex, 8-spored, ascospores overlapping irregularly uniseriate to biseriate. *Ascospore*s fusiform with rounded ends, 3-septate, slightly constricted at septum, hyaline, becoming pale yellow-brown, smooth-walled to finely striate. *Conidiophores* mononematous, septate and irregularly branched, bearing terminal phialides. *Conidiogenous cells* monophialidic, cylindrical to subcylindrical, smooth- and thin-walled, with periclinal thickening inconspicuous to absent. *Macroconidia* fusoid and multiseptate, 1–7-septate, curved, hyaline, with a wide, blunt apical cell and a poorly- to well-developed, foot-shaped basal cell. *Micro*- and *mesoconidia* unknown. *Chlamydospores* unknown.

[Description adapted from Rossman (1983) and Schroers *et al.* (2011)].

*Diagnostic features*: Off-white to pale luteous perithecia that do not change colour on KOH or lactic acid, formed on well-developed stroma producing clavate asci containing fusiform, 3-septate, finely striate ascospores and fusarioid asexual morph characterised by monophialides producing robust multiseptate conidia from aerial conidiophores, lacking micro- and mesoconidia, and chlamydospores.  

***Luteonectria albida*** (Rossman) Sand.-Den. & L. Lombard, ***comb. nov.*** MycoBank MB 838665.

*Basionym*: *Nectria albida* Rossman, Mycol. Pap. 150: 79. 1983.

*Synonyms*: *Albonectria albida* (Rossman) Guu & Y.M. Ju, Bot. Stud. (Taipei) 48: 189. 2007.

*Fusarium albidum* (Rossman) O'Donnell & Geiser, Phytopathology 103: 404. 2013.  

*Typus*: **Jamaica**, Hanover Parish, Dolphin Head Mt. near Askenish, on bark of woody stem of unknown host, 22 Jan. 1971, R.P. Korf *et al.* (**holotype** CUP-MJ 942, culture ex-type ATCC 44543 = CTR 71-110 = BBA 67603 = NRRL 13950 = NRRL 22152).  

*Description and illustration:*Rossman (1983), Guu *et al.* (2007), Schroers *et al.* (2011).  

*Additional material examined*: **Costa Rica**, Limón, Central Distrito Valle, Valle del Estrella, Selva Biologia Hitoi Caneri, 100 –150 m alt, on bark of living tree, 7 Jul. 1999, G.J. Samuels *et al.*, BPI 746587, culture CBS 102683. **Jamaica**, Newcastle, Chesterville Youth Developmental Camp, on undetermined host, 8 Jan. 1971, A.Y. Rossman, BPI 550103. **Venezuela**, Los Venados, El Avila, along Trail 1–2 km above Los Venados, El Avila, on undetermined substrate, 24 Jul. 1972, K.P. Dumont *et al.*, BPI 1108875.  

***Luteonectria nematophila*** (Nirenberg & Hagedorn) Sand.-Den. & L. Lombard, ***comb. nov.*** MycoBank MB 838666.

*Basionym*: *Fusarium nematophilum* Nirenberg & Hagedorn, Nachrichtenbl. Deutsch. Pflanzenschutzdienstes 60: 214. 2008.  

*Typus*: **Germany**, Berlin, from soil with roots of *Hedera helix*, unknown date and collector (**holotype** BBA 72279 in B, culture ex-type BBA 72279 = NRRL 54600).  

*Description and illustration*: Nirenberg & Hagedorn (2008).  

**Fig. 31:**
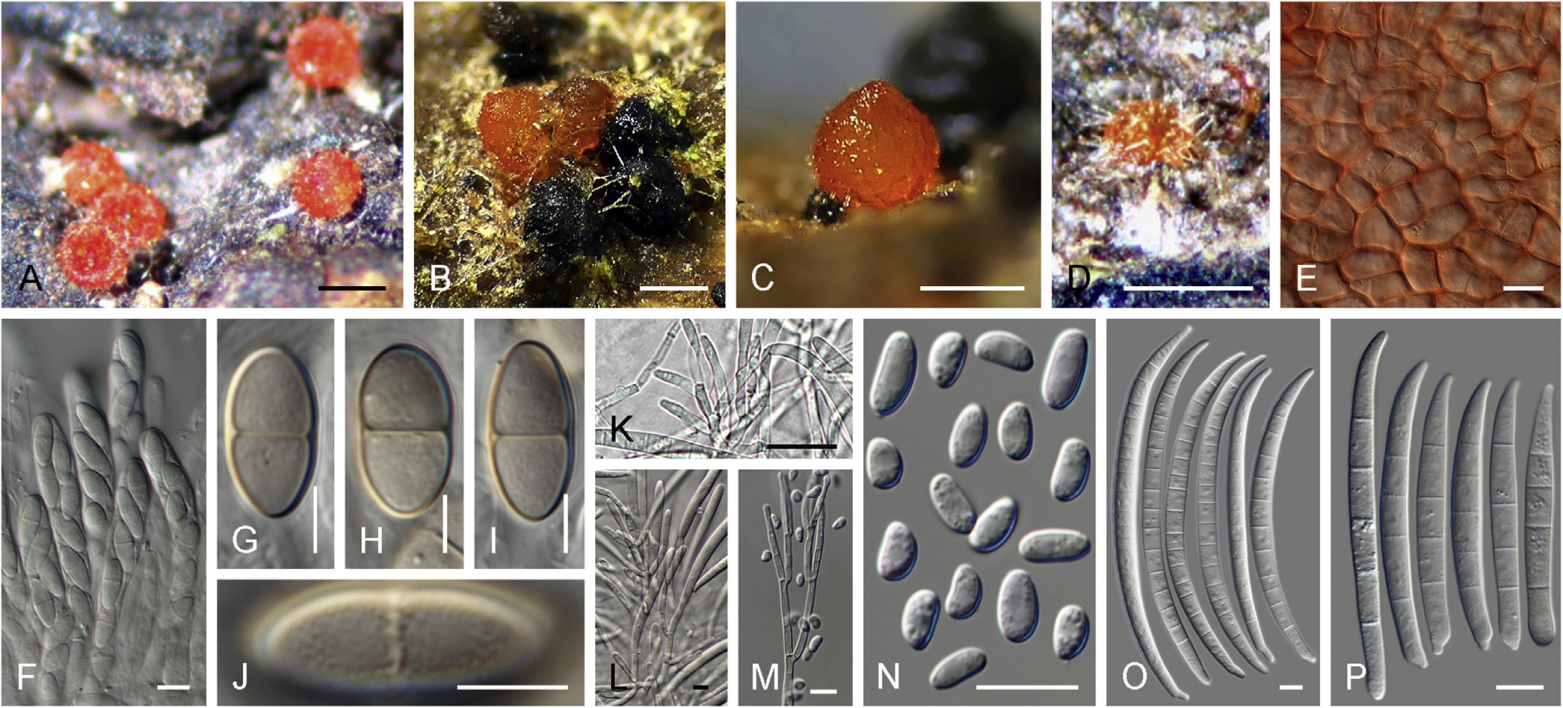
*Macroconia* spp. **A–D.** Ascomata on natural substrate. **E.** Surface view of perithecial wall in 2 % KOH. **F.** Asci. **G–J.** Ascospores (J. Surface view). **K–M.** Conidiophores and conidiogenous cells. **N.** Microconidia. **O, P.** Macroconidia. A. *Macroconia cupularis* [HMAS 97514, adapted from Luo & Zhuang (2008)]. B, C. *Macroconia leptosphaeriae* (photo P. Mlčoch). D. *Macroconia gigas* [HMAS 99592, adapted from Luo & Zhuang (2008)]. E–J. *Macroconia leptosphaeriae* (CBS H-15051). K, L. *Macroconia phlogioides* (CBS 125496). M, N. *Macroconia leptosphaeriae* (CBS 10001). O. *Macroconia phlogioides* (CBS 146500). P. *Macroconia bulbipes* (CBS 146679). Scale bars: A–D = 100 μm; G–J = 5 μm; all others = 10 μm.

***Macroconia*** (Wollenw.) Gräfenhan *et al.*, Stud. Mycol. 68: 101. 2011. Figs 8, 31.

*Basionym*: *Nectria* sect. *Macroconia* Wollenw., Angew. Bot. 8: 179. 1926.  

*Type species*: *Macroconia leptosphaeriae* (Niessl) Gräfenhan & Schroers, Stud. Mycol. 68: 102. 2011.

*Synonyms*: *Nectria leptosphaeriae* Niessl, in Krieger, Fungi Saxon. Exs.: no. 165. 1886.

*Cucurbitaria leptosphaeriae* (Niessl) Kuntze, Revis. Gen. Pl. 3: 461. 1898.

*Hypomyces leptosphaeriae* (Niessl) Wollenw., Fusaria Autogr. Delin. 1: 57. 1916.

*Lasionectria leptosphaeriae* (Niessl) Petch, Trans. Brit. Mycol. Soc. 21: 268. 1938.

*Cosmospora leptosphaeriae* (Niessl) Rossman & Samuels, Stud. Mycol. 42: 122. 1999.  

*Ascomata* perithecial, solitary, with stroma inconspicuous or absent, subglobose with or without a small apical papilla, orange to carmine red, turning dark red to violet in KOH, sometimes with hyphal hairs arising from the outer wall. *Asci* cylindrical to narrowly clavate, with a simple apex, 8-spored, uniseriate or partially biseriate. *Ascospores* yellowish, 1-septate, smooth, sometimes becoming striate when mature. *Conidiophores* initially as lateral phialides on somatic hyphae, later monochasial to verticillate, hyaline. *Conidiogenous cells* monophialidic, cylindrical to subulate, hyaline. *Microconidia* rare or absent, ellipsoidal to allantoid, hyaline. *Macroconidia* subcylindrical to curved, apical cell conical or hooked, basal cell poorly- to well-developed, foot-shaped, 3–7(–14)-septate, hyaline. *Chlamydospores* absent to rare, globose, single, in pairs or chains in hyphae.

[Description adapted from Gräfenhan *et al.* (2011)].  

*Diagnostic features*: Orange-red to carmine-red perithecia with or without a small papilla producing cylindrical to narrowly clavate asci bearing 1-septate ascospores that sometimes become striate when mature, and asexual morphs characterised by verticillate conidiophores producing large, multiseptate fusarioid macroconidia.  

**Fig. 32:**
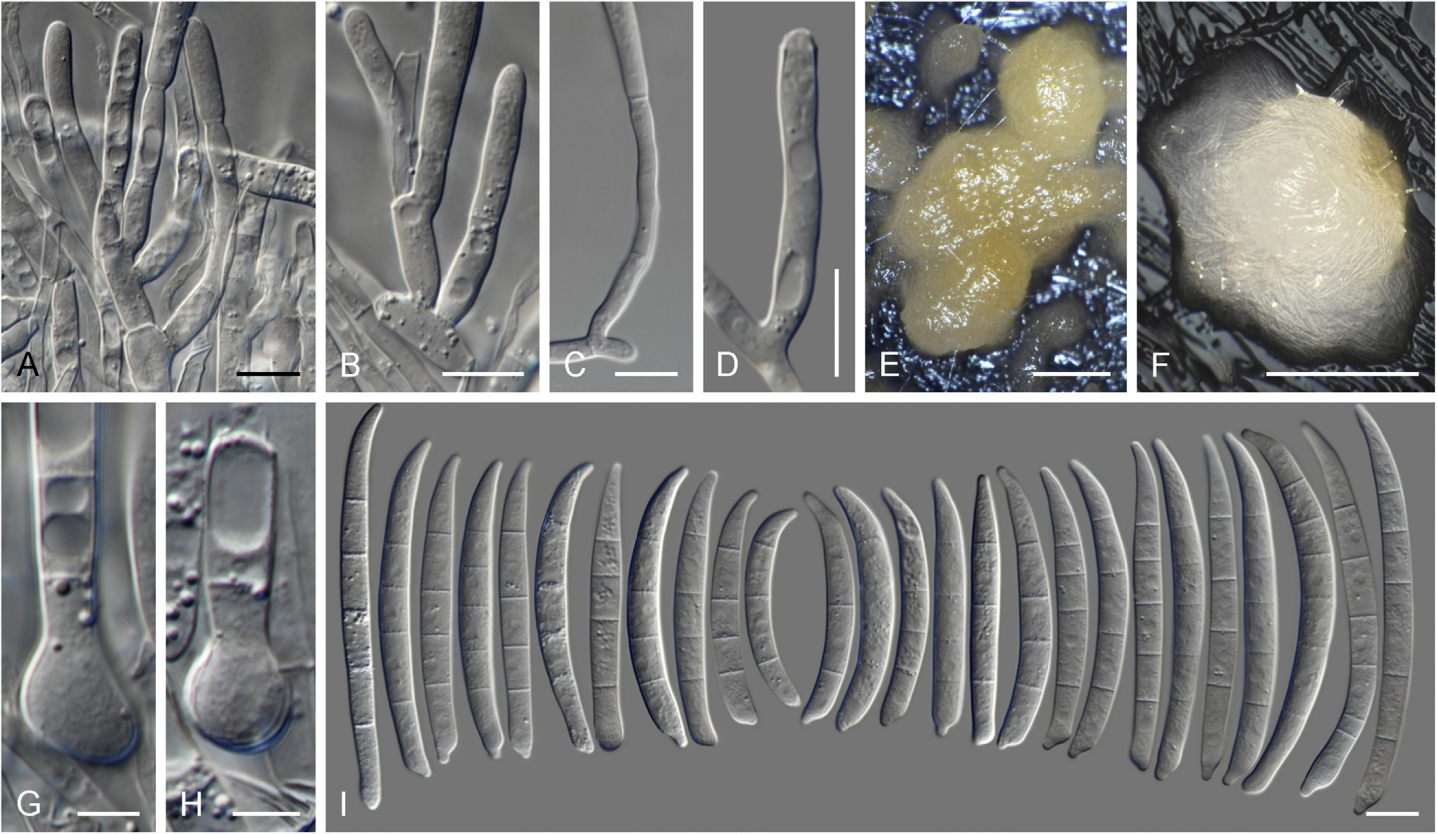
*Macroconia bulbipes* (CBS 146679). **A–D.** Conidiophores and conidiogenous cells. **E, F.** Sporodochia formed on the agar surface. **G, H.** Detail of macroconidia basal cells. **I.** Macroconidia. Scale bars: E, F = 100 μm; G, H = 5 μm; all others = 10 μm.

***Macroconia bulbipes*** Crous & Sand.-Den., ***sp. nov*.** MycoBank MB 838667. Fig. 32.

*Etymology*: Named after the shape of the basal cell, which is commonly swollen, bulbous.  

*Typus*: **South Africa**, Western Cape Province, Swellendam, Bontebok National Park, from *Erica* sp. associated with *Dimerosporiopsis engleriana*, 24 Sep. 2018, A.R. Wood (**holotype** CBS H-24664, culture ex-type CBS 146679 = CPC 37138).  

*Conidiophores* commonly aggregated into sporodochia, more rarely simple (aerial). *Aerial conidiophores* borne laterally on hyphae and commonly reduced to single conidiogenous cells, hyaline, thin- and smooth-walled, 23.5–39.6 μm long; *conidiogenous cells* monophialidic, subcylindrical to cylindrical, hyaline, (23–)24–25(–26.5) × 3–4 μm, without discernible periclinal thickening or collarettes. *Sporodochia* abundantly formed on carnation leaves and on the agar surface, pink to pink-brown coloured. *Sporodochia* light orange-peach, turning dark brick coloured in old cultures; *sporodochial conidiophores* irregularly or verticillately branched, 40–55.5 μm long, irregularly branched, bearing lateral and terminal solitary monophialides. *Sporodochial conidiogenous cells* monophialidic, cylindrical to subcylindrical to subulate, (8–)14.5–26.5(–30.5) × 3.5–5.5 μm with inconspicuous periclinal thickening, flared collarettes absent. *Microconidia* absent. *Macroconidia* straight to moderately dorsiventrally curved, tapering toward the apex, apical cell conical or hooked, and slightly extended, basal cell well-developed, foot shaped, commonly irregularly swollen at bottom, (2–)3–5(–6)-septate, predominantly 4-septate, hyaline, thick- and smooth-walled: 2-septate conidia: 43–45.5 × 5–5.5 μm (av. 44.2 × 5.1 μm); 3-septate conidia: (38.5–)41–53(–55) × 5–6 μm (av. 48.1 × 5.4 μm); 4-septate conidia: (45.5–)50–62(–67.5) × 5–6(–7) μm (av. 56.1 × 5.8 μm); 5-septate conidia: (58–)61–77(–80.5) × 5–6.5 μm (av. 68.9 × 5.8 μm); 6-septate conidia: (70–)71–74 × 5.5–6.5(–7) μm (av. 72.1 × 6.4 μm); overall: (38.5–)48–68(–80.5) × 5–6(–7) μm (av. 58 × 5.7 μm). *Chlamydospores* commonly formed in the substrate mycelium and conidia, spherical to subspherical, 8.5–11(–12.5) μm diam, hyaline and smooth-walled. *Sexual morph* not observed.  

*Culture characteristics*: Colonies on PDA reaching 21–24 mm diam at 25 °C after 7 d. Surface salmon to buff, flat, membranous to velvety, with scant aerial mycelium and pionnotal, margin white and regular; reverse pale salmon with radial white to pale yellow patches. On OA, salmon to buff, flat, membranous and pionnotal, with regular margin; reverse pale pink to salmon.  

*Additional material examined*: **South Africa**, Western Cape Province, Swellendam, Bontebok National Park, from *Erica* sp. associated with *Dimerosporiopsis engleriana*, 24 Sep. 2018, A.R. Wood, culture CBS 146678 = CPC 37137.  

*Notes*: *Macroconia bulbipes* resolved as the closest phylogenetic relative to *Ma. gigas* and *Ma. papilionacearum* (Fig. 13). The former is, however, clearly distinguished morphologically by its smaller and less septate conidia (rarely up to 80.5 μm long and up to 6-septate *vs* longer than 100 μm and more than 10-septate in the latter two species). On the contrary, the asexual morph of *Ma. bulbipes* is closer to that of *Ma. leptosphaeriae* and *Ma. sphaeriae* (recognised as two distinct species in Gräfenhan *et al.* 2011). The conidia of *Ma. bulbipes*, however, differ by having commonly swollen basal cells.  

**Fig. 33:**
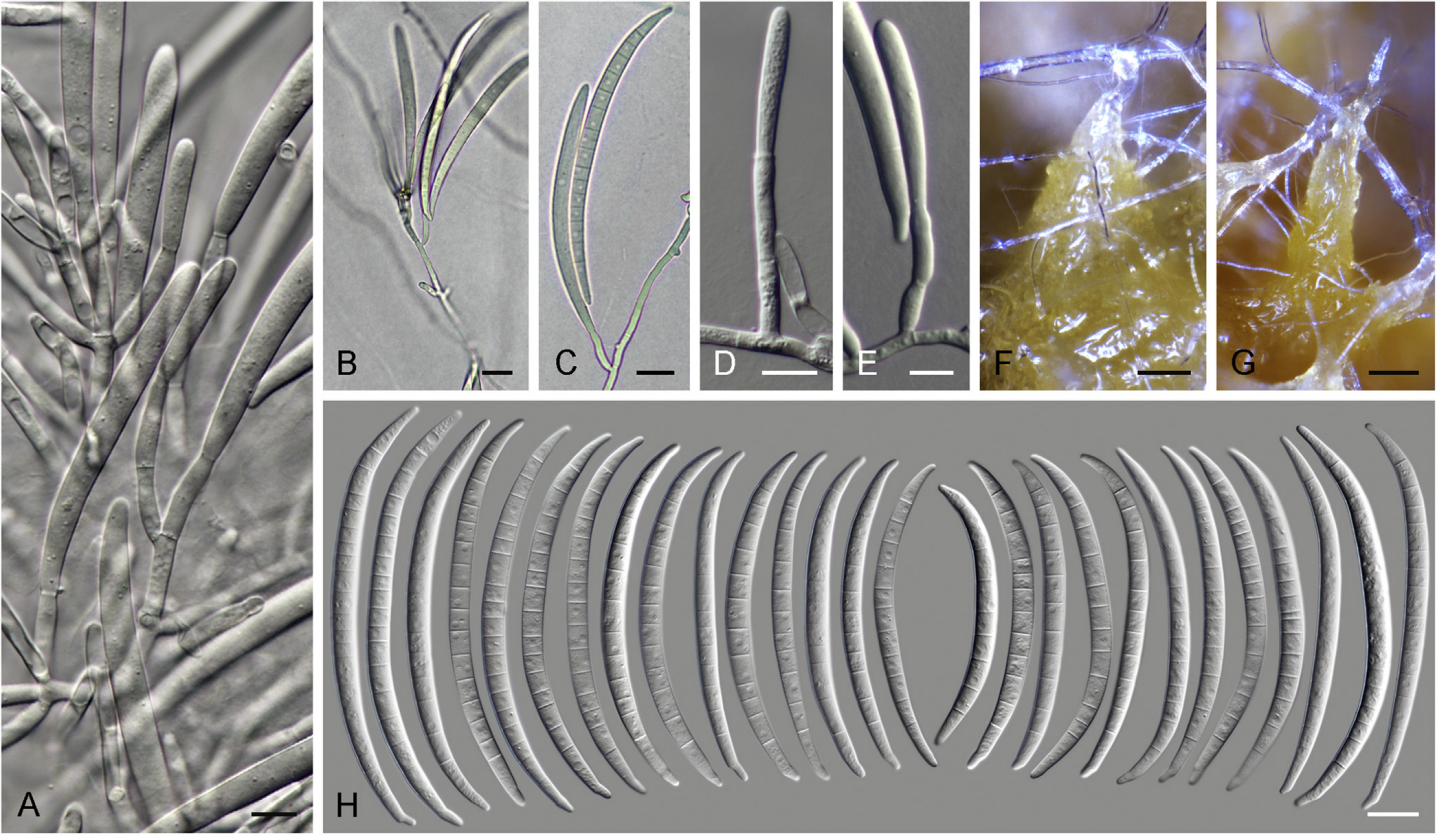
*Macroconia phlogioides* (CBS 146501). **A–C.** Conidiophores. **D, E.** Conidiogenous cells. **F, G.** Sporodochia formed on the agar surface. **H.** Macroconidia. Scale bars: B, C = 20 μm; F, G = 50 μm; all others = 10 μm.

***Macroconia phlogioides*** Sand.-Den. & Crous, ***sp. nov*.** MycoBank MB 838668. Fig. 33.

*Etymology:* From Greek *flóga*. Referring to the flame-like macroscopic semblance of the sporodochia.  

*Typus*: **South Africa**, Limpopo Province, Tzaneen, on leaf of *Encephalartos* sp., 2019, P.W. Crous (**holotype** CBS H-24665, culture ex-type CBS 146501 = CPC 35389).  

*Conidiophores* simple (aerial) or aggregated into sporodochia. *Aerial conidiophores* often borne laterally on hyphae and reduced to single conidiogenous cells, rarely 1-septate, hyaline, thin- and smooth-walled, 13–17 × 26–32 μm; *conidiogenous cells* monophialidic, subcylindrical to cylindrical, hyaline, (13–)16–24(–27.5) × (3.5–)4–5 μm conidiogenous opening rather wide, with inconspicuous periclinal thickening and no discernible apical collarettes. *Sporodochia* orange-pink to pink-brown coloured, often acquiring a flame-like, somewhat pointy macroscopic appearance and later merging into pionnotal crusts; *sporodochial conidiophores* irregularly or verticillately branched, 37.5–46 μm long, often bearing groups of 2–3 conidiogenous cells; *sporodochial conidiogenous cells* monophialidic, subcylindrical to subulate, (10–)18.5–26(–30) × (2.5–)3.5–5 μm with inconspicuous periclinal thickening, collarettes absent. *Microconidia* absent. *Macroconidia* robust, often with a nearly straight central portion and markedly curved and tapering towards both ends, apical cell conical to hooked, basal cell well-developed, foot-shaped, (1–)9–15(–19)-septate, predominantly 11-septate, hyaline, thick- and smooth-walled: 9-septate conidia: (106.5–)119.5–140(–143.5) × 7.5–8.5(–9) μm (av. 129 × 8 μm); 10-septate conidia: (116–)120–144.5(–164) × (7–)7.5–9 μm (av. 132 × 8 μm); 11-septate conidia: (122–)127–140(–153.5) × 7.5–9(–9.5) μm (av. 134 × 8.5 μm); 12-septate conidia: (119–)127.5–146.5(–153) × 7.5–9.5(–10) μm (av. 137 × 8.5 μm); 13-septate conidia: (128–)132–155(–172) × (7–)8–9(–10) μm (av. 143.5 × 8.5 μm); 14-septate conidia: (133.5–)136–157(–168) × 8–9.5 μm (av. 146.5 × 9 μm); 15-septate conidia: 147–163.5(–173.5) × 8.5–9.5(–10) μm (av. 155 × 9 μm); overall: (86–)123.5–150(–175) × (7–)8–9(–10) μm (av. 137 × 8.5 μm). *Chlamydospores* and *sexual morph* not observed.  

*Culture characteristics*: Colonies on PDA reaching 17–25 mm diam at 25 °C after 7 d. Surface salmon, buff to rosy buff, flat to slightly raised at centre, glabrous or with central patches of white, dense aerial mycelium; membranous to dusty with regular margin; reverse pale luteous to sulphur yellow, with salmon patches. On OA, salmon, flat, membranous, inconspicuously radially folded with regular margin; reverse pale pink to luteous with more intense salmon-coloured patches.  

*Additional material examined*: **South Africa**, Limpopo Province, Tzaneen, on leaf of *Encephalartos* sp., 2019, P.W. Crous, culture CBS 146500 = CPC 35388. **USA**, Arizona, Huachuca Mountains, Miller Canyon, on branch of *Quercus* sp. in stream, 1 Oct. 2008, T. Gräfenhan, culture CBS 125496.  

*Notes: Macroconia phlogioides* is morphologically related to *Ma. papilionacearum* and *Ma. gigas*. These three species are characterised by producing robust and large (often above 100 μm long) macroconidia. Unlike the above-mentioned species, however, conidia of *Ma. phlogioides* tend to present a higher number of septa (up to 19 *vs* up to 12 and 14, for *Ma. papilionacearum* and *Ma. gigas*, respectively), with rounder and less tapered apical cells, contrasting with the elongated conidial apices of *Ma. gigas*. Conidia of *Ma. phlogioides* also differ by having a more pronounced and continuous curvature compared to *Ma. gigas* and *Ma. papilionacearum*. These three species are clearly different phylogenetically, clustering in distant monophyletic lineages of the genus (Fig. 13).  

***Mariannaea*** G. Arnaud ex Samson, Stud. Mycol. 6: 74. 1974. Fig. 8.  

*Type species*: *Mariannaea elegans* (Corda) Samson, Stud. Mycol. 6: 75. 1974.

*Basionym*: *Penicillium elegans* Corda, Icon. Fung. 2: 17. 1838.

*Synonyms*: *Hormodendron elegans* (Corda) Bonorden, Handb. Allg. Mykol.: 76. 1851.

*Spicaria elegans* (Corda) Harz., Bull. Soc. Imp. Naturalistes Moscou 44: 238. 1871.

*Paecilomyces elegans* (Corda) Mason & Hughes, Mycol. Pap. 45: 27. 1951.  

*Ascomata* perithecial, solitary, non-stromatic or on inconspicuous stroma, superficial, globose with flat apex, not collapsing or laterally pinched when dry, pale yellow, orange or brown, not reacting in KOH, smooth-walled to slightly rugose, lacking hairs or appendages. *Asci* cylindrical to narrowly clavate, 8-spored sometimes with inconspicuous apical ring, uniseriate to apically biseriate. *Ascospores* 1-septate, hyaline, smooth-walled to spinulose. *Conidiophores* verticillate to penicillate, hyaline, with phialides arising directly from the stipe or forming whorls of metulae on lower parts of stipe; stipe hyaline, becoming yellow-brown at the base. *Conidiogenous cells* monophialidic, ampulliform, hyaline, usually with obvious periclinal thickening and inconspicuous collarettes. *Conidia* limoniform, aseptate, hyaline, in chains that collapse to form slimy heads. *Chlamydospores* globose to ellipsoidal, hyaline, formed in intercalary chains.

[Description adapted from Samson (1974), Gräfenhan *et al.* (2011) and Lombard *et al.* (2015)].  

*Diagnostic features*: Pale yellow, orange to brown perithecia with flattened apex producing cylindrical to narrowly clavate asci bearing 1-septate ascospores and asexual morphs characterised by verticillate to penicillate conidiophores producing small, aseptate, limoniform conidia in chains that collapse into slimy heads.  

**Fig. 34:**
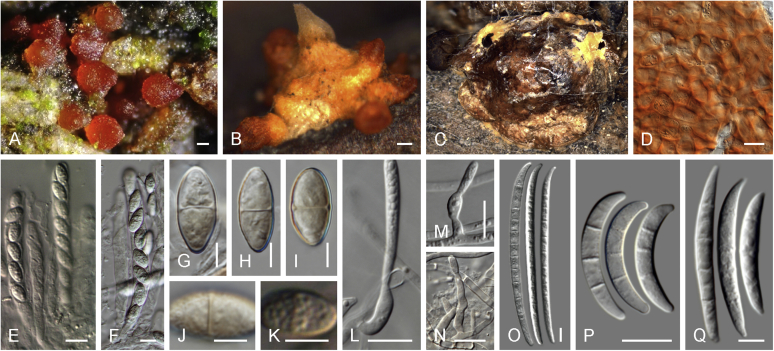
*Microcera* spp. **A–C.** Ascomata on natural substrate. **D.** Surface view of perithecial wall in 2 % KOH. **E, F.** Asci. **G–K.** Ascospores (J, K. Surface view). **L–N.** Conidiophores and conidiogenous cells. **O–Q.** Macroconidia. A. *Microcera auranticola* (photo N. Aplin). B, O. *Microcera coccophila* [adapted from Gräfenhan *et al.* (2011)]. C. *Microcera larvarum* [adapted from Gräfenhan *et al.* (2011)]. D, F–J. *Microcera coccophila* (K(M) 165807). E, K. *Microcera larvarum* (photo P. Cannon). L, M, Q. *Microcera rubra* (CBS 638.76). N, P. *Microcera larvarum* (CBS 169.30). Scale bars: A, B = 100 μm; G–K = 5 μm; all others = 10 μm.

***Microcera*** Desm., Ann. Sci. Nat. Bot., sér. 3, 10: 359. 1848. Figs 8, 34.

*Synonym*: *Pseudomicrocera* Petch, Trans. Brit. Mycol. Soc. 7: 164. 1921.  

*Type species*: *Microcera coccophila* Desm., Ann. Sci. Nat. Bot., sér. 3, 10: 359. 1848.

(See *F. coccophilum* in List section for synonyms)  

*Ascomata* perithecial, solitary or gregarious, with stroma and/or byssus covering host, globose, with a blunt papilla, orange to dark red, turning dark red or violet in KOH, finely roughened. *Asci* cylindrical to narrowly clavate, with an apical ring, 8-spored. *Ascospores* hyaline to pale yellow-brown, 1(–3)-septate, smooth, sometimes becoming tuberculate when mature. *Conidiophores* as lateral phialides on somatic hyphae, becoming monochasial, verticillate to penicillate, hyaline, forming discrete sporodochia or synnemata on the host. *Conidiogenous cells* monophialidic, cylindrical to subulate to subclavate, hyaline. *Macroconidia* pale, orange, pink or bright red in mass, subcylindrical, moderately or conspicuously curved, apical cell often slightly or conspicuously hooked, basal cell papillate to well-developed, foot-shaped, (0–)3–5(–12)-septate, hyaline.

[Description adapted from Gräfenhan *et al.* (2011)].  

*Diagnostic features*: Orange to dark red perithecia with a blunt papilla producing cylindrical to narrowly clavate asci bearing yellow-brown, 1(–3)-septate ascospores; asexual morphs characterised by verticillate to penicillate conidiophores producing small macroconidia; species typically associated with scale insects.  

**Fig. 35:**
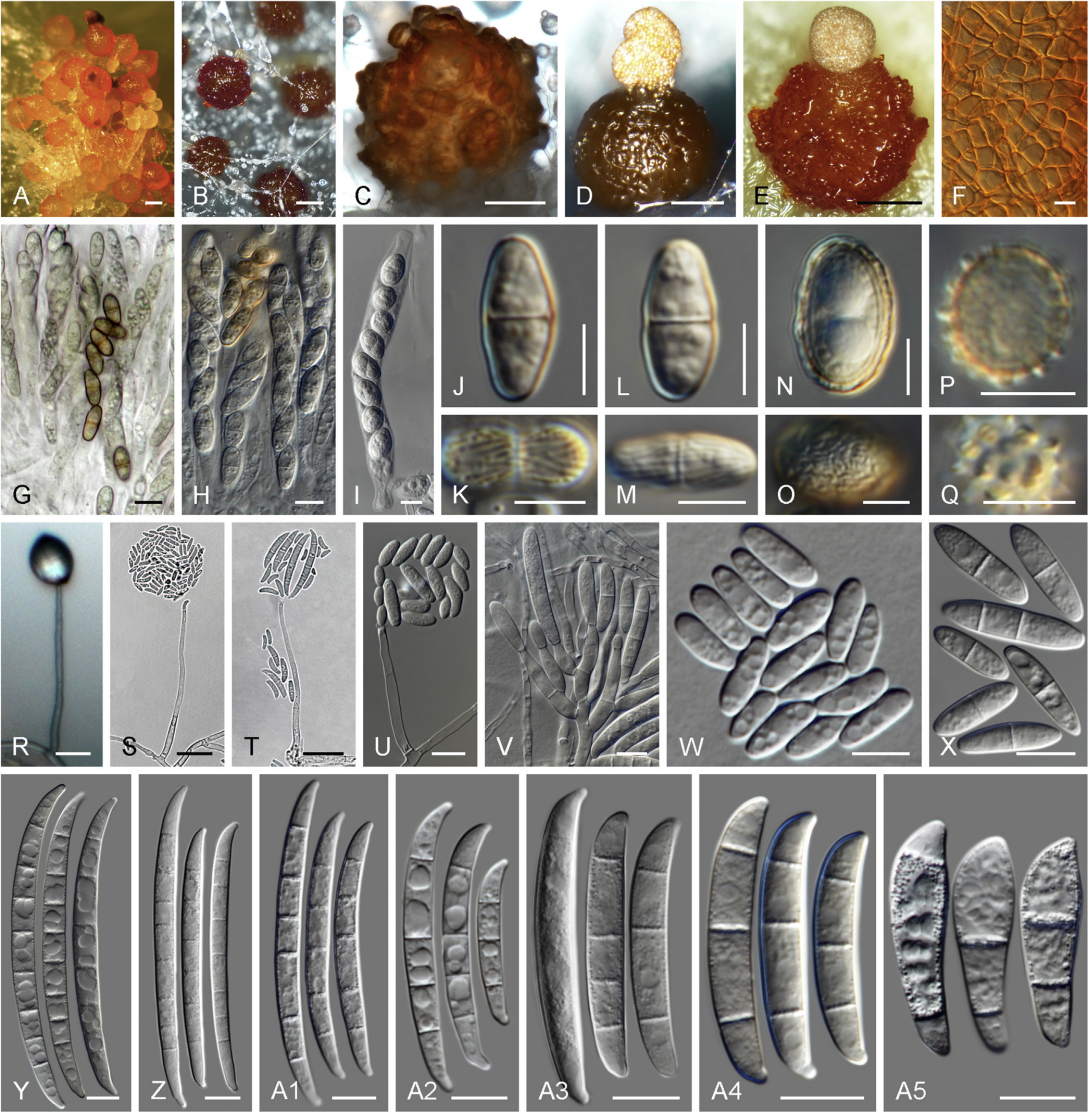
*Neocosmospora* spp. **A–E.** Ascomata on culture. **F.** Surface view of perithecial wall in 2 % KOH. **G–I.** Asci. **J–Q.** Ascospores (K, M, O, Q. Surface view). **R–U.** Aerial conidiophores. **V.** Sporodochial conidiophores. **W, X.** Microconidia. **Y–A5.** Macroconidia. A, I, N, O. *Neocosmospora vasinfecta* (CBS 446.93). B. *Neocosmospora* sp. (CPC 34617). C, S, W, A1. *Neocosmospora elegans* (CBS 144396). D. *Neocosmospora vasinfecta* (CBS 863.70). E. *Neocosmospora bataticola* (CBS 144398). F, L, M. *Neocosmospora ipomoeae* (CBS 833.97). G. *Neocosmospora robiniae* (CBS 119601). H, J, K. *Neocosmospora diminuta* (CBS 144390). O, Q. *Neocosmospora spinulosa* (CBS H-5443). R, V, A3. *Neocosmospora solani* (CBS 140079). T. *Neocosmospora bataticola* (CBS 144398). U. *Neocosmospora suttoniana* (CBS 143214). X. *Neocosmospora tonkinensis* (CBS 115.40). Y. *Neocosmospora longissima* (CBS 126407). Z. *Neocosmospora mori* (CBS 145467). A2. *Neocosmospora pseudoradicicola* (CBS 145472). A4. *Neocosmospora keratoplastica* (CBS 490.63). A5. *Neocosmospora oligoseptata* (CBS 143241). [A, C, S, T, W, Y, Z, A1, A2. Adapted from adapted from Sandoval-Denis *et al.* (2019). R, V, A3, Adapted from Crous *et al.* (2019a). U, X, A4. Adapted from Sandoval-Denis & Crous (2018)]. Scale bars: A, B = 200 μm; C–E 100 μm; R–T = 20 μm; J–Q, W, X = 5 μm; all others = 10 μm.

***Neocosmospora*** E.F. Sm., Bull. U.S.D.A. 17: 45. 1899. Figs 8, 35.

*Type species*: *Neocosmospora vasinfecta* E.F. Sm., Bull. U.S.D.A. 17: 45. 1899.

(See *F. neocosmosporiellum* in List section for synonyms)  

*Ascomata* perithecial, solitary or gregarious, non-stromatic or with reduced basal stroma, superficial, globose to pyriform, not collapsing when dry, orange-brown to bright red, darkening or becoming purple in KOH, papillate or with short ostiolar neck, commonly tuberculate, rarely smooth-walled, lacking hairs or appendages. *Ascomatal wall* of two regions: outer region of thick-walled, pigmented cells forming a *textura angularis*; inner region of elongate, hyaline, thin-walled cells, becoming thinner towards the centrum. *Asci* saccate, clavate to cylindrical, unitunicate, apex simple, rounded or flattened, 8-spored, uniseriate to irregularly biseriate. *Ascospores* globose to ellipsoidal, with or without slightly truncate ends, typically 1-septate, hyaline when young becoming yellow golden-brown at maturity, thick-walled, longitudinally striate; ascospores in some species 0-septate, cerebriform or spinulose. *Conidiophores* mononematous (aerial) or grouped on sporodochia, or somewhat erect, loosely branched sporodochial pustules. *Aerial conidiophores* simple, sparsely to highly branched; aerial *conidiogenous cells* monophialidic, elongate subulate to subcylindrical. *Aerial conidia* hyaline, smooth- and thick-walled, of two types: *microconidia* subglobose, ellipsoidal to somewhat clavate, 0–2(–4)-septate, borne in false heads on phialides; *macroconidia* falcate, slightly to strongly curved dorsiventrally, 1-septate to multiseptate, with blunt to hooked to slightly pointed apical cell and papillate to well-developed foot-shaped basal cell. *Sporodochia* cream, pale luteous, light green, olivaceous, bluish, hazel to greyish sepia; s*porodochial conidiophores* verticillately or sympodially branched or sparingly branched and densely packed, consisting of short, smooth- and thin-walled stipes bearing apical whorl of 2–4 monophialides; *sporodochial conidiogenous cells* monophialidic, doliiform, short subcylindrical to subulate, smooth- and thin-walled, periclinal thickening and collarettes inconspicuous or absent. *Sporodochial macroconidia* falcate, smooth- and thick-walled, straight or curved with parallel walls to unequally curved, in some species clavate and asymmetrical, tapering towards both ends, with a pointed to blunt to hooked apical cell and papillate to well-developed foot-shaped basal cell. *Chlamydospores* globose to subglobose to ovoid to obovoid, hyaline to pale golden brown, smooth-walled to slightly verrucose, terminal or intercalary, solitary or in pairs or forming chains or aggregating in some species to form buff, olive aeruginous or bluish microsclerotia.

[Description adapted from Rossman *et al.* (1999) and Sandoval-Denis *et al.* (2019)].  

*Diagnostic features*: Orange-brown to frequently bright, blood red warted perithecia with papillate or short ostiolar neck producing saccate, clavate to cylindrical asci bearing globose to ellipsoidal, 0- or 1-septate, longitudinally striate, cerebriform or spinulose ascospores and asexual morphs producing micro- and macroconidia on elongate subulate to subcylindrical aerial conidiophores with monophialides or only macroconidia in sporodochia. Chlamydospores formed in hyphae, rarely observed in macroconidia.  

**Fig. 36:**
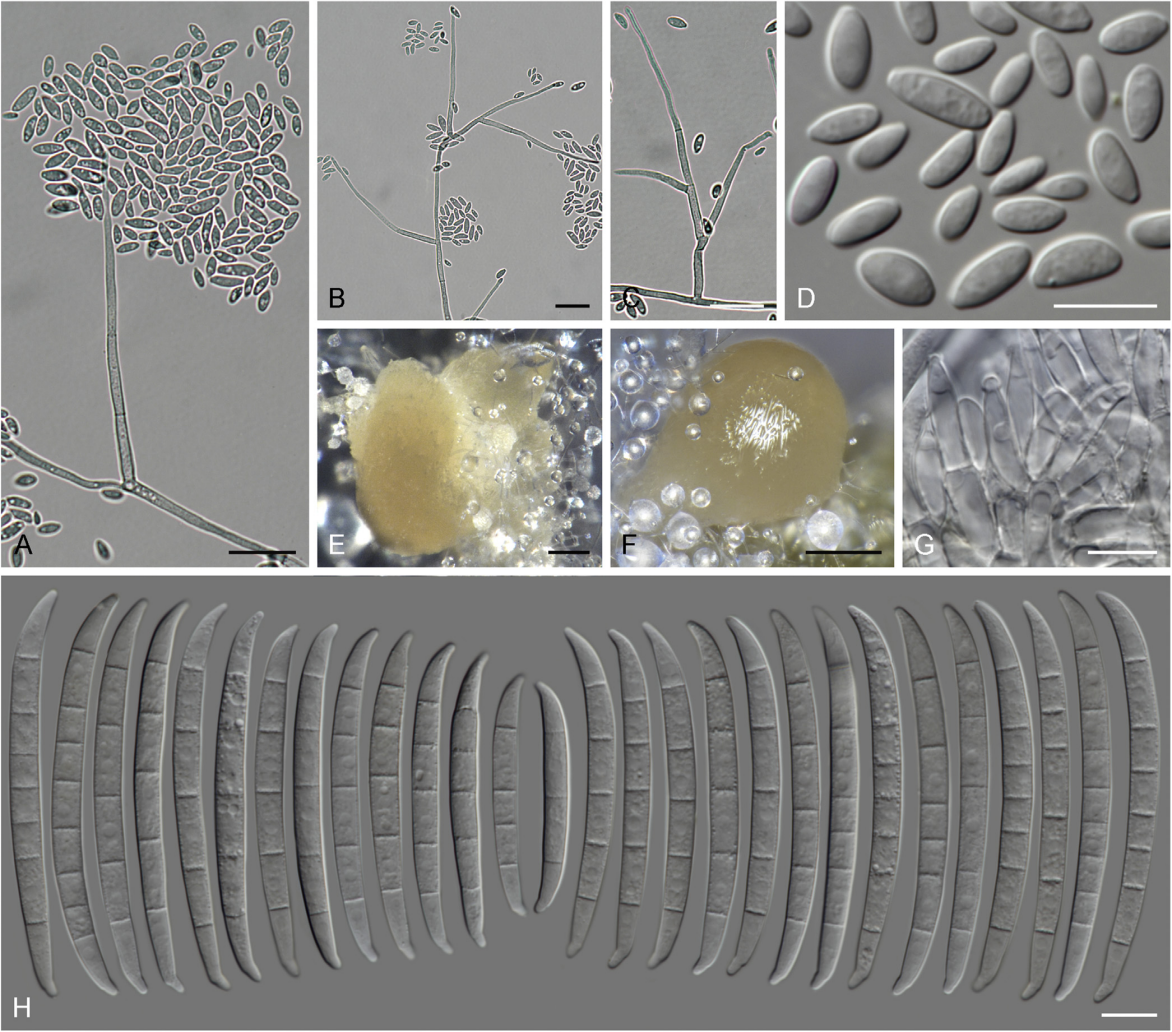
*Neocosmospora epipeda* (CBS 146524). **A–C.** Aerial conidiophores and conidiogenous cells. **D.** Microconidia. **E, F.** Sporodochia formed on the surface of carnation leaves. **G.** Sporodochial conidiophores and conidiogenous cells. **H.** Macroconidia. Scale bars: A–C = 20 μm; E, F = 200 μm; D, G, H = 10 μm.

***Neocosmospora epipeda*** Quaedvl. & Sand.-Den., ***sp. nov*.** MycoBank MB 838669. Fig. 36.

*Etymology*: From the Greek *επίπεδα*, flat; referring to the microconidia of this species commonly being flattened on one side.  

*Typus*: **Netherlands**, from *Bouvardia* sp. imported from Uganda, 2019, W. Quaedvlieg (**holotype** CBS H-24666, culture ex-type CBS 146523 = CPC 38310).  

*Conidiophores* borne on the agar substrate and aerial mycelium, 78–230 μm tall, unbranched or more commonly sympodially branched at various levels, bearing terminal single phialides; aerial *conidiogenous cells* monophialidic, subulate, subcylindrical to acicular, smooth- and thin-walled, 27.5–62 × 2–3.5 μm, short apical collarettes and periclinal thickening inconspicuous or absent. *Aerial conidia* microconidial, arranged in false heads on phialide tips, hyaline, broadly ellipsoidal, ellipsoidal to short clavate, commonly asymmetrical with a somewhat flattened side, smooth- and thin-walled, aseptate, (4.5–)6–10(–13.5) × (2–)3–5 μm (av. 8 × 3.5 μm). *Sporodochia* pale luteous to orange, formed abundantly on the surface of carnation leaves; *sporodochial conidiophores* laterally and irregularly branched bearing apical groups of 2–3 monophialides; *sporodochial conidiogenous cells* monophialidic, subulate to subcylindrical, 11–19.5 × 3–4.5 μm, smooth and thin-walled, with short, non-flared collarettes and inconspicuous or absent periclinal thickening. *Sporodochial conidia* falcate, almost straight to slightly curved dorsoventrally, broadest near the half portion or the upper third, tapering towards both ends, with a blunt to somewhat pointy and slightly curved apical cell and an often well-developed foot-shaped basal cell, (3–)4–7(–8)-septate, predominantly 5-septate, hyaline, smooth- and thick-walled; 3-septate conidia: 42.5 × 4.4 μm; 4-septate conidia: (41.5–)44–58(–60) × 4–5 μm (av. 51.1 × 4.4 μm); 5-septate conidia: (53.5–)59–69.5(–76) × 4–6 μm (av. 64.3 × 5 μm); 6-septate conidia: 68–75.5(–79.5) × 4.5–6 μm (av. 71.7 × 5.3 μm); 7-septate conidia: (68–)69–74.5(–77) × 5–6 μm (av. 71.7 × 5.5 μm); 8-septate conidia: 74–75.5 × 5–6 μm (av. 74.7 × 5.3 μm); overall: (42.5–)59–73.5(–79.5) × (4–)5–6 μm (av. 66.3 × 5.1 μm). *Chlamydospores* and *sexual morph* not observed.  

*Culture characteristics*: Colonies on PDA reaching 38–53 mm diam at 25 °C after 7 d. Surface white to sulphur yellow with scarce pale ochreous to pale rust patches, flat to slightly raised with abundant white aerial mycelium, cottony to woolly, margin filiform; reverse pale luteous to sulphur yellow, pale apricot to pale rust at centre. On OA, pale luteous, flat, membranous with entire margin; reverse pale luteous.  

*Additional material examined*: **Netherlands**, from *Bouvardia* sp. imported from Uganda, 2019, W. Quaedvlieg, culture CBS 146524 = CPC 38311.  

*Notes*: The name *N. epipeda* is coined here for a novel phylogenetic lineage discovered on a *Bouvardia* sp. imported from Uganda. The new species clusters as the closest phylogenetic relative of *N. catenata* (Fig. 14), an opportunistic animal-pathogenic species characterised by abundant production of catenate to clustered, pigmented chlamydospores, and by the absence (as far as known) of macroconidia (O'Donnell *et al.* 2016, Sandoval-Denis & Crous 2018). These characters form the most notable differences with respect to *N. epipeda*. Additionally, *N. epipeda* can be differentiated from *N. catenata* by its less septate and shorter microconidia (aseptate and up to 13.5 μm *vs* up to 1-septate and 11 μm in *N. catenata*). Other species producing macroconidia of similar size and shape to those of *N. epipeda* include *N. quercicola*, *N. robusta*, and *N. silvicola*; however, the three latter species are genetically distant in that they belong to monophyletic lineages of clade 3 (*N. quercicola* and *N. silvicola*) and clade 1 (*N. robusta*) of *Neocosmospora sensu* O'Donnell *et al.* (2008a). *Neocosmospora epipeda* can be distinguished morphologically from *N. robusta* by the production of microconidia with absence of aerial macroconidia in the former species. Morphological differentiation of the novel species from *N. quercicola* and *N. silvicola* is difficult because of overlapping features; nevertheless, subtle differences exist in the size and morphology of the microconidia (aseptate in *N*. *epipeda vs* up to 1-septate in both *N. quercicola* and *N. silvicola*, being also reniform and longer in the latter species) and sporodochial colour (pale luteous to orange in *N. epipeda vs* greenish to citrine in *N. quercicola* and *N. silvicola*, respectively).  

**Fig. 37:**
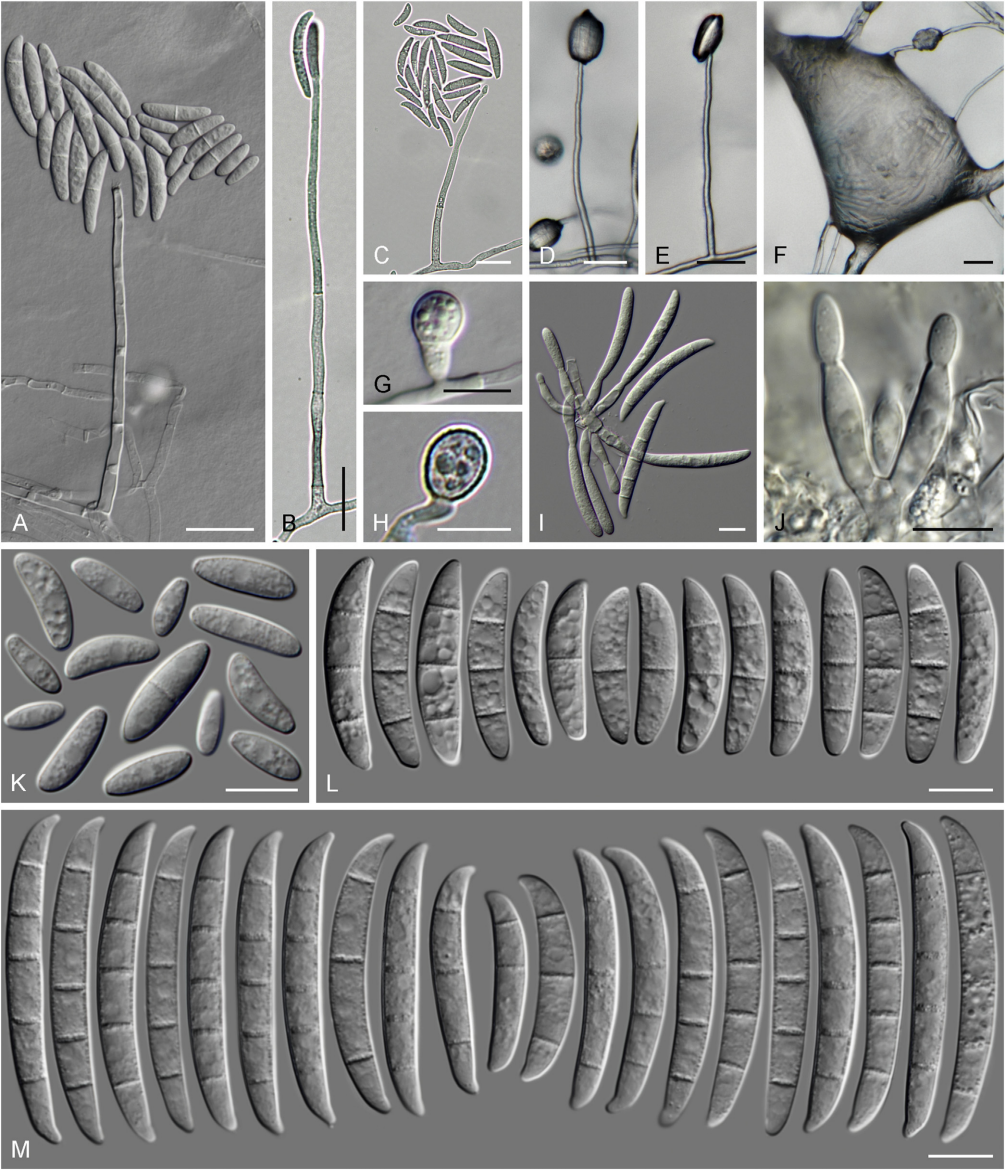
*Neocosmospora merkxiana* (CBS 146525). **A–E.** Aerial conidiophores and conidiogenous cells. **F.** Sporodochium on aerial mycelium. **G, H.** Chlamydospores. **I, J.** Sporodochial conidiophores and conidiogenous cells. **K.** Microconidia. **L.** Aerial macroconidia. **M.** Sporodochial macroconidia. Scale bars: A, E = 100 μm; C = 20 μm; all others = 10 μm.

***Neocosmospora merkxiana*** Quaedvl. & Sand.-Den., ***sp. nov*.** MycoBank MB 838670. Fig. 37.

*Etymology:* Named after Trix Merkx, senior technician at the Westerdijk Fungal Biodiversity Institute, in recognition of her career as the foremost link in strain handling between the research groups and the culture collection.  

*Typus*: **Netherlands**, from *Chrysanthemum* sp. imported from Uganda, unknown date, W. Quaedvlieg (**holotype** CBS H-24669, culture ex-type CBS 146525 = CPC 38701).  

*Conidiophores* borne on the agar substrate and aerial mycelium, 99–205 μm tall, unbranched or rarely laterally branched, bearing terminal single phialides; aerial *conidiogenous cells* monophialidic, subulate to subcylindrical, smooth- and thin-walled, 41.5–77 × 2.5–4.5 μm, with short and flared apical collarettes and inconspicuous periclinal thickening. *Aerial conidia* of two types: *microconidia* oval to broadly ellipsoidal, straight to slightly curved and asymmetrical, smooth- and thin-walled, 0(–1)-aseptate, (8.5–)9–15.5(–18.5) × 3–5.5 μm (av. 12.4 × 4.3 ìm), arranged in false heads on phialide tips; *macroconidia* falcate to navicular, smooth- and thin-walled, almost straight to slightly dorsiventrally curved, ventral face almost straight, with a blunt apical cell, basal cell obtuse to poorly-developed, foot-shaped, 1–3-septate, predominantly 1-septate, 1-septate conidia: (17.5–)20.5–27(–30.5) × (4.5–)5–6.5(–7.5) μm (av. 23.8 × 5.8 μm); 2-septate conidia: (25.5–)27–30(–32) × 5.5–7 μm (av. 28.4 × 6 μm); 3-septate conidia: (27–)28.5–33.5(–35.5) × 5–7.5 μm (av. 31.1 × 6.3 μm); overall: (17.5–)22–31(–35.5) × (4.5–)5–6.5(–7.5) μm (av. 26.4 × 6 μm), arranged in false heads at the tip of monophialides and produced intermixed with microconidia. *Sporodochia* pale luteous, formed on aerial and substrate mycelium, uncommon on carnation leaves. *Sporodochial conidiophores* laterally and irregularly branched bearing apical groups of 2–3 monophialides; *sporodochial conidiogenous cells* monophialidic, doliiform, subulate to subcylindrical, (13.5–)15–21.5(–27) × 2.5–5.5 μm, smooth and thin-walled, lacking apical collarettes and with inconspicuous periclinal thickening. *Sporodochial macroconidia* falcate, straight to slightly dorsiventrally curved, broadest at the half portion and tapering towards both ends, apical cell blunt and slightly curved, basal cell poorly- to well-developed, foot-shaped, (1–)3–5-septate, predominantly 4-septate, hyaline, smooth- and thick-walled; 1-septate conidia: (23.5–)24.5–28.5 × 5–6.5 μm (av. 25.8 × 5.6 μm); 2-septate conidia: 27–29 × 5.5–6.5 μm (av. 28 × 6 μm); 3-septate conidia: (29–)35–45 × (4.5–)5–6 μm (av. 40.1 × 5.3 μm); 4-septate conidia: (41–)44.5–49.5(–51.5) × 4.5–6.5 μm (av. 47 × 5.6 μm); 5-septate conidia: (42–)45.5–51.5(–52.5) × 5–6 μm (av. 48.5 × 5.6 μm); overall: (24.5–)39–51.5(–52.5) × 4.5–6(–6.5) μm (av. 45.2 × 5.6 μm). *Chlamydospores* obovoidal, subspherical to spherical, hyaline to pale yellow brown, smooth-walled to slightly roughened, thick-walled, 5–13.5 μm, single or in chains, terminal, intercalary or produced on short lateral stipes.  

*Culture characteristics*: Colonies on PDA reaching 45–56 mm diam at 25 °C after 7 d. Surface pale luteus to sulphur yellow, becoming buff to honey, flat with abundant aerial mycelium, cottony to woolly with entire to filiform margin; reverse luteous to buff, pale scarlet to bay at centre. On OA pale luteous to peach with sparse white cushions of aerial mycelium, flat, velvety to cottony; reverse pale luteous, peach to pale scarlet.  

*Additional material examined*: **Netherlands**, from *Chrysanthemum* sp. imported from Uganda, unknown date, W. Quaedvlieg, culture CBS 146526 = CPC 38702.  

*Notes*: *Neocosmospora merkxiana* represents the phylogenetic species formerly known as “FSSC 41”, one of the few previously known clades lacking a Latin binomial, originally reported as an agent of collar rot on *Passiflora edulis* *f. flavicarpa* in Brazil (Cardoso 2015, Sandoval-Denis *et al.* 2019). Here, this species is reported causing collar and stem rot symptoms in *Chrysanthemum* imported from Uganda.

In the phylogenetic analysis (Fig. 14), *N. merkxiana* resolved as the most basal taxon within a lineage containing the morphologically similar species *N. ipomoeae*, *N. martii*, and *N. noneumartii*, all characterised by producing both aerial microconidia and macroconidia, in addition to relatively long sporodochial conidia. Differing from the aforementioned species, *N. merkxiana* can be differentiated by its fewer septate and shorter aerial and sporodochial macroconidia formed on pale luteous sporodochia, and its pale luteous colonies on PDA, thus contrasting with the greenish sporodochial colouration observed in both *N. ipomoeae* and *N. noneumartii,* and the red pigmentation on PDA typical of *N. martii*. Sexual morphs were not observed in the isolates studied here; however, this lineage was reported as heterothallic, and fertile perithecial ascomata have been induced *in vitro* (Cardoso 2015), characterised by ascomata measuring 230–355 × 175–290 μm, 57.5–75 × 5 μm asci producing 1-septate, 10–12.5 × 5 μm ascospores.  

**Fig. 38:**
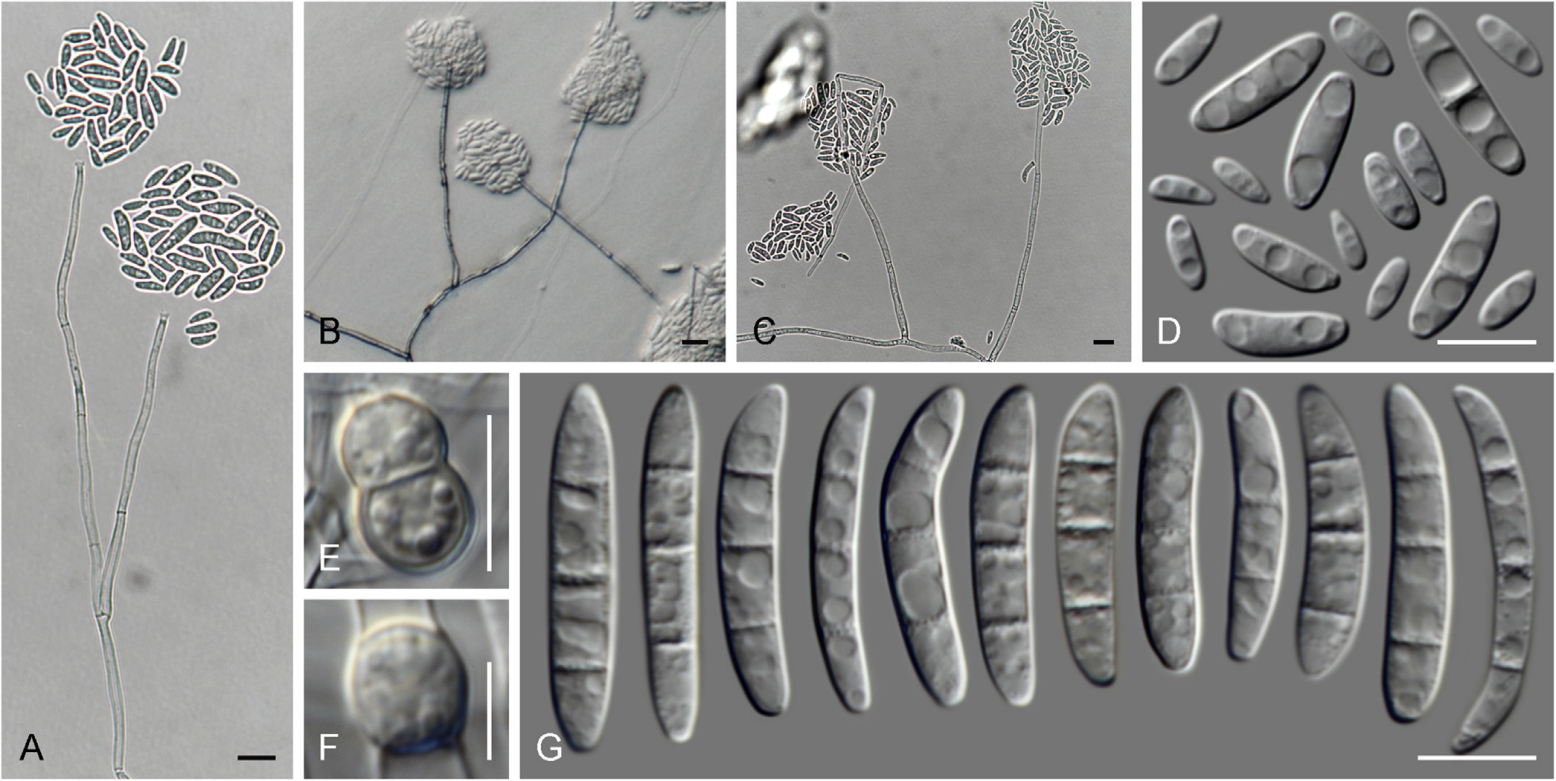
*Neocosmospora neerlandica* (CBS 232.34). **A–C.** Conidiophores. **D.** Microconidia. **E, F.** Chlamydospores. **G.** Macroconidia. Scale bars: F = 5 μm; all others = 10 μm.

***Neocosmospora neerlandica*** Crous & Sand.-Den., ***sp. nov*.** MycoBank MB 838671. Fig. 38.

*Etymology*: Named after the country where the type was isolated, the Netherlands.  

*Typus*: **Netherlands**, Zeeland Province, Zuid-Beveland, near Wolphaartsdijk, from *Pisum sativum*, unknown date, J.C. Went (**holotype** CBS H-24667, culture ex-type CBS 232.34).  

*Conidiophores* borne on agar substrate and aerial mycelium up to 290 μm tall, unbranched or irregularly laterally branched, bearing terminal single monophialides, commonly proliferating percurrently; *aerial conidiogenous cells* monophialidic, subulate to subcylindrical, commonly extended percurrently, smooth- and thin-walled, 21–87 × 1.5–3.5 μm, with short and flared apical collarettes and rather evident periclinal thickening. *Aerial conidia* of two types: *microconidia* oval to broadly ellipsoidal, smooth- and thin-walled, 0- or 1-septate, (5.5–)8–14(–30) × (2–)3–4.5(–5.5) μm (av. 11 × 3.8 μm), arranged in false heads on phialide tips; *macroconidia* fusiform to falcate, smooth- and thick-walled, straight to slightly curved, with a blunt apical cell, basal cell often flattened to obtuse, (1–)2–3-septate, predominantly 3-septate, 1-septate conidia: 22.5–26 × 4.5–6 μm (av. 24.4 × 5.1 μm); 2-septate conidia: (22.5–)23.5–32 × 3.5–5 μm (av. 27 × 4.3 μm); 3-septate conidia: (24–)25–32.5(–38.5) × (3.5–)4.5–5.5(–6) μm (av. 28.7 × 4.8 μm); overall: (22.5–)24–31.5(–38.5) × (3.5–)4.5–6 μm (av. 27.7 × 4.8 μm), arranged in false heads at the tip of monophialides and produced intermixed with microconidia. *Chlamydospores* subspherical to spherical, pale golden brown, smooth- and thick-walled, 6–8 μm, single or in pairs, terminal or more often formed intercalary on hyphae. *Sexual morph* and *sporodochia* unknown.  

*Culture characteristics*: Colonies on PDA reaching 42–51 mm diam at 25 °C after 7 d. Surface white to pale luteous, flat with abundant dense aerial mycelium, velvety to cottony, margin regular and filiform; reverse pale luteous to sulphur yellow. On OA white to pale luteous, flat to slightly raised, velvety to cottony, margin regular and filiform; reverse pale luteous.  

*Notes*: The type of *N. neerlandica* was originally deposited as *N. pisi*, an important root pathogen of *Pisum sativum*. Besides sharing the same host association, both species are genetically related, but cluster in distinct phylogenetic lineages and have a different morphology. Although *N. pisi* produces typical wedge-shaped, larger macroconidia (up to 46 um long) on abundant sporodochia (Šišić *et al.* 2018b), *N. neerlandica* is characterised by short falcate macroconidia (up to 38.5 um long) produced on aerial conidiophores, while sporodochia are not formed. The latter features relate *N. neerlandica* to *N. diminuta*, a phylogenetically distant species that produces the shortest falcate conidia known in *Neocosmospora* (Sandoval-Denis *et al.* 2019). Nevertheless, *N. diminuta* is a homothallic species that conspicuously produces sexual structures, while a sexual morph is not known for *N. neerlandica*. Additionally, macroconidia of *N. neerlandica* differ from those of *N. diminuta* by having less curved apices and poorly developed or non foot-shaped basal cells.  

**Fig. 39:**
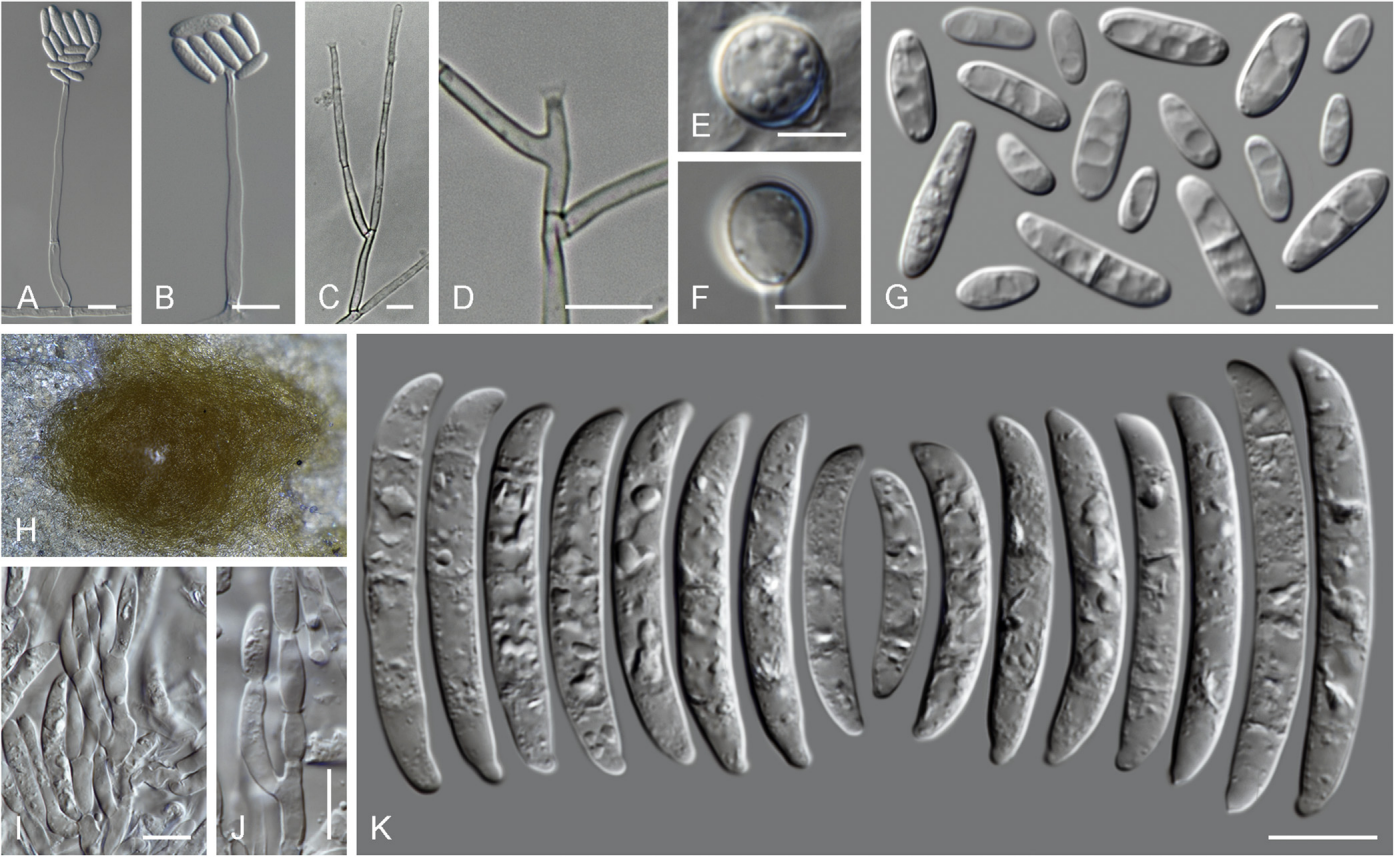
*Neocosmospora nelsonii* (CBS 309.75). **A–D.** Conidiophores and conidiogenous cells. **E, F.** Chlamydospores. **G.** Microconidia. **H.** Sporodochium. **I, J.** Sporodochial conidiophores and conidiogenous cells. **K.** Macroconidia. Scale bars: E, F = 5 μm; all others = 10 μm.

***Neocosmospora nelsonii*** Crous & Sand.-Den., ***sp. nov*.** MycoBank MB 838672. Fig. 39.

*Etymology*: In honour of Paul E. Nelson, prominent *Fusarium* researcher and collector of the ex-type strain of this species.  

*Typus*: **Unknown country**, from *Pisum sativum*, unknown date, P.E. Nelson (**holotype** CBS H-12719, culture ex-type CBS 309.75).  

*Conidiophores* borne on agar substrate and aerial mycelium, 59–330 μm tall, often simple and reduced to solitary phialides borne laterally from hyphae, or laterally irregularly and sympodially branching one or two times, bearing terminal single phialides; *aerial conidiogenous cells* monophialidic, subulate to subcylindrical, smooth- and thin-walled, 21–57.5 × 2–5 μm, flared apical collarettes and periclinal thickening present. *Aerial microconidia* arranged in false heads on phialide tips, hyaline, broadly ellipsoidal, obovate to broadly clavate, smooth- and thin-walled, 0(–1)-septate, (5–)7–13(–17) × 2.5–5 μm (av. 10.1 × 3.7 μm). *Sporodochia* (from holotype specimen) pale citrine to olivaceous; *sporodochial conidiophores* copiously branched, laterally, verticillate and irregularly, bearing apical groups of 2–3 monophialides and lateral solitary phialides; *sporodochial conidiogenous cells* monophialidic, doliiform, subulate to subcylindrical, 6–21.5 × 3–4.5 μm, smooth and thin-walled, with short, conspicuously flared collarettes and conspicuous periclinal thickening, profusely proliferating percurrently. *Sporodochial macroconidia* falcate, gently and regularly curved dorsoventrally or with an almost straight ventral line, broadest at the middle portion, apical cell blunt and slightly hooked, basal cell papillate to well-developed, foot-shaped, 1–3(–4)-septate, predominantly 3-septate, hyaline, smooth- and thick-walled; 1-septate conidia: (17.5–)19–26(–29.5) × 4–5 μm (av. 22.4 × 4.4 μm); 2-septate conidia: (26–)27–34 × 3.5–5.5 μm (av. 30 × 4.7 μm); 3-septate conidia: (25.5–)30.5–38(–42) × 4–5.5 μm (av. 34.3 × 4.8 μm); 4-septate conidia: 38.5–43.5 × 4.5–5.5 μm (av. 40.7 × 5.0 μm); overall: (17.5–)27–38(–43.5) × (3.5–)4–5.5 μm (av. 32.5 × 4.7 μm). *Chlamydospores* subspherical to spherical, pale golden brown, smooth- and thick-walled, 4–11.5 μm, formed singly and terminally on hyphae. *Sexual morph* not observed.  

*Culture characteristics*: Colonies on PDA reaching 35–49 mm diam at 25 °C after 7 d. Surface pale luteous, pale saffron to sulphur yellow, flat with abundant dense and short aerial mycelium, velvety to woolly, margin filiform; reverse sulphur yellow. On OA pale luteous, flat, membranous to dusty with filiform margin; reverse pale luteous.  

*Notes*: The ex-type of *N. nelsonii*, originally determined as “*F.” solani*, currently presents a very simple microconidial morphology with a rather acremonioid touch given its slender, generally simple conidiophores and mostly aseptate microconidia. Hence, there are no clear phenotypic characters to differentiate the species. Failed attempts to induce formation of sporodochia indicate that the ex-type strain may have lost the ability to produce macroconidia *in vitro*. The holotype material, is, however, a dried subculture from the type strain dated from 1982. It still contains a large amount of well-preserved sporodochia and sporodochial conidia, which we describe here. These macroconidia are comparable in size to those observed in closely related species such as *N. brevis*, *N. pisi*, and *N. neerlandica.* However, macroconidia in *N. brevis* and *N. neerlandica* are produced only in the aerial mycelium, while *N. nelsonii* produces only a single type of aerial conidia (microconidia), which also differ from those observed in the aforementioned species by their reduced size. In addition, sporodochial conidia in *N. nelsonii* are shorter and stout, with shorter and rounder apices compared to those of *N. pisi*.  

**Fig. 40:**
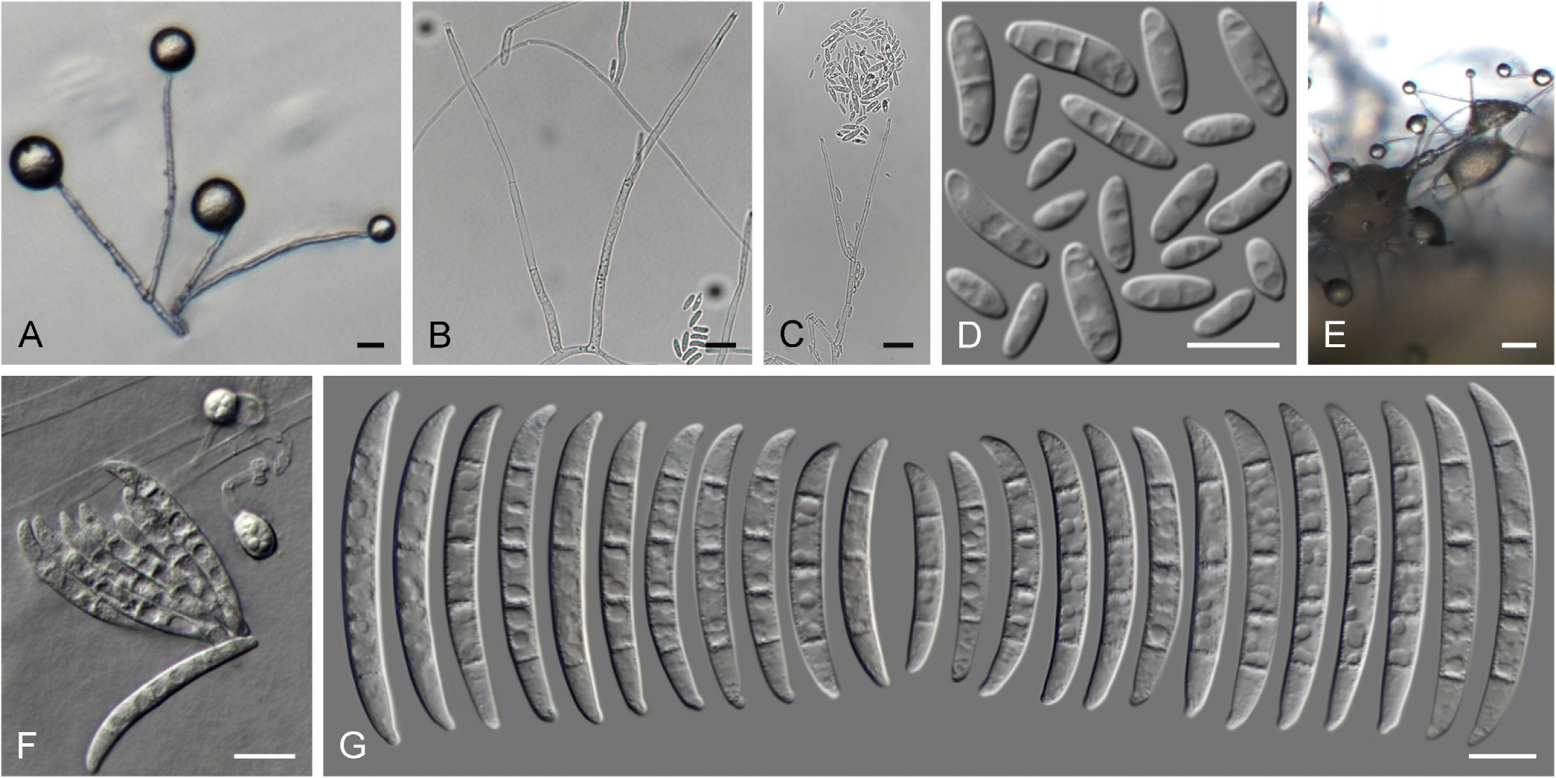
*Neocosmospora pseudopisi* (CBS 266.50). **A–C.** Conidiophores and conidiogenous cells. **D.** Microconidia. **E.** Sporodochia formed on aerial hyphae. **F.** Macroconidia and chlamydospores. **G.** Macroconidia. Scale bars: C = 20 μm; E = 100 μm; all others = 10 μm.

***Neocosmospora pseudopisi*** Sand.-Den. & L. Lombard, ***sp. nov*.** MycoBank MB 838673. Fig. 40.

*Etymology*: Named after its morphological, phylogenetic and host affinity with *Neocosmospora pisi*.  

*Typus*: **Unknown country**, from *Pisum sativum*, unknown date and collector (**holotype** CBS H-24668, culture ex-type CBS 266.50).  

*Conidiophores* borne on agar substrate and aerial mycelium, erect and prostrate, up to 340 μm tall, unbranched or irregularly laterally branched, bearing terminal single phialides, rarely proliferating percurrently; *aerial conidiogenous cells* monophialidic, rarely extended percurrently, subulate to subcylindrical, smooth- and thin-walled, 24.5–74 × 2–4 μm, with cup-shaped, elongated, and flared apical collarettes and conspicuous periclinal thickening. *Aerial microconidia* arranged in false heads on phialide tips, hyaline, broadly ellipsoidal to clavate, often lightly curved and asymmetrical, smooth- and thin-walled, 0(–1)-septate, (4.5–)6.5–11(–17.5) × (2–)3–4(–5) μm (av. 8.6 × 3.2 μm). *Sporodochia* pale luteous to pale sienna coloured, rarely formed on the surface of carnation leaves, agar surface or on aerial mycelium; *sporodochial conidiophores* unbranched or laterally and irregularly branched bearing single monophialides or groups of groups of up to three monophialides; *sporodochial conidiogenous cells* monophialidic, subulate to subcylindrical, 10–25 × 2–5 μm, smooth and thin-walled, collarettes and periclinal thickening present. *Sporodochial macroconidia* falcate, gently tapering towards both ends, slightly curved dorsoventrally to almost straight, apical cell blunt to inconspicuously papillate, basal cell obtuse to poorly-developed, foot-shaped, 1–4(–5)-septate, predominantly 4-septate, hyaline, smooth- and thick-walled; 1-septate conidia: 21.5–26(–27.5) × 4–5 μm (av. 24.7 × 4.3 μm); 2-septate conidia: 28–30 × 4.5–5 μm; 3-septate conidia: (28.5–)34–46.5(–50) × 4–5.5 μm (av. 40.1 × 4.7 μm); 4-septate conidia: (36–)42.5–54(–56) × 4–5.5 μm (av. 48 × 4.9 μm); 5-septate conidia: 50.5 × 5 μm; overall: (21.5–)34.5–51.5(–56) × 4–5.5 μm (av. 42.9 × 4.8 μm). *Chlamydospores* subspherical to spherical, hyaline to pale yellow, smooth-walled, thick-walled, 5.5–10.5 μm, single or in pairs, terminal or intercalary. *Sexual morph* not observed.  

*Culture characteristics*: Colonies on PDA reaching 35–48 mm diam at 25 °C after 7 d. Surface pale luteous to pale sulphur yellow, flat with abundant short aerial mycelium, velvety to dusty, margin regular entire to filiform; reverse pale luteous to sulphur yellow. On OA pale luteous to pale sulphur yellow, flat, velvety to dusty, margin entire to filiform; reverse pale luteous.  

*Notes*: The type of *N. pseudopisi* was determined as pathogenic to *Pisum sativum* and deposited in WI by W.C. Snyder. It is phylogenetically and morphologically related to *N. pisi*, a major pathogen of *Pisum sativum* (Šišić *et al.* 2018b). However, both species resolved as very closely related lineages in the seven-marker phylogeny (Fig. 14), as well as on the individual *CaM*, ITS, *rpb1*, and *rpb2* phylogenies (data not shown). Morphologically, *N. pseudopisi* can be differentiated from *N. pisi* by its longer sporodochial conidia (up to 56 μm long *vs* up to 46 μm long in *N. pisi*, Šišić *et al.* 2018b). Based on the features of its macroconidia, *N. pseudopisi* resembles *N. crassa* and *N. pseudotonkinensis*; the two latter species, though, are phylogenetically well-separated. *Neocosmospora pseudopisi*, however, differs from *N. crassa* and *N. pseudotonkinensis* by the absence of aerial macroconidia in the former species, while unlike *N. crassa*, the sporodochial conidia of *N. pseudopisi* are often wider on its apical third (*vs* wider at its basal part in *N. crassa*).  

***Neonectria*** Wollenw., Ann. Mycol. 15: 52. 1917, *nom. cons. prop*. Fig. 8.

*Synonym*: *Cylindrocarpon* Wollenw., Phytopathology 3: 225. 1913.

(see Chaverri *et al.* 2011 for additional synonyms)  

*Type species*: *Neonectria ramulariae* Wollenw., Ann. Mycol. 15: 52. 1917.  

*Ascomata* perithecial, gregarious, seated on an erumpent stroma, superficial, subglobose to broadly obpyriform, red, turning dark red in KOH, pigment dissolving in lactic acid, not collapsing when dry, with blunt to acute apex, rarely papillate, smooth to slightly rugulose, lacking hairs or appendages. *Ascomatal wall* of two regions: outer region of thick-walled, pigmented cells forming a *textura epidermoidea*; inner region of elongate, hyaline, thin-walled cells, becoming thinner toward the centrum. *Asci* cylindrical, 8-spored, without an apical ring, uniseriate. *Ascospores* ellipsoidal to fusoid, 1-septate, hyaline, smooth or finely spinulose. *Sporodochia* not formed. *Conidiophores* mononematous, hyaline, septate, unbranched or irregularly branched, terminating in 1–3 phialides or reduced to lateral phialides. *Conidiogenous cells* monophialidic, cylindrical, tapering towards the apex, with inconspicuous periclinal thickening and collarettes. *Microconidia* abundant, ellipsoidal to obovoid, hyaline, aseptate, sometimes forming false heads on phialides. *Macroconidia* cylindrical, mostly straight, 3–7(–9)-septate, with rounded ends. *Chlamydospores* globose to subglobose, hyaline to subhyaline, smooth-walled to slightly verrucose, terminal or intercalary, solitary or in pairs or forming chains.

[Description adapted from Chaverri *et al.* (2011)].  

*Diagnostic features*: Red, mostly smooth-walled perithecia lacking papilla producing cylindrical asci bearing ellipsoidal to fusoid, 1-septate ascospores and *Cylindrocarpon* asexual morph.  

***Nothofusarium*** Crous, Sand.-Den. & L. Lombard, ***gen. nov*.** MycoBank MB 838674. Fig. 8.  

*Etymology:* From the Greek prefix notho-, false, illegitimate; and *Fusarium*, in reference to the genetic affinity and morphological resemblance to the genus *Fusarium s. str.*  

*Type species*: *Nothofusarium devonianum* L. Lombard, Crous & Sand.-Den.  

*Ascomata* unknown. *Conidiophores* mononematous (aerial conidiophores) or grouped on sporodochia. *Aerial conidiophores* simple, unbranched or irregularly branched, sometimes reduced to single lateral phialides or phialidic pegs on the hyphae; *conidiogenous cells* monophialidic, cylindrical, tapering towards apex, smooth- and thin-walled, with periclinal thickening inconspicuous or absent, solitary. *Microconidia* not formed. *Aerial macroconidia* falcate, 1–5(–6)-septate, thick-walled, curved to lunate, with a blunt apical cell and often obtuse, poorly- to well-developed foot-shaped basal cell. *Sporodochia* white, pale luteous to pale citrine. *Sporodochial conidiophores* irregularly and verticillately branched, consisting of short, smooth- and thin- to thick-walled stipes bearing apical whorls of mono- and polyphialides. *Sporodochial conidiogenous cells* monophialidic and polyphialidic, doliiform, subulate to subcylindrical, smooth- and thin-walled, with reduced apical collarette. *Sporodochial macroconidia* similar to aerial macroconidia. *Chlamydospores* subglobose to ellipsoidal, solitary or most commonly in chains.  

*Diagnostic features*: Fusarioid asexual morph characterised by aerial monophialides and sporodochial mono- and polyphialides producing slightly curved and slender, mostly 3-septate macroconidia.  

**Fig. 41:**
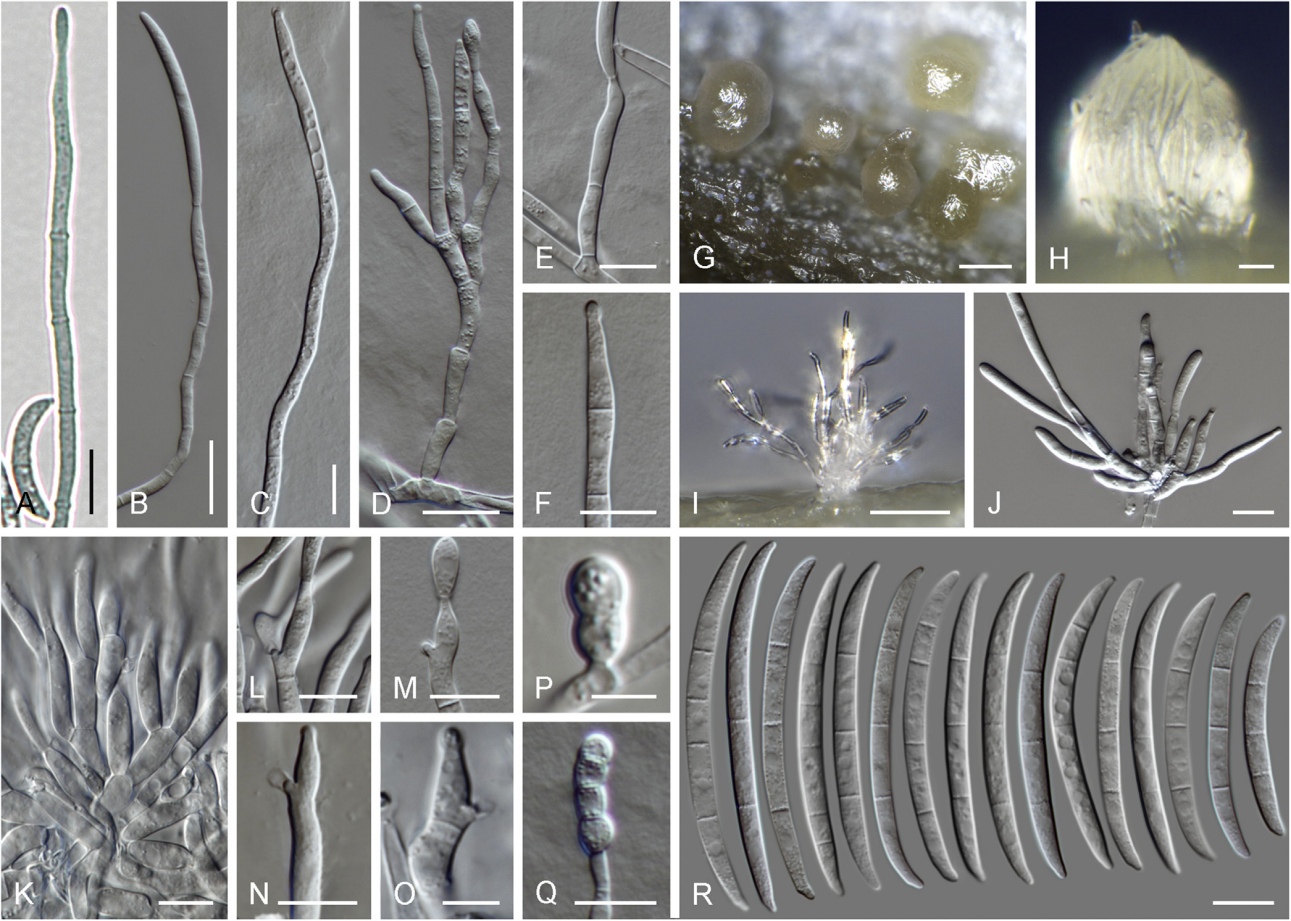
*Nothofusarium devonianum* (CBS 147304). **A–F.** Aerial conidiophores and conidiogenous cells. **G–I.** Sporodochia formed on the surface of carnation leaves. **J–O.** Sporodochial conidiophores and conidiogenous cells. **P, Q.** Chlamydospores. **R.** Macroconidia. Scale bars: B, D = 20 μm; G, H = 200 μm; O, P = 5 μm; all others = 10 μm.

***Nothofusarium devonianum*** L. Lombard, Crous & Sand.-Den., ***sp. nov*.** MycoBank MB 838675. Fig. 41.

*Etymology*: The epithet refers to Devon, the English county where the type specimen was collected.  

*Typus*: **UK**, England, Devon, Totnes, Berry Pomeroy, Loventor Manor, on dead cladodes of *Ruscus aculeatus*, 17 Jul. 1983, B.C. Sutton & A.V. Sutton (**holotype** CBS H-24670, culture ex-type CBS 147304 = IMI 279297 = NRRL 22134).  

*Conidiophores* borne on substrate mycelium, prostrate or erect and quickly collapsing to the agar surface, 70–240 μm tall, unbranched or less commonly irregularly laterally branched, bearing terminal single phialides; *aerial conidiogenous cells* monophialidic, subulate to cylindrical, smooth- and thin-walled, 9–34 μm long, 2–5 μm at the widest part, or reduced to short phialidic pegs, 3–6 × 2–3.5 μm, formed laterally on aerial hyphae, apical collarettes short or lacking, periclinal thickening absent. *Aerial macroconidia* borne on tips of conidiogenous cells on aerial conidiophores, almost straight or slightly curved, falcate, 1–5(–6)-septate, predominantly 3-septate, hyaline, smooth- and thick-walled, with a blunt apical cell and obtuse, sometimes papillate to poorly-developed, foot-shaped basal cell, 1-septate conidia: (15.5–)19–28(–32) × 2.5–4 μm (av. 23.5 × 4.3 μm); 2-septate conidia: (25.5–)27–31 × 2.5–4 μm (av. 28.8 × 3.2 μm); 3-septate conidia: (13–)41–57(–63.5) × 3–4(–4.5) μm (av. 49 × 3.6 μm); 4-septate conidia: (48.5–)50–60(–61.5) × 3–4.5 μm (av. 55.1 × 3.8 μm); 5-septate conidia: (47–)50–64(–71) × 3.5–4.5 μm (av. 56.9 × 3.9 μm); 6-septate conidia: (54–)55–71.5 × 3.5–4 μm (av. 62.3 × 3.8 μm); overall: (13–)35.5–59(–71.5) × 2.5–4.5 μm (av. 47.2 × 3.6 μm). *Sporodochia* pale luteous to pale citrine coloured, small, formed abundantly on the agar surface and less regularly on the surface of carnation leaves; *sporodochial conidiophores* irregularly verticillately branched bearing solitary lateral and terminal phialides or apical groups of 2–3 phialides; *sporodochial conidiogenous cells* mono- and polyphialidic, doliiform, subulate to subcylindrical, 3–25.5 × 2.5–5 μm, smooth and thin-walled, commonly proliferating sympodially, collarettes and periclinal thickening absent or inconspicuous. *Sporodochial conidia* undifferentiable from aerial conidia. *Chlamydospores* subglobose to ellipsoidal, solitary or most commonly in chains. *Sexual morph* unknown.  

*Culture characteristics*: Colonies on PDA reaching 23–27 mm diam at 25 °C after 7 d. Surface straw-coloured, pale luteous to pale ochreous, flat, dusty to velvety; reverse white to pale luteous without diffusible pigments. On OA, grey-white to pale luteous, flat, membranous to dusty, with irregular velvety peripheral patches cottony; reverse pale luteous.  

*Notes*: The type of *No. devonianum* was erroneously assigned to *Trichofusarium rusci* (Sutton, 1986) and recombined in *Fusarium* (*Fusarium rusci*, Geiser *et al.* 2013). Nevertheless, the morphology exhibited by this strain does not match in respect with the original description of the supposed basionym nor its purported synonym *Pycnofusarium rusci*, as confirmed also by examination of authentic material of *T. rusci* (BPI 453152A and IMI 291476). The latter taxon is characterised by a setose sporodochial asexual morph with small, fusoid, aseptate conidia, more reminiscent of the genus *Alfaria* (*Stachybotryaceae*, Crous *et al.* 2014).  

**Fig. 42:**
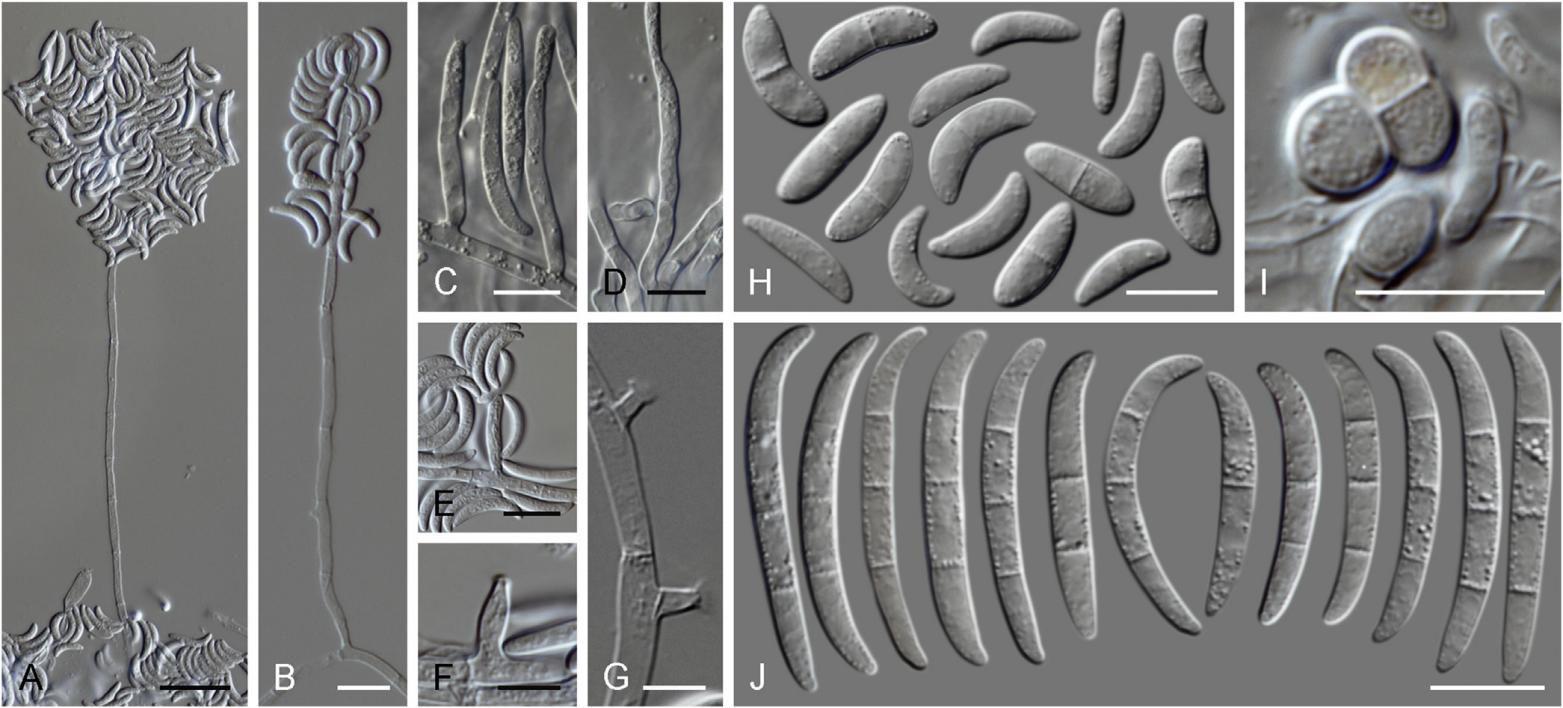
*Pseudofusicolla belgica*. **A, B.** Conidiophores. **C–G.** Conidiogenous cells. **H.** Microconidia. **I.** Chlamydospores. **J.** Macroconidia. A, B, D–J. IHEM 2413. C. IHEM 5322. Scale bars: A = 20 μm; F, G = 5 μm; all others = 10 μm.

***Pseudofusicolla*** D. Triest, Mycobiology 44: 127. 2016. Figs 8, 42.

*Type species*: *Pseudofusicolla belgica* D. Triest, Mycobiology 44: 127. 2016.  

*Ascomata* unknown. *Conidiophores* initially as lateral phialides on somatic hyphae, sometimes monochasial, verticillate or penicillate, hyaline. *Conidiogenous cells* monophialidic, cylindrical to subulate, hyaline, producing micro- and macroconidia. *Microconidia* strongly falcate, 0- or 1-septate, hyaline. *Macroconidia* strongly falcate, narrowing towards the ends, apical cell hooked with a pointed tip, basal cell papillate to poorly-developed, foot-shaped, 0–3-septate, hyaline. *Chlamydospores* globose, in terminal pairs or intercalary chains.

[Description adapted from Triest *et al.* (2016)].  

*Diagnostic features*: Fusarioid asexual morph that produces strongly curved, 0- or 1-septate microconidia, and 0–3-septate macroconidia.  

**Fig. 43:**
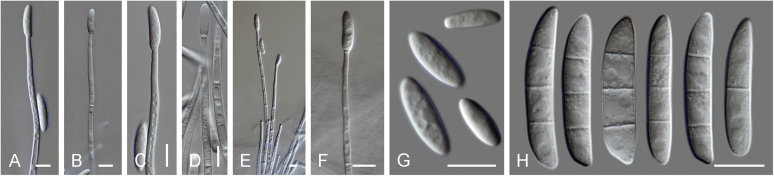
*Rectifusarium* spp. **A–F.** Conidiophores and conidiogenous cells. **G.** Microconidia. **H.** Macroconidia. A–D, H. *Rectifusarium robinianum* (CBS 430.91). E–G. *Rectifusarium ventricosum* (CBS 748.79). Scale bars = 10 μm.

***Rectifusarium*** L. Lombard *et al.*, Stud. Mycol. 80: 229. 2015. Figs 8, 43.

*Type species*: *Rectifusarium ventricosum* (Appel & Wollenw.) L. Lombard & Crous, Stud. Mycol. 80: 229. 2015.

*Basionym*: *Fusarium ventricosum* Appel & Wollenw., Phytopathology 3: 32. 1913.

(See *F. ventricosum* in List section for synonyms)  

*Ascomata* perithecial, mostly gregarious, non-stromatic or on a thin stroma erumpent through the epidermis, superficial, subglobose to globose, laterally pinched when dry, dark red, with short ostiolar neck, smooth-walled, lacking hairs and appendages. >*Ascomatal wall* of two regions: outer region of thick-walled, pigmented cells forming a *textura angularis* or *textura globulosa*; inner region of elongate, hyaline, thin-walled cells, becoming thinner towards the centrum. *Asci* clavate, apex rounded with distinct pore, 8-spored often with an apical ring, uniseriate to biseriate. *Ascospores* ellipsoidal, 1-septate, constricted at the septum, pale tan, verrucose. *Sporodochia* not formed. *Conidiophores* simple, mononematous, straight to flexuous, hyaline, septate, unbranched or rarely branched, terminating in single phialides. *Conidiogenous cells* monophialidic, cylindrical, tapering towards the apex, with periclinal thickening and flared collarettes, usually producing macroconidia. *Microconidia* rarely formed, ellipsoidal to fusoid, 0- or 1-septate, hyaline. *Macroconidia* falcate, straight to slightly curved dorsiventrally, 3-septate, with blunt to slightly pointed apical cell and poorly-developed foot-shaped basal cell. *Chlamydospores* globose to subglobose to ovoid, hyaline to subhyaline, verrucose, terminal or intercalary, solitary or in pairs or forming chains or developing directly from macroconidia.

[Description adapted from Booth (1971), Gerlach & Nirenberg (1982) and Lombard *et al.* (2015)].  

*Diagnostic features*: Dark red, smooth-walled perithecia with short ostiolar neck producing clavate asci bearing ellipsoidal, 1-septate ascospores and asexual morphs producing micro- and macroconidia on elongate cylindrical aerial conidiophores with monophialides, and not forming sporodochia. Chlamydospores formed in hyphae and macroconidia.  

***Rugonectria*** P. Chaverri & Samuels, Stud. Mycol. 68: 73. 2011. Fig. 8.  

*Type species*: *Rugonectria rugulosa* (Pat. & Gaillard) Samuels *et al.*, Stud. Mycol. 68: 73. 2011.

*Basionym*: *Nectria rugulosa* Pat. & Gaillard, Bull. Soc. Mycol. France 4: 115. 1889.

*Synonyms*: *Cucurbitaria rugulosa* (Pat. & Gaillard) Kuntze, Revis. Gen. Pl. 3: 461. 1898.

*Neonectria rugulosa* (Pat. & Gaillard) Mantiri & Samuels, Canad. J. Bot. 79: 339. 2001.

*Cylindrocarpon rugulosum* Brayford & Samuels, Sydowia 46: 148. 1994.  

*Ascomata* perithecial, solitary or gregarious, stromatic, superficial or partly immersed in stroma, subglobose to globose, orange to red, turning dark red in KOH, pigment dissolving in lactic acid, non-papillate, rugose to tuberculate, lacking hairs or appendages. *Ascomatal wall* of two regions: outer region of thick-walled, pigmented cells forming a *textura angularis*; inner region of elongate, hyaline, thin-walled cells, becoming thinner towards the centrum. *Asci* clavate, apex simple, 8-spored. *Ascospores* ellipsoidal to oblong, 1-septate, not to slightly constricted at the septum, pale yellow, striate. *Sporodochia* not formed. *Conidiophores* simple, mononematous, straight to flexuous, hyaline, septate, unbranched or rarely to irregularly branched, terminating in single phialides. *Conidiogenous cells* monophialidic, cylindrical, tapering towards the apex, with periclinal thickening and flared collarettes, producing micro- and macroconidia. *Microconidia* ovoid to cylindrical, 0- or 1-septate, hyaline. *Macroconidia* fusoid, curved, (3–)5–7(–9)-septate, tapering to both ends, basal cell obtuse with inconspicuous hilum. *Chlamydospores* not observed.

[Description adapted from Samuels *et al.* (1990), Samuels & Brayford (1994) and Chaverri *et al.* (2011)].  

*Diagnostic features*: Orange to red, rugose to tuberculate, partially immersed perithecia producing clavate asci bearing fusoid, 1-septate yellowish, striate ascospores and cylindrocarpon-like asexual morph characterised by curved, multiseptate macroconidia with inconspicuous hilum.  

**Fig. 44:**
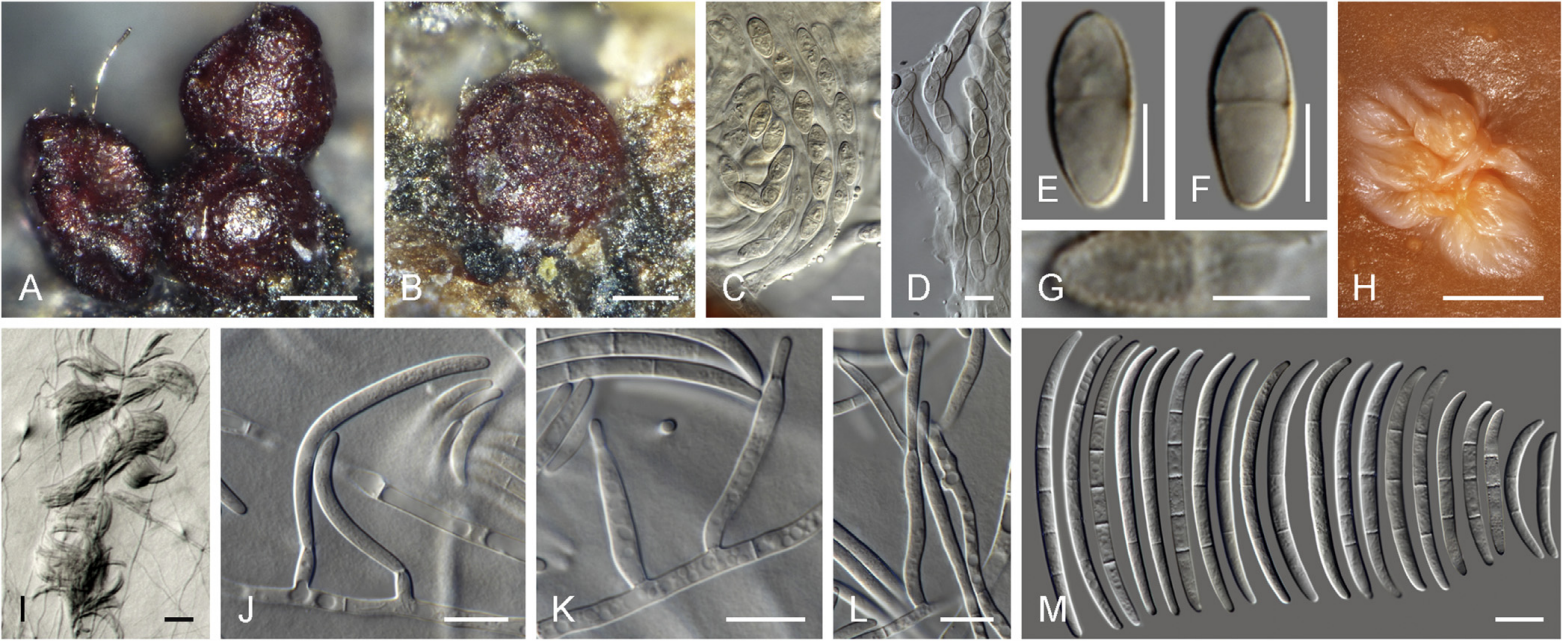
*Scolecofusarium ciliatum*. **A, B.** Ascomata on natural substrate. **C, D.** Asci. **E–G.** Ascospores (G. Surface view). **H.** Pionnote on agar surface. **I.** Sporodochium. **J–L.** Conidiophores and conidiogenous cells. **M.** Macroconidia. A–H, J–L. CBS 146674. I. CBS 146676. M. CBS 144385. Scale bars: A, B = 100 μm; E–G = 5 μm; H = 1 mm; I = 20 μm; all others = 10 μm.

***Scolecofusarium*** L. Lombard, Sand.-Den. & Crous, ***gen. nov*.** MycoBank MB 838676. Figs 8, 44.

*Etymology*: From Greek skṓlēx, worm, in reference to the worm-like appearance of the macroconidia.  

*Type species*: *Scolecofusarium ciliatum* (Link) L. Lombard, Sand.-Den. & Crous  

*Ascomata* perithecial, solitary or gregarious, partially immersed on a stroma, smooth- and thin-walled, globose to broadly pyriform, red, with a broad, discoid apical region, turning darker in KOH, pigment dissolving in lactic acid to become yellow, lacking hairs and warts. *Ascomatal wall* of a single region composed of unevenly thickened cells of *textura epidermoidea*. *Asci* cylindrical, apex with an obscure refractive ring, 8-spored, ascospores uniseriate. *Ascospore*s ellipsoidal to fusiform-ellipsoidal, 1-septate, not constricted at septum, yellow-brown, finely spinulose. *Conidiophores* mononematous (aerial) or grouped on sporodochia. *Aerial conidiophores* unbranched to loosely irregularly branched, bearing terminal phialides; *conidiogenous cells* monophialidic, subcylindrical, smooth- and thin-walled, with evident periclinal thickening and a non-flared collarette, producing only macroconidia. *Sporodochia* pink, orange to salmon coloured; *sporodochial conidiophores* irregularly and verticillately branched, consisting of short, often swollen, smooth- and thin-walled stipes bearing single terminal monophialides or apical whorls of 2–3 monophialides; *sporodochial conidiogenous cells* monophialidic, cylindrical to subcylindrical, smooth- and thin-walled, with evident periclinal thickening. *Macroconidia* formed in pink to salmon slimy masses, subcylindrical, (0–)3–7(–10)-septate, straight or slightly curved, with blunt apical cell and obtuse to poorly developed, foot-shaped basal cell. *Microconidia* unknown. *Chlamydospores* unknown.

[Description adapted from Samuels *et al.* (1991) & Gerlach & Nirenberg (1982)].  

*Diagnostic features*: Red perithecia producing cylindrical asci containing ellipsoidal, 1-septate, finely spinulose ascospores and fusarioid asexual morph characterised by monophialides producing slender and delicate, almost cylindrical macroconidia from aerial conidiophores and pink to salmon coloured sporodochia, lacking microconidia as well as chlamydospores.  

***Scolecofusarium ciliatum*** (Link) L. Lombard, Sand.-Den. & Crous, ***comb. nov*.** MycoBank MB 838677.

*Basionym*: *Atractium ciliatum* Link, Mag. Ges. Naturf. Freunde Berlin 7: 32. 1816.

*Synonyms*: *Fusarium ciliatum* (Link) Link, in Willdenow, Sp. Pl., Ed. 4, 6: 105. 1825.

*Microcera ciliata* (Link) Wollenw., Fusaria Autogr. Delin. 1: 435. 1916.

*Calonectria ciliata* (Link) W.C. Snyder & H.N. Hansen, Amer. J. Bot. 32: 664. 1945.

*Sphaeria agnina* Desm., Ann. Sci. Nat., Bot. sér. 3, 6: 72. 1846.

*Calonectria agnina* (Desm.) Sacc., Michelia 1: 311. 1878.

*Dialonectria agnina* (Desm.) Cooke, Grevillea 12: 111. 1884.

*Fusarium peltigerae* Westend., Herb. Crypt. Belg. 9: no. 414. 1849.

*Fusarium parasiticum* Westend., Bull. Séances Cl. Sci. Acad. Roy. Sci. Belgique, sér. 2, 11: 652. 1861.

*Nectria massariae* Pass., in Rabenhorst, Fungi Eur. Exs. no. 1827. 1874.

*Microcera massariae* Sacc., Michelia 1: 263. 1878.

*Calonectria massariae* (Pass.) Sacc., Michelia 1: 312. 1878.

*Fusisporium filisporum* Cooke, Grevillea 8: 8. 1879.

*Fusarium filisporum* (Cooke) Sacc., Syll. Fung. 4: 708. 1886.

*Fusarium scolecoides* Sacc. & Ellis, Atti Reale Ist. Veneto Sci. Lett. Arti, sér. 6, 3: 728. 1885.

*Fusarium elongatum* Cooke, Grevillea 19: 4. 1890.

*Calonectria dearnessii* Ellis & Everh., Proc. Acad. Nat. Sci. Philadelphia 42: 245. 1891.  

*Typus*: **Germany**, on branch canker of *Fagus sylvatica*, 1961, W. Gerlach (**neotype** of *Atractium ciliatum* CBS H-12687 *hic designatus*, MBT 10000646, culture ex-neotype CBS 191.65 = ATCC 16068 = ATCC 24137 = BBA 9661 = DSM 62172 = IMI 112499 = NRRL 20431).  

*Additional descriptions and illustrations*: Wollenweber & Reinking (1935), Doidge (1938), Gerlach & Nirenberg (1982).  

*Additional material examined*: **Belgium**, Mons, Pommeroeul, on leaf of *Fagus sylvatica*, 1984, unknown collector, culture CBS 144385 = IHEM 2989. **Denmark**, on *Hordeum vulgare* mouldy grain, associated with scale insects, 1986, U. Thrane, culture CBS 155.86 = NRRL 22284. **Netherlands**, Noord-Brabant Province, Boxmeer, on *Quercus* sp., Mar. 2016, S. Helleman, cultures CBS 146672 = CPC 30654; CBS 146673 = CPC 30655; CBS 146674 = CPC 30656; CBS 146675 = CPC 30657; CBS 146676 = CPC 30658; CBS 146677 = CPC 30659.  

*Notes:* No existent holotype material was located for *At. ciliatum*. Therefore, a neotype is designated here. The neotype specimen originates from a representative isolate studied by Gerlach & Nirenberg (1982).  

**Fig. 45:**
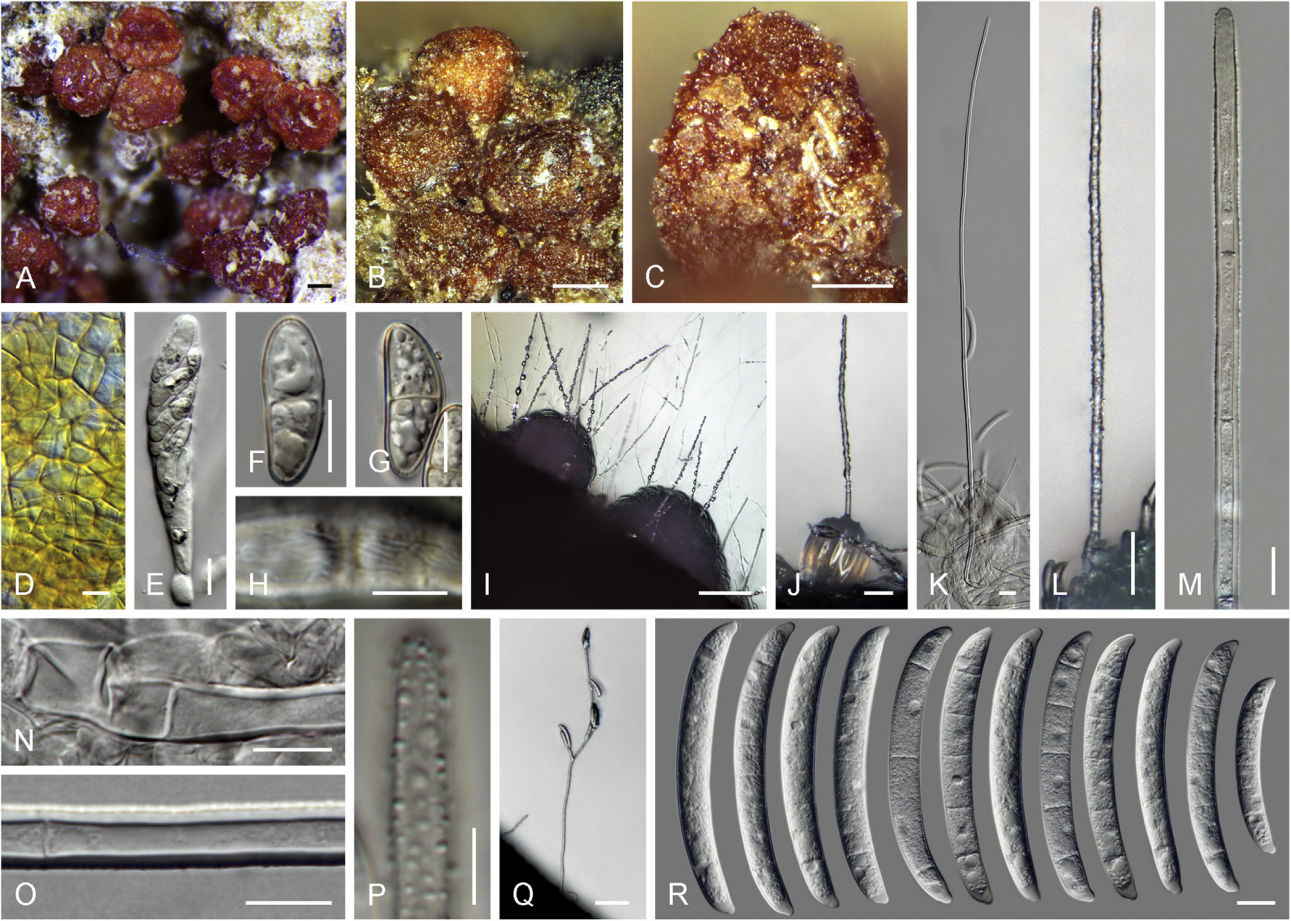
*Setofusarium setosum*. **A–C.** Ascomata on natural substrate. **D.** Surface view of perithecial wall in lactic acid. **E.** Ascus. **F–H.** Ascospores (H. Surface view). **I, J.** Setose sporodochia. **K–M.** Setae. **N–P.** Detail of setae (N. Base. O. Middle portion wall. P. Surface view of apical wall). **Q.** Conidiophore. **R.** Macroconidia. A–H. BPI 882043. I–R. CBS 635.92. Scale bars: A–C, I, Q = 100 μm; J–L = 20 μm; H, P = 5 μm; all others = 10 μm.

***Setofusarium*** (Nirenberg & Samuels) Crous & Sand.-Den., ***gen. et stat. nov*.** MycoBank MB 838678. Figs 8, 45.

*Basionym*: *Fusarium* sect. *Setofusarium* Nirenberg & Samuels, Canad. J. Bot. 67: 3376. 1989.  

*Etymology*: The name refers to the presence of setose sporodochia and to its resemblance to the genus *Fusarium*.  

*Type species*: *Fusarium setosum* Nirenberg & Samuels, Canad. J. Bot. 67: 3372. 1989.  

*Ascomata* perithecial, solitary or gregarious on a well-developed immersed stroma composed of pseudoparenchymatous to hyphal cells, scaly to warty and thick-walled, pyriform, dark red with an often darker red-coloured, flattened and non-papillate apical region, turning darker in KOH, pigment dissolving in lactic acid to become yellow, lacking hairs. >*Ascomatal wall* of two regions: outer region of thick-walled, pigmented cells of *textura angularis* to *textura globulosa* at warts cells; inner region of elongate, hyaline, thin-walled cells, becoming thinner towards the centrum. *Asci* cylindrical to clavate, with rounded to flattened simple apex, 8-spored, ascospores overlapping uniseriate to biseriate. *Ascospore*s ellipsoidal, 1-septate, not constricted at septum, pale yellow-brown, smooth-walled to finely striate. *Conidiophores* mononematous (aerial) or grouped on sporodochia. *Aerial conidiophores* unbranched or rarely branched, bearing terminal phialides; *conidiogenous cells* monophialidic, cylindrical to subcylindrical, smooth- and thin-walled, with periclinal thickening inconspicuous to evident, producing only macroconidia. *Sporodochia* grey; *setae* arising between and around sporodochia, stiff, erect, thick-walled with acute tip, at first hyaline later becoming pale golden brown; *sporodochial conidiophores* irregularly and verticillately branched and densely packed, consisting of short, often swollen, smooth- and thin-walled stipes bearing apical whorl of 2–3 monophialides or single, terminal monophialides; *sporodochial conidiogenous cells* monophialidic, cylindrical to subcylindrical, smooth- and thin-walled, with inconspicuous to evident periclinal thickening. *Macroconidia* formed in off-white or grey slimy masses, cylindrical, (0–)3–5(–7)-septate, gently curved, with a blunt apical cell and an obtuse to poorly developed foot-shaped basal cell. *Microconidia* unknown. *Chlamydospores* unknown.

[Description adapted from Samuels & Nirenberg (1989)].  

*Diagnostic features*: Dark red perithecia producing cylindrical to clavate asci containing ellipsoidal, 1-septate, finely striate ascospores and fusarioid asexual morph characterised by monophialides producing robust, almost cylindrical macroconidia from aerial conidiophores and setose sporodochia, lacking microconidia as well as chlamydospores.  

***Setofusarium setosum*** (Nirenberg & Samuels) Sand.-Den. & Crous, ***comb. nov*.** MycoBank MB 838679.

*Basionym*: *Fusarium setosum* Nirenberg & Samuels, Canad. J. Bot. 67: 3372. 1989.

*Synonym*: *Nectria setofusarii* Samuels & Nirenberg, Canad. J. Bot. 67: 3372. 1989.  

*Typus*: **French Guiana**, piste de Saint-Elie: km 16 on road between Sinnamary and St. Elie, ORSTOM research area “ECEREX”, on bark of living liana, Mar. 1986, G.J. Samuels, **holotype** NY00927992. **Epitype** of *F. setosum* (CBS H-24723 *hic designatus*, MBT 10000647): **French Guiana**, Vic. Cayenne, 15 km from Remise, trail to Vidal-old farm, secondary forest, from bark, 25 Feb. 1988, A.Y. Rossman, culture ex-epitype CBS 635.92 = G.J.S. 88-12.  

*Description and illustrations*: Samuels & Nirenberg (1989).  

*Additional material examined*: **French Guiana**, unknown host and collection date, A.Y. Rossman, culture CBS 574.94; from wood from unknown host, Feb. 1988, A.Y. Rossman, IMI 324476. **Ghana**, Western Region: Wiawso District, Bia National Park, trail from camp 1, disturbed forest, on living liana, J.G. Samuels & H.C. Evans, BPI 882043.  

*Notes*: The monotypic, former *Fusarium* section *Setofusarium* is here elevated to generic rank to accommodate “*Fusarium setosum*”, a genetically and morphologically divergent taxon easily differentiated from any known fusarioid taxa by the production of setose sporodochia (Samuels & Nirenberg 1989). No living ex-type culture could be located for this taxon. Isolate CBS 635.92 (as G.J.S. 88-12) is an authentic strain of *Fusarium setosum* (Samuels & Nirenberg 1989). Therefore, a dried culture from this strain is designated as epitype here.  

**Fig. 46:**
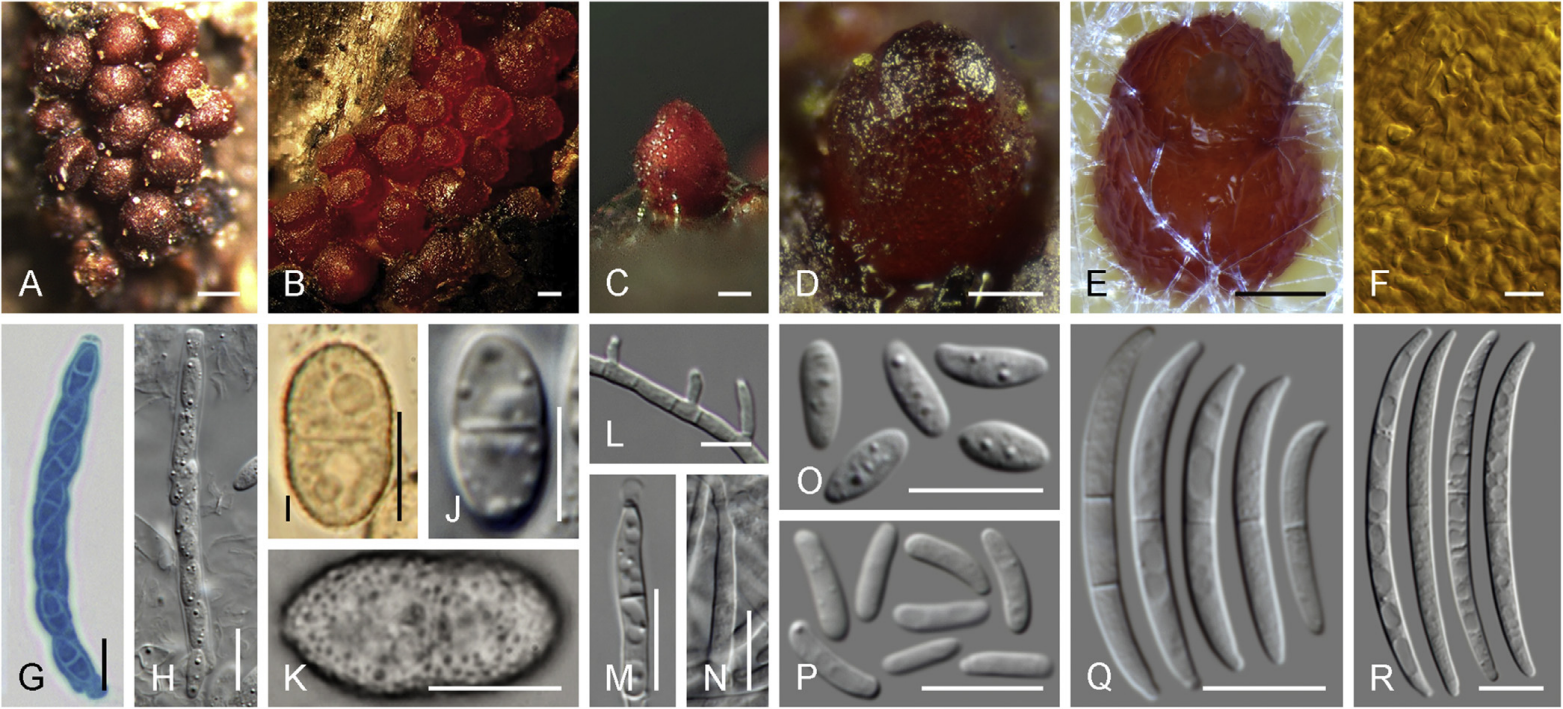
*Stylonectria* spp. **A–D.** Ascomata on natural substrate. **E.** Ascomata on culture. **F.** Surface view of perithecial wall in lactic acid. **G, H.** Asci. **I–K.** Ascospores. **L–M.** Conidiophores and conidiogenous cells. **O, P.** Microconidia. **Q, R.** Macroconidia. A, G. *Stylonectria qilianshanensis* [HMAS 255803, adapted from Zeng *et al.* (2020)]. B. *Stylonectria norvegica* [CLL14047, adapted from Lechat *et al.* (2015)]. C. *Stylonectria purtonii* (photo P. Mlčoch). D, I, K. *Stylonectria wegeliana* (photo B. Bergen). E, F, Q. *Stylonectria hetmanica* (CBS 147306). H, J, O. *Stylonectria* sp. (HPC 2668). L, M. *Stylonectria corniculata* (CBS 125491). N, P, R. *Stylonectria applanata* (CBS 125489). Scale bars: A–E = 100 μm; I–K = 5 μm; all others = 10 μm.

***Stylonectria*** Höhn., Sitzungsber. Kaiserl. Akad. Wiss. Wien, Math.-Naturwiss. Cl., Abt. 1, 124: 52. 1915. Figs 8, 46.

*Type species*: *Stylonectria applanata* Höhn., Sitzungsber. Kaiserl. Akad. Wiss. Wien, Math.-Naturwiss. Kl., Abt. 1, 124: 52. 1915.  

*Ascomata* perithecial, gregarious in groups of up to 20, on a thin, white to yellow hyphal or subiculum-like stroma, superficial, subglobose, pyriform to subcylindrical, pale yellow, orange-red, orange-brown, or pale to dark red, becoming dark red to purple in KOH, with a rounded or broad, circular, flat disc on a venter-like neck, smooth to slightly rugulose, lacking hairs or appendages. *Ascomatal wall* consisting of two layers; inner layer of hyaline, thin-walled, compressed, elongated cells and outer layer of distinct, isodiametric to oblong, angular or globose, thick-walled cells. *Asci* cylindrical to clavate, 8-spored, with simple apex or apical ring. *Ascospores* cylindrical to allantoid to ellipsoidal, 1-septate, hyaline or yellow to pale brown, smooth or tuberculate. *Conidiophores* initially formed as unbranched phialides on somatic hyphae, sometimes loosely branched, sometimes forming small sporodochia. *Conidiogenous cells* monophialidic, cylindrical to subcylindrical, with a distinct collarette. *Microconidia* sparse, allantoid to lunulate, slightly or strongly curved, aseptate, in slimy heads. *Macroconidia* orange in mass, subcylindrical or moderately to strongly curved, falcate, 0- or 1-septate, apex narrower than base, apical cell blunt or hooked, basal cell not or scarcely foot-shaped.

[Description adapted from Höhnel (1915) and Gräfenhan *et al.* (2011)].  

*Diagnostic features*: Pale yellow to dark red, mostly smooth-walled perithecia with rounded or broad, circular, flat disc on a venter-like neck, producing cylindrical to clavate asci bearing cylindrical to allantoid to ellipsoidal, 1-septate hyaline or yellow to pale brown ascospores and fusarioid asexual morph characterised by 0- or 1-septate macroconidia with blunt or hooked apical cell, lacking a foot-shaped basal cell.  

**Fig. 47:**
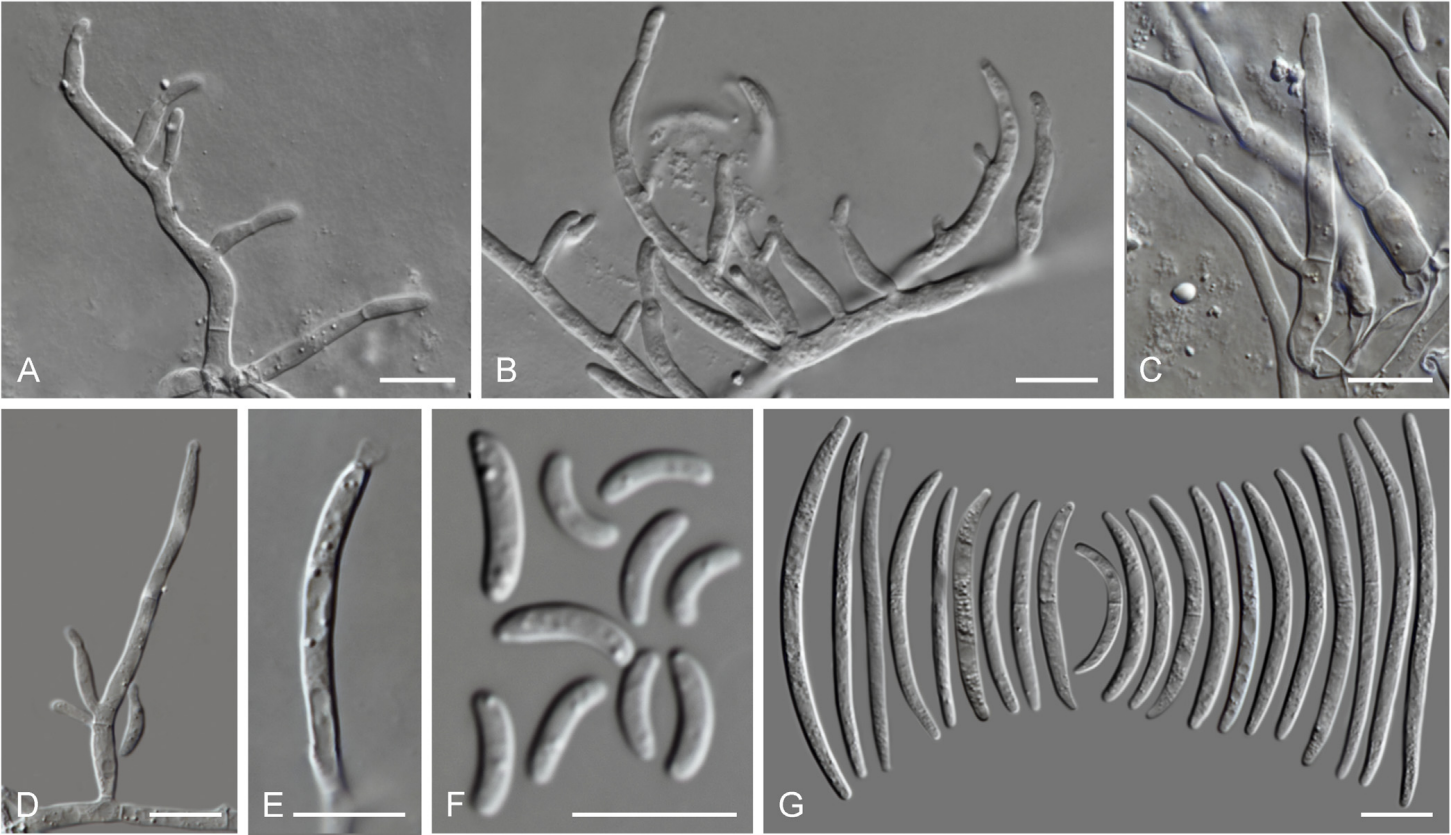
*Stylonectria corniculata* (CBS 125491). **A–E.** Conidiophores and conidiogenous cells. **F.** Microconidia. **G.** Macroconidia. Scale bars = 10 μm.

***Stylonectria corniculata*** Gräfenhan, Crous & Sand.-Den., ***sp. nov*.** MycoBank MB 838680. Fig. 47.

*Etymology*: From Latin *corniculum*, little horn. Referring to the shape of the conidiophores.  

*Typus*: **Germany**, Brandenburg, Stolpe, near Gellmersdorfer Forst, from unidentified ascomycete on *Carpinus* sp., 1 Mar. 2007, T. Gräfenhan, **holotype** CBS H-24671, culture ex-type CBS 125491.  

*Conidiophores* often as single phialides borne laterally on substrate and aerial hyphae, or irregularly branched and crowded with phialides produced laterally and terminally, hyaline, thin- and smooth-walled, 24–89 μm long. *Conidiogenous cells* monophialidic, short doliiform, subcylindrical to subulate, 6–28.5 × 2–3.5 μm, often with a conspicuous flared collarette, periclinal thickening absent, producing micro- and macroconidia. *Microconidia* cylindrical to allantoid, hyaline, thin- and smooth-walled, 0(–1)-septate, (4.5–)6–13.5(–21) × (1.5–)2–3 μm (av. 9.7 × 2.1 μm). *Macroconidia* falcate, almost straight or gently dorsiventrally curved, tapering toward the basal portion, (0–)1-septate, with a blunt apical cell and obtuse basal cell, (20–)28–47(–56) × 2–3.5 μm (av. 37.6 × 2.5 μm). *Chlamydospores* and *sexual morph* not observed.  

*Culture characteristics*: Colonies on PDA reaching 16–20 mm diam at 25 °C after 7 d. Surface at first white and membranous, becoming slimy, saffron to orange, to bright orange at the centre, flat, aerial mycelium absent, moisty at the centre, velvety at the margin, margin regular, filiform to undulate; reverse white, pale saffron to orange at centre. On OA, white to pale orange, flat, membranous to slimy, with regular and undulate margin; reverse pale luteous to pale saffron.  

*Notes*: The species is here described based on its morphology *in vitro*, where only the asexual morph was obtained. This prevents further comparisons with known species of this genus. The only known collection, CBS 125491, has been shown to represent the most basal lineage in *Stylonectria* in previous phylogenetic studies (Gräfenhan *et al.* 2011, Lechat *et al.* 2015), which was confirmed here (Fig. 15). Although with neither a clear host association – an important character for species recognition in *Stylonectria* – nor any known sexual morphology, *St. corniculata* shows a distinctive morphology when it comes to its asexual morph, especially regarding the branching pattern and the shape of its mature conidiophores, which can be very elaborate and largely resemble antlers (Fig. 47).  

**Fig. 48:**
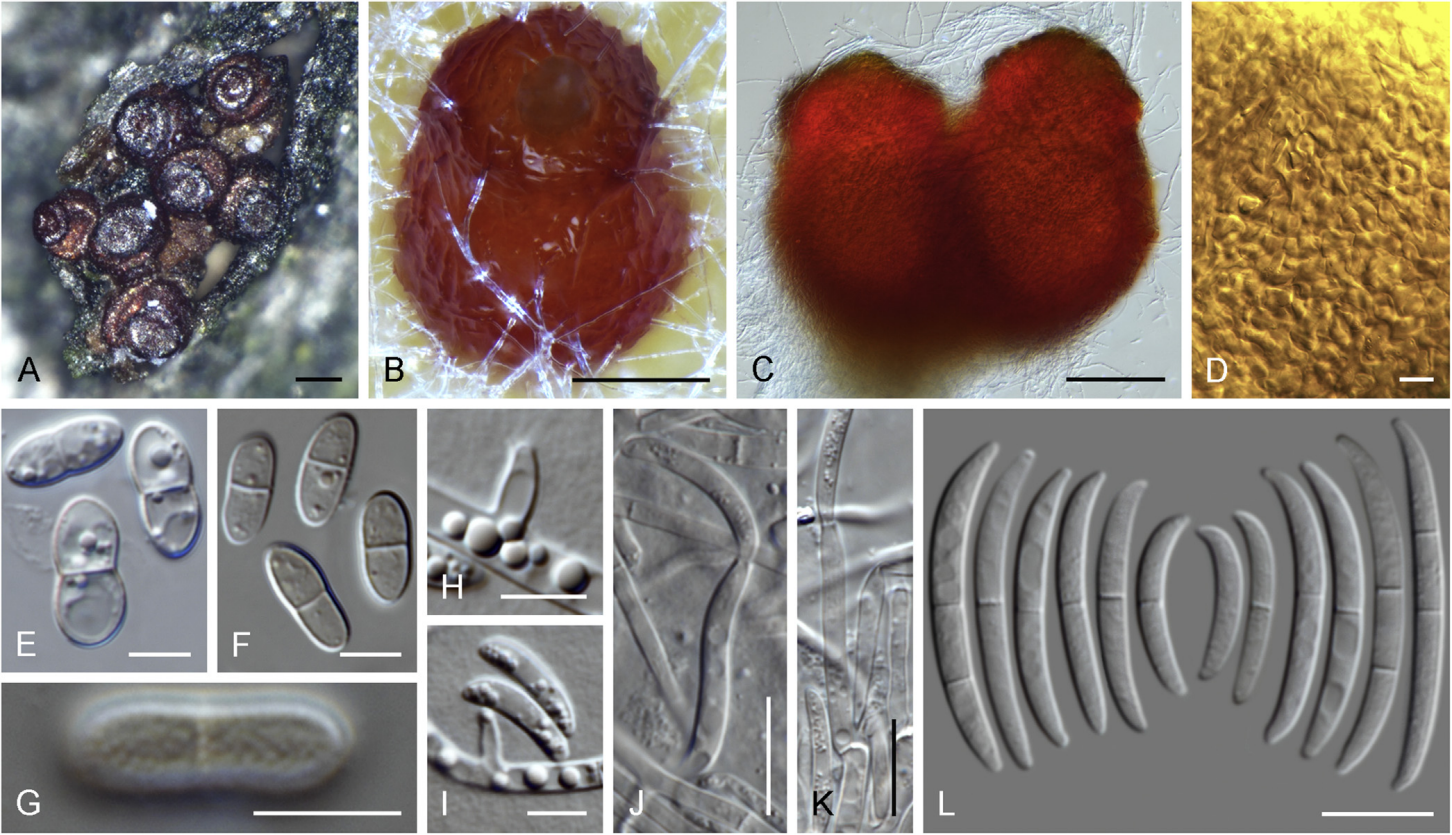
*Stylonectria hetmanica* (CBS 147305). **A–C.** Ascomata (A. On natural substrate. B, C. In culture). **D.** Surface view of perithecial wall in lactic acid. **E–G.** Ascospores (G. Surface view). **H–K.** Conidiophores and conidiogenous cells. **L.** Macroconidia. Scale bars: A–C = 100 μm; E–I = 5 μm; all others = 10 μm.

***Stylonectria hetmanica*** Akulov, Crous & Sand.-Den., ***sp. nov*.** MycoBank MB 838681. Fig. 48.

*Etymology:* The epithet refers to the Cossack Hetmanate (Ukrainian Hetmanščyna), the name of the former Cossack state territories where the type was collected.  

*Typus*: **Ukraine**, Sumy, Okhtyrka, vicinities of Klymentove village, Hetmanskyi National Nature Park, on the ascomata of *Diaporthe* sp., associated with *Phomopsis* asexual morph, on dead branches of *Frangula alnus* still attached to the tree, 13 Oct. 2019, Ya. Mieshkov, CWU (Myc) AS 7177, **holotype** CBS H-24672, culture ex-type CBS 147305 = CPC 38725.  

*Ascomata* perithecial, gregarious or solitary, broadly pyriform, 220–310 μm wide, with a distinctive flat and discoid papilla, 130–225 μm wide, dark red, becoming darker in 3 % KOH and light yellow in lactic acid. *Ascomatal wall* smooth, 30–45 um thick, composed of two regions: outer region 25–40 μm thick, of irregularly shaped cells of *textura intricata* to *textura epidermoidea*; inner region 5–10 μm thick of thin-walled, flattened cells of *textura prismatica* to *textura angularis*. *Asci* subcylindrical, 45–72 × 4–8 μm, 8-spored, apices rounded and simple, uniseriate or irregularly biseriate. *Ascospores* ellipsoidal, 1-septate, often constricted at septum, (7.5–)8.5–11(–12.5) × 3–4.5(–5.5) μm, smooth to finely spinulose, thick-walled, hyaline at first, becoming pale golden brown at maturity. *Conidiophores* often as single phialides or short phialidic pegs borne laterally on the substrate and aerial hyphae, rarely irregularly to verticillately branched. *Conidiogenous cells* monophialidic, short doliiform, subcylindrical to subulate, 4–21(–27.5) × 2–3.5 μm, often with a conspicuous flared collarette, periclinal thickening absent, producing micro- and macroconidia. *Microconidia* allantoid, hyaline, smooth- and thin-walled, 0(–1)-septate, (9–)10.5–13.5(–15) × 2–3 μm (av. 12 × 2.4 μm). *Macroconidia* subcylindrical to falcate, almost straight or moderately dorsiventrally curved, tapering towards both ends, 0–1(–2)-septate, apical cell blunt to slightly hooked, basal cell obtuse to poorly-developed, foot-shaped (11.5–)16.5–28(–34) × 2–3 μm (av. 22.2 × 2.5 μm). *Chlamydospores* not observed.  

*Culture characteristics*: Colonies on PDA reaching 2.5–3 mm diam at 25 °C after 7 d. Surface straw-coloured to luteous, pale orange at centre, flat or radially folded, membranous to slimy, margin filiform to undulate; reverse pale luteous to pale orange. On OA orange to pale apricot, flat, membranous to slimy, margin filiform with abundant submerged mycelium; reverse pale orange.  

*Additional material examined*: **Ukraine**, Sumy, Okhtyrka, in the vicinities of the village Klymentove, Hetmanskyi National Nature Park, on the conidiomata of *Dothiorella sarmentorum*, on recently dead branches of *Acer platanoides* still attached to the tree, 13 Oct. 2019, A. Akulov, CWU (Myc) AS 7278, culture CBS 147306 = CPC 38848.  

*Notes*: The morphological description of *St. hetmanica* is based on its growth on OA, where both studied strains showed optimal growth and sporulation. Contrary to most fusarioid genera, *St. hetmanica* grows very poorly and fails to sporulate on SNA and WA. *Stylonectria hetmanica* is morphologically comparable and genetically close to *St. purtonii*, *St. norvegica*, and *St. wegeliana*. Nevertheless, ascospores of *St. hetmanica* are smaller than those of *St. purtonii* and *St. wegeliana*. Additionally, macroconidia of *St. hetmanica*, while similar in size to those of *St. purtonii*, are less septate (0- or 1-septate, rarely 2-septate in *St. hetmanica*, and up to 3-septate in *St. purtonii*). The sexual morph of the recently described *St. norvegica* is very similar to that of *St*. *hetmanica*, although both species are genetically less closely related. The latter species can be distinguished by the production of shorter macroconidia.  

***Thelonectria*** P. Chaverri & C. Salgado, Stud. Mycol. 68: 76. 2011. Fig. 8.  

*Type species*: *Thelonectria discophora* (Mont.) P. Chaverri & C. Salgado, Stud. Mycol. 68: 76. 2011.

*Basionym*: *Sphaeria discophora* Mont., Ann. Sci. Nat. Bot., sér. 2, 3: 353. 1835.

*Synonyms*: *Nectria discophora* (Mont.) Mont., Fl. Chil. 7: 454. 1850.

*Cucurbitaria discophora* (Mont.) Kuntze, Revis. Gen. Pl. 3: 461. 1898.

*Neonectria discophora* (Mont.) Mantiri & Samuels, Canad. J. Bot. 79: 339. 2001.

*Nectria tasmanica* Berk., in Hooker, Bot. Antarct. Voy. III, Fl. Tasman. 2: 279. 1860.

*Cucurbitaria tasmanica* (Berk.) Kuntze, Revis. Gen. Pl. 3: 462. 1898.

*Nectria umbilicata* Henn., Hedwigia 41: 3. 1902.

*Creonectria discostiolata* Chardón, Bol. Soc. Venez. Ci. Nat. 5: 341. 1939.  

*Ascomata* perithecial, solitary to gregarious, non-stromatic or sometimes seated on an immersed inconspicuous stroma, superficial, globose to subglobose or pyriform to elongated, orange to red, with prominent areolate papilla or darkly pigmented apex, smooth to slightly rugulose, lacking hairs or appendages. *Ascomatal wall* of two regions: outer region of thick-walled, pigmented cells forming a *textura epidermoidea*; inner region of elongate, hyaline, thin-walled cells, becoming thinner towards the centrum. *Asci* cylindrical to narrowly clavate, 8-spored, with an apical ring, uniseriate. *Ascospores* ellipsoidal to fusoid, 1-septate, hyaline, smooth or finely spinulose or striate. *Sporodochia* not formed. *Conidiophores* mononematous, hyaline, septate, irregularly branched, terminating in 1–3 phialides or reduced to lateral phialides. *Conidiogenous cells* monophialidic, cylindrical or slightly swollen, tapering towards the apex, with periclinal thickening and flared collarettes, producing usually macroconidia. *Microconidia* rarely formed, globose to ovoid, hyaline, aseptate, with displaced inconspicuous hilum. *Macroconidia* subcylindrical to slightly fusoid, curved, broadest at upper third, (3–)5–7(–9)-septate, with rounded ends or flattened at the basal cell. *Chlamydospores* unknown.

[Description adapted from Chaverri *et al.* 2011)].  

*Diagnostic features*: Orange to red, mostly smooth-walled perithecia with prominent darkened papilla producing cylindrical to narrowly clavate asci bearing ellipsoidal to fusoid, 1-septate ascospores and cylindrocarpon-like asexual morph.  

***Tumenectria*** C. Salgado & Rossman, Fungal Diversity 80: 451. 2016. Fig. 8.  

*Type species*: *Tumenectria laetidisca* (Rossman) C. Salgado & Rossman, Fungal Diversity 80: 451. 2016.

*Basionym*: *Nectria laetidisca* Rossman, Mycol. Pap. 150: 36. 1983.

*Synonym*: *Cylindrocarpon bambusicola* Matsush., Matsush. Mycol. Mem. 5: 9. 1987.  

*Ascomata* perithecial, mostly solitary to gregarious, non-stromatic, superficial, broadly pyriform, not collapsing when dry, orange to sienna, turning blood red in KOH, pigment dissolving in lactic acid, broadly rounded to flattened papilla, smooth-walled, lacking hairs and appendages. *Ascomatal wall* of two regions: outer region of thick-walled, pigmented cells forming a *textura angularis*; inner region of elongate, hyaline, thin-walled cells, becoming thinner towards the centrum. *Asci* narrowly clavate, apex simple, 8-spored, lacking an apical ring, irregularly multiseriate. *Ascospores* fusoid, 3-septate, hyaline, smooth or finely spinulose. *Sporodochia* not formed. *Conidiophores* simple, mononematous, straight to flexuous, hyaline, septate, unbranched or rarely branched, terminating in a single phialide or reduced to lateral phialides. *Conidiogenous cells* monophialidic, cylindrical or slightly swollen, tapering towards the apex, with periclinal thickening and flared collarettes. *Microconidia* not formed. *Macroconidia* cylindrical to slightly fusoid, straight to slightly curved, 3–6-septate, with rounded ends. *Chlamydospores* unknown.

[Description adapted from Rossman (1983) and Salgado-Salazar *et al.* (2016)].  

*Diagnostic features*: Orange to sienna, smooth-walled perithecia with broadly rounded to flattened papilla producing narrowly clavate asci bearing fusoid, 3-septate phragmo-ascospores and cylindrocarpon-like asexual morph.  

## *Fusarium* and allied genera: list of accepted names

The following nomenclator lists names that have been introduced in *Fusarium* up to January 2021, as well as their current status (with accepted names indicated in bold and underlined for easier recognition). Where type specimens have been located, these details, as well as any ex-type cultures and diagnostic DNA barcode data are provided, along with notes regarding potential synonymy. This list will be updated and republished at regular intervals, and will form the basis for a monograph of *Fusarium* and allied genera that will be freely available on www.Fusarium.org.  

***aberrans Fusarium*** J.W. Xia *et al.*, Persoonia 43: 192. 2019.

*Holotypus*: CBS H-24050.

*Ex-type culture*: CBS 131385.

*Type locality*: **Australia**, Northern Territory, Roper River area.

*Type substrate*: Stem of *Oryza australiensis.*

*Descriptions and illustrations*: See Xia *et al.* (2019).

*Diagnostic DNA barcodes*: *rpb2*: MN170378; *tef1*: MN170445.  

*acaciae Fusarium* Berl. & Voglino, Syll. Fung., Addit. I–IV: 201. 1886, *nom. illegit*., Art. 53.1.

(See *Fusarium acaciae* Cooke & Harkn.)  

*acaciae Fusarium* Cooke & Harkn., Grevillea 12: 96. 1884.

*Synonyms*: ?*Fusarium acaciae* Berl. & Voglino, Syll. Fung., Addit. I–IV: 201. 1886, *nom. illegit*., Art. 53.1.

?*Fusarium acaciae* Sacc., Syll. Fung. 9: 958. 1891, *nom. illegit*., Art. 53.1.

(See ***Fusarium lateritium***)

*Holotypus*: ?BPI 451718.

*Type locality*: **USA**, California.

*Type substrate*: Stem of *Acacia* sp*.*

*Note*: Synonym *fide*Wollenweber & Reinking (1935).  

*acaciae Fusarium* Sacc., Syll. Fung. 9: 958. 1891, *nom. illegit.*, Art. 53.1.

(See *Fusarium acaciae* Cooke & Harkn.)  

***acaciae-mearnsii Fusarium*** O'Donnell *et al.*, Fungal Genet. Biol. 41: 619. 2004.

*Holotypus*: BPI 843477.

*Ex-type culture*: CBS 110254 = MRC 5120 = NRRL 25754.

*Type locality*: **South Africa**, KwaZulu-Natal, Pietermaritzburg.

*Type substrate*: *Acacia mearnsii.*

*Descriptions and illustrations*: See O'Donnell *et al.* (2004).

*Diagnostic DNA barcodes*: *rpb1*: JAAWUD010000100; *rpb2*: JAAWUD010000080; *tef1*: AF212448.  

*acicola Fusarium* Bres., in Strasser, Verh. Zool.-Bot. Ges. Wien 60: 328. 1910.

*Holotypus*: Not located.

*Type locality*: **Austria**, Sonntagberg.

*Type substrate*: Rotting needles of *Pinus* sp*.*

*Descriptions and illustrations*: See Strasser (1910).

*Note*: Status unclear. Not *Fusarium fide*Wollenweber & Reinking (1935).  

*acremoniopsis Fusarium* Vincens, Bull. Soc. Mycol. France 31: 26. 1915.

(See ***Fusarium larvarum***)

*Holotypus*: ?PC.

*Type locality*: **Brazil**, Pará, Belém.

*Type substrate*: *Agrotis* sp. (cutworm)*.*

*Descriptions and illustrations*: See Vincens (1915).

*Note*: Synonym *fide*Wollenweber & Reinking (1935).  

*acridiorum Fusarium* (Trab.) Brongn. & Delacr., Bull. Séances Soc. Natl. Agric. France 51: 631. 1891.

***Trichothecium acridiorum*** (Trab.) Madelin, Trans. Brit. Mycol. Soc. 49: 284. 1966.

*Basionym*: *Botrytis acridiorum* Trab., Compt. Rend. Hebd. Séances Acad. Sci. 112: 1383. 1891.

*Synonym*: *Lachnidium acridiorum* Giard, Compt. Rend. Hebd. Séances Acad. Sci. 112: 1520. 1891.

*Holotypus*: Not located.

*Type locality*: **Algeria**.

*Type substrate*: *Acrididae* (locust)*.*

*Description and illustrations*: See Madelin (1966).  

***acuminatum Fusarium*** Ellis & Everh., Proc. Acad. Nat. Sci. Philadelphia 47: 441. 1895.

*Synonyms*: *Microcera acuminata* (Ellis & Everh.) Höhn., in Weese, Sitzungsber. Akad. Wiss. Wien, Math.-Naturwiss. Kl., Abt. 1. 128: 729. 1919.

*Fusarium scirpi* var. *acuminatum* (Ellis & Everh.) Wollenw., Fusaria Autogr. Delin. 3: 930. 1930.

*Fusarium scirpi* subsp. *acuminatum* (Ellis & Everh.) Raillo, Fungi of the Genus Fusarium: 177. 1950.

*Fusarium gibbosum* var. *acuminatum* (Ellis & Everh.) Bilaĭ, Mikrobiol. Zhurn. 49: 6. 1987.

?*Selenosporium hippocastani* Corda, Icon. Fung. 2: 7. 1838.

*Fusarium hippocastani* (Corda) Sacc., Syll. Fung. 4: 703. 1886.

*Fusarium erubescens* Appel & Oven, Landwirtsch. Jahrb. 1905, *nom. illegit.*, Art. 53.1.

*Fusarium caudatum* Wollenw., J. Agric. Res. 2: 262. 1914.

*Fusarium scirpi* var. *caudatum* (Wollenw.) Wollenw., Fusaria Autogr. Delin. 3: 934 & 935. 1930.

*Fusarium equiseti* var. *caudatum* (Wollenw.) Joffe, Mycopathol. Mycol. Appl. 53(1–4): 220. 1974.

*Fusarium arcuosporum* Sherb., Mem. Cornell Univ. Agric. Exp. Sta. 6: 186. 1915.

*Fusarium ferruginosum* Sherb., Mem. Cornell Univ. Agric. Exp. Sta. 6: 190. 1915.

*Fusarium sanguineum* Sherb., Mem. Cornell Univ. Agric. Exp. Sta. 6: 193. 1915.

*Fusarium lanceolatum* O.A. Pratt, J. Agric. Res. 13: 83. 1918.

*Fusarium pseudoeffusum* Murashk., Arb. Landwirtsch. Akad. Omsk. 3: 19. 1924.

*Fusarium moronei* Curzi, Revista Biol. (Lisbon)10: 141. 1928.

*Fusarium russianum* Manns, Bull. N. Dakota Agric. Exp. Sta. 259: 34. 1932.

*Gibberella acuminata* Wollenw., *Fusarien*: 68. 1935.

*Spermospora oryza* M. Rao, Sci. & Cult. 32: 94. 1966.

*Gibberella acuminata* C. Booth, The Genus Fusarium: 161. 1971, *nom. illegit.*, Art. 53.1.

*Holotypus*: NY00928689.

*Type locality*: **USA**, New York, Geneva.

*Type substrate*: *Solanum tuberosum.*

*Descriptions and illustrations*: See Sherbakoff (1915), Booth (1971), Gerlach & Nirenberg (1982), Burgess & Summerell (2000) and Leslie & Summerell (2006).

*Notes*: *Fusarium acuminatum* is an established name in the *Fusarium* literature, but it lacks living type material to confirm its taxonomic position. Although an older epithet, based on *Selenosporium hippocastani,* could be used, we refrain from providing a new combination for this well-known species due to a lack of DNA-based evidence to support this combination. Moreover, Holubová-Jechová *et al.* (1994) could not locate any holotype material for *S. hippocastani*, abstaining from introducing a neotype, which they argued would cause nomenclatural instability, a view we fully support.  

***acutatum Fusarium*** Nirenberg & O'Donnell, Persoonia 46: 144. 2021.

*Synonym*: *Fusarium acutatum* Nirenberg & O'Donnell, Mycologia 90: 435. 1998, *nom. inval*., Art. 40.1.

*Holotypus*: B 70 0001695.

*Ex-type culture*: BBA 69580 = CBS 402.97 = FRC 0-1117 = IMI 376110 = NRRL 13309.

*Type locality*: **India**.

*Type substrate*: Unknown.

*Descriptions and illustrations*: See Nirenberg & O'Donnell (1998) and Yilmaz *et al.* (2021).

*Diagnostic DNA barcodes*: *rpb1*: MT010947; *rpb2*: KT154005; *tef1*: KR071754.  

*acutisporum Fusarium* (Sand.-Den. & Crous) O'Donnell *et al.*, Index Fungorum 440: 1. 2020.

***Neocosmospora acutispora*** Sand.-Den. & Crous, Persoonia 43: 108. 2019.

*Holotypus*: CBS H-23969.

*Ex-type culture*: BBA 62213 = CBS 145461 = NRRL 22574.

*Type locality*: **Guatemala**.

*Type substrate*: *Coffea arabica*

*Descriptions and illustrations*: See Sandoval-Denis *et al.* (2019).

*Diagnostic DNA barcodes*: *rpb1*: MW834210; *rpb2*: LR583814; *tef1*: LR583593.  

*aderholdii Fusarium* Osterw., Bericht Schweiz. Versuchsanst. Obst-, Wein- und Gartenbau 1913/14: 519. 1915.

***Ilyonectria destructans*** (Zinssm.) Rossman *et al.*, Stud. Mycol. 80: 217. 2015.

*Basionym*: *Ramularia destructans* Zinssm., Phytopathology 8: 570. 1918.

*Synonyms*: *Cylindrocarpon destructans* (Zinssm.) Scholten, Netherlands J. Plant Pathol. 70 suppl. (2): 9. 1964.

*Fusarium polymorphum* Marchal, Bull. Soc. Roy. Bot. Belgique 34: 145. 1895, *nom. illegit.*, Art. 53.1.

*Cylindrocarpon radicicola* Wollenw., Fusaria Autogr. Delin. 2: 651. 1924.

*Nectria radicicola* Gerlach & L. Nilsson, Phytopathol. Z. 48: 255. 1963.

*Neonectria radicicola* (Gerlach & L. Nilsson) Mantiri & Samuels, Canad. J. Bot. 79: 339. 2001.

*Ilyonectria radicicola* (Gerlach & L. Nilsson) P. Chaverri & C.G. Salgado, Stud. Mycol. 68: 71. 2011.

?*Fusarium rhizogenum* Aderh., Centralbl. Bacteriol. Parasitenk., 2. Abth., 6: 623. 1900, *nom. illegit.*, Art. 53.1.

?*Septocylindrium radicicola* Aderh., Centralbl. Bakteriol. Parasitenk., 2. Abth., 6: 623, 1900, nom. illegit., Art. 53.1.

?*Septocylindrium aderholdii* Sacc & P. Syd., Syll. Fung. 16: 1048. 1902.

*Holotypus*: Not located*.*

*Type locality*: **Germany**.

*Type substrate*: Unknown.

*Notes*: Synonymy *fide*Wollenweber & Reinking (1935). Although older epithets are available for *Ilyonectria destructans*, we refrain from providing a new combination for this well-known species due to a lack of DNA-based evidence to support this combination.  

*adesmiae Fusarium* Henn., Hedwigia 36: 246. 1897.

*Synonym*: *Ramularia adesmiae* (Henn.) Wollenw., Fusaria Autogr. Delin. 1: 466. 1916.

*Holotypus*: In B *fide*Hein (1988).

*Type locality*: **Chile**, Bío-Bío Province.

*Type substrate*: *Adesmia* sp.

*Note*: Status unclear, not *Ramularia fide*Braun (1998).  

*aduncisporum Fusarium* Weimer & Harter, J. Agric. Res. 32: 312. 1926.

(See *Fusarium solani*)

*Lectotypus*: BPI 451321, designated in Sandoval-Denis *et al.* (2019).

*Lectotype locality*: **USA**, California, Ventura.

*Lectotype substrate*: Stems of *Melilotus alba*.

*Note*: Synonym *fide*Wollenweber & Reinking (1935).  

*aecidii-tussilaginis Fusarium* Allesch., Ber. Bot. Vereines Landshut 12: 131. 1892.

(See ***Fusarium avenaceum***)

*Holotypus*: In M.

*Type locality*: **Germany**, Oberammergau.

*Type substrate*: *Aecidium tussilaginis*.

*Note*: Synonym *fide*Wollenweber & Reinking (1935).  

*aeruginosum Fusarium* Delacr., Bull. Soc. Mycol. France 7: 110. 1891.

(See ***Fusarium caeruleum***)

*Lectotypus* (*hic designatus*, MBT 10000648): **France**, Paris, from *Solanum tuberosum*, April 1891, G. Delacroix, Bull. Soc. Mycol. France 7: pl. VIII, fig. h.

*Notes*: Synonym *fide*Wollenweber & Reinking (1935). No holotype specimen could be located and therefore an illustration is designated as lectotype.  

***aethiopicum Fusarium*** O'Donnell *et al.*, Fungal Genet. Biol. 45: 1521. 2008.

*Holotypus*: BPI 878409.

*Ex-type culture*: CBS 122858 = NRRL 46726.

*Type locality*: **Ethiopia**, Bure district, west Gojjam zone of Amhara region.

*Type substrate*: *Triticum aestivum.*

*Descriptions and illustrations*: See O'Donnell *et al.* (2008b).

*Diagnostic DNA barcodes*: *rpb1*: MW233298; *rpb2*: MW233470; *tef1*: FJ240298.  

*affine Fusarium* Fautrey & Lambotte, Rev. Mycol. (Toulouse) 18: 68. 1896.

*Syntypes*: ILL00221136 (Roumeguère, Fungi Sel. Gall. Exs. no. 6927) & ILL00221137 (Roumeguère, Fungi Sel. Gall. Exs. no. 6928).

*Type locality*: **France**.

*Type substrate*: *Solanum tuberosum.*

*Notes*: Booth (1971) examined the *exsiccatae* (Fung. Sel. Gall. Exs., No. 6927 & 6928) of *F. affine* and found that one part (no. 6927) is *F. solani* and the other part (no. 6928) represented another fungus that was interpreted as *Hymenula affinis* by Wollenweber (1916–1935). Booth (1971) indicated that *F. affine* might be a possible synonym of *F. tabacinum*, which is now regarded as *Plectosphaerella cucumerina* (Palm *et al.* 1995, Giraldo & Crous 2019). However, both Gams & Gerlach (1968) and Palm *et al.* (1995) considered *F. affine* as a misapplied synonym of *P. cucumerina*. Sherbakoff (1915) also treated the fungus as *F. affine*, which was later reinterpreted as *Septomyxa affine* by Wollenweber (1916–1935). Therefore, the current status of *F. affine* is uncertain and requires further investigation.  

***agapanthi Fusarium*** O'Donnell *et al.*, Mycologia 108: 987. 2016.

*Holotypus*: VPRI 41777.

*Ex-type culture*: NRRL 54463 = VPRI 41777.

*Type locality*: **Australia**, Victoria, Melbourne, Royal Botanic Gardens.

*Type substrate*: *Agapanthus praecox.*

*Descriptions and illustrations*: See Edwards *et al.* (2016).

*Diagnostic DNA barcodes*: *rpb1*: KU900620; *rpb2*: KU900625; *tef1*: KU900630.  

*agaricorum Fusarium* Sarrazin, Rev. Mycol. (Toulouse) 9: 170. 1887.

*Lectotypus* (*hic designatus*, MBT 10000649): **France**, on the cap of *Psalliota campestris* (syn. *Agaricus campestris*), 1887, F. Sarrazin, ILL00218415 (Roumeguère, Fungi Sel. Gall. Exs. no. 4298).

*Notes*: Status unclear. Not *Fusarium fide*Wollenweber & Reinking (1935).  

*ailanthinum Fusarium* Speg., Anales Mus. Nac. Hist. Nat. Buenos Aires 6: 350. 1899.

(See ***Fusarium lateritium***)

*Holotypus*: In LPS (Fungi Argent. n.v.c. #864).

*Type locality*: **Argentina**.

*Type substrate*: Trunk and branches of *Ailanthus glandulosa.*

*Note*: Synonym *fide*Wollenweber & Reinking (1935).  

*alabamense Fusarium* Sacc., Syll. Fung. 4: 722. 1886, nom. illegit., Art. 52.1.

*Synonym*: *Fusarium erubescens* Berk. & M.A. Curtis, Grevillea 3: 98. 1875.

*Holotypus*: ?K(M).

*Type locality*: **USA**, Alabama, Beaumont.

*Type substrate*: Bark.

*Notes*: Status unclear. Not *Fusarium fide*Wollenweber & Reinking (1935).  

*albedinis Fusarium* Kill. & Maire ex Malençon, Compt. Rend. Hebd. Séances Acad. Sci. 198: 1261. 1934, *nom. inval*., Art. 6.10.

*Synonym*: *Cylindrophora albedinis* Kill. & Maire, Bull. Soc. Hist. Nat. Afrique N. 21: 97. 1930, *nom. inval*., Art. 36.1.

(See ***Fusarium oxysporum***)

*Authentic material*: Not located.

*Original locality*: Indicated as ‘oasis in Sahara’.

*Original substrate*: Dead trunk and leaf of *Phoenix dactylifera*.

*Note*: Synonym *fide*Booth (1971).  

*albertii Fusarium* Roum., Fungi Sel. Gall. Exs., Cent. 19: no. 1867. 1881, *nom. inval*., Art. 38.1(a).

(See ***Fusarium lateritium***)

*Authentic material*: BR5020140140720.

*Original locality*: **France**.

*Original substrate*: Petiole of *Ziziphus volubilis*.

*Note*: Synonym *fide*Wollenweber & Reinking (1935).  

*albidoviolaceum Fusarium* Dasz. (as ‘*albido-violaceum*’), Bull. Soc. Bot. Genève, sér. 2, 4: 293. 1912.

(See ***Fusarium oxysporum***)

*Lectotypus* (*hic designatus*, MBT 10000650): **Switzerland**, Geneva, from soil, 1912, W. Daszewska, Bull. Soc. Bot. Genève, 2 sér. 4: 292, fig. 15.

*Notes*: Wollenweber (1916–1935; Fusaria Autogr. Delin. 1: 361) indicated that the living ex-type culture was lodged in the laboratory of W.C. Scholten in Amsterdam, which in turn has been accessioned into the CBS. However, no record or culture can be located in the CBS collection. Therefore, an illustration accompanying the original protologue is designated as lectotype here.  

*albidum Fusarium* (Rossman) O'Donnell & Geiser, Phytopathology 103: 404. 2013.

***Luteonectria albida*** (Rossman) Sand.-Den. & L. Lombard, Stud. Mycol. 98 (no. 100116): 60. 2021.

*Basionym*: *Nectria albida* Rossman, Mycol. Pap. 150: 79. 1983.

*Synonym*: *Albonectria albida* (Rossman) Guu & Y.M. Ju, Bot. Stud. (Taipei) 48: 189. 2007.

*Holotypus*: CUP-MJ 942.

*Ex-type culture*: ATCC 44543 = BBA 67603 = CTR 71-110 = NRRL 13950 = NRRL 22152.

*Type locality*: **Jamaica**, Hanover Parish, Dolphin Head Mountain, near Askenish.

*Type substrate*: Erumpent through thin bark of woody stem*.*

*Diagnostic DNA barcode*: *rpb1*: JX171492; *rpb2*: HQ897738; *tef1*: MW834283.  

*albiziae Fusarium* Woron., Vestn. Tiflissk. Bot Sada 48: 34. 1920.

(See *Fusarium merismoides*)

*Syntypes*: BPI 451733, BPI 451734 & CUP-017160.

*Type locality*: **Georgia**, Batumi, Adjara.

*Type substrate*: *Albizia julibrissin.*

*Notes*: Synonym *fide*Wollenweber & Reinking (1935). Lectotypification requires further investigation of the syntypes.  

*albocarneum Fusarium* (Cooke & Harkn.) Sacc., Syll. Fung. 4: 720. 1886.

*Basionym*: *Fusidium albocarneum* Cooke & Harkn., Grevillea 9: 129. 1881.

*Syntype*: BPI 408577.

*Type locality*: **USA**, California, San Francisco, San Francisco Odd Fellows Cemetery.

*Type substrate*: *Eucalyptus* sp.

*Notes*: The generic name *Cylindrocarpon* (= *Neonectria*; Rossman *et al.* 2013) was conserved over *Fusidium*, making the latter generic name a *nom. rej.* (Art. 14.1, 14.6 & 14.7). Therefore, *Fusidium albocarneum* should be transferred to *Neonectria* after further investigation. Lectotypification requires further investigation of the syntype.  

*albosuccineum Fusarium* (Pat.) O'Donnell & Geiser, Phytopathology 103: 404. 2013.

***Albonectria albosuccinea*** (Pat.) Rossman & Samuels, Stud. Mycol. 42: 107. 1999.

*Basionym*: *Calonectria albosuccinea* Pat., Bull. Soc. Mycol. France 8: 132. 1892.

*Synonyms*: *Nectria albosuccinea* (Pat.) Rossman, Mycotaxon 8: 487. 1979.

*Calonectria ecuadorica* Petrak, Sydowia 4: 463. 1950.

*Holotypus*: In FH *fide*Rossman (1983).

*Type locality*: **Ecuador**, Puente Chimbo.

*Type substrate*: Bark.  

*album Fusarium* Sacc., Michelia 1: 82. 1877.

***Neonectria punicea*** (J.C. Schmidt) Castl. & Rossman, Canad. J. Bot. 84: 1425. 2006.

*Basionym*: *Sphaeria punicea* J.C. Schmidt, in Schmidt & Kunze, Mykol. Hefte 1: 61. 1817.

*Synonyms*: *Nectria punicea* (J.C. Schmidt) Fr., Summa Veg. Scand. 2: 387. 1849.

*Cucurbitaria punicea* (J.C. Schmidt) Kuntze, Revis. Gen. Pl. 3: 461. 1898.

*Cylindrocarpon album* (Sacc.) Wollenw., Fusaria Autogr. Delin. 1: no. 473. 1916.

*Nectria punicea f. ilicicola* Rehm, Ascomyceten: no. 337. 1876.

*Nectria punicea* var. *ilicis* C. Booth, Mycol. Pap. 73: 54. 1959.

*Cylindrocarpon album* var. *majus* Wollenw., Z. Parasitenk. (Berlin) 1: 154. 1928.

*Fusarium album* var. *abietinum* Beeli, Bull. Soc. Roy. Bot. Belgique 62: 131. 1930.

*Holotypus*: Not located.

*Type locality*: **Italy**.

*Type substrate*: Bark of *Pinus* sp.

*Note*: Synonym *fide*Wollenweber & Reinking (1935).  

*aleurinum Fusarium* Ellis & Everh., Bull. Torrey Bot. Club 24: 476. 1897.

(See ***Fusarium avenaceum***)

*Syntypes*: In BPI, BRU, CLEMS, CUP, F, FLAS, ILL, ILLS, ISC, MICH, MSC, MU, NCU, NEB, OSC, PH, PUL, UC, WIS & WSP.

*Type locality*: **USA**, West Virginia, Fayette County Nuttallburg, south of Edmond.

*Type substrate*: Wheat flour that had been on the ground for four months.

*Notes*: Synonym *fide*Wollenweber & Reinking (1935). Lectotypification requires further investigation of the syntypes.  

*aleyrodis Fusarium* Petch, Trans. Brit. Mycol. Soc. 7: 164. 1921.

*Lectotypus* (*hic designatus*, MBT 10000651): **USA**, Florida, Sutherland, from *Aleyrodes citri*, 13 Sep. 1907, F. Wills, in Petch 1921, Trans. Brit. Mycol. Soc. 7, pl. V, fig. 12.

*Notes*: Wollenweber & Reinking (1935) considered this species as a synonym of *F. scirpi*. However, based on the descriptions and illustrations provided by Fawcett (1908) and Petch (1920), this species belongs to the genus *Microcera*, which is also in agreement with its aetiology. Therefore, a new combination will presumably be required after further investigation.  

***algeriense Fusarium*** Laraba & O'Donnell, Mycologia 109: 944. 2017.

*Holotypus*: BPI 910347.

*Ex-type culture*: CBS 142638 = NRRL 66647.

*Type locality*: **Algeria**, Guelma Province, Djeballah Khemissi.

*Type substrate*: *Triticum durum.*

*Descriptions and illustrations*: See Laraba *et al.* (2017).

*Diagnostic DNA barcodes*: *rpb1*: MF120488; *rpb2*: MF120499; *tef1*: MF120510.  

***alkanophilum Fusarium*** Palacios-Prü & V. Marcano, Rev. Ecol. Latinoamer. 8: 5. 2001.

*Holotypus*: EMC, Palacios-Prü, 3 April 1998.

*Type locality*: **Venezuela**, Merida State, south of Sierra La Culata, Valle de San Javier, Los Pinos.

*Type substrate*: Beetle immersed in kerosene.

*Descriptions and illustrations*: See Marcano *et al.* (2001).

*Note*: No living type material could be located.  

*allescheri Fusarium* Sacc. & P. Syd., Syll. Fung. 14: 1128. 1899.

*Replaced synonym*: *Fusarium glandicola* Allesch., Ber. Bot. Vereines Landshut 12: 130. 1892, *nom. illegit.*, Art. 53.1; *non* Cooke & W.R. Gerard 1878.

(See *Fusarium melanochlorum*)

*Holotypus*: In M.

*Type locality*: **Germany**, München.

*Type substrate*: *Quercus pedunculata*.

*Note*: Synonym *fide*Wollenweber & Reinking (1935).  

*allescherianum Fusarium* Henn., Verh. Bot. Vereins Prov. Brandenburg 40: 175. 1899.

*Synonym*s: *Gloeosporium allescherianum* (Henn.) Wollenw., Fusaria Autogr. Delin. 1: 495. 1916.

?*Fusarium personatum* Cooke, in Harkness, Grevillea 7: 12. 1878.

*Holotypus*: In B *fide*Hein (1988).

*Type locality*: **Germany**.

*Type substrate*: Leaves of *Ocotea foetens*.

*Notes*: Status unclear. The taxonomic status of *Gloeosporium allescherianum* is questionable. Furthermore, there is no DNA-based evidence linking *F. allescherianum* to *F. personatum* although Wollenweber & Reinking (1935) considered them both synonyms under *G. allescherianum*.  

*allii-sativi Fusarium* Allesch., Ber. Bot. Vereines Landshut 12: 131. 1892.

(See *Fusarium solani*)

*Holotypus*: In M.

*Type locality*: **Germany**, Unterammergau.

*Type substrate*: *Allium sativum*.  

*alluviale Fusarium* Wollenw. & Reinking, Phytopathology 15: 167. 1925.

(See *Fusarium solani*)

*Holotypus*: Not located.

*Type locality*: **Honduras**.

*Type substrate*: Alluvial soil.  

*aloes Fusarium* Kalchbr. & Cooke, Grevillea 9: 23. 1880.

(See ***Fusarium scirpi***)

*Holotypus*: ?K(M).

*Type locality*: **South Africa**, Eastern Cape Province, Somerset East.

*Type substrate*: *Aloe arborescens*.

*Note*: Synonym *fide*Wollenweber & Reinking (1935).  

*ambrosium Fusarium* (Gadd & Loos) Agnihothr. & Nirenberg, Stud. Mycol. 32: 98. 1990.

***Neocosmospora ambrosia*** (Gadd & Loos) L. Lombard & Crous, Stud. Mycol. 80: 227. 2015.

*Basionym*: *Monacrosporium ambrosium* Gadd & Loos, Trans. Brit. Mycol. Soc. 31: 17. 1947.

*Synonyms*: *Dactylella ambrosia* (Gadd & Loos) K.Q. Zhang *et al.*, Mycosystema 7: 112. 1995.

*Fusarium bugnicourtii* Brayford, Trans. Brit. Mycol. Soc. 89: 350. 1987.

*Lectotypus*: Trans. Brit. Mycol. Soc. 31: 16, Text-fig. 5. 1947, designated by Aoki *et al.* (2018).

*Lectotype locality*: **Sri Lanka**.

*Lectotype substrate*: Gallery of *Euwallacea fornicatus* infesting *Camellia sinensis*.

*Epitypus*: BPI 910524, designated by Aoki *et al.* (2018).

*Ex-epitype culture*: BBA 65390 = CBS 571.94 = NRRL 22346 = MAFF 246287.

*Epitype locality*: **India**, Upasi Tea Institute.

*Epitype substrate*: Gallery of *Euwallacea fornicatus* infesting *Camellia sinensis*.

*Diagnostic DNA barcodes*: *rpb1*: KC691587; *rpb2*: EU329503; *tef1*: FJ240350  

*amenti Fusarium* Rostr., Bot. Tidsskr. 14: 240. 1885.

(See ***Fusarium avenaceum***)

*Holotypus*: F-604398 in UPS.

*Type locality*: **Denmark**, Fyn, Holmdrup.

*Type substrate*: *Salix cinerea.*

*Note*: Synonym *fide*Wollenweber & Reinking (1935).  

*amentorum Fusarium* Lacroix, Fl. Maine-et-Loire 2 (Suppl.): [1]. 1854.

(See ***Fusarium avenaceum***)

*Lectotypus* (*hic designatus*, MBT 10000652): **France**, St. Romain-sur-Vienne, from *Salix cinerea*, date unknown*,* J.B.H.J. Desmazières, BR5020140143752.

*Note*: Synonym *fide*Wollenweber & Reinking (1935).  

*amethysteum Fusarium* P. Crouan & H. Crouan, Fl. Finistère: 14. 1867.

*Holotypus*: Not located.  

*Type locality*: **France**.

*Type substrate*: Dead stem of *Urtica* sp.

*Notes*: Status unclear. Not *Fusarium fide*Wollenweber & Reinking (1935).  

*ampelodesmi Fusarium* Fautrey & Roum., in Roumeguère, Rev. Mycol. (Toulouse) 13: 82. 1891.

(See ***Fusarium reticulatum***)

*Syntype*: ILL00219841 (Roumeguère, Fungi Sel. Gall. Exs. no. 5687).

*Type locality*: **France**, Jardin de Noidan.

*Type substrate*: *Ampelodesmos tenax*

*Notes*: Synonym *fide*Wollenweber & Reinking (1935). Lectotypification requires further investigation of the syntype.  

*amplum Fusarium* (Sand.-Den. & Crous) O'Donnell *et al.*, Index Fungorum 440: 1. 2020.

***Neocosmospora ampla*** Sand.-Denis & Crous, Persoonia 43: 110. 2019.

*Holotypus*: CBS H-23970.

*Ex-type culture*: BBA 4170 = CBS 202.32.

*Type locality*: **German East Africa**.

*Type substrate*: *Coffea* sp*.*

*Descriptions and illustrations*: See Sandoval-Denis *et al.* (2019).

*Diagnostic DNA barcodes*: *rpb1*: MW834212; *rpb2*: LR583815; *tef1*: LR583594.  

***ananatum Fusarium*** A. Jacobs *et al.*, Fung. Biol. 114: 522. 2010.

*Holotypus*: PREM 58713.

*Ex-type culture*: CBS 118516 = CMW 18685 = FCC 2986 = MRC 8165.

*Type locality*: **South Africa**, KwaZulu-Natal Province, Hluhluwe.

*Type substrate*: *Ananas comosus.*

*Descriptions and illustrations*: See Jacobs *et al.* (2010).

*Diagnostic DNA barcodes*: *rpb1*: MT010937; *rpb2*: LT996137; *tef1*: MT010996.  

***andinum Fusarium*** Syd., Ann. Mycol. 37: 437. 1939.

*Holotypus*: S-F 45569.

*Type locality*: **Ecuador**, Tungurahua.

*Type substrate*: *Chusquea serrulata*.

*Descriptions and illustrations*: See Sydow & Sydow (1939).  

***andiyazi Fusarium*** Marasas *et al.*, Mycologia 93: 1205. 2001.

*Holotypus*: BPI 748223.

*Ex-type culture*: CBS 119857 = IMI 386078 = KSU 4804 = MRC 6122.

*Type locality*: **South Africa**, KwaZulu-Natal Province, Greytown.

*Type substrate*: Soil debris of *Sorghum bicolor.*

*Descriptions and illustrations*: See Marasas *et al.* (2001) and Leslie & Summerell (2006).

*Diagnostic DNA barcodes*: *rpb1*: LT996189; *rpb2*: LT996138; *tef1*: LT996092.  

*andropogonis Fusarium* Cooke ex Sacc., Syll. Fung. 10: 726. 1892.

*Synonyms*: *Fusisporium andropogonis* Cooke ex Thüm., Mycoth. Univ. 7: no. 676. 1877, nom. inval., Art. 38.1(a).

*Ramularia andropogonis* (Cooke ex Sacc.) Wollenw., Fusaria Autogr. Delin. 1: 469. 1916.

*Lectotypus* (*hic designatus*, MBT 10000653): **USA**, New Jersey, Newfield, from dead stem of *Andropogon virginicus*, Oct. 1874, J.B. Ellis, BR5020081431482 (Thümen, Mycoth. Univ. 7: no. 676).

*Notes*: Status unclear, not *Ramularia fide*Braun (1998). Synonym *fide*Wollenweber & Reinking (1935).  

***anguioides Fusarium*** Sherb., Mem. Cornell Univ. Agric. Exp. Sta. 6: 169. 1915.

*Typus*: ?CUP-007479.

*Type locality*: **USA**, New York, Castile.

*Type substrate*: *Solanum tuberosum.*

*Descriptions and illustrations*: See Sherbakoff (1915), Gerlach & Nirenberg (1982) and Nelson *et al.* (1995).

*Notes*: Nelson *et al.* (1995) designated BPI 72044 as neotype of *F. anguioides*, erroneously stating that no materials were available for epi- and lectotypification. However, Sherbakoff (1915) did provide an illustration with the original protologue of *F. anguioides* and placed material in CUP, as CUP-007479. Furthermore, the neotype (BPI 72044) of Nelson *et al.* (1995) originated from China and was isolated from soil in a bamboo grove. An isolate from the original locality (USA) and host (*Solanum tuberosum*) needs to be selected. Lectotypification pending study of material lodged in CUP.  

*angustum Fusarium* Sherb., Mem. Cornell Univ. Agric. Exp. Sta. 6: 203. 1915.

(See ***Fusarium oxysporum***)

*Typus*: ?CUP-007435.

*Type locality*: **USA**, New York, Ithaca.

*Type substrate*: *Solanum tuberosum.*

*Descriptions and illustrations*: See Sherbakoff (1915).

*Notes*: Synonym *fide*Wollenweber & Reinking (1935). Lectotypification pending study of material lodged in CUP.  

*anisophilum Fusarium* Picado, J. Dept. Agric. Porto Rico 16: 391. 1932.

(See ***Fusarium lateritium***)

*Holotypus*: Not located.

*Type locality*: **Costa Rica**.

*Type substrate*: Living stem of *Coffea* sp.

*Note*: Synonym *fide*Wollenweber & Reinking (1935).  

***annulatum Fusarium*** Bugnic., Rev. Gén. Bot. 59: 17. 1952.

*Holotypus*: IMI 202878.

*Ex-type culture*: BBA 63629 = CBS 258.54 = IMI 202878 = MUCL 8059 = NRRL 13619.

*Type locality*: **New Caledonia**.

*Type substrate*: Grain of *Oryza sativa.*

*Descriptions and illustrations*: See Bugnicourt (1952), Yilmaz *et al.* (2021).

*Diagnostic DNA barcodes*: *rpb1*: MT010944; *rpb2*: MT010983; *tef1*: MT010994.  

***annuumFusarium*** Leonian, Bull. New Mex. Coll. Agric. Mech. Arts 121: 9. 1919.

*Lectotypus* (*hic designatus*, MBT 10000654): **USA**, New Mexico, from *Capsicum annuum*, 1919, L.H. Leonian, In Bull. New Mex. Coll. Agric. Mech. Arts 121: 32, fig. 7.

*Notes*: No type specimen could be located. Wollenweber & Reinking (1935) mentioned this species but did not study or treat it any further. A new collection is required for epitypification from the type locality and substrate.  

*anomalum Fusarium* Berk. & M.A. Curtis, in Berkeley, Grevillea 3: 99. 1875.

*Holotypus*: ?K(M).

*Type locality*: **USA**, the New England region.

*Type substrate*: *Gleditsia* sp*.*

*Notes*: Status unclear. Not *Fusarium fide*Wollenweber & Reinking (1935).  

***anthophilum Fusarium*** (A. Braun) Wollenw., Ann. Mycol. 15: 14. 1917.

*Basionym*: *Fusisporium anthophilum* A. Braun, in Rabenhorst, Fungi Eur. Exs.: no. 1964. 1875.

*Synonyms*: *Fusarium moniliforme* var. *anthophilum* (A. Braun) Wollenw., Fusaria Autogr. Delin. 3: 975. 1930.

*Fusarium tricinctum* var. *anthophilum* (A. Braun) Bilaĭ, Fusarii (Biologija I sistematika): 251. 1955.

*Fusarium sporotrichiella* var. *anthophilum* (A. Braun) Bilaĭ, Mikrobiol. Zhurn. 49: 7. 1987.

*Fusarium sanguineum* var. *pallidius* Sherb., Mem. Cornell Univ. Agric. Exp. Sta. 6: 196. 1915.

*Fusarium wollenweberi* Raillo, Fungi of the Genus Fusarium: 189. 1950, *nom. inval*., Art. 41.1.

*Lectotypus*: Rabenhorst, Fungi Eur. Exs. no. 1964 in B, designated by Yilmaz *et al.* (2021).

*Lectotype locality*: **Germany**, Berchtesgaden.

*Lectotype substrate*: *Succisa pratensis*.

*Epitypus*: CBS 222.76 (preserved as metabolically inactive culture), designated by Yilmaz *et al.* (2021).

*Ex-epitype culture*: BBA 63270 = CBS 222.76 = IMI 196084 = IMI 202880 = NRRL 22943 = NRRL 25216.

*Epitype locality*: **Germany**, Berlin.

*Epitype substrate*: *Euphorbia pulcherrima*.

*Descriptions and illustrations*: See Wollenweber & Reinking (1935), Nirenberg (1976), Gerlach & Nirenberg (1982), Nelson *et al.* (1983) and Leslie & Summerell (2006).

*Diagnostic DNA barcodes*: *rpb1*: MW402641; *rpb2*: MW402811; *tef1*: MW402114.  

*apii Fusarium* P.E. Nelson & Sherb., Techn. Bull. Michigan Agric. Exp. Sta. 155: 42. 1937.

(See ***Fusarium oxysporum***)

*Holotypus*: Not located.

*Type locality*: **USA**.

*Type substrate*: *Apium graveolens* var*. dulce.*  

*apiogenum Fusarium* Sacc., Syll. Fung. 4: 717. 1886.

(See ***Fusarium lactis***)

*Holotypus*: Not located.

*Type locality*: **Germany**.

*Type substrate*: Rotten fruit.  

*aquaeductuum Fusarium* (Radlk. & Rabenh.) Lagerh. & Rabenh., Centralbl. Bakteriol. Parasitenk. Abth.9: 655. 1891.

***Fusicolla aquaeductuum*** (Radlk. & Rabenh.) Gräfenhan *et al.*, Stud. Mycol. 68: 100. 2011.

*Basionym*: *Selenosporium aquaeductuum* Radlk. & Rabenh., Kunst- und Gewerbeblatt des Polytechnischen Vereins des Königreichs Bayern 41(1): 10. 1863.

*Synonyms*: *Fusisporium moschatum* Kitasato, Centralbl. Bakteriol. Parasitenk., 1. Abth. 5: 368. 1889.

*Fusarium moschatum* (Kitasato) Sacc., Syll. Fung. 10: 729. 1892.

*Fusarium magnusianum* Allesch., Fungi Bav. no. 400. 1895.

*Fusarium aquaeductuum* var. *pusillum* Wollenw., Ann. Mycol. 15: 53. 1917.

*Fusarium aquaeductuum* var. *volutum* Wollenw., Ann. Mycol. 15: 53. 1917.

*Fusarium aquaeductuum* var. *elongatum* Wollenw., Fusaria Autogr. Delin. 3: 847. 1930.

*Fusarium aquaeductuum* var. *majus* Wollenw., Fusaria Autogr. Delin. 3: 845. 1930.

*Fusarium bicellulare* Kirschst., Hedwigia 80: 136. 1941.

*Lectotypus*: B 700014034, designated in Gräfenhan *et al.* (2011).

*Lectotype locality*: **Germany**, Bayern, München, Gasteigberg.

*Lectotype substrate*: Water in water fountain.

*Epitypus*: BBA 64559, designated in Gräfenhan *et al.* (2011).

*Ex-epitype culture*: BBA 64559 = CBS 837.85 = NRRL 20865 = NRRL 37595.

*Epitype locality*: **Germany**.

*Epitype substrate*: Water from plugged water tap in BBA.

*Descriptions and illustrations*: See Gerlach & Nirenberg (1982).

*Diagnostic DNA barcodes*: *rpb1*: KM232250; *rpb2*: HQ897744; *tef1*: KM231955.  

*arachnoideum Fusarium* (Corda) Sacc., Syll. Fung. 4: 721. 1886.

*Basionym*: *Fusisporium arachnoideum* Corda, Icon. Fung. 1: 11. 1837.

(See *Fusarium merismoides*)

*Typus*: In PRM.

*Type locality*: **Czech Republic**, Prague.

*Type substrate*: Soil.

*Note*: Synonym *fide*Wollenweber & Reinking (1935). Lectotypification pending study of material lodged in PRM.  

***arcuatisporum Fusarium*** M.M. Wang *et al.*, Persoonia 43: 78. 2019.

*Holotypus*: HAMS 248034.

*Ex-type culture*: CGMCC 3.19493 = LC 12147.  

*Type locality*: **China**, Hubei.

*Type substrate*: Pollen of *Brassica campestris.*

*Descriptions and illustrations*: See Wang *et al.* (2019).

*Diagnostic DNA barcodes*: *rpb1*: MK289799; *rpb2*: MK289739; *tef1*: MK289584.  

*arcuatum Fusarium* Berk. & M.A. Curtis, Grevillea 3: 99. 1875.

(See ***Fusarium avenaceum***)

*Lectotypus* (*hic designatus*, MBT 10000655): **USA**, South Carolina, *Malus pumila* (syn. *Pyrus malus*), date unknown, M.A. Curtis, PH00005557.

*Note*: Synonym *fide*Wollenweber & Reinking (1935).  

*arcuosporum Fusarium* Sherb., Mem. Cornell Univ. Agric. Exp. Sta. 6: 186. 1915.

(See ***Fusarium acuminatum***)

*Typus*: ?CUP-007477.

*Type locality*: **USA**, New York, Castile.

*Type substrate*: *Solanum tuberosum.*

*Descriptions and illustrations*: See Sherbakoff (1915).

*Notes*: Synonym *fide*Wollenweber & Reinking (1935). Lectotypification pending study of material lodged in CUP.  

*argillaceum Fusarium* (Fr.) Sacc., Syll. Fung. 4: 718. 1886.

*Basionym*: *Fusisporium argillaceum* Fr., Syst. Mycol. 3: 446. 1832.

*Synonyms*: *Fusarium solani* var. *argillaceum* (Fr.) Bilaĭ, Mikrobiol. Zhurn. 49: 7. 1987.

*Nectria solani* Reinke & Berthold, Untersuch. Bot. Lab. Univ. Göttingen 1: 39. 1879.

*Dialonectria solani* (Reinke & Berthold) Cooke, Grevillea 12: 111. 1884.

*Cucurbitaria solani* (Reinke & Berthold) Kuntze, Revis. Gen. Pl. 3: 461. 1898.

*Holotypus*: Not located.

*Type locality*: **Unknown**.

*Type substrate*: Periderm of *Cucumis* sp*.*

*Notes*: Status unclear. Requires recollection from type locality and substrate.  

*aridum Fusarium* O.A. Pratt, J. Agric. Res. 13: 89. 1918.

(See ***Fusarium sambucinum***)

*Lectotypus* (*hic designatus*, MBT 10000656): **USA**, Idaho, from soil, 1918, O.A. Pratt, in J. Agric. Res.13: 87, fig. 2Q.

*Notes*: Synonym *fide*Wollenweber & Reinking (1935). No holotype specimen could be located and therefore an illustration is designated as lectotype.  

***armeniacum Fusarium*** (G.A. Forbes *et al.*) L.W. Burgess & Summerell, ***comb. nov*.** MycoBank MB 837636.

*Basionym*: *Fusarium acuminatum* subsp. *armeniacum* G.A. Forbes *et al.*, Mycologia 85: 120. 1993.

*Holotypus*: DAR 67507.

*Ex-type culture*: ATCC 90020 = CBS 485.94 = FRC R-9335 = IMI 352099 = MRC 6230 = NRRL 26908 = NRRL 25141 = NRRL 29133.

*Type locality*: **Australia**, New South Wales, Edgeroi.

*Type substrate*: *Triticum aestivum.*

*Descriptions and illustrations*: See Burgess *et al.* (1993), Burgess & Summerell (2000) and Leslie & Summerell (2006).

*Diagnostic DNA barcodes*: *rpb1*: KT597715; *rpb2*: GQ915485; *tef1*: GQ915501.

*Notes*: When proposing *F. armeniacum*, Burgess & Summerell (2000) cited the basionym as *F. acuminatum* subsp. *armeniacum* with reference to the entire pagination of Burgess *et al.*'s (1993) paper, rather than the intended basionym alone, rendering the combination invalid (Art. 41.5, Ex. 15). Here we validate the new combination with the correct citation of the basionym.  

***arthrosporioides Fusarium*** Sherb., Mem. Cornell Univ. Agric. Exp. Sta. 6: 175. 1915.

*Typus*: ?CUP-007467.

*Type locality*: **USA**, New York, Castile.

*Type substrate*: *Solanum tuberosum.*

*Descriptions and illustrations*: See Sherbakoff (1915), Booth (1971) and Gerlach & Nirenberg (1982).

*Notes*: Synonym *fide*Wollenweber & Reinking (1935). Lecto- and epitypification pending study of material lodged in CUP.  

*arundinis Fusarium* (Corda) Sacc., Syll. Fung. 4: 724. 1886.

***Trichoderma viride*** Pers., Neues Mag. Bot. 1: 92. 1794, *nom. sanct*. [Fr., Syst. Mycol. 3: 215. 1829].

*Synonyms*: *Pyrenium lignorum* Tode, Fung. Mecklenb. Sel. 1: 33, tab. 3, fig. 29. 1790.

*Trichoderma lignorum* (Tode) Harz, Bull. Soc. Imp. Naturalistes Moscou 44: 116. 1871.

*Trichoderma viride* Schumach., Enum. Pl. 2: 235. 1803, *nom. illegit.*, Art. 53.1.

*Fusisporium arundinis* Corda, Icon. Fung. 1: 11. 1837.

*Trichoderma glaucum* E.V. Abbott, Iowa State Coll. J. Sci. 1: 27. 1927.

*Lectotypus* (*hic designatus*, MBT 10000657): **Czech Republic**, Prague, rotten leaves of reeds, 1837, A.C.J. Corda, Icon. Fung. 1, tab. II, fig. 163.

*Notes*: Synonyms *fide*Wollenweber & Reinking (1935). No holotype specimen could be located and therefore an illustration is designated as lectotype.  

*arvense Fusarium* Speg., Anales Soc. Ci. Argent. 10: 60. 1880.

(See *Fusarium merismoides*)

*Holotypus*: In LPS (Fungi Argent. pug. 2, #153).

*Type locality*: **Argentina**.

*Type substrate*: Dried fruits of *Solanum elaeagnifolium.*

*Note*: Synonym *fide*Wollenweber & Reinking (1935).  

*asclepiadeum Fusarium* Fautrey, Rev. Mycol. (Toulouse) 18: 68. 1896.

(See ***Fusarium lateritium***)

*Syntype*: ILL00221138 (Fungi Sel. Gall. Exs. #6929).

*Type locality*: **France**, Montagne de Bard.

*Type substrate*: *Vincetoxicum officinale* (syn. *V. hirundinaria*).

*Note*: Synonym *fide*Wollenweber & Reinking (1935).  

*asclerotium Fusarium* (Sherb.) Wollenw., Fusaria Autogr. Delin. 1: 364. 1916.

*Basionym*: *Fusarium oxysporum* var. *asclerotium* Sherb., Mem. Cornell Univ. Agric. Exp. Sta. 6: 222. 1915.

(See ***Fusarium oxysporum***)

*Lectotypus* (*hic designatus*, MBT 10000658): **USA**, New York, Atlanta, rotten tuber of *Solanum tuberosum*, 1915, C.D. Sherbakoff, in Mem. Cornell Univ. Agric. Exp. Sta. 6: 221, fig. 35 B–J.

*Notes*: Synonym *fide*Wollenweber & Reinking (1935). No holotype specimen could be located and therefore an illustration is designated as lectotype.  

***asiaticum Fusarium*** O'Donnell *et al.*, Fungal Genet. Biol. 41: 619. 2004.

*Holotypus*: BPI 843478.

*Ex-type culture*: CBS 110257 = FRC R-5469 = NRRL 13818.

*Type locality*: **Japan**.

*Type substrate*: *Hordeum vulgare.*

*Descriptions and illustrations*: See O'Donnell *et al.* (2004).

*Diagnostic DNA barcodes*: *rpb1*: JX171459; *rpb2*: JX171573; *tef1*: AF212451.  

*asparagi Fusarium* Briard, Rev. Mycol. (Toulouse) 12: 142. 1890.

(See ***Fusarium incarnatum***)

*Holotypus*: ?PC.

*Type locality*: **France**, Aube, Troyes.

*Type substrate*: *Asparagus* sp*.*

*Note*: Synonym *fide*Wollenweber & Reinking (1935).  

*asparagi Fusarium* Delacr., Bull. Soc. Mycol. France 6: 99. 1890, *nom. illegit.*, Art. 53.1., *non Fusarium asparagi* Briard 1890.

*Replacing synonym*: *Fusarium delacroixii* Sacc., Syll. Fung. 10: 725. 1892.

(See ***Fusarium sambucinum***)

*Notes*: Synonym *fide*Wollenweber & Reinking (1935). See *F. delacroixii* for lectotypification.  

*asperifoliorum Fusarium* (Westend.) Sacc., Syll. Fung. 4: 703. 1886.

*Basionym*: *Selenosporium asperifoliorum* Westend., Bull. Acad. Roy. Sci. Belgique, sér. 2, 11: 652. 1861.

*Holotypus*: BR5020140146784.

*Type locality*: **Belgium**, Oudenaarde.

*Type substrate*: *Borago officinalis.*

*Notes*: Status unclear. Not *Fusarium fide*Wollenweber & Reinking (1935).  

*aspidioti Fusarium* Sawada, Bot. Mag. (Tokyo) 28: 312. 1914.

(See *Fusarium larvarum*)

*Holotypus*: TNS-F-218710.

*Type locality*: **Japan**, Shizuoka.

*Type substrate*: *Quadraspidiotus perniciosus* (= *Aspidiotus perniciosus)* (San Jose scale).

*Note*: Synonym *fide*Wollenweber & Reinking (1935).  

***atrovinosum Fusarium*** L. Lombard & Crous, Fungal Syst. Evol. 4: 190. 2019.

*Holotypus*: CBS H-24015.

*Ex-type culture*: CBS 445.67 = BBA 10357 = DSM 62169 = IMI 096270 = NRRL 26852 = NRRL 26913.

*Type locality*: **Australia**.

*Type substrate*: *Triticum aestivum*.

*Descriptions and illustrations*: See Lombard *et al.* (2019a).

*Diagnostic DNA barcodes*: *rpb1*: MN120713; *rpb2*: MW928822; *tef1*: MN120752.  

*atrovirens Fusarium* (Berk.) Mussat, Syll. Fung. 15: 144. 1901, *nom. inval*., Arts. 35.1, 36.1(a), (c).

***Fusariella atrovirens*** (Berk.) Sacc., Atti Reale Ist. Veneto Sci. Lett. Arti, ser. 6, 2: 463. 1884.

*Basionym*: *Fusisporium atrovirens* Berk., in Smith, Engl. Fl. 5 (2): 351. 1836.

*Holotypus*: ?K(M).

*Type locality*: **UK**, Northamptonshire, Kings Cliffe.

*Type substrate*: *Allium* sp.

*Note*: Synonym *fide*Wollenweber & Reinking (1935).  

*aurantiacum Fusarium* Corda, in Sturm, Deutschl. Fl., 3 Abt. (Pilze Deutschl.) 2: 19. 1829.

(See ***Fusarium oxysporum***)

*Typus*: No. 156060 in PRM.

*Isotypus*: IMI 133948 (slide).

*Type locality*: **France**.

*Type substrate*: Dead branch.

*Note*: Synonym *fide*Wollenweber & Reinking (1935). Lectotypification pending study of material lodged in PRM.  

*aureum Fusarium* Corda, Icon. Fung. 1: 4. 1837.

***Hymenella aurea*** (Corda) L. Lombard, ***comb. nov*.** MycoBank MB 837637.

*Basionym*: *Fusarium aureum* Corda, Icon. Fung. 1: 4. 1837.

*Synonym*: *Hymenula aurea* (Corda) Wollenw., *Fusarien*: 319. 1935.

*Typus*: In PRM *fide*Pilat (1938).

*Type locality*: **Czech Republic**, Prague.

*Type substrate*: Rotten vegetables.

*Notes*: Wollenweber & Reinking (1935) provided a new combination for *F. aureum* in the genus *Hymenula*. However, the generic name *Hymenella* (1822) predates the generic name *Hymenula* (1828) and therefore we provide a new combination in the latter genus. Lectotypification pending study of material lodged in PRM.  

***austroafricanum Fusarium*** A. Jacobs *et al.*, Mycologia 110: 1197. 2018.

*Holotypus*: PREM 62137.

*Ex-type culture*: NRRL 66741 = PPRI 10408.

*Type locality*: **South Africa**, Eastern Cape Province, Humansdorp.

*Type substrate*: Endophyte of *Pennisetum clandestinum*.

*Descriptions and illustrations*: See Jacobs-Venter *et al.* (2018).

*Diagnostic DNA barcodes*: *rpb1*: MH742537; *rpb2*: MH742616; *tef1*: MH742687.  

***austroamericanum Fusarium*** T. Aoki *et al.*, Fungal Genet. Biol. 41: 617. 2004.

*Holotypus*: BPI 843473.

*Ex-type culture*: CBS 110244 = NRRL 2903.

*Type locality*: **Brazil**.

*Type substrate*: Polypore.

*Descriptions and illustrations*: See O'Donnell *et al.* (2004).

*Diagnostic DNA barcodes*: *rpb1*: JAAMOD010000230; *rpb2*: JAAMOD010000315; *tef1*: JAAMOD010000079.  

***avenaceum Fusarium*** (Fr.) Sacc., Syll. Fung. 4: 713. 1886.

*Basionym*: *Fusisporium avenaceum* Fr., Syst. Mycol. 2: 238. 1822, *nom. sanct*. [Fr., l.c.].

*Synonyms*: *Sarcopodium avenaceum* (Fr.) Fr., Summa Veg. Scand. 2: 472. 1849.

*Fusarium herbarum* var. *avenaceum* (Fr.) Wollenw., Fusaria Autogr. Delin. 3: 899. 1930.

*Fusarium roseum* var. *avenaceum* (Fr.) W.C. Snyder & H.N. Hansen, Amer. J. Bot. 32: 663. 1945.

*Fusisporium pyrinum* Fr., Syst. Mycol. 3: 445. 1832, *nom. sanct*. [Fr., l.c.].

*Fusarium pyrinum* (Fr.) Sacc., Syll. Fung. 4: 720. 1886, *nom. illegit.*, Art. 53.1.

*Fusarium tenue* Corda, Icon. Fung. 1: 3. 1837.

*Selenosporium tubercularioides* Corda, Icon. Fung. 1: 7. 1837.

*Fusarium tubercularioides* (Corda) Sacc., Syll. Fung. 4: 697. 1886.

*Fusarium herbarum* var. *tubercularioides* (Corda) Wollenw., Fusaria Autogr. Delin. 3: 892. 1930.

*Selenosporium herbarum* Corda, Icon. Fung. 3: 34. 1839.

*Fusarium herbarum* (Corda) Fr., Summa Veg. Scand. 2: 472. 1849.

*Fusarium graminum* var. *herbarum* (Corda) Wollenw., Fusaria Autogr. Delin. 3: 941. 1930.

*Fusarium avenaceum* var. *herbarum* (Corda) Bilaĭ, Fusarii (Biologija i sistematika): 95. 1955.

*Fusarium tritici* Liebman bis, Tidsskr. Landoekon., n.s., 2: 515. 1840.

*Fusisporium zeae* Westend., Bull. Acad. Roy. Sci. Belgique 18: 414. 1851.

*Fusarium zeae* (Westend.) Sacc., Syll. Fung. 4: 713. 1886.

*Fusarium amentorum* Lacroix, Fl. Maine-et-Loire 2 (Suppl.): [1]. 1854.

*Gloeosporium amentorum* (Lacroix) Lind, Ann. Mycol. 3: 431. 1905.

*Calogloeum amentorum* (Lacroix) Nannf., Svensk Bot. Tidskr. 25: 25. 1931.

*Platycarpium amentorum* (Lacroix) Petr., Sydowia 7: 296. 1953.

*Fusamen amentorum* (Lacroix) Arx, Verh. Kon. Akad. Wetensch., Afd. Natuurk. 51: 57. 1957.

*Fusisporium incarcerans* Berk., Intellectual Observ. 2: 11. 1863.

*Fusarium incarcerans* (Berk.) Sacc., Syll. Fung. 4: 713. 1886.

*Fusarium stercoris* Fuckel, Fungi Rhen. Exs., Suppl. Fasc. 5: no. 1921, 1867.

*Menispora penicillata* Harz, Bull. Soc. Imp. Naturalistes Moscou 44: 127. 1871.

*Fusarium penicillatum* (Harz) Sacc., Syll. Fung. 4: 710. 1886.

*Fusisporium schiedermayeri* Thüm., Fungi Austr. Exs. Cent. 1: no. 78. 1871.

*Fusarium schiedermayeri* (Thüm.) Sacc., Syll. Fung. 4: 712. 1886.

*Fusarium arcuatum* Berk. & M.A. Curtis, Grevillea 3: 99. 1875.

*Fusarium viticola* Thüm., Pilze Weinst.: 52. 1878.

*Fusarium herbarum* var. *viticola* (Thüm.) Wollenw., Fusaria Autogr. Delin. 3: 898. 1930.

*Fusarium gaudefroyanum* Sacc., Michelia 2: 132. 1880.

*Fusisporium cucurbitariae* Pat., Rev. Mycol. (Toulouse) 3: 10. 1881.

*Fusarium cucurbitariae* (Pat.) Sacc., Syll. Fung. 4: 708. 1886, *nom. illegit.*, Art. 53.1, *non Fusarium cucurbitariae* Peyronel 1918.

*Fusarium amenti* Rostr., Bot. Tidsskr. 14: 240. 1885.

*Fusarium urenidicola* Jul. Müll., Ber. Deutsch. Bot. Ges. 3: 395. 1885.

*Fusarium diffusum* Carmich., Grevillea 16: 81. 1888.

*Fusarium iridis* Oudem., Ned. Kruidk. Arch., ser. 2, 5: 515. 1889.

*Fusarium ustilaginis* Kellerm. & Swingle, Rep. (Annual) Kansas Agric. Exp. Sta. 2: 285. 1890.

*Fusarium ruberrimum* Delacr., Bull. Soc. Mycol. France 6: 139. 1890.

*Fusarium peckii* Sacc., Syll. Fung. 10: 727. 1892, *nom. illegit.*, Art. 53.1 [*pro. p*. *fide* Wollenweber & Reinking (1935)].

*Fusarium aecidii-tussilaginis* Allesch., Ber. Bot. Vereines Landshut 12: 131. 1892.

*Fusarium subviolaceum* Roum. & Fautrey, Rev. Mycol. (Toulouse) 14: 106. 1892.

*Fusarium granulosum* Ellis & Everh., Proc. Acad. Nat. Sci. Philadelphia 45: 466. 1894 [1893].

*Fusarium jungiae* Pat., Bull. Soc. Mycol. France 11: 234. 1895.

*Fusarium schnablianum* Allesch., Hedwigia 34: 289. 1895.

*Fusarium* see*menianum* Henn., Allg. Bot. Z. Syst. 2: 83. 1896.

*Fusarium aleurinum* Ellis & Everh., Bull. Torrey Bot. Club 24: 476. 1897.

*Fusarium pseudonectria* Speg., Anales Mus. Nac. Hist. Nat. Buenos Aires 6: 351. 1899.

*Fusarium limosum* Rostr., Bot. Tidsskr. 22: 263. 1899.

*Fusarium gracile* McAlpine, Proc. Linn. Soc. New South Wales 28: 554. 1903.

*Fusarium putrefaciens* Osterw., Mitth. Thurgauischen Naturf. Ges. 16: 123. 1904.

*Fusarium paspali* Henn., Bot. Jahrb. Syst. 38: 129. 1905.

*Fusarium sorghi* Henn., Ann. Mus. Congo Belge, Bot., Sér. 5, 2: 105. 1907.

*Fusarium speiseri* Lindau, Rabenh. Krypt.-Fl., ed. 2, 1(9): 580. 1909.

*Fusarium palczewskii* Jacz., Bull. Soc. Mycol. France 28: 345. 1912.

*Fusarium pseudoheterosporum* Jacz., Bull. Soc. Mycol. France 28: 347. 1912.

*Fusarium metachroum* Appel & Wollenw., Arbeiten Kaiserl. Biol. Anst. Land- Forstw. 8: 141. 1913.

*Fusarium subulatum* Appel & Wollenw., Arbeiten Kaiserl. Biol. Anst. Land- Forstw. 8: 131. 1913.

*Fusarium biforme* Sherb., Mem. Cornell Univ. Agric. Exp. Sta. 6: 166. 1915.

*Fusarium lucidum* Sherb., Mem. Cornell Univ. Agric. Exp. Sta. 6: 157. 1915.

*Fusarium metachroum* var. *minus* Sherb., Mem. Cornell Univ. Agric. Exp. Sta. 6: 145. 1915.

*Fusarium subulatum* var. *brevius* Sherb., Mem. Cornell Univ. Agric. Exp. Sta. 6: 149. 1915.

*Fusarium truncatum* Sherb., Mem. Cornell Univ. Agric. Exp. Sta. 6: 155. 1915.

*Fusarium avenaceum* var. *pallens* Wollenw., Fusaria. Autogr. Delin. 2: 575. 1924.

*Fusarium venerorum* Dounin & Goldmacher. Actes du premier Congres Internat. des Sylvicult.: 284–298. 1927.

*Fusarium herbarum* var. *volutum* Wollenw., Fusaria Autogr. Delin. 3: 893. 1930.

*Fusarium avenaceum* var. *volutum* (Wollenw.) Wollenw. & Reinking, *Fusarien*: 56. 1935.

*Fusarium avenaceum* subsp. *volutum* (Wollenw.) Raillo, Fungi of the Genus Fusarium: 188. 1950.

*Fusarium avenaceum* var. *fabae* T.F. Yu, Phytopathology 34: 392. 1944.

*Fusarium avenaceum f. fabae* (T.F. Yu) W. Yamam., Sci. Rep. Hyogo Univ. Agric., Ser. Agr. Biol. 2: 60. 1955.

*Gibberella avenacea* R.J. Cook, Phytopathology 57: 735. 1967.

*Fusarium avenaceum f*. *fabalis* X.Y. Ruan *et al.*, Acta Phytopathol. Sin. 12: 32. 1982, *nom*. *inval*., Art. 39.1.

*Fusarium avenaceum f*. *fabarum* X.Y. Ruan *et al.*, Acta Phytopathol. Sin. 12: 32. 1982, *nom*. *inval*., Art. 39.1.

*Neotypus* (*hic designatus*, MBT 10000659): **Denmark**, *Hordeum vulgare*, 3 Feb. 1986, U. Thrane, CBS 408.86 (preserved as metabolically inactive culture).

*Ex-neotype culture*: CBS 408.86 = FRC R-8510 = IMI 309354 = NRRL 26850 = NRRL 26911.

*Descriptions and illustrations*: See Wollenweber & Reinking (1935), Booth (1971), Gerlach & Nirenberg (1982), Nelson *et al.* (1983) and Leslie & Summerell (2006).

*Diagnostic DNA barcodes*: *rpb1*: MG282372; *rpb2*: MG282401; *tef1*: MW928836.

*Notes*: No type material could be located for this species. Therefore, to provide taxonomic stability to this important cereal-associated *Fusarium* species, CBS 408.86 is designated here as ex-neotype of *Fusisporium avenaceum* (= *Fusarium avenaceum*).  

***awaxy Fusarium*** Petters-Vandresen *et al.*, Persoonia 43: 363. 2019.

*Holotypus*: UPCB93138-H.

*Ex-type culture*: CMRP 4013 = LGMF1930.

*Type locality*: **Brazil**, Paraná, Guarapuava.

*Type substrate*: Rotten stalks of *Zea mays*.

*Descriptions and illustrations*: See Crous *et al.* (2019b).

*Diagnostic DNA barcodes*: *rpb2*: MK766941; *tef1*: MG839004.  

***aywerte Fusarium*** (Sangal. & L.W. Burgess) Benyon & L.W. Burgess, Mycol. Res. 104: 1171. 2000.

*Basionym*: *Fusarium avenaceum* subsp. *aywerte* Sangal. & L.W. Burgess, Mycol. Res. 99: 287. 1995.

*Holotypus*: DAR 69501.

*Ex-type culture*: F10108 = NRRL 25410.

*Type locality*: **Australia**, Northern Territory, Deep Well.

*Type substrate*: Soil.

*Descriptions and illustrations*: See Sangalang *et al.* (1995), Benyon *et al.* (2000) and Leslie & Summerell (2006).

*Diagnostic DNA barcodes*: *rpb1*: JX171513; *rpb2*: JX171626; *tef1*: JABCQV010000336.  

*azedarachinum Fusarium* (Thüm.) Sacc., Syll. Fung. 4: 704. 1886.

*Basionym*: *Fusisporium azedarachinum* Thüm., Mycoth. Univ. 14: no. 1379. 1879.

(See ***Fusarium lateritium***)

*Syntypes*: In BPI, CUP, ILL, NEB, NY, NYS PH & PUL (Mycotheca Universalis no. 1379).

*Type locality*: **USA**, South Carolina, Aiken.

*Type substrate*: *Melia azedarach.*

*Note*: Synonym *fide*Wollenweber & Reinking (1935).  

*azukiicola Fusarium* T. Aoki *et al.* (as ‘*azukicola’*), Mycologia 104: 1075. 2012.

***Neocosmospora phaseoli*** (Burkh.) L. Lombard & Crous, Stud. Mycol. 80: 227. 2015.

*Basionym*: *Fusarium martii f*. *phaseoli* Burkh., Mem. Cornell Univ. Agric. Exp. Sta. 26: 1007. 1919.

*Synonyms*: *Fusarium solani f*. *phaseoli* (Burkh.) W.C. Snyder & H.N. Hansen, Amer. J. Bot. 28: 740. 1941.

*Fusarium phaseoli* (Burkh.) T. Aoki & O'Donnell, Mycologia 95: 671. 2003.

?*Fusarium epimyces* Cooke, Grevillea 17: 15. 1888.

?*Fusarium pestis* Sorauer, Atlas Pfl.-Krankh. 4: 19, pl. XXV. 1890.

?*Fusarium martii* var. *viride* Sherb., Mem. Cornell Univ. Agric. Exp. Sta. 6: 247. 1915.

*Fusarium solani* var. *martii* Appel & Wollenw. f. 3 Snyder, Centralbl. Bakteriol. Parasitenk., 2. Abth. 91: 179. 1934.

*Fusarium solani f. sp*. *glycines* K. Roy, Pl. Dis. 81: 264. 1997.

*Fusarium tucumaniae* T. Aoki *et al.*, Mycologia 95: 664. 2003.

*Neocosmospora tucumaniae* (T. Aoki *et al.*) L. Lombard & Crous, Stud. Mycol. 80: 228. 2015.

*Fusarium virguliforme* O'Donnell & T. Aoki, Mycologia 95: 667. 2003.

*Neocosmospora virguliformis* (O'Donnell & T. Aoki) L. Lombard & Crous, Stud. Mycol. 80: 228. 2015.

*Fusarium brasiliense* T. Aoki & O'Donnell, Mycoscience 46: 166. 2005.

*Fusarium cuneirostrum* O'Donnell & T. Aoki, Mycoscience 46: 170. 2005.

*Fusarium crassistipitatum* Scandiani *et al.*, Mycoscience 53: 171. 2011.

*Holotypus*: BPI 881712.

*Ex-type culture*: MAFF 242371 = NRRL 54364.

*Type locality*: **Japan**, Hokkaido, Tokachi, Urahoro.

*Type substrate*: Roots of *Vigna angularis*.

*Descriptions and illustrations*: See Aoki *et al.* (2012b).

*Diagnostic DNA barcodes*: *rpb1*: KJ511276; *rpb2*: KJ511287; *tef1*: JQ670137.  

***babinda Fusarium*** Summerell *et al.*, Mycol. Res. 99: 1345. 1995.

*Holotypus*: DAR 70287.

*Ex-type culture*: BBA 69872 = F11217 = NRRL 25807.

*Type locality*: **Australia**, Queensland, Mount Lewis.

*Type substrate*: Plant material in soil*.*

*Descriptions and illustrations*: See Summerell *et al.* (1995) and Leslie & Summerell (2006).

*Diagnostic DNA barcode*: *rpb2*: MN534245; *tef1*: AF160305.

*Note*: The *Fusarium babinda* species complex encompassed strains incorrectly assigned to this taxon, based on reference strains of *F. babinda*, plus one unnamed *Fusarium* species (O'Donnell *et al*. 2013, Jacobs-Venter *et al*. 2019, Geiser *et al*. 2021). However, DNA sequences from diverse gene regions and phylogenetic analyses made by several authors place the ex-type of *F. babinda* (NRRL 25807) within the *Fusarium fujikuroi* species complex, as confirmed here (Fig. 8) (O'Donnell *et al*. 2000b, Lima *et al*. 2012, Herron *et al*. 2015, Crous *et al*. 2019b). Hence, the species in FBSC need to be reassessed and the species complex renamed accordingly.  

*baccharidicola Fusarium* Henn., Hedwigia 48: 20. 1908.

(See *Fusarium coccophilum*)

*Syntype*: Puttemans no. 1274 in B (syntype *fide*Hein (1988).

*Type locality*: **Brazil**, São Paulo, Pirutuba.

*Type substrate*: *Baccharis dracunculifolia* in association with cochineal (*Dactylopius coccus*)

*Note*: Synonym *fide*Wollenweber & Reinking (1935).  

*bacilligerum Fusarium* (Berk. & Broome) Sacc., Syll. Fung. 4: 711. 1886.

***Pseudocercospora bacilligera*** (Berk. & Broome) Y.L. Guo & X.J. Liu, Mycosystema 2: 229. 1989.

*Basionym*: *Fusisporium bacilligerum* Berk. & Broome, Ann. Mag. Nat. Hist., ser. 2, 7: 178. 1851.

*Synonyms*: *Cercospora bacilligera* (Berk. & Broome) Wollenw., Fusaria Autogr. Delin. 1: 450. 1916.

*Fusisporium erubescens* Durieu & Mont., Exploration scientifique de l'Algérie 1–9: 351.1848.

*Fusarium erubescens* (Durieu & Mont.) Sacc., Syll. Fung. 4: 719. 1886, *nom. illegit.*, Art. 53.1.

*Holotypus*: ?K(M).

*Type locality*: **UK**, Wiltshire, Spye Park.

*Type substrate*: Leaves of *Rhamnus alaternus*.

*Note*: Synonyms *fide*Wollenweber & Reinking (1935).  

***bactridioides Fusarium*** Wollenw., Science, N.Y. 79: 572. 1934.

*Lectotypus*: NY00936830, designated in Seifert & Gräfenhan (2012).

*Ex-type culture*: BBA 4748 = BBA 63602 = CBS 100057 = CBS 177.35 = DAOM 225115 = IMI 375323 = NRRL 22201.

*Type locality*: **USA**, Arizona, Chiricahua Mountains.

*Type substrate*: Parasitic on *Cronartium conigenum* growing on a mummified cone of *Pinus leiophylla.*

*Descriptions and illustrations*: See Wollenweber (1934), Gerlach & Nirenberg (1982) and Seifert & Gräfenhan (2012).

*Diagnostic DNA barcodes*: *rpb1*: MT010939; *rpb2*: MT010963; *tef1*: KC514053.  

*bagnisianum Fusarium* Thüm., Nuovo Giorn. Bot. Ital. 8: 252. 1876.

***Ascochyta caricis*** Fuckel, Fungi Rhen. Suppl. Fasc. 2: no. 1697. 1866.

*Synonyms*: *Phyllosticta caricis* (Fuckel) Sacc., Syll. Fung. 3: 61. 1884.

*Ascochyta caricis* Lambotte & Fautrey, Rev. Mycol. (Toulouse) 19: 141. 1897, *nom. illegit.*, Art. 53.1.

*Syntypes*: In BPI, ILL, NEB, NY, PUL & S.

*Type locality*: **Italy**, Rome, Insugherata.

*Type substrate*: *Spartium junceum.*

*Note*: Synonym *fide*Wollenweber & Reinking (1935).  

***bambusae Fusarium*** (Teng) Z.Q. Zeng & W.Y. Zhuang, Mycosystema 36: 279. 2017.

*Basionym*: *Lisea australis* var. *bambusae* Teng, Sinensia 4: 278. 1934.

*Synonym*: *Gibberella bambusae* (Teng) W.Y. Zhuang & X.M. Zhang, Nova Hedwigia 76: 195. 2003.

*Holotypus*: BPI 631179.

*Type locality*: **China**, Anhui, Huang-shan.

*Type substrate*: *Bambusoideae* culm*.*

*Descriptions and illustrations*: See Zhang & Zhuang (2003) and Zeng & Zhuang (2017a).  

***bambusicola Fusarium*** Hara, Bot. Mag. (Tokyo) 27: 255. 1913.

*Holotypus*: Not located.

*Type locality*: **Japan**, Tokyo.

*Type substrate*: *Phyllostachys reticulata.*

*Note*: Type material (specimen(s) and/or living ex-type culture) not located.  

*baptisiae Fusarium* Henn., Notizbl. Bot. Gart. Berlin 2: 383. 1899.

(See *Fusarium dimerum*)

*Holotypus*: In B *fide*Hein (1988).

*Type locality*: **Germany**, Berlin, Botanical Garden.

*Type substrate*: *Baptisia tinctoria.*

*Note*: Synonym *fide*Wollenweber & Reinking (1935).  

*barbatum Fusarium* Ellis & Everh., J. Mycol. 4: 45. 1888.

***Raffaelea barbata*** (Ellis & Everh.) D. Hawksw. (as ‘*barbatum'*’), Bull. Brit. Mus. (Nat. Hist.), Bot. 6: 272. 1979.

*Holotypus*: NY00928690.

*Type locality*: **USA**, New Jersey, Newfield.

*Type substrate*: *Usnea barbata.*  

*bartholomaei Fusarium* Peck, Bull. Torrey Bot. Club 36: 157. 1909.

***Septogloeum bartholomaei*** (Peck) Wollenw., Fusaria Autogr. Delin. 2: 638. 1924.

*Synonym*: *Trichofusarium bartholomaei* (Peck) Sacc., Syll. Fung. 22: 1473. 1913.

*Holotypus*: NYS-F-000437.

*Type locality*: **USA**, Kansas, Stockton.

*Type substrate*: *Sorghastrum nutans.*

*Note*: Synonym *fide*Wollenweber & Reinking (1935).  

*batatas Fusarium* Wollenw. (as ‘*batatae*’), J. Agric. Res. 2: 268. 1914.

(See ***Fusarium oxysporum***)

*Lectotypus* (*hic designatus*, MBT 10000660): **USA**, Washington, *Ipomoea batatas*, 1914, L.L. Harter & E.C. Field, in Wollenweber, J. Agric. Res. 2: 268, pl. XVI, figs A–E.

*Notes*: Synonym *fide*Wollenweber & Reinking (1935). As no holotype specimen could be located, an illustration accompanying the original protologue is designated here as lectotype.  

*bataticola Fusarium* (Sand.-Den. & Crous) O'Donnell *et al.*, Index Fungorum 440: 1. 2020.

***Neocosmospora bataticola*** Sand.-Den. & Crous, Persoonia 43: 112. 2019.

*Synonym*: ?*Fusarium solani f. batatas* T.T. McClure, Phytopathology 41: 75. 1951, *nom. inval*., Art. 39.1.

*Holotypus*: CBS H-23971.

*Ex-type culture*: BBA 64954 = CBS 144398 = FRC S-0567 = NRRL 22402.

*Type locality*: **USA**, North Carolina.

*Type substrate*: *Ipomoea batatas.*

*Descriptions and illustrations*: See Sandoval-Denis *et al.* (2019).

*Diagnostic DNA barcodes*: *rpb1*: MW218100; *rpb2*: FJ240381; *tef1*: AF178344.  

***begoniae Fusarium*** Nirenberg & O'Donnell, Mycologia 90: 437. 1998.

*Holotypus*: B 70 0001694.

*Ex-type culture*: BBA 67781 = CBS 403.97 = IMI 375315 = NRRL 25300.

*Type locality*: **Germany**.

*Type substrate*: *Begonia elatior.*

*Descriptions and illustrations*: See Nirenberg & O'Donnell (1998) and Leslie & Summerell (2006).

*Diagnostic DNA barcodes*: *rpb1*: JAAOAG010000375; *rpb2*: MN193886; *tef1*: AF160293.  

***beomiforme Fusarium*** P.E. Nelson *et al.*, Mycologia 79: 886. 1987.

*Holotypus*: DAOM 196987.

*Ex-type culture*: CBS 100160 = DAR 58880 = FRC M-1425 = IMI 316127 = MRC 4593 = NRRL 13606.

*Type locality*: **Australia**, Queensland, Rockhampton.

*Type substrate*: Soil*.*

*Descriptions and illustrations*: See Nelson *et al.* (1987) and Leslie & Summerell (2006).

*Diagnostic DNA barcodes*: *rpb1*: MF120485; *rpb2*: MF120496; *tef1*: MF120507.  

*berenice Fusarium* (Berk. & M.A. Curtis) Sacc., Syll. Fung. 4: 721. 1886.

***Ascocalyx berenice*** (Berk. & M.A. Curtis) Baschien, IMA Fungus 5: 93. 2014.

*Basionym*: *Fusisporium berenice* Berk. & M.A. Curtis, in Berkeley, Grevillea 3: 147. 1875.

*Synonyms*: *Bothrodiscus berenice* (Berk. & M.A. Curtis) J.W. Groves, Canad. J. Bot. 46: 1273. 1968.

*Holotypus*: ?K(M).

*Type locality*: **USA**, Massachusetts, Boston, Murray.

*Type substrate*: *Peziza* sp*.*  

*berkeleyi Fusarium* (Mont.) Berk. & Broome, North Amer. Fung.: 108. 1875.

*Basionym*: *Gloeosporium berkeleyi* Mont., Ann. Sci. Nat., Bot., sér. 3, 12: 296. 1849.

(See ***Fusarium lateritium***)

*Holotypus*: Not located.

*Type locality*: **USA**, Alabama.

*Type substrate*: Leaves of *Hibiscus syriacus.*

*Note*: Synonym *fide*Wollenweber & Reinking (1935).  

*betae Fusarium* (Desm.) Sacc., Michelia 2: 132. 1880.

***Fusicolla betae*** (Desm.) Bonord., Handb. Mykol.: 150. 1851.

*Basionym*: *Fusisporium betae* Desm., Ann. Sci. Nat., Bot., Sér. 1, 19: 436. 1830.

*Synonyms*: *Pionnotes betae* (Desm.) Sacc., Syll. Fung. 4: 726. 1886.

*Pionnotes rhizophila* var. *betae* (Desm.) De Wild. & Durieu, Prodr. Fl. Belg. 2: 367. 1898.

*Lectotypus*: K(M) 167520, designated in Gräfenhan *et al.* 2011.

*Lectotype locality*: **France**.

*Lectotype substrate*: Tuber of *Beta vulgaris.*

*Epitypus*: BBA 64317, designated in Gräfenhan *et al.* 2011.

*Ex-epitype culture*: BBA 64317.

*Epitype locality*: **Germany**, Schleswig-Holstein, Kiel.

*Epitype substrate*: *Triticum aestivum.*

*Descriptions and illustrations*: See Gräfenhan *et al.* (2011).

*Diagnostic DNA barcodes*: *rpb2*: HQ897781.  

*beticola Fusarium* A.B. Frank, Kampfbuch gegen die Schädlinge unserer Feldfrüchte: 137. 1897.

(See ***Fusarium oxysporum***)

*Holotypus*: ?NY.

*Type locality*: **Germany**.

*Type substrate*: *Beta* sp*.*

*Note*: Synonym *fide*Wollenweber & Reinking (1935).  

*biasolettianum Fusarium* Corda, Icon. Fung. 2: 3. 1838.

(See *Fusarium merismoides*)

*Typus*: PRM 155487.

*Type locality*: **Czech Republic**, near Prague.

*Type substrate*: Young stalks of *Betula* sp.

*Notes*: Synonym *fide*Wollenweber & Reinking (1935). Synonymy under *Fusicolla merismoides* still questionable (See Gräfenhan *et al.* 2011). Lectotypification pending study of material lodged in PRM.  

*bicellulare Fusarium* Kirschst., Hedwigia 80: 136. 1941.

(See *Fusarium aquaeductuum*)

*Holotypus*: B 70 0100184.

*Type locality*: **Germany**.

*Type substrate*: Parasitic on *Cryptosporella hypodermia* with *Nectria episphaeria.*

*Note*: Synonym *fide*Wollenweber & Reinking (1935).  

*biforme Fusarium* Sherb., Mem. Cornell Univ. Agric. Exp. Sta. 6: 166. 1915.

(See ***Fusarium avenaceum***)

*Lectotypus* (*hic designatus*, MBT 10000661): **USA**, Wisconsin, rotten tubers of *Solanum tuberosum*, date unknown, C.D. Sherbakoff, in Mem. Cornell Univ. Agric. Exp. Sta. 6: 166, fig. 17 (1915).

*Notes*: Synonym *fide*Wollenweber & Reinking (1935). As no holotype specimen could be located, an illustration accompanying the original protologue is designated here as lectotype.  

*bipunctatum Fusarium* Preuss, Linnaea 25: 741. 1852.

(See *Fusarium tortuosum*)

*Holotypus*: ?B.

*Type locality*: **Germany**.

*Type substrate*: Branches of unknown tree.

*Note*: Synonym *fide*Wollenweber & Reinking (1935).  

*biseptatum Fusarium* Sawada, Special Publ. Coll. Agric. Natl. Taiwan Univ. 8: 228. 1959, *nom. inval.*, Art. 39.1.

*Authentic material*: Not located.

*Original locality*: **Taiwan**.

*Original substrate*: Leaves of *Stephania cepharantha*.  

*biseptatum Fusarium* Schroers *et al.*, Mycologia 101: 59. 2009. (*non Fusarium biseptatum* Sawada 1959).

***Bisifusarium biseptatum*** (Schroers *et al.*) L. Lombard & Crous, Stud. Mycol. 80: 224. 2015.

*Holotypus*: CBS H-20126.

*Ex-type culture*: CBS 110311 = FRC E-0228 = NRRL 36184.

*Type locality*: **South Africa**, Transkei.

*Type substrate*: Soil*.*

*Descriptions and illustrations*: See Schroers *et al.* (2009).

*Diagnostic DNA barcode*: *tef1*: EU926319.  

*blackmannii Fusarium* W. Br. & A.S. Horne (as ‘*blackmanni'*’), Ann. Bot. (London) 38: 379. 1924.

(See ***Fusarium lateritium***)

*Notes*: Name withdrawn by original author (W. Brown), See Brown (1928). Synonym *fide* Wollenweber & Reinking (1935).  

*blasticola Fusarium* Rostr. (as ‘*blasticolum’*), Gartn.-Tidende 1895: 122. 1895.

(See ***Fusarium oxysporum***)

*Holotypus*: Not located.

*Type locality*: **Germany**.

*Type substrate*: *Pinus sylvestris.*

*Note*: Synonym *fide*Wollenweber & Reinking (1935).  

*bomiense Fusarium* (Z.Q. Zeng & W.Y. Zhuang) O'Donnell *et al.*, Index Fungorum 440: 1. 2020.

***Neocosmospora bomiensis*** Z.Q. Zeng & W.Y. Zhuang, Phytotaxa 319: 177. 2017.

*Holotypus*: HMAS 254519.

*Ex-type culture*: HMAS 248885.

*Type locality*: **China**, Tibet Autonomous Region, Bomê County.

*Type substrate*: Twigs*.*

*Descriptions and illustrations*: See Zeng & Zhuang (2017b).

*Diagnostic DNA barcode*: *tef1*: KY829449.  

*bonordenii Fusarium* Sacc., Syll. Fung. 4: 699. 1886.

*Replaced synonym*: *Selenosporium aurantiacum* Bonord., Abh. Naturf. Ges. Halle 8: 97. 1864, *nom. illegit.*, Art. 53.1, *non Fusarium aurantiacum* Corda 1829.

(See *Fusarium dimerum*)

*Holotypus*: Not preserved *fide*Holubová-Jechová *et al.* (1994).

*Type locality*: **Germany**.

*Type substrate*: Branches of unknown tree*.*

*Note*: Synonym *fide*Wollenweber & Reinking (1935).  

***boothii Fusarium*** O'Donnell *et al.*, Fungal Genet. Biol. 41: 618. 2004.

*Holotypus*: BPI 843475.

*Ex-type culture*: CBS 316.73 = IMI 160243 = NRRL 26916.

*Type locality*: **South Africa**.

*Type substrate*: *Zea mays.*

*Descriptions and illustrations*: See O'Donnell *et al.* (2004).

*Diagnostic DNA barcodes*: *rpb1*: KM361641; *rpb2*: KM361659; *tef1*: GQ915503.  

*borneense Fusarium* (Petr.) O'Donnell *et al.*, Index Fungorum 440: 1. 2020.

***Neocosmospora borneensis*** (Petr.) Sand.-Den. & Crous, Persoonia 43: 115. 2019.

*Basionym*: *Nectria borneensis* Petr., Sydowia 8: 20. 1954.

*Holotypus*: K(M) 252860.

*Epitypus*: CBS H-23972, designated in Sandoval-Denis *et al.* (2019).

*Ex-epitype culture*: BBA 65095 = CBS 145462 = G.J.S. 85-197 = NRRL 22579.

*Epitype locality*: **Indonesia**, North Sulawesi, Bogani Nani Wartabone National Park.

*Epitype substrate*: Bark of a recently dead unidentified tree*.*

*Descriptions and illustrations*: See Sandoval-Denis *et al.* (2019).

*Diagnostic DNA barcodes*: *rpb1*: MW834213; *rpb2*: FJ240381; *tef1*: AF178344.  

*bostrycoides Fusarium* Wollenw. & Reinking, Phytopathology 15: 166. 1925.

***Neocosmospora bostrycoides*** (Wollenw. & Reinking) Sand.-Den. *et al.*, Persoonia 43: 115. 2019.

*Neotypus*: CBS H-23973, designated in Sandoval-Denis *et al.* (2019).

*Ex-neotype culture*: CBS 144.25.

*Neotype locality*: **Honduras**, Tela.

*Neotype substrate*: Soil*.*

*Descriptions and illustrations*: See Sandoval-Denis *et al.* (2019).

*Diagnostic DNA barcodes*: *rpb1*: MW218101; *rpb2*: LR583818; *tef1*: LR583597.  

***brachiariae Fusarium*** M.M. Costa *et al.*, Mycol. Progr. 20: 67. 2021.

*Holotypus*: UB 24188.

*Ex-type culture*: CML 3032.

*Type locality*: **Brazil**, Mato Grosso do Sul, Campo Grande.

*Type substrate*: Seed of *Brachiaria decumbens.*

*Descriptions and illustrations*: See Costa *et al.* (2021).

*Diagnostic DNA barcodes*: *rpb2*: MT901314; *tef1*: MT901348.  

***brachygibbosum Fusarium*** Padwick, Mycol. Pap. 12: 11. 1945.

*Holotypus*: IMI 268019.

*Ex-type culture*: BBA 64691 = NRRL 20954.

*Type locality*: **India**, Telangana, Hyderabad, Parbhani.

*Type substrate*: *Sorghum vulgare.*

*Descriptions and illustrations*: See Padwick (1945).

*Diagnostic DNA barcodes*: *rpb1*: MW233246; *rpb2*: MW233418; *tef1*: MW233075.  

***brasilicum Fusarium*** T. Aoki *et al.*, Fungal Genet. Biol. 41: 620. 2004.

*Holotypus*: BPI 843480.

*Ex-type culture*: CBS 119180 = NRRL 31281.

*Type locality*: **Brazil**.

*Type substrate*: *Avena sativa.*

*Descriptions and illustrations*: See O'Donnell *et al.* (2004).

*Diagnostic DNA barcodes*: *rpb1*: JABCJS010000032; *rpb2*: JABCJS010000357; *tef1*: AY452964.  

*brasiliense Fusarium* T. Aoki & O'Donnell, Mycoscience 46: 166. 2005.

(See *Fusarium azukiicola*)

*Holotypus*: BPI 843352.

*Ex-type culture*: MAFF 239050 = NRRL 31757.

*Type locality*: **Brazil**, Districto Federal, Brasilia.

*Type substrate*: *Glycines max.*

*Descriptions and illustrations*: See Aoki *et al.* (2005).

*Diagnostic DNA barcodes*: *rpb1*: MAEC01003448; *rpb2*: EU329565; *tef1*: MAEC01004196.  

*brassicae Fusarium* Lib. ex Cooke, Grevillea 8: 83. 1880.

(See *Fusarium candidum* Ehrenb.)

*Holotypus*: In B, Libert s.n. *fide* Index Fungorum.

*Type locality*: **France**.

*Type substrate*: Stem of *Brassica oleracea.*

*Note*: Synonym *fide*Wollenweber & Reinking (1935).  

*brassicae Fusarium* (Thüm.) Sacc., Syll. Fung. 4: 701. 1886, *nom. illegit.*, Art. 53.1.

*Basionym*: *Selenosporium brassicae* Thüm., Hedwigia 19: 191. 1880.

(See ***Fusarium avenaceum***)

*Holotypus*: Not located.

*Type locality*: **Belgium**.

*Type substrate*: Stem of *Brassica oleracea.*

*Note*: Synonym *fide*Wollenweber & Reinking (1935).  

*breve Fusarium* (Sand.-Den. & Crous) O'Donnell *et al.*, Index Fungorum 440: 1. 2020.

***Neocosmospora brevis*** Sand.-Den. & Crous, Persoonia 43: 119. 2019.

*Holotypus*: CBS H-23975.

*Ex-type culture*: CBS 144387 = MUCL 16108.

*Type locality*: **Belgium**, Heverlee.

*Type substrate*: Soil-water polluted with diethylene glycerol and ethylene glycerol*.*

*Descriptions and illustrations*: See Sandoval-Denis *et al.* (2019).

*Diagnostic DNA barcodes*: *rpb1*: MW834214; *rpb2*: LR583822; *tef1*: LR583601.  

***brevicatenulatum Fusarium*** Nirenberg *et al.*, Mycologia 90: 460. 1998.

*Holotypus*: Specimen in B *fide*Nirenberg *et al.* (1998).

*Ex-type culture*: BBA 69197 = CBS 404.97 = DAOM 225122= IMI 375329 = NRRL 25446.

*Type locality*: **Madagascar**.

*Type substrate*: *Striga asiatica.*

*Descriptions and illustrations*: See Nirenberg *et al.* (1998) and Leslie & Summerell (2006).

*Diagnostic DNA barcodes*: *rpb1*: MT010948; *rpb2*: MT010979; *tef1*: MT011005.  

***brevicaudatum Fusarium*** J.W. Xia *et al.*, Persoonia 43: 195. 2019.

*Holotypus*: CBS H-24051.

*Ex-type culture*: NRRL 43638 = UTHSC R-3500.

*Type locality*: **USA**, Florida.

*Type substrate*: *Trichechus* sp. (manatee).

*Descriptions and illustrations*: See Xia *et al.* (2019).

*Diagnostic DNA barcodes*: *rpb1*: KC808322; *rpb2*: GQ505843; *tef1*: GQ505665.  

*breviconum Fusarium* (Wollenw.) O'Donnell *et al.*, Index Fungorum 440: 1. 2020.

***Neocosmospora brevicona*** (Wollenw.) Sand.-Den. & Crous, Persoonia 43: 117. 2019.

*Basionym*: *Hypomyces haematococcus* var. *breviconus* Wollenw., Fusaria Autogr. Delin. 3: no. 828 (1930).

*Synonyms*: *Fusarium solani* var. *minus* Wollenw., *Fusarien*: 134. 1935.

*Nectria haematococca* var. *brevicona* (Wollenw.) Gerlach, Fusarium: Diseases, Biology, and Taxonomy (Philadelphia): 422. 1981.

*Lectotypus*: Fig. 828 in Wollenweber (1930), designated in Sandoval-Denis *et al.* (2019).

*Epitypus*: CBS H-23974 designated in Sandoval-Denis *et al.* (2019).

*Ex-epitype culture*: BBA 2123 = CBS 204.31 = NRRL 22659.

*Epitype locality*: **Indonesia**, West Java, Bogor.

*Epitype substrate*: *Gladiolus* sp.

*Descriptions and illustrations*: See Sandoval-Denis *et al.* (2019).

*Diagnostic DNA barcodes*: *rpb1*: MW218103; *rpb2*: LR583821; *tef1*: LR583600.  

*briosianum Fusarium* Ferraris, Fl. Ital. Crypt. Hyphales, Fasc. 13: 857. 1914.

(See ***Fusarium lateritium***)

*Holotypus*: Not located.

*Type locality*: **Italy**, Pavia.

*Type substrate*: Branches of *Styphnolobium japonicum* (syn. *Sophora japonica*).

*Note*: Synonym *fide*Wollenweber & Reinking (1935).  

***bubalinum Fusarium*** J.W. Xia *et al.*, Persoonia 43: 195. 2019

*Holotypus*: CBS H-24052.

*Ex-type culture*: CBS 161.25 = NRRL 26857 = NRRL 26918.

*Type locality*: **Australia**.

*Type substrate*: Unknown.

*Descriptions and illustrations*: See Xia *et al.* (2019).

*Diagnostic DNA barcodes*: *rpb2*: MN170381; *tef1*: MN170448.  

*bufonicola Fusarium* (Speg.) Sacc. & Trotter, Syll. Fung. 22: 1486. 1913.

(See ***Fusarium graminearum***)

*Basionym*: *Selenosporium bufonicola* Speg., Anales Mus. Nac. Buenos Aires, ser. 3, 13: 458. 1910.

*Holotypus*: In LPS (Myc. Argent. ser. 5, no. 1166) *fide*Farr (1973).

*Type locality*: **Argentina**, Buenos Aires.

*Type substrate*: Decaying body of *Amphibia* (toad).

*Note*: Synonym *fide*Wollenweber & Reinking (1935).  

*bugnicourtii Fusarium* Brayford, Trans. Brit. Mycol. Soc. 89: 350. 1987.

(See *Fusarium ambrosium*)

*Synonym*: *Fusarium tumidum* var. *coeruleum* Bugnic., Encycl. Mycol. 11: 83. 1939.

*Holotypus*: IMI 296597.

*Ex-type culture*: IMI 296597 = NRRL 20438 = MAFF 246291.

*Type locality*: **India**, Chinchona.

*Type substrate*: *Euwallacea fornicatus* on *Camellia sinensis*.

*Descriptions and illustrations*: See Brayford (1987).

*Diagnostic DNA barcodes*: *rpb1*: JX171470; *rpb2*: JX171584; *tef1*: AF178332.  

***buharicum Fusarium*** Jacz. ex Babajan & Teterevn.-Babajan, Mater. Mikol. Fitopatol. 8: 216. 1929.

*Holotypus*: LEP 127667.

*Epitypus* (*hic designatus*, MBT 10000662): **Uzbekistan**, Tashkent, on *Gossypium herbaceum*, 1928, A.I. Raillo, CBS 178.35 (preserved as metabolically inactive culture).

*Ex-epitype culture*: CBS 178.35 = DSM 62166 = NRRL 25488.

*Descriptions and illustrations*: See Gerlach & Nirenberg (1982).

*Diagnostic DNA barcodes*: *rpb1*: KX302920; *rpb2*: KX302928; *tef1*: KX302912.

*Notes*: Gerlach & Nirenberg (1982) designated CBS 178.35 as neotype of *F. buharicum* as they were unable to locate the type specimen. However, A. Jaczweski did place a specimen in LEP. Therefore, the neotype designation is superseded here (Art. 9.13) and CBS 178.35 is retained as epitype for this species.  

***bulbicola Fusarium*** Nirenberg & O'Donnell, Mycologia 90: 452. 1998.

*Replaced synonym*: *Fusarium sacchari* var. *elongatum* Nirenberg, Mitt. Biol. Bundesanst. Land- Forstw. Berlin-Dahlem 169: 59. 1976, *non Fusarium elongatum* Reinking 1934.

*Holotypus*: IMI 202877.

*Ex-type culture*: BBA 63628 = CBS 220.76 = DAOM 225114 = IMI 375322 = NRRL 13618.

*Type locality*: **Germany**.

*Type substrate*: *Haemanthus* sp*.*

*Descriptions and illustrations*: See Nirenberg (1976), Nirenberg & O'Donnell (1998) and Leslie & Summerell (2006).

*Diagnostic DNA barcodes*: *rpb1*: KF466394; *rpb2*: KF466404; *tef1*: AF160294.  

*bulbigenum Fusarium* Cooke & Massee, Grevillea 16: 49. 1887.

(See ***Fusarium oxysporum***)

*Holotypus*: ?K(M).

*Type locality*: **UK**.

*Type substrate*: *Narcissus* sp*.*

*Note*: Synonym *fide*Wollenweber & Reinking (1935).  

*bullatum Fusarium* Sherb., Mem. Cornell Univ. Agric. Exp. Sta. 6: 198. 1915.

(See ***Fusarium equiseti***)

*Typus*: CUP-007455.

*Type locality*: **USA**, Iowa.

*Type substrate*: Rotten tuber of *Solanum tuberosum.* Lectotypification pending study of material lodged in CUP.  

***burgessii Fusarium*** M.H. Laurence *et al.*, Fungal Diversity 49: 109. 2011.

*Holotypus*: CBS 125537 (preserved as metabolically inactive culture).

*Ex-type culture*: CBS 125537 = NRRL 66654 = RBG 5315.

*Type locality*: **Australia**, Queensland, Idalia National Park.

*Type substrate*: Soil*.*

*Descriptions and illustrations*: See Laurence *et al.* (2011).

*Diagnostic DNA barcodes*: *rpb1*: MT409440; *rpb2*: HQ646393; *tef1*: HQ667148.  

*butleri Fusarium* Wollenw., Phytopathology 3: 38. 1913, *nom. illegit.*, Art. 52.1.

(See ***Fusarium udum***)

*Authentic material*: ?B.

*Original locality*: **India**.

*Original substrate*: *Cajanus cajan.*

*Note*: Synonym *fide*Wollenweber & Reinking (1935).  

*butleri Fusarium* Kr.P. Singh & Edward, Allahabad Farmer 49: 94. 1979, *nom. illegit.*, Art. 53.1, *non Fusarium butleri* Wollenw. 1913.

*Synonym*: *Gibberella butleri* Kr.P. Singh & Edward, Allahabad Farmer 49: 92. 1979.

*Authentic material*: Not located.

*Original locality*: **India**.

*Original substrate*: *Cajanus cajan.*

*Notes*: Status unclear. No further records available for this taxon.  

*buxi Fusarium* Spreng., Syst. Veg., ed. 16, 4: 565. 1827.

***Pseudonectria buxi*** (DC.) Seifert *et al.*, Stud. Mycol. 68: 107. 2011.

*Basionym*: *Tubercularia buxi* DC., Fl. Franç., ed. 3, 5/6: 110. 1815.

*Synonyms*: *Fusisporium buxi* (DC.) Fr., Syst. Mycol. 3: 447. 1832, *nom. sanct*. [Fr., l.c.]

*Psilonia buxi* (DC.) Fr., Syst. Mycol. 3: 447. 1832, *nom. inval.*, Art. 36.1(c).

*Chaetostroma buxi* (DC.) Corda, Icon. Fung. 2: 31. 1838.

*Volutella buxi* (DC.) Berk. & Broome, Ann. Mag. Nat. Hist., ser. 2, 5: 465. 1850.

*Chaetodochium buxi* (DC.) Höhn., Mitt. Bot. Inst. T. H. Wien 9: 45. 1932.

*Nectria rousseliana* Mont., Ann. Sci. Nat., Bot., sér. 3, 16: 44. 1851.

*Stigmatea rousseliana* (Mont.) Fuckel, Jahrb. Nassauischen Vereins Naturk. 23–24: 97. 1870.

*Nectriella rousseliana* (Mont.) Sacc., Syll. Fung. 2: 452. 1883.

*Lasionectria rousseliana* (Mont.) Cooke (as ‘*rousselliana’*), Grevillea 12: 111. 1884.

*Pseudonectria rousseliana* (Mont.) Wollenw., Z. Parasitenk. (Berlin) 3: 489. 1931.

*Notarisiella rousseliana* (Mont.) Clem. & Shear, The genera of Fungi: 280. 1931.

*Nectria rousseliana* var. *viridis* Berk. & Broome, Ann. Mag. Nat. Hist., ser. 3, 3: 376. 1859.

*Volutella buxi f. rusci* Sacc., Michelia 2: 644. 1882.

*Holotypus*: ?PC.

*Type locality*: ?**Germany**/**France**.

*Type substrate*: Leaf of *Buxus* sp.  

*buxicola Fusarium* Sacc., Syll. Fung. 2: 518. 1883.

***Cyanonectria buxi*** (Fuckel) Schroers *et al.*, Stud. Mycol. 68: 120. 2011.

*Basionym*: *Gibbera buxi* Fuckel, Jahrb. Nassauischen Vereins Naturk. 27–28: 32. 1874.

*Synonyms*: *Lisea buxi* (Fuckel) Sacc., Syll. Fung. 2: 518. 1883.

*Gibberella buxi* (Fuckel) Cooke, Grevillea 12: 112. 1884.

*Fusarium subcorticale* Oudem., Ned. Kruidk. Arch., sér. 3, 3: 135. 1898.

*Fusarium dimorphum* J.V. Almeida & Sousa da Câmara, Revista Agron. (Lisbon) 1: 306. 1903.

*Fusarium buxicola* var. *chlamydosporum* Batikyan (as ‘*chlamydosporeae’*), Biol. Zhurn. Armenii 22: 90. 1969.

*Fusarium lateritium* var. *buxi* C. Booth, The Genus Fusarium: 113. 1971.

*Lectotypus*: G 00111019, selected in Schroers *et al.* (2011).

*Epitypus*: CBS H-20379, designated in Schroers *et al.* (2011).

*Ex-epitype culture*: CBS 125551.

*Epitype locality*: **Slovenia**, Arboretum Volčji Potok.

*Epitype substrate*: Decaying twig of *Buxus sempervirens* var. *elegantissima*.

*Descriptions and illustrations*: See Schroers *et al.* (2011).

*Diagnostic DNA barcodes*: *rpb2*: HM626689; *tef1*: HM626648.  

*byssinum Fusarium* McAlpine, Proc. Linn. Soc. New South Wales 22: 698. 1897.

*Holotypus*: VPRI 2556.

*Type locality*: **Australia**, New South Wales, Murwillumbah.

*Type substrate*: *Desmodium* sp*.*

*Notes*: Status unclear. This species was considered a member of *Diymopsis* by Saccardo (1899); *Hymenula* by Wollenweber & Reinking (1935); and *Aschersonia* by Walker (1962), who examined the type specimen and found that the fungus occurred in association with a scale insect on *Desmodium*. It is likely that this species belongs in *Microcera*, which are usually parasites of scale insects.  

***caapi Fusarium*** M.M. Costa *et al.*, Mycol. Progr. 20: 67. 2021.

*Holotypus*: UB 24189.

*Ex-type culture*: CML 3657.

*Type locality*: **Brazil**, São Paulo, Guaíra.

*Type substrate*: *Brachiaria brizantha.*

*Descriptions and illustrations*: See Costa *et al.* (2021).

*Diagnostic DNA barcodes*: *rpb2*: MT901316; *tef1*: MT901350.  

***caatingaense Fusarium*** A.C.S. Santos *et al.*, Mycologia 111: 248. 2019.

*Holotypus*: URM 91192.

*Ex-type culture*: MUM 1859 = URM 6779.

*Type locality*: **Brazil**, Pernambuco, Ibimirim.

*Type substrate*: *Dactylopius opuntiae.*

*Descriptions and illustrations*: See Santos *et al.* (2019).

*Diagnostic DNA barcodes*: *rpb2*: LS398495; *tef1*: LS398466.  

***cactacearum Fusarium*** Pasin. & Buzz.-Trav., Nuovo Giorn. Bot. Ital. 42: 120. 1935.

*Lectotypus* (*hic designatus*, MBT 10000663): **Italy**, Milan, *Thelocactus nidulans*, 1935, L. Pasinetti & A. Buzzati-Traverso, in Nuovo Giorn. Bot. Ital. 42: Pl. I, fig. 1.

*Descriptions and illustrations*: See Pasinetti & Buzzati-Traverso (1935).

*Notes*: Based on illustrations by Pasinetti & Buzzati-Traverso (1935), this species could be a synonym of *Neocosmospora solani* but requires further investigation. No holotype specimen could be located and therefore an illustration is designated as lectotype.  

***cacti-maxonii Fusarium*** Pasin. & Buzz.-Trav., Nuovo Giorn. Bot. Ital. 42: 120. 1935.

*Lectotypus* (*hic designatus*, MBT 10000664): **Italy**, Milan, *Cactus maxonii*, 1935, L. Pasinetti & A. Buzzati-Traverso, in Nuovo Giorn. Bot. Ital. 42: Pl. I, fig. 4.

*Descriptions and illustrations*: See Pasinetti & Buzzati-Traverso (1935).

*Notes*: Based on illustrations by Pasinetti & Buzzati-Traverso (1935), this species could be a synonym of *Fusarium oxysporum* but requires further investigation. No holotype specimen could be located and therefore an illustration is designated as lectotype.  

*caeruleum Fusarium* Lib. ex Sacc. (as ‘*cæruleum’*), Syll. Fung. 4: 705. 1886.

*Synonyms*: *Fusarium solani* var. *caeruleum* (Lib. ex Sacc.) Bilaĭ, Fusarii (Biologija i sistematika): 287. 1955, *nom. inval*., Art. 41.5.

*Fusarium solani* var. *caeruleum* (Lib. ex Sacc.) C. Booth, The Genus Fusarium: 51. 1971.

?*Fusarium violaceum* Fuckel, Fungi Rhen. Exs., Fasc. 3: no. 209. 1863.

*Fusarium aeruginosum* Delacr., Bull. Soc. Mycol. France 7: 110. 1891.

*Selenosporium caeruleum* Lib., 1834. (in herb.; *nom. inval*., Art. 38.1a).

*Fusarium caeruleum* var. *cellulosae* Sartory *et al.*, Papier 38: 43. 1935.

?*Hypomyces asclepiadis* Zerova, Zhurn. Inst. Bot. Vseukraïns'k. Akad. Nauk 11: 103. 1937.

*Holotypus*: BR5020140171069.

*Type locality*: **Belgium**.

*Type substrate*: *Solanum tuberosum.*

*Notes*: Status doubtful. See Sandoval-Denis *et al.* (2019).  

*calcareum Fusarium* (Thüm.) Sacc., Syll. Fung. 4: 712. 1886.

(See ***Fusarium oxysporum***)

*Basionym*: *Fusisporium calcareum* Thüm., Inst. Coimbra 28: 262. 1881.

*Holotypus*: S-F45605.

*Type locality*: **Portugal**, Coimbra.

*Type substrate*: *Lagenaria vulgaris.*

*Note*: Synonym *fide*Wollenweber & Reinking (1935).  

?*calidariorum Fusarium* Sacc., Ann. Mycol. 4: 274. 1906.

***Colletotrichum anthurii*** Delacr., Bull. Soc. Mycol. France 13: 110. 1897.

*Synonyms*: *Fusoma calidariorum* Sacc., Ann. Mycol. 4: 274. 1906.

*Fusoma calidariorum* var. *acanthi* Lindegg, Riv. Patol. Veg. 25: 233. 1935.

*Holotypus*: In PAD.

*Type locality*: **Italy**, Padua, botanical garden.

*Type substrate*: *Anthurium scherzerianum.*

*Notes*: Synonym *fide*Wollenweber & Reinking (1935). No record could be located for the transfer of this epithet to the genus *Fusarium*. In Saccardo (1906) on p. 274, no new combination is provided and only the new name *Fusoma calidariorum* was introduced. Similarly, Lindegg (1935) introduced a new variety as *Fusoma calidariorum* var. *acanthi*, not in the genus *Fusarium*. Although Wollenweber & Reinking (1935) did treat this as *Fusoma*, Booth (1971) incorrectly treated the variety *acanthi* in the genus *Fusarium*.  

***callistephi Fusarium*** L. Lombard & Crous, Persoonia 43: 15. 2018 [2019].

*Holotypus*: CBS H-23608.

*Ex-type culture*: CBS 187.53 = NRRL 36330.

*Type locality*: **Netherlands**, Oostenbrink.

*Type substrate*: *Callistephus chinensis.*

*Descriptions and illustrations*: See Lombard *et al.* (2019b).

*Diagnostic DNA barcodes*: *rpb2*: MH484875; *tef1*: MH484966.  

*callosporum Fusarium* Pat., Bull. Soc. Mycol. France 9: 164. 1893.

(See *Fusarium coccophilum*)

*Holotypus*: Not located.

*Type locality*: **Ecuador**, Quito.

*Type substrate*: Parasitic on *Septobasidium pedicellatum.*

*Note*: Synonym *fide*Wollenweber & Reinking (1935).  

*camerunense Fusarium* Henn., Bot. Jahrb. Syst. 22: 81. 1895.

***Gloeosporium camerunense*** (Henn.) Wollenw., Fusaria Autogr. Delin. 1: 499. 1916.

*Holotypus*: In B *fide*Hein (1988).

*Type locality*: **Cameroon**, Itoki.

*Type substrate*: Bark of unknown tree*.*

*Note*: Synonym *fide*Wollenweber & Reinking (1935).  

***camptoceras Fusarium*** Wollenw. & Reinking, Phytopathology 15: 158. 1925.

*Neotypus*: CBS H-24077, designated in Xia *et al.* (2019).

*Ex-neotype culture*: ATCC 16065 = ATCC 24364 = BBA 9810 = CBS 193.65 = DSM 62167 = IMI 112500 = NRRL 20716 = NRRL 36344.

*Neotype locality*: **Costa Rica**.

*Neotype substrate*: Cushion gall of *Theobroma cacao*.

*Descriptions and illustrations*: See Wollenweber & Reinking (1935), Booth (1971), Gerlach & Nirenberg (1982), Marasas *et al.* (1998) and Leslie & Summerell (2006).

*Diagnostic DNA barcodes*: *rpb1*: MW928800; *rpb2*: MN170383; *tef1*: MN170450.  

*campylopodii Fusarium* Weir, Mycologia 60: 374. 1968, *nom. inval*., Art. 38.1(a).

*Authentic material*: Not located.

*Original locality*: **USA**, Washington.

*Original substrate*: *Arceuthobium* sp.

*Note*: The name is mentioned but neither a diagnosis nor a description was provided.  

*candidulum Fusarium* Sacc., Ann. Mycol. 6: 567. 1908.

(See ***Fusarium oxysporum***)

*Holotypus*: Not located.

*Type locality*: **Mexico**.

*Type substrate*: *Myrtillocactus geometrizans*.

*Note*: Synonym *fide*Wollenweber & Reinking (1935).  

*candidum Fusarium* Ehrenb., Sylv. Mycol. Berol.: 24. 1818.

***Neonectria candida*** (Ehrenb.) Rossman *et al.*, Stud. Mycol. 80: 217. 2015.

*Synonyms*: *Ramularia candida* (Ehrenb.) Wollenw., Phytopathology 1: 220. 1913.

*Cylindrocarpon ehrenbergii* Wollenw., Fusaria Autogr. Delin. 1: 461. 1916.

*Sclerotium castaneum* Lib., in herb. 1832, *nom. nud.*

*Fusarium castaneum* Lindau (as “(Lib.) Lindau”), Rabenh. Krypt.-Fl. 1(9): 556. 1909.

?*Fusidium candidum* Willk., Die mikroskopischen Feinde des Waldes 1: 103. 1866, *nom. illegit.*, Art. 53.1.

?*Fusarium candidum* Sacc. & D. Sacc., Syll. Fung. 18: 674. 1906, *nom. illegit.*, Art. 53.1.

?*Fusarium candidum* Dasz., Bull. Soc. Bot. Genève, 2 sér. 4: 293. 1913, *nom. illegit.*, Art. 53.1.

*Fusarium brassicae* Lib. ex Cooke, Grevillea 8: 83. 1880.

*Selenosporium brassicae* Thüm., Hedwigia 19: 191. 1880.

*Fusarium brassicae* (Thüm.) Sacc., Syll. Fung. 4: 701. 1886, *nom. illegit.*, Art. 53.1.

*Fusarium obtusiusculum* Sacc., Michelia 2: 297. 1881.

*Fusarium rhizogenum* Pound & Clem., Bot. Surv. Nebraska 3: 12. 1894.

*Fusarium oxysporum* var. *obtusiusculum* (Sacc.) Cif., Ann. Bot. (Rome) 16: 221. 1924.

*Cylindrocarpon obtusiusculum* (Sacc.) U. Braun, Cryptog. Bot. 4: 113. 1993.

*Fusarium eichleri* Bres., Ann. Mycol. 1: 130. 1903.

*Neonectria ramulariae* Wollenw., Ann. Mycol. 15: 52. 1917.

*Nectria ramulariae* (Wollenw.) E. Müll., Beitr. Kryptogamenfl. Schweiz 11: 634. 1962.

*Cylindrocarpon magnusianum* Wollenw., Z. Parasitenk. (Berlin) 1: 172. 1928.

*Holotypus*: Not located.

*Type locality*: **Germany**, Berlin.

*Type substrate*: Unknown.  

*candidum Fusarium* (Link) Sacc., Syll. Fung. 4: 720. 1886, *nom. illegit.*, Art. 53.1.

***Neonectria ditissima*** (Tul. & C. Tul.) Samuels & Rossman, CBS Biodiversity Ser. 4: 134. 2006.

*Basionym*: *Nectria ditissima* Tul. & C. Tul., Select. Fung. Carpol. 3: 73. 1865.

*Synonyms*: *Cucurbitaria ditissima* (Tul. & C. Tul.) Kuntze, Revis. Gen. Pl. 3: 461. 1898.

*Fusidium candidum* Link, Mag. Neuesten Entdeck. Gesammten Naturk. Ges. Naturf. Freunde Berlin 3: 8. 1809, *nom. sanct*. [Fr., Syst. Mycol. 3: 481. 1832].

*Cylindrocarpon candidum* (Link) Wollenw., Fusaria Autogr. Delin. 1: 476. 1916.

?*Fusisporium cylindricum* Mont., Ann. Sci. Nat., Bot., sér. 2, 17: 120. 1842.

?*Fusarium cylindricum* (Mont.) Sacc., Syll. Fung. 4: 720. 1886.

*Fusarium fissum* Peyl, Lotos 8: 30. 1858.

?*Fusarium heteronemum* Berk. & Broome (as ‘*heteronema'*’), Ann. Mag. Nat. Hist., Ser. 3, 15: 402. 1865.

?*Cylindrocarpon heteronema* (Berk. & Broome) Wollenw. (as ‘*heteronemum’*), Fusaria Autogr. Delin. 1: 460. 1916.

?*Ramularia heteronema* (Berk. & Broome) Wollenw. (as ‘*heteronemum'*’), Fusaria Autogr. Delin. 1: 460. 1916.

*Fusarium ulmi* P. Crouan & H. Crouan, Fl. Finistère: 14. 1867.

*Fusarium fragrans* P. Crouan & H. Crouan, Fl. Finistère: 14. 1867.

*Fusarium decipiens* Cooke & Massee, in Cooke, Handb. Austral. Fungi: 388. 1892, *nom. inval*., Art. 39.1.

*Fusarium mali* Allesch., Ber. Bot. Vereines Landshut 12: 130. 1892.

*Fusarium sarcochroum f. mali* (Allesch.) Ferraris, 1910.

*Cylindrocarpon mali* (Allesch.) Wollenw., Phytopathology 18: 225. 1928.

*Sporotrichum amenti* P. Karst., Hedwigia 31: 296. 1892.

*Fusarium fractum* Sacc. & Cavara, Nuovo Giorn. Bot. Ital., n.s. 7: 308. 1900.

*Cylindrocarpon fractum* (Sacc. & Cavara) Wollenw., Fusaria Autogr. Delin. 2: 655. 1924.

*Nectria galligena* Bres., in Strasser, Verh. K. K. Zool.-Bot. Ges. Wien 51: 413. 1901.

*Dialonectria galligena* (Bres.) Petch ex E.W. Mason & Grainger, Cat. Yorkshire Fung.: 32. 1937.

*Neonectria galligena* (Bres.) Rossman & Samuels, Stud. Mycol. 42: 159. 1999.

*Fusarium prunorum* McAlpine, Fungus Diseases of stone-fruit trees in Australia: 91. 1902.

*Fusarium willkommii* Lindau, Rabenh. Krypt.-Fl. ed. 2, 1(9): 551. 1909.

*Cylindrocarpon willkommii* (Lindau) Wollenw., Z. Parasitenk. (Berlin) 1: 150. 1928.

*Fusarium luteum* Parav., Ann. Mycol. 16: 302. 1918, *nom. illegit.*, Art. 53.1.

*Nectria ditissima* var. *arctica* Wollenw., Angew. Bot. 8: 189. 1926.

*Cylindrocarpon candidum* var. *medium* Wollenw., Z. Parasitenk. (Berlin) 1: 158. 1928.

*Cylindrocarpon candidum* var. *majus* Wollenw., Z. Parasitenk. (Berlin) 1: 158. 1928.

*Cylindrocarpon candidum* var. *minus* Wollenw., Z. Parasitenk. (Berlin) 1: 155. 1928.

*Cylindrocarpon mali* var. *flavum* Wollenw., Z. Parasitenk. (Berlin) 1: 150. 1928.

*Cylindrocarpon willkommii* var. *pluriseptatum* Wollenw., Z. Parasitenk. (Berlin) 1: 152. 1928.

*Cylindrocarpon willkommii* var. *minus* Wollenw., Z. Parasitenk. (Berlin) 1: 152. 1928.

*Holotypus*: Not located.

*Type locality*: **Unknown**.

*Type substrate*: Branch.

*Notes*: Synonyms *fide*Wollenweber & Reinking (1935). Several names that include *Fusidium candidum* (1809), *Fusisporium cylindricum* (1842) and *Fusarium fissum* (1858) should take preference for this taxon. However, the epithet “*candidum*” is already occupied in the genus *Neonectria* and cannot be used. Furthermore, the link between *Fusisporium cylindricum* and *Fusarium fissum* with *Neonectria ditissima* still needs to be established. Therefore, we choose to retain the name *Neonectria ditissima* for this taxon.  

*candidum Fusarium* Sacc. & D. Sacc., Syll. Fung. 18: 674. 1906, *nom. illegit.*, Art. 53.1, *non Fusarium candidum* Ehrenb. 1818.

*Basionym*: *Fusidium candidum* Willk., Die mikroskopischen Feinde des Waldes 1: 103. 1866.

*Replacing synonym*: *Fusarium willkommii* Lindau, Rabenh. Krypt.-Fl. ed. 2, 1(9): 551. 1910.

(See *Fusarium willkommii*)  

*capitatum Fusarium* Schwein., Trans. Amer. Philos. Soc., n.s., 4: 302. 1832.

*Synonym*: *Pionnotes capitata* (Schwein.) Fr., Summa Veg. Scand. 2: 481. 1849.

*Holotypus*: PH00081394.

*Type locality*: **USA**, Pennsylvania.

*Type substrate*: *Tsuga canadensis*.

*Notes*: The type material of *Fusarium capitatum*, type species of the genus *Pionnotes*, was re-examined by Seifert (2013). It represents not a hyphomycete but a basidiomycete identical to *Dacrymyces chrysospermus*. Therefore, the generic name *Pionnotes* is a synonym of *Dacrymyces* rather than *Fusarium*. Further evaluations are necessary in future phylogenetic revisions of the *Dacrymycetales*.  

*caricis Fusarium* Oudem., Verslagen Meded. Afd. Natuurk. Kon. Akad. Wetensch., ser. 3, 7: 325. 1890.

(See ***Fusarium graminearum***)

*Holotypus*: ?L.

*Type locality*: **Netherlands**, Zuid-Holland Province, Den Haag.

*Type substrate*: Leaves of *Carex* sp.

*Note*: Synonym *fide*Wollenweber & Reinking (1935).  

*caries Fusarium* Nees, Nova Acta Phys.-Med. Acad. Caes. Leop.-Carol. Nat. Cur. 19, Suppl. 1: 478. 1843.

*Holotypus*: ?B, L or STR.

*Type locality*: **China**.

*Type substrate*: *Meoschium lodiculare*.

*Notes*: Status unclear. Not *Fusarium fide*Wollenweber & Reinking (1935).  

***carminascens Fusarium*** L. Lombard *et al.*, Persoonia 43: 19. 2018 [2019].

*Holotypus*: CBS H-23609.

*Ex-type culture*: CBS 144738 = CPC 25800.

*Type locality*: **South Africa**, KwaZulu-Natal Province.

*Type substrate*: *Zea mays.*

*Descriptions and illustrations*: See Lombard *et al.* (2019b).

*Diagnostic DNA barcodes*: *rpb1*: MW928801; *rpb2*: MH484937; *tef1*: MH485028.  

*carneolum Fusarium* P. Karst., Meddeland. Soc. Fauna Fl. Fenn. 16: 35. 1888.

***Vermicularia herbarum*** Westend., Herb. Crypt. Belg. no. 393. 1849.

*Holotypus*: ?H.

*Type locality*: **Finland**, Tammela.

*Type substrate*: *Iris pseudacorus*.

*Note*: Synonym *fide*Wollenweber & Reinking (1935).  

*carneoroseum Fusarium* Cooke, Grevillea 19: 4. 1890.

(See ***Fusarium lateritium***)

*Holotypus*: In K(M), Colenso 538 *fide* Index Fungorum.

*Type locality*: **New Zealand**.

*Type substrate*: Bark.

*Note*: Synonym *fide*Wollenweber & Reinking (1935).  

*carneum Fusarium* (Mont.) Sacc., Syll. Fung. 4: 724. 1886.

*Basionym*: *Fusisporium carneum* Mont., Ann. Sci. Nat., Bot., sér. 2, 17: 120. 1842.

*Holotypus*: ?PC.

*Type locality*: **Cuba**.

*Type substrate*: Leaf of monocotyledon.

*Notes*: Status unclear. Not *Fusarium fide*Wollenweber & Reinking (1935).  

*carniforme Fusarium* Ellis & Tracy, *nom. inval*., Art. 38.1(a).

*Synonym*: *Ramularia carniformis* Sherb., Phytopathology 18: 149. 1928.

*Authentic material*: NY0093683.

*Original locality*: **USA**, Mississippi, Starkville.

*Original substrate*: *Tripsacum dactyloides*.

*Notes*: Status unclear. Braun (1998) considered this species doubtful as conidia appeared microdochium-like.  

***carpineum Fusarium*** Davis, Trans. Wisconsin Acad. Sci. 18: 106. 1915.

*Holotypus*: BPI 442722.

*Type locality*: **USA**, Wisconsin, Wyalusing.

*Type substrate*: *Carpinus caroliniana*.

*Notes*: This species was not treated by any of Wollenweber & Reinking (1935), Booth (1971), or Gerlach & Nirenberg (1982). A literature search could not find any additional information pertaining to this species.  

*carpini Fusarium* Schulzer & Sacc., Hedwigia 23: 128. 1884.

(See ***Fusarium expansum***)

*Holotypus*: Not located.

*Type locality*: **Croatia**, Vinkovci.

*Type substrate*: *Carpinus betulus*.

*Note*: Synonym *fide*Wollenweber & Reinking (1935).  

***cassiae Fusarium*** R.H. Perera *et al.*, Mycosphere 11: 2138. 2020.

*Holotypus*: MFLU 18-2751.

*Ex-type culture*: MFLUCC 18-0573.

*Type locality*: **Thailand**, Phayao Province.

*Type substrate*: Pods of *Cassia fistula.*

*Descriptions and illustrations*: See Perera *et al.* (2020).

*Diagnostic DNA barcodes*: *rpb2*: MT212197; *tef1*: MT212205.  

*castagnei Fusarium* Mont., Ann. Sci. Nat., Bot., sér. 3, 12: 296. 1849.

***Myxosporium castagnei*** (Mont.) Wollenw., Fusaria. Autogr. Delin. 1: 489. 1916.

*Holotypus*: ?PC.

*Type locality*: **France**, Marseille.

*Type substrate*: *Psoralea bituminosa*.

*Note*: Synonym *fide*Wollenweber & Reinking (1935).  

*castaneicola Fusarium* W. Yamam., Trans. Mycol. Soc. Japan 3: 114. 1962, *nom. inval*., Art. 39.1 & 40.1.

***Rugonectria castaneicola*** (W. Yamam. & Oyasu) Hirooka & P. Chaverri, Stud. Mycol. 68: 73. 2011.

*Basionym*: *Nectria castaneicola* W. Yamam. & Oyasu, Sci. Rep. Hyogo Univ. Agric. 3: 15. 1957.

*Synonyms*: *Neonectria castaneicola* (W. Yamam. & Oyasu) Tak. Kobay. & Hirooka, J. Gen. Pl. Pathol. 71: 126. 2005, *nom. inval*., Art. 41.5.

*Cylindrocarpon castaneicola* Tak. Kobay. & Hirooka, J. Gen. Pl. Pathol. 71: 126. 2005.

*Authentic material*: Not designated.

*Original locality*: **Japan**, Hyogo.

*Original substrate*: *Castanea crenata*.

*Note*: This *Fusarium* epithet is invalid as neither a Latin diagnosis (Art. 39.1) nor a type designation (Art. 40.1) was included in the original description.  

*castaneum Fusarium* Lindau (as “(Lib.) Sacc.“), Rabenh. Krypt.-Fl. ed. 2, 1(9): 556. 1909.

*Synonym*: *Sclerotium castaneum* Lib., in herb. 1832, *nom. nud.*

(See *Fusarium candidum* Ehrenb.)

*Authentic material*: Not located.

*Original locality*: **Belgium**, Ardennes.

*Original substrate*: *Brassica oleracea*.

*cataleptum Fusarium* Cooke & Harkn., Grevillea 12: 96. 1884.

(See *Fusarium coccophilum*)

*Holotypus*: In K(M), Harkness 1981 *fide* Index Fungorum.

*Type locality*: **USA**, California, San Rafael.

*Type substrate*: *Acacia* sp.

*Note*: Synonym *fide*Wollenweber & Reinking (1935).  

*catenatum Fusarium* (Sand.-Den. & Crous) O'Donnell *et al.*, Index Fungorum. 440: 1. 2020.

***Neocosmospora catenata*** Sand.-Den. & Crous, Persoonia 41: 115. 2018.

*Holotypus*: CBS H-23225.

*Ex-type culture*: CBS 143229 = NRRL 54993 = UTHSC 09-1009.

*Type locality*: **USA**, Georgia.

*Type substrate*: *Stegostoma fasciatum*.

*Descriptions and illustrations*: See Sandoval-Denis & Crous (2018).

*Diagnostic DNA barcodes*: *rpb1*: KC808292; *rpb2*: KC808355; *tef1*: KC808214.  

***cateniforme Fusarium*** J.W. Xia *et al.*, Persoonia 43: 197. 2019

*Holotypus*: CBS H-24053.

*Ex-type culture*: ATCC 11853 = CBS 150.25.

*Type locality*: **Unknown**.

*Type substrate*: Unknown.

*Descriptions and illustrations*: See Xia *et al.* (2019).

*Diagnostic DNA barcodes*: *rpb2*: MN170384; *tef1*: MN170451.  

***catenulatum Fusarium*** F.J. Chen, Mycosystema 19: 459. 2000.

*Holotypus*: HMAS 71749.

*Ex-type culture*: AS 3.4704.

*Type locality*: **China**, Shaanxi, Yangling.

*Type substrate*: *Gossypium hirsutum.*

*Descriptions and illustrations*: See Chen (2000).  

*caucasicum Fusarium* Letov, Mater. Mikol. Fitopatol. 8: 225. 1929.

*Holotypus*: Not located.

*Ex-type culture*: CBS 179.35 = IFO 5979 = NRRL 13954.

*Type locality*: **Republic of Azerbaijan**.

*Type substrate*: *Gossypium hirsutum.*

*Descriptions and illustrations*: See Gerlach & Nirenberg (1982).

*Notes*: Status doubtful/unclear. The ex-type culture (CBS 179.35) accessioned in CBS appears to be either contaminated or transpositioned by another *Fusarium* sp. (Sandoval-Denis *et al.* 2019). A sequence of the *tef1* gene region (DQ247543) from the copy accessioned at NRRL (NRRL 13954) places this species within the *Neocosmospora falciformis* clade (Sandoval-Denis *et al.* 2019). The status of the copy accessioned at IFO is not known.  

*caudatum Fusarium* Wollenw., J. Agric. Res. 2: 262. 1914.

(See ***Fusarium scirpi***)

*Lectotypus* (*hic designatus*, MBT 10000665): **USA**, South Carolina, Clemson College, *Ipomoea batatas*, date unknown, Harter & Field, in Wollenweber, J. Agric. Res. 2: 262, pl. 16, fig. M.

*Notes*: Synonym *fide*Wollenweber & Reinking (1935). No holotype specimen could be located and therefore an illustration is designated as lectotype.  

*cavispermum Fusarium* Corda, Icon. Fung. 1: 3. 1837.

***Cosmosporella cavisperma*** (Corda) Sand.-Den. *et al.*, Stud. Mycol. 98 (no. 100116): 44. 2021.

*Synonyms*: *Fusarium aquaeductuum* var. *cavispermum* (Corda) Raillo, Fungi of the Genus Fusarium: 280. 1950.

*Fusarium oxydendri* Ellis & Everh., Bull. Torrey Bot. Club 24: 477. 1897.

*Fusarium cavispermum* var. *minus* Wollenw., Fusaria Autogr. Delin. 3: 848. 1930.

*Lectotypus*: AKJ. Corda, Icon. Fung. 1: pl. I, fig. 58, designated in this study.

*Type locality*: **Czech Republic**.

*Type substrate*: Resin of *Pinus* sp.

*Epitypus*: CBS 172.31 (metabolic inactive specimen) designated in this study.

*Ex-epitype*: CBS 172.31 = NRRL 13996.

*Epitype locality*: **Norway**.

*Epitype substrate*: *Pinus sylvestris*.

*Diagnostic DNA barcodes*: *rpb1*: JX171465; *rpb2*: JX171579.  

*celosiae Fusarium* T. Abe, Mem. Coll. Agric. Kyoto Imp. Univ. 7: 51. 1928.

(See ***Fusarium fujikuroi***)

*Holotypus*: Not located.

*Type locality*: **Japan**.

*Type substrate*: Living stems and leaves of *Celosia cristata*.

*Note*: Synonym *fide*Wollenweber & Reinking (1935).  

***celtidicola Fusarium*** Q.J. Shang *et al.*, Phytotaxa 361: 255. 2018.

*Holotypus*: MFLU 15-3646.

*Ex-type culture*: KUMCC 16-0019 = MFLUCC 16-0526.

*Type locality*: **Italy**, Forlì-Cesena Province, Forlì, Viale dell’Appennino.

*Type substrate*: *Celtis australis.*

*Descriptions and illustrations*: See Shang *et al.* (2018).

*Diagnostic DNA barcodes*: *rpb1*: MH576579; *rpb2*: MH576577; *tef1*: MH576581.  

*celtidis Fusarium* Ellis & Tracy, J. Mycol. 6: 76. 1890.

(See ***Fusarium lateritium***)

*Syntypes*: In BPI, ISC & MICH.

*Type locality*: **USA**, Mississippi, Starkville.

*Type substrate*: *Celtis occidentalis.*

*Note*: Synonym *fide*Wollenweber & Reinking (1935).  

*celtidis Fusarium* Pass., Atti Reale Accad. Lincei, Rendiconti Cl. Sci. Fis., 4 sér. 7: 51. 1891, *nom. illegit.*, Art. 53.1.

*Replacing synonym*: *Fusarium sphaeriiforme* Sacc. (as ‘*sphaeriaeforme*’), Syll. Fung. 10: 723. 1892.

(See *Fusarium melanochlorum*)

*Holotypus*: ?PARMA.

*Type locality*: **Italy**, Parma, Vigheffio.

*Type substrate*: Dead branch of *Celtis australis*.

*Note*: Synonym *fide*Wollenweber & Reinking (1935).  

*cepae Fusarium* Hanzawa, Mycol. Centralbl. 5(1): 5. 1914.

(See ***Fusarium oxysporum***)

*Lectotypus* (*hic designatus*, MBT 10000666): **Japan**, Sapporo, *Allium cepa*, 1914, J. Hanzawa, 5(1): 6, fig. 1.

*Notes*: Synonym *fide*Wollenweber & Reinking (1935). No holotype specimen could be located and therefore an illustration is designated as lectotype.  

*cerasi Fusarium* Rolland & Ferry, in Roumeguère, Rev. Mycol. (Toulouse) 14: 170. 1892.

?***Foveostroma drupacearum*** (Lév.) DiCosmo, Canad. J. Bot. 56: 1682. 1978.

*Basionym*: *Micropera drupacearum* Lév., Ann. Sci. Nat., Bot., sér. 3, 5: 283. 1846.

*Synonyms*: ?*Peziza cerasi* Pers., Neues Mag. Bot. 1: 115. 1794.

?*Dermea cerasi* (Pers.) Fr., Syst. Orb. Veg. 1: 115. 1825.

*Syntype*: ILL00220294 (Fungi Sel. Gall. Exs. No. 6119).

*Type locality*: **France**, Saint-Dié-des-Vosges.

*Type substrate*: *Prunus* sp. (cherry tree).

*Note*: This species was excluded from *Fusarium* by Wollenweber (1943). Gerlach & Nirenberg (1982) considered this species as a possible synonym of *Micropera drupacearum* on which the present synonymies are based.  

*cerealis Fusarium* (P. Karst.) Gruyter & J.H.M. Schneid., Jaarb. Plantenziektenk. Dienst 1989/1990, no. 168: 135. 1991, *nom. inval*., Art. 41.4.

***Gliomastix cerealis*** (P. Karst.) C.H. Dickinson, Mycol. Pap. 115: 19. 1968.

*Basionym*: *Coniosporium cerealis* P. Karst., Meddeland. Soc. Fauna Fl. Fenn. 14: 109. 1887.

*Synonyms*: *Acremonium cerealis* (P. Karst.) W. Gams, *Cephalosporium-artige Schimmelpilze* (Stuttgart): 88. 1971.

*Gliomastix guttuliformis* J.C. Br. & W.B. Kendr., Trans. Brit. Mycol. Soc. 41: 499. 1958.

*Holotypus*: In herb. P.A. Karsten in H *fide*Dickinson (1968).

*Type locality*: **Finland**, Mustiala.

*Type substrate*: *Secale cereale*.  

***cerealis Fusarium*** (Cooke) Sacc., Syll. Fung. 4: 713. 1886.

*Basionym*: *Fusisporium cerealis* Cooke, Grevillea 6: 139. 1878.

*Synonym*: *Fusarium culmorum* var. *cerealis* (Cooke) Wollenw., Fusaria Autogr. Delin. 3: 946. 1930.

*Fusarium roseum f*. *cerealis* (Cooke) W.C. Snyder & H.N. Hansen, Amer. J. Bot. 32: 663. 1945.

*Gibberella rosea f*. *cerealis* (Cooke) W.C. Snyder & H.N. Hansen, Amer. J. Bot. 32: 664. 1945.

*Fusarium sambucinum* var. *cerealis* (Cooke) Raillo, Fungi of the Genus Fusarium: 211. 1950.

*Fusarium crookwellense* L.W. Burgess *et al.*, Trans. Brit. Mycol. Soc. 79: 498. 1982.

*Holotypus*: ?K(M) 133541.

*Type locality*: **USA**, California, Gainesville.

*Type substrate*: *Zea mays.*

*Notes*: Wollenweber & Reinking (1935) considered *F. cerealis* as a variety of *F. culmorum*, whereas Raillo (1950) considered it as a variety of *F. sambucinum*. Gerlach & Nirenberg (1982) applied a broader concept to *F. culmorum* that did not separate this variety in *F. culmorum*. Nirenberg (1990) recognised *F. cerealis* as a species and considered *F. crookwellense* as a synonym of *F. cerealis*. However, Leslie & Summerell (2006) recommend the use of the name *F. crookwellense* over *F. cerealis*, indicating that no type material is available for *F. cerealis*. We choose to follow Nirenberg (1990) to consider *F. crookwellense* a synonym under *F. cerealis*. The material lodged in K(M) requires further investigation to determine whether epi- or neotypification is required.  

*cesatii Fusarium* Rabenh., Klotzschii Herb. Viv. Mycol. Cent. 15: no. 1440. 1850.

***Hymenula rubella*** Fr., Elench. Fung. 2: 38. 1828.

*Lectotypus* (of *Fusarium cesatii*, *hic designatus*, MBT 10000667): **Italy**, Vercelli, *Carex* sp., 1849, collector unknown, Rabenh., Klotzschii Herb. Viv. Mycol. Ed. I no. 1440 in HAL.

*Note*: Synonym *fide*Wollenweber & Reinking (1935).  

*cesatii Fusarium* Thüm., Pilze Weinst.: 49. 1878, *nom. illegit.*, Art. 53.1, *non Fusarium cesatii* Rabenh. 1850.

***Elsinoe ampelina*** (de Bary) Shear, Phytopathology 19: 677. 1929.

*Basionym*: *Sphaceloma ampelina* de Bary, Ann. Oenol. 4: 165. 1874.

*Synonyms*: *Manginia ampelina* (de Bary) Viala & Pacottet, C. r. hebd. Séanc. Acad. Sci., Paris 139: 88. 1904.

*Pionnotes cesatii* Sacc., Syll. Fung. 4: 726. 1886.

*Ramularia ampelophaga* Pass., Bol. Comit. Agric. Parmense 9: 125. 1876.

*Gloeosporium ampelophagum* (Pass.) Sacc., Michelia 1: 217. 1878.

*Authentic material*: S-F47363.

*Original locality*: **Italy**, Vercelli.

*Original substrate*: Decaying stump of *Vitis vinifera*.

*Note*: Synonym *fide*Wollenweber & Reinking (1935).  

*chaetomium Fusarium* Wallr., Fl. Crypt. Germ. 2: 242. 1833.

***Colletotrichum chaetomium*** (Wallr.) S. Hughes, Canad. J. Bot. 36: 753. 1958.

*Holotypus*: ?STR.

*Type locality*: **Germany**.

*Type substrate*: Decaying *Cucurbita*.  

*chenopodinum Fusarium* (Thüm.) Sacc., Syll. Fung. 4: 701. 1886.

(See ***Fusarium scirpi***)

*Basionym*: *Fusisporium chenopodinum* Thüm., Mycoth. Univ. Cent. 14: no. 1378. 1879.

*Syntypes*: In BPI, CHRB, ILL, NEB, NY, NYS & PUL.

*Type locality*: **Austria**, Niederösterreich, Klosterneuburg.

*Type substrate*: *Chenopodium album.*

*Note*: Synonym *fide*Wollenweber & Reinking (1935).  

*chilense Fusarium* (Mont.) Sacc., Syll. Fung. 4: 716. 1886.

***Gloeosporium chilense*** (Mont.) Wollenw., Z. Parasitenk. (Berlin) 3: 496. 1931.

*Basionym*: *Fusisporium chilense* Mont., in Gay, Fl. Chil. 8: 25. 1852.

*Fusisporium argillaceum* Mont., Bull. Mass. Agric. Exp. Sta. no. 55. 1842, *nom. illegit.*, Art. 53.1, *non Fusarium argillaceum* Fr. 1832.

*Holotypus*: In UPS *fide* Wollenweber, Fusaria Autogr. Delin. 2: 658.

*Type locality*: **Chile**, Juan Fernández Islands.

*Type substrate*: Bark of *Urtica excelsa*.

*Note*: Synonym *fide*Wollenweber & Reinking (1935).  

***chinhoyiense Fusarium*** Yilmaz & Crous, Persoonia 46: 147. 2021.

*Holotypus*: PREM 63215.

*Ex-type culture*: BBA 69031 = DAOM 225149 = Frank 5bCn8 = IMI 375355 = NRRL 25221 = NY007.I2.

*Type locality*: **Zimbabwe**, Chinhoyi.

*Type substrate*: *Zea mays*.

*Descriptions and illustrations*: See Yilmaz *et al.* (2021).

*Diagnostic DNA barcodes*: *rpb1*: MW402711; *rpb2*: MN534262; *tef1*: MN534050.  

***chlamydosporum Fusarium*** Wollenw. & Reinking, Phytopathology 15: 156. 1925.

*Synonyms*: *Fusarium sporotrichioides* var. *chlamydosporum* (Wollenw. & Reinking) Joffe, Mycopathol. Mycol. Appl. 53: 211. 1974, *nom. inval*., Art. 41.1.

*Dactylium fusarioides* Gonz. Frag. & Cif., Bol. Real Soc. Esp. Hist. Nat. 27: 280. 1927.

*Fusarium fusarioides* (Gonz. Frag. & Cif.) C. Booth, The Genus Fusarium: 88. 1971.

*Pseudofusarium purpureum* Matsush., *Microfungi of the Solomon Islands and Papua New Guinea*: 47. 1971.

*Neotypus*: CBS 145.25 (preserved as metabolically inactive culture), designated in Lombard *et al.* (2019a).

*Ex-neotype culture*: CBS 145.25 = NRRL 26851 = NRRL 26912.

*Neotype locality*: **Honduras**, Tela.

*Neotype substrate*: *Musa sapientum.*

*Descriptions and illustrations*: See Wollenweber & Reinking (1925), Booth (1971), Gerlach & Nirenberg (1982) and Leslie & Summerell (2006).

*Diagnostic DNA barcodes*: *rpb1*: MN120715; *rpb2*: MN120735; *tef1*: MN120754.  

*cicatricum Fusarium* (Berk.) O'Donnell & Geiser, Phytopathology 103: 404. 2013.

***Geejayessia cicatricum*** (Berk.) Schroers, Stud. Mycol. 68: 124. 2011.

*Basionym*: *Sphaeria sanguinea* var. *cicatricum* Berk., Mag. Zool. Bot. 1: 48. 1837.

*Synonyms*: *Nectria cicatricum* (Berk.) Tul. & C. Tul., Select. Fung. Carpol. 3: 77. 1865.

*Sphaeria sanguinea* var. *cicatricum* Haller, Syst. Nat., ed 13, 1: LII. 1768.

*Sphaeria coccinea* var. *cicatricum* Desm., Ann. Sci. Nat., Bot., sér. 3, 10: 351. 1848.

*Nectria coccinea* var. *cicatricum* (Desm.) Sacc., Syll. Fung. 2: 482. 1883.

*Cucurbitaria cicatricum* (Desm.) Kuntze, Revis. Gen. Pl. 3: 462. 1898.

*Nectria gibbera* Fuckel, Jahrb. Nassauischen Vereins Naturk. 23–24: 177. 1870.

*Fusarium fuckelii* Sacc., Syll. Fung. 4: 695. 1886.

*Nectria desmazieri* Fuckel ex Sacc., Syll. Fung. 4: 695. 1886, *nom. inval*., Art. 36.1(d).

*Lectotypus*: K(M) 160064 (MBT 10001323 *hic designatus*).

*Epitypus*: CBS H-20374 (MBT 10001324 *hic designatus*).

*Ex-epitype culture*: CBS 125549.

*Epitype locality*: **Slovenia**, Arboretum Volčji Potok.

*Epitype substrate*: Decaying twigs of *Buxus sempervirens.*

*Descriptions and illustrations*: See Schroers *et al.* (2011).

*Diagnostic DNA barcodes*: *rpb1*: KM232231; *rpb2*: HM626679; *tef1*: HM626643.

*Notes*: The epitypification in Schroers *et al.* (2011) was not Code compliant as neither a supporting holo-, lecto- nor epitype was cited. The specimen in the Kew herbarium was cited as isotype. In the protologue a single gathering is mentioned, but an illustration is also cited so a lectotypification is necessary. The epitypification is validated herein.  

*ciliatum Fusarium* (Link) Link, in Willdenow, Sp. Pl., Ed. 4, 6: 105. 1825.

***Scolecofusarium ciliatum*** (Link) L. Lombard *et al.*, Stud. Mycol. 98 (no. 100116): 74. 2021.

*Basionym*: *Atractium ciliatum* Link, Mag. Neuesten Entdeck. Gesammten Naturk. Ges. Naturf. Freunde Berlin 7: 32. 1816.

*Synonyms*: *Microcera ciliata* (Link) Wollenw., Fusaria Autogr. Delin. 1: 435. 1916.

*Calonectria ciliata* (Link) W.C. Snyder & H.N. Hansen, Amer. J. Bot. 32: 664. 1945.

*Sphaeria agnina* Desm., Ann. Sci. Nat., Bot. sér. 3, 6: 72. 1846.

*Calonectria agnina* (Desm.) Sacc., Michelia 1(3): 311. 1878.

*Dialonectria agnina* (Desm.) Cooke, Grevillea 12: 111. 1884.

*Fusarium peltigerae* Westend., Herb. Crypt. Belg. 9: no. 414. 1849.

*Fusarium parasiticum* Westend., Bull. Séances Cl. Sci. Acad. Roy. Sci. Belgique, sér. 2, 11: 652. 1861.

*Nectria massariae* Pass., in Rabenhorst, Fungi Eur. Exs. no. 1827. 1874.

*Microcera massariae* Sacc., Michelia 1(2): 262. 1878.

*Calonectria massariae* (Pass.) Sacc., Michelia 1(3): 312. 1878.

*Fusisporium filisporum* Cooke, Grevillea 8: 8. 1879.

*Fusarium filisporum* (Cooke) Sacc., Syll. Fung. 4: 708. 1886.

*Fusarium scolecoides* Sacc. & Ellis, Atti Reale Ist. Veneto Sci. Lett. Arti, sér. 6, 3: 728. 1885.

*Fusarium elongatum* Cooke, Grevillea 19: 4. 1890.

*Calonectria dearnessii* Ellis & Everh., Proc. Acad. Nat. Sci. Philadelphia 42: 245. 1891.

*Neotypus*: CBS H-12687 designated in this study.

*Ex-neotypus*: ATCC 16068 = ATCC 24137 = BBA 9661 = CBS 191.65 = DSM 62172 = IMI 112499 = NRRL 20431.

*Neotype locality*: **Germany**.

*Neotype substrate*: Branch canker of *Fagus sylvatica.*

*Diagnostic DNA barcodes*: *rpb1*: MW834264*; rpb2*: MW834035*; tef1*: MW834296*.*  

*cinctum Fusarium* Corda, Icon. Fung. 5: 80. 1842.

***Striaticonidium cinctum*** (Corda) L. Lombard & Crous, Persoonia 36: 229. 2016.

*Synonyms*: *Myrothecium cinctum* (Corda) Sacc., Syll. Fung. 4: 751. 1886.

?*Myrothecium ellipsosporum* Fuckel (as ‘*ellipsisporium’*), Fungi Rhen. Exs. Cent. 16, no. 1529 (1865).

?*Hymenopsis ellipsospora* (as ‘*ellipsosporum*’) (Fuckel) Sacc., Syll. Fung. 4: 745. 1886.

*Myrothecium striatisporum* N.C. Preston, Trans. Brit. Mycol. Soc. 31: 275. 1948.

*Myrothecium longistriatisporum* Matsush., Microfungi Solomon Isl. Papua-New Guinea: 39. 1971.

*Lectotypus*: PR 155489, designated in Tulloch (1972).

*Epitypus*: CBS H-22471, designated in Lombard *et al.* (2016).

*Ex-epitype culture*: CBS 932.69 = IMI 145760.

*Epitype locality*: **Netherlands**, Eastern Flevoland.

*Epitype substrate*: Agricultural soil.

*Note*: The lectotype was cited as holotype in Lombard *et al.* (2016) but this is correctable according to Art. 9.10 of the Code (see also Ex. 11).  

*cinnabarinum Fusarium* (Berk. & M.A. Curtis) Sacc., Syll. Fung. 4: 722. 1886.

(See ***Fusarium lateritium***)

*Basionym*: *Fusisporium cinnabarinum* Berk. & M.A. Curtis, Grevillea 3: 146. 1875.

*Syntypes*: In PH, Pul & USCH:Fungi (Ellis, N. Amer. F. 3990).

*Type locality*: **USA**, Alabama.

*Type substrate*: *Acer negundo.*

*Note*: Synonym *fide*Wollenweber & Reinking (1935).  

***circinatum Fusarium*** Nirenberg & O'Donnell, Mycologia 90: 442. 1998.

*Synonyms*: *Gibberella circinata* Nirenberg & O'Donnell, Mycologia 90: 440. 1998, *nom. inval.*, Art. 40.3.

*Gibberella circinata* Nirenberg & O'Donnell ex Britz *et al.*, Sydowia 54: 16. 2002.

*Holotypus*: B 70 0001693.

*Ex-type culture*: BBA 69720 = CBS 405.97 = DAOM 225113 = IMI 375321 = NRRL 25331.

*Type locality*: **USA**, California.

*Type substrate*: *Pinus radiata.*

*Descriptions and illustrations*: See Nirenberg & O'Donnell (1998).

*Diagnostic DNA barcodes*: *rpb1*: JX171510; *rpb2*: JX171623; *tef1*: AF160295.  

*cirrosum Fusarium* Höhn., Sitzungsber. Kaiserl. Akad. Wiss. Wien, Math.-Naturwiss. Cl., Abt. 1., 116: 153. 1907.

(See ***Fusarium expansum***)

*Holotypus*: FH00284266.

*Type locality*: **Austria**, Niederösterreich, Irenental near Untertullnerbach.

*Type substrate*: Parasictic in the acervuli of *Steganosporium pyriforme* (syn. *Steganosporium ovatum*).

*Note*: Synonym *fide*Wollenweber & Reinking (1935).  

***citri Fusarium*** M.M. Wang *et al.*, Persoonia 43: 79. 2019.

*Holotypus*: HAMS 248036.

*Ex-type culture*: CGMCC 3.19467 = LC6896.

*Type locality*: **China**, Hunan Province.

*Type substrate*: Leaves of *Citrus reticulata.*

*Descriptions and illustrations*: See Wang *et al.* (2019).

*Diagnostic DNA barcodes*: *rpb1*: MK289828; *rpb2*: MK289771; *tef1*: MK289617.  

***citricola Fusarium*** Guarnaccia *et al.*, Persoonia 40: 12. 2017. [2018].

*Holotypus*: CBS H-23020.

*Ex-type culture*: CBS 142421 = CPC 27805.

*Type locality*: **Italy**, Cosenza, Rocca Imperiale.

*Type substrate*: *Citrus reticulata* ‘Caffin’*.*

*Descriptions and illustrations*: See Sandoval-Denis *et al.* (2018a).

*Diagnostic DNA barcodes*: *rpb1*: LT746290; *rpb2*: LT746310; *tef1*: LT746197.  

*citriforme Fusarium* Jamal., Valt. Maatalousk. Julk. 123: 11. 1943.

(See ***Fusarium tricinctum***)

*Lectotypus* (*hic designatus*, MBT 10000668): **Finland**, Pyhajärvi, *Hordeum sativum*, 1938, E. Jamalainen, in Valt. Maatalousk. Julk. 123: 10. 1943, fig. 2.

*Ex-type culture*: CBS 253.50.

*Diagnostic DNA barcodes*: *rpb1*: MW928802; *rpb2*: MW928823; *tef1*: KR071775.

*Notes*: Jamalainen (1943) cited various specimens in the protologue of *F. citriforme*, but failed to indicate a holotype. Therefore, a lectotypification is done here to fix the name. Isolate CBS 253.50 was deposited in the public collection of CBS by E. Jamalainen in 1950. The isolate was indicated as the living ex-type culture of *F. citriforme*.  

*citrinum Fusarium* Wollenw., in Lewis, Bull. Maine Agric. Exp. Sta. 219: 256. 1913.

(See ***Fusarium oxysporum***)

*Lectotypus* (*hic designatus*, MBT 10000669): **Germany**, Berlin, Dahlem, rotten fruit of *Solanum lycopersicum*, Oct. 1910, H.W. Wollenweber, B70 0100185.

*Notes*: Synonym *fide*Wollenweber & Reinking (1935). Only one specimen located at B matches the original collection event, but it is not indicated as the type. Therefore B 70 0100185 is designated as lectotype here.  

*citrulli Fusarium* Taubenh., Bull. Texas Agric. Exp. Sta. 260: 27. 1920.

(See ***Fusarium oxysporum***)

*Lectotypus* (*hic designatus*, MBT 10000670): **USA**, Texas, Waller County, seedlings of *Citrullus lanatus*, 1920, J.J. Taubenhaus, in Bull. Texas Agric. Exp. Sta. 260: 30, fig. 8h.

*Notes*: Synonym *fide*Wollenweber & Reinking (1935). No holotype specimen could be located and therefore an illustration is designated as lectotype.  

*citrulli Fusarium* Sartory, Compt. Rend. Hebd. Séances Acad. Sci. 188: 1434. 1929, *nom. inval*., Art. 35.2; *nom. illegit.*, Art. 53.1.

***Neocosmospora martii*** (Appel & Wollenw.) Sand.-Den. & Crous, Persoonia 43: 142. 2019.

*Basionym*: *Fusarium martii* Appel & Wollenw., Arbeiten Kaiserl. Biol. Anst. Land-Forstw. 8: 83. 1910.

*Synonyms*: *Fusarium solani* var. *martii* (Appel & Wollenw.) Wollenw., Fusaria Autogr. Delin. 3: 1034. 1930.

?*Selenosporium fuscum* Bonord., Handb. Mykol.: 135. 1851.

?*Fusarium fuscum* (Bonord.) Sacc., Syll. Fung. 4: 699. 1886.

*Fusarium citrulli* Sartory & J. Mey., Compt. Rend. Soc. Biol. 107: 55. 1931, *nom. illegit.*, Art. 53.1, *non Fusarium citrulli* Taubenh. 1920.

*Neocosmospora croci* Guarnaccia *et al.*, Persoonia 40: 17. 2017 [2018].

*Authentic material*: Not located.

*Original locality*: **France**.

*Original substrate*: *Citrullus vulgaris*.

*Note*: Synonyms *fide*Wollenweber & Reinking (1935) and Sandoval-Denis *et al.* (2019).  

*clavatum Fusarium* Sherb., Mem. Cornell Univ. Agric. Exp. Sta. 6: 234. 1915.

(See ***Fusarium flocciferum***)

*Lectotypus* (*hic designatus*, MBT 10000671): **USA**, New York, Castile, rotten tuber of *Solanum tuberosum*, 1915, C.D. Sherbakoff, in Mem. Cornell Univ. Agric. Exp. Sta. 6: 235, fig. 40.

*Notes*: Synonym *fide*Wollenweber & Reinking (1935). No holotype specimen could be located and therefore an illustration is designated as lectotype.  

***clavus Fusarium*** J.W. Xia *et al.* (as ‘*clavum*’), Persoonia 43: 199. 2019.

*Holotypus*: CBS H-24054.

*Ex-type culture*: CBS 126202 = RMF N 38.

*Type locality*: **Namibia**, northern Karoo, 30 km west of Maltahöhe.

*Type substrate*: Desert soil*.*

*Descriptions and illustrations*: See Xia *et al.* (2019).

*Diagnostic DNA barcodes*: *rpb2*: MN170389; *tef1*: MN170456.  

*clematidis Fusarium* Rolland & Fautrey, Rev. Mycol. (Toulouse) 16: 72. 1894.

***Macroconia sphaeriae*** (Fuckel) Gräfenhan & Schroers, Stud. Mycol. 68: 103. 2011.

*Basionym*: *Fusarium sphaeriae* Fuckel, Fungi Rhen. Exs. Fasc. 3: no. 212. 1863.

*Synonyms*: *Fusarium sphaeriae* var. *robustum* Davis, Trans. Wisconsin Acad. Sci. 19: 714. 1919.

*Septogloeum robustum* (Davis) Wollenw. & Reinking, *Fusarien*: 336. 1935.

?*Nectria leptosphaeriae* var. *macrospora* Wollenw., Angew. Bot. 8: 187. 1926.

*Syntype*: ILL00220727 (Roumeguère, Fungi Sel. Gall. Exs. no. 6537).

*Type locality*: **France**.

*Type substrate*: *Clematis vitalba.*

*Note*: Synonym *fide*Wollenweber & Reinking (1935).  

*clypeaster Fusarium* (Corda) Sacc., Syll. Fung. 4: 706. 1886.

***Septogloeum clypeaster*** (Corda) Wollenw., *Fusarien*: 321. 1935.

*Basionym*: *Fusisporium clypeaster* Corda, Icon. Fung. 4: 26. 1840.

*Lectotypus* (*hic designatus*, MBT 10000672): **Czech Republic**, *Phragmites*, May 1839, A.C.J. Corda, in Icon. Fung. 4, Tab. 6, fig. 82. 1840.

*Notes*: Synonym *fide*Wollenweber & Reinking (1935). No holotype specimen could be located and therefore an illustration is designated as lectotype.  

*coccidicola Fusarium* Henn. (as ‘*coccideicola*’), Bot. Jahrb. Syst. 34: 57. 1904.

***Microcera diploa*** (Berk. & M.A. Curtis) Gräfenhan & Seifert, Stud. Mycol. 68: 106. 2011.

*Basionym*: *Nectria diploa* Berk. & M.A. Curtis, J. Linn. Soc., Bot. 10: 378. 1868.

*Synonyms*: *Cucurbitaria diploa* (Berk. & M.A. Curtis) Kuntze, Revis. Gen. Pl. 3: 461. 1898.

*Creonectria diploa* (Berk. & M.A. Curtis) Seaver, Mycologia 1: 190. 1909.

*Calonectria diploa* (Berk. & M.A. Curtis) Wollenw., Angew. Bot. 8: 193. 1926.

*Cosmospora diploa* (Berk. & M.A. Curtis) Rossman & Samuels, Stud. Mycol. 42: 121. 1999.

*Fusarium derridis* Henn., Beibl. Hedwigia 41: (66). 1902.

*Fusarium juruanum* Henn., Hedwigia 43: 398. 1904.

*Fusarium pentaclethrae* Henn., Hedwigia 44: 71. 1905.

*Aschersonia henningsii* Koord., Verh. Kon. Ned. Akad. Wetensch., Afd. Natuurk. 13: 213. 1907.

*Microcera henningsii* (Koord.) Petch, Ann. Roy. Bot. Gard. (Peradeniya) 5: 533. 1914.

*Pseudomicrocera henningsii* (Koord.) Petch, Trans. Brit. Mycol. Soc. 7: 100. 1921.

*Microcera fujikuroi* Miyabe & Sawada, J. Fac. Agric. Hokkaido Imp. Univ. 5: 83. 1913.

*Microcera merrillii* Syd. & P. Syd., Ann. Mycol. 12(6): 576. 1914.

*Pseudomicrocera henningsii* var. *longispora* Petch, Trans. Brit. Mycol. Soc. 7: 164. 1921.

*Fusarium microcera* Bilaĭ, Fusarii (Biologija i sistematika): 292. 1955, *nom. inval*., Art. 39.1.

*Holotypus*: Zimmerman no. 26 in B *fide*Hein (1988).

*Type locality*: **Tanzania**, East Usambara, Magrotto.

*Type substrate*: Parasitic on *Coccoidea* sp. on *Camellia sinensis.*  

*coccinellum Fusarium* Kalchbr., Flora (Regensburg) 59: 426. 1876.

(See *Fusarium coccophilum*)

*Syntype*: ?NY00899913.

*Type locality*: **South Africa**, Eastern Cape Province, Somerset-East.

*Type substrate*: *Acacia horrida.*

*Note*: Synonym *fide*Wollenweber & Reinking (1935).  

*coccineum Fusarium* Schwein., Trans. Amer. Philos. Soc., n.s. 4: 302. 1834.

*Holotypus*: ?PH00062490.

*Type locality*: **USA**, Pennsylvania, Northhampton, Nazareth.

*Type substrate*: Bark of *Castanea* sp*.*

*Notes*: Status unclear. Not *Fusarium fide*Wollenweber & Reinking (1935).  

*coccophilum Fusarium* (Desm.) Wollenw. & Reinking, *Fusarien*: 34. 1935.

***Microcera coccophila*** Desm., Ann. Sci. Nat. Bot., sér. 3, 10: 359. 1848.

*Synonyms*: *Tubercularia coccophila* (Desm.) Bonord., Abh. Naturf. Ges. Halle 8: 96. 1864.

*Fusarium episphaeria f*. *coccophilum* (Desm.) W.C. Snyder & H.N. Hansen, Amer. J. Bot. 32: 662. 1945.

*Nectria episphaeria f*. *coccophila* (Desm.) W.C. Snyder & H.N. Hansen, Amer. J. Bot. 32: 662. 1945.

*Fusarium coccinellum* Kalchbr., Flora (Regensburg) 56: 426. 1876.

*Fusisporium coccinellum* (Kalchbr.) Kalchbr., in Thümen, Mycoth. Univ. no. 782. 1877.

*Fusarium cataleptum* Cooke & Harkn., Grevillea 12: 96. 1884.

*Microcera pluriseptata* Cooke & Massee, Grevillea 17: 43. 1888.

*Fusarium callosporum* Pat., Bull. Soc. Mycol. France 9: 164. 1893.

*Fusarium baccharidicola* Henn., Hedwigia 48: 20. 1908.

*Microcera coccophila* var. *platyspora* Sousa da Câmara, Revista Agron. (Lisbon): 5 (extr.). 1920.

*Lectotypus*: K(M) 165807, designated in Gräfenhan *et al.* (2011).

*Type locality*: **France**, Normandy, near Caen.

*Type substrate*: Parasitic on *Eulecanium tiliae* on *Salix* sp. and *Fraxinus excelsior.*

*Descriptions and illustrations*: See Gräfenhan *et al.* (2011).

*Notes*: No living type material available. Gräfenhan *et al.* (2011) designated a lectotype but did not designate an epitype, which is still required.  

***coffeatum Fusarium*** L. Lombard & Crous, Fungal Syst. Evol. 4: 191. 2019.

*Replaced synonym*: *Fusarium chlamydosporum* var. *fuscum* Gerlach, Phytopathol. Z. 90: 41. 1977.

*Holotypus*: BBA 62053.

*Isotypus*: CBS H-631.

*Ex-type culture*: BBA 62053 = CBS 635.76 = NRRL 20841.

*Type locality*: **South Africa**.

*Type substrate*: *Cynodon lemfuensis.*

*Descriptions and illustrations*: See Gerlach (1977a), Gerlach & Nirenberg (1982) and Xia *et al.* (2019).

*Diagnostic DNA barcodes*: *rpb1*: MN120717; *rpb2*: MN120736; *tef1*: MN120755.  

*coffeicola Fusarium* Henn., Bot. Jahrb. Syst. 22: 82. 1895.

*Synonym*: *Gloeosporium coffeicola* (P. Henn.) Wollenw., Fusaria Autogr. Delin. 1: 493. 1916, *nom. illegit.*, Art. 53.1, *non Gloeosporium coffeicola* Tassi 1900.

*Holotypus*: In B *fide*Hein (1988).

*Type locality*: **Cameroon**, Victoria.

*Type substrate*: *Coffea liberica.*

*Notes*: Status unclear. Not *Fusarium fide*Wollenweber & Reinking (1935).  

***coicis Fusarium*** Johanssen *et al.*, Fungal Diversity 77: 356. 2015 [2016].

*Holotypus*: RBG 5368.

*Ex-type culture*: FRL 19329 = NRRL 66233 = RBG 5368.

*Type locality*: **Australia**, Queensland, Mareeba.

*Type substrate*: *Coix gasteenii.*

*Descriptions and illustrations*: See Laurence *et al.* (2016).

*Diagnostic DNA barcodes*: *rpb1*: KP083269; *rpb2*: KP083274; *tef1*: KP083251.  

*colorans Fusarium* (De Jonge) Appel & Wollenw., Arbeiten Kaiserl. Biol. Anst. Land-Forstw. 8: 39. 1913.

***Albonectria rigidiuscula*** (Berk. & Broome) Rossman & Samuels, Stud. Mycol. 42: 105. 1999.

*Basionym*: *Nectria rigidiuscula* Berk. & Broome, J. Linn. Soc., Bot. 14: 116. 1873 [1875]. *Synonyms*: *Calonectria rigidiuscula* (Berk. & Broome) Sacc., Michelia 1(3): 313. 1878.

*Fusarium rigidiusculum* (Berk. & Broome) W.C. Snyder & H.N. Hansen, Amer. J. Bot. 32: 664. 1945.

*Calonectria eburnea* Rehm, Hedwigia 37: 196. 1898.

*Calonectria lichenigena* Speg., Bol. Acad. Nac. Ci. Republ. Argent. 11: 530. 1889.

*Calonectria sulcata* Starbäck, Bih. Kongl. Svenska Vetensk.-Akad. Handl. 25: 29. 1899.

*Fusarium decemcellulare* Brick, Jahresber. Vereinigung Angew. Bot. 6: 227. 1908.

*Spicaria colorans* De Jonge, Recueil Trav. Bot. Néerl. 6: 48. 1909.

*Scoleconectria tetraspora* Seaver, N. Amer. Fl. 3: 27. 1910.

*Calonectria tetraspora* (Seaver) Sacc. & Trotter, Syll. Fung. 22: 487. 1913.

*Nectria rigidiuscula f*. *theobromae* E.J. Ford *et al.*, Phytopathology 57: 712. 1967.

*Holotypus*: Not located.

*Type locality*: **Surinam**.

*Type substrate*: *Theobroma cacao.*

*Notes*: Synonym *fide*Wollenweber & Reinking (1935). Wollenweber (1916–1935) indicated that cultures and specimens of *Spicaria colorans* (basionym of *F. colorans*) were deposited in the Willie Commelin Scholten collection in Amsterdam. This collection has been accessioned into the CBS collection (CBS & CBS H). However, no cultures and specimens or records could be located at CBS.  

***commune Fusarium*** K. Skovg. *et al.*, Mycologia 95: 632. 2003.

*Holotypus*: BBA 71639 in B.

*Ex-type culture*: AAS 156 = BBA 71639 = CBS 110090 = NRRL 31076.

*Type locality*: **Denmark**.

*Type substrate*: Soil*.*

*Descriptions and illustrations*: See Skovgaard *et al.* (2003).

*Diagnostic DNA barcodes*: *rpb1*: MW928803; *rpb2*: MW934368; *tef1*: AF362263.  

*commutatum Fusarium* Sacc., Syll. Fung. 4: 710. 1886.

(See *Fusarium solani*)

*Replaced synonym*: *Fusisporium candidum* Bonord., Handb. Allg. Mykol.: 96 (1851), *nom. illegit.*, Art. 53.1, *non Fusisporium candidum* Link 1824.

*Holotypus*: Not located.

*Type locality*: **Germany**.

*Type substrate*: *Solanum tuberosum.*

*Note*: Synonyms *fide*Wollenweber & Reinking (1935) and Sandoval-Denis *et al.* (2019).  

***compactum Fusarium*** (Wollenw.) Raillo, Fungi of the Genus Fusarium: 180. 1950.

*Basionym*: *Fusarium scirpi* var. *compactum* Wollenw., Fusaria Autogr. Delin. 3: no. 924. 1930.

*Synonym*: *Fusarium compactum* (Wollenw.) Gordon, Canad. J. Bot. 30: 224. 1952, *nom. inval*., Art. 53.1.

*Lectotypus*: Illustration in Wollenweber, Fusaria Autogr. Delin. no. 924 (1930), designated in Xia *et al.* 2019.

*Epitypus*: CBS 186.31 (preserved as metabolically inactive culture), designated in Xia *et al.* (2019).

*Ex-epitype culture*: CBS 186.31 = NRRL 36323.

*Epitype locality*: **UK**, Kew.

*Epitype substrate*: Cotton thread*.*

*Descriptions and illustrations*: See Wollenweber (1916–1935, no. 924), Raillo (1950), Gordon (1952), Gerlach & Nirenberg (1982) and Leslie & Summerell (2006).

*Diagnostic DNA barcodes*: *rpb2*: GQ505826; *tef1*: GQ505648.  

***concentricum Fusarium*** Nirenberg & O'Donnell, Mycologia 90: 442. 1998.

*Holotypus*: B 70 0001694.

*Ex-type culture*: BBA 64354 = CBS 450.97 = DAOM 225146 = IMI 375352 = NRRL 25181.

*Type locality*: **Costa Rica**.

*Type substrate*: *Musa sapientum.*

*Descriptions and illustrations*: See Nirenberg & O'Donnell (1998) and Leslie & Summerell (2006).

*Diagnostic DNA barcodes*: *rpb1*: LT996192; *rpb2*: LT575063; *tef1*: AF160282.  

***concolor Fusarium*** Reinking, Zentralbl. Bakteriol., 2. Abt. 89: 512. 1934.

*Synonym*: *Fusarium polyphialidicum* Marasas *et al.*, Mycologia 78: 678. 1986.

*Holotypus*: IMI 112502.

*Ex-type culture*: BBA 2607 = BBA 63601 = CBS 183.34 = DAOM 225131 = DSM 62179 = IMI 112502 = NRRL 13994.

*Type locality*: **Uruguay**, Montevideo.

*Type substrate*: *Hordeum vulgare.*

*Descriptions and illustrations*: See Gerlach & Nirenberg (1982) and Marasas *et al.* (1986).

*Diagnostic DNA barcodes*: *rpb1*: MH742492; *rpb2*: MH742569; *tef1*: MH742650.  

*conglutinans Fusarium* Wollenw., Ber. Deutsch. Bot. Ges. 31: 34. 1913.

(See ***Fusarium oxysporum***)

*Holotypus*: Not located.

*Type locality*: **USA**, Wisconsin.

*Type substrate*: *Brassica oleracea* var. *capitata.*  

*congoense Fusarium* Wollenw., Fusaria Autogr. Delin. 1: 307. 1916.

(See ***Fusarium heterosporum***)

*Syntype*: BPI 451889.

*Type locality*: **Democratic Republic of the Congo**.

*Type substrate*: *Bromus willdenowii*.

*Note*: Synonyms *fide*Wollenweber & Reinking (1935).  

*coniosporiicola Fusarium* Henn., Ann. Mus. Congo Belge, Bot., Sér. 5, 2: 106. 1907.

***Dendrodochium coniosporiicola*** (Henn.) Hansf., Proc. Linn. Soc. London 155: 60. 1943.

*Synonym*: *Fusidium coniosporiicola* (Henn.) Wollenw., Fusaria Autogr. Delin. 1: 477. 1916.

*Syntypes*: In BR & S.

*Type locality*: **Democratic Republic of the Congo**, Gongolo.

*Type substrate*: *Albizia* aff. *fastigiata.*  

*constrictum Fusarium* Penz., Michelia 2: 486. 1882.

*Synonym*: *Ramularia constricta* (Penz.) Wollenw., *Fusarien*: 322. 1935.

*Holotypus*: Not located; destroyed *fide* U. Braun.

*Type locality*: **Italy**, Padua.

*Type substrate*: Leaves of *Citrus* sp*.*

*Notes*: Status unclear. Neither *Fusarium fide*Wollenweber & Reinking (1935) nor *Ramularia* (pers. comm. U. Braun).  

***contaminatum Fusarium*** L. Lombard & Crous, Persoonia 43: 20. 2018 [2019].

*Holotypus*: CBS H-23610.

*Ex-type culture*: CBS 114899.

*Type locality*: **Germany**, Schlüchtern.

*Type substrate*: Pasteurised chocolate milk*.*

*Descriptions and illustrations*: See Lombard *et al.* (2019b).

*Diagnostic DNA barcodes*: *rpb2*: MH484901; *tef1*: MH484992.  

***continuum Fusarium*** X. Zhou *et al.*, Mycologia 108: 677. 2016.

*Holotypus*: HMNWAFU *NX-Ffpl-10-20100851*.

*Ex-type culture*: CBS 140841 = F201030 = NRRL 66286.

*Type locality*: **China**, Shaanxi, Fuping, Lei village.

*Type substrate*: *Zanthoxylum bungeanum.*

*Descriptions and illustrations*: See Zhou *et al.* (2016).

*Diagnostic DNA barcodes*: *rpb1*: KM520387; *rpb2*: KM236782; *tef1*: KM236722.  

***convolutans Fusarium*** Sand.-Den. *et al.*, MycoKeys 34: 77. 2018.

*Holotypus*: CBS H-23495.

*Ex-type culture*: CBS 144207 = CPC 33733.

*Type locality*: **South Africa**, Kruger National Park, Skukuza, Granite Supersite.

*Type substrate*: Rhizosphere of *Kyphocarpa angustifolia.*

*Descriptions and illustrations*: See Sandoval-Denis *et al.* (2018b).

*Diagnostic DNA barcodes*: *rpb1*: LT996193; *rpb2*: LT996141; *tef1*: LT996094.  

*corallinum Fusarium* Mattir., Atti Accad. Sci. Ist. Bologna, Cl. Sci. Fis., Mem. 6: 677. 1897, *nom. illegit.*, Art. 53.1.

(See *Fusarium culmorum*)

*Authentic material*: Not located.

*Type locality*: **Italy**.

*Type substrate*: *Andropogon* sp*.*

*Note*: Synonym *fide* Wollenweber (1931).  

*corallinum Fusarium* Sacc., Nuovo Giorn. Bot. Ital. 8: 196. 1876.

(See ***Fusarium graminum***)

*Holotypus*: In PAD.

*Type locality*: **Italy**, Treviso, Selva.

*Type substrate*: *Cynodon dactylon.*  

*cordae Fusarium* Massee, Brit. Fung.-Fl. 3: 481. 1893.

(See ***Fusarium oxysporum***)

*Notes*: Massee introduced this name to replace *F. aurantiacum* Corda, indicating that *F. aurantiacum* (Link) Sacc., based on *Fusisporium aurantiacum* Link (1809), predates Corda's use of the epithet. However, Corda's use of the epithet in *Fusarium* predates Saccardo's recombination into *Fusarium*.  

***cortaderiae Fusarium*** O'Donnell *et al.*, Fungal Genet. Biol. 41: 620. 2004.

*Holotypus*: BPI 843479.

*Ex-type culture*: CBS 119183 = ICMP 5435 = NRRL 29297.

*Type locality*: **New Zealand**, Auckland, Henderson.

*Type substrate*: *Cortaderia selloana.*

*Descriptions and illustrations*: See O'Donnell *et al.* (2004).

*Diagnostic DNA barcodes*: *rpb1*: KM361644; *rpb2*: KM361662; *tef1*: AY225885.  

*crassistipitatum Fusarium* Scandiani *et al.*, Mycoscience 53: 171. 2011.

(See *Fusarium azukiicola*)

*Holotypus*: BPI 871490.

*Ex-type culture*: MAFF 239757 = NRRL 36877.

*Type locality*: **Argentina**, Santa Fe, Zavalla.

*Type substrate*: *Glycine max*.

*Descriptions and illustrations*: See Aoki *et al.* (2012a).

*Diagnostic DNA barcodes*: *rpb2*: FJ240405; *tef1*: FJ240351.  

*crassum Fusarium* (Sand.-Den. & Crous) O'Donnell *et al.*, Index Fungorum 440: 1. 2020.

***Neocosmospora crassa*** Sand.-Den. & Crous, Persoonia 43: 122. 2019.

*Holotypus*: CBS H-23976.

*Ex-type culture*: CBS 144386 = MUCL 11420.

*Type locality*: **France**, Paris.

*Type substrate*: Unknown.

*Descriptions and illustrations*: See Sandoval-Denis *et al.* (2019).

*Diagnostic DNA barcodes*: *rpb1*: MW218109; *rpb2*: LR583823; *tef1*: LR583604.  

***croceum Fusarium*** J.W. Xia *et al.*, Persoonia 43: 201. 2019.

*Holotypus*: CBS H-24055.

*Ex-type culture*: CBS 131777.

*Type locality*: **Iran**, Golestan Province, Gonbad-e Qabus.

*Type substrate*: *Triticum* sp*.*

*Descriptions and illustrations*: See Xia *et al.* (2019).

*Diagnostic DNA barcodes*: *rpb2*: MN170396; *tef1*: MN170463.  

*croci Fusarium* (Guarnaccia, Sand.-Den. & Crous) O'Donnell *et al.*, Index Fungorum 440: 1. 2020.

*Basionym*: *Neocosmospora croci* Guarnaccia, Sand.-Den. & Crous, Persoonia 40: 17. 2017.

(See *Fusarium citrulli* Sartory)

*Holotypus*: CBS H-23022.

*Ex-type culture*: CBS 142423 = CPC 27186.

*Type locality*: **Italy**, Sicily, Catania, Paternó.

*Type substrate*: *Citrus sinensis.*

*Descriptions and illustrations*: See Sandoval-Denis *et al.* (2018a).

*Diagnostic DNA barcodes*: *rpb2*: LT746329; *tef1*: LT746216.  

*cromyophthoron Fusarium* Sideris, Phytopathology 14: 212. 1924.

(See ***Fusarium oxysporum***)

*Lectotypus* (*hic designatus*, MBT 10000673): **USA**, California, Stockton, roots of *Allium* sp*.,*1924, C.P. Sideris, in Phytopathology 14, pl. IX.

*Notes*: Synonym *fide*Wollenweber & Reinking (1935). No holotype specimen could be located and therefore an illustration is designated as lectotype.  

*crookwellense Fusarium* L.W. Burgess *et al.*, Trans. Brit. Mycol. Soc. 79: 498. 1982.

(See ***Fusarium cerealis (Cooke) Sacc.***)

*Holotypus*: FRC R-3090.

*Ex-type culture*: NRRL 13163.

*Type locality*: **Australia**, New South Wales, Crookwell.

*Type substrate*: *Solanum tuberosum* tubers*.*

*Descriptions and illustrations*: See Burgess *et al.* (1982).

*Note*: See *Note*s under *F. cerealis*.  

*cruentum Fusarium* Teich, Byull. Sredne-Aziatsk. Gosud. Univ. 19: 178. 1934.

*Holotypus*: Not located.

*Type locality*: **Uzbekistan**, Tashkent.

*Type substrate*: Roots and stems of *Vitis vinifera.*

*Notes*: Status unclear. This species was not treated by any of Wollenweber & Reinking (1935), Raillo (1950), Bilaĭ (1955), Booth (1971), Joffe (1974), or Gerlach & Nirenberg (1982). Furthermore, no additional records could be located.  

*cryptoseptatum Fusarium* (Sand.-Den. & Crous) O'Donnell, Index Fungorum 440: 1. 2020.

***Neocosmospora cryptoseptata*** Sand.-Den. & Crous, Persoonia 43: 122. 2019.

*Holotypus*: CBS H-23977.

*Ex-type culture*: BBA 65024 = CBS 145463 = NRRL 22412.

*Type locality*: **French Guiana**.

*Type substrate*: Bark*.*

*Descriptions and illustrations*: See Sandoval-Denis *et al.* (2019).

*Diagnostic DNA barcodes*: *rpb1*: MW834215; *rpb2*: EU329510; *tef1*: AF178351.  

*cryptum Fusarium* McAlpine, Fungus Diseases of Citrus trees in Australia: 106. 1899.

(See ***Fusarium larvarum***)

*Holotypus*: VPRI 2557.

*Type locality*: **Australia**, South Australia.

*Type substrate*: Twigs of *Citrus limonia.*

*Note*: Synonym *fide*Wollenweber & Reinking (1935).  

*cubense Fusarium* E.F. Sm., Science, N.Y. 31: 754. 1910.

(See ***Fusarium oxysporum***)

*Holotypus*: Not located.

*Type locality*: **Cuba**.

*Type substrate*: *Musa* sp*.*

*Note*: Synonym *fide*Wollenweber & Reinking (1935).  

*cucumerinum Fusarium* Berk. & Broome, Ann. Mag. Nat. Hist., ser. 4, 17: 141. 1876.

*Holotypus*: ?K(M).

*Type locality*: **UK**, Northamptonshire, Daventry, Sibbertoft.

*Type substrate*: Diseased *Cucumis sativus.*

*Notes*: Status unclear. Wollenweber & Reinking (1935) synonymised this species under *Septomyxa persicina*. In contrast, Index Fungorum indicates that this species is a synonym under *F. oxysporum*. The original protologue (Berkeley & Broome 1876) fits neither *S. persicina* nor *F. oxysporum*.  

*cucurbitae Fusarium* Taubenh., Bull. Texas Agric. Exp. Sta. 260: 27. 1920.

*Lectotypus* (*hic designatus*, MBT 10000674): **USA**, Texas, Waller County, from squash, date unkown, J.J. Taubenhaus, in Bull. Texas Agric. Exp. Sta. 260: 30, fig. 8j. 1920.

*Notes*: Based on the description and illustrations provided by Taubenhaus (1920), this species could represent *F. oxysporum*. However, recollection and epitypification are required to confirm this. No holotype specimen could be located and therefore an illustration is designated as lectotype.  

*cucurbitariae Fusarium* (Pat.) Sacc., Syll. Fung. 4: 708. 1886.

(See ***Fusarium avenaceum***)

*Basionym*: *Fusisporium cucurbitariae* Pat., Rev. Mycol. (Toulouse) 3: 10. 1881.

*Holotypus*: ?FH01093588.

*Type locality*: **France**, Lons-le-Saunier.

*Type substrate*: Diseased *Cucumis sativus.*

*Note*: Synonym *fide*Wollenweber & Reinking (1935).  

*cucurbitariae Fusarium* Peyronel, Nuovo Giorn. Bot. Ital., n.s. 25: 436. 1918, *nom. illegit.*, Art. 53.1.

*Holotypus*: ?ROPV.

*Type locality*: **Italy**, Piemonte, Riclaretto.

*Type substrate*: Parasitic on perithecia of *Camarosporidiella laburni* (≡ *Cucurbitaria laburni*)*.*

*Notes*: Status unclear. Not treated by any of Wollenweber & Reinking (1935), Booth (1971), or Gerlach & Nirenberg (1982).  

*cucurbiticola Fusarium* O'Donnell *et al.*, Index Fungorum 440: 2. 2020.

***Neocosmospora cucurbitae*** Sand.-Den. *et al.*, Persoonia 43: 125. 2019.

*Synonyms*: *Fusarium solani f*. *cucurbitae* W.C. Snyder & H.N. Hansen, Amer. J. Bot. 28: 740. 1941.

*Fusarium solani f. sp*. *cucurbitae* W.C. Snyder & H.N. Hansen, Root rots caused by Phycomycetes 28: 740. 1941.

*Hypomyces solani f*. *cucurbitae* W.C. Snyder & H.N. Hansen, Amer. J. Bot. 28: 741. 1941.

*Nectria haematococca* var. *cucurbitae* (W.C. Snyder & H.N. Hansen) Dingley, New Zealand J. Agric. Res. 4: 337. 1961.

*Nectria solani f. cucurbitae* (W.C. Snyder & H.N. Hansen) G.R.W. Arnold, Z. Pilzk. 37: 193. 1972.

*Holotypus*: CBS H-23978.

*Ex-type culture*: BBA 64411 = CBS 616.66 = NRRL 22399.

*Type locality*: **Netherlands**.

*Type substrate*: *Cucurbita viciifolia.*

*Descriptions and illustrations*: See Sandoval-Denis *et al.* (2019).

*Diagnostic DNA barcodes*: *rpb1*: MW834217; *rpb2*: LR583825; *tef1*: DQ247592.  

***cugenangense Fusarium*** Maryani *et al.*, Stud. Mycol. 92: 181. 2018 [2019].

*Holotypus*: InaCC F984 (preserved as metabolically inactive culture).

*Ex-type culture*: InaCC F984.

*Type locality*: **Indonesia**, West Java, Cianjur, Cugenang.

*Type substrate*: Pseudostem of *Musa* var. Pisang Kepok*.*

*Descriptions and illustrations*: See Maryani *et al.* (2019a).

*Diagnostic DNA barcodes*: *rpb1*: LS479560; *rpb2*: LS479308; *tef1*: LS479757.  

***culmorum Fusarium*** (Wm.G. Sm.) Sacc., Syll. Fung. 10: 726. 1892.

*Basionym*: *Fusisporium culmorum* Wm.G. Sm., *Diseases of field and garden crops, chiefly as are caused by fungi*: 209. 1884.

*Synonyms*: *Fusarium schribauxii* Delacr., Bull. Soc. Mycol. France 6: 99. 1890.

*Fusarium corallinum* Mattir., Atti Accad. Sci. Ist. Bologna, Cl. Sci. Fis., Mem. 6: 677. 1897, *nom. illegit.*, Art. 53.1.

*Fusarium versicolor* Sacc., Syll. Fung. 16: 1099. 1902.

*Fusarium heidelbergense* Sacc., Ann. Mycol. 8: 346. 1910.

?*Fusarium neglectum* Jacz., Bull. Trimestriel Soc. Mycol. France 28: 348. 1912.

*Fusarium rubiginosum* Appel & Wollenw., Arbeiten Kaiserl. Biol. Anst. Land-Forstw. 8: 108. 1910 [1913].

*Fusarium culmorum* var. *leteius* Sherb., Mem. Cornell Univ. Agric. Exp. Sta. 6: 242. 1915.

*Fusarium culmorum* var. *majus* Wollenw., Fusaria Autogr. Delin. 2: 613. 1924.

*Lectotypus* (*hic designatus*, MBT 10000675): **UK**, infected ear of *Triticum* sp., 1884, W.G. Smith, in Diseases of field and garden crops, chiefly as are caused by fungi: 210. fig. 92.

*Epitypus* (*hic designatus*, MBT 10000676): **Denmark**, moldy kernel of *Hordeum vulgare*, 3 Feb. 1986, U. Thrane, CBS 417.86 (preserved as metabolic inactive culture).

*Ex-epitype culture*: CBS 417.86 = FRC R-8504 = IMI 309344= NRRL 25475.

*Descriptions and illustrations*: See Wollenweber & Reinking (1935), Booth (1971), Gerlach & Nirenberg (1982) and Leslie & Summerell (2006).

*Diagnostic DNA barcodes*: *rpb1*: JX171515; *rpb2*: JX171628; *tef1*: MW233082.

*Notes*: No holotype specimen could be located. Therefore, an illustration is designated as lectotype and CBS 417.86 is designated as epitype as this isolate is commonly used as an authentic strain for *F. culmorum* in literature (Ward *et al.* 2002, O'Donnell *et al.* 2013, 2020, Geiser *et al.* 2021).  

*cuneiforme Fusarium* Sherb., Mem. Cornell Univ. Agric. Exp. Sta. 6: 129. 1915.

(See *Fusarium ventricosum*)

*Typus*: ?CUP-007474.

*Type locality*: **USA**, New York.

*Type substrate*: *Solanum tuberosum*.

*Notes*: Synonym *fide*Wollenweber & Reinking (1935) and Booth (1971). Lectotypification pending study of material lodged in CUP.  

*cuneirostrum Fusarium* O'Donnell & T. Aoki, Mycoscience 46: 170. 2005.

(See *Fusarium azukiicola*)

*Holotypus*: BPI 843353.

*Ex-type culture*: FRC S-1551 = MAFF 239038 = NRRL 31157.

*Type locality*: **USA**, Michigan, Presque Isle.

*Type substrate*: *Phaseolus vulgaris*.

*Descriptions and illustrations*: See Aoki *et al.* (2005).

*Diagnostic DNA barcodes*: *rpb1*: KJ511271; *rpb2*: FJ240389; *tef1*: MAEA01003816.  

***curvatum Fusarium*** L. Lombard & Crous, Persoonia 43: 21. 2018 [2019].

*Holotypus*: CBS H-23611.

*Ex-type culture*: CBS 238.94 = NRRL 26422 = PD 94/184.

*Type locality*: **Netherlands**.

*Type substrate*: *Beaucarnea* sp.

*Descriptions and illustrations*: See Lombard *et al.* (2019b).

*Diagnostic DNA barcodes*: *rpb1*: MW928804; *rpb2*: MH484893; *tef1*: MH484984.  

*cuticola Fusarium* (R. Blanch.) Guég., Champ. Paras. Homme: 262. 1904.

(See ***Fusarium oxysporum***)

*Basionym*: *Selenosporium cuticola* R. Blanch., Compt. Rend. Hebd. Séances Acad. Sci. 111: 479. 1890.

*Holotypus*: Not located.

*Type locality*: **France**.

*Type substrate*: Skin of *Chamaeleo vulgaris* and *Lacerta viridis* (lizards)*.*

*Notes*: Synonym *fide*Wollenweber & Reinking (1935). Based on the substrate, this species could belong to the genus *Bisifusarium*. However, the protologue is not definitive, and recollection from type substrate is needed to confirm its taxonomic position.  

*cyanescens Fusarium* (G.A. de Vries *et al.*) O'Donnell *et al.*, Index Fungorum 440: 2. 2020.

***Neocosmospora cyanescens*** (G.A. de Vries *et al.*) Summerb. *et al.*, Biology of Microfungi (Cham): 183. 2016.

*Basionym*: *Phialophora cyanescens* G.A. de Vries *et al.*, Antonie van Leeuwenhoek 50: 150. 1984.

*Synonyms*: *Cylindrocarpon cyanescens* (G.A. de Vries *et al.*) Sigler, J. Clin. Microbiol. 29: 1858. 1991.

*Holotypus*: CBS 518.82 (maintained as metabolically inactive culture).

*Ex-type culture*: CBS 518.82.

*Type locality*: **Netherlands**, Groningen Province, Groningen.

*Type substrate*: Subcutaneous tissue of the right foot of a male *Homo sapiens*.

*Descriptions and illustrations*: See de Vries *et al.* (1984) and Zoutman & Sigler (1991).

*Diagnostic DNA barcodes*: *rpb1*: MW218110; *rpb2*: LR583826; *tef1*: LR583605.  

*cyanostomum Fusarium* (Sacc. & Flageolet) O'Donnell & Geiser, Phytopathology 103: 404. 2013.

***Cyanonectria cyanostoma*** (Sacc. & Flageolet) Samuels & P. Chaverri, Mycol. Progr. 8: 56. 2009.

*Basionym*: *Nectria cyanostoma* Sacc. & Flageolet, Rendiconti Congr. Bot. Palermo 1902: 53. 1902.

*Lectotypus*: BPI 551652, designated in Samuels *et al.* (2009).

*Epitypus*: BPI 748307, designated in Samuels *et al.* (2009).

*Ex-epitype culture*: BBA 70964 = CBS 101734 = G.J.S. 98-127.

*Epitype locality*: **France**.

*Epitype substrate*: *Buxus sempervirens*.

*Descriptions and illustrations*: See Samuels *et al.* (2009).

*Diagnostic DNA barcodes*: *rpb1*: JX171546; *rpb2*: HQ897759; *tef1*: HM626647.  

*cyclogenum Fusarium* Sacc., Nuovo Giorn. Bot. Ital. 8: 197. 1876.

?***Gloeosporium orbiculare*** (Berk.) Berk., Just's Bot. Jahresber. 4: 1274. 1876.

*Basionym*: *Cytospora orbicularis* Berk., Ann. Nat. Hist. 1: 207. 1838.

*Synonyms*: *Myxosporium orbiculare* (Berk.) Berk., Outl. Brit. Fungol.: 325. 1860.

*Colletotrichum orbiculare* (Berk.) Arx, Verh. Kon. Ned. Akad. Wetensch., Afd. Natuurk., Sect. 2, 51: 112. 1957, *nom. inval*., Art. 36.2 (Melbourne).

*Sirogloea orbicularis* (Berk.) Arx, Verh. Kon. Ned. Akad. Wetensch., Afd. Natuurk., Sect. 2, 51: 113. 1957, *nom. inval*., Art. 36.2 (Melbourne).

*Syntypes*: In BPI & S.

*Type locality*: **Italy**, Treviso, Selva.

*Type substrate*: *Citrullus* sp.

*Note*: *Cytospora orbicularis* is not a *Colletotrichum* nor a *Fusarium* (small ellipsoidal conidia discharged in tendrils) as outlined in Damm et al. (2013).  

*cydoniae Fusarium* Allesch., Ber. Bot. Vereines Landshut 12: 130. 1892.

(See ***Fusarium lateritium***)

*Holotypus*: In M.

*Type locality*: **Germany**, München.

*Type substrate*: *Cydonia vulgaris.*

*Note*: Synonym *fide*Wollenweber & Reinking (1935).  

*cydoniae Fusarium* Roum. & Fautrey, Rev. Mycol. (Toulouse) 14: 170. 1892, *nom. illegit.*, Art. 53.1, *non* Allescher 1892.

(See *Fusarium rollandianum*)  

*cydoniae Fusarium* (Schulzer) Sacc. & Traverso, Syll. Fung. 19: 724. 1910, *nom. illegit.*, Art. 53.1, *non* Allescher 1892, *nec* Roum. & Fautrey 1892.

*Basionym*: *Selenosporium cydoniae* Schulzer, Verhand. K.K. Zool.-Bot. Ges. Wien 21: 1240. 1871.

(See ***Fusarium lateritium***)

*Holotypus*: Not located.

*Type locality*: **Austria**, Vienna.

*Type substrate*: *Cydonia vulgaris*.

*Note*: Synonyms *fide*Wollenweber & Reinking (1935).  

*cylindricum Fusarium* (Mont.) Sacc., Syll. Fung. 4: 720. 1886.

*Basionym*: *Fusisporium cylindricum* Mont., Ann. Sci. Nat., Bot., sér. 2, 17: 120. 1842.

(See *Fusarium candidum* (Link) Sacc.)

*Holotypus*: ?PC.

*Type locality*: **Cuba**.

*Type substrate*: Sarcocarp of unknown fruit*.*

*Note*: Synonyms *fide*Wollenweber & Reinking (1935).  

*cymbiferum Fusarium* Berk. & M.A. Curtis, in Berkeley, Grevillea 3: 98. 1875.

***Colletotrichum coccodes*** (Wallr.) S. Hughes, Canad. J. Bot. 36: 754. 1958.

*Basionym*: *Chaetomium coccodes* Wallr., Fl. Crypt. Germ. 2: 265. 1833.

*Synonyms*: *Fusarium effusum* Schwein., Trans. Amer. Philos. Soc., n.s. 4: 302. 1832 [1834].

*Fusarium georginae* Corda, Icon. Fung. 2: 4. 1838.

*Vermicularia atramentaria* Berk. & Broome, Ann. Mag. Nat. Hist. 5: 378. 1850.

*Colletotrichum atramentarium* (Berk. & Broome) Taubenh., Mem. New York Bot. Gard. 6: 554. 1916.

*Acrothecium solani* Sacc., Michelia 1(3): 74. 1877.

*Fusisporium elasticae* Thüm., Boll. Soc. Adriat. Sci. Nat. Trieste 3: 440. 1877.

*Fusarium elasticae* (Thüm.) Sacc., Syll. Fung. 4: 711. 1886.

*Gloeosporium elasticae* Cooke & Massee, in Cooke, Grevillea 18: 74. 1890.

*Fusarium foliicola* Allesch., Hedwigia 34: 289. 1895.

*Gloeosporium foliicola* (Allesch.) Wollenw., *Fusarien*: 325. 1935, *nom. illegit.*, Art. 53.1.

*Colletotrichum solanicola* O'Gara, Mycologia 7: 39. 1915.

*Colletotrichum biologicum* Chaudhuri, Ann. Bot. 38: 735. 1924.

*Holotypus*: ?K(M).

*Type locality*: **USA**.

*Type substrate*: Stems of some herbaceous plants*.*

*Note*: Synonyms *fide*Wollenweber & Reinking (1935).  

*cypericola Fusarium* Henn., Hedwigia 48: 116. 1908.

***Libertella cypericola*** (Henn.) Wollenw., Fusaria Autogr. Delin. 1: 486. 1916.

*Syntype*: In B *fide*Hein (1988).

*Type locality*: **Brazil**, Pará.

*Type substrate*: *Cyperus exaltatus.*

*Note*: Synonym *fide*Wollenweber & Reinking (1935).  

***dactylidis Fusarium*** T. Aoki *et al.*, Mycologia 107: 412. 2015.

*Holotypus*: BPI 892886.

*Ex-type culture*: CBS 119181 = ICMP 5269 = NRRL 29298.

*Type locality*: **New Zealand**, Manawatu, Palmerston North.

*Type substrate*: *Dactylis glomerata.*

*Descriptions and illustrations*: See Aoki *et al.* (2015).

*Diagnostic DNA barcodes*: *rpb1*: KM361654; *rpb2*: KM361672; *tef1*: DQ459748.  

*decemcellulare Fusarium* Brick, Jahresber. Vereinigung Angew. Bot. 6: 227. 1908.

(See *Fusarium colorans*)

*Holotypus*: ?HBG.

*Type locality*: **Cameroon**.

*Type substrate*: *Theobroma cacao.*  

*decipiens Fusarium* Cooke & Massee, in Cooke, Handb. Austral. Fungi: 388. 1892, *nom. inval*., Art. 39.1.

(See *Fusarium candidum* (Link) Sacc.)

*Authentic material*: ?K(M).

*Original locality*: **Australia**, Queensland.

*Original substrate*: *Ficus aspera.*

*Note*: Synonym *fide*Wollenweber & Reinking (1935).  

*deformans Fusarium* J. Schröt., Jahresber. Schles. Ges. Vaterl. Cult. 61: 179. 1883.

***Gloeosporium deformans*** (J. Schröt.) Lind, Ann. Bot. 7: 19. 1908.

*Synonyms*: *Fusamen deformans* (J. Schröt.) P. Karst., Bidrag Kännedom Finlands Natur Folk 51: 485. 1892.

*Calogloeum deformans* (J. Schröt.) Nannf., Svensk Bot. Tidskr. 25: 25. 1931.

*Platycarpium deformans* (J. Schröt.) Petr., Sydowia 7: 296. 1953.

*Holotypus*: In B *fide*Wollenweber (1916–1935).

*Type locality*: **Poland**, Breslau.

*Type substrate*: *Salix cinerea.*

*Note*: Synonyms *fide*Wollenweber & Reinking (1935).  

*delacroixii Fusarium* Sacc., Syll. Fung. 10: 725. 1892.

(See ***Fusarium sambucinum***)

*Replaced synonym*: *Fusarium asparagi* Delacr., Bull. Soc. Mycol. France 6: 99. 1890, *nom. illegit.*, Art. 53.1, *non Fusarium asparagi* Briard 1890.

*Lectotypus* (*hic designatus*, MBT 10000677): **France**, Paris, *Asparagus officinalis*, 1890, M.G. Delaroix, in Bull. Soc. Mycol. France 6, pl. XV. fig. III.

*Notes*: Synonyms *fide*Wollenweber & Reinking (1935). No holotype material is available for the replaced synonym *F. asparagi* Delacr. and therefore, an illustration from the original protologue is designated as lectotype.  

*delphinoides Fusarium* Schroers *et al.*, Mycologia 101: 57. 2009.

***Bisifusarium delphinoides*** (Schroers *et al.*) L. Lombard & Crous, Stud. Mycol. 80: 224. 2015.

*Holotypus*: CBS H-20124.

*Ex-type culture*: CBS 120718 = NRRL 53290.

*Type locality*: **South Africa**, Western Cape Province, Clanwilliam.

*Type substrate*: *Hoodia gordonii* stem lesions*.*

*Descriptions and illustrations*: See Schroers *et al.* (2009).

*Diagnostic DNA barcodes*: *rpb1*: KM232210; *tef1*: EU926296.  

***denticulatum Fusarium*** Nirenberg & O'Donnell, Mycologia 90: 445. 1998.

*Holotypus*: B 70 0001691.

*Ex-type culture*: BBA 67772 = CBS 407.97 = IMI 376115 = NRRL 25311.

*Type locality*: **USA**, Louisiana.

*Type substrate*: *Ipomoea batatas.*

*Descriptions and illustrations*: See Nirenberg & O'Donnell (1998) and Leslie & Summerell (2006).

*Diagnostic DNA barcodes*: *rpb1*: MT010953; *rpb2*: MT010970; *tef1*: KR909385.  

*derridis Fusarium* Henn., Beibl. Hedwigia 41: (66). 1902.

(See *Fusarium coccidicola*)

*Holotypus*: In B *fide*Hein (1988).

*Type locality*: **Papua New Guinea**.

*Type substrate*: *Derris* sp.

*Note*: Synonym *fide*Wollenweber & Reinking (1935).  

*desaboruense Fusarium* N. Maryani *et al.*, Persoonia 43: 59. 2019.

(See ***Fusarium sacchari***)

*Holotypus*: InaCC F951 (preserved as metabolically inactive culture).

*Ex-type culture*: InaCC F951.

*Type locality*: **Indonesia**, East Nusa Tenggara, Sikka Flores, Kecamatan Waigate, Desa Boru.

*Type substrate*: *Musa* var. Pisang Kepok*.*

*Descriptions and illustrations*: See Maryani *et al.* (2019b).

*Diagnostic DNA barcodes*: *rpb1*: LS479870; *rpb2*: LS479852.  

*desciscens Fusarium* Oudem., Ned. Kruidk. Arch., 2 sér., 5: 515. 1889.

(See ***Fusarium sarcochroum***)

*Holotypus*: ?L.

*Type locality*: **Netherlands**, Zuid-Holland Province, Den Haag, Scheveningen.

*Type substrate*: *Sarothamnus vulgaris*.

*Note*: Synonym *fide*Wollenweber & Reinking (1935).  

*detonianum Fusarium* Sacc. (as ‘*de-tonianum*’), Syll. Fung. 4: 708. 1886, *nom. illegit.*, Art. 52.1.

(See *Fusarium miniatum* Sacc.)

*Authentic material*: Not located.

*Original locality*: **Italy**.

*Original substrate*: Sporangium of *Cyathus vernicosa*.  

*dianthi Fusarium* Prill. & Delacr., Compt. Rend. Hebd. Séances Acad. Sci. 129: 745. 1899.

(See ***Fusarium oxysporum***)

*Holotypus*: Not located.

*Type locality*: **France**, Antibes.

*Type substrate*: *Dianthus caryophyllus*.  

*didymum Fusarium* (Harting) Lindau, Rabenh. Krypt.-Fl. Ed. 2, 1(9): 574. 1909.

*Basionym*: *Fusisporium didymum* Harting, Nieuwe Verh. Eerste Kl. Kon. Ned. Inst. Wetensch. Amsterdam 12: 228. 1846.

(See *Fusarium eichleri*)

*Lectotypus* (*hic designatus*, MBT 10000678): **Netherlands**, *Solanum tuberosum*, date unknown, Harting, in Nieuwe Verh. Eerste Kl. Kon. Ned. Inst. Wetensch. Amsterdam 12 (1846), tab. II, figs 2–4.

*Notes*: Requires recombination into *Neonectria* after further investigation. No preserved specimen could be located and therefore an illustration is designated as lectotype.  

*diffusum Fusarium* Carmich., Grevillea 16: 81. 1888.

(See ***Fusarium avenaceum***)

*Holotypus*: ?K(M).

*Type locality*: **UK**, Scotland, Appin.

*Type substrate*: Stems of *Asteraceae* (thristle).

*Note*: Synonym *fide*Wollenweber & Reinking (1935).  

*dimerum Fusarium* Penz., Michelia 2: 484. 1882.

***Bisifusarium dimerum*** (Penz.) L. Lombard & Crous, Stud. Mycol. 80: 225. 2015.

*Synonyms*: *Fusarium aquaeductuum* var. *dimerum* (Penz.) Raillo, Fungi of the Genus Fusarium: 279. 1950.

*Microdochium dimerum* (Penz.) Arx, Trans. Brit. Mycol. Soc. 83: 374. 1984.

?*Fusisporium flavum* Fr., Syst. Mycol. 3: 444. 1832.

?*Pionnotes flava* (Fr.) Sacc., Syll. Fung. 4: 726. 1886.

?*Fusarium flavum* (Fr.) Wollenw., Z. Parasitenk. 3: 305. 1931.

?*Fusarium aquaeductuum* var. *flavum* (Fr.) Raillo, Fungi of the Genus Fusarium: 280. 1950.

*Selenosporium aurantiacum* Bonord., Abh. Naturf. Ges. Halle 8: 97. 1864, *nom. illegit.*, Art. 53.1.

*Fusarium bonordenii* Sacc., Syll. Fung. 4: 699. 1886.

*Fusarium baptisiae* Henn., Notizbl. Bot. Gart. Berlin 2: 383. 1899.

*Fusarium subnivale* Höhn., in Penther & Zederbauer, Ann. K.K. Naturhist. Hofmus. 20: 369. 1905.

*Fusarium dimerum* var. *majusculum* Wollenw., Fusaria Autogr. Delin. 1: 90. 1916.

?*Fusarium pusillum* Wollenw., Fusaria Autogr. Delin. 2: 550. 1924.

?*Fusarium dimerum* var. *pusillum* (Wollenw.) Wollenw., Fusaria Autogr. Delin. 3: 851. 1930.

*Fusarium dimerum* var. *violaceum* Wollenw., Fusaria Autogr. Delin. 3: 854. 1930.

*Lectotypus*: Fig. 1212 in Penzig (1882), designated in Schroers *et al.* (2009).

*Epitypus*: CBS H-20129, designated in Schroers *et al.* (2009).

*Ex-epitype culture*: CBS 108944 = NRRL 36140.

*Epitype locality*: **Netherlands**.

*Epitype substrate*: Blood of *Homo sapiens* with acute myeloid leukemia.

*Descriptions and illustrations*: See Schroers *et al.* (2009).

*Diagnostic DNA barcodes*: *rpb1*: KM232212; *rpb2*: KM232363; *tef1*: EU926334.

*Note*: Synonyms *fide*Wollenweber & Reinking (1935) and Booth (1971).  

*diminutum Fusarium* (Sand.-Den. & Crous) O'Donnell *et al.*, Index Fungorum 440: 2. 2020.

***Neocosmospora diminuta*** Sand.-Den. & Crous, Persoonia 43: 127. 2019.

*Holotypus*: CBS H-23979.

*Ex-type culture*: CBS 144390 = MUCL 18798.

*Type locality*: ?**Ivory Coast**.

*Type substrate*: Treated wood of *Coelocaryon preussii.*

*Descriptions and illustrations*: See Sandoval-Denis *et al.* (2019).

*Diagnostic DNA barcodes*: *rpb1*: MW834218; *rpb2*: LR583828; *tef1*: LR583607.  

*dimorphum Fusarium* J.V. Almeida & Sousa da Câmara, Revista Agron. (Lisbon) 1: 306. 1903.

(See *Fusarium buxicola*)

*Holotypus*: MA-Funhist:6036-1.

*Type locality*: **Portugal**.

*Type substrate*: *Buxus sempervirens*.

*Note*: Synonym *fide*Wollenweber & Reinking (1935).  

*diplosporum Fusarium* Cooke & Ellis, Grevillea 7: 38. 1878.

(See ***Fusarium sarcochroum***)

*Holotypus*: ?K(M).

*Type locality*: **USA**, New Jersey.

*Type substrate*: Stems of *Solanum tuberosum*.

*Note*: Synonym *fide*Wollenweber & Reinking (1935).  

*discoideum Fusarium* Fautrey & Roum., Rev. Mycol. (Toulouse) 13: 173. 1891.

(See ***Fusarium lateritium***)

*Syntype*: ILL00220061 (Roumeguère, Fungi Sel. Gall. Exs. no. 5898).

*Type locality*: **France**, Noidan.

*Type substrate*: *Sambucus nigra.*

*Note*: Synonym *fide*Wollenweber & Reinking (1935).  

*discolor Fusarium* Appel & Wollenw., Arbeiten Kaiserl. Biol. Anst. Land-Forstw. 8: 114. 1913.

(See ***Fusarium sambucinum***)

*Holotypus*: ?S-F45617.

*Type locality*: **Germany**, Berlin.

*Type substrate*: *Solanum tuberosum*.

*Note*: Synonym *fide*Wollenweber & Reinking (1935).  

***diversisporum Fusarium*** Sherb., Mem. Cornell Univ. Agric. Exp. Sta. 6: 161. 1915.

*Typus*: ?CUP-007430.

*Type locality*: **USA**, New York.

*Type substrate*: *Solanum tuberosum*

*Descriptions and illustrations*: See Sherbakoff (1915) and Gerlach & Nirenberg (1982).

*Notes*: This species is recognised by Gerlach & Nirenberg (1982) who considered isolate CBS 795.70 as authentic for *F. diversisporum*. However, typification of *F. diversisporum* first requires study of the specimen lodged in CUP.  

***dlaminii Fusarium*** Marasas *et al.*, Mycologia 77: 971. 1986 [1985].

*Holotypus*: DAOM 191112.

*Ex-type culture*: ATCC 58097 = BBA 69859 = CBS 175.88 = DAOM 191112 = FRC M-1637 = IMI 290241 = MRC 3032 = NRRL 13164.

*Type locality*: **South Africa**, Eastern Cape Province, Butterworth.

*Type substrate*: Plant debris in soil*.*

*Descriptions and illustrations*: See Marasas *et al.* (1985) and Leslie & Summerell (2006).

*Diagnostic DNA barcodes*: *rpb1*: KU171681; *rpb2*: KU171701; *tef1*: KU171721.  

*domesticum Fusarium* (Fr.) H.P. Bachm., LWT – Food Sci. Technol. 38: 405. 2005, *nom. inval*., Art. 41.5, See Art. 41.7.

***Bisifusarium domesticum*** (Fr.) L. Lombard & Crous, Stud. Mycol. 80: 225. 2015.

*Basionym*: *Trichothecium domesticum* Fr., Syst. Mycol. 3: 427. 1832.

*Neotypus*: CBS 434.34 (preserved as metabolically inactive culture), designated in Bachmann *et al.* (2005).

*Ex-neotype culture*: ATCC 13417 = CBS 434.34 = MUCL 9826.

*Type locality*: **Belgium**.

*Type substrate*: Cheese*.*

*Descriptions and illustrations*: See Schroers *et al.* (2009).  

***dominicanum Fusarium*** Cif., Sydowia 9: 325. 1955

*Holotypus*: ?PAV.

*Type locality*: **Dominican Republic**, Santo Domingo, Villa Altagracia.

*Type substrate*: *Byrsonima* sp. (between mycelium of *Meliola byrsonimae*)*.*

*Descriptions and illustrations*: See Ciferri (1955).

*Notes*: Ciferri (1955) considered this a ‘conventional’ species as the author indicated that more information based on culture characteristics is required. No living material of this species could be located and recollection from the type locality is required.  

***duofalcatisporum Fusarium*** J.W. Xia *et al.*, Persoonia 43: 201. 2019.

*Holotypus*: CBS H-24056.

*Ex-type culture*: CBS 384.92 = NRRL 36448.

*Type locality*: **Sudan**, Nile Province.

*Type substrate*: Seeds of *Phaseolus vulgaris.*

*Descriptions and illustrations*: See Xia *et al.* (2019).

*Diagnostic DNA barcodes*: *rpb2*: GQ505830; *tef1*: GQ505652.  

***duoseptatum Fusarium*** Maryani *et al.*, Stud. Mycol. 92: 181. 2018 [2019].

*Holotypus*: InaCC F916 (preserved as metabolically inactive culture).

*Ex-type culture*: InaCC F916.

*Type locality*: **Indonesia**, Central Kalimantan, Kapuas Timur, Anjir Serapat Tengah.

*Type substrate*: Pseudostem of *Musa* var. Pisang Kepok*.*

*Descriptions and illustrations*: See Maryani *et al.* (2019a).

*Diagnostic DNA barcodes*: *rpb1*: LS479495; *rpb2*: LS479239; *tef1*: LS479688.  

***echinatum Fusarium*** Sand.-Den. & G.J. Marais, Stud. Mycol. 98 (no. 100116): 47. 2021.

*Holotypus*: CBS H-24658.

*Ex-type culture*: CAMS 000733 = CBS 146497 = CPC 30815.

*Type locality*: **South Africa**.

*Type substrate*: Unidentified tree.

*Descriptions and illustrations*: See this study.

*Diagnostic DNA barcodes*: *rpb1:* MW834187; *rpb2:* MW834004; *tef1:* MW834273.  

***echinosporum Fusarium*** Sibilia, Ann. Reale. Ist. Super. Agrar. Forest., ser. 2, 1: 77. 1925.

*Holotypus*: Not located.

*Type locality*: **Italy**.

*Type substrate*: *Cedrus deodara.*

*Descriptions and illustrations*: See Sibilia (1925).

*Notes*: This species is recognised in Petrak's Lists V. 3. Wollenweber & Reinking (1935) mention this species, but they did not treat it any further. Booth (1971) considered it a possible synonym of *F. graminearum*. Requires recollection from the type locality and substrate.  

*effusum Fusarium* Schwein., Trans. Amer. Philos. Soc., n.s., 4: 302. 1832 [1834].

(See *Fusarium cymbiferum*)

*Holotypus*: PH00062491.

*Type locality*: **USA**, Pennsylvania, Northampton, Bethlehem.

*Type substrate*: *Hypericum frondosum*.

*Note*: Synonym *fide*Wollenweber & Reinking (1935).  

*eichleri Fusarium* Bres., Ann. Mycol. 1: 130. 1903.

(See *Fusarium candidum* Ehrenb.)

*Holotypus*: S-F45618.

*Type locality*: **Poland**.

*Type substrate*: *Salix caprea.*  

***elaeidis Fusarium*** L. Lombard & Crous, Persoonia 43: 23. 2018 [2019].

*Holotypus*: CBS H-23612.

*Ex-type culture*: CBS 217.49 = NRRL 36358.

*Type locality*: **Zaire**.

*Type substrate*: *Elaeis* sp*.*

*Descriptions and illustrations*: See Lombard *et al.* (2019b).

*Diagnostic DNA barcodes*: *rpb1*: MW928805; *rpb2*: MH484870; *tef1*: MH484961.  

*elasticae Fusarium* (Thüm.) Sacc., Syll. Fung. 4: 711. 1886.

*Basionym*: *Fusisporium elasticae* Thüm., in Bolle & Thümen, Boll. Soc. Adriat. Sci. Nat. Trieste 3: 440. 1877.

(See *Fusarium cymbiferum*)

*Lectotypus* (*hic designatus*, MBT 10000679): **Italy**, Gorizia, *Ficus elastica*, 1877, F. de Thümen, in Bolle & Thümen, Boll. Soc. Adriat. Sci. Nat. Trieste 3, tab. I, fig. 13.

*Notes*: Synonyms *fide*Wollenweber & Reinking (1935). No holotype specimen could be located and therefore an illustration is designated as lectotype.  

*elegans Fusarium* Appel & Wollenw., Arbeiten Kaiserl. Biol. Anst. Land-Forstw. 8: 94. 1913, *nom. inval*., Art. 36.1(a).

(See ***Fusarium oxysporum***)

*Notes*: Appel and Wollenweber (*l.c*.) proposed this name only provisionally under *Fusarium solani*. They added an illustration of conidia on page 38 (fig. 2D).  

*elegans Fusarium* W. Yamam. & Maeda, Trans. Mycol. Soc. Japan 3: 115. 1962.

***Neocosmospora elegans*** (W. Yamam. & Maeda) Sand.-Den. & Crous, Persoonia 43: 127. 2019.

*Basionym*: *Nectria elegans* W. Yamam. & Maeda, Hyogo Univ. Agric. ser. Agric. Biol. 3: 15. 1957.

*Synonyms*: ?*Fusarium solani f*. *xanthoxyli* Y. Sakurai & Matuo, Ann. Phytopathol. Soc. Japan 26: 117. 1961, *nom. inval*., Art. 39.1.

?*Hypomyces solani f. xanthoxyli* Y. Sakurai & Matuo, Ann. Phytopathol. Soc. Japan 26: 117. 1961, *nom. inval*., Art. 39.1.

*Fusarium yamamotoi* O'Donnell *et al.*, Index Fungorum 440: 5. 2020.

*Lectotypus*: figs 1–9, p. 16, in Yamamoto *et al.* (1957), designated in Sandoval-Denis *et al.* (2019).

*Epitypus*: CBS H-23980, designated in Sandoval-Denis *et al.* (2019).

*Ex-epitype culture*: ATCC 42366 = CBS 144396 = MAFF 238541 = NRRL 22277 = SUF XV-1.

*Epitype locality*: **Japan**, Hyōgo.

*Epitype substrate*: Trunk of *Zanthoxylum piperitum*

*Diagnostic DNA barcodes*: *rpb1*: MW218113; *rpb2*: FJ240380; *tef1*: AF178336

*Note*: This is a valid species name that is not a homonym since the name *F. elegans* Appel & Wollenw. is an invalid name.  

*eleocharidis Fusarium* Rostr. (as ‘*heleocharidis’*), in Thümen, Mycoth. Univ., Cent. 22: no. 2185. 1883.

(See ***Fusarium heterosporum***)

*Syntypes*: In BPI, NEB & S (Mycoth. Univ., Cent. 22: no. 2185).

*Type locality*: **Denmark**, Fyn, Langeland.

*Type substrate*: *Eleocharis palustris.*

*Notes*: Synonym *fide*Wollenweber & Reinking (1935).  

*elongatum Fusarium* Cooke, Grevillea 19: 4. 1890.

(See *Fusarium ciliatum*)

*Holotypus*: In K(M), Colenso 538 *fide* Index Fungorum .

*Type locality*: **New Zealand**.

*Type substrate*: Twigs*.*

*Note*: Synonym *fide*Wollenweber & Reinking (1935).  

*elongatum Fusarium* De Wild., Ann. Soc. Belge Microsc*.* 17: 42. 1893, *nom illegit.*, Art. 53.1.

*Replacing synonym*: *Fusarium longissimum* Sacc. & P. Syd., Syll. Fung. 14: 1128. 1899.

***Amniculicola longissima*** (Sacc. & P. Syd.) Nadeeshan & K.D. Hyde, IMA Fungus 7: 301. 2016.

*Synonyms*: *Anguillospora longissima* (Sacc. & P. Syd.) Ingold, Trans. Brit. Mycol. Soc. 25: 402. 1942.

*Holotypus*: Not located.

*Type locality*: **Belgium**, Brussels, Botanical Garden.

*Type substrate*: Submerged plant material.

*Note*: Synonyms *fide*Rossman *et al.* (2016).  

*elongatum Fusarium* O.A. Pratt, J. Agric. Res. 13: 84. 1918, *nom. illegit.*, Art. 53.1.

(See ***Fusarium sambucinum***)

*Authentic material*: Not located.

*Original locality*: **USA**, Idaho.

*Original substrate*: Soil.

*Note*: Synonym *fide*Wollenweber & Reinking (1935).  

*elongatum Fusarium* Reinking, Zentralbl. Bakteriol. Parasitenk., Abt. 2, 89: 511. 1934, *nom. illegit.*, Art. 53.1.

(See ***Fusarium sublunatum***)

*Authentic material*: B 70 0100189.

*Original culture*: CBS 190.34 = NRRL 20897.

*Original locality*: **Costa Rica**.

*Original substrate*: Soil from *Musa sapientum* and *Theobroma cacao* plantation.

*Diagnostic DNA barcodes*: *rpb1*: KX302927; *rpb2*: KX302935; *tef1*: KX302919.

*Note*: Synonym *fide*Wollenweber & Reinking (1935).  

*ensiforme Fusarium* Wollenw. & Reinking, Phytopathology 15: 169. 1925.

*Synonym*: *Fusarium javanicum* var. *ensiforme* (Wollenw. & Reinking) Wollenw., Z. Parasitenk. 3: 483. 1931.

*Fusarium javanicum* subsp. *ensiforme* (Wollenw. & Reinking) Raillo, Fungi of the Genus Fusarium: 229. 1950.

*Holotypus*: Not located.

*Type locality*: **Honduras**.

*Type substrate*: Rotten fruit of *Ficus* sp*.*

*Notes*: Synonyms *fide*Wollenweber & Reinking (1935). Synonym of *F. javanicum fide*Gerlach & Nirenberg (1982). Status unclear [see Sandoval-Denis *et al.* (2019)].  

*entomophilum Fusarium* Petch, Trans. Brit. Mycol. Soc. 11: 260. 1926.

(See ***Fusarium lateritium***)

*Holotypus*: ?K(M).

*Type locality*: **Sri Lanka**, Suduganga.

*Type substrate*: *Clitellaria heminopla.*

*Note*: Synonym *fide*Wollenweber & Reinking (1935).  

*epicoccum Fusarium* McAlpine, Fungus Diseases of Citrus trees in Australia: 113. 1899.

(See *Fusarium larvarum*)

*Lectotypus* (*hic designatus*, MBT 10000680): **Australia**, Victoria, Melbourne, *Aspidiotus aurantium* on *Citrus deliciosa*, 1899, D. McAlpine, in Fungus Diseases of Citrus trees in Australia, figs 177–180.

*Note*: Synonym *fide*Wollenweber & Reinking (1935). No holotype specimen could be located and therefore an illustration is designated as lectotype.  

*epimyces Fusarium* Cooke, Grevillea 17: 15. 1888, *nom. inval*., Art. 38.1(a).

(See *Fusarium azukiicola*)

*Authentic material*: In K(M) *fide* Index Fungorum.

*Original locality*: **UK**, Reading.

*Original substrate*: *Scleroderma* sp*.*

*Note*: Synonym *fide*Wollenweber & Reinking (1935).  

*episphaeria Fusarium* (Tode) W.C. Snyder & H.N. Hansen, Amer. J. Bot. 32: 662. 1945.

***Dialonectria episphaeria*** (Tode) Cooke (as ‘*episphærica*’), Grevillea 12: 82. 1884.

*Basionym*: *Sphaeria episphaeria* Tode, Fung. Mecklenb. Sel. 2: 21. 1791.

*Synonyms*: *Nectria episphaeria* (Tode) Fr., Summa Veg. Scand. 2: 388. 1849.

*Cucurbitaria episphaeria* (Tode) Kuntze, Revis. Gen. Pl. 3: 461. 1898.

*Cosmospora episphaeria* (Tode) Rossman & Samuels, Stud. Mycol. 42: 121. 1999.

*Hypoxylon phoeniceum* Bull., Hist. Champ. France 1: 171. 1791.

*Sphaeria sanguinea* var. *media* Fr., Syst. Mycol. 2: 453. 1823.

*Nectria episphaeria* var. *media* (Fr.) Sacc., Syll. Fung. 2: 497. 1883.

*Dialonectria episphaeria* var. *verruculosa* Cooke, Grevillea 12: 82. 1884.

*Nectria episphaeria* var. *verruculosa* (Cooke) Berl. & Voglino, Syll. Fung., Addit. Vol. 1–4: 203. 1886.

*Nectria episphaeria* var. *kretzschmariae* Henn., Bot. Jahrb. Syst. 14: 364. 1891.

*Nectria episphaeria* var. *gregaria* Starbäck, Ark. Bot. 5: 9. 1905.

*Lectotypus*: L 0112704 (Herb. Lugd. Bat. 910267659 ex Herb. Persoon), selected in Booth (1959).

*Type locality*: **Unknown**.

*Type substrate*: Partially decorticated twig of *Diatrype stigma.*  

*episphaericum Fusarium* (Cooke & Ellis) Sacc., Syll. Fung. 4: 708. 1886.

*Basionym*: *Fusisporium episphaericum* Cooke & Ellis, Grevillea 5: 50. 1876.

***Cosmospora nothepisphaeria*** (Samuels) Rossman & Samuels, Stud. Mycol. 42: 123. 1999.

*Basionym*: *Nectria nothepisphaeria* Samuels, Mycol. Pap. 164: 30. 1991.

*Synonyms*: *Fusarium ciliatum* var. *episphaericum* (Cooke & Ellis) Wollenw., Fusaria Autogr. Delin. 3: 871. 1930.

*Fusarium ciliatum* var. *majus* Wollenw., Fusaria Autogr. Delin. 3: 872. 1930.

*Lectotypus* (of *Fusisporium episphaericum, hic designatus*, MBT 10000681): **USA**, New Jersey, parasitic on *Diatrypella* sp. on *Corylus avellana*, 1876, M.C. Cooke & J.B. Ellis, in Grevillea 5, pl. 80, fig. 10.

*Note*: No holotype specimen could be located and therefore an illustration is designated as lectotype.  

*epistroma Fusarium* (Höhn.) C. Booth (as ‘*epistromum*’), The Genus Fusarium: 66. 1971.

***Fusicolla epistroma*** (Höhn.) Gräfenhan & Seifert, Stud. Mycol. 68: 100. 2011.

*Basionym*: *Dendrodochium epistroma* Höhn., Sitzungsber. Kaiserl. Akad. Wiss. Wien. Math.-Naturwiss. Cl., Abt. 1., 118: 424. 1909.

*Lectotypus*: B 700014042, designated in Gräfenhan *et al.* (2011).

*Lectotype locality*: **Germany**, Brandenburg, “Schmidt's Grund” near Tamsel.

*Lectotype substrate*: Old stromata of *Diatrypella favacea*.

*Epitypus*: IMI 85601, designated in Gräfenhan *et al.* (2011).

*Ex-epitype culture*: ATCC 24369 = BBA 62201 = NRRL 20439 = NRRL 20461.

*Epitype locality*: **UK**, Yorkshire.

*Epitype substrate*: *Diatrypella* on *Betula.*

*Diagnostic DNA barcode*: *rpb2*: HQ897765.  

*epithele Fusarium* McAlpine, Fungus Diseases of Citrus trees in Australia: 80. 1899.

(See ***Fusarium reticulatum***)

*Holotypus*: VPRI 2563.

*Type locality*: **Australia**, New South Wales.

*Type substrate*: Rotten fruit of *Citrus* x *limon.*

*Note*: Synonym *fide*Wollenweber & Reinking (1935).  

*equinum Fusarium* Növgaard, Science, N.Y. 14: 899. 1901.

*Holotypus*: Not located.

*Type locality*: **USA**.

*Type substrate*: Infected skin of *Equus* sp. (horse)*.*

*Notes*: Status unclear. Doubtful species *fide*Wollenweber & Reinking (1935). Based on the original substrate, this species might belong to the medically important genus *Neocosmospora*. However, recollection is required to confirm its taxonomic affiliation.  

***equiseti Fusarium*** (Corda) Sacc., Syll. Fung. 4: 707. 1886.

*Basionym*: *Selenosporium equiseti* Corda, Icon. Fung. 2: 7. 1838.

*Synonyms*: *Fusisporium ossicola* Berk. & M.A. Curtis, Grevillea 3: 147. 1875.

*Fusarium ossicola* (Berk. & M.A. Curtis) Sacc., Syll. Fung. 4: 714. 1886.

*Fusarium nectriae-palmicolae* Henn., Bot. Jahrb. Syst. 23: 290. 1896.

*Fusarium gibbosum* Appel & Wollenw., Arbeiten Kaiserl. Biol. Anst. Land-Forstw. 8: 190. 1910.

*Fusarium roseum* var. *gibbosum* (Appel & Wollenw.) Messiaen & R. Cass., Ann. Inst. Natl. Rech. Agron. Tunisie 19: 435. 1968, *nom. inval*., Art. 41.5.

*Fusarium roseum* var. *gibbosum* (Appel & Wollenw.) Messiaen & R. Cass., Agronomie 8: 220. 1988, *nom. inval*., Art. 41.1.

*Fusarium bullatum* Sherb., Mem. Cornell Univ. Agric. Exp. Sta. 6: 198. 1915.

*Fusarium equiseti* var. *bullatum* (Sherb.) Wollenw., Fusaria Autogr. Delin. 3: 916. 1930.

*Fusarium gibbosum* var. *bullatum* (Sherb.) Bilaĭ, Mikrobiol. Zhurn. 49: 6. 1987.

*Fusarium bullatum* var. *roseum* Sherb., Mem. Cornell Univ. Agric. Exp. Sta. 6: 201. 1915.

*Fusarium roseobullatum* Wollenw. (as ‘*roseo-bullatum'*’), Fusaria Autogr. Delin. 1: 117. 1916.

*Fusarium vasinfectum* var. *pisi* Schikora, Arbeiten. Biol. Anst. Land-Forstwirt. 5: 188, pl. 7. 1906, *nom. illegit.*, Art. 53.1.

*Fusarium falcatum* Appel & Wollenw., Arbeiten Kaiserl. Biol. Anst. Land-Forstw. 8: 184. 1910.

*Fusarium falcatum* var. *fuscum* Sherb., Mem. Cornell Univ. Agric. Exp. Sta. 6: 138. 1915.

*Fusarium equiseti* var. *crassum* Wollenw., Fusaria Autogr. Delin. 3: 921. 1930.

*Fusarium terrestre* Manns, Bull. North Dakota Agric. Exp. Sta.: no. 259. 1932.

*Gibberella intricans* Wollenw., Fusaria Autogr. Delin. 3: 810. 1930.

*Fusarium eucheliae* Sartory, R. Sartory & J. Mey., Ann. Mycol. 30: 471. 1932.

*Fusarium equiseti* var*. intermedium* Saccas, Agron. Trop. (Maracay) 10: 49. 1955, *nom. inval*., Art. 39.1.

*Lectotypus*: (*hic designatus*, MBT 10001325): **Czech Republic**, Kuchelbad, near Prague, on stems of *Equisetum* sp., 1836, AKJ. Corda. Icon. Fung. 2, tab. IX, fig. 32.

*Epitypus* (*hic designatus*, MBT 10000682): **Germany**, Braunschweig, Niedersachsen, soil, 3 Aug. 1994, H. I. Nirenberg, CBS H-5570.

*Ex-epitype culture*: BBA 68556 = CBS 307.94 = NRRL 26419.

*Descriptions and illustrations*: See Wollenweber & Reinking (1935), Booth (1971), Gerlach & Nirenberg (1982), Holubová-Jechová *et al.* (1994) and Leslie & Summerell (2006).

*Diagnostic DNA barcodes*: *rpb2*: GQ505777; *tef1*: GQ505599.

*Notes*: Holubová-Jechová *et al.* (1994) incorrectly designated CBS 307.94 (CBS H-5570) as neotype for *Selenosporium equiseti* even though original material was available in PRM as well as an illustration provided in the protologue. A lectotypification rather than a neotypification was required. Therefore, the original illustration is selected as lectotype and CBS H-5570 (= CBS 307.94) is designated as epitype here, superseding the neotype designation.  

*equiseticola Fusarium* Allesch., Hedwigia 34: 289. 1895.

(See ***Fusarium scirpi***)

*Holotypus*: In M.

*Type locality*: **Germany**, Oberammergau.

*Type substrate*: Dried stems of *Equisetum limosum.*

*Note*: Synonym *fide*Wollenweber & Reinking (1935).  

*equisetorum Fusarium* Desm., Pl. Crypt. N. France: no. 1546/1846? 1843.

*Basionym*: *Hymenula equiseti* Lib., Pl. Crypt. Arduenna 3: no. 236. 1834.

(See ***Fusarium oxysporum***)

*Syntypes*: In BPI, BRU, CUP, ISC PH, S & UPS (Pl. Crypt. Arduenna 3: no. 236).

*Type locality*: **Belgium**.

*Type substrate*: *Equisetum limosum*.

*Notes*: Synonym *fide*Wollenweber & Reinking (1935).  

*ershadii Fusarium* M. Papizadeh *et al.*, Europ. J. Pl. Pathol. 151: 693. 2018, *nom. illegit.*, Art. 52.1.

*Basionym*: *Cylindrocarpon tonkinense* Bugnic., Encycl. Mycol. 11: 181. 1939.

(See *Fusarium tonkinense*)  

*erubescens Fusarium* Berk. & M.A. Curtis, Grevillea 3: 98. 1875.

*Synonym*: *Fusarium alabamense* Sacc., Syll. Fung. 4: 722. 1886, *nom. illegit.*, Art. 52.1.

*Holotypus*: ?K(M).

*Type locality*: **USA**, Alabama, Beaumont.

*Type substrate*: Dead bark*.*

*Notes*: Status unclear. Not *Fusarium fide*Wollenweber & Reinking (1935).  

*erubescens Fusarium* (Durieu & Mont.) Sacc., Syll. Fung. 4: 719. 1886, *nom. illegit.*, Art. 53.1.

*Basionym*: *Fusisporium erubescens* Durieu & Mont., Exploration scientifique de l'Algérie 1–9: 351. 1848.

(See *Fusarium bacilligerum*)

*Holotypus*: ?PC.

*Type locality*: **Algeria**, Béjaïa.

*Type substrate*: *Rhamnus alaternus.*

*Note*: Synonym *fide*Wollenweber & Reinking (1935).  

*erubescens Fusarium* Appel & Oven, Landwirtsch. Jahrb. 1905, *nom. illegit.*, Art. 53.1.

(See ***Fusarium acuminatum***)

*Authentic material*: Not located.

*Original locality*: **Germany**.

*Original substrate*: *Solanum lycopersicum.*

*Note*: Synonym *fide*Wollenweber & Reinking (1935).  

*eucalypticola Fusarium* Henn., Hedwigia 40: 355. 1901.

*Holotypus*: In B *fide*Hein (1988).

*Type locality*: **Australia**, Western Australia, Cranbrook.

*Type substrate*: *Eucalyptus baxteri* (syn. *E. santalifolia*)

*Notes*: Status unclear. Not *Fusarium fide*Wollenweber & Reinking (1935).  

*eucalyptorum Fusarium* Cooke & Harkn., Grevillea 9: 128. 1881.

(See ***Fusarium oxysporum***)

*Syntype*: BPI 452103.

*Type locality*: **USA**, California, San Francisco Masonic Cemetery.

*Type substrate*: *Eucalyptus* sp.

*Note*: Synonym *fide*Arya & Jain (1962).  

*eucheliae Fusarium* Sartory, R. Sartory & J. Mey., Ann. Mycol. 30: 471. 1932.

(See ***Fusarium equiseti***)

*Lectotypus* (*hic designatus*, MBT 10000683): **France**, digestive track of living caterpillar, 1932, A. Sartory, R. Sartory & J. Meyer, in Ann. Mycol. 30: 473, figs 1–13.

*Notes*: Synonym *fide*Wollenweber & Reinking (1935). No holotype specimen could be located and therefore an illustration is designated as lectotype.  

*eumartii Fusarium* C.W. Carp., J. Agric. Res. 5: 204. 1915.

(See *Fusarium solani*)

*Lectotypus*: Illustration Plate XIV, number 4, in Carpenter (1915), designated in Sandoval-Denis *et al.* (2019).

*Type locality*: **Unknown**.

*Type substrate*: *Solanum tuberosum.*  

*euonymi Fusarium* Syd., Beibl. Hedwigia 39: (6). 1900.

(See ***Fusarium lateritium***)

*Syntype*: S-F45621 (Sydow, Mycoth. March. no. 4896).

*Type al locality*: **Germany**, Berlin.

*Type substrate*: *Euonymus bungeanus.*

*Note*: Synonym *fide*Wollenweber & Reinking (1935).  

*euonymi-japonici Fusarium* Henn., Hedwigia 41: 139. 1902.

(See ***Fusarium lateritium***)

*Holotypus*: In B *fide*Hein (1988).

*Type locality*: **Germany**, Berlin.

*Type substrate*: *Euonymus japonicus.*

*Note*: Synonym *fide*Wollenweber & Reinking (1935).  

*euwallaceae Fusarium* S. Freeman *et al.*, Mycologia 105: 1599. 2013.

***Neocosmospora euwallaceae*** (S. Freeman *et al.*) Sand.-Den. *et al.*, Persoonia 43: 129. 2019.

*Holotypus*: BPI 884203.

*Ex-type culture*: CBS 135854 = NRRL 54722.

*Type locality*: **Israel**, central coastal region, Kibbutz Glil Yam.

*Type substrate*: *Euwallacea* sp. beetle infecting *Persea americana* cv. Hass*.*

*Descriptions and illustrations*: See Freeman *et al.* (2013).

*Diagnostic DNA barcodes*: *rpb1*: JQ038021; *rpb2*: JQ038028; *tef1*: JQ038007.  

***expansum Fusarium*** Schltdl., Fl. Berol. 2: 139. 1824.

*Synonym*: ?*Fusarium carpini* Schulzer & Sacc., Hedwigia 23: 128. 1884.

*Fusarium socium* Sacc., Atti Ist. Veneto Sci. Lett. Arti, sér. 6, 2: 450. 1884.

*Fusarium cirrosum* Höhn., Sitzungsber. Kaiserl. Akad. Wiss. Wien, Math.-Naturwiss. Cl., Abt. 1., 116: 153. 1907.

*Fusarium macounii* Dearn., Mycologia 9: 363. 1917.

*Holotypus*: HAL 1614 F.

*Type locality*: **Germany**, Berlin.

*Type substrate*: *Carpinus betulus.*

*Descriptions and illustrations*: See Wollenweber (1916–1935) and Gerlach & Nirenberg (1982).

*Notes*: Both Wollenweber & Reinking (1935) and Gerlach & Nirenberg (1982) recognised this species. This species requires epitypification from the type locality.  

***fabacearum Fusarium*** L. Lombard *et al.*, Persoonia 43: 24. 2018 [2019].

*Holotypus*: CBS H-23613.

*Ex-type culture*: CBS 144743 = CPC 25802.

*Type locality*: **South Africa**, Western Cape Province.

*Type substrate*: *Glycine max.*

*Descriptions and illustrations*: See Lombard *et al.* (2019b).

*Diagnostic DNA barcodes*: *rpb1*: MW928806; *rpb2*: MH484938; *tef1*: MH485029.  

*falcatum Fusarium* Appel & Wollenw., Arbeiten Kaiserl. Biol. Anst. Land-Forstw. 8: 184. 1913.

*Replaced synonym*: *Fusarium vasinfectum* var. *pisi* Schikora, Arbeiten Biol. Anst. Land-Forstwirt. 5: 188, pl. 7. 1906, *nom. illegit.*, Art. 53.1

(See ***Fusarium equiseti***)

*Holotypus*: Not located.

*Type locality*: **Germany**, Berlin.

*Type substrate*: *Pisum sativum.*

*Notes*: Synonym *fide*Wollenweber & Reinking (1935).  

*falciforme Fusarium* (Carrión) Summerb. & Schroers, J. Clin. Microbiol. 40: 2872. 2002.

***Neocosmospora falciformis*** (Carrión) L. Lombard & Crous, Stud. Mycol. 80: 227. 2015.

*Basionym*: *Cephalosporium falciforme* Carrión, Mycologia 43: 523. 1951.

*Synonyms*: *Acremonium falciforme* (Carrión) W. Gams, Cephalosporium-artige Schimmelpilze: 139. 1971.

*Fusarium paranaense* S.S. Costa *et al.*, Fungal Biology 120: 55. 2015 [2016].

*Holotypus*: CBS 475.67 (preserved as metabolically inactive culture).

*Ex-type culture*: CBS 475.67 = IHM 939 = IMI 268681.

*Type locality*: **Puerto Rico**.

*Type substrate*: Mycetoma from *Homo sapiens.*

*Diagnostic DNA barcodes*: *rpb1*: MW218114; *rpb2*: LT960558; *tef1*: LT906669.  

***fasciculatum Fusarium*** J.W. Xia *et al.*, Persoonia 43: 203. 2019.

*Holotypus*: CBS H-24057.

*Ex-type culture*: CBS 131382.

*Type locality*: **Australia**, Northern Territories, Roper River area.

*Type substrate*: Stems of *Oryza australiensis.*

*Descriptions and illustrations*: See Xia *et al.* (2019).

*Diagnostic DNA barcodes*: *rpb2*: MN170406; *tef1*: MN170473.  

*fautreyi Fusarium* Sacc., Syll. Fung. 10: 934. 1892.

*Replaced synonym*: *Fusarium parasiticum* Fautrey, Rev. Mycol. (Toulouse) 11: 153. 1889, *nom. illegit.*, Art. 53.1.

(See ***Fusarium lateritium***)

*Typus*: BR5020140789424.

*Type locality*: **France**, Noidan.

*Type substrate*: *Vitis vinifera.*

*Note*: Synonyms *fide*Wollenweber & Reinking (1935).  

*ferrugineum Fusarium* (Sand.-Den. & Crous) O'Donnell *et al.*, Index Fungorum 440: 2. 2020.

***Neocosmospora ferruginea*** Sand.-Den. & Crous, Persoonia 43: 130. 2019.

*Holotypus*: CBS H-23981.

*Ex-type culture*: CBS 109028 = NRRL 32437.

*Type locality*: **Switzerland**.

*Type substrate*: Subcutaneous nodule of *Homo sapiens.*

*Descriptions and illustrations*: See Sandoval-Denis *et al.* (2019).

*Diagnostic DNA barcodes*: *rpb1*: HM347157*; rpb2*: EU329581; *tef1*: DQ246979.  

*ferruginosum Fusarium* Sherb., Mem. Cornell Univ. Agric. Exp. Sta. 6: 190. 1915.

(See ***Fusarium acuminatum***)

*Typus*: ?CUP-007445.

*Type locality*: **USA**, New York, Long Island

*Type substrate*: *Solanum tuberosum*

*Notes*: Synonym *fide*Wollenweber & Reinking (1935). Lectotypification pending study of material lodged in CUP.  

***ficicrescens Fusarium*** Al-Hatmi *et al.*, Fungal Biol. 120: 274. 2015 [2016].

*Holotypus*: CBS H-21815.

*Ex-type culture*: CBS 125178.

*Type locality*: **Iran**, Estahban.

*Type substrate*: Fruit of *Ficus carica.*

*Descriptions and illustrations*: See Al-Hatmi *et al.* (2016).

*Diagnostic DNA barcodes*: *rpb1*: MT010950; *rpb2*: MT010977; *tef1*: MT011004.  

*filiferum Fusarium* (Preuss) Wollenw., Fusaria Autogr. Delin. 1: 220. 1916.

*Basionym*: *Fusoma filiferum* Preuss, Linnaea 25: 73. 1852.

*Synonym*: *Fusarium scirpi* var. *filiferum* (Preuss) Wollenw., Fusaria Autogr. Delin. 3: 936. 1930.

(See ***Fusarium scirpi***)

*Holotypus*: Not located.

*Type locality*: **Germany**.

*Type substrate*: Bark of *Pinus* sp*.*

*Note*: Synonym *fide*Wollenweber & Reinking (1935).  

*filisporum Fusarium* (Cooke) Sacc., Syll. Fung. 4: 708. 1886.

*Basionym*: *Fusisporium filisporum* Cooke, Grevillea 8: 8. 1879.

(See *Fusarium ciliatum*)

*Holotypus*: In K(M), Muller s.n. *fide* Index Fungorum.

*Type locality*: **UK**, Eastbourne.

*Type substrate*: *Orthotrichum* sp*.*

*Note*: Synonym *fide*Wollenweber & Reinking (1935).  

*fissum Fusarium* Peyl, Lotos 8: 30. 1858.

(See *Fusarium candidum* (Link.) Sacc.)

*Lectotypus* (*hic designatus*, MBT 10000684): **Germany**, twigs of *Citrus aurantiacum*, 1858, J. Peyl, in Lotos 8, fig. 17.

*Notes*: Synonym *fide*Wollenweber & Reinking (1935). No holotype specimen could be located and therefore an illustration is designated as lectotype.  

***flagelliforme Fusarium*** J.W. Xia *et al.*, Persoonia 43: 204. 2019.

*Holotypus*: CBS H-24058.

*Ex-type culture*: CBS 162.57 = NRRL 36269.

*Type locality*: **Croatia**, Zagreb.

*Type substrate*: Seedlings of *Pinus nigra.*

*Descriptions and illustrations*: See Xia *et al.* (2019).

*Diagnostic DNA barcodes*: *rpb2*: GQ505823; *tef1*: GQ505645.  

*flavidum Fusarium* (Bonord.) Sacc., Syll. Fung. 4: 698. 1886.

*Basionym*: *Fusisporium flavidum* Bonord., Bot. Zeitung (Berlin) 19: 194. 1861.

(See ***Fusarium reticulatum***)

*Lectotypus* (*hic designatus*, MBT 10000685): **Germany**, rotten tree, 1861, H.F. Bonorden, in Bot. Zeitung (Berlin) 19: tab. VIII, fig. 3.

*Notes*: Synonym *fide*Wollenweber & Reinking (1935). No holotype specimen could be located and therefore an illustration is designated as lectotype.  

*flavum Fusarium* (Fr.) Wollenw., Z. Parasitenk. (Berlin) 3: 305. 1931.

*Basionym*: *Fusisporium flavum* Fr., Syst. Mycol. 3: 444. 1832.

(See *Fusarium dimerum*)

*Holotypus*: Not located.

*Type locality*: **Germany**, Bonn.

*Type substrate*: *Aster* sp*.*

*Note*: Synonym *fide*Booth (1971).  

***flocciferum Fusarium*** Corda, in Sturm, Deutschl. Fl., Abt. 3, Pilze Deutschl. 2: 17. 1828.

*Synonyms*: *Fusarium vinosum* Massee, Brit. Fung.-Fl. 3: 479. 1893.

*Fusarium clavatum* Sherb., Mem. Cornell Univ. Agric. Exp. Sta. 6: 234. 1915.

*Fusarium idahoanum* O.A. Pratt, J. Agric. Res. 13: 86. 1918.

*Fusarium nigrum* O.A. Pratt, J. Agric. Res. 13: 90. 1918.

*Lectotypus*: (*hic designatus*, MBT 10001326) **Germany**, Berlin, on shell of the fruit of *Aesculus hippocastanum*. AKJ. Corda, Sturm, Deutschl. Fl., Abt. 3, Pilze Deutschl. 2, pl. 7.

*Epitypus* (*hic designatus*, MBT 10000686): **Germany**, greenhouse soil, 1966, D. Bredemeier, CBS 821.68 (preserved as metabolically inactive culture).

*Ex-epitype culture*: CBS 821.68 = NRRL 28450.

*Descriptions and illustrations*: See Booth (1971) and Gerlach & Nirenberg (1982).

*Diagnostic DNA barcodes*: *rpb1*: MW928807; *rpb2*: MW928824; *tef1*: MW928837.

*Notes*: Corda's original illustration of *Fusarium flocciferum* is here selected as lectotype. Gerlach & Nirenberg (1982) considered isolate CBS 821.68, along with CBS 792.70, as good representatives of *F. flocciferum*. Based on their observations and collection locality, CBS 821.68 is designated as epitype of *F. flocciferum*.  

*floridanum Fusarium* T. Aoki *et al.*, Mycologia 111: 922. 2019.

***Neocosmospora floridana*** (T. Aoki *et al.*) L. Lombard & Sand.-Den., ***comb. nov*.** MycoBank MB 837664.

*Basionym*: *Fusarium floridanum* T. Aoki *et al.*, Mycologia 111: 922. 2019.

*Holotypus*: BPI 910972.

*Ex-type culture*: MAFF 246849 = NRRL 62628.

*Type locality*: **USA**, Florida, Gainsville.

*Type substrate*: Mycangium of *Euwallacea interjectus* infesting *Acer negundo.*

*Descriptions and illustrations*: See Aoki *et al.* (2019).

*Diagnostic DNA barcodes*: *rpb1*: KC691593; *rpb2*: KC691624, KC691653; *tef1*: KC691535.

*Notes*: A new combination is provided in the genus *Neocosmospora* based on the phylogenetic relationship (Aoki *et al.* 2019) of this species to other *Neocosmospora* spp. in the ambrosia clade.  

*foeni Fusarium* (Berk. & Broome) Sacc., Syll. Fung. 4: 699. 1886.

*Basionym*: *Fusisporium foeni* Berk. & Broome, Ann. Mag. Nat. Hist., ser. 2, 7: 179. 1851.

(See *Fusarium merismoides*)

*Holotypus*: ?K(M).

*Type locality*: **UK**, Northamptonshire, Apethrope.

*Type substrate*: A hay stalk*.*

*Note*: Synonym *fide*Wollenweber & Reinking (1935).  

***foetens Fusarium*** Schroers *et al.*, Mycologia 96: 398. 2004.

*Holotypus*: CBS 110286 (preserved as metabolically inactive culture).

*Ex-type culture*: CBS 110286 = NRRL 31852 = PD 2001/7244.

*Type locality*: **Netherlands**, Zuid-Holland Province, Maasland.

*Type substrate*: *Begonia elatior* hybrid*.*

*Descriptions and illustrations*: See Schroers *et al.* (2004) and Leslie & Summerell (2006).

*Diagnostic DNA barcodes*: *rpb1*: MW928808; *rpb2*: MW928825; *tef1*: AY320087.  

*foliicola Fusarium* Allesch., Hedwigia 34: 289. 1895.

(See *Fusarium cymbiferum*)

*Holotypus*: In M.

*Type locality*: **Germany**, Oberammergau.

*Type substrate*: *Arabis alpina.*

*Note*: Synonym *fide*Wollenweber & Reinking (1935).  

***fracticaudum Fusarium*** Herron *et al.*, Stud. Mycol*.* 80: 137. 2015.

*Holotypus*: PREM 60895.

*Ex-type culture*: CBS 137233 = CMW 25245.

*Type locality*: **Colombia**, Risaralda, Angela Maria (Santa Rosa).

*Type substrate*: *Pinus maximinoi.*

*Descriptions and illustrations*: See Herron *et al.* (2015).

*Notes*: Comparisons of recently generated sequences for the living ex-type (CBS 137233 = CMW 25245) of *F. fracticaudum* indicate a strain transposition or contamination by another *Fusarium* species. Therefore, this species needs to be recollected from the type locality and substrate or sequences need to be generated from the holotype specimen.  

***fractiflexum Fusarium*** T. Aoki *et al.*, Mycoscience 42: 462. 2001.

*Holotypus*: NIAES 20515.

*Ex-type culture*: MAFF 237529 = NRRL 28852.

*Type locality*: **Japan**, Yamanashi, Enzan.

*Type substrate*: *Cymbidium* sp*.*

*Descriptions and illustrations*: See Aoki *et al.* (2001).

*Diagnostic DNA barcodes*: *rpb1*: LR792578; *rpb2*: LT575064; *tef1*: AF160288.  

*fractum Fusarium* Sacc. & Cavara, Nuovo Giorn. Bot. Ital., n.s., 7: 308. 1900.

(See *Fusarium candidum* (Link) Sacc.)

*Holotypus*: In PAD.

*Type locality*: **Italy**.

*Type substrate*: *Fagus* sp.

*Note*: Synonym *fide*Wollenweber & Reinking (1935).  

*fragrans Fusarium* P. Crouan & H. Crouan, Fl. Finistère: 14. 1867.

(See *Fusarium candidum* (Link) Sacc.)

*Holotypus*: ?PC.

*Type locality*: **France**.

*Type substrate*: *Salix* sp.

*Note*: Synonym *fide*Wollenweber & Reinking (1935).  

*fraxini Fusarium* Allesch., Ber. Bot. Vereines Landshut 12: 130. 1892.

(See ***Fusarium sambucinum***)

*Holotypus*: In M.

*Type locality*: **Germany**, München.

*Type substrate*: *Fraxinus excelsior*.

*Note*: Synonym *fide*Wollenweber & Reinking (1935).  

*fraxini Fusarium* Kabát & Bubák, Fungi Imperf. Exs., no. 900. 1912, *nom. illegit.*, Art. 53.1.

***Fusicoccum fraxini*** Sherb., Phytopathology 18: 148. 1928.

*Authentic material*: BPI 451324.

*Original locality*: **Czech Republic**.

*Original substrate*: *Fraxinus excelsior*.  

***fredkrugeri Fusarium*** Sand.-Den. *et al.*, MycoKeys 34: 79. 2018.

*Holotypus*: CBS H-23496.

*Ex-type culture*: CBS 144209 = CPC 33747.

*Type locality*: **South Africa**, Kruger National Park, Skukuza, Granite Supersite.

*Type substrate*: Rhizosphere soil of *Melhania acuminata.*

*Descriptions and illustrations*: See Sandoval-Denis *et al.* (2018b).

*Diagnostic DNA barcodes*: *rpb1*: LT996199; *rpb2*: LT996147; *tef1*: LT996097.  

*fructigenum Fusarium* Fr., Syst. Mycol. 3: 471. 1832.

(See ***Fusarium lateritium***)

*Holotypus*: Not located.

*Type locality*: **Unknown**.

*Type substrate*: Fruit of *Rosa pomifera*.

*Note*: Synonym *fide*Wollenweber & Reinking (1935).  

*fuckelii Fusarium* Sacc., Syll. Fung. 4: 695. 1886.

***Geejayessia desmazieri*** (Becc. & De Not.) Schroers *et al.*, Stud. Mycol. 68: 130. 2011.

*Basionym*: *Nectria desmazieri* Becc. & De Not., Schem. di Classif. Sferiacei: 10. 1863.

*Synonyms*: *Dialonectria desmazieri* (Becc. & De Not.) Petch, Naturalist (London): 281. 1937.

*Nectria coccinea* var. *cicatricum* Desm., Ann. Sci. Nat., Bot 10: 351. 1848 (*fide* Wollenweber & Reinking 1935 and Booth 1971).

*Nectria gibbera* Fuckel, Jahrb. Nassauischen Vereins Naturk. 23–24: 177. 1870.

*Lectotypus*: G 00110886 (Fuckel, Fungi Rhen. No. 2357), designated in Schroers *et al.* (2011).

*Type locality*: **Germany**, Rheingau.

*Type substrate*: *Buxus sempervirens*.  

***fujikuroi Fusarium*** Nirenberg, Mitt. Biol. Bundesanst. Land- Forstw. Berlin-Dahlem 169: 32. 1976

*Synonyms*: *Lisea fujikuroi* Sawada, Special Bull. Agric. Exp. Sta. Gov. Formosa 19: 251. 1919.

*Gibberella fujikuroi* (Sawada) Wollenw., Z. Parasitenk. (Berlin) 3: 514. 1931.

?*Gibberella fujikuroi* var. *subglutinans* E.T. Edwards, Agric. Gaz. New South Wales 44: 895. 1933.

?*Gibberella subglutinans* (E.T. Edwards) P.E. Nelson *et al.*, Fusarium species. An illustrated manual for identification (University Park): 135. 1983.

?*Oospora cephalosporioides* Luchetti & Favilli, Annali Fac. Agrar. R. Univ. Pisa 1: 399. 1938.

?*Gibberella fujikuroi f*. *oryzae* Saccas, Rev. Pathol. Veg. Entomol. Agric. France 30: 77. 1951.

?*Gibberella fujikuroi* var. *intermedia* Kuhlman, Mycologia 74: 766. 1982.

*Holotypus*: IMI 202879.

*Ex-type culture*: BBA 12428 = BBA 63630 = CBS 221.76 = IHEM 3821 = IMI 196086 = IMI 202879 = NRRL 13620 = NRRL 13998 = NRRL 22174.

*Type locality*: **Taiwan**.

*Type substrate*: *Oryza sativa.*

*Descriptions and illustrations*: See Nirenberg (1976), Gerlach & Nirenberg (1982) and Leslie & Summerell (2006).

*Diagnostic DNA barcodes*: *rpb1*: JX171456; *rpb2*: JX171570; *tef1*: AF160279.  

***fuliginosporum Fusarium*** Sibilia, Ann. Reale. Ist. Super. Agrar. Forest., ser. 2, 1: 77. 1925.

*Holotypus*: Not located.

*Type locality*: **Italy**.

*Type substrate*: Forest containing mostly *Cedrus deodara*.

*Note*: Mentioned by Wollenweber & Reinking (1935), but no additional records of this species could be located.  

*fungicola Fusarium* (Har. & P. Karst.) Sacc., Syll. Fung. 10: 730. 1892.

?***Alysidium hypophleodes*** (Corda) Bonord., Handb. Allg. Mykol.: 35. 1851.

*Basionym*: *Fusidium hypophleodes* Corda, Icon. Fung. 1: 3, tab. 1, fig. 50. 1837.

*Synonym*: *Fusamen fungicola* Har. & P. Karst. (as ‘*fungicolum*’), Rev. Mycol. (Toulouse) 12: 129. 1890.

*Holotypus*: Not located.

*Type locality*: **Finland**, Mustiala.

*Type substrate*: *Lenzites betulina*.

*Note*: Synonyms *fide*Wollenweber & Reinking (1935).  

*funicola Fusarium* Tassi, Bull. Lab. Orto Bot. Reale Univ. Siena 3: 131. 1900.

(See ***Fusarium graminearum***)

*Holotypus*: ?SIENA.

*Type locality*: **Italy**.

*Type substrate*: Rotten string.

*Note*: Synonym *fide*Wollenweber & Reinking (1935).  

*fusarioides Fusarium* (Gonz. Frag. & Cif.) C. Booth, The Genus Fusarium: 88. 1971.

*Basionym*: *Dactylium fusarioides* Gonz. Frag. & Cif., Bol. Real Soc. Esp. Hist. Nat. 27: 280. 1927.

(See ***Fusarium chlamydosporum***)

*Holotypus*: ?MA-Funhist: 7609-1.

*Type locality*: **Dominican Republic**, Moca.

*Type substrate*: leaves of *Crotalaria* sp*.*

*Note*: Synonym *fide*Gerlach & Nirenberg (1982).  

*fuscum Fusarium* (Bonord.) Sacc., Syll. Fung. 4: 699. 1886.

*Basionym*: *Selenosporium fuscum* Bonord., Handb. Mykol.: 135. 1851.

(See *Fusarium citrulli* Sartory)

*Holotypus*: Not preserved *fide*Holubová-Jechová *et al.* (1994).

*Type locality*: **Germany**.

*Type substrate*: Bark.

*Note*: Synonym *fide*Wollenweber & Reinking (1935).  

***gaditjirrii Fusarium*** Phan *et al.*, Stud. Mycol. 50: 265. 2004.

*Synonym*: *Gibberella gaditjirrii* Phan *et al.*, Stud. Mycol. 50: 264. 2004.

*Holotypus*: DAR 76663.

*Ex-type culture*: CBS 116011 = F15048 = NRRL 53678.

*Type locality*: **Australia**, Queensland, Walkamin Research Station.

*Type substrate*: *Heteropogon triticeus.*

*Descriptions and illustrations*: See Phan *et al.* (2004).

*Diagnostic DNA barcodes*: *rpb2*: HQ662690; *tef1*: AY639636.  

*gallinaceum Fusarium* Cooke & Harkn., Grevillea 9: 8. 1880.

(See *Fusarium merismoides*)

*Holotypus*: BPI 452133.

*Type locality*: **USA**, California, Sausalito.

*Type substrate*: Feathers of *Gallus* sp. (chicken).

*Note*: Synonym *fide*Wollenweber & Reinking (1935).  

***gamsii Fusarium*** Torbati *et al.*, Mycol. Progr. 18: 127. 2018 [2019].

*Holotypus*: CBS H-23561.

*Ex-type culture*: CBS 143610 = CPC 30862.

*Type locality*: **Iran**, West Azerbaijan Province, Orumieh-Salmas.

*Type substrate*: *Agaricus bisporus.*

*Descriptions and illustrations*: See Torbati *et al.* (2019).

*Diagnostic DNA barcodes*: *rpb2*: LT970760; *tef1*: LT970788.  

*gaudefroyanum Fusarium* Sacc., Michelia 2: 132. 1880.

(See ***Fusarium avenaceum***)

*Holotypus*: In PAD.

*Type locality*: **France**, Paris.

*Type substrate*: *Cyperaceae*.

*Note*: Synonym *fide*Wollenweber & Reinking (1935).  

*gemmiperda Fusarium* Aderh., Z. Pflanzenkrankh. 11: 70. 1901.

(See ***Fusarium lateritium***)

*Lectotypus* (*hic designatus*, MBT 10000687): **Germany**, *Prunus cerasus*, 1901, R. Aderhold, in Z. Pflanzenkrankh. 11: pl. II, figs 1–4.

*Notes*: Synonym *fide*Wollenweber & Reinking (1935). No holotype specimen could be located and therefore an illustration is designated as lectotype.  

*genevense Fusarium* Dasz., Bull. Soc. Bot. Genève, sér. 2, 4: 305. 1912.

(See ***Fusarium sambucinum***)

*Lectotypus* (*hic designatus*, MBT 10000688): **Switzerland**, Geneva, from soil, 1912, M. Daszewska, in Bull. Soc. Bot. Genève, sér. 2, 4: 306, fig. 27.

*Notes*: Synonym *fide*Wollenweber & Reinking (1935). No holotype specimen could be located and therefore an illustration is designated as lectotype.  

*georginae Fusarium* Corda, Icon. Fung. 2: 4. 1838.

(See *Fusarium cymbiferum*)

*Typus*: In PRM *fide*Pilat (1938).

*Type locality*: **Czech Republic**, Prague.

Type substrate: *Dahlia* sp.

*Notes*: Synonym *fide*Wollenweber & Reinking (1935). Lectotypification pending study of material lodged in PRM.  

***gerlachii Fusarium*** T. Aoki *et al.*, Fungal Genet. Biol. 44: 1202. 2007.

*Holotypus*: BPI 871657.

*Ex-type culture*: LRG 00-551 = NRRL 36905.

*Type locality*: **USA**, Minnesota, Polk County, Climax.

*Type substrate*: *Triticum aestivum.*

*Descriptions and illustrations*: See Starkey *et al.* (2007).

*Diagnostic DNA barcodes*: *rpb1*: KM361646; *rpb2*: KM361664; *tef1*: DQ459742.  

*gibbosum Fusarium* Appel & Wollenw., Arbeiten Kaiserl. Biol. Anst. Land- Forstw. 8: 190. 1910 [1913].

(See ***Fusarium equiseti***)

*Holotypus*: ?BPI 452135.

*Type locality*: **Germany**, Berlin

*Type substrate*: *Solanum tuberosum*

*Note*: Synonym *fide*Booth (1971).  

***gigas Fusarium*** Speg. Anales Soc. Ci. Argent. 22: 221. 1886.

*Holotypus*: In LPS *fide*Farr (1973).

*Type locality*: **Paraguay**.

*Type substrate*: *Bambusa* sp*.*

*Descriptions and illustrations*: See Wollenweber & Reinking (1935), Booth (1971) and Gerlach & Nirenberg (1982).

*Notes*: This species requires epitypification. Wollenweber & Reinking (1935), Booth (1971), and Gerlach & Nirenberg (1982) accepted this species, although limited information is available.  

*glandicola Fusarium* Cooke & W.R. Gerard, Grevillea 7: 14. 1878.

***Tubercularia glandicola*** (Cooke & W.R. Gerard) Wollenw. & Reinking, *Fusarien*: 325. 1935.

*Holotypus*: In K(M), Gerard s.n. *fide* Index Fungorum.

*Type locality*: **USA**, New York.

*Type substrate*: Acorns of *Quercus* sp.

*Note*: Synonym *fide*Wollenweber & Reinking (1935).  

*glandicola Fusarium* Allesch., Ber. Bot. Vereines Landshut 12: 130. 1892, *nom. illegit.*, Art. 53.1.

*Replacing synonym*. *Fusarium allescheri* Sacc. & P. Syd., Syll. Fung. 14: 1128. 1899.

(See *Fusarium melanochlorum*)

*Authentic material*: In M.

*Original locality*: **Germany**, München.

*Original substrate*: Fruits of *Quercus robur* (syn. *Q. pedunculata*).

*Note*: Synonyms *fide*Wollenweber & Reinking (1935).  

*gleditschiae Fusarium* Therry (as ‘*gledrischiae*’), in Roumeguère, Fungi Sel. Gall. Exs.: no. 5496. 1890, *nom. nud.*, Art. 38.1(a).

***Gloeosporium gleditschiae*** Therry ex Wollenw., Z. Parasitenk. (Berlin) 3: 437. 1931.

*Note*: Synonym *fide*Wollenweber & Reinking (1935).  

*gleditschiicola Fusarium* Dearn. & Barthol. (as ‘*gleditsiaecolum*’), Mycologia 9: 363. 1917.

(See ***Fusarium lateritium***)

*Holotypus*: JD 4379 in DAOM.

*Type locality*: **USA**, Kansas, Stockton.

*Type substrate*: *Gleditsia triacanthos*.

*Note*: Synonym *fide*Wollenweber & Reinking (1935).  

***globosum Fusarium*** Rheeder *et al.*, Mycologia 88: 509. 1996.

*Holotypus*: BPI 802834.

*Ex-type culture*: CBS 428.97 = DOAM 214966 = FRC M-8014 = IMI 375330 = MRC 6647 = NRRL 26131 = PREM 51878.

*Type locality*: **South Africa**, Eastern Cape Province, Butterworth district, Teko Experimental Farm.

*Type substrate*: *Zea mays.*

*Descriptions and illustrations*: See Rheeder *et al.* (1996) and Leslie & Summerell (2006).

*Diagnostic DNA barcodes*: *rpb1*: KF466396; *rpb2*: KF466406; *tef1*: KF466417.  

*globulosum Fusarium* Pass., in Rabenhorst, Fungi Eur. Exs. no. 2262. 1877.

*Syntypes*: In BPI, CUP, ILL & S (Fungi Eur. Exs. # 2262).

*Type locality*: **Italy**, Parma.

*Type substrate*: *Salvia verticillata.*

*Note*: Not *Fusarium fide*Wollenweber & Reinking (1935).  

*gloeosporioides Fusarium* Speg. (as ‘*gloeosporoide*’), Anales Mus. Nac. Hist. Nat. Buenos Aires 6: 350. 1898 [1899].

(See ***Fusarium incarnatum***)

*Holotypus*: In LPS *fide*Farr (1973).

*Type locality*: **Argentina**, La Plata.

*Type substrate*: Fruits of *Passiflora tweediana*.

*Note*: Synonym *fide*Wollenweber & Reinking (1935).  

*gloeosporioides Fusarium* (Speg.) Sacc. & Trotter, Syll. Fung. 22: 1482. 1913, *nom. illegit.*, Art. 53.1.

*Basionym*: *Selenosporium gloeosporioides* Speg. (as ‘*gloesporioides*’), Anales Mus. Nac. Hist. Nat. Buenos Aires 13: 458. 1911.

(See ***Fusarium lateritium***)

*Holotypus*: In LPS (Myc. Argent. ser. 5, no. 1167) *fide*Farr (1973).

*Type locality*: **Argentina**, Buenos Aires.

*Type substrate*: *Pircunia dioica*.

*Note*: Synonym *fide*Wollenweber & Reinking (1935).  

*glumarum Fusarium* Sacc., Syll. Fung. 4: 706. 1886.

*Replaced synonym*: *Fusarium pallens* Berk. & M.A. Curtis, Grevillea 3: 99. 1875, *nom. illegit.*, Art. 53.1, *non Fusarium pallens* Nees & T. Nees 1818.

(See ***Fusarium incarnatum***)

*Syntype*: CBRU00007755.

*Type locality*: **USA**.

*Type substrate: Juncus* sp.

*Note*: Synonym *fide*Wollenweber & Reinking (1935).  

***glycines Fusarium*** L. Lombard *et al.*, Persoonia 41: 25. 2018 [2019].

*Holotypus*: CBS H-23614.

*Ex-type culture*: CBS 144746 = CPC 25808.

*Type locality*: **South Africa**, North West Province.

*Type substrate*: *Glycine max.*

*Descriptions and illustrations*: See Lombard *et al.* (2019b).

*Diagnostic DNA barcodes*: *rpb1*: MW928809; *rpb2*: MH484942; *tef1*: MH485033.  

***goolgardi Fusarium*** D.M. Robinson *et al.*, Fungal Diversity 77: 357. 2015 [2016].

*Holotypus*: RGB5411.

*Ex-type culture*: NRRL 66250 = RGB5411.

*Type locality*: **Australia**, New South Wales, Bungonia State Conservation Area.

*Type substrate*: *Xanthorrhoea glauca.*

*Descriptions and illustrations*: See Laurence *et al.* (2016).

*Diagnostic DNA barcodes*: *rpb1*: KP083270; *rpb2*: KP083280; *tef1*: KP101123.  

***gossypinum Fusarium*** L. Lombard & Crous, Persoonia 41: 26. 2018 [2019].

*Holotypus*: CBS H-23615.

*Ex-type culture*: CBS 116613.

*Type locality*: **Ivory Coast**, Bouaké.

*Type substrate*: *Gossypium hirsutum.*

*Descriptions and illustrations*: See Lombard *et al.* (2019b).

*Diagnostic DNA barcodes*: *rpb2*: MH484909; *tef1*: MH485000.  

*gracile Fusarium* McAlpine, Proc. Linn. Soc. New South Wales 28: 554. 1903.

(See ***Fusarium avenaceum***)

*Holotypus*: VPRI 2564.

*Type locality*: **Australia**, Victoria, Sandringham.

*Type substrate*: Flowering stem of *Lobelia gibbosa*.

*Note*: Synonym *fide*Wollenweber & Reinking (1935).  

***gracilipesFusarium*** J.W. Xia *et al.*, Persoonia 43: 205. 2019.

*Holotypus*: CBS H-24059.

*Ex-type culture*: NRRL 43635.

*Type locality*: **USA**, Nebraska.

*Type substrate*: *Equus* sp. (horse)*.*

*Descriptions and illustrations*: See Xia *et al.* (2019).

*Diagnostic DNA barcodes*: *rpb1*: HM347188; *rpb2*: GQ505840; *tef1*: GQ505662.  

***graminearum Fusarium*** Schwabe, Fl. Anhalt. 2: 285. 1839.

*Synonyms*: *Sphaeria zeae* Schwein., Schriften Naturf. Ges. Leipzig 1: 48. 1822, *non Fusarium zeae* (Westend.) Sacc. 1886.

*Dothidea zeae* (Schwein.) Schwein., Trans. Amer. Philos. Soc., n.s., 4: 230. 1832.

*Hendersoniopsis zeae* (Schwein.) Woron., Fungal and Bacterial Diseases of Agricultural Plants: 255. 1922.

*Gibberella zeae* (Schwein.) Petch, Ann. Mycol. 34: 260. 1936.

*Fusarium stictoides* Durieu & Mont., Explor. Sci. Algérie 1: 334. 1848.

*Sphaeria saubinetii* Durieu & Mont., Explor. Sci. Algérie 1: 479. 1849.

*Gibbera saubinetii* (Durieu & Mont.) Mont., Syll. Gen. Sp. Crypt.: 252. 1856.

*Botryosphaeria saubinetii* (Durieu & Mont.) Niessl, Verh. Naturf. Vereins Brünn 10: 195. 1872.

*Gibberella pulicaris* subsp. *saubinetii* (Durieu & Mont.) Sacc., Michelia 1: 317. 1878.

*Gibberella saubinetii* (Durieu & Mont.) Sacc., Michelia 1: 513. 1879.

*Fusisporium insidiosum* Berk., Gard. Chron. 1860: 480. 1860.

*Fusarium insidiosum* (Berk.) Sacc., Syll. Fung. 4: 707. 1886, *nom. illegit.*, Art. 53.1.

*Gibberella saubinetii* var. *coronillae* Sacc., Michelia 1: 513. 1879.

*Fusarium mollerianum* Thüm., Inst. Coimbra 28: 263. 1881.

*Gibberella saubinetii* subsp. *pachyspora* Sacc., Michelia 2: 74. 1880.

*Gibberella saubinetii* var. *pachyspora* (Sacc.) Sacc., Syll. Fung. 2: 555. 1883.

*Fusarium caricis* Oudem., Verslagen Meded. Afd. Natuurk. Kon. Akad. Wetensch., ser. 3, 7: 325. 1890.

*Fusarium graminearum* var. *caricis* (Oudem.) Wollenw., Z. Parasitenk. (Berlin) 3: 365. 1931.

?*Fusarium rhoicola* Fautrey, Rev. Mycol. (Toulouse) 17: 171. 1895.

*Fusarium funicola* Tassi, Bull. Lab. Orto Bot. Reale Univ. Siena 3: 131. 1900.

*Gibberella saubinetii f*. *acuum* Feltgen, Vorstud. Pilzfl. Luxemburg, Nachtr. III: 303. 1903.

*Gibberella saubinetii* var. *acuum* (Feltgen) Sacc. & D. Sacc., Syll. Fung. 17: 813. 1905.

*Gibberella saubinetii* var. *tetraspora* Feltgen, Vorstud. Pilzfl. Luxemburg, Nachtr. III: 302. 1903.

*Gibberella saubinetii* var. *calami* Henn., Beibl. Hedwigia 42: (79). 1903.

*Gibberella saubinetii* var. *mate* Speg., Anales Mus. Nac. Hist. Nat. Buenos Aires 17: 129. 1908.

?*Selenosporium bufonicola* Speg., Anales Mus. Nac. Hist. Nat. Buenos Aires, ser. 3, 13: 458. 1910.

?*Fusarium bufonicola* (Speg.) Sacc. & Trotter, Syll. Fung. 22: 1486. 1913.

*Fusarium rostratum* Appel & Wollenw., Arbeiten Kaiserl. Biol. Anst. Land- Forstw. 8: 30. 1910 [1913].

*Gibberella saubinetii* var. *flacca* Wollenw., Z. Parasitenk. (Berlin) 3: 433. 1931.

*Lectotypus* (*hic designatus*, MBT 10000689): **Germany**, inflorescence of *Triticum* sp., 1839, S.H. Schwabe, in Flora Anhaltina 2, tab. VI. fig. 7.

*Epitypus* (*hic designatus*, MBT 10000690): **Germany**, *Hordeum vulgare*, 1988, L. Niessen, CBS 136009 (preserved as metabolically inactive culture).

*Ex-epitype culture*: CBS 136009.

*Descriptions and illustrations*: See Booth (1971), Gerlach & Nirenberg (1982) and Leslie & Summerell (2006).

*Diagnostic DNA barcodes*: *rpb1*: MW928810; *rpb2*: MW928826; *tef1*: MW928838.

*Notes*: This well-known and economically important pathogen of gramineous hosts has a global distribution and is accepted as originally circumscribed. However, no type material is available for taxonomic reference. Therefore, a lectotype based on an illustration from the original protologue and an epitype is designated here to provide taxonomic stability for this species.  

***graminum Fusarium*** Corda, Icon. Fung. 1: 3. 1837.

*Synonym*: *Fusarium herbarum* var. *graminum* (Corda) Wollenw., Fusaria Autogr. Delin. 3: 891. 1930.

*Fusarium avenaceum* var. *graminum* (Corda) Raillo, Fungi of the Genus Fusarium: 188. 1950.

*Fusarium corallinum* Sacc., Nuovo Giorn. Bot. Ital. 8: 196. 1876.

*Lectotypus* (*hic designatus*, MBT 10000691): **Germany**, gramineous plant, 1837, A.C.J Corda, in Icon. Fung. 1, tab. I, fig. 59.

*Descriptions and illustrations*: See Wollenweber & Reinking (1935) and Gerlach & Nirenberg (1982).

*Notes*: This species is recognised by Wollenweber & Reinking (1935) and Gerlach & Nirenberg (1982). Recollection from the type host and locality is required. No holotype specimen could be located and therefore an illustration is designated as lectotype.  

*granulare Fusarium* Kalchbr., Crypt. Austro-Afric., no. 1068. 1874.

(See ***Fusarium sambucinum***)

*Holotypus*: ?B 70 0100191 (Crypt. Austro-Afric., no. 1068).

*Type locality*: **South Africa**, Eastern Cape Province, Somerset-East.

*Type substrate*: *Datura stramonium* (syn. *Datura tatula*).

*Note*: Synonym *fide*Wollenweber & Reinking (1935).  

*granulosum Fusarium* Ellis & Everh., Proc. Acad. Nat. Sci. Philadelphia 45: 466. 1894 [1893].

(See ***Fusarium avenaceum***)

*Holotypus*: Commons 2091 in NY.

*Type locality*: **USA**, Delaware, New Castle, Mount Cuba.

*Type substrate*: *Smilax hispida*.

*Note*: Synonym *fide*Wollenweber & Reinking (1935).  

***grosmichelii Fusarium*** Maryani *et al.*, Stud. Mycol. 92: 176. 2018 [2019].

*Holotypus*: InaCC F833 (preserved as metabolically inactive culture).

*Ex-type culture*: InaCC F833.

*Type locality*: **Indonesia**, West Java, Bogor, Suakarya (Megamendung).

*Type substrate*: Pseudostem of *Musa acuminata* var. Pisang Ambon Lumut*.*

*Descriptions and illustrations*: See Maryani *et al.* (2019a).

*Diagnostic DNA barcodes*: *rpb1*: LS479548; *rpb2*: LS479295; *tef1*: LS479744.  

***guilinense Fusarium*** M.M. Wang *et al.*, Persoonia 43: 80. 2019.

*Holotypus*: HAMS 248037.

*Ex-type culture*: CGMCC 3.19495 = LC12160.

*Type locality*: **China**, Guangxi Province, Guilin.

*Type substrate*: Leaf of *Musa nana.*

*Descriptions and illustrations*: See Wang *et al.* (2019).

*Diagnostic DNA barcodes*: *rpb1*: MK289831; *rpb2*: MK289747; *tef1*: MK289594.  

***guttiforme Fusarium*** Nirenberg & O'Donnell, Mycologia 90: 446. 1998.

*Holotypus*: B 70 0001690.

*Ex-type culture*: BBA 69661 = CBS 409.97 = IMI 376113 = NRRL 25295.

*Type locality*: **Brazil**.

*Type substrate*: *Ananas comosus.*

*Descriptions and illustrations*: See Nirenberg & O'Donnell (1998).

*Diagnostic DNA barcodes*: *rpb1*: MT010938; *rpb2*: MT010967; *tef1*: KC514066.  

*gymnosporangii Fusarium* Jaap, Ann. Mycol. 14: 44. 1916.

***Nectria gymnosporangii*** (Jaap) Rossman, Mycotaxon 8: 515. 1979.

*Basionym*: *Calonectria gymnosporangii* Jaap, Ann. Mycol. 14: 10. 1916.

*Synonyms*: *Bactridium gymnosporangii* (Jaap) Wollenw., Fusaria Autogr. Delin. 1: 458. 1916.

*Cylindrocarpon gymnosporangii* (Jaap) Rossman, Mycol. Pap. 150: 31. 1983.

*Holotypus*: In HBG *fide*Rossman (1979).

*Type locality*: **Croatia**, Dalmatia, Lapad near Ragusa.

*Type substrate*: Parasitic on *Gymnosporangium confusum* on *Juniperus phoenicea* branches.  

*haematococcum Fusarium* Nalim *et al.*, Mycologia 103: 1322. 2011.

***Neocosmospora haematococca*** (Berk. & Broome) Samuels *et al.*, Mycologia 103: 1322. 2011.

*Basionym*: *Nectria haematococca* Berk. & Broome, J. Linn. Soc., Bot. 14: 116. 1875.

*Synonyms*: *Dialonectria haematococca* (Berk. & Broome) Cooke, Grevillea 12: 110. 1884.

*Cucurbitaria haematococca* (Berk. & Broome) Kuntze, Revis. Gen. Pl. 3: 461. 1898.

*Hypomyces haematococcus* (Berk. & Broome) Wollenw., Angew. Bot. 8: 191. 1926.

*Haematonectria haematococca* (Berk. & Broome) Samuels & Nirenberg, Stud. Mycol. 42: 135. 1999.

?*Nectria lanata* Pat., Bull. Soc. Mycol. France 8: 52. 1892 (*fide* Samuels 1976).

?*Nectria aurantiella* Speg., Anales Mus. Nac. Hist. Nat. Buenos Aires 6: 287. 1898.

?*Nectria episphaerioides* Penz. & Sacc., Malpighia 11: 511. 1898 [1897].

?*Nectria cinnabarina* var. *jaraguensis* Höhn., Denkschr. Kaiserl. Akad. Wiss. Wien, Math.-Naturwiss. Kl. 83: 18. 1907.

?*Nectria bogoriensis* C. Bernard, Bull. Dép. Agric. Indes Néerl. 11: 45. 1907.

?*Nectria victoriae* Henn., in Rehm, Ann. Mycol. 5: 81. 1907, *nom. inval*., Art. 38.1(a).

?*Nectria calonectricola* Henn., Hedwigia 48: 105. 1908.

?*Nectria citri* Henn., Hedwigia 48: 104. 1908.

?*Nectria luteococcinea* Höhn., Sitzungsber. Kaiserl. Akad. Wiss. Wien, Math.-Naturwiss. Cl., Abt. 1. 118: 299. 1909.

?*Nectria bainii* var. *hypoleuca* Sacc., Nuovo Giorn. Bot. Ital. 23: 205. 1916.

?*Nectria confluens* Seaver, Sci. Surv. Porto Rico & Virgin Islands 8: 44. 1926, *nom. illegit.*, Art. 53.1.

*Lectotypus*: K(M) 252877, designated in Samuels (1976).

*Lectotype locality*: **Sri Lanka**.

*Lectotype substrate*: Unknown

*Epitypus*: BPI 871363, designated in Nalim *et al.* (2011).

*Ex-epitype culture*: CBS 119600 = FRC S-1832.

*Epitype locality*: **Sri Lanka**, Sabaragamuwa Province, Sinharaja Man and Biosphere Reserve, Morningside, vicinity Bungalow in forested slope.

*Epitype substrate*: Dying tree.

*Descriptions and illustrations*: See Nalim *et al.* (2011).

*Diagnostic DNA barcodes*: *rpb2*: LT960561; *tef1*: KM231926.  

***hainanense Fusarium*** M.M. Wang *et al.*, Persoonia 43: 82. 2019.

*Holotypus*: HAMS 248038.

*Ex-type culture*: CGMCC 3.19478 = LC11638.

*Type locality*: **China**, Hainan Province.

*Type substrate*: Stem of *Oryza* sp.

*Descriptions and illustrations*: See Wang *et al.* (2019).

*Diagnostic DNA barcodes*: *rpb1*: MK289833; *rpb2*: MK289735; *tef1*: MK289581.  

*hakeae Fusarium* Henn., Verh. Bot. Vereins Prov. Brandenburg 40: 175. 1899.

***Gloeosporium hakeae*** (Henn.) Wollenw., Fusaria Autogr. Delin. 1: 494. 1916.

*Holotypus*: In B *fide*Hein (1988).

*Type locality*: **Germany**, Berlin.

*Type substrate*: *Hakea salicifolia*.

*Note*: Synonym *fide*Wollenweber & Reinking (1935).  

*heidelbergense Fusarium* Sacc., Ann. Mycol. 8: 346. 1910.

(See ***Fusarium culmorum***)

*Holotypus*: In PAD.

*Type locality*: **Germany**, Heidelberg.

*Type substrate*: *Cymbidium* sp.

*Note*: Synonym *fide*Wollenweber & Reinking (1935).  

*helgardnirenbergiae Fusarium* O'Donnell *et al.*, Index Fungorum 440: 2. 2020.

***Neocosmospora nirenbergiana*** Sand.-Den. & Crous, Persoonia 43: 143. 2019.

*Holotypus*: CBS H-23988.

*Ex-type culture*: BBA 65023 = CBS 145469 = G.J.S. 87-127 = NRRL 22387.

*Type locality*: **French Guiana**.

*Type substrate*: Bark of unidentified tree.

*Descriptions and illustrations*: See Sandoval-Denis *et al.* (2019).

*Diagnostic DNA barcodes*: *rpb2*: EU329505; *tef1*: AF178339.  

*helianthi Fusarium* (Schwein.) Wollenw., Fusaria Autogr. Delin. 2: 555. 1924.

*Basionym*: *Vermicularia subeffiguratahelianthi* Schwein., Trans. Amer. Philos. Soc., n.s., 4: 228. 1832.

(See ***Fusarium tricinctum***)

*Holotypus*: PH00078405.

*Type locality*: **Unknown**.

*Type substrate*: *Helianthus annuus*.

*Note*: Synonyms *fide*Wollenweber & Reinking (1935).  

*helotioides Fusarium* Berk. & M.A. Curtis, in Berkeley, Grevillea 3: 98. 1875.

*Holotypus*: ?K(M).

*Type locality*: **USA**, Alabama.

*Type substrate*: *Ilex decidua* (syn. *Ilex prinoides*).

*Notes*: Status unclear. Not *Fusarium fide*Wollenweber & Reinking (1935).  

*hengyangense Fusarium* (Z.Q. Zeng & W.Y. Zhuang) O'Donnell *et al.*, Index Fungorum 440: 2. 2020.

***Neocosmospora hengyangensis*** Z.Q. Zeng & W.Y. Zhuang, Phytotaxa 319: 179. 2017.

*Holotypus*: HMAS 254518.

*Ex-type culture*: HMAS 248884.

*Type locality*: **China**, Hunan, Hengyang, Gouloufeng.

*Type substrate*: Twigs*.*

*Descriptions and illustrations*: See Zeng & Zhuang (2017b).

*Diagnostic DNA barcodes*: *tef1*: KY829448.  

*herbarum Fusarium* (Corda) Fr., Summa Veg. Scand. 2: 472. 1849.

*Basionym*: *Selenosporium herbarum* Corda, Icon. Fung. 3: 34, tab. 6, fig. 88. 1839.

(See ***Fusarium avenaceum***)

*Typus*: PRM 155731.

*Type locality*: **Czech Republic**, Prague.

*Type substrate*: Gramineous plant part.

*Note*: Synonyms *fide*Wollenweber & Reinking (1935). Lectotypification pending study of material lodged in PRM.  

*heteronemum Fusarium* Berk. & Broome (as ‘*heteronema’*), Ann. Mag. Nat. Hist., ser. 3, 15: 402. 1865.

(See *Fusarium candidum* (Link) Sacc.)

*Holotypus*: ?K(M).

*Type locality*: **UK**, Batheaston.

*Type substrate*: Decaying *Pyrus* sp*.*

*Note*: Synonym *fide*Wollenweber & Reinking (1935).  

*heterosporioides Fusarium* Fautrey, in Roumeguère, Fungi Sel. Gall. Exs. No. 5399. 1890 and Rev. Mycol. (Toulouse) 12: 126. 1890.

*Syntype*: ILL00219542 (Roumeguère, Fungi Sel. Gall. Exs no. 5399).

*Type locality*: **France**, Charny

*Type substrate*: *Sclerotium clavus* on *Glyceria fluitans.*

*Notes*: Status unclear. Not *Fusarium fide*Wollenweber & Reinking (1935).  

***heterosporum Fusarium*** Nees & T. Nees, Nova Acta Phys.-Med. Acad. Caes. Leop.-Carol. Nat. Cur. 9: 235. 1818.

*Synonyms*: ?*Fusarium leucoconium* Corda, Icon. Fung. 1: 4. 1837. [*fide* Booth (1971)].

*Sphaeria cyanea* Sollm., Bot. Zeitung (Berlin) 21: 193. 1863.

*Botryosphaeria cyanea* (Sollm.) Weese, Sitzungsber. Kaiserl. Akad. Wiss. Wien, Math.-Naturwiss. Cl., Abt. 1, 128: 707. 1919.

*Gibberella cyanea* (Sollm.) Wollenw., Fusaria Autogr. Delin. 1: 39. 1919.

*Fusarium secalis* Fée, Mém. Soc. Mus. Hist. Nat. Strassbourg 3: 35. 1843.

*Fusarium eleocharidis* Rostr. (as ‘*heleocharidis’*), in Thümen, Mycoth. Univ., Cent. 22, no. 2185. 1883.

*Fusisporium lolii* Wm.G. Sm., Diseases of field and garden crops, chiefly as are caused by fungi: 213. 1884.

*Fusarium lolii* (Wm.G. Sm.) Sacc., Syll. Fung. 11: 652. 1895.

*Fusarium heterosporum* var. *lolii* (Wm.G. Sm.) Wollenw., Z. Parasitenk. (Berlin) 3: 349. 1931.

*Fusarium heterosporum f. paspali* Ellis & Everh., in Ellis, North Amer. Fung., Ser. 2, no. 2395. 1886.

*Fusarium parasiticum* Ellis & Kellerm., J. Mycol. 3: 127. 1887, *nom. illegit.*, Art. 53.1.

*Fusarium pucciniophilum* Sacc. & P. Syd., Syll. Fung. 14: 1128. 1899.

*Fusarium stromaticum* Delacr., Bull. Soc. Mycol. France 9: 186. 1893.

*Fusarium paspalicola* Henn., Monsunia 1: 38. 1899 [1900].

*Fusarium heterosporum* var. *paspalicola* (Henn.) Wollenw., Z. Parasitenk. (Berlin) 3: 349. 1931.

*Fusarium congoense* Wollenw., Fusaria Autogr. Delin. 1: 307. 1916.

*Fusarium heterosporum* var. *congoense* (Wollenw.) Wollenw., Z. Parasitenk. (Berlin) 3: 350. 1931.

*Fusarium heterosporum f*. *aleuritis* Saccas & Drouillon (as ‘*aleuritidis*’), Agron. Trop. 6: 251. 1951.

*Gibberella gordonii* C. Booth, The Genus Fusarium: 177. 1971.

*Lectotypus* (*hic designatus*, MBT 10000692): **Germany**, sclerotium of *Claviceps purpurea* on a spike of *Triticum* sp., 1818, G.C.D. Nees von Esenbeck, in Nova Acta Phys.-Med. Acad. Caes. Leop.-Carol. Nat. Cur., tab. V. fig. 5.

*Epitypus* (*hic designatus*, MBT 10000693): **Germany**, Rotenburg near Bremen, sclerotium of *Claviceps purpurea* on *Lolium perenne*, Aug. 1967, U.G. Schlösser, CBS 391.68 (preserved as metabolically inactive culture).

*Ex-epitype culture*: CBS 391.68 = NRRL 25798.

*Descriptions and illustrations*: See Wollenweber & Reinking (1935), Booth (1971), Gerlach & Nirenberg (1982) and Leslie & Summerell (2006).

*Diagnostic DNA barcodes*: *rpb1*: MW928811; *rpb2*: MW928827; *tef1*: MW928839.

*Notes*: This species is recognised by Wollenweber & Reinking (1935), Gerlach & Nirenberg (1982), Booth (1971), and Leslie & Summerell (2006). Index Fungorum indicates that the correct name for this species is *F. lolii*. However, this name is not commonly used and considered as a synonym of *F. heterosporum*. Additionally, the epithet ‘*heterosporum*’ is older than the epithet ‘*lolii*’ and should have priority. No holotype specimen is available and therefore an illustration is designated as lectotype.  

*heveae Fusarium* Vincens, Bull. Soc. Pathol. Vég. France 2: 19. 1915.

(See ***Fusarium incarnatum***)

*Holotypus*: ?PC.

*Type locality*: **Brazil**, Para.

*Type substrate*: *Hevea brasiliensis*.

*Note*: Synonym *fide*Wollenweber & Reinking (1935).  

***hexaseptatum Fusarium*** Maryani *et al.*, Stud. Mycol. 92: 183. 2018 [2019].

*Holotypus*: InaCC F866 (preserved as metabolically inactive culture).

*Ex-type culture*: InaCC F866.

*Type locality*: **Indonesia**, West Java, Sukabumi, Parakan Lima.

*Type substrate*: *Musa acuminata* var. Pisang Ambon Kuning*.*

*Descriptions and illustrations*: See Maryani *et al.* (2019a).

*Diagnostic DNA barcodes*: *rpb2*: LS479359; *tef1*: LS479805.  

*hibernans Fusarium* Lindau, Rabenh. Krypt.-Fl., ed. 2, 1(9): 542. 1909, *nom. superfl*., Art. 52.1.

*Basionym*: *Fusarium nivale* Ces. ex Berl. & Voglino, in Saccardo, Syll. Fung*.*, Addit. I–IV: 390. 1886, *non* (Fr.) Sorauer, 1901.

(See *Fusarium nivale*)

*Authentic material*: Klotzsch, Herb. Viv. Mycol. no. 1439 in HAL.

*Original locality*: **Italy**, Vercelli.

*Original substrate*: Leaves of overwintered crop.

*Note*: Synonyms *fide*Wollenweber & Reinking (1935).  

*hippocastani Fusarium* (Corda) Sacc., Syll. Fung. 4: 703. 1886.

*Basionym*: *Selenosporium hippocastani* Corda, Icon. Fung. 2: 7. 1838.

(See ***Fusarium acuminatum***)

*Lectotypus* (*hic designatus*, MBT 10000694): **Czech Republic**, Prague, *Aesculus hippocastanum*, 1836, A.C.J. Corda, in Icon. Fung. 2: tab. IX. fig. 31.

*Notes*: According to Pilat (1938) and Holubová-Jechová *et al.* (1994), no material was preserved in PRM. Therefore, an illustration is selected as lectotype.  

***hoodiae Fusarium*** L. Lombard *et al.*, Persoonia 41: 27. 2018 [2019].

*Holotypus*: CBS H-23616.

*Ex-type culture*: CBS 132474.

*Type locality*: **South Africa**, Northern Cape Province, Prieska.

*Type substrate*: Root of *Hoodia gordonii.*

*Descriptions and illustrations*: See Lombard *et al.* (2019b).

*Diagnostic DNA barcodes*: *rpb2*: MH484929; *tef1*: MH485020.  

*hordearium Fusarium* Ducomet, Rech. Dével. Champ. Parasit.: 87. 1907.

*Holotypus*: ?MPA.

*Type locality*: **France**.

*Type substrate*: Unknown.

*Notes*: Status unclear. Not *Fusarium fide*Wollenweber & Reinking (1935).  

*hordei Fusarium* (Wm.G. Sm.) Sacc., Syll. Fung. 11: 652. 1895.

*Basionym*: *Fusisporium hordei* Wm.G. Sm., Diseases of field and garden crops, chiefly as are caused by fungi: 212. 1884.

(See ***Fusarium sambucinum***)

*Lectotypus* (*hic designatus*, MBT 10000695): **Denmark**, *Hordeum* sp., 1884, W. G. Smith, in Diseases of field and garden crops, chiefly as are caused by fungi: 211, fig. 94.

*Notes*: Synonym *fide*Wollenweber & Reinking (1935). No holotype material could be located and therefore an illustration is designated as lectotype.  

***hostae Fusarium*** Geiser & Juba, Mycologia 93: 672. 2001.

*Synonym*: *Gibberella hostae* Geiser & Juba, Mycologia 93: 672. 2001.

*Holotypus*: BPI 748169.

*Ex-type culture*: FRC O-2074 = NRRL 29889.

*Type locality*: **USA**, South Carolina.

*Type substrate*: *Hosta* sp*.*

*Descriptions and illustrations*: See Geiser *et al.* (2001).

*Diagnostic DNA barcodes*: *rpb1*: JX171527; *rpb2*: JX171640; *tef1*: AY329034.  

***humi Fusarium*** (Reinking) Nirenberg & Hagedorn, Nachrichtenbl. Deutsch. Pflanzenschutzdienstes 60: 215. 2008.

*Basionym*: *Fusarium tumidum* var. *humi* Reinking, Zentralbl. Bakteriol., 2. Abth. 89: 513. 1934.

*Lectotypus* (*hic designatus*, MBT 10000706): **Honduras**, soil, 1931, O.A. Reinking, in Wollenweber's Fusaria Autogr. Delin. no. 1152 of type culture 5236.

*Notes*: This species is recognised by Wollenweber & Reinking (1935), Gerlach & Nirenberg (1982), and Nirenberg & Hagedorn (2008). Recollection from the type host and locality is required. No holotype material could be located and therefore an illustration is designated as lectotype.  

***humicola Fusarium*** L. Lombard & Crous, Fungal Syst. Evol. 4: 191. 2019.

*Holotypus*: CBS H-24016.

*Ex-type culture*: ATCC 24372 = CBS 124.73 = IMI 128101 = NRRL 25535.

*Type locality*: **Pakistan**.

*Type substrate*: Soil.

*Descriptions and illustrations*: See Lombard *et al.* (2019a).

*Diagnostic DNA barcodes*: *rpb1*: MN120718; *rpb2*: MN120738; *tef1*: MN120757.  

***humuli Fusarium*** M.M. Wang *et al.*, Persoonia 43: 83. 2019.

*Holotypus*: HAMS 248039.

*Ex-type culture*: CGMCC 3.19374 = CQ1039.

*Type locality*: **China**, Jiangsu Province.

*Type substrate*: Leaves of *Humulus scandens*.

*Descriptions and illustrations*: See Wang *et al.* (2019).

*Diagnostic DNA barcodes*: *rpb1*: MK289840; *rpb2*: MK289724; *tef1*: MK289570.  

*hydnicola Fusarium* Ellis & Everh. (as ‘*hydnicolum*’), J. Mycol. 4(4–5): 45. 1888.

***Alysidium hypophleodes*** (Corda) Bonord., Handb. Mykol.: 35. 1851.

*Basionym*: *Fusidium hypophleodes* Corda, Icon. Fung. 1: 3, tab. 1, fig. 50. 1837.

*Holotypus*: NY (fide Index Fungorum).

*Type locality*: **USA**, Missouri, Concordia.

*Type substrate*: Bark of dead *Hydnum membranaceum*.

*Note*: Synonym *fide*Wollenweber & Reinking (1935).  

*hymenula Fusarium* Pound & Clem., Bot. Surv. Nebraska 4: 7. 1896.

***Gloeosporium intermedium***var.***brevipes*** Sacc., Syll. Fung. 3: 703. 1884.

*Holotypus*: NEB0040541.

*Type locality*: **USA**, Nebraska.

*Type substrate*: *Helianthus* sp.

*Notes*: Synonym *fide*Wollenweber & Reinking (1935). The name is misspelled as ‘*lymenula*’ in the NEB database.  

*hyperoxysporum Fusarium* Wollenw., J. Agric. Res. 2: 268. 1914.

(See ***Fusarium oxysporum***)

*Holotypus*: Not located.

*Type locality*: **USA**.

*Type substrate*: *Ipomoea batatas*.

*Note*: Synonym *fide*Wollenweber & Reinking (1935).  

*hypocreoideum Fusarium* Cooke & Massee, Grevillea 16: 76. 1888.

***Aschersonia hypocreoidea*** (Cooke & Massee) Petch, Ann. Roy. Bot. Gard. (Peradeniya) 7: 255. 1922.

*Holotypus*: K(M) 127920.

*Type locality*: **Australia**, Queensland.

*Type substrate*: *Ficus aspera*.  

*hypodermium Fusarium* (Link) Link, in Willdenow, Sp. Pl., ed. 4, 6: 96. 1825.

*Basionym*: *Fusidium hypodermium* Link, Mag. Neuesten Entdeck. Gesammten Naturk. Ges. Naturf. Freunde Berlin 8: 31. 1816 [1815].

***Marssonina aurantiaca*** (Link) Magnus, Hedwigia 45: 90. 1906.

*Basionym*: *Cryptosporium aurantiacum* Link, in Willdenow, Sp. Pl., ed 4, 6: 96. 1825, *nom. sanct*. (Fries, Syst. Mycol. 3: 481. 1832).

*Synonyms*: *Fusidium aurantiacum* (Link) Fr., Syst. Mycol. 3: 481. 1832.

*Gloeosporium aurantiacum* (Link) Sacc., Syll. Fung. 3: 717. 1884.

*Marssonia aurantiaca* (Link) Rostr., Bot. Tidsskr. 19: 217. 1895.

*Note*: Synonyms *fide*Wollenweber & Reinking (1935).  

*hypothenemi Fusarium* (Sand.-Den. & Crous) O'Donnell *et al.*, Index Fungorum 440: 2. 2020.

***Neocosmospora hypothenemi*** Sand.-Den. & Crous, Persoonia 43: 132. 2019.

*Holotypus*: CBS H-23982.

*Ex-type culture*: ARSEF 5878 = CBS 145464 = NRRL 52782.

*Type locality*: **Benin**, Niaouli.

*Type substrate*: Adult *Hypothenemus hampei* (coffee borer beetle).

*Descriptions and illustrations*: See Sandoval-Denis *et al.* (2019).

*Diagnostic DNA barcodes*: *rpb1*: MW218117; *rpb2*: JF741176; *tef1*: JF740850.  

*idahoanum Fusarium* O.A. Pratt, J. Agric. Res. 13: 86. 1918.

(See ***Fusarium flocciferum***)

*Lectotypus* (*hic designatus*, MBT 10000707): **USA**, Idaho, soil, 1918, O.A. Pratt, in J. Agric. Res. 13: 87, fig. 2.

*Notes*: Synonyms *fide*Wollenweber & Reinking (1935). No holotype material could be located and therefore an illustration is designated as lectotype.  

*illosporioides Fusarium* Sacc., Harriman Alaska Exped. 5: 15. 1904.

(See ***Fusarium lateritium***)

*Holotypus*: In PAD.

*Type locality*: **USA**, Alaska, Sitka.

*Type substrate*: *Ribes* sp.

*Note*: Synonym *fide*Wollenweber & Reinking (1935).  

*illudens Fusarium* C. Booth, The Genus Fusarium: 54. 1971.

***Neocosmospora illudens*** (Berk.) L. Lombard & Crous, Stud. Mycol. 80: 227. 2015.

*Basionym*: *Nectria illudens* Berk., in Hooker, Bot. Antarct. Voy. II (Fl. Nov.-Zel.): 203. 1855.

*Synonyms*: *Cucurbitaria illudens* (Berk.) Kuntze, Revis. Gen. Pl. 3: 461. 1898.

*Haematonectria illudens* (Berk.) Samuels & Nirenberg, Stud. Mycol. 42: 136. 1999.

*Neotypus*: PAD S00012, designated in Forin *et al.* (2020)

*Neotype locality*: **New Zealand**.

*Neotype substrate*: Bark of unknown host plant,  

“*inaequale Fusarium*” Auersw. Bot. Zeitung (Berlin) 8: 439. 1850, typographic error (see Notes).

***Ramularia rosea*** (Fuckel) Sacc., Fungi Ital. Del., Tab. 1001. 1881.

*Basionym*: *Fusidium roseum* Fuckel, Fungi Rhen. Fasc. III, no. 219. 1863.

*Synonyms*: *Ovularia rosea* (Fuckel) Massee, Brit. Fung.-Fl. 3: 323. 1893.

*Cylindrospora rosea* (Fuckel) J. Schröt., in Cohn, Krypt.-Fl. Schles., Pilze II: 493. 1897.

*Fusidium inaequale* Auersw., in Rabenh., Klotzschii Herb. Viv. Mycol., Cent. 14: no. 1383. 1850.

*Ramularia lucidae* Davis, Trans. Wis. Acad. Sci. Art. Lett. 19: 687. 1919.

*Authentic material*: Rabenh., Klotzschii Herb. Viv. Mycol. 1383 in HAL.

*Original locality*: **Germany**, Leipzig.

*Original substrate*: *Salix amygdalina*.

*Notes*: Not *Fusarium fide*Wollenweber & Reinking (1935). This species was first published as *Fusidium inaequale* Auersw., in Rabenh., Klotzschii Herb. Viv. Mycol., Cent. 14: no. 1383, 1850. The description was repeated in Bot. Zeitung 8: 439, 1850 and Flora 33: 283, 1850 (in the latter publication also under *Fusidium*), so that in the simultaneous publication in “Botanische Zeitung” the “F.” was undoubtedly also meant to be *Fusidium* and not *Fusarium*. Syntype material deposited at HAL has recently been examined, and *Fusidium inaequale* turned out to be a heterotypic synonym of *Ramularia rosea* (Fuckel) Sacc (see Braun 1998).  

*incarcerans Fusarium* (Berk.) Sacc., Syll. Fung. 4: 713. 1886.

*Basionym*: *Fusisporium incarcerans* Berk., Intellectual Observ. 2: 11. 1863.

(See ***Fusarium avenaceum***)

*Holotypus*: ?K(M).

*Type locality*: **UK**, Northamptonshire, Fotheringhay Castle.

*Type substrate*: *Orthotrichum* sp.

*Note*: Synonym *fide*Wollenweber & Reinking (1935).  

***incarnatum Fusarium*** (Roberge ex Desm.) Sacc., Syll. Fung. 4: 712. 1886.

*Basionym*: *Fusisporium incarnatum* Roberge ex Desm., Ann. Sci. Nat., Bot., sér. 3, 11: 274. 1849.

*Synonyms*: *Fusarium semitectum* Berk. & Ravenel, Grevillea 3: 98. 1875.

*Pseudofusarium semitectum* (Berk. & Ravenel) Matsush., Icon. Microfung. Matsush. Lect. (Kobe): 119. 1975.

*Fusarium pallens* Berk. & M.A. Curtis, Grevillea 3: 99. 1875, *nom. illegit.*, Art. 53.1, *non Fusarium pallens* Nees & T. Nees 1818.

*Fusarium glumarum* Sacc., Syll. Fung. 4: 706. 1886 (*nom. nov*. for *F. pallens* Berk. & M.A. Curtis).

*Fusisporium pallidoroseum* Cooke, Grevillea 6: 139. 1878.

*Fusarium pallidoroseum* (Cooke) Sacc., Syll. Fung. 4: 720. 1886.

*Fusarium asparagi* Briard, Rev. Mycol. (Toulouse) 12: 142. 1890.

*Fusarium gloeosporioides* Speg. (as ‘*gloeosporioide*’), Anales Mus. Nac. Hist. Nat. Buenos Aires 6: 350. 1899.

*Fusarium juglandinum* Peck, Bull. Torrey Bot. Club 36: 157. 1909.

*Fusarium heveae* Vincens, Bull. Soc. Pathol. Vég. France 2: 19. 1915.

*Fusarium tenuistipes* Sacc., Atti Mem. Reale Accad. Sci. Lett. Arti, Padova 33: 195. 1917.

*Fusarium semitectum* var. *majus* Wollenw., Fusaria Autogr. Delin. 3: 907–910. 1930.

*Fusarium semitectum* var. *violaceum* Batikyan & Abramyan (as ‘*violaceae*’), Biol. Zhurn. Armenii 22: 58. 1969, *nom. inval*., Art. 39.1.

*Lectotypus*: (*hic designatus*, MBT 10001327) **France**, from *Tagetes erecta*, 1848, M. Roberge in Desmazières, Pl. Crypt. N. France, éd 2, No. 1303, in PC.

*Epitypus*: (*hic designatus*, MBT 10001328) **Malawi**, on *Trichosanthes dioica,* date unknown, H.M. Phiri, CBS H-24060.

*Ex-epitype culture*: ATCC 24387 = CBS 132.73 = IMI 128222 = NRRL 25478.

*Descriptions and illustrations*: See Booth (1971), Gerlach & Nirenberg (1982) and Xia *et al.* (2019).

*Diagnostic DNA barcodes*: *rpb2*: MN170409; *tef1*: MN170476.

*Note*: The epitypification of *Fusarium incarnatum* by Xia *et al.* (2019) was not effective as the holo- or lectotype was not correctly indicated (Art. 9.9). Here, a lectotype is selected and the epitypification is validated.  

***inflexum Fusarium*** R. Schneid., in Schneider & Dalchow, Phytopathol. Z. 82: 80. 1975.

*Holotypus*: DSM 63203.

*Ex-type culture*: ATCC 32213 = BBA 63203 = CBS 716.74 = DAOM 225130 = DSM 63203 = IMI 375336 = NRRL 20433.

*Type locality*: **Germany**, Hamburg, Vierlanden.

*Type substrate*: Stem of *Vicia faba.*

*Descriptions and illustrations*: See Schneider & Dalchow (1975) and Gerlach & Nirenberg (1982).

*Diagnostic DNA barcodes*: *rpb1*: JX171469; *rpb2*: JX171583; *tef1*: AF008479.  

*inseptatum Fusarium* Schwein., Trans. Amer. Philos. Soc., n.s., 4: 302. 1832 [1834].

*Holotypus*: PH00062493.

*Type locality*: **USA**, Pennsylvania, Bethlehem.

*Type substrate*: *Daphne mezereum*.

*Notes*: Status unclear. Not *Fusarium fide*Wollenweber & Reinking (1935).  

*insidiosum Fusarium* Roum., Michelia 2: 132. 1880.

(See ***Fusarium lateritium***)

*Syntypes*: In BR, CUP & ILL (Roum., Fungi Sel. Gall. Exs. No. 57).

*Type locality*: **France**, Pyrénées-Orientales, Environs de Perpignan.

*Type substrate*: *Phytolacca decandra.*

*Note*: Synonym *fide*Wollenweber & Reinking (1935).  

*insidiosum Fusarium* (Berk.) Sacc., Syll. Fung. 4: 707. 1886, *nom. illegit.*, Art. 53.1.

*Basionym*: *Fusisporium insidiosum* Berk., Gard. Chron. 1860: 480. 1860.

(See ***Fusarium graminearum***)

*Holotypus*: ?K(M).

*Type locality*: **UK**.

*Type substrate*: *Agrostis pulchella*.

*Note*: Synonyms *fide*Wollenweber & Reinking (1935).  

***ipomoeae Fusarium*** M.M. Wang *et al.*, Persoonia 43: 83. 2019.

*Holotypus*: HAMS 248040.

*Ex-type culture*: CGMCC 3. 19496 = LC12165.

*Type locality*: **China**, Jiangsu Province.

*Type substrate*: Leaves of *Ipomoea aquatica*.

*Descriptions and illustrations*: See Wang *et al.* (2019).

*Diagnostic DNA barcodes*: *rpb1*: MK289859; *rpb2*: MK289752; *tef1*: MK289599.  

***iranicum Fusarium*** Torbati *et al.*, Mycol. Progr. 18: 129. 2018 [2019].

*Holotypus*: CBS H-23560.

*Ex-type culture*: CBS 143608 = CPC 30860.

*Type locality*: **Iran**, West Azerbaijan Province, Orumieh-Salmas.

*Type substrate*: *Agaricus bisporus*.

*Descriptions and illustrations*: See Torbati *et al.* (2019).

*Diagnostic DNA barcodes*: *rpb2*: LT970757; *tef1*: LT970785.  

*iridis Fusarium* Oudem., Ned. Kruidk. Arch., 2 sér. 5: 515. 1889.

(See ***Fusarium avenaceum***)

*Holotypus*: ?L.

*Type locality*: **Netherlands**.

*Type substrate*: *Iris pseudacorus*.

*Note*: Synonym *fide*Wollenweber & Reinking (1935).  

***irregulare Fusarium*** M.M. Wang *et al.*, Persoonia 43: 84. 2019.

*Holotypus*: HAMS 248041.

*Ex-type culture*: CGMCC 3.19489 = LC7188.

*Type locality*: **China**, Guangdong Province.

*Type substrate*: *Bambusoideae*.

*Descriptions and illustrations*: See Wang *et al.* (2019).

*Diagnostic DNA barcodes*: *rpb1*: MK289863; *rpb2*: MK289783; *tef1*: MK289629.  

*japonicum Fusarium* Allesch., Beibl. Hedwigia 36: (164). 1897.

(See *Fusarium tortuosum*)

*Syntype*: S-F45631 (Sydow, Mycoth. March. no. 4592).

*Type locality*: **Germany**, Berlin.

*Type locality*: *Prunus japonica.*

*Note*: Synonym *fide*Wollenweber & Reinking (1935).  

*javanicum Fusarium* Koord., Verh. Kon. Akad. Wetensch., Afd. Natuurk., Sect. 2, 13: 247. 1907.

*Holotypus*: Not located.

*Type locality*: **Indonesia**, Central Java, Purworejo.

*Type substrate*: *Ficus elastica*.

*Note*: Status unclear *fide*Sandoval-Denis *et al.* (2019).  

*juglandinum Fusarium* Peck, Bull. Torrey Bot. Club 36: 157. 1909.

(See ***Fusarium incarnatum***)

*Holotypus*: NYSf1607.

*Type locality*: **USA**, Kansas, Rooks, Stockton.

*Type substrate*: *Juglans nigra*.

*Note*: Synonym *fide*Wollenweber & Reinking (1935).  

*junci Fusarium* P. Crouan & H. Crouan, Fl. Finistère: 14. 1867.

*Holotypus*: ?CO.

*Type locality*: **France**, Paris.

*Type substrate*: *Juncus effusus*.

*Note*: ?*Fusidium fide*Wollenweber & Reinking (1935).  

*jungiae Fusarium* Pat., Bull. Soc. Mycol. France 11: 234. 1895.

(See ***Fusarium avenaceum***)

*Holotypus*: FH00965356.

*Type locality*: **Argentina**, San Jorge.

*Type substrate*: Parasitic on *Puccinia* sp. on *Jungia* sp.

*Note*: Synonym *fide*Wollenweber & Reinking (1935).  

*juruanum Fusarium* Henn., Hedwigia 43: 398. 1904.

(See *Fusarium coccidicola*)

*Holotypus*: In B.

*Type locality*: **Brazil**, Rio Jurua.

*Type substrate*: *Annonaceae* sp.

*Note*: Synonym *fide*Gerlach & Nirenberg (1982).  

***kalimantanense Fusarium*** Maryani *et al.*, Stud. Mycol. 92: 187. 2018 [2019].

*Holotypus*: InaCC F917 (preserved as metabolically inactive culture).

*Ex-type culture*: InaCC F917.

*Type locality*: **Indonesia**, Central Kalimantan, Katingan, Pulau Malan.

*Type substrate*: *Musa acuminata* var. Pisang Ambon*.*

*Descriptions and illustrations*: See Maryani *et al.* (2019a).

*Diagnostic DNA barcodes*: *rpb1*: LS479497; *rpb2*: LS479241; *tef1*: LS479690.  

*kelerajum Fusarium* Samuels *et al.*, Mycologia 103: 1326. 2011.

***Neocosmospora keleraja*** Samuels *et al.*, Mycologia 103: 1326. 2011.

*Holotypus*: BPI 871413.

*Ex-type culture*: FRC S-1839 = G.J.S. 02-122.

*Type locality*: **Sri Lanka**, Minneriya Natl. Forest.

*Type substrate*: Trunk of *Yakuda marang.*

*Descriptions and illustrations*: See Nalim *et al.* (2011).

*Diagnostic DNA barcode*: *tef1*: DQ247518.  

*keratoplasticum Fusarium* Geiser *et al.*, Fung. Gen. Biol. 53: 68. 2013.

***Neocosmospora keratoplastica*** (Geiser *et al.*) Sand.-Den. & Crous, Persoonia 41: 120. 2018.

*Synonyms*: *Cephalosporium keratoplasticum* T. Morik., Mycopathologia 2: 66. 1939, *nom. inval.*, Art. 39.1.

*Hyalopus keratoplasticum* T. Morik. ex M.A.J. Barbosa, Subsidios Para o Estudo Parasitologico do Genero Hyalopus Corda, 1838: 19. 1941, *nom. inval*., Art. 39.1.

*Fusarium sedimenticola* M.M. Wang *et al.*, Botanica Marina 63: 174. 2020.

*Holotypus*: FRC S-2477.

*Ex-type culture*: CBS 490.63 = FRC S-2477 = NRRL 22661.

*Type locality*: **USA**, Virginia, Winchester.

*Type substrate*: Indoor plumbing*.*

*Descriptions and illustrations*: See Nalim *et al.* (2011).

*Diagnostic DNA barcodes*: *rpb1*: MW218121; *rpb2*: JN235897; *tef1*: JN235712.  

***konzum Fusarium*** Zeller *et al.*, Mycologia 95: 947. 2003.

*Synonym*: *Gibberella konza* Zeller *et al.*, Mycologia 95: 947. 2003.

*Holotypus*: DAR 76034.

*Ex-type culture*: CBS 119849 = KSU 10653 = NRRL 53394.

*Type locality*: **USA**, Kansas, Manhattan, Konza Praire Biological Station.

*Type substrate*: *Sorghastrum nutans.*

*Descriptions and illustrations*: See Zeller *et al.* (2003) and Leslie & Summerell (2006).

*Diagnostic DNA barcodes*: *rpb1*: LT996200; *rpb2*: LT996148; *tef1*: LT996098.  

***kotabaruense Fusarium*** Maryani *et al.*, Persoonia 43: 65. 2019.

*Holotypus*: InaCC F963 (preserved as metabolically inactive culture).

*Ex-type culture*: InaCC F963.

*Type locality*: **Indonesia**, South Kalimantan, Kota Baru, Kecamatan Pamukan Barat, Desa Sungai Birah.

*Type substrate*: *Musa* var. Pisang Hawa*.*

*Descriptions and illustrations*: See Maryani *et al.* (2019b).

*Diagnostic DNA barcodes*: *rpb1*: LS479875; *rpb2*: LS479859; *tef1*: LS479445.  

*kuehnii Fusarium* (Fuckel) Sacc., Syll. Fung. 4: 714. 1886.

*Basionym*: *Fusisporium kuehnii* Fuckel, Fungi Rhen. Exs., Suppl., Fasc. 5, no. 1920. 1867.

?***Athelia arachnoidea*** (Berk.) Jülich, Willdenowia 7: 53. 1972. (*fide* Gerlach & Nirenberg 1982)

*Basionym*: *Corticium arachnoideum* Berk., Ann. Mag. Nat. Hist., ser. 1, 13: 345. 1844.

*Synonym*: *Fusisporium devastans* J.G. Kühn, Krankh. Kulturgew.: 32. 1858, *nom. inval*., Art. 38.1(a).

*Syntype*: Fuckel, Fungi Rhen. Exs., Suppl., Fasc. 5, 1920 (*e.g*., HAL).

*Type locality*: **Germany**.

*Type substrate*: Lichens and mosses.

*Notes*: Status doubtful. Considered a possible synonym of *F. dimerum* by Booth (1971).  

*kurdicum Fusarium* Petr., Sydowia 13: 96. 1959.

***Cosmospora kurdica*** (Petr.) Rossman & Samuels, Stud. Mycol. 42: 122. 1999.

*Basionym*: *Calonectria kurdica* Petr., Sydowia 13: 95. 1959.

*Synonyms*: *Nectria kurdica* (Petr.) Rossman, Mycol. Pap. 150: 35. 1983.

?*Stagonopsis sclerotioides* Höhn., Ann. K. K. Naturhist. Hofmus. 20: 368. 1905.

?*Botryocrea sclerotioides* (Höhn.) Petr., Sydowia 3: 141. 1949.

?*Fusarium sclerotioides* (Höhn.) Samuels & Rossman, Mycol. Pap. 164: 23. 1991.

*Holotypus*: K.H. Rechinger, 31 Jul. 1957, in W.

*Type locality*: **Iran**, Kurdistan.

*Type substrate*: *Astragalus* sp.

*Note*: Synonyms *fide*Rossman *et al.* (1999).  

*kuroshium Fusarium* F. Na *et al.*, Plant Disease 102: 1159. 2018, *nom. inval*., Art. 40.7.

***Neocosmospora kuroshio*** F. Na *et al.* ex Sand.-Den. & Crous, Persoonia 43: 137. 2019.

*Holotypus*: BPI 910340.

*Ex-type culture*: CBS 142642 = UCR 3641.

*Type locality*: **USA**, California, San Diego, El Cajon.

*Type substrate*: *Euwallacea* sp. galleries in *Platanus racemosa.*

*Descriptions and illustrations*: See Na *et al.* (2018).

*Diagnostic DNA barcodes*: *rpb1*: KX262236; *rpb2*: KX262256; *tef1*: KX262216.  

*kurunegalense Fusarium* Samuels *et al.*, Mycologia 103: 1323. 2011.

***Neocosmospora kurunegalensis*** Samuels *et al.*, Mycologia 103: 1324. 2011.

*Holotypus*: BPI 871391.

*Ex-type culture*: CBS 119599 = G.J.S. 02-94.

*Type locality*: **Sri Lanka**, Wagamba Province, Kurunegala.

*Type substrate*: Recently felled tree*.*

*Descriptions and illustrations*: See Nalim *et al.* (2011).

*Diagnostic DNA barcodes*: *rpb1*: MW834228; *rpb2*: LR583838; *tef1*: DQ247511.  

***kyushuense Fusarium*** O'Donnell & T. Aoki, Mycoscience 39: 2. 1998.

*Holotypus*: NIAES99701.

*Ex-type culture*: ATCC 56750 = FRC T-346A = MAFF 237645 = MRC 1767 = NRRL 3509.

*Type locality*: **Japan**, Kumamoto.

*Type substrate*: Seed of *Triticum aestivum.*

*Descriptions and illustrations*: See Aoki & O'Donnell (1998).

*Diagnostic DNA barcodes*: *rpb2*: MH582098*; tef1*: MH582292.  

*laboulbeniae Fusarium* Cépède, Arch. Parasitol. 16: 373. 1914.

(See *Fusarium larvarum*)

*Holotypus*: Not located.

*Type locality*: **France**, Pas-de-Calais, Wimereux.

*Type substrate*: *Demetrias unipunctata*.

*Note*: Synonym *fide*Wollenweber & Reinking (1935).  

***lacertarum Fusarium*** Subrahm. (as ‘*laceratum*’), Mykosen 26: 478. 1983.

*Holotypus*: IMI 300797.

*Ex-type culture*: ATCC 42771 = CBS 130185 = IMI 300797 = NRRL 20423.

*Type locality*: **India**, Poona, Pimpri.

*Type substrate*: Skin of lizard*.*

*Descriptions and illustrations*: See Subrahmanyam (1983).

*Diagnostic DNA barcodes*: *rpb1*: JX171467; *rpb2*: JX171581; *tef1*: GQ505593.  

***lactis Fusarium*** Pirotta, Arch. Lab. Bot. Crittog. Univ. Pavia 2 & 3: 316. 1879.

*Synonyms*: ?*Fusarium pyrinum* Schwein., Trans. Amer. Philos. Soc., n.s. 4: 302. 1834.

?*Fusarium apiogenum* Sacc., Syll. Fung. 4: 717. 1886.

*Fusarium rubrum* Parav., Ann. Mycol. 16: 311. 1918.

*Lectotypus*: Arch. Lab. Bot. Crittog. Univ. Pavia 2 & 3, Tab. 21, figs 1–6, designated by Yilmaz *et al.* (2021).

*Lectotype locality*: **Italy**, Pavia.

*Lectotype substrate*: Clotted milk.

*Epitypus*: B 70 0001686, designated by Yilmaz *et al.* (2021).

*Ex-epitype culture*: BBA 68590 = CBS 411.97 = IMI 375351 = NRRL 25200.

*Epitype locality*: **USA**, California.

*Epitype substrate*: *Ficus carica*.

*Descriptions and illustrations*: See Nirenberg & O'Donnell (1998) and Leslie & Summerell (2006).

*Diagnostic DNA barcodes*: *rpb1*: LT996201; *rpb2*: LT996149; *tef1*: AF160272.  

*lagenariae Fusarium* (Schwein.) Sacc., Syll. Fung. 4: 724. 1886.

*Basionym*: *Fusisporium lagenariae* Schwein., Trans. Amer. Philos. Soc., n.s., 4: 275. 1834.

(See ***Fusarium oxysporum***)

*Holotypus*: PH00062516

*Type locality*: **USA**, Pennsylvania, Bethlehem.

*Type substrate*: *Lagenaria siceraria*.

*lagenarium Fusarium* Pass., Erb. Critt. Ital., ser. 2: no. 148. 1871.

*Synonym*: *Gloeosporium lagenarium* (Pass.) Sacc. & Roum., Rev. Mycol., Toulouse 2(8): 201. 1880.

(See *Fusarium cyclogenum*)

*Holotypus*: In PAD.

*Type locality*: **Italy**, Parma.

*Type substrate*: *Lagenaria* sp.

*Note*: Synonym *fide*Wollenweber & Reinking (1935).  

*lanceolatum Fusarium* O.A. Pratt, J. Agric. Res. 13: 83. 1918.

(See ***Fusarium acuminatum***)

*Lectotypus* (*hic designatus*, MBT 10000709): **USA**, Idaho, from soil, 1918, O.A. Pratt, in J. Agric. Res. 13: 82, fig. 1A–E.

*Notes*: Synonym *fide*Wollenweber & Reinking (1935). No holotype specimen could be located and therefore an illustration is designated as lectotype.  

***langsethiae Fusarium*** Torp & Nirenberg, Int. J. Food Microbiol*.* 95: 248. 2004.

*Holotypus*: B 70 0012234.

*Ex-type culture*: BBA 70945 = CBS 113234.

*Type locality*: **Norway**.

*Type substrate*: Kernal of *Avena sativa.*

*Descriptions and illustrations*: See Torp & Nirenberg (2004).

*Diagnostic DNA barcodes*: *rpb1*: MW928812; *rpb2*: MW928828; *tef1*: AB674298.  

***languescens Fusarium*** L. Lombard & Crous, Persoonia 43: 28. 2018 [2019].

*Holotypus*: CBS H-23617.

*Ex-type culture*: CBS 645.78 = NRRL 36531.

*Type locality*: **Morocco**.

*Type substrate*: *Solanum lycopersicum.*

*Descriptions and illustrations*: See Lombard *et al.* (2019b).

*Diagnostic DNA barcodes*: *rpb1*: MW928813; *rpb2*: MH484880; *tef1*: MH484971.  

***laricis Fusarium*** Sawada, Bull. Gov. Forest Exp. Sta., Meguro 46: 130. 1950.

*Holotypus*: TFM:FPH 00771.

*Type locality*: **Japan**, Aomori, Kamikita, Noheji

*Type substrate*: *Larix kaempferi*.  

*larvarum Fusarium* Fuckel, Jahrb. Nassauischen Vereins Naturk. 23–24: 369. 1870.

***Microcera larvarum*** (Fuckel) Gräfenhan *et al.*, Stud. Mycol. 68: 105. 2011.

*Synonyms*: *Fusarium nivale* var. *larvarum* (Fuckel) Bilaĭ, Fusarii (Biologija i sistematika): 295. 1955, *nom. inval*., Art. 41.1

*Fusarium cryptum* McAlpine, *Fungus Diseases of* Citrus *trees in Australia*: 106. 1899.

*Fusarium epicoccum* McAlpine, *Fungus Diseases of* Citrus *trees in Australia*: 113. 1899.

*Microcera parlatoriae* Trab., Bull. Agric. Algérie Tunisie 13: 33. 1907.

*Microcera curta* Sacc., Ann. Mycol. 7: 437. 1909.

*Microcera tonduzii* Pat., Bull. Soc. Mycol. France 28: 142. 1912.

*Fusarium aspidioti* Sawada, Bot. Mag. (Tokyo) 28: 312. 1914.

*Fusarium laboulbeniae* Cépède, Arch. Parasitol. 16: 373. 1914.

*Fusarium acremoniopsis* Vincens, Bull. Soc. Mycol. France 31: 26. 1915.

?*Fusarium meliolicola* F. Stevens, Bot. Gaz. 65: 245. 1918.

?*Nectria meliolicola* F. Stevens, Bot. Gaz. 65: 231. 1918.

*Microcera aurantiicola* Petch, Trans. Brit. Mycol. Soc. 7: 158. 1921.

*Lectotypus*: G 00111015, selected in Gräfenhan *et al.* (2011).

*Lectotype locality*: **Germany**, Hessen, Rheingau, near Oestrich-Winkel.

*Lectotype substrate*: Larva cuticles of insects on *Malus domestica*.

*Epitypus*: BBA 62239, designated in Gräfenhan *et al.* (2011).

*Ex-epitype culture*: BBA 62239 = CBS 738.79 = MUCL 19033 = NRRL 20473.

*Epitype locality*: **Iran**, Gilan Province, near Rasht.

*Epitype substrate*: Parasitic on *Quadraspidiotus perniciosus* (scale) on *Prunus domestica.*

*Diagnostic DNA barcodes*: *rpb1*: KM232252; *rpb2*: KM232387; *tef1*: KM231957.  

***lateritium Fusarium*** Nees, Syst. Pilze: 31. 1817.

*Synonyms*: *Selenosporium lateritium* (Nees) Desm., Fl. Cryptog. Flandres 2: 99. 1867.

*Fusarium microsporum* Schltdl., Fl. Berol. 2: 139. 1824.

*Fusarium fructigenum* Fr., Syst. Mycol. 3: 471. 1832.

*Fusarium lateritium* var. *fructigenum* (Fr.) Wollenw., Fusaria Autogr. Delin. 3: 959. 1930.

*Sphaeria baccata* Wallroth, Fl. Crypt. Germ. 2: 838. 1833.

*Gibbera baccata* (Wallr.) Fuckel, Jahrb. Nassauischen Vereins Naturk. 23–24: 167. 1870.

*Gibberella pulicaris* subsp. *baccata* (Wallr.) Sacc., Michelia 1 (3): 317. 1878.

*Gibberella baccata* (Wallr.) Sacc., Syll. Fung. 2: 553. 1883.

*Fusarium lateritium* var. *mori* Desm., Ann. Sci. Nat. Bot., ser. 2, 8: 10. 1837.

*Selenosporium urticarum* Corda (as ‘*urticearum’*), Icon. Fung. 2: 7. 1838.

*Fusarium urticarum* (Corda) Sacc., Syll. Fung. 4: 698. 1886.

?*Fusarium protractum* Lév., Ann. Sci. Nat., Bot., sér. 3, 9: 246. 1848.

*Gloeosporium berkeleyi* Mont., Ann. Sci. Nat., Bot., sér. 3, 12: 296. 1849.

*Fusarium berkeleyi* (Mont.) Berk. & Broome, N. Amer. Fung.: 108. 1875.

*Botryosphaeria moricola* Ces. & De Not., Hedwigia 4: 27. 1865.

*Gibberella moricola* (Ces. & De Not.) Sacc., Syll. Fung. 2: 553. 1883.

*Gibbera euonymi* Fuckel, Jahrb. Nassauischen Vereins Naturk. 23–24: 167. 1870.

*Gibberella euonymi* (Fuckel) Sacc., Michelia 1: 318. 1878.

*Hendersonia euonymi* (Fuckel) Sacc., Syll. Fung. 2: 556. 1883.

*Selenosporium cydoniae* Schulzer, Verh. K.K. Zool.-Bot. Ges. Wien 21: 1240. 1871.

*Fusarium cydoniae* (Schulzer) Sacc. & Traverso, Syll. Fung. 19: 724. 1910, *nom. illegit.*, Art. 53.1.

*Fusarium sticticum* Berk. & M.A. Curtis, Grevillea 3: 99. 1875.

*Fusisporium zavianum* Sacc., Michelia 1: 83. 1877.

*Fusarium zavianum* (Sacc.) Sacc., Syll. Fung. 4: 709. 1886.

*Fusarium cydoniae* Roum. & Fautrey, Rev. Mycol. (Toulouse) 14: 170. 1892, *nom. illegit.*, Art. 53.1.

*Fusarium salicis* Fuckel, Fungi Rhen. Exs., Suppl., Fasc. 7, no. 2110. 1868.

*Fusarium salicis* var. *minus* Wollenw., Fusaria Autogr. Delin. 2: 582. 1924.

*Fusarium sambucinum* var. *minus* Wollenw., Fusaria Autogr. Delin. 3: 941. 1930.

*Gibbera mori* Fuckel, Jahrb. Nassauischen Vereins Naturk. 23–24: 168. 1870.

*Fusarium semitectum* Berk. & Ravenel, in Berkeley, Grevillea 3: 98. 1875.

*Fusisporium cinnabarinum* Berk. & M.A. Curtis, Grevillea 3: 146. 1875.

*Fusarium cinnabarinum* (Berk. & M.A. Curtis) Sacc., Syll. Fung. 4: 722. 1886.

*Fusisporium miniatum* Berk. & M.A. Curtis, Grevillea 3: 147. 1875.

*Fusarium miniatum* (Berk. & M.A. Curtis) Sacc., Syll. Fung. 4: 722. 1886, *nom. illegit.*, Art. 53.1.

*Fusisporium putaminum* Thüm., Oesterr. Bot. Z. 27: 272. 1877.

*Fusarium putaminum* (Thüm.) Sacc., Syll. Fung. 4: 703. 1886.

*Fusisporium leguminum* Cooke, Grevillea 6: 139. 1878.

*Fusarium leguminum* (Cooke) Sacc., Syll. Fung. 4: 712. 1886.

*Fusarium limonis* Briosi, Att. Staz. Chim. Agrar. Rome. 1878.

*Fusarium yuccae* Cooke, Grevillea 7: 34. 1878, *nom. inval*., Art. 36.1(a).

*Fusisporium azedarachinum* Thüm., Mycoth. Univ., cent. 14: no. 1379. 1879.

*Fusarium azedarachinum* (Thüm.) Sacc., Syll. Fung. 4: 704. 1886.

*Fusarium insidiosum* Roum., Michelia 2: 132. 1880.

*Fusarium roumeguerei* Sacc. (as ‘*roumegueri*’), Syll. Fung. 4: 702. 1886, *nom. illegit.*, Art. 52.1.

*Fusarium albertii* Roum., Fungi Sel. Gall. Exs., Cent. 19: no. 1867. 1881.

*Fusarium rimicola* Sacc. (as ‘*rimicolum*’), Michelia 2: 297. 1881.

*Fusarium ziziphinum* Pass., Rev. Mycol., (Toulouse) 4: 22. 1882.

*Fusarium acaciae* Cooke & Harkn., Grevillea 12: 96. 1884.

*Fusarium longisporum* Cooke & Massee, Grevillea 16: 4. 1887.

*Fusarium sphaeroideum* Pass., Atti Reale Accad. Lincei, Rendiconti Cl. Sci. Fis., sér. 4, 4: 105. 1888.

*Fusarium parasiticum* Fautrey, Rev. Mycol. (Toulouse) 11: 153. 1889, *nom. illegit.*, Art. 53.1.

*Fusarium fautreyi* Sacc., Syll. Fung. 10: 934. 1892.

*Fusarium carneoroseum* Cooke, Grevillea 19: 4. 1890.

*Fusarium celtidis* Ellis & Tracy, J. Mycol. 6: 76. 1890.

*Fusarium nucicola* P. Karst. & Har., Rev. Mycol. (Toulouse) 12: 131. 1890.

*Fusarium discoideum* Fautrey & Roum., Rev. Mycol. (Toulouse) 13: 173. 1891.

*Fusarium cydoniae* Allesch., Ber. Bot. Vereines Landshut 12: 130. 1892.

?*Fusarium luteum* Clem., Bot. Surv. Nebraska 3: 12. 1894.

*Fusarium asclepiadeum* Fautrey, Rev. Mycol. (Toulouse) 18: 68. 1896.

*Fusarium samararum* Allesch., Ber. Bayer. Bot. Ges. 4: 39. 1896.

*Fusarium sophorae* Allesch., Beibl. Hedwigia 36: (164). 1897.

*Fusarium ailanthinum* Speg., Anales Mus. Nac. Hist. Nat. Buenos Aires 6: 350. 1899.

*Fusarium euonymi* Syd., Beibl. Hedwigia 39: (6). 1900.

*Fusarium gemmiperda* Aderh., Z. Pflanzenkrankh. 11: 70. 1901.

*Fusarium euonymi-japonici* Henn., Hedwigia 41: 139. 1902.

*Fusarium illosporioides* Sacc., in Saccardo *et al.*, Harriman Alaska Expedition 5: 15. 1904.

*Fusarium schawrowi* Speschnew, Arbeiten Kaukas. Stat. Seidenzucht 10: 30–41. 1906.

*Selenosporium gloeosporioides* Speg. (as ‘*gloesporioides*’), Anales Mus. Nac. Hist. Nat. Buenos Aires 13: 458. 1911.

*Fusarium gloeosporioides* (Speg.) Sacc. & Trotter, Syll. Fung. 22: 1482. 1913, *nom. illegit.*, Art. 53.1.

*Fusarium briosianum* Ferraris, Fl. Ital. Crypt. Fungi Fasc. 13: 857. 1912.

*Fusarium pseudacaciae* Rapaics, Z. Pflanzenkrankh. 25: 208. 1915.

*Fusarium gleditschiicola* Dearn. & Barthol. (as ‘*gleditschicola*’), Mycologia 9: 363. 1917.

*Gibberella briosiana* Turconi & Maffei, Atti Ist. Bot. Univ. Pavia, sér. 2, 15: 148. 1918.

*Botryosphaeria briosiana* (Turconi & Maffei) Weese, Sitzungsber. Akad. Wiss. Wien, Math.-Naturwiss. Kl., Abt. 1, 128: 708. 1919.

*Fusarium uncinatum* Wollenw., Ann. Mycol. 15(1/2): 54. 1917.

*Fusarium blackmannii* W. Br. & A.S. Horne (as ‘*blackmanni*’), Ann. Bot. (London) 38: 379. 1924.

*Fusarium entomophilum* Petch, Trans. Brit. Mycol. Soc. 11: 260. 1925.

*Fusarium lateritium* var. *tenue* Wollenw., Fusaria Autogr. Delin. 3: 955. 1930.

*Gibberella saubinetii* var. *flacca* Wollenw., Z. Parasitenk. (Berlin) 3: 433. 1931.

*Fusarium anisophilum* Picado, J. Dept. Agric. Porto Rico 16: 391. 1932.

*Lectotypus* (*hic designatus*, MBT 10000710): **Germany**, unknown host, 1817, G.C.D. Nees von Esenbeck, in System der Pilze und Schwämme: 31, tab. 2, fig. 26.

*Descriptions and illustrations*: See Wollenweber & Reinking (1935), Booth (1971), Gerlach & Nirenberg (1982), Nelson *et al.* (1983) and Leslie & Summerell (2006).

*Notes*: Re-collection from the type host and locality is required. No holotype specimen could be located and therefore an illustration was designated as lectotype.  

*laxum Fusarium* Peck, Bull. New York State Mus. Nat. Hist. 67: 30. 1903.

(See ***Fusarium oxysporum***)

*Holotypus*: NYS-F-001667.

*Type locality*: **USA**, New York, Albany, Delmar.

*Type substrate*: *Equisetum hyemale*.

*Note*: Synonym *fide*Wollenweber & Reinking (1935).  

*leguminum Fusarium* (Cooke) Sacc., Syll. Fung. 4: 712. 1886.

*Basionym*: *Fusisporium leguminum* Cooke, Grevillea 6: 139. 1878.

(See ***Fusarium lateritium***)

*Syntypes*: In CUP, ISC, NEB & PH (Fungi Amer. Exs. no. 298).

*Type locality*: **USA**, South Carolina, Aiken.

*Type substrate*: *Acacia* sp.

*Note*: Synonym *fide*Wollenweber & Reinking (1935).  

*leucoconium Fusarium* Corda, Icon. Fung. 1: 4. 1837.

(See ***Fusarium heterosporum*** and ***F. reticulatum***)

*Typus*: In PRM *fide*Pilat (1938).

*Type locality*: **Czech Republic**, Prague.

*Type substrate*: Rotten plants.

*Note*: Synonym *fide*Wollenweber & Reinking (1935) and Booth (1971). Lectotypification pending study of material lodged in PRM.  

***libertatis Fusarium*** L. Lombard & Crous, Persoonia 43: 29. 2018 [2019].

*Holotypus*: CBS H-23618.

*Ex-type culture*: CBS 144749 = CPC 28465.

*Type locality*: **South Africa**, Western Cape Province, Robben Island, Van Riebeeck's Quarry.

*Type substrate*: Rock surface*.*

*Descriptions and illustrations*: See Lombard *et al.* (2019b).

*Diagnostic DNA barcodes*: *rpb2*: MH484944; *tef1*: MH485035.  

*lichenicola Fusarium* C. Massal., in Maire & Saccardo, Ann. Mycol. 1: 223. 1903.

***Neocosmospora lichenicola*** (C. Massal.) Sand.-Den. & Crous, Persoonia 41: 120. 2018.

*Synonyms*: *Bactridium lichenicola* (C. Massal.) Wollenw. (‘as *lichenicolum*’), Fusaria Autogr. Delin. 1: 456. 1916.

*Cylindrocarpon lichenicola* (C. Massal.) D. Hawksw., Bull. Brit. Mus. (Nat. Hist.), Bot. 6: 273. 1979.

*Selenosporium lichenicola* Speg., Anales Mus. Nac. Hist. Nat. Buenos Aires, ser. 3, 13: 459. 1911.

*Fusarium lichenicola* (Speg.) Sacc. & Trotter, Syll. Fung. 22: 1486. 1913, *nom. illegit.*, Art. 53.1.

*Monacrosporium tedeschii* A. Agostini (as *‘tedeschi’*), Atti Ist. Bot. Univ. Lab. Crittog. Pavia, ser. 3, 4: 195. 1933.

*Euricoa dominguiesii* Bat. & H. Maia, Anais Soc. Biol. Pernambuco 13: 152. 1955.

*Hyaloflorea ramosa* Bat. & H. Maia, Anais Soc. Biol. Pernambuco 13: 155. 1955.

*Neocosmospora ramosa* (Bat. & H. Maia) L. Lombard & Crous, Stud. Mycol. 80: 227. 2015.

*Mastigosporium heterosporum* R.H. Petersen, Mycologia 51: 729. 1959.

*Holotypus*: In PAD.

*Epitypus*: CBS H-23983, designated in Sandoval-Denis *et al.* (2019).

*Ex-epitype culture*: CBS 623.92.

*Epitype locality*: **Germany**, Göttingen.

*Epitype substrate*: Necrotic wounds of *Homo sapiens* under chemotherapy*.*

*Descriptions and illustrations*: See Sandoval-Denis *et al.* (2019).

*Diagnostic DNA barcodes*: *rpb2*: LR583845; *tef1*: LR583620.  

*limonis Fusarium* Briosi, Ann. R. Staz. Chim.-Agrar. Sper. Roma. 1878.

(See ***Fusarium lateritium***)

*Holotypus*: Not located.

*Type locality*: **Italy**, Sicily.

*Type substrate*: *Citrus limon*.

*Notes*: Synonym *fide*Wollenweber & Reinking (1935). Protologue not located.  

*limosum Fusarium* Rostr., Bot. Tidsskr. 22: 263. 1899.

(See ***Fusarium avenaceum***)

*Holotypus*: C-F-111719.

*Type locality*: **Sweden**.

*Type substrate*: Mixture of lime and sugar.

*Note*: Synonym *fide*Wollenweber & Reinking (1935).  

*lineare Fusarium* Moesz, Bot. Közlem. 19: 57. 1920.

(See *Fusarium obtusisporum*)

*Holotypus*: ?BP.

*Type locality*: **Hungary**.

*Type substrate*: *Staphylea pinnata*.

*Note*: Synonym *fide*Wollenweber & Reinking (1935).  

*lini Fusarium* Bolley, Proc. Annual Meeting Soc. Promot. Agric. Sci. 22: 42. 1901.

(See ***Fusarium oxysporum***)

*Holotypus*: Not located.

*Type locality*: **USA**.

*Type substrate*: *Linum usitatissimum*.  

*lini Fusarium* Remer, Jahresber. Schles. Ges. Vaterl. Cult. 80: 25. 1903, *nom. illegit.*, Art. 53.1

*Holotypus*: Not located.

*Type locality*: **Poland**.

*Type substrate*: *Linum* sp.  

*liriodendri Fusarium* (Sand.-Den. & Crous) O'Donnell *et al.*, Index Fungorum 440: 2. 2020.

***Neocosmospora liriodendri*** Sand.-Den. & Crous, Persoonia 43: 139. 2019.

*Holotypus*: CBS H-23984.

*Ex-type culture*: BBA 67587 = CBS 117481 = G.J.S 91-148 = NRRL 22389.

*Type locality*: **USA**, Maryland.

*Type substrate*: *Liriodendron tulipifera.*

*Descriptions and illustrations*: See Sandoval-Denis *et al.* (2019).

*Diagnostic DNA barcodes*: *rpb1*: MW218124; *rpb2*: EU329506; *tef1*: AF178340.  

*loliaceum Fusarium* Ducomet, Ann. École Natl. Agric. Rennes 2: 14. 1909.

(See *Fusarium nivale*)

*Holotypus*: ?MPA.

*Type locality*: **France**.

*Type substrate*: Unknown.

*Note*: Synonym *fide*Wollenweber & Reinking (1935).  

*lolii Fusarium* (Wm.G. Sm.) Sacc., Syll. Fung. 11: 652. 1895.

*Basionym*: *Fusisporium lolii* Wm.G. Sm., Diseases of field and garden crops, chiefly as are caused by fungi: 213. 1884.

(See ***Fusarium heterosporum***)

*Lectotypus* (*hic designatus*, MBT 10000711): **UK**, *Lolium perenne*, date unknown, W.G. Smith, in W.G. Smith, Diseases of field and garden crops, chiefly as are caused by fungi: 213, fig. 96.

*Notes*: Synonym *fide*Wollenweber & Reinking (1935). No holotype specimen could be located and therefore an illustration is designated as lectotype.  

*loncheceras Fusarium* Sideris, Phytopathology 14: 213. 1924.

(See ***Fusarium oxysporum***)

*Lectotypus* (*hic designatus*, MBT 10000712): **USA**, California, Stockton, roots of *Allium cepa*, 1924, C.P. Sideris*,* in Phytopathology 14, pl. XI, fig. of *F. loncheceras*.

*Notes*: Synonym *fide*Wollenweber & Reinking (1935). No holotype specimen could be located and therefore an illustration is designated as lectotype.  

***longicaudatum Fusarium*** J.W. Xia *et al.*, Persoonia 43: 208. 2019.

*Holotypus*: CBS H-24061.

*Ex-type culture*: ATCC 24370 = CBS 123.73 = IMI 160825 = NRRL 25477.

*Type locality*: **Tanzania**, Tropical Products Research Inst.

*Type substrate*: Unknown*.*

*Descriptions and illustrations*: See Xia *et al.* (2019).

*Diagnostic DNA barcodes*: *rpb2*: MN170414; *tef1*: MN170481.  

***longicornicola Fusarium*** Sand.-Den., *et al.*, Persoonia 46: 149. 2021.

*Holotypus*: CBS H-24661.

*Ex-type culture*: ARSEF 6455 = CBS 147247 = NRRL 52706.

*Type locality*: **Ethiopia**, Kobo, Welo.

*Type substrate*: *Aiolopus longicornis*.

*Descriptions and illustrations*: See Yilmaz *et al.* (2021).

*Diagnostic DNA barcodes*: *rpb2*: JF741114; *tef1*: JF740788.  

***longifundum Fusarium*** J.W. Xia *et al.*, Persoonia 43: 208. 2019.

*Holotypus*: CBS H-24062.

*Ex-type culture*: CBS 235.79 = NRRL 36372.

*Type locality*: **Netherlands Antilles**, Curaçao.

*Type substrate*: Air*.*

*Descriptions and illustrations*: See Xia *et al.* (2019).

*Diagnostic DNA barcodes*: *rpb2*: GQ505827; *tef1*: GQ505649.  

***longipes Fusarium*** Wollenw. & Reinking, Phytopathology 15: 160. 1925.

*Synonyms*: *Fusarium scirpi* var. *longipes* (Wollenw. & Reinking) Wollenw., Z. Parasitenk. (Berlin) 3: 337. 1931.

*Fusarium equiseti* var. *longipes* (Wollenw. & Reinking) Joffe, Mycopathol. Mycol. Appl. 53: 221. 1974.

*Neotypus* (*hic designatus*, MBT 10000713): **USA**, Florida, soil, 1977, W. Gams, CBS 476.77 (preserved as metabolically inactive culture).

*Ex-neotype culture*: CBS 476.77 = NRRL 20695.

*Descriptions and illustrations*: See Gerlach & Nirenberg (1982), Nelson *et al.* (1983).

*Diagnostic DNA barcodes*: *rpb1*: MW233244; *rpb2*: GQ915493; *tef1*: GQ915509.

*Notes*: This species is recognised by Gerlach & Nirenberg (1982), Nelson *et al.* (1983), and Leslie & Summerell (2006). No holotype specimen could be located and no illustration accompanied the original protologue. Although an illustration of the original culture (O.A. Reinking no. R34) is provided in Wollenweber's Fusaria Autogr. Delin. no. 937 (1924), this cannot be used to designate a lectotype as it does not form part of the original protologue. Therefore, isolate CBS 476.77 is designated as neotype here to provide taxonomic stability to this species, as it appears to have a paraphyletic phylogenetic structure (O'Donnell *et al.* 2013).  

*longisporum Fusarium* Cooke & Massee, Grevillea 16: 4. 1887.

(See ***Fusarium lateritium***)

*Holotypus*: K(M) 159680.

*Type locality*: **Australia**, Queensland, Brisbane.

*Type substrate*: Twigs of *Passiflora* sp.

*Note*: Synonym *fide*Wollenweber & Reinking (1935).  

*longissimum Fusarium* Sacc. & P. Syd., Syll. Fung. 14: 1128. 1899.

*Replaced synonym*: *Fusarium elongatum* De Wild., Ann. Soc. Belge Microscop. 17: 43. 1893, *nom. illegit.*, Art. 53.1, *non Fusarium elongatum* Cooke 1890.

(See *Fusarium elongatum* De Wild.)

*Holotypus*: Not located.

*Type locality*: **Belgium**, Brussels, Botanical Garden.

*Type substrate*: Submerged plant material.

*Note*: Synonymy *fide*Rossman *et al.* (2016).  

*longum Fusarium* (Wallr.) Sacc., Syll. Fung. 4: 719. 1886.

*Basionym*: *Fusisporium longum* Wallr., Fl. Crypt. Germ. 2: 283. 1833.

*Holotypus*: ?STR.

*Type locality*: **Germany**, Berlin.

*Type substrate*: Dead branch.

*Notes*: Status unclear. Not *Fusarium fide*Wollenweber & Reinking (1935).  

***louisianense Fusarium*** L.R. Gale *et al.*, Fungal Genet. Biol*.* 48: 1105. 2011.

*Holotypus*: BPI 881005.

*Ex-type culture*: CBS 127525 = NRRL 54197.

*Type locality*: **USA**, Louisiana.

*Type substrate*: Seeds of *Triticum* sp*.*

*Descriptions and illustrations*: See Sarver *et al.* (2011).

*Diagnostic DNA barcodes*: *rpb1*: KM889655; *rpb2*: KM889657; *tef1*: KM889633.  

*lucidum Fusarium* Sherb., Mem. Cornell Univ. Agric. Exp. Sta. 6: 157. 1915.

(See ***Fusarium avenaceum***)

*Typus*: ?CUP-007473.

*Type locality*: **USA**, New York.

*Type substrate*: *Solanum tuberosum.*

*Notes*: Synonym *fide*Wollenweber & Reinking (1935). Lectotypification pending study of material lodged in CUP.  

*lucumae Fusarium* Henn., Hedwigia 48: 116. 1908.

***Ascochyta lucumae*** (Henn.) Wollenw., Fusaria Autogr. Delin. 1: 504. 1916.

*Syntypes*: In BPI, ILL, MIN & WIS (Baker 218).

*Type locality*: **Brazil**, Pará.

*Type substrate*: *Lucuma rivicoa*

*Note*: Synonym *fide*Wollenweber & Reinking (1935).  

***luffae Fusarium*** M.M. Wang *et al.*, Persoonia 43: 85. 2019

*Holotypus*: HAMS 248042.

*Ex-type culture*: CGMCC 3.19497 = LC12167.

*Type locality*: **China**, Fujian.

*Type substrate*: *Luffa aegyptiaca*.

*Descriptions and illustrations*: See Wang *et al.* (2019).

*Diagnostic DNA barcodes*: *rpb1*: MK289869; *rpb2*: MK289754; *tef1*: MK289601.  

***lumajangense Fusarium*** Maryani *et al.*, Persoonia 43: 59. 2019.

*Holotypus*: InaCC F872 (preserved as metabolically inactive culture).

*Ex-type culture*: InaCC F872.

*Type locality*: **Indonesia**, East Java, Lumajang, Kecamatan Senduro, Desa Kandang Kepus.

*Type substrate*: *Musa acuminata* var. Pisang Mas Kirana*.*

*Descriptions and illustrations*: See Maryani *et al.* (2019b).

*Diagnostic DNA barcodes*: *rpb2*: LS479850; *tef1*: LS479441.  

*lunatum Fusarium* (Ellis & Everh.) Arx, Verh. Kon. Ned. Akad. Wetensch., Afd. Natuurk., Sect. 2, 51: 101. 1957.

***Bisifusarium lunatum*** (Ellis & Everh.) L. Lombard & Crous, Stud. Mycol. 80: 225. 2015.

*Basionym*: *Gloeosporium lunatum* Ellis & Everh., Proc. Acad. Nat. Sci. Philadelphia 43: 82. 1891.

*Synonyms*: *Microdochium lunatum* (Ellis & Everh.) Arx, Trans. Brit. Mycol. Soc. 83: 374. 1984.

*Fusarium dimerum* var. *violaceum* Wollenw., Fusaria Autogr. Delin. 3: 854. 1930.

*Holotypus*: NY00883039.

*Type locality*: **USA**, Texas, San Antonio.

*Type substrate*: Living leaves of *Opuntia* sp.

*Notes*: This species requires epitypification. Gerlach & Nirenberg (1982) designated CBS 632.76 (= NRRL 20690) as neotype of *F. dimerum* var. *violaceum*, which was originally collected in Germany. However, Schroers *et al.* (2009) showed that *F. lunatum* is paraphyletic and needs further investigation. Therefore, CBS 632.76 cannot be designated as epitype for *B. lunatum* at this time.  

***lunulosporum Fusarium*** Gerlach, Phytopathol. Z. 88: 283. 1977.

*Holotypus*: BBA 62459.

*Ex-type culture*: ATCC 36747 = BBA 62459 = CBS 636.76 = IMI 322097 = NRRL 13393.

*Type locality*: **South Africa**.

*Type substrate*: *Citrus paradisi.*

*Descriptions and illustrations*: See Gerlach (1977b), Gerlach & Nirenberg (1982) and Nelson *et al.* (1983).

*Diagnostic DNA barcodes*: *rpb1*: KM361637; *rpb2*: KM361655; *tef1*: AF212467.  

*luteum Fusarium* Clem., Bot. Surv. Nebraska 3: 12. 1894.

(See ***Fusarium lateritium***)

*Holotypus*: NEB00040542.

*Type locality*: **USA**, Nebraska, Lincoln.

*Type substrate*: Decaying wood.

*Note*: Synonym *fide*Wollenweber & Reinking (1935).  

*luteum Fusarium* Parav., Ann. Mycol. 16: 302. 1918, *nom. illegit.*, Art. 53.1.

(See *Fusarium candidum*)

*Authentic material*: In Ann. Mycol. 16, pl. 4., figs 1–22.

*Original locality*: **Switzerland**.

*Original substrate*: *Pyrus* sp.

*Notes*: Synonym *fide*Wollenweber & Reinking (1935).  

*lutulatum Fusarium* Sherb., Mem. Cornell Univ. Agric. Exp. Sta. 6: 209. 1915.

(See ***Fusarium oxysporum***)

*Typus*: CUP-007458.

*Type locality*: **USA**, Iowa.

*Type substrate*: *Solanum tuberosum*.

*Note*: Synonym *fide*Wollenweber & Reinking (1935). Lectotypification pending study of material lodged in CUP.  

***lyarnte Fusarium*** J.L. Walsh, Sangal., L.W. Burgess, E.C.Y. Liew & Summerell, ***sp. nov*.** MycoBank MB 837697.

*Synonym*: *Fusarium lyarnte* J.L. Walsh, Sangal., L.W. Burgess, E.C.Y. Liew & Summerell, Fungal Diversity 44: 153. 2010, *nom. inval.*, Art. 40.7.

*Etymology*. ‘Lyarnte’, meaning circle in eastern and central Arrernte Aboriginal language (Henderson & Dobson 1994), in reference to the conspicuous globose microconidia.

For diagnosis see Walsh *et al.*, Fungal Diversity 44: 153. 2010.

*Holotypus*: CBS 125536 (preserved as metabolically inactive culture).

*Ex-type culture*: CBS 125536 = NRRL 54252 = RBG 5331.

*Type locality*: **Australia**, Northern Territory, Litchfield.

*Type substrate*: Soil*.*

*Descriptions and illustrations*: See Walsh *et al.* (2010).

*Diagnostic DNA barcodes*: *rpb1*: JX171549; *rpb2*: JX171661; *tef1*: EF107118.

*Notes*: Walsh *et al.* (2010) failed to indicate the holotype for *F. lyarnte*, thereby rendering the species name invalid (Art. 40.7). Here we validate the name.  

*lycopersici Fusarium* (Sacc.) Mussat, Syll. Fung. 15: 144. 1901, *nom. inval*., Art. 36.1(a), (c).

*Basionym*: *Fusarium oxysporum* subsp. *lycopersici* Sacc., Syll. Fung. 4: 705. 1886.

(See ***Fusarium oxysporum***)

*Authentic material*: Not located.

*Original locality*: **Italy**.

*Original substrate*: *Solanum lycopersicum*.  

*lycopersici Fusarium* Bruschi, Atti Reale Accad. Lincei, Rendiconti Cl. Sci. Fis., ser. 5, 21: 298. 1912.

(See ***Fusarium oxysporum***)

*Synonym*: *Fusarium bulbigenum* var. *lycopersici* (Bruschi) Wollenw. & Reinking, *Fusarien*: nos. 996–997. 1935.

*Holotypus*: Not located*.*

*Type locality*: **Italy**.

*Type substrate*: *Solanum lycopersicum*.

*Note*: Synonym *fide*Wollenweber & Reinking (1935).  

*lycopersici Fusarium* (Sacc.) Wollenw., Phytopathology 3: 29. 1913, *nom. illegit.*, Art. 53.1.

*Basionym*: *Fusarium oxysporum* subsp. *lycopersici* Sacc., Syll. Fung. 4: 705. 1886.

(See ***Fusarium oxysporum***)

*Authentic material*: Not located.

*Original locality*: **Italy**.

*Original substrate*: *Solanum lycopersicum*.  

*macounii Fusarium* Dearn., Mycologia 9: 363. 1917.

(See ***Fusarium expansum***)

*Holotypus*: DAOM 223428b.

*Type locality*: **Canada**, Vancouver Island.

*Type substrate*: *Acer* sp.

*Note*: Synonym *fide*Wollenweber & Reinking (1935).  

***macroceras Fusarium*** Wollenw. & Reinking, Phytopathology 15: 166. 1925.

*Holotypus*: CBS 146.25 (preserved as metabolically inactive culture).

*Ex-type culture*: CBS 146.25 = NRRL 13958.

*Type locality*: **Honduras**.

*Type substrate*: *Phaseolus vulgaris.*

*Descriptions and illustrations*: See Wollenweber & Reinking (1925, 1935) and Gerlach & Nirenberg (1982).

*Notes*: Phylogenetic inference (not shown) revealed that the ex-type culture housed at CBS clustered within the *N. petroliphila* clade, indicating a possible strain transposition or contamination of the culture in the past. These species are not morphologically conspecific based on the original protologue (Wollenweber & Reinking 1925) of *F. macroceras*.  

*macrosporum Fusarium* (Sand.-Den. *et al.*) O'Donnell *et al.*, Index Fungorum 440: 2. 2020.

***Neocosmospora macrospora*** Sand.-Den. *et al.*, Persoonia 40: 21 2017 [2018].

*Holotypus*: CBS H-23023.

*Ex-type culture*: CBS 142424 = CPC 28191.

*Type locality*: **Italy**, Sicily, Catania, Guardia.

*Type substrate*: *Citrus sinensis.*

*Descriptions and illustrations*: See Sandoval-Denis *et al.* (2018a).

*Diagnostic DNA barcodes*: *rpb1*: MW218125; *rpb2*: LT746331; *tef1*: LT746218.  

*macroxysporum Fusarium* Lindf., Meddel. Centralanst. Försöksväs. Jordbruksomr. Avd. Lantbruksbot. 25: 8. 1922.

(See ***Fusarium oxysporum***)

*Holotypus*: Not located.

*Type locality*: **Sweden**.

*Type substrate*: *Pinus sylvestris*.

*Note*: Synonym *fide*Wollenweber & Reinking (1935).  

*maculans Fusarium* Bérenger, Atti Riunione Sci. Ital. 6: 474. 1845.

***Neophloeospora maculans*** (Bérenger) Videira & Crous, Stud. Mycol. 87: 338. 2017.

*Synonyms*: *Phloeospora maculans* (Bérenger) Allesch., Rabenh. Krypt.-Fl., ed. 2, 1: 935. 1900.

*Phloeosporella maculans* (Bérenger) Ho¨hn., Mitt. Bot. Inst. Techn. Hochsch. Wien 4: 77. 1927.

*Cercosporella maculans* (Bérenger) F.A. Wolf, J. Elisha Mitchell Sci. Soc. 51: 165. 1935.

*Septoria mori* Lév., Ann. Sci. Nat., Bot., ser. 3, 5: 279. 1846.

*Cheilaria mori* (Lév.) Desm., Ann. Sci. Nat., Bot., ser. 3, 8: 27. 1847.

*Phloeospora mori* (Lév.) Sacc., Michelia 1: 175. 1878.

*Septogloeum mori* (Lév.) Briosi & Cavara, Fung. Paras. Piante Colt. Util., Fasc. 1: no. 21. 1888.

*Cylindrosporium mori* (Lév.) Berl., Riv. Patol. Veg. 5: 205. 1896.

*Sphaeria mori* Nitschke, Fungi Rhen. Exs. no. 1784. 1866, *nom. inval*., Art. 38.1(a).

*Sphaerella mori* Fuckel, Jahrb. Nassauischen Vereins Naturk. 23–24: 106. 1870.

*Mycosphaerella mori* (Fuckel) F.A. Wolf, J. Elisha Mitchell Sci. Soc. 51: 165. 1935.

*Sphaerella morifolia* Pass., Erb. Critt. Ital., Ser. 2, Fasc. 30, no. 1464. 1885.

*Mycosphaerella morifolia* (Pass.) Cruchet, Bull. Soc. Vaud. Sci. Nat. 55: 43. 1923.

*Cercospora pulvinulata f*. *angulosa* Săvul. & Sandu, Herb. Mycol. Roman. no. 188. 1931.

*Holotypus*: Not located.

*Type locality*: **Italy**.

*Type substrate*: Leaves of *Morus* sp.  

***madaense Fusarium*** Ezekiel *et al.*, MycoKeys 67: 112. 2020.

*Holotypus*: CBS H-24346.

*Ex-type culture*: CBS 146669 = CPC 38344.

*Type locality*: **Nigeria**, Nasarawa, Mada Station.

*Type substrate*: *Arachis hypogaea.*

*Descriptions and illustrations*: See Ezekiel *et al.* (2020).

*Diagnostic DNA barcodes*: *rpb1*: LR792575; *rpb2*: LR792589; *tef1*: LR792625.  

***magnoliae-champaca Fusarium*** R.H. Perera *et al.*, Mycosphere 11: 2140. 2020.

*Holotypus*: MFLU 18-2736.

*Ex-type culture*: MFLUCC 18-0580.

*Type locality*: **Thailand**, Chiang Rai, Mae Fah Luang University garden.

*Type substrate*: Dried fruits of *Magnolia champaca.*

*Descriptions and illustrations*: See Perera *et al.* (2020).

*Diagnostic DNA barcode*: *rpb2*: MT212198.  

*magnusianum Fusarium* Allesch., Fungi Bav. no. 400. 1895.

(See *Fusarium aquaeductuum*)

*Holotypus*: In M.

*Type locality*: **Germany**, München.

*Type substrate*: *Salix incana.*

*Note*: Synonym *fide*Wollenweber & Reinking (1935).  

*mahasenii Fusarium* Samuels *et al.*, Mycologia 103: 1325. 2011.

***Neocosmospora mahasenii*** Samuels *et al.*, Mycologia 103: 1325. 2011.

*Holotypus*: BPI 881228.

*Ex-type culture*: CBS 119594 = FRC S-1845 = G.J.S. 02-105.

*Type locality*: **Sri Lanka**, North Central Province, Giritale. Giritale Forest Training Center.

*Type substrate*: Small branch of live tree*.*

*Descriptions and illustrations*: See Nalim *et al.* (2011).

*Diagnostic DNA barcodes*: *rpb1*: MW834231; *rpb2*: LT960563; *tef1*: DQ247513.  

*mali Fusarium* Allesch., Ber. Bot. Vereines Landshut 12: 130. 1892.

(See *Fusarium candidum*)

*Holotypus*: In M.

*Type locality*: **Germany**, München.

*Type substrate*: *Malus pumila*.

*Note*: Synonym *fide*Wollenweber & Reinking (1935).  

*malli Fusarium* Taubenh., Bull. Texas Agric. Exp. Sta. 273: 25. 1921.

(See *Fusarium solani*)

*Holotypus*: ?CUP-011254.

*Type locality*: **USA**, Texas, Brazos, College Station.

*Type substrate*: *Allium cepa.*

*Note:* Typification pending study of material lodged in CUP.  

*malvacearum Fusarium* Taubenh., Bull. Texas Agric. Exp. Sta. 260: 27. 1920.

(See ***Fusarium oxysporum***)

*Lectotypus* (*hic designatus*, MBT 10000714): **USA**, Texas, *Abelmoschus esculentus*, 1920, J.J. Taubenhhaus, in Taubenhaus, Bull. Texas Agric. Exp. Sta. 260: 30, fig. 8g.

*Notes*: Synonym *fide*Wollenweber & Reinking (1935). No holotype specimen could be located and therefore an illustration was designated as lectotype.  

***mangiferae Fusarium*** Britz *et al.*, Mycologia 94: 725. 2002.

*Holotypus*: PREM 57299.

*Ex-type culture*: CBS 120994 = KSU 11781 = MRC 7559 = MUCL 54671 = NRRL 53980.

*Type locality*: **Israel**, Bet Dagan, Volcani Center.

*Type substrate*: *Mangifera indica.*

*Descriptions and illustrations*: See Britz *et al.* (2002).

*Diagnostic DNA barcodes*: *rpb1*: MW402530; *rpb2*: LT575059; *tef1*: LT574978.  

***marasasianum Fusarium*** Herron *et al.*, Stud. Mycol. 80: 146. 2015.

*Holotypus*: PREM 60899.

*Ex-type culture*: CBS 137238 = CMW 25261.

*Type locality*: **Colombia**, Vivero Peñas Negra, Valle del Cauca.

*Type substrate*: *Pinus patula.*

*Descriptions and illustrations*: See Herron *et al.* (2015).

*Notes*: Comparisons of recently generated sequences for the living ex-type (CBS 137238 = CMW 25261) of *F. marasasianum* indicate a strain transposition or contamination by another *Fusarium* species. Therefore, this species needs to be recollected from the type locality and substrate or sequences need to be generated from the holotype specimen.  

*marginatum Fusarium* Berk. & M.A. Curtis, Grevillea 3: 97. 1875.

*Holotypus*: ?K(M).

*Type locality*: **USA**, Alabama, Beaumont.

*Type substrate*: *Smilax* sp.

*Note*: Not *Fusarium fide*Wollenweber & Reinking (1935).  

*martiellae-discolorioides Fusarium* Batikyan, Biol. Zhurn. Armenii 22: 87. 1969, *nom. inval*., Art. 39.1.

*Authentic material*: Not located.

*Original locality*: **Armenia**.

*Original substrate*: Soil of wheatfield.

*Notes*: Published without Latin diagnosis *fide*Gerlach & Nirenberg (1982). Also described in Biol. Zhurn. Armenii 26(2): 73. 1973, but also not in Latin.  

*martii Fusarium* Appel & Wollenw., Arbeiten Kaiserl. Biol. Anst. Land- Forstw. 8: 83. 1913.

***Neocosmospora martii*** (Appel & Wollenw.) Sand.-Den. & Crous, Persoonia 43: 137. 2019.

*Synonyms*: *Fusarium solani* var. *martii* (Appel & Wollenw.) Wollenw., Fusaria Autogr. Delin. 3: 1034. 1930.

*Neocosmospora croci* Guarnaccia *et al.*, Persoonia 40: 17. 2017 [2018].

*Lectotypus*: BPI 452385, selected in Sandoval-Denis *et al.* (2019).

*Epitypus*: CBS H-23986, designated in Sandoval-Denis *et al.* (2019).

*Ex-epitype culture*: CBS 115659 = FRC S-0679 = MRC 2198.

*Lecto- and epitype locality*: **Germany**, Berlin.

*Lecto- and epitype substrate*: *Solanum tuberosum.*

*Descriptions and illustrations*: See Sandoval-Denis *et al.* (2019).

*Diagnostic DNA barcodes*: *rpb1*: MW834232; *rpb2*: JX435256; *tef1*: JX435156.  

***massalimae Fusarium*** A.D. Cavalcanti *et al.*, Mycol. Progr. 19: 1137. 2020.

*Holotypus*: URM 94324.

*Ex-type culture*: URM 8239.

*Type locality*: **Brazil**, Alagoas, Quebrangulo, Pedra Talhada Biological Reserve.

*Type substrate*: *Handroanthus chrysotrichus.*

*Descriptions and illustrations*: See Cavalcanti *et al.* (2020).

*Diagnostic DNA barcodes*: *rpb2*: MN939767; *tef1*: MN939763.  

*matuoi Fusarium* Hosoya & Tubaki, Mycoscience 45: 264. 2004.

***Fusicolla matuoi*** (Hosoya & Tubaki) Gräfenhan & Seifert, Stud. Mycol. 68: 101. 2011.

*Synonyms*: *Fusarium splendens* Matuo & Takah. Kobay., Trans. Mycol. Soc. Japan 2(4): 13. 1960, *nom. inval*., Art. 39.1.

*Cosmospora matuoi* Hosoya & Tubaki, Mycoscience 45: 262. 2004.

*Holotypus*: TNS F-11127.

*Ex-type culture*: MAFF 410976.

*Type locality*: **Japan**, Honshu.

*Type substrate*: Twigs of *Albizia julibrissin.*

*Descriptions and illustrations*: See Hosoya & Tubaki (2004).  

*mauroi Fusarium* Av.-Saccá, Revista Agric. (Piracicaba) 8: 93. 1933.

***Macronectria jungneri*** (Henn.) Salgado & P. Chaverri, Fungal Diversity 80: 448. 2016.

*Basionym*: *Nectria jungneri* Henn., Bot. Jahrb. Syst. 22: 75. 1895.

*Synonyms*: *Cucurbitaria jungneri* (Henn.) Kuntze, Revis. Gen. Pl. 3: 461. 1898.

*Neonectria jungneri* (Henn.) Samuels & Brayford (as ‘*Nenectria*’), Mycologia 96: 580. 2004.

*Thelonectria jungneri* (Henn.) P. Chaverri & Salgado, Stud. Mycol. 68: 76. 2011.

*Nectria eustoma* Penz. & Sacc., Malpighia 11: 509. 1898 [1897].

*Nectria leucocoma* Starbäck, Bih. Kongl. Svenska Vetensk.-Akad. Handl. 25: 28. 1899.

*Nectria cinereopapillata* Henn. & E. Nyman, inWarburg, Monsunia 1: 161. 1900 [1899].

*Nectria striatospora* Zimm., Centralbl. Bakteriol. II, 7: 105. 1901.

*Nectria theobromae* Massee, Bull. Misc. Inform. Kew 1908: 218. 1908.

*Cylindrocarpon victoriae* Wollenw., Z. Parasitenk. (Berlin) 1: 161. 1928.

Nectria azureo-ostiolata Doi, Mem. Nat. Sci. Mus. Tokyo 10: 23. 1977.

*Holotypus*: Not located.

*Type locality*: **Brazil**.

*Type substrate*: *Caconema radicicola*.

*Note*: Synonyms *fide*Wollenweber & Reinking (1935) and Salgado-Salazar *et al.* (2016).  

*maydiperdum Fusarium* Bubák, Centralbl. Bakteriol. 2. Abth. 31: 497. 1911.

(See ***Fusarium poae***)

*Holotypus*: BPI 452399.

*Type locality*: **Czech Republic**, Tabor.

*Type substrate*: Seeds of *Zea mays*.

*Note*: Synonym *fide*Wollenweber & Reinking (1935).  

*maydis Fusarium* Kalchbr., Math. Term. Közlem. 3: 285. 1865.

(See ***Fusarium sambucinum***)

*Holotypus*: BRACR33140.

*Type locality*: **Hungary**.

*Type substrate*: *Zea mays*.

*Note*: Synonym *fide*Wollenweber & Reinking (1935).  

*melanochlorum Fusarium* (Casp.) Sacc., Syll. Fung. 4: 725. 1886.

*Basionym*: *Fusisporium melanochlorum* Casp., Ber. Bekanntm. Verh. Königl. Preuss. Akad. Wiss. Berlin 1855: 309, 314. 1855.

***Cosmospora flavoviridis*** (Fuckel) Rossman & Samuels, Stud. Mycol. 42: 121. 1999.

*Basionym*: *Sphaerostilbe flavoviridis* Fuckel, Jahrb. Nassauischen Vereins Naturk. 25–26: 310. 1871.

*Synonyms*: *Nectria flavoviridis* (Fuckel) Wollenw. Angew. Bot. 8: 186. 1926.

*Fusarium celtidis* Pass., Atti Reale Accad. Lincei, Rendiconti Cl. Sci. Fis., 4 sér. 7: 51. 1891, *nom. illegit.*, Art. 53.1.

*Fusarium sphaeriiforme* Sacc. (as ‘*sphaeriaeforme*’), Syll. Fung. 10: 723. 1892.

*Fusarium glandicola* Allesch., Ber. Bot. Vereines Landshut12: 130. 1892, *nom. illegit.*, Art. 53.1, *non* Cooke & W.R. Gerard, 1878.

*Fusarium allescheri* Sacc. & P. Syd., Syll. Fung. 14: 1128. 1899.

*Holotypus*: Not located.

*Type locality*: **Germany**, Berlin.

*Type substrate*: Rotten aquatic plants.

*meliolicola Fusarium* F. Stevens (as ‘*meliolicolum*’), Bot. Gaz. 65: 245. 1918.

(See *Fusarium larvarum*)

*Holotypus*: ILL00011251.

*Type locality*: **Puerto Rico**, Mayagüez.

*Type substrate*: Parasitic on *Meliola paulliniae* on *Casearia sylvestris*.

*Note*: Synonym *fide*Wollenweber & Reinking (1935).  

***meridionale Fusarium*** T. Aoki *et al.*, Fungal Genet. Biol. 41: 618. 2004.

*Holotypus*: BPI 843474.

*Ex-type culture*: CBS 110247 = FRC R-5329 = NRRL 28436.

*Type locality*: **New Caledonia**.

*Type substrate*: *Citrus sinensis.*

*Descriptions and illustrations*: See O'Donnell *et al.* (2004).

*Diagnostic DNA barcodes*: *rpb1*: KM361642; *rpb2*: KM361660; *tef1*: AF212435.  

*merismoides Fusarium* Corda, Icon. Fung. 2: 4. 1838.

***Fusicolla merismoides*** (Corda) Gräfenhan *et al.*, Stud. Mycol. 68: 101. 2011.

*Synonyms*: *Fusisporium georginae* Klotzsch, Herb. Viv. Mycol., Cent. 2: 186. 1832, *nom. nud.*, Art. 38.1 (a).

*Fusarium rhizophilum* Corda, Icon. Fung. 2: 3. 1838.

*Pionnotes rhizophila* (Corda) Sacc., Syll. Fung. 4: 727. 1886.

?*Fusisporium arachnoideum* Corda, Icon. Fung. 1: 11. 1837.

?*Fusarium arachnoideum* (Corda) Sacc., Syll. Fung. 4: 721. 1886.

?*Fusarium biasolettianum* Corda, Icon. Fung. 2: 3. 1838.

?*Fusisporium biasolettianum* (Corda) Sacc., Mycoth. Ven. no. 1040. 1877.

?*Pionnotes biasolettiana* (Corda) Sacc., Syll. Fung. 4: 725. 1886.

*Fusisporium udum* Berk., Ann. Mag. Nat. Hist. 6: 438. 1841.

*Pionnotes uda* (Berk.) Sacc., Syll. Fung. 4: 726. 1886.

*Fusarium udum* (Berk.) Wollenw., Phytopathology 3: 38. 1913, *nom. illegit.*, Art. 53.1.

*Fusidium udum* Berk., in Trotter, Syll. Fung. 25: 979. 1931, *nom. inval*., Art. 36.1.

*Fusisporium foeni* Berk. & Broome, Ann. Mag. Nat. Hist., ser. 2, 7: 179. 1851.

*Fusarium foeni* (Berk. & Broome) Sacc., Syll. Fung. 4: 699. 1886.

*Fusisporium roseolum* H.O. Stephens ex Berk. & Broome, Ann. Mag. Nat. Hist., ser. 2, 7: 178. 1851.

*Fusarium roseolum* (H.O. Stephens ex Berk. & Broome) Sacc., Syll. Fung. 4: 710. 1886.

*Fusisporium rimosum* Peck, Rep. (Annual) New York State Mus. Nat. Hist. 30: 58. 1878.

*Fusarium rimosum* (Peck) Sacc., Syll. Fung. 4: 713. 1886.

*Fusarium roesleri* Thüm., Pilze Weinst.: 51. 1878.

*Fusarium arvense* Speg., Anales Soc. Ci. Argent. 10: 60. 1880.

*Fusarium gallinaceum* Cooke & Harkn., Grevillea 9: 8. 1880.

*Fusarium nicotianae* Oudem., Ned. Kruidk. Arch., sér. 3, 2: 777. 1902.

*Fusarium udum* var. *pusillum* Wollenw., Phytopathology 1: 206. 1913, *nom. nud.*

*Fusarium udum* var. *solani* Sherb., Mem. Cornell Univ. Agric. Exp. Sta. 6: 131. 1915.

*Fusarium merismoides f*. *nicotianae* (Oudem.) Subram., Hyphomycetes: 676. 1971.

*Fusarium oxysporum f. sp. nicotianae* (Oudem.) Subram., Hyphomycetes: 676. 1971.

*Fusarium pelargonii* P. Crouan & H. Crouan, Fl. Finistère: 14. 1867.

*Fusarium albiziae* Woron., Vestn. Tiflissk. Bot Sada 48: 34. 1920.

*Fusarium merismoides* var. *majus* Wollenw., Fusaria Autogr. Delin. 3: 857a. 1930.

*Fusarium merismoides* var. *chlamydosporale* Wollenw., Z. Parasitenk. (Berlin) 3: 308. 1931.

*Fusarium merismoides* var. *artocarpi* X.H. Fu & Q.T. Chen, Acta Mycol. Sin. 8: 42. 1989.

*Fusarium merismoides* var. *persicicola* X.H. Fu & Q.T. Chen, Acta Mycol. Sin. 8: 44. 1989.

*Typus*: PRM 155493.

*Type locality*: **Czech Republic**, Prague.

*Type substrate*: Wet shards of a plant pot.

*Note*: Lectotypification pending study of material lodged in PRM.  

*mesentericum Fusarium* Cooke & Harkn., Grevillea 9: 128. 1881.

*Holotypus*: ?K(M).

*Type locality*: **USA**, California, San Francisco Masonic Cemetery.

*Type substrate*: *Eucalyptus* sp.

*Notes*: Status unclear. Not *Fusarium fide*Wollenweber & Reinking (1935).  

***mesoamericanum Fusarium*** T. Aoki *et al.*, Fungal Genet. Biol. 41: 619. 2004.

*Holotypus*: BPI 843476.

*Ex-type culture*: CBS 415.86 = FRC R-8506 = IMI 309346 = NRRL 25797.

*Type locality*: **Honduras**.

*Type substrate*: *Musa* sp*.*

*Descriptions and illustrations*: See O'Donnell *et al.* (2004).

*Diagnostic DNA barcodes*: *rpb1*: KM361639; *rpb2*: KM361657; *tef1*: AF212441.  

*metachroum Fusarium* Appel & Wollenw., Arbeiten Kaiserl. Biol. Anst. Land- Forstw. 8: 141. 1910 [1913].

(See ***Fusarium avenaceum***)

*Holotypus*: BPI 452408.

*Type locality*: **Poland**, Poznań, Slivno Manor.

*Type substrate*: *Triticum aestivum*.

*Note*: Synonym *fide*Wollenweber & Reinking (1935).  

*metavorans Fusarium* Al-Hatmi *et al.*, Medical Mycol. 56: S147. 2018.

***Neocosmospora metavorans*** (Al-Hatmi *et al.*) Sand.-Den. & Crous, Persoonia 41: 121. 2018.

*Holotypus*: CBS 135789 (preserved as metabolically inactive culture).

*Ex-type culture*: CBS 135789.

*Type locality*: **Greece**, Athens.

*Type substrate*: Pleural effusion of *Homo sapiens.*

*Descriptions and illustrations*: See Al-Hatmi *et al.* (2018) and Sandoval-Denis et al., 2018a, Sandoval-Denis et al., 2018b.

*Diagnostic DNA barcodes*: *rpb1*: MW218127; *rpb2*: LR583849; *tef1*: LR583627.  

***mexicanum Fusarium*** T. Aoki *et al.*, Phytopathology 100: 1180. 2010.

*Holotypus*: BPI 879150.

*Ex-type culture*: NRRL 53147.

*Type locality*: **Mexico**, Nueva Italia, Michoacán.

*Type substrate*: *Mangifera indica.*

*Descriptions and illustrations*: See Otero-Colina *et al.* (2010).

*Diagnostic DNA barcodes*: *rpb1*: MG838088; *rpb2*: MN724973; *tef1*: MG838032.  

*microcera Fusarium* Bilaĭ, Fusarii (Biologija i sistematika): 292. 1955, *nom. inval*., Art. 39.1.

(See *Fusarium coccidicola*)

*Note*: This species was invalidly published without Latin diagnosis.  

***microconidium Fusarium*** L. Lombard & Crous, Fungal Syst. Evol. 4: 192. 2019.

*Holotypus*: CBS H-24017.

*Ex-type culture*: CBS 119843 = MRC 8391 = KSU 11396.

*Type locality*: **Unknown**.

*Type substrate*: Unknown*.*

*Descriptions and illustrations*: See Lombard *et al.* (2019a).

*Diagnostic DNA barcodes*: *rpb1*: MN120721; *tef1*: MN120759.  

*microphlyctis Fusarium* Mont., Ann. Sci. Nat., Bot., sér. 3, 12: 297. 1849.

*Holotypus*: ?PC.

*Type locality*: **France**.

*Type substrate*: Fruit of *Olea* sp.

*Note*: *Gloeosporium fide*Wollenweber & Reinking (1935).  

*micropus Fusarium* Sacc., Philipp. J. Sci. 18: 605. 1921.

***Infrafungus micropus*** (Sacc.) Cif., Mycopathol. Mycol. Appl. 6: 26. 1951.

*Holotypus*: In PAD.

*Type locality*: **China**, Guangdong Province.

*Type substrate*: Parasitic on *Cladosporium herbarum* on leaf of *Morus alba*.  

*microspermum Fusarium* Berk. & M.A. Curtis, Grevillea 3: 98. 1875.

*Holotypus*: ?K(M).

*Type locality*: **USA**, South Carolina, Santee River.

*Type substrate*: Ficus sp.

*Note*: *Hymenula fide*Wollenweber & Reinking (1935).  

*microsporum Fusarium* Schltdl., Fl. Berol. 2: 139. 1824.

(See ***Fusarium lateritium***)

*Holotypus*: HAL 1615 F.

*Type locality*: **Germany**, Berlin.

*Type substrate*: *Robinia pseudoacaciae*.

*Note*: Synonym *fide*Wollenweber & Reinking (1935).  

*mikaniae Fusarium* Berk. & M.A. Curtis, Grevillea 3: 98. 1875.

*Holotypus*: ?K(M).

*Type locality*: **USA**, South Carolina, Santee River.

*Type substrate*: Stems and leaves of *Mikania scandens*.

*Notes*: Status unclear. Not *Fusarium fide*Wollenweber & Reinking (1935).  

***mindoanum Fusarium*** Petr., Sydowia 4: 576. 1950.

*Holotypus*: In W as no. 03550 (Petrak, Pilzherbarium no. 32229).

*Type locality*: **Ecuador**, Pichincha, Mindo.

*Type substrate*: *Dryopteris diplazioides*.

*Notes*: No living material available to confirm taxonomic status. Requires recollection from type locality and substrate.  

*miniatulum Fusarium* Sacc., Syll. Fung. 10: 727. 1892.

*Replaced synonym*: *Fusarium miniatum* Prill. & Delacr., Bull. Soc. Mycol. France 7: 117. 1891, *nom. illegit.*, Art. 53.1

(See *Fusarium nivale*)

*Holotypus*: Not located.

*Type locality*: **France**, Paris.

*Type substrate*: *Secale cereale*.

*Note*: Synonym *fide*Wollenweber & Reinking (1935).  

*miniatum Fusarium* Sacc., Michelia 1: 83. 1877.

*Synonym*: *Fusarium detonianum* Sacc., Syll. Fung. 4: 708. 1886, *nom. illegit.*, Art. 52.1.

*Holotypus*: In PAD.

*Type locality*: **Italy**.

*Type substrate*: Sporangium of *Cyathus vernicosa*.

*Note*: Status unclear. Requires further investigation.  

*miniatum Fusarium* (Berk. & M.A. Curtis) Sacc., Syll. Fung. 4: 722. 1886, *nom. illegit.*, Art. 53.1.

*Basionym*: *Fusisporium miniatum* Berk. & M.A. Curtis, Grevillea 3: 147. 1875.

(See ***Fusarium lateritium***)

*Holotypus*: ?K(M).

*Type locality*: **USA**, North Carolina.

*Type substrate*: *Cornus florida*.

*Note*: Synonym *fide*Wollenweber & Reinking (1935).  

*miniatum Fusarium* Prill. & Delacr., Bull. Soc. Mycol. France 7: 117. 1891, *nom. illegit.*, Art. 53.1.

*Replacing synonym*: *Fusarium miniatulum* Sacc., Syll. Fung. 10: 727. 1892

(See *Fusarium nivale*)

*Authentic material*: Not located.

*Original locality*: **France**, Paris.

*Original substrate*: *Secale cereale*.

*Note*: Synonym *fide*Wollenweber & Reinking (1935).  

*minimum Fusarium* Fuckel, Fungi Rhen. Exs., Fasc. 3, no. 213. 1863.

(See *Fusarium nivale*)

*Syntypes*: In BPI, MICH, MU & S (Fungi Rhen. Exs., Fasc. 3, no. 213).

*Type locality*: **Germany**, Oestrich, Nassau region.

*Type substrate*: Dry leaves of *Poaceae* (mainly *Zea mays*)

*Note*: Synonym *fide*Wollenweber & Reinking (1935).  

*minutissimum Fusarium* (Desm.) Sacc., Syll. Fung. 4: 703. 1886.

***Passalora minutissima*** (Desm.) U. Braun & Crous, CBS Biodiversity Ser. 1: 276. 2003.

*Basionym*: *Selenosporium minutissimum* Desm., Pl. Crypt. France, ed. 3, Fasc. 10: no. 456. 1857.

*Phaeoramularia minutissima* (Desm.) U. Braun, Nova Hedwigia 55: 214. 1992.

*Ramularia geranii-sanguinei* C. Massal., Atti Ist. Veneto Sci. Lett. Arti 59: 688. 1900.

*Cercospora geranii-sanguinei* Henn., Nytt Mag. Naturvidensk. 42: 33. 1904.

*Lectotypus*: Desm., Pl. Crypt. France, Fasc. X, no. 456 in PC *fide*Braun (1998).

*Lectotype locality*: **France**, Louvigny, Caen.

*Lectotype substrate*: *Geranium molle*.  

*minutulum Fusarium* Corda, Icon. Fung. 2: 4. 1838.

?***Clonostachys solani*** (Harting) Schroers & W. Gams, Stud. Mycol. 46: 111. 2001.

*Basionym*: *Spicaria solani* Harting, Nieuwe Verh. Eerste Kl. Kon. Ned. Inst. Wetensch. Amsterdam, ser. 2, 12: 226. 1846.

*Synonyms*: ?*Gliocladium solani* (Harting) Petch, Trans. Brit. Mycol. Soc. 27: 149. 1945.

?*Hypomyces solani* Reinke & Berthold, Untersuch. Bot. Lab. Univ. Göttingen 1: 27. 1879.

?*Hypolyssus solani* (Reinke & Berthold) Kuntze, Revis. Gen. Pl. 3: 488. 1898.

?*Hyphonectria solani* (Reinke & Berthold) Petch, Bot. J. (London) 74: 220. 1937 [1936].

?*Nectriopsis solani* (Reinke & Berthold) C. Booth, Mycol. Pap. 74: 8. 1960.

?*Bionectria solani* (Reinke & Berthold) Schroers, Stud. Mycol. 46: 111. 2001.

?*Gliocladium nigrovirens* J.F.H. Beyma, Verh. Kon. Akad. Wetensch., Afd. Natuurk., Sect. 2, 29: 30. 1931.

?*Clonostachys solani f. nigrovirens* (J.F.H. Beyma) Schroers, Stud. Mycol. 46: 115. 2001.

*Typus*: In PRM *fide*Pilat (1938).

*Type locality*: **Czech Republic**, Prague.

*Type substrate*: Wood splinters of *Corylus* sp.

*Notes*: Synonym *fide*Wollenweber & Reinking (1935). Lectotypification pending study of material lodged in PRM.  

***miscanthi Fusarium*** W. Gams *et al.*, Mycologia 91: 264. 1999.

*Holotypus*: CBS H-6063.

*Ex-type culture*: CBS 577.97 = NRRL 26231.

*Type locality*: **Denmark**, Zealand, Højbakkegård Experimental field.

*Type substrate*: *Miscanthus sinensis.*

*Descriptions and illustrations*: See Gams *et al.* (1999).

*Diagnostic DNA barcodes*: *rpb1*: JX171521; *rpb2*: JX171634; *tef1*: MN193878.  

*mollerianum Fusarium* Thüm., Inst. Coimbra 28: 263. 1881.

(See ***Fusarium graminearum***)

*Holotypus*: ?S-F45644.

*Type locality*: **Portugal**, Coimbra.

*Type substrate*: *Melia azedarach.*

*Note*: Synonym *fide*Wollenweber & Reinking (1935).  

*moniliforme Fusarium* J. Sheld., Annual Rep. Nebraska Agric. Exp. Sta. 17: 23. 1904.

(See ***Fusarium verticillioides***)

*Syntypes*: BPI 452450 & BPI 452452.

*Type locality*: **USA**, Nebraska.

*Type substrate*: *Zea mays.*

*Note*: Typification pending further study of the syntypes.  

***monophialidicum Fusarium*** J.W. Xia *et al.*, Persoonia 43: 211. 2019.

*Holotypus*: CBS H-24063.

*Ex-type culture*: NRRL 54973 = UTHSC 06-1473.

*Type locality*: **USA**, Ohio.

*Type substrate*: Eye of *Rhinocerotidae* (rhinoceros)*.*

*Descriptions and illustrations*: See Xia *et al.* (2019).

*Diagnostic DNA barcodes*: *rpb1*: KC808299; *rpb2*: KC808362; *tef1*: MN170483.  

*mori Fusarium* (Sand.-Den. & Crous) O'Donnell *et al.*, Index Fungorum 440: 2. 2020.

***Neocosmospora mori*** Sand.-Den. & Crous, Persoonia 43: 143. 2019.

*Holotypus*: CBS H-23987.

*Ex-type culture*: ATCC 44934 = CBS 145467 = MAFF 238539 = NRRL 22230.

*Type locality*: **Japan**, Miyazaki.

*Type substrate*: Twigs of *Morus alba*.

*Descriptions and illustrations*: See Sandoval-Denis *et al.* (2019).

*Diagnostic DNA barcodes*: *rpb1*: MW834235; *rpb2*: EU329499; *tef1*: AF178358.  

*moronei Fusarium* Curzi, Revista Biol. (Lisbon) 10: 141. 1928.

(See ***Fusarium acuminatum***)

*Holotypus*: *?*PAV.

*Type locality*: **Italy**.

*Type substrate*: Vesicle on skin of *Canis lupus familiaris* (dog).

*Note*: Synonym *fide*Wollenweber & Reinking (1935).  

*moschatum Fusarium* (Kitasato) Sacc., Syll. Fung. 10: 729. 1892.

*Basionym*: *Fusisporium moschatum* Kitasato, Centralbl. Bakteriol. Parasitenk., 1. Abth. 5: 365. 1889.

(See *Fusarium aquaeductuum*)

*Holotypus*: Not located.

*Type locality*: **Germany**.

*Type substrate*: Metallic medical equipment.

*Note*: Synonym *fide*Wollenweber & Reinking (1935).  

***mucidum Fusarium*** J.W. Xia *et al.*, Persoonia 43: 211. 2019.

*Holotypus*: CBS H-24064.

*Ex-type culture*: CBS 102395.

*Type locality*: **El Salvador**, Cooperación Coralama.

*Type substrate*: Mouldy nut of *Anacardium occidentale.*

*Descriptions and illustrations*: See Xia *et al.* (2019).

*Diagnostic DNA barcodes*: *rpb2*: MN170418; *tef1*: MN170485.  

*muentzii Fusarium* Delacr. (as ‘*müntzii*’), Bull. Soc. Mycol. France 8: 192. 1892.

(See ***Fusarium tricinctum***)

*Lectotypus* (*hic designatus*, MBT 10000715): **France**, Paris, on animal waste, May 1891, *G. Delacroix*, Bull. Soc. Mycol. France 8, pl. XVII, fig. V.  

***multiceps Fusarium*** J.W. Xia *et al.*, Persoonia 43: 212. 2019.

*Holotypus*: CBS H-24065.

*Ex-type culture*: CBS 130386 = NRRL 43639 = UTHSC 04-135.

*Type locality*: **USA**, Florida.

*Type substrate*: *Trichechus* sp*.*

*Descriptions and illustrations*: See Xia *et al.* (2019).

*Diagnostic DNA barcodes*: *rpb1*: HM347190; *rpb2*: GQ505844; *tef1*: GQ505666.  

***mundagurra Fusarium*** M.H. Laurence *et al.*, Fungal Diversity 77: 359. 2015 [2016].

*Holotypus*: RBG5717.

*Ex-type culture*: NRRL 66235 = RBG5717.

*Type locality*: **Australia**, Queensland, Carnarvon Gorge National Park.

*Type substrate*: Soil*.*

*Descriptions and illustrations*: See Laurence *et al.* (2016).

*Diagnostic DNA barcodes*: *rpb1*: KP083272; *rpb2*: KP083276; *tef1*: KP083256.  

***musae Fusarium*** Van Hove *et al.*, Mycologia 103: 579. 2011.

*Synonym*: *Gibberella musae* Van Hove *et al.*, Mycologia 103: 577. 2011.

*Holotypus*: MUCL 52574.

*Ex-type culture*: CBS 624.87 = MUCL 52574 = NRRL 25059.

*Type locality*: **Honduras**.

*Type substrate*: *Musa* sp*.*

*Descriptions and illustrations*: See Van Hove *et al.* (2011).

*Diagnostic DNA barcodes*: *rpb1*: MW402689; *rpb2*: FN552108; *tef1*: FN552086.  

***musarum Fusarium*** Logrieco & Marasas, Mycologia 90: 510. 1998.

*Holotypus*: BPI 802928.

*Ex-type culture*: FRC R-9400 = MRC 6240 = NRRL 28507.

*Type locality*: **Panama**.

*Type substrate*: *Musa sapientum.*

*Descriptions and illustrations*: See Marasas *et al.* (1998).

*Diagnostic DNA barcodes*: *rpb1*: MW233265*; rpb2*: MW928829; *tef1*: MW233094.  

*mycophilum Fusarium* (P. Karst.) Sacc., Syll. Fung. 10: 730. 1892.

*Basionym*: *Leptosporium mycophilum* P. Karst., Meddel. Soc. Fauna Fl. Fenn. 16: 24. 1888.

*Holotypus*: ?H.

*Type locality*: **Finland**, Merimasku.

*Type substrate*: *Myxogastria*.

*Note*: *Hymenula fide*Wollenweber & Reinking (1935).  

*mucophytum Fusarium* (W.G. Sm.) Massee, Brit. Fung.-Fl. 3: 483. 1893.

*Basionym*: *Fusisporium mucophytum* W.G. Sm., Gard. Chron. n.s., 22: 245. 1884.

(See ***Fusarium scirpi***)

*Holotypus*: ?K(M).

*Type locality*: **UK**.

*Type substrate*: *Agaricus arvensis*.

*Note*: Synonym *fide*Wollenweber & Reinking (1935).  

*myosotidis Fusarium* Cooke, Grevillea 16: 49. 1887.

(See ***Fusarium oxysporum***)

*Holotypus*: In K(M).

*Type locality*: **UK**, Forden.

*Type substrate*: *Myosotis* sp.

*Note*: Synonym *fide*Wollenweber & Reinking (1935).  

***nanum Fusarium*** M.M. Wang *et al.*, Persoonia 43: 85. 2019.

*Holotypus*: HAMS 248043.

*Ex-type culture*: CGMCC 3.19498 = LC12168.

*Type locality*: **China**, Guangxi Province, Guilin.

*Type substrate*: Leaves of *Musa nana.*

*Descriptions and illustrations*: See Wang *et al.* (2019).

*Diagnostic DNA barcodes*: *rpb1*: MK289871; *rpb2*: MK289755; *tef1*: MK289602.  

***napiforme Fusarium*** Marasas *et al.*, Mycologia 79: 910. 1988 [1987].

*Holotypus*: DAOM 196924.

*Ex-type culture*: BBA 69861 = CBS 748.97 = DAOM 196924 = DAOM 225147 = FRC M-3563 = IMI 375353 = MRC 4144 = NRRL 13604.

*Type locality*: **Namibia**, Ovambo.

*Type substrate*: *Pennisetum typhoides.*

*Descriptions and illustrations*: See Marasas *et al.* (1987).

*Diagnostic DNA barcodes*: *rpb1*: HM347136; *rpb2*: EF470117; *tef1*: AF160266.  

*nectriae-palmicolae Fusarium* Henn., Bot. Jahrb. Syst. 23: 290. 1896.

(See ***Fusarium equiseti***)

*Holotypus*: In B *fide*Hein (1988).

*Type locality*: **Samoa**, Upolu.

*Type substrate*: Leaves of *Areca* sp.

*Note*: Synonym *fide*Wollenweber & Reinking (1935).  

*nectriae-turraeae Fusarium* Henn., Bot. Jahrb. Syst. 22: 82. 1895.

(See *Fusarium coccophilum*)

*Holotypus*: In B *fide*Hein (1988).

*Type locality*: **Tanzania**, Marangu.

*Type substrate*: *Turraea volkensii*.

*Note*: Synonym *fide*Wollenweber & Reinking (1935).  

***nectricreans Fusarium*** Kirschst., Ann. Mycol. 37: 138. 1939.

*Holotypus*: B 70 0100202.

*Type locality*: **Germany**, Berlin.

*Type substrate*: Rotting stem of garden plant.

*Note*: No living material was available for confirmation of taxonomic status.  

*nectrioides Fusarium* (Wollenw.) Schroers *et al.*, Mycologia 101: 59. 2009.

***Bisifusarium nectrioides*** (Wollenw.) L. Lombard & Crous, Stud. Mycol. 80: 225. 2015.

*Basionym*: *Fusarium dimerum* var. *nectrioides* Wollenw., Fusaria Autogr. Delin. 3: 855. 1930.

*Lectotypus*: No. 855 in Wollenweber, Fusaria Autogr. Delin. (1930), designated in Schroers *et al.* (2009).

*Ex-type culture*: CBS 176.31 = NRRL 20689.

*Lectotype and ex-type locality*: **Honduras**.

*Lectotype and ex-type substrate*: Soil*.*

*Descriptions and illustrations*: See Schroers *et al.* (2009).

*Diagnostic DNA barcodes*: *rpb1*: JX171477; *rpb2*: JX171591; *tef1*: EU926312.  

*neglectum Fusarium* Jacz., Bull. Trimestriel Soc. Mycol. France 28: 348. 1912.

(See *Fusarium culmorum*)

*Holotypus*: Not located.

*Type locality*: **Ukraine***,* Poltava.

*Type substrate*: *Zea mays*.

*Note*: Synonym *fide*Wollenweber & Reinking (1935).  

*negundinis Fusarium* Sherb., in Hubert, J. Agric. Res. 26: 451. 1923.

(See ***Fusarium reticulatum***)

*Holotypus*: Not located.

*Type locality*: **USA**, Wisconsin, Madison.

*Type substrate*: *Acer negundo*.

*Note*: Synonym *fide*Wollenweber & Reinking (1935).  

***nelsonii Fusarium*** Marasas & Logrieco, Mycologia 90: 508. 1998.

*Holotypus*: BPI 802927.

*Ex-type culture*: CBS 119876 = FRC R-8670 = MRC 4570 = NRRL 28505 = NRRL 53945.

*Type locality*: **South Africa**, Western Cape Province, Malmesbury.

*Type substrate*: Plant debris in *Triticum* soil*.*

*Descriptions and illustrations*: See Marasas *et al.* (1998).

*Diagnostic DNA barcodes*: *rpb1*: MN120722; *rpb2*: GQ505468; *tef1*: GQ505404.  

*nematophilum Fusarium* Nirenberg & Hagedorn, Nachrichtenbl. Deutsch. Pflanzenschutzdienstes 60: 214. 2008.

***Luteonectria nematophila*** (Nirenberg & Hagedorn) Sand.-Den. & L. Lombard, Stud. Mycol. 98 (no. 100116): 60. 2021.

*Holotypus*: BBA 72279 in B.

*Ex-type culture*: BBA 72279 = NRRL 54600.

*Type locality*: **Germany**, Berlin.

*Type substrate*: Isolated from soil with roots of *Hedera helix.*

*Descriptions and illustrations*: See Nirenberg & Hagedorn (2008).

*Diagnostic DNA barcodes*: *rpb1*: JX171552; *rpb2*: JX171664; *tef1*: JABFFA010003988.  

*neoceras Fusarium* Wollenw. & Reinking, Phytopathology 15: 164. 1925.

(See ***Fusarium sacchari***)

*Holotypus*: CBS 147.25 (preserved as metabolically inactive culture).

*Ex-type culture*: BBA 69863 = CBS 147.25 = DAOM 225410 = IMI 375345= NRRL 20471.

*Type locality*: **Honduras**.

*Type substrate*: Rotting *Musa sapientum.*

*Descriptions and illustrations*: See Gerlach & Nirenberg (1982).

*Diagnostic DNA barcodes*: *rpb1*: MT010941; *rpb2*: MT010962; *tef1*: MT010988.  

*neocosmosporiellum Fusarium* O'Donnell & Geiser, Phytopathology 103: 405. 2013.

***Neocosmospora vasinfecta*** E.F. Sm., Bull. Div. Veg. Physiol. Pathol. U.S.D.A. 17: 45. 1899.

*Synonyms*: *Nectriella tracheiphila* E.F. Sm., Proc. Amer. Assoc. Advancem. Sci. 44: 190. 1896, *nom. inval*. *fide* Cannon & Hawksworth 1984.

*Neocosmospora vasinfecta* var. *nivea* E.F. Sm., Bull. Div. Veg. Physiol. Pathol. U.S.D.A. 17: 45. 1899.

*Neocosmospora vasinfecta* var. *tracheiphila* E.F. Sm., Bull. Div. Veg. Physiol. Pathol. U.S.D.A. 17: 45. 1899.

*Fusarium tracheiphilum* (E.F. Sm.) Wollenw., Phytopathology 3: 29. 1913.

*Fusarium vasinfectum* var. *pisi* C.J.J. Hall, Ber. Deutsch. Bot. Ges. 21: 4. 1903.

*Neocosmospora vasinfecta* var. *pisi* (C.J.J. Hall) Sacc., Syll. Fung. 20: 192. 1911.

*Neocosmospora africana* Arx, Antonie van Leeuwenhoek 21: 161. 1955.

*Neocosmospora vasinfecta* var. *africana* (Arx) P.F. Cannon & D. Hawksw., Trans. Brit. Mycol. Soc. 82: 676. 1984.

?*Pseudonectria ornata* Bat. & Maia, Anais Soc. Biol. Pernambuco 13: 74. 1955 (*fide* Cannon & Hawksworth 1984).

*Neocosmospora vasinfecta* var. *major* P. Rama Rao, Mycopathol. Mycol. Appl. 21: 218. 1963.

*Neocosmospora ornamentata* M.A.F. Barbosa, Garcia de Orta 13: 17. 1965.

*Fusarium ornamentatum* (M.A.F. Barbosa) O'Donnell *et al.*, Index Fungorum 440: 3. 2020.

*Neocosmospora vasinfecta f. conidiifera* Kamyschko, Novosti Sist. Nizsh. Rast. 1965: 115. 1965.

*Neocosmospora boninensis* Udagawa *et al.*, Sydowia 41: 350. 1989.

*Lectotypus*: Pl. V, figs 1–2 (Smith, Bull. Div. Veg. Physiol. Pathol. U.S.D.A. 17, 1899), designated in Sandoval-Denis *et al.* (2019).

*Lectotype locality*: **USA**.

*Lectotype substrate*: *Gossypium* sp*.*

*Epitypus*: BPI 910920, designated in Aoki *et al.* (2020).

*Ex-epitype culture*: ATCC 62199 = NRRL 22166.

*Epitype locality*: **USA**, Illinois, southern area.

*Epitype substrate*: A cyst of *Heterodera glycines* in a soil sample from soybean field.

*Diagnostic DNA barcodes*: *rpb1*: SSHR01002742; *rpb2*: EU329497; *tef1*: AF178350.  

***neoscirpi Fusarium*** L. Lombard *et al.*, Persoonia 43: 213. 2019.

*Holotypus*: CBS H-24066.

*Ex-type culture*: CBS 610.95 = NRRL 26861 = NRRL 26922.

*Type locality*: **France**.

*Type substrate*: Soil*.*

*Descriptions and illustrations*: See Xia *et al.* (2019).

*Diagnostic DNA barcodes*: *rpb2*: GQ505779; *tef1*: GQ505601.  

***neosemitectum Fusarium*** L. Lombard *et al.*, Persoonia 43: 214. 2019.

*Holotypus*: CBS H-24067.

*Ex-type culture*: CBS 189.60.

*Type locality*: **Democratic Republic of the Congo**.

*Type substrate*: *Musa sapientum.*

*Descriptions and illustrations*: See Xia *et al.* (2019).

*Diagnostic DNA barcodes*: *rpb2*: MN170422; *tef1*: MN170489.  

***nepalense Fusarium*** T. Aoki *et al.*, Fungal Genet. Biol. 48: 1105. 2011.

*Holotypus*: BPI 881006.

*Ex-type culture*: CBS 127503 = NRRL 54222.

*Type locality*: **Nepal**.

*Type substrate*: *Oryza sativa.*

*Descriptions and illustrations*: See Sarver *et al.* (2011).

*Diagnostic DNA barcodes*: *rpb1*: KM361650; *rpb2*: KM361668; *tef1*: KM889631.  

*nervisequum Fusarium* (Fuckel) Fuckel, Jahrb. Nassauischen Vereins Naturk. 23–24: 369. 1870.

*Basionym*: *Labrella nervisequa* Fuckel, Fungi Rhen. Exs., Fasc. 5, no. 427. 1863.

***Apiognomonia platani*** (Lév.) L. Lombard, ***comb. nov*.** MycoBank MB 837698.

*Basionym*: *Hymenula platani* Lév., Ann. Sci. Nat., Bot., sér. 3, 9: 128. 1848.

*Synonyms*: *Fusarium platani* (Lév.) Mont., Ann. Sci. Nat., Bot., sér. 3, 11: 55. 1849.

*Fusarium nervisequum f. platani* (Lév.) Fuckel, Jahrb. Nassauischen Vereins Naturk. 23–24: 369. 1870.

*Gloeosporidium platani* (Lév.) Höhn., Sitzungsber. Kaiserl. Akad. Wiss. Wien, Math.-Naturwiss. Cl., Abt. 1, 125: 95. 1916.

*Myxosporina platani* (Lév.) Höhn., Hedwigia 62: 48. 1920, *nom. inval*., Art. 35.1.

*Gloeosporium nervisequum* (Fuckel) Sacc., Syll. Fung. 3: 711. 1884.

*Discula nervisequa* (Fuckel) M. Morelet, Bull. Soc. Sci. Nat. Archéol. Toulon & Var 203: 12. 1973.

*Gloeosporium platani* Oudem., Ned. Kruidk. Arch., sér. 2, 1: 258. 1873.

*Laestadia veneta* Sacc. & Speg., Michelia 1: 351. 1878.

*Carlia veneta* (Sacc. & Speg.) Kuntze, Revis. Gen. Pl. 2: 846. 1891.

*Apiospora veneta* (Sacc. & Speg.) Sacc. ex Kleb., Z. Pflanzenkrankh. 12: 258. 1902.

*Gnomonia veneta* (Sacc. & Speg.) Kleb., Jahrb. Wiss. Bot. 41: 533. 1905, *nom. illegit.*, Art. 53.1.

*Gnomonia platani* Kleb., Verhandl. Deutsch. Bot. Ges. 1: 28. 1914.

*Guignardia veneta* (Sacc. & Speg.) Traverso, Fl. Ital. Crypt. 1: 392. 1907.

*Apiosporopsis veneta* (Sacc. & Speg.) Traverso, Syll. Fung. 22: 78. 1913.

*Apiognomonia veneta* (Sacc. & Speg.) Höhn., Ann. Mycol. 16: 51. 1918.

*Laestadia veneta* var. *cylindrasca* Sacc. & Speg., Michelia 1: 369. 1878.

*Laestadia cylindrasca* (Sacc. & Speg.) Sacc., Syll. Fung. 1: 422. 1882.

*Carlia cylindrasca* (Sacc. & Speg.) Kuntze, Revis. Gen. Pl. 2: 846. 1891.

*Guignardia cylindrasca* (Sacc. & Speg.) Lindau (as ‘*cylindracea*’), in Engler & Prantl, Nat. Pflanzenfam., Teil. I, 1(1): 422. 1897.

*Diaporthe veneta* Sacc. & Speg., Michelia 1: 383. 1878.

*Discella platani* Peck, Rep. (Annual) New York State Mus. Nat. Hist. 29: 49. 1878, *nom. illegit.*, Art. 53.1.

*Discula platani* Sacc., Syll. Fung. 3: 674. 1884.

*Sporonema platani* Bäumler, Oesterr. Bot. Z. 40: 17. 1890.

*Placosphaeria platani* (Bäumler) Limber, Mycologia 47: 398. 1955.

*Myxosporium platanicola* Ellis & Everh. (as ‘*platanicolum*’), Proc. Acad. Nat. Sci. Philadelphia 46: 372. 1894.

*Cryptosporiopsis platanicola* (Ellis & Everh.) G.F. Laundon, CBS List of Cultures (Baarn): (1). 1975.

*Gloeosporidina platani* Butin & Kehr, Eur. J. Forest Pathol. 28: 299. 1998.

*Lectotypus*: BPI (Fuckel, Fungi Rhen. 427) of *Labrella nervisequa* Fuckel, designated in Sogonov *et al.* (2007).

*Lectotype locality*: **Germany**, Reichartshausen.

*Lectotype substrate*: *Plantanus orientalis*.

*Epitypus*: BPI 871953, designated in Sogonov *et al.* (2007).

*Epitype locality*: **Switzerland**, Geneva.

*Epitype substrate*: *Plantanus orientalis.*

*Notes*: Based on priority and synonymies proposed by Sogonov *et al.* (2007), the name *Hymenula platani* Lév. (1848) takes precedence over *Laestadia veneta* Sacc. & Speg. (1878). Therefore, a new combination is proposed here applying the older name.  

***newnesense Fusarium*** M.H. Laurence *et al.*, Fungal Diversity 77: 360. 2015 [2016].

*Holotypus*: RBG 610.

*Ex-type culture*: NRRL 66241 = RBG 610.

*Type locality*: **Australia**, New South Wales, Newnes State Forest.

*Type substrate*: Soil*.*

*Descriptions and illustrations*: See Laurence *et al.* (2016).

*Diagnostic DNA barcodes*: *rpb1*: JABCJW010000176; *rpb2*: JABCJW010000963; *tef1*: KP083261.  

*ngaiotongaense Fusarium* O'Donnell *et al.*, Index Fungorum 440: 3. 2020.

***Neocosmospora longissima*** Sand.-Den. & Crous, Persoonia 43: 141 (2019).

*Holotypus*: CBS H-23985.

*Ex-type culture*: CBS 126407 = G.J.S. 85-72.

*Type locality*: **New Zealand**, Russell State Forest, Ngaiotonga Scenic Reserve.

*Type substrate*: From tree bark*.*

*Descriptions and illustrations*: See Sandoval-Denis *et al.* (2019).

*Diagnostic DNA barcodes*: *rpb1*: MW834230; *rpb2*: LR583846; *tef1*: LR583621.  

*nicotianae Fusarium* Oudem., Ned. Kruidk. Arch., sér. 3, 2: 777. 1902.

(See *Fusarium merismoides*)

*Holotypus*: ?L.

*Type locality*: **Netherlands**, Noord-Holland Province, Bussum.

*Type substrate*: *Nicotiana tabacum*.

*nigrum Fusarium* O.A. Pratt, J. Agric. Res. 13: 90. 1918.

(See ***Fusarium flocciferum***)

*Lectotypus* (*hic designatus*, MBT 10000716): **USA**, Idaho, from soil, 1918, O.A. Pratt, in J. Agric. Res. 13: 82, fig. 1J–L.

*Notes*: Synonym *fide*Wollenweber & Reinking (1935). As the holotype specimen was not located, an illustration accompanying the original protologue is designated here as lectotype.  

***nirenbergiae Fusarium*** L. Lombard & Crous, Persoonia 43: 29. 2018 [2019].

*Holotypus*: CBS H-23619.

*Ex-type culture*: CBS 840.88.

*Type locality*: **Netherlands**, Noord-Holland Province, Aalsmeer.

*Type substrate*: *Dianthus caryophyllus.*

*Descriptions and illustrations*: See Lombard et al., 2019b, Lombard et al., 2019a.

*Diagnostic DNA barcodes*: *rpb2*: MH484887; *tef1*: MH484978.  

***nisikadoi Fusarium*** T. Aoki & Nirenberg, Mycoscience 38: 330. 1997.

*Holotypus*: BBA 69015 in B.

*Ex-type culture*: BBA 69015 = CBS 456.97 = MAFF 237506 = NRRL 25205 = NRRL 25308.

*Type locality*: **Japan**, Oita, Hita.

*Type substrate*: *Triticum aestivum.*

*Descriptions and illustrations*: See Nirenberg & Aoki (1997).

*Diagnostic DNA barcodes*: *rpb1*: MG282391; *rpb2*: MG282421; *tef1*: KR909358.  

*nitidum Fusarium* Berk. & M.A. Curtis, Grevillea 3: 98. 1875.

*Holotypus*: ?K(M).

*Type locality*: **USA**, Pennsylvania, Michener.

*Type substrate*: *Aralia spinosa*.

*Note*: Doubtful species *fide*Wollenweber & Reinking (1935).  

*nivale Fusarium* Ces. ex Berl. & Voglino, Syll. Fung., Addit. I–IV: 390. 1886.

***Microdochium nivale*** (Fr.) Samuels & I.C. Hallett, Trans. Brit. Mycol. Soc. 81: 479. 1983.

*Basionym*: *Lanosa nivalis* Fr., Summa Veg. Scand. 2: 495. 1849.

*Synonyms*: *Fusarium nivale* (Fr.) Sorauer, Z. Pflanzenkrankh. 11: 220. 1901, *nom. illegit.*, Art. 53.1.

*Fusarium hibernans* Lindau, Rabenh. Krypt.-Fl., ed. 2, 1(9): 542. 1909, *nom. superfl*., Art. 52.1.

*Gerlachia nivalis* (Ces. ex Berl. & Voglino) W. Gams & E. Müll., Netherlands J. Pl. Pathol. 86: 49. 1980.

*Fusarium minimum* Fuckel, Fungi Rhen. Exs., Fasc. 3, no. 213. 1863.

*Fusarium ustilaginis* Rostr., Bot. Foren. Festskr. 54: 137. 1890, *nom. illegit.*, Art. 53.1.

*Fusarium miniatum* Prill. & Delacr., Bull. Soc. Mycol. France 7: 117. 1891, *nom. illegit.*, Art. 53.1.

*Fusarium tritici* Erikss., Fungi Paras. Scand. Exs. no. 400. 1891, *nom. illegit*., Art. 53.1.

*Fusarium miniatulum* Sacc., Syll. Fung. 10: 727. 1892.

*Nectria pseudograminicola* Weese, Ann. Mycol. 8: 466. 1910, *nom. inval*., Art. 38.1.

*Fusarium loliaceum* Ducomet, Ann. École Natl. Agric. Rennes 2: 14. 1909.

*Fusarium secalis* Jacz., Bull. Trimestriel Soc. Mycol. France 28: 346. 1912, *nom. illegit.*, Art. 53.1.

*Sphaerulina divergens* Rehm, Ann. Mycol. 11: 397. 1913.

*Monographella divergens* (Rehm) Petr., Ann. Mycol. 22: 144. 1924.

*Calonectria nivalis* Schaffnit, Mycol. Centralbl. 2: 257. 1913.

*Griphosphaeria nivalis* (Schaffnit) E. Müll. & Arx, Phytopathol. Z. 24: 356. 1955.

*Micronectriella nivalis* (Schaffnit) C. Booth, The Genus Fusarium: 42. 1971.

*Monographella nivalis* (Schaffnit) E. Müll., Rev. Mycol. (Paris) 41: 132. 1977.

*Calonectria graminicola* F. Stevens, Bot. Gaz. 65: 232. 1918, *nom. illegit.*, Art. 53.1.

*Melioliphila graminicola* Speg., Bol. Acad. Ci. (Córdoba) 26: 344. 1921.

*Calonectria graminicola* var. *neglecta* Krampe, Angew. Bot. 8: 252. 1926.

*Monographella nivalis* var. *neglecta* (Krampe) Gerlach, Netherlands J. Pl. Pathol. 86: 49. 1980.

*Fusarium nivale* var. *oryzae* Zambett., Mitt. Inst. Colombo-Aleman Invest. Ci. 30: 489. 1950, *nom. inval*., Art. 39.1.

*Syntypes*: In HAL & ILL [Rabenhorst, Klotzschii Herb. Viv. Mycol. no. 1439 (sub *F. oxysporum*)].

*Type locality*: **Italy**.

*Type substrate*: *Poaceae*.  

*niveum Fusarium* E.F. Sm., Proc. Amer. Assoc. Advancem. Sci. 43: 289. 1894, *nom. inval*., Art. 36.1(a).

(See ***Fusarium oxysporum***)

*Authentic material*: Not located.

*Original locality*: **USA**.

*Original substrate*: *Citrullus vulgaris*.

*niveum Fusarium* McAlpine, Australas. J. Pharm. 17: 3. 1902.

*Note*: Unable to locate protologue.  

***nodosum Fusarium*** L. Lombard & Crous, Fungal Syst. Evol. 4: 193. 2019.

*Holotypus*: CBS H-24018.

*Ex-type culture*: CBS 201.63.

*Type locality*: **Portugal**, Lisbon.

*Type substrate*: Seed of *Arachis hypogaea.*

*Descriptions and illustrations*: See Lombard *et al.* (2019a).

*Diagnostic DNA barcodes*: *rpb1*: MN120725; *rpb2*: MN120743; *tef1*: MN120763.  

*noneumartii Fusarium* (Sand.-Den. & Crous) O'Donnell *et al.*, Index Fungorum 440: 3. 2020.

***Neocosmospora noneumartii*** Sand.-Den. & Crous, Persoonia 43: 145. 2019.

*Holotypus*: CBS H-23989.

Ex-type culture: CBS 115658 = FRC S-0661.

*Type locality*: **Israel**, Palestine.

*Type substrate*: *Solanum tuberosum*.

*Descriptions and illustrations*: See Sandoval-Denis *et al.* (2019).

*Diagnostic DNA barcodes*: *rpb1*: MW218129; *rpb2*: MW446618; *tef1*: LR583630.  

*nucicola Fusarium* P. Karst. & Har., Rev. Mycol. (Toulouse) 12: 131. 1890.

(See ***Fusarium lateritium***)

*Holotypus*: ?UPS *fide* Wollenweber, Fusaria Autogr. Delin. 1: 236. 1916.

*Type locality*: **France**.

*Type substrate*: Epicarp of nut.

*Note*: Synonym *fide*Wollenweber & Reinking (1935).  

***nurragi Fusarium*** (Summerell & L.W. Burgess) Benyon *et al.*, Mycol. Res. 104: 1171. 2000.

*Basionym*: *Fusarium avenaceum* subsp. *nurragi* Summerell & L.W. Burgess, Mycol. Res. 99: 289. 1995.

*Holotypus*: DAR 69502.

*Ex-type culture*: CBS 393.96 = DAR 69501 = F10108 = F11121.

*Type locality*: **Australia**, Victoria, Wilson's Promontory National Park.

*Type substrate*: Soil*.*

*Descriptions and illustrations*: See Sangalang *et al.* (1995).

*Diagnostic DNA barcodes*: *rpb1*: MW928814; *rpb2*: MW928830; *tef1*: MW928840.  

***nygamai Fusarium*** L.W. Burgess & Trimboli, Mycologia 78: 223. 1986.

*Synonym*: *Gibberella nygamai* Klaasen & P.E. Nelson, Mycologia 88: 967. 1997.

*Holotypus*: FRC-M-1375.

*Ex-type culture*: ATCC 58555 = BBA 69862 = CBS 749.97 = FRC M-1375 = IMI 375354 = NRRL 13448.

*Type locality*: **Australia**, New South Wales, Narrabri.

*Type substrate*: Necrotic roots of *Sorghum* sp*.*

*Descriptions and illustrations*: See Burgess & Trimboli (1986).

*Diagnostic DNA barcodes*: *rpb1*: LT996202; *rpb2*: KU604262; *tef1*: MT011009.  

*obliquiseptatum Fusarium*, T. Aoki *et al.*, Mycologia 111: 929. 2019.

***Neocosmospora obliquiseptata*** (T. Aoki *et al.*) L. Lombard & Sand.-Den., ***comb. nov*.** MycoBank MB 837699.

*Basionym*: *Fusarium obliquiseptatum*, T. Aoki *et al.*, Mycologia 111: 929. 2019.

*Holotypus*: BPI 910970.

*Ex-type culture*: MAFF 246845 = NRRL 62611.

*Type locality*: **Australia**, Queensland, Beerwah.

*Type substrate*: A gallery wall of an ambrosia beetle (*Euwallacea* sp.) infecting *Persea americana*.

*Descriptions and illustrations*: See Aoki *et al.* (2019).

*Diagnostic DNA barcodes*: *rpb1*: KC691606; *rpb2*: KC691637, KC691666; *tef1*: KC691535.

*Note*: A new combination is provided in the genus *Neocosmospora* based on the phylogenetic relationship (Aoki *et al.* 2019) and morphology.  

*oblongum Fusarium* (Sand.-Den. & Crous) O'Donnell *et al.*, Index Fungorum 440: 3. 2020.

***Neocosmospora oblonga*** Sand.-Den. & Crous, Persoonia 43: 148. 2019.

*Holotypus*: CBS H-23990.

*Ex-type culture*: CBS 130325 = CDC B-4701= NRRL 28008.

*Type locality*: **USA**.

*Type substrate*: Eye of *Homo sapiens*.

*Descriptions and illustrations*: See Sandoval-Denis *et al.* (2019).

*Diagnostic DNA barcodes*: *rpb1*: MW834239; *rpb2*: LR583853; *tef1*: LR583631.  

*obtusatum Fusarium* Corda, Icon. Fung. 1: 3. 1837.

(See *Fusarium tortuosum*)

*Typus*: In PRM *fide*Pilat (1938).

*Type locality*: **Czech Republic**, Liberec (Reichenberg).

Type substrate: Branches of trees and shrubs.

*Note*: Not *Fusarium fide*Wollenweber & Reinking (1935). Lectotypification pending study of material lodged in PRM.  

*obtusisporum Fusarium* Cooke & Harkn., Grevillea 12: 97. 1884.

***Neonectria obtusispora*** (Cooke & Harkn.) Rossman *et al.*, Phytopathol. Medit. 53: 529. 2014.

*Synonyms*: *Cylindrocarpon obtusisporum* (Cooke & Harkn.) Wollenw., Fusaria Autogr. Delin. 1: 465. 1916.

*Ramularia obtusispora* (Cooke & Harkn.) Wollenw., Fusaria Autogr. Delin. 1: 465. 1916.

*Fusarium lineare* Moesz, Bot. Közlem. 19: 57. 1920.

*Holotypus*: K(M) 128869.

*Type locality*: **USA**, California.

*Type substrate*: Twigs of *Acacia* sp.  

*obtusiusculum Fusarium* Sacc., Michelia 2: 297. 1881.

(See *Fusarium candidum* Ehrenb.)

*Holotypus*: In PAD.

*Type locality*: **Italy**, Padua.

*Type substrate*: *Nelumbium* sp.  

*obtusum Fusarium* (Cooke) Sacc., Syll. Fung. 4: 708. 1886.

*Basionym*: *Fusisporium obtusum* Cooke, Grevillea 5: 58. 1876.

***Mycogloea macrospora*** (Berk. & Broome) McNabb, Trans. Brit. Mycol. Soc. 48: 187. 1965.

*Basionym*: *Dacrymyces macrosporus* Berk. & Broome, Ann. Mag. Nat. Hist., ser. 4, 11: 343. 1873.

*Holotypus*: In K(M) *fide* Index Fungorum.

*Type locality*: **UK**, Scotland, Forres.

*Type substrate*: *Diatrype* sp.  

*ochraceum Fusarium* (Mont.) Sacc., Syll. Fung. 4: 722. 1886.

*Basionym*: *Fusisporium ochraceum* Mont., Ann. Sci. Nat., Bot., sér. 2, 3: 355. 1835.

*Holotypus*: In ?PC.

*Type locality*: **Chile**, Juan Fernández Islands.

*Type substrate*: Bark.

*Note*: Not *Fusarium fide*Wollenweber & Reinking (1935).  

***odoratissimum Fusarium*** Maryani *et al.*, Stud. Mycol. 92: 159. 2019.

*Synonym*: *Fusarium purpurascens* Maryani *et al.*, Stud. Mycol*.* 92: 160. 2018 [2019a].

*Holotypus*: InaCC F822 (preserved as metabolically inactive culture).

*Ex-type culture*: InaCC F822.

*Type locality*: **Indonesia**, East Kalimantan, Kampung Salak Martadinata.

*Type substrate*: *Musa* sp. cv. Pisang Kepok*.*

*Descriptions and illustrations*: See Maryani *et al.* (2019a).

*Diagnostic DNA barcodes*: *rpb1*: LS479618; *rpb2*: LS479386; *tef1*: LS479828.

*Notes*: Re-analysis of the sequence data set of Maryani *et al.* (2019a) revealed that the ex-type strain of *F. purpurascens* (InaCC F971) clustered within the *F. odoratissimum* clade. Therefore, we consider *F. purpurascens* a synonym of *F. odoratissimum*.  

*oidioides Fusarium* Speg., Rev. Mycol. (Toulouse) 8: 183. 1886.

*Holotypus*: In LPS (Fungi Japon. No. 2) *fide*Farr (1973).

*Type locality*: **Japan**, Tokyo.

*Type substrate*: *Fallopia multiflora*.

*Note*: Not *Fusarium fide*Wollenweber & Reinking (1935).  

*oligoseptatum Fusarium* T. Aoki *et al.*, Fung. Syst. Evol. 1: 29. 2018.

***Neocosmospora oligoseptata*** (T. Aoki *et al.*) Sand.-Den. & Crous, Persoonia 43: 149. 2019.

*Holotypus*: BPI 910525.

*Ex-type culture*: CBS 143241 = FRC S-2581 = MAFF 246283 = NRRL 62579.

*Type locality*: **USA**, Pennsylvania, Dauphin.

*Type substrate*: From a live female ambrosia beetle (*Euwallacea validus*), extracted from a gallery in a tree-of-heaven (*Ailanthus altissima*)*.*

*Descriptions and illustrations*: See Aoki *et al.* (2018).

*Diagnostic DNA barcodes*: *rpb1*: KC691596; *rpb2*: KC691627, KC691656; *tef1*: KC691538.  

***ophioides Fusarium*** A. Jacobs, *et al.*, Persoonia 46: 149. 2021.

*Holotypus*: CBS H-24659.

*Ex-type culture*: CBS 118512 = FCC 2979 = FCC 2980 = MRC 6744.

*Type locality*: **South Africa**, Mpumulanga Province, Ngodwana.

*Type substrate*: *Panicum maximum*.

*Descriptions and illustrations*: See Yilmaz *et al.* (2021).

*Diagnostic DNA barcodes*: *rpb2*: MN534303; *tef1*: EU921239.  

*opuli Fusarium* Oudem., Hedwigia 37: 318. 1898.

*Holotypus*: ?L.

*Type locality*: **Netherlands**, Gelderland Province, Nunspeet.

*Type substrate*: *Viburnum opulus*.

*Note*: Not *Fusarium fide*Wollenweber & Reinking (1935).  

*opuntiarum Fusarium* Speg., Anales Mus. Nac. Hist. Nat. Buenos Aires 6: 350. 1898 [1899].

(See ***Fusarium oxysporum***)

*Holotypus*: In LPS (Fungi Argent. n.v.c. no. 866) *fide*Farr (1973).

*Type locality*: **Argentina**, La Plata.

*Type substrate*: Branches of *Opuntia* sp.

*Note*: Synonym *fide*Wollenweber & Reinking (1935).  

*orchidis Fusarium* Petch, Ann. Roy. Bot. Gard. (Peradeniya) 6: 256. 1917.

(See ***Fusarium reticulatum***)

*Holotypus*: PDA 4798.

*Type locality*: **Sri Lanka**.

*Type substrate*: Leaves of *Orchidaceae*.

*Note*: Synonym *fide*Wollenweber & Reinking (1935).  

*ornamentatum Fusarium* (M.A.F. Barbosa) O'Donnell *et al.*, Index Fungorum 440: 3. 2020.

(See *Fusarium neocosmosporiellum*)

*Holotypus*: CBS 562.70 (preserved as metabolically inactive culture).

*Ex-type culture*: ATCC 32363 = CBS 562.70 = IMI 251387.

*Type locality*: **Guinea-Bissau**.

*Type substrate*: Stored nuts of *Arachis hypogaea*.

*Descriptions and illustrations*: See Sandoval-Denis *et al.* (2019).

*Diagnostic DNA barcodes*: *rpb2*: LR583901; *tef1*: DQ247606.

*Note*: Synonym *fide*Sandoval-Denis *et al.* (2019).  

*orobanches Fusarium* Jacz., Ezhegodnik Svedeniy Boleznykh i Povrezhdeniyakh Kult'turnykh i Dikorastushchikh Poleznykh Rasteniy. Pertograd. 6: 190. 1910 [1912].

*Holotypus*: Not located.

*Type locality*: **Russia**, Saratov.

*Type substrate*: *Orobanche* sp.

*Notes*: Status unclear. Could be a synonym of *F. oxysporum*.  

*orthoceras Fusarium* Appel & Wollenw., Arbeiten Kaiserl. Biol. Anst. Land- Forstw. 8: 155. 1910.

(See ***Fusarium oxysporum***)

*Syntypes*: B 70 0100192 & B 70 0100193.

*Type locality*: **Germany**, Berlin, Dahlem.

*Type substrate*: *Solanum tuberosum.*

*Note*: Typification pending further study of the syntypes in B.  

*orthoconium Fusarium* Wollenw., Fusaria Autogr. Delin. 2: 637. 1926.

***Mycogloea orthospora*** (Syd.) McNabb ex Dingley, Mem. New York Bot. Gard. 49: 206. 1989.

*Basionym*: *Microcera orthospora* Syd., Ann. Mycol. 22: 317. 1924, *non Fusarium orthosporum* Sacc. 1902.

*Synonyms*: *Fusarium microcera* var. *orthoconium* (Wollenw.) Bilaĭ, Mikrobiol. Zhurn. 49: 7. 1987, *nom. inval*., Arts. 35.1, 41.4.

*Holotypus*: Not located.

*Type locality*: **New Zealand**, Wellington, York Bay.

*Type substrate*: *Nothofagus* sp.  

*orthosporum Fusarium* Sacc. & P. Syd., Syll. Fung. 16: 1100. 1902.

***Cylindrodendrum orthosporum*** (Sacc. & P. Syd.) L. Lombard, ***comb. nov*.** MycoBank MB 837700.

*Basionym*: *Fusarium orthosporum* Sacc. & P. Syd., Syll. Fung. 16: 1100. 1902.

*Synonyms*: *Cylindrocarpon orthosporum* (Sacc. & P. Syd.) Wollenw., Fusaria Autogr. Delin. 1: 462. 1916.

*Ramularia orthospora* (Sacc. & P. Syd.) Wollenw., Fusaria Autogr. Delin. 1: 462. 1916.

*Neonectria hubeiensis* W.Y. Zhuang *et al.*, Fungal Diversity 24: 351. 2007.

*Ilyonectria hubeiensis* (W.Y. Zhuang *et al.*) Z.Q. Zeng & W.Y. Zhuang, Phytotaxa 85: 17. 2013.

*Cylindrodendrum hubeiense* (W.Y. Zhuang *et al.*) L. Lombard & Crous, Phytopathol. Medit. 53: 523. 2014.

*Holotypus*: In PAD.

*Type locality*: **France**.

*Type substrate*: *Juglans nigra*.

*Descriptions and illustrations*: See Zhuang *et al.* (2007) and Lombard *et al.* (2014).

*Notes*: The epithet of *Fusarium orthosporum* Sacc. & P. Syd (1902) predates that of *Neonectria hubeiensis* W.Y. Zhuang *et al.* (2007). Therefore, a new combination is proposed here with the older epithet.  

*oryzae Fusarium* Vincens, Rev. Pathol. Veg. Entomol. Agric. France 10: 126. 1923.

*Holotypus*: ?PC.

*Type locality*: **Vietnam**.

*Type substrate*: *Oryza sativa*.

*Notes*: Status unclear. Could be a synonym of *F. fujikuroi*.  

*osiliense Fusarium* Bres. & Vestergr., Bot. Not. 1900: 33. 1900.

***Septogloeum oxysporum*** Sacc. *et al.*, Bull. Soc. Roy. Bot. Belgique 29: 294. 1890.

*Syntypes*: In BPI, NEB, S & UPS.

*Type locality*: **Estonia**, Osilia.

*Type substrate*: *Briza media.*

*Notes*: Synonym *fide*Wollenweber & Reinking (1935). Typification pending further study of the syntypes.  

*ossicola Fusarium* (Berk. & M.A. Curtis) Sacc., Syll. Fung. 4: 714. 1886.

*Basionym*: *Fusisporium ossicola* Berk. & M.A. Curtis, Grevillea 3: 147. 1875.

(See ***Fusarium equiseti***)

*Holotypus*: ?K(M).

*Type locality*: **USA**.

*Type substrate*: Old decaying bones.

*Note*: Synonyms *fide*Wollenweber & Reinking (1935).  

*osteophilum Fusarium* Speg., Anales Soc. Ci. Argent. 10: 60. 1880.

(See ***Fusarium scirpi***)

*Holotypus*: In LPS (Fungi Argent. pug. 2, no. 155) *fide*Farr (1973).

*Type locality*: **Argentina**, Rio de la Plata, La Recoleta.

*Type substrate*: Decayed bones of *Gallus* sp. (chicken).

*Note*: Synonym *fide*Wollenweber & Reinking (1935).  

*otomycosis Fusarium* Y.N. Ming & T.F. Yu, Acta Microbiol. Sin. 12: 178. 1966.

*Holotypus*: Not located.

*Type locality*: **China**, Beijing.

*Type substrate*: Ear of *Homo sapiens*.

*Notes*: Status unclear. Requires further investigation.  

*oxydendri Fusarium* Ellis & Everh., Bull. Torrey Bot. Club 24: 477. 1897.

(See *Fusarium cavispermum*)

*Syntypes*: In BPI, BRU, CLEM, CUP, F, FLAS, ILL, ILLS, ISC, MICH, MSC, MU, NEB, OSC, PH, PUL, UC & WIS.

*Type locality*: **USA**, West Virginia.

*Type substrate*: *Oxydendrum arboreum.*

*Notes*: Synonym *fide*Wollenweber & Reinking (1935). Typification pending further study of the syntypes.  

***oxysporum Fusarium*** Schltdl., Fl. Berol. 2: 139. 1824.

*Synonyms*: *Fusisporium aurantiacum* Link, Mag. Ges. Naturf. Freunde Berlin 3: 19. 1809.

*Fusarium aurantiacum* (Link) Sacc., Syll. Fung. 4: 720. 1886, *nom. illegit.*, Art. 53.1.

*Fusarium aurantiacum* Corda, in Sturm, Deutschl. Fl., 3 Abt. (Pilze Deutschl.) 2: 19. 1829.

*Fusarium oxysporum* var. *aurantiacum* (Corda) Rabenh., Deutschl. Krypt.-Fl., 1: 51. 1844.

*Atractium aurantiacum* (Corda) Bonord., Abh. Naturf. Ges. Halle 8: 135. 1851.

*Fusisporium lagenariae* Schwein., Trans. Amer. Philos. Soc., n.s. 4: 275. 1834.

*Fusarium lagenariae* (Schwein.) Sacc., Syll. Fung. 4: 724. 1886.

*Hymenula equiseti* Lib., Pl. Crypt. Arduenna Fasc. 3: no. 236. 1834.

*Fusarium equisetorum* (Lib.) Desm., Pl. Crypt. N. France no. 1546/1846? 1843.

*Fusarium parasiticum* Thüm., Nuovo Giorn. Bot. Ital. 12: 198. 1880, *nom. illegit.*, Art. 53.1.

*Fusarium thuemenii* Sacc., Syll. Fung. 4: 722. 1886.

*Fusisporium calcareum* Thüm., Inst. Coimbra 28: 262. 1881.

*Fusarium calcareum* (Thüm.) Sacc., Syll. Fung. 4: 712. 1886.

*Fusarium eucalyptorum* Cooke & Harkn., Grevillea 9: 128. 1881.

*Fusarium oxysporum f*. *eucalypti* (Cooke & Harkn.) Arya & G.L. Jain, Phytopathology 52: 641. 1962.

*Fusarium oxysporum f*. *lycopersici* Sacc., Syll. Fung. 4: 705. 1886.

*Fusarium lycopersici* (Sacc.) Mussat, Syll. Fung. 15: 144. 1901, *nom. inval*., Art. 36.1(a), (c).

*Fusarium lycopersici* (Sacc.) Wollenw., Phytopathology 3: 29. 1913, *nom. illegit.*, Art. 53.1.

*Fusarium bulbigenum* Cooke & Massee, Grevillea 16: 49. 1887.

*Fusarium myosotidis* Cooke, Grevillea 16: 49. 1887.

*Leptosporium mycophilum* P. Karst., Meddel. Soc. Fauna Fl. Fenn. 16: 24. 1888.

*Fusarium mycophilum* (P. Karst.) Sacc., Syll. Fung. 10: 730. 1892.

?*Selenosporium cuticola* R. Blanch., Compt. Rend. Hebd. Séances Acad. Sci., Ser. D. 111: 479. 1890.

?*Fusarium cuticola* (R. Blanch.) Guég., Champ. Paras. Homme: 262. 1904.

*Fusarium sclerodermatis* Peck, Rep. (Annual) Regents Univ. State New York New York State Mus. 43: 77. 1890, *nom. illegit.*, Art. 53.1.

*Fusarium peckii* Sacc., Syll. Fung. 10: 727. 1892, *nom. illegit.*, Art. 53.1 [*pro. p. fide* Wollenweber & Reinking (1935)].

*Fusarium saccardoanum* P. Syd., Syll. Fung. 14: 1128. 1899.

*Fusarium vasinfectum* G.F. Atk., Bull. Alabama Agric. Exp. Sta. 41: 28. 1892.

*Fusarium cordae* Massee, Brit. Fung.-Fl. 3: 481. 1893.

*Fusarium niveum* E.F. Sm., Proc. Amer. Assoc. Advancem. Sci. 43: 289. 1894, *nom. inval*., Art. 36.1(a).

*Fusarium bulbigenum* var. *niveum* E.F. Sm. ex Wollenw., *Fusarien*: 117. 1931.

*Fusarium blasticola* Rostr. (as ‘*blasticolum*’), Gartn.-Tidende 1895: 122. 1895.

*Fusoma blasticola* (Rostr.) Sacc. & Traverso, Syll. Fung. 20: 1241. 1911.

*Fusarium bulbigenum* var*. blasticola* (Rostr.) Wollenw., Z. Parasitenk. (Berlin) 3: 412. 1931.

*Fusarium beticola* A.B. Frank, Kampfbuch gegen die Schädlinge unserer Feldfrüchte: 137. 1897.

*Fusarium dianthi* Prill. & Delacr., Compt. Rend. Hebd. Séances Acad. Sci. 129: 745. 1899.

*Fusarium oxysporum f*. *dianthi* (Prill. & Delacr.) W.C. Snyder & H.N. Hansen, Amer. J. Bot. 27: 66. 1940.

*Fusarium oxysporum* var. *dianthi* (Prill. & Delacr.) Raillo, Fungi of the Genus Fusarium: 255. 1950.

*Fusarium opuntiarum* Speg., Anales Mus. Nac. Hist. Nat. Buenos Aires 6: 350. 1898 [1899].

*Fusoma pini* Hartig, Lehrb. Pflanzenkrankh., Bot., Forstl., Landw. Gärt.: 116. 1900.

*Fusarium laxum* Peck, Bull. New York State Mus. Nat. Hist. 67: 30. 1903.

*Fusarium lini* Bolley, Proc. Annual Meeting Soc. Promot. Agric. Sci. 22: 42. 1902.

*Fusarium oxysporum f*. *lini* (Bolley) W.C. Snyder & H.N. Hansen, Amer. J. Bot. 27: 66. 1940.

*Fusarium tabacivorum* Delacr., Ann. Inst. Natl. Rech. Agron., ser. 2, 5: 207. 1906.

*Fusarium candidulum* Sacc., Ann. Mycol. 6: 567. 1908.

*Fusarium cubense* E.F. Sm., Science, N.Y. 31: 754. 1910.

*Fusarium oxysporum* var. *cubense* (E.F. Sm.) Wollenw., *Fusarien*: 119. 1935.

*Fusarium oxysporum f*. *cubense* (E.F. Sm.) W.C. Snyder & H.N. Hansen, Amer. J. Bot. 27: 66. 1940.

*Fusarium orthoceras* Appel & Wollenw., Arbeiten Kaiserl. Biol. Anst. Land- Forstw. 8: 155. 1910.

*Fusarium oxysporum* var. *orthoceras* (Appel & Wollenw.) Bilaĭ, Microbiol. Zhurn. 49: 7. 1987.

?*Fusarium violae* F.A. Wolf, Mycologia 2: 21. 1910.

*Fusarium albidoviolaceum* Dasz. (as ‘*albido-violaceum*’), Bull. Soc. Bot. Genève, sér. 2, 4: 293. 1912.

*Fusarium orthoceras* var. *albidoviolaceum* (Dasz.) Wollenw., Fusaria Autogr. Delin. 1: 361. 1916.

*Fusarium lycopersici* Bruschi, Atti Reale Accad. Lincei, Rendiconti Cl. Sci. Fis., ser. 5, 21: 298. 1912.

*Fusarium bulbigenum* var. *lycopersici* (Bruschi) Wollenw. & Reinking, *Fusarien*: 114. 1935.

*Fusarium citrinum* Wollenw., in Lewis, Bull. Maine Agric. Exp. Sta. 219: 256. 1913.

*Fusarium conglutinans* var. *citrinum* (Wollenw.) Wollenw., Z. Parasitenk. (Berlin) 3: 407. 1931.

*Fusarium conglutinans* Wollenw., Ber. Deutsch. Bot. Ges. 31: 34. 1913.

*Fusarium orthoceras* var. *conglutinans* (Wollenw.) Padwick, Indian J. Agric. Sci. 10: 282. 1940.

*Fusarium oxysporum f. conglutinans* (Wollenw.) W.C. Snyder & H.N. Hansen, Amer. J. Bot. 27: 66. 1940.

*Fusarium elegans* Appel & Wollenw., Arbeiten Kaiserl. Biol. Anst. Land- Forstw. 8: 94. 1913, *nom*. *inval*., Art. 36.1(a) (*non Fusarium elegans* W. Yamam. & Maeda 1962).

*Fusarium batatas* Wollenw. (as ‘*batatae*’), J. Agric. Res. 2: 268. 1914.

*Fusarium bulbigenum* var. *batatas* (Wollenw.) Wollenw., Z. Parasitenk. (Berlin) 3: 414. 1931.

*Fusarium oxysporum f*. *batatas* (Wollenw.) W.C. Snyder & H.N. Hansen, Amer. J. Bot. 27: 66. 1940.

*Fusarium cepae* Hanzawa, Mycol. Centralbl. 5(1): 5. 1914.

*Fusarium oxysporum f*. *cepae* (Hanzawa) W.C. Snyder & H.N. Hansen, Amer. J. Bot. 27: 66. 1940.

*Fusarium oxysporum* var. *cepae* (Hanzawa) Raillo, Fungi of the Genus Fusarium: 253. 1950.

*Fusarium hyperoxysporum* Wollenw., J. Agric. Res. 2: 268. 1914.

*Fusarium angustum* Sherb., Mem. Cornell Univ. Agric. Exp. Sta. 6: 203. 1915.

*Fusarium lutulatum* Sherb., Mem. Cornell Univ. Agric. Exp. Sta. 6: 209. 1915.

*Fusarium vasinfectum* var. *lutulatum* (Sherb.) Wollenw., Fusaria Autogr. Delin. 3: 1019. 1930.

*Fusarium lutulatum* var. *zonatum* Sherb., Mem. Cornell Univ. Agric. Exp. Sta. 6: 214. 1915.

*Fusarium zonatum* (Sherb.) Wollenw., Fusaria Autogr. Delin. 1: 392. 1916.

*Fusarium vasinfectum* var. *zonatum* (Sherb.) Wollenw., Fusaria Autogr. Delin. 3: 1020. 1930.

*Fusarium oxysporum* var. *asclerotium* Sherb., Mem. Cornell Univ. Agric. Exp. Sta. 6: 222. 1915.

*Fusarium asclerotium* (Sherb.) Wollenw., Fusaria Autogr. Delin. 1: 364. 1916.

*Fusarium sclerotioides* Sherb., Mem. Cornell Univ. Agric. Exp. Sta. 6: 214. 1915.

*Fusarium sclerotioides* var. *brevius* Sherb., Mem. Cornell Univ. Agric. Exp. Sta. 6: 218. 1915.

*Fusarium trifolii* Jacz., Jahrb. Pflanzenkrankh. Russl. VII-VIII: Abt. 6. 1917.

*Fusarium citrulli* Taubenh., Bull. Texas Agric. Exp. Sta. 260: 27. 1920.

*Fusarium malvacearum* Taubenh., Bull. Texas Agric. Exp. Sta. 260: 27. 1920.

*Fusarium poolense* Taubenh., Bull. Texas Agric. Exp. Sta. 260: 27. 1920.

*Fusarium macroxysporum* Lindf., Meddel. Centralanst. Försöksväs. Jordbruksomr. Avd. Lantbruksbot. 25: 8. 1922.

*Fusarium spinaciae* Hungerf., Phytopathology 13: 209. 1923.

*Fusarium cromyophthoron* Sideris, Phytopathology 14: 212. 1924.

*Fusarium loncheceras* Sideris, Phytopathology 14: 213. 1924.

*Fusarium loncheceras* var. *microsporon* Sideris, Phytopathology 14: 213. 1924.

*Fusarium rhizochromatistes* Sideris, Phytopathology 14: 212. 1924.

*Fusarium sclerostromaton* Sideris, Phytopathology 14: 213. 1924.

*Fusarium zonatum f*. 1 Link & Bailey, J. Agric. Res. 33: 941. 1926.

*Fusarium zonatum f.* 2 Link & Bailey, J. Agric. Res. 33: 941. 1926.

*Fusarium conglutinans* var. *betae* D. Stewart, Phytopathology 21: 67. 1931.

*Fusarium oxysporum f*. *betae* (D. Stewart) W.C. Snyder & H.N. Hansen, Amer. J. Bot. 27: 66. 1940.

*Fusarium oxysporum f*. 7 Wollenw., Fusaria Autogr. Delin. 4: 1176. 1935

*Fusarium apii* P.E. Nelson & Sherb., Techn. Bull. Michigan Agric. Exp. Sta 155: 42. 1937.

*Fusarium orthoceras* var. *apii* (R. Nelson & Sherb.) Wollenw. & Reinking, *Fusarien*: 112. 1935.

*Fusarium oxysporum f. apii* (R. Nelson & Sherb.) W.C. Snyder & H.N. Hansen, Amer. J. Bot. 27: 66. 1940.

*Fusarium apii* var. *pallidum* R. Nelson & Sherb., Techn. Bull. Michigan Agric. Exp. Sta. 155: 42. 1937.

*Fusarium bulbigenum* var. *apii* (R. Nelson & Sherb.) Raillo, Fungi of the Genus Fusarium: 251. 1950.

*Cylindrophora albedinis* Kill. & Maire, Bull. Soc. Hist. Nat. Afrique N. 21: 97. 1930, *nom. inval*., Art. 36.1(b).

*Fusarium oxysporum* var. *albedinis* Kill. & Maire ex Malençon, Rev. Mycol. (Paris) 15: 45– 60. 1950, *nom. inval*., Art. 36.1(b).

*Fusarium oxysporum f. sp. albedinis* Kill. & Maire ex W.L. Gordon, Canad. J. Bot. 43: 1310. 1965.

*Fusarium perniciosum* Hepting, Circ. U.S.D.A. 535: 7. 1939.

*Fusarium oxysporum f. perniciosum* (Hepting) Toole, Phytopathology 31: 599. 1941.

*Fusarium vasinfectum* var. *perniciosum* (Hepting) Carrera, Rev. Fac. Agron. Buenos Aires 13(3): 483 1955

?*Fusarium retusum* Wellman, Phytopathology 33: 957. 1943.

*Holotypus*: HAL 1612 F.

*Epitypus*: CBS H-23620, designated in Lombard *et al.* (2019b).

*Ex-epitype culture*: CBS 144134.

*Type locality*: **Germany**, Berlin.

*Type substrate*: *Solanum tuberosum.*

*Descriptions and illustrations*: See Lombard *et al.* (2019b)

*Diagnostic DNA barcodes*: *rpb2*: MH484953; *tef1*: MH485044.  

*palczewskii Fusarium* Jacz., Bull. Soc. Mycol. France 28: 345. 1912.

(See ***Fusarium avenaceum***)

*Lectotypus* (*hic designatus*, MBT 10000717): **Russia**, Ussuriysk, Primorsky krai (Far East Territory), grain of *Lolium* sp., 1912, A.A. Jaczewski, in Bull. Soc. Mycol. France 28: 345, fig. 1.

*Notes*: Synonyms *fide*Wollenweber & Reinking (1935). As no holotype specimen could be located; an illustration accompanying the original protologue is designated here as lectotype.  

*pallens Fusarium* Berk. & M.A. Curtis, Grevillea 3: 99. 1875, *nom. illegit.*, Art. 53.1.

*Replacing synonym*: *Fusarium glumarum* Sacc., Syll. Fung. 4: 706. 1886.

(See ***Fusarium incarnatum***)

*Authentic material*: Car. Inf. no. 3799, in K(M).

*Original locality*: **USA**.

*Original substrate*: *Juncus* sp.

*Note*: Synonyms *fide*Wollenweber & Reinking (1935).  

*pallens Fusarium* (Nees & T. Nees) Link, Sp. pl. 6(2): 104. 1825.

*Basionym*: *Atractium pallens* Nees & T. Nees, Nova Acta Phys.-Med. Acad. Caes. Leop.-Carol. Nat. Cur. 9: 237. 1818.

*Synonyms*: *Volutella pallens* (Nees & T. Nees) Fr., Syst. Mycol. 3: 468. 1832.

*Selenosporium pallens* (Nees & T. Nees) Corda, Icon. Fung. 1: 7. 1837.

*Holotypus*: In B.

*Type locality*: **Germany**.

*Type substrate*: Fallen branch.

*Notes*: The type material of *Atractium pallens* is deposited at B and examined by Gräfenhan *et al.* (2011), identifying it as a coelomycete.  

*pallidoroseum Fusarium* (Cooke) Sacc., Syll. Fung. 4: 720. 1886.

*Basionym*: *Fusisporium pallidoroseum* Cooke, Grevillea 6: 139. 1878.

(See ***Fusarium incarnatum***)

*Holotypus*: S. Car. no. 2279 in ?K(M).

*Type locality*: **USA**, South Carolina, Aiken.

*Type substrate*: *Chenopodium anthelminticum*.

*Note*: Synonyms *fide*Wollenweber & Reinking (1935).  

*pallidulum Fusarium* Sacc. & Trotter, Syll. Fung. 22: 1483. 1913.

*Replaced synonym*: *Atractium pallidum* Bonord., Handb. Mykol.: 135. 1851.

*Synonym*: *Fusarium pallidum* (Bonord.) Sacc. & Traverso, Syll. Fung. 19: 727. 1910, *nom. illegit.*, Art. 53.1.

*Lectotypus* (*hic designatus*, MBT 10000718): **Germany**, decaying bark, 1913, H.F. Bonorden, in Handb. Mykol., tab. 10, fig. 219.

*Notes*: Status unclear. Not *Fusarium fide*Wollenweber & Reinking (1935). As no holotype specimen could be located, an illustration accompanying the original protologue is designated here as lectotype.  

*pallidum Fusarium* Berk. & M.A. Curtis, J. Linn. Soc., Bot. 10: 359. 1869.

*Holotypus*: In K(M).

*Type locality*: **Cuba**.

*Type substrate*: Dead twigs.

*Notes*: Status unclear. Not *Fusarium fide*Wollenweber & Reinking (1935).  

***palustre Fusarium*** W.H. Elmer & Marra, ***sp. nov*.** MycoBank MB 837702.

*Synonym*: *Fusarium palustre* W.H. Elmer & Marra, Mycologia 103(4): 815. 2011, *nom. inval.*, Art. 40.7.

*Etymology*. ‘*palustre’*, from Latin *palus*, referring to marsh habitat in which this fungus is found.

For diagnosis see Elmer & Marra, Mycologia 103(4): 815. 2011.

*Holotypus*: CBS 126795 (preserved as metabolically inactive culture).

*Ex-type culture*: CBS 126796 = NRRL 54056.

*Type locality*: **USA**, Connecticut, Madison, Hammonasset Beach State Park.

*Type substrate*: *Spartina alterniflora.*

*Descriptions and illustrations*: See Elmer & Marra (2011).

*Diagnostic DNA barcodes*: *rpb1*: KT597718; *rpb2*: KT597731; *tef1*: GQ856941.

*Notes*: Elmer & Marra (2011) failed to indicate the holotype for *F. palustre*, rendering the species name invalid (Art. 40.7). Here we validate the name.  

*pampini Fusarium* Thüm. & Pass., Pilze Weinst.: 50. 1878.

***Gloeosporium physalosporae*** Cavara, Rev. Mycol. (Toulouse) 10: 99. 1888.

*Holotypus*: Not located.

*Type locality*: **Italy**, Parma.

*Type substrate*: *Vitis vinifera*.

*Note*: Synonym *fide*Wollenweber & Reinking (1935).  

*pandani Fusarium* (Corda) Sacc., Syll. Fung. 4: 724. 1886.

*Basionym*: *Fusisporium pandani* Corda, Icon. Fung. 1: 11. 1837.

*Lectotypus* (*hic designatus*, MBT 10000719): **Czech Republic**, Liberec (Reichenberg), *Pandanus* sp., 1837, A.C.J. Corda, in Icon. Fung. 1, tab. 2, fig. 162.

*Notes*: Status unclear. Not *Fusarium fide*Wollenweber & Reinking (1935). As no holotype specimen could be located, an illustration accompanying the original protologue is designated here as lectotype.  

*pannosum Fusarium* Massee, Bull. Misc. Inform. Kew 1898: 117. 1898.

(See ***Fusarium sambucinum***)

*Holotypus*: K(M) 191093.

*Type locality*: **India**, Punjab.

*Type substrate*: *Cornus macrophylla*.

*Note*: Synonym *fide*Wollenweber & Reinking (1935).  

*paraeumartii Fusarium* (Sand.-Den. & Crous) O'Donnell *et al.*, Index Fungorum 440: 3. 2020.

***Neocosmospora paraeumartii*** Sand.-Den. & Crous, Persoonia 43: 149. 2019.

*Holotypus*: CBS H-23991.

*Ex-type culture*: BBA 62215 = CBS 487.76 = NRRL 13997.

*Type locality*: **Argentina**.

*Type substrate*: Decaying stem base of *Solanum tuberosum*.

*Descriptions and illustrations*: See Sandoval-Denis *et al.* (2019).

*Diagnostic DNA barcodes*: *rpb1*: MW834240; *rpb2*: LR583855; *tef1*: DQ247549.  

*paranaense Fusarium* S.S. Costa *et al.*, Fungal Biology 120: 55. 2015 [2016].

(See *Fusarium falciforme*)

*Holotypus*: CML 1830.

*Ex-type culture*: CBS 141593 = CML 1830.

*Type locality*: **Brazil**, Goiás State, Cristalina.

*Type substrate*: Diseased tissue of *Glycine max.*

*Descriptions and illustrations*: See Costa *et al.* (2016).

*Diagnostic DNA barcodes*: *rpb2*: KF680011; *tef1*: KF597797.

*Note*: Synonym *fide*Sandoval-Denis *et al.* (2019).  

*parasiticum Fusarium* Westend., Bull. Acad. Roy. Sci. Belgique, Cl. Sci., sér. 2, 11: 652. 1861.

(See *Fusarium ciliatum*)

*Holotypus*: BR5020140791441.

*Type locality*: **Belgium**, Louette-Saint-Pierre.

*Type substrate*: *Sphaeria gigaspora*.

*Note*: Synonym *fide*Wollenweber & Reinking (1935).  

*parasiticum Fusarium* Thüm., Nuovo Giorn. Bot. Ital. 12: 198. 1880, *nom. illegit.*, Art. 53.1.

*Replacing synonym*: *Fusarium thuemenii* Sacc., Syll. Fung. 4: 722. 1886.

(See ***Fusarium oxysporum***)

*Authentic material*: Not located.

*Original locality*: **Russia**, Orenburg.

*Original substrate*: *Betula pendula*.

*Note*: Synonyms *fide*Wollenweber & Reinking (1935).  

*parasiticum Fusarium* Ellis & Kellerm., J. Mycol. 3: 127. 1887, *nom. illegit.*, Art. 53.1.

*Replacing synonym*: *Fusarium pucciniophilum* Sacc. & P. Syd., Syll. Fung. 14: 1128. 1899.

(See ***Fusarium heterosporum***)

*Authentic material*: Kellerman & Swingle 1104 in NY.

*Original locality*: **USA**, Manhattan.

*Original substrate*: Parasitic on *Puccinia seymeriae* on *Swietenia macrophylla*.

*Note*: Synonyms *fide*Wollenweber & Reinking (1935).  

*parasiticum Fusarium* Fautrey, Rev. Mycol. (Toulouse) 11: 153. 1889, *nom. illegit.*, Art. 53.1.

*Replacing synonym*: *Fusarium fautreyi* Sacc., Syll. Fung. 10: 934. 1892.

(See ***Fusarium lateritium***)

*Authentic material*: BR5020140789424.

*Original locality*: **France**, Noidan.

*Original substrate*: *Vitis vinifera*.

*Note*: Synonyms *fide*Wollenweber & Reinking (1935).  

*parceramosum Fusarium* (Sand.-Den. & Crous) O'Donnell *et al.*, Index Fungorum 440: 3. 2020.

***Neocosmospora parceramosa*** Sand.-Den. & Crous, Persoonia 43: 151. 2019.

*Holotypus*: CBS H-23992.

*Ex-type culture*: CBS 115695 = CPC 1246.

*Type locality*: **South Africa**.

*Type substrate*: Soil.

*Descriptions and illustrations*: See Sandoval-Denis *et al.* (2019).

*Diagnostic DNA barcodes*: *rpb2*: JX435249; *tef1*: JX435149.  

***parvisorum Fusarium*** Herron *et al.*, Stud. Mycol. 80: 146. 2015.

*Holotypus*: PREM 60897.

*Ex-type culture*: CBS 137236 = CMW 25267.

*Type locality*: **Colombia**, Vivero, Peñas Negra, Valle del Cauca.

*Type substrate*: *Pinus patula.*

*Descriptions and illustrations*: See Herron *et al.* (2015).

*Notes*: Comparisons of recently generated sequences for the living ex-type (CBS 137236 = CMW 25267) of *F. parvisorum* indicate a strain transposition or contamination by another *Fusarium* species. Therefore, this species needs to be recollected from the type locality and substrate or sequences need to be generated from the holotype specimen to confirm that it is indeed distinct.  

*paspali Fusarium* Henn., Bot. Jahrb. Syst. 38: 129. 1905.

(See ***Fusarium avenaceum***)

*Syntype*: In B as Zenker, Georg August, no. 2152 *fide*Hein (1988).

*Type locality*: **Cameroon**, Bipindi.

*Type locality*: *Paspalum* sp.

*Notes*: Synonym *fide*Wollenweber & Reinking (1935). Typification pending further study of the syntype in B.  

*paspalicola Fusarium* Henn., in Warburg, Monsunia 1: 38. 1899 [1900].

(See ***Fusarium heterosporum***)

*Holotypus*: In B *fide* Wollenweber, Fusaria Autogr. Delin. 1: 299. (1916) & Hein (1988).

*Type locality*: **Philippines**, Mindanao, Davao.

*Type substrate*: *Paspalum* sp.

*Note*: Synonym *fide*Wollenweber & Reinking (1935).  

*patouillardii Fusarium* Sacc. (as ‘*patouillardi*’), Syll. Fung. 10: 729. 1892.

*Replaced synonym*: *Fusarium uredinicola* Pat. & Gaillard, Bull. Soc. Mycol. France 4: 127. 1888, *nom. illegit.*, Art. 53.1.

*Holotypus*: ?PC or FH.

*Type locality*: **Venezuela**, Caracas.

*Type substrate*: Parasitic on *Sphaerellopsis filum* on *Puccinia pallidissima*.

*Notes*: Status unclear. Not *Fusarium fide*Wollenweber & Reinking (1935).  

*peckii Fusarium* Sacc., Syll. Fung. 4: 713. 1886.

*Replaced synonym*: *Fusisporium parasiticum* Peck, Rep. (Annual) New York State Mus. Nat. Hist. 29: 53. 1878, *non Fusarium parasiticum* Westend. 1861.

*Holotypus*: NYSf2260.

*Type locality*: **USA**, New York, Albany.

*Type substrate*: *Sphaeria collinsii*.

*Notes*: Status unclear. Not treated by Wollenweber & Reinking (1935) or Booth (1971).  

*peckii Fusarium* Sacc., Syll. Fung. 10: 727. 1892, *nom. illegit.*, Art. 53.1.

*Replaced synonyms*: *Fusarium sclerodermatis* Peck, Rep. (Annual) Regents Univ. State New York New York State Mus. 43: 77. 1890, *nom. illegit.*, Art. 53.1, *non Fusarium sclerodermatis* Oudem. 1889.

*Fusarium saccardoanum* Syd., Syll. Fung. 13: 1130. 1898.

(See ***Fusarium oxysporum*** pr. p. & ***Fusarium avenaceum*** pr. p.)

*Authentic material*: NYSf2731.

*Original locality*: **USA**, New York, Suffolk.

*Original substrate*: *Scleroderma vulgaris*.

*Note*: Synonyms *fide*Wollenweber & Reinking (1935).  

*pelargonii Fusarium* P. Crouan & H. Crouan, Fl. Finistère: 14. 1867.

(See *Fusarium merismoides*)

*Holotypus*: ?PC.

*Type locality*: **France**, Finistère.

*Type substrate*: *Pelargonium* sp.

*Note*: Synonym *fide*Wollenweber & Reinking (1935).  

*peltigerae Fusarium* Westend., Herb. Crypt. Belg. Fasc. 9: no. 414. 1849.

(See *Fusarium ciliatum*)

*Syntypes*: In BR & PH (Herb. Crypt. Belg. 9: no. 414).

*Type locality*: **Belgium**.

*Type substrate*: *Peltigera rufescens.*

*Notes*: Synonym *fide*Wollenweber & Reinking (1935). Typification pending further study of the syntypes.  

*penicillatum Fusarium* (Harz) Sacc., Syll. Fung. 4: 710. 1886.

*Basionym*: *Menispora penicillata* Harz, Bull. Soc. Imp. Naturalistes Moscou 44: 127. 1871.

(See ***Fusarium avenaceum***)

*Lectotypus* (*hic designatus*, MBT 10000720): **Germany**, Berlin, decaying *Sclerotium clavus*, 1886, C. Harz, in Bull. Soc. Imp. Naturalistes Moscou 44, tab. 1, fig. 4.

*Notes*: Synonym *fide*Wollenweber & Reinking (1935). As no holotype specimen could be located, an illustration accompanying the original protologue is designated here as lectotype.  

*pentaclethrae Fusarium* Henn., Hedwigia 44: 71. 1905.

(See *Fusarium coccidicola*)

*Syntype*: In B (Ule no. 3011) *fide*Hein (1988).

*Type locality*: **Brazil**, Manaus, Rio Nigro.

*Type substrate*: Leaves of *Pentaclethra* sp.

*Notes*: Synonym *fide*Wollenweber & Reinking (1935). Typification pending further study of the syntype in B.  

*penzigii Fusarium* Schroers *et al.*, Mycologia 101: 61. 2009.

***Bisifusarium penzigii*** (Schroers *et al.*) L. Lombard & Crous, Stud. Mycol. 80: 225. 2015.

*Holotypus*: CBS H-20125.

*Ex-type culture*: CBS 317.34 = NRRL 22109.

*Type locality*: **UK**, Surrey.

*Type substrate*: Decayed wood of *Fagus sylvatica.*

*Descriptions and illustrations*: See Schroers *et al.* (2009).

*Diagnostic DNA barcodes*: *rpb1*: KM232211; *rpb2*: KM232362; *tef1*: EU926324.  

***pernambucanum Fusarium*** A.C.S. Santos *et al.*, Mycologia 111: 253. 2019.

*Holotypus*: URM 91193.

*Ex-type culture*: MUM 1862 = URM 7559.

*Type locality*: **Brazil**, Pernambuco, Paudalho.

*Type substrate*: *Aleurocanthus woglumi.*

*Descriptions and illustrations*: See Santos *et al.* (2019).

*Diagnostic DNA barcodes*: *rpb1*: MH668869; *rpb2*: LS398519; *tef1*: LS398489.  

*perniciosum Fusarium* Hepting, Circul. U.S.D.A. 535: 7. 1939.

(See ***Fusarium oxysporum***)

*Holotypus*: Not located.

*Type locality*: **USA**.

*Type substrate*: *Albizia julibrissin*.  

*persicae Fusarium* (Sacc.) G.F. Atk., J. Elisha Mitchell Sci. Soc. 8: 41. 1892.

*Basionym*: *Cercospora persicae* Sacc. (as ‘*persica*’), Hedwigia 15: 119. 1876.

***Mycosphaerella pruni*-*persicae*** Deighton, Trans. Brit. Mycol. Soc. 50: 328. 1967.

*Synonyms*: *Cercosporella persicae* (Sacc.) Sacc. (as ‘*persica*’), Michelia 2: 20. 1880.

*Clasterosporium persicae* (Sacc.) Tsuji, Ann. Phytopathol. Soc. Japan 1(2): 33. 1919.

*Miuraea persicae* (Sacc.) Hara, Byogaichu-Hoten (Manual of Pests and Diseases): 224. 1948.

*Mycosphaerella persicae* B.B. Higgins & F.A. Wolf (as ‘*persica*’), Phytopathology 27: 695. 1937.

*Syntype*: In HAL, ILL & NEB (Saccardo, Mycoth. Ven. no. 598).

*Type locality*: **Italy**.

*Type substrate*: *Prunus persica*.  

***persicinum Fusarium*** J.W. Xia *et al.*, Persoonia 43: 215 2019.

*Holotypus*: CBS H-24068.

*Ex-type culture*: CBS 479.83.

*Type locality*: **Unknown**.

*Type substrate*: Unknown*.*

*Descriptions and illustrations*: See Xia *et al.* (2019).

*Diagnostic DNA barcodes*: *rpb2*: MN170428; *tef1*: MN170495.  

*personatum Fusarium* Cooke, in Harkness, Grevillea 7: 12. 1878.

(See *Fusarium allescherianum*)

*Holotypus*: ?K(M).

*Type locality*: **USA**, California.

*Type substrate*: *Oreodaphne californica*.

*Note*: Synonym *fide*Wollenweber & Reinking (1935).  

*perseae Fusarium* (Sand.-Den. & Guarnaccia) O'Donnell *et al.*, Index Fungorum 440: 3. 2020.

***Neocosmospora perseae*** Sand.-Den. & Guarnaccia, Fungal Syst. Evol. 1: 136. 2018.

*Holotypus*: CBS H-23433.

*Ex-type culture*: CBS 144142 = CPC 26829.

*Type locality*: **Italy**, Catania, San Leonardello.

*Type substrate*: Trunk canker lesions on *Persea americana*.

*Descriptions and illustrations*: See Guarnaccia *et al.* (2018).

*Diagnostic DNA barcodes*: *rpb1*: MW218130; *rpb2*: LT991909; *tef1*: LT991902.  

***peruvianum Fusarium*** L. Lombard & Crous, Fungal Syst. Evol. 4: 194. 2019.

*Holotypus*: CBS H-24019.

*Ex-type culture*: CBS 511.75.

*Type locality*: **Peru**.

*Type substrate*: Seedlings of *Gossypium* sp.

*Descriptions and illustrations*: See Lombard *et al.* (2019a).

*Diagnostic DNA barcodes*: *rpb1*: MN120728; *rpb2*: MN120746; *tef1*: MN120767.  

*pestis Fusarium* Sorauer, Atlas Pfl.-Krankh. 4: 19, pl. XXV. 1890.

(See *Fusarium azukiicola*)

*Holotypus*: Not located.

*Type locality*: **Germany**.

*Type substrate*: *Solanum tuberosum*.

*Note*: Synonym *fide*Wollenweber & Reinking (1935).  

***petersiae Fusarium*** L. Lombard, Persoonia 39: 457. 2017.

*Holotypus*: CBS H-23233.

*Ex-type culture*: CBS 143231.

*Type locality*: **Netherlands**, Gelderland Province, Arnhem.

*Type substrate*: Soil*.*

*Descriptions and illustrations*: See Crous *et al.* (2017).

*Diagnostic DNA barcodes*: *rpb1*: MG386139; *rpb2*: MG386150; *tef1*: MG386160.  

*petroliphilum Fusarium* (Q.T. Chen & X.H. Fu) Geiser *et al.*, Fungal Genet. Biol. 53: 69. 2013.

***Neocosmospora petroliphila*** (Q.T. Chen & X.H. Fu) Sand.-Den. & Crous, Persoonia 41: 121. 2018.

*Basionym*: *Fusarium solani* var. *petroliphilum* Q.T. Chen & X.H. Fu, Acta Mycol. Sin., Suppl. 1: 330. 1987.

*Synonyms*: *Fusarium solani f. sp*. *cucurbitae* (Race 2) W.C. Snyder & H.N. Hansen, Amer. J. Bot. 28: 740. 1941.

*Holotypus*: HMAS 43748.

*Ex-type culture*: FRC S-2176 = NF4475 = NRRL 22268.

*Type locality*: **China**, Beijing.

*Type substrate*: Deteriorated petroleum*.*

*Descriptions and illustrations*: See Sandoval-Denis & Crous (2018).  

*peziziforme Fusarium* Berk. & M.A. Curtis (as ‘*pezizaeforme*’), J. Linn. Soc., Bot. 10: 360. 1869.

*Holotypus*: In K(M).

*Type locality*: **Cuba**.

*Type substrate*: *Poaceae*.

*Note*: Not *Fusarium fide*Wollenweber & Reinking (1935).  

*pezizoides Fusarium* Desm., Ann. Sci. Nat., Bot., sér. 3, 18: 373. 1852.

***Trochila craterium*** (DC.) Fr., Summa Veg. Scand. 2: 367. 1849.

*Basionym*: *Sphaeria craterium* DC., Fl. Franç., ed. 3, 2: 298. 1805.

*Synonyms*: *Phacidium craterium* (DC.) Gillet, Champ. France Discomyc. (7): 167. 1886.

*Sphaeria punctiformis* var. *hederae* Pers., Syn. Meth. Fung. 1: 90. 1801.

*Myxosporium paradoxum* De Not., Mem. Reale Accad. Sci. Torino, ser. 2, 3: 81. 1841.

*Gloeosporium paradoxum* (De Not.) Mont., in Berkeley & Broome, Ann. Mag. Nat. Hist. 5: 455. 1850.

*Gloeosporidium paradoxum* (De Not.) Petr., Ann. Mycol. 20: 14. 1922.

*Cryptocline paradoxa* (De Not.) Arx, Verh. Kon. Ned. Akad. Wetensch., Afd. Natuurk. 51: 115. 1957.

*Gloeotrochila paradoxa* (De Not.) Petr., Sydowia 1: 50. 1947.

*Trochila craterium* var. *nucleata* Rehm, Ber. Bayer. Bot. Ges. 13: 125. 1912.

*Ceuthospora hederae* Grove, Bull. Misc. Inform. Kew 1923: 355. 1923.

*Holotypus*: ?PC.

*Type locality*: **France**.

*Type substrate*: *Peziza insidiosa*.

*Note*: Synonyms *fide*Wollenweber & Reinking (1935).  

*pezizoideum Fusarium* (Berk. & M.A. Curtis) Sacc., Syll. Fung. 4: 711. 1886.

*Basionym*: *Fusisporium pezizoideum* Berk. & M.A. Curtis, Grevillea 3: 147. 1875.

(See ***Fusarium sambucinum***)

*Holotypus*: ?K(M).

*Type locality*: **USA**, Pennsylvania.

*Type substrate*: Stems of herbaceous plants.

*Note*: Synonyms *fide*Wollenweber & Reinking (1935).  

*phacidioideum Fusarium* Dearn., Mycologia 21: 331. 1929.

*Holotypus*: JD 4303 in DAOM.

*Type locality*: **Canada**, Vancouver, Stanley Park.

*Type substrate*: Dead branches of *Pseudotsuga taxifolia*.

*Note*: Status unclear; requires recollection from type locality and substrate.  

***pharetrum Fusarium*** L. Lombard & Crous, Persoonia 43: 32. 2018 [2019].

*Holotypus*: CBS H-23621.

*Ex-type culture*: CBS 144751 = CPC 30824.

*Type locality*: **South Africa**.

*Type substrate*: *Aloidendron dichotomum.*

*Descriptions and illustrations*: See Lombard *et al.* (2019b).

*Diagnostic DNA barcodes*: *rpb1*: MW928815; *rpb2*: MH484952; *tef1*: MH485043.  

*phaseoli Fusarium* (Burkh.) T. Aoki & O'Donnell, Mycologia 95: 671. 2003.

*Basionym*: *Fusarium martii f. phaseoli* Burkh., Mem. Cornell Univ. Agric. Exp. Sta. 26: 1007. 1919.

(See *Fusarium azukiicola*)

*Lectotypus* (*hic designatus*, MBT 10000721): **USA**, New York, roots of *Phaseolus vulgaris*, 1919, W.H. Burkholder, in Mem. Cornell Univ. Agric. Exp. Sta. 26: 1009, fig. 134.

*Notes*: Synonym *fide*Sandoval-Denis *et al.* (2019). Although Burkholder deposited several specimens in CUP, none are directly linked to the original protologue (Burkholder 1919). Several of these specimens appear to have been isolated from greenhouse assays undertaken by Burkholder. Therefore, an illustration accompanying the original protologue is designated here as lectotype.  

***phialophorum Fusarium*** Maryani *et al.*, Stud. Mycol. 92: 169. 2018 [2019].

*Holotypus*: InaCC F971 (preserved as metabolically inactive culture).

*Ex-type culture*: InaCC F971.

*Type locality*: **Indonesia**, South Kalimantan, Tanah Bumbu, Kampung Betung.

*Type substrate*: *Musa* var. Pisang Awak*.*

*Descriptions and illustrations*: See Maryani *et al.* (2019a).

*Diagnostic DNA barcodes*: *rpb1*: LS479545; *rpb2*: LS479292; *tef1*: LS479741.  

*phormii Fusarium* Henn., Verh. Bot. Vereins Prov. Brandenburg 40: 175. 1898 [1899].

***Colletotrichum phormii*** (Henn.) D.F. Farr & Rossman, Mycol. Res. 110: 1403. 2006.

*Synonym*: *Gloeosporium phormii* (Henn.) Wollenw., Fusaria Autogr. Delin. No. 498. 1916, *nom. illegit.*, Art. 53.1, *non Gloeosporium phormii* Sacc. 1915.

*Holotypus*: B 70 0005220.

*Epitypus*: CBS H-20720, designated in Damm *et al.* (2012).

*Ex-epitype*: A.R. 3546 = CBS 118194.

*Type locality*: **Germany**, Berlin.

*Type substrate*: *Phormium tenax*.  

*phragmiticola Fusarium* Kirschst., Ann. Mycol. 34: 183. 1936, *nom. inval*., Art. 39.1.

*Authentic material*: B 70 0100199, B 70 0100200, B 70010020.

*Original locality*: **Germany**.

*Original substrate*: *Phragmites communis*.  

*phragmitis Fusarium* Matsush., Icon. Microfung. Matsush. Lect.: 72. 1975, *nom. inval*., Art. 40.1.

*Authentic material*: Not indicated.

*Original locality*: **Japan**.

*Original substrate*: Rotten wood of *Fagus crenata*.  

*phyllachorae Fusarium* Henn., in de Wildeman, Mission E. Laurent, Fasc. 4: 363. 1907.

*Syntype*: Laurent in B *fide*Hein (1988).

*Type locality*: **Democratic Republic of Congo**, between Kinshasa and Kwamouth.

*Type substrate*: *Panicum maximum.*

*Notes*: Not *Fusarium fide*Wollenweber & Reinking (1935). Typification pending further study of the syntype in B.  

*phyllogenum Fusarium* (Cooke & Peck) Sacc., Syll. Fung. 4: 703. 1886.

*Basionym*: *Fusisporium phyllogenum* Cooke & Peck, Rep. (Annual) New York State Mus. Nat. Hist. 29: 53. 1878.

*Syntype*: NYSf2335.

*Type locality*: **USA**, New York, Albany, Bethlehem.

*Type substrate*: *Erigeron annuum.*

*Notes*: Status unclear. Not *Fusarium fide*Wollenweber & Reinking (1935). Typification pending further study of the syntype in NYS.  

***phyllophilum Fusarium*** Nirenberg & O'Donnell, Mycologia 90: 447. 1998.

*Holotypus*: IMI 202874.

*Ex-type culture*: BBA 63625 = CBS 216.76 = DAOM 225132 = IMI 375338 = NRRL 13617.

*Type locality*: **Italy**.

*Type substrate*: *Dracaena deremensis.*

*Descriptions and illustrations*: See Nirenberg & O'Donnell (1998).

*Diagnostic DNA barcodes*: *rpb1*: KF466399; *rpb2*: KF466410; *tef1*: KF466421.  

***phyllostachydicola Fusarium*** W. Yamam., Trans. Mycol. Soc. Japan 3: 118. 1962.

*Basionym*: *Gibberella phyllostachydicola* W. Yamam., Hyogo Univ. Agric. ser. Agric. Biol. 3: 15. 1957.

*Lectotypus* (*hic designatus*, MBT 10000722): **Japan**, Tamba, Sasayama-cho, culms of *Phyllostachys bambusoides*, 31 Aug. 1956, W. Yamamoto, in Hyogo Univ. Agric. ser. Agric. Biol. 3: 17, figs 16–18.

*Descriptions and illustrations*: See Yamamoto *et al.* (1957).

*Notes*: This species requires recollection from the type host and locality. As no holotype specimen could be located, an illustration accompanying the original protologue is designated here as lectotype.  

***pilosicola Fusarium*** Yilmaz *et al.*, Persoonia 46: 152. 2021.

*Holotypus*: PREM 63216.

*Ex-type culture*: CMWF 1183 = NRRL 29124 = NY007.H7.

*Type locality*: **USA**, Florida.

*Type substrate*: *Bidens pilosa*.

*Descriptions and illustrations*: See Yilmaz *et al.* (2021).

*Diagnostic DNA barcodes*: *rpb2*: MN534248; *tef1*: MN534055.  

***pininemorale Fusarium*** Herron *et al.*, Stud. Mycol. 80: 146. 2015.

*Holotypus*: PREM 60901.

*Ex-type culture*: CBS 137240 = CMW 25243.

*Type locality*: **Colombia**, Risaralda, Angela Maria (Santa Rosa).

*Type substrate*: *Pinus tecunumanii.*

*Descriptions and illustrations*: See Herron *et al.* (2015).

*Notes*: Comparisons of recently generated sequences from the living ex-type (CBS 137240 = CMW 25243) of *F. pininemorale* indicate a strain transposition or contamination by another *Fusarium* species. Therefore, this species needs to be recollected from the type locality and substrate or sequences need to be generated from the holotype specimen to confirm its phylogenetic affiliation.  

*piperis Fusarium* (F.C. Albuq.) O'Donnell *et al.*, Index Fungorum 440: 3. 2020.

***Neocosmospora piperis*** (F.C. Albuq.) Sand.-Den. & Crous, Persoonia 43: 152. 2019.

*Basionym*: *Fusarium solani f. piperis* F.C. Albuq., Circ. Inst. Agron. N. 5: 19. 1961.

*Holotypus*: IAN 825 in the herbarium of Embrapa Amazoˆnia Oriental.

*Epitypus*: CBS H-23993, designated in Sandoval-Denis *et al.* (2019).

*Ex-epitype culture*: CBS 145470 = CML 1888 = G.J.S. 89-14 = NRRL 22570.

*Type locality*: **Brazil**.

*Type substrate*: *Piper nigrum*.

*Descriptions and illustrations*: See Sandoval-Denis *et al.* (2019).

*Diagnostic DNA barcodes*: *rpb1*: MW834241; *rpb2*: EU329513; *tef1*: AF178360.  

*pisi Fusarium* (F.R. Jones) A. Šišić *et al.*, Sci. Rep. 8(no. 1252): 2. 2018, *nom. inval*., Art. F.5.1.

***Neocosmospora pisi*** (F.R. Jones) Sand.-Den. & Crous, Persoonia 43: 154. 2019.

*Basionym*: *Fusarium martii* var. *pisi* F.R. Jones, J. Agric. Res. 26: 459. 1923.

*Synonyms*: *Fusarium solani f*. *pisi* (F.R. Jones) W.C. Snyder & H.N. Hansen, Amer. J. Bot. 28: 740. 1941.

*Fusarium vanettenii* O'Donnell *et al.*, Index Fungorum 440: 5. 2020.

*Fusarium solani* var. *martii* ‘f 2’ Wollenw., Z. Parasitenk. (Berlin) 3: 290. 1931.

*Hypomyces solani f. sp. pisi* Reichle, W.C. Snyder & Matuo, Nature 203: 664. 1964.

*Lectotypus*: Jones (1923; fig. 1 on p. 463), designated in Sandoval-Denis *et al.* (2019).

*Epitypus*: CBS H-23994, designated in Sandoval-Denis *et al.* (2019).

*Ex-epitype culture*: ATCC MYA-4622 = CBS 123669 = NRRL 45880 = Vanetten 77-13-4.

*Type locality*: **USA**.

*Type substrate*: Sexual cross of parents from *Pisum sativum* and soil from a potato field*.*

*Descriptions and illustrations*: See Šišić *et al.* (2018b) and Sandoval-Denis *et al.* (2019).

*Diagnostic DNA barcodes*: *rpb1*: JX171543; *rpb2*: EU329640; *tef1*: FJ240352.  

*plagianthi Fusarium* (Dingley) O'Donnell & Geiser, Phytopathology 103: 404. 2013.

***Neocosmospora plagianthi*** (Dingley) L. Lombard & Crous, Stud. Mycol. 80: 227. 2015.

*Basionym*: *Nectria plagianthi* Dingley, Trans. Roy. Soc. New Zealand 79: 196. 1951.

?*Nectria pulverulenta* Dingley, Trans. Roy. Soc. New Zealand 83: 657. 1956.

*Holotypus*: PDD 10916.

*Type locality*: **New Zealand**, Fiordland, Hollyford Valley.

*Type substrate*: *Plagianthus betulinus.*

*Descriptions and illustrations*: See Dingley (1951) and Samuels & Brayford (1994).  

*platani Fusarium* (Lév.) Mont., Ann. Sci. Nat., Bot., sér. 3, 11: 55. 1849.

*Basionym*: *Hymenula platani* Lév., Ann. Sci. Nat., Bot., sér. 3, 9: 128. 1848

(See *Fusarium nervisequum*)

*Holotypus*: ?PC.

*Type locality*: **France**.

*Type substrate*: *Platanus orientalis*.  

*platanoidis Fusarium* Oudem., Ned. Kruidk. Arch., sér. 3, 2: 1131. 1904.

*Holotypus*: ?L.

*Type locality*: **Netherlands**, Gelderland Province, Nunspeet.

*Type substrate*: *Acer platanoides*.

*Note*: Not *Fusarium fide*Wollenweber & Reinking (1935).  

***poae Fusarium*** (Peck) Wollenw., in Lewis, Bull. Maine. Agric. Exp. Sta. 219: 254. 1913 [1914].

*Basionym*: *Sporotrichum poae* Peck, Bull. New York State Mus. 67: 29. 1904 [1903].

*Synonyms*: *Fusarium tricinctum f*. *poae* (Peck) W.C. Snyder & H.N. Hansen, Amer. J. Bot. 32: 663. 1945.

*Fusarium sporotrichiella* var. *poae* (Peck) Bilaĭ, Yadovitye griby na zerne khlebnykh zlakov (*Poisonous fungi on cereal* see*d*): 86. 1953, *nom. inval.*, Art. 39.1.

*Fusarium sporotrichiella* var. *poae* (Peck) Bilaĭ, Microbiol. Zhurn. 49: 6. 1987, *nom. inval*., Arts. 35.1, 41.4.

*Sporotrichum anthophilum* Peck, Bull. New York State Mus. 105: 28. 1906.

*Fusarium maydiperdum* Bubák, Centralbl. Bakteriol. Parasitenk., 2. Abth. 31: 497. 1911.

*Holotypus*: NYSf2393.

*Type locality*: **USA**, New York, Geneva.

*Type substrate*: Sheaths and culms of *Poa pratensis*.

*Epitypus* (*hic designatus*, MBT 10000723): **USA**, North Dakota, Minot, from infected barley kernel, date and collector unknown, NRRL 26941 (preserved as metabolically inactive culture).

*Ex-epitype culture*: NRRL 26941.

*Descriptions and illustrations*: See Wollenweber & Reinking (1935), Booth (1971), Gerlach & Nirenberg (1982) and Leslie & Summerell (2006).

*Diagnostic DNA barcodes*: *rpb1*: KU171686; *rpb2*: KU171706; *tef1*: JABFFD010000730.1

*Note*: No living material linked to the holotype is available for this important mycotoxin producing species, and therefore, an epitype is designated here to provide taxonomic stability for this species.  

*poincianae Fusarium* Pass., Atti Reale Accad. Lincei, Rendiconti Cl. Sci. Fis., sér. 4, 4: 105. 1888.

*Holotypus*: Not located.

*Type locality*: **Italy**, Parma.

*Type substrate*: *Poinciana gilliesii*.

*Note*: Not *Fusarium fide*Wollenweber & Reinking (1935).

*polymorphum Fusarium* Matr., Rech. Dével. Mucéd.: 84. 1892.

(See ***Fusarium sambucinum***)

*Lectotypus* (*hic designatus*, MBT 10000724): **France**, horse dung, 1892, L. Matruchot, in Rech. Dével. Mucéd., Pl. 7, figs 6–14.

*Notes*: Synonym *fide*Wollenweber & Reinking (1935). As no holotype specimen could be located, an illustration accompanying the original protologue is designated here as lectotype.  

*polymorphum Fusarium* Marchal, Bull. Soc. Roy. Bot. Belgique 34: 145. 1895, *nom. illegit.*, Art. 53.1.

(See *Fusarium aderholdii*)

*Authentic material*: Not located.

*Original locality*: **Belgium**, Brussels.

*Original substrate*: *Homo sapiens*.

*Note*s: Synonym *fide*Wollenweber & Reinking (1935).  

*polyphialidicum Fusarium* Marasas *et al.*, Mycologia 78: 678. 1986.

(See ***Fusarium concolor***)

*Holotypus*: DAOM 192986.

*Ex-type culture*: ATCC 60096 = CBS 961.87 = DAR 52851 = FRC M-2405 = MRC 3389 = NRRL 13459.

*Type locality*: **South Africa**, Mpumalanga Province, Nelspruit.

*Type substrate*: Plant debris in soil*.*

*Descriptions and illustrations*: See Marasas *et al.* (1986).

*Diagnostic DNA barcodes*: *rpb1*: JX171455; *rpb2*: JX171569; *tef1*: MH742681.  

*poncetii Fusarium* Guiart (as ‘*ponceti*’), Compt.-Rend. Séances Mém. Soc. Biol. 73: 271. 1912, *nom. inval*., Art. 36.1(a).

*Authentic material*: Not located.

*Original locality*: **?France**.

*Original substrate*: *Homo sapiens* granuloma teleangiectaticum.

*Notes*: Status unclear. Not treated by any of Wollenweber & Reinking (1935), Booth (1971), or Gerlach & Nirenberg (1982).  

*poolense Fusarium* (as ‘*poolensis*’) Taubenh., Bull. Texas Agric. Exp. Sta. 260: 27. 1920.

(See ***Fusarium oxysporum***)

*Lectotypus* (*hic designatus*, MBT 10000725): **USA**, *Citrullus lanatus*, 1920, J.J. Taubenhaus, in Bull. Texas Agric. Exp. Sta. 260: 30, fig. 8i.

*Notes*: Synonym *fide*Wollenweber & Reinking (1935). As no holotype specimen could be located, an illustration accompanying the original protologue is designated here as lectotype.  

***praegraminearum Fusarium*** Gräfenhan & O'Donnell, Mycologia 108: 1232. 2016.

*Holotypus*: PDD 47563.

*Ex-type culture*: CBS 141369 = ICMP 8996 = NRRL 39664.

*Type locality*: **New Zealand**, North Island, Levin (near Wellington).

*Type substrate*: Litter in maize paddock*.*

*Descriptions and illustrations*: See Gräfenhan *et al.* (2016).

*Diagnostic DNA barcodes*: *rpb1*: KX260125; *rpb2*: KX260126; *tef1*: KX260120.  

***prieskaense Fusarium*** G.J. Marais & Sand.-Den., Stud. Mycol. 98 (no. 100116): 50. 2021.

*Holotypus*: CBS H-24660.

*Ex-type culture*: CAMS 001176 = CBS 146498 = CPC 30826.

*Type locality*: **South Africa**, Northern Cape Province, Prieska.

*Type substrate*: *Prunus spinosa*.

*Descriptions and illustrations*: See this study.

*Diagnostic DNA barcodes*: *rpb1:* MW834190; *rpb2:* MW834007; *tef1:* MW834275.  

***proliferatum Fusarium*** (Matsush.) Nirenberg ex Gerlach & Nirenberg, Mitt. Biol. Bundesanst. Land- Forstw. 209: 309. 1982.

*Basionym*: *Cephalosporium proliferatum* Matsush., Microfungi of the Solomon Islands and Papua-New Guinea: 11. 1971.

*Synonyms*: *Fusarium proliferatum* (Matsush.) Nirenberg, Mitt. Biol. Bundesanst. Land- Forstw. 169: 38. 1976, *nom. inval*., Art. 41.3.

*Fusarium proliferatum* var. *minus* Nirenberg, Mitt. Biol. Bundesanst. Land- Forstw. 169: 43. 1976. *nom. inval.*, Art. 41.3.

*Lectotypus*: Microfungi of the Solomon Islands and Papua-New Guinea: 11, fig 121.2, designated by Yilmaz *et al.* (2021).

*Lectotype locality*: **Papua New Guinea**.

*Lectotype substrate*: Forest soil.

*Epitypus*: CBS 480.96 (preserved as metabolically inactive culture), designated by Yilmaz *et al.* (2021).

*Epitype locality*: **Papua New Guinea**, Morobe Province, Bulolo.

*Epitype substrate*: Forest soil*.*

*Ex-epitype culture*: CBS 480.96 = IAM 14682 = NRRL 26427 = NY007.B6.

*Descriptions and illustrations*: See Matsushima (1971), Yilmaz *et al.* (2021).

*Diagnostic DNA barcodes*: *rpb2:* MN534272; *tef1:* MN534059.  

*protoensiforme Fusarium* (Sand.-Den. & Crous) O'Donnell *et al.*, Index Fungorum 440: 3. 2020.

***Neocosmospora protoensiformis*** Sand.-Den. & Crous, Persoonia 43: 156. 2019.

*Holotypus*: CBS H-23995.

*Ex-type culture*: CBS 145471 = G.J.S. 90-168 = NRRL 22178.

*Type locality*: **Venezuela**.

*Type substrate*: Bark of dicot tree.

*Descriptions and illustrations*: See Sandoval-Denis *et al.* (2019).

*Diagnostic DNA barcodes*: *rpb1*: MW834244; *rpb2*: EU329498; *tef1*: AF178334.  

*protractum Fusarium* Lév., Ann. Sci. Nat., Bot., sér. 3, 9: 246. 1848.

(See ***Fusarium lateritium***)

*Holotypus*: ?PC.

*Type locality*: **France**, Romainville.

*Type substrate*: Dead shoots of *Solanum dulcamara*.

*Note*: Synonym *fide*Wollenweber & Reinking (1935).  

*prunorum Fusarium* McAlpine, *Fungus Diseases of stone-fruit trees in Australia*: 91. 1902.

(See *Fusarium candidum* (Link) Sacc.)

*Lectotypus* (*hic designatus*, MBT 10000726): **Australia**, Victoria, Melbourne, Burnley, from shriveled and blackened apricot fruit, Jun. 1900, D. McAlpine, in Fungus Diseases of stone-fruit trees in Australia (1902), pl. XX, fig. 42.

*Notes*: Synonym *fide*Wollenweber & Reinking (1935). As no holotype specimen could be located, an illustration accompanying the original protologue is designated here as lectotype.  

*pseudacaciae Fusarium* Rapaics, Z. Pflanzenkrankh. 25: 208. 1915.

(See ***Fusarium lateritium***)

*Holotypus*: Not located.

*Type locality*: **Hungary**, Debrecen.

*Type substrate*: *Robinia pseudoacaciae*.

*Note*: Synonym *fide*Wollenweber & Reinking (1935).  

*pseudensiforme Fusarium* Samuels *et al.*, Mycologia 103: 1323. 2011.

***Neocosmospora pseudensiformis*** Samuels *et al.*, Mycologia 103: 1323. 2011.

*Holotypus*: BPI 881226.

*Ex-type culture*: CBS 125729 = FRC S-1834 = G.J.S 02-95 = G.J.S 9318 = NRRL 46517.

*Type locality*: **Sri Lanka**, Wagamba, Kurunegala, Arangakele.

*Type substrate*: Bark of tree*.*

*Descriptions and illustrations*: See Nalim *et al.* (2011).

*Diagnostic DNA barcodes*: *rpb1*: KC691615; *rpb2*: KC691645; *tef1*: KC691555.  

***pseudoanthophilum Fusarium*** Nirenberg *et al.*, Mycologia 90: 461. 1998.

*Holotypus*: In B.

*Ex-type culture*: BBA 69002 = CBS 414.97 = IMI 376112 = NRRL 25211.

*Type locality*: **Zimbabwe**, Gambiza.

*Type substrate*: *Zea mays.*

*Descriptions and illustrations*: See Nirenberg *et al.* (1998).

*Diagnostic DNA barcodes*: *rpb1*: MT010949; *rpb2*: MT010980; *tef1*: MK639073.  

***pseudocircinatum Fusarium*** O'Donnell & Nirenberg, Mycologia 90: 448. 1998.

*Holotypus*: B 70 0001689.

*Ex-type culture*: BBA 69636 = CBS 126.73= CBS 449.97 = DAOM 225117 = IMI 375316 = NRRL 22946.

*Type locality*: **Ghana**.

*Type substrate*: *Solanum* sp*.*

*Descriptions and illustrations*: See Nirenberg & O'Donnell (1998).

*Diagnostic DNA barcodes*: *rpb1*: MG838070; *rpb2*: MN724939; *tef1*: MG838023.  

*pseudoeffusum Fusarium* Murashk., Proc. Siberian Agric. Acad. Omsk 3: 106. 1924.

(See ***Fusarium acuminatum***)

*Holotypus*: Not located.

*Type locality*: **Russia**, Siberia.

*Type substrate*: *Triticum polonicum*.

*Note*: Synonym *fide*Wollenweber & Reinking (1935).  

***pseudograminearum Fusarium*** O'Donnell & T. Aoki, Mycologia 91: 604. 1999.

*Holotypus*: BPI 746087.

*Ex-type culture*: FRC R-5291 = NRRL 28062.

*Type locality*: **Australia**, New South Wales, Young.

*Type substrate*: *Hordeum vulgare.*

*Descriptions and illustrations*: See Aoki & O'Donnell (1999).

*Diagnostic DNA barcodes*: *rpb1*: JX171524; *rpb2*: JX171637; *tef1*: AF212468.  

*pseudoheterosporum Fusarium* Jacz., Bull. Soc. Mycol. France 28: 347. 1912.

(See ***Fusarium avenaceum***)

*Holotypus*: Not located.

*Type locality*: **France**.

*Type substrate*: *Lolium* sp. and *Triticum* sp.

*Note*: Synonym *fide*Wollenweber & Reinking (1935).  

*pseudonectria Fusarium* Speg., Anales Mus. Nac. Hist. Nat. Buenos Aires 6: 351. 1898 [1899].

(See ***Fusarium avenaceum***)

*Holotypus*: In LPS (Fungi Argent. n.v.c. no. 867) *fide*Farr (1973).

*Type locality*: **Ecuador**, San Salvador Island.

*Type substrate*: Dead culms of *Poaceae*.

*Note*: Synonym *fide*Wollenweber & Reinking (1935).  

***pseudonygamai Fusarium*** O'Donnell & Nirenberg, Mycologia 90: 449. 1998.

*Holotypus*: B 70 0001688.

*Ex-type culture*: BBA 69552 = CBS 417.97 = DAOM 225136 = FRC M-1166 = IMI 375342 = NRRL 13592.

*Type locality*: **Nigeria**.

*Type substrate*: *Pennisetum typhoides.*

*Descriptions and illustrations*: See Nirenberg & O'Donnell (1998).

*Diagnostic DNA barcodes*: *rpb1*: LT996205; *rpb2*: LT996152; *tef1*: AF160263.  

*pseudoradicicola Fusarium* (Sand.-Den. & Crous) O'Donnell *et al.*, Index Fungorum 440: 3. 2020.

***Neocosmospora pseudoradicicola*** Sand.-Den. & Crous, Persoonia 43: 157. 2019.

*Holotypus*: CBS H-23996.

*Ex-type culture*: ARSEF 2313 = CBS 145472 = NRRL 25137.

*Type locality*: **Papua New Guinea**, East New Britain, Keravat, Lowlands Agricultural Experiment Station.

*Type substrate*: Diseased pods of *Theobroma cacao*.

*Descriptions and illustrations*: See Sandoval-Denis *et al.* (2019).

*Diagnostic DNA barcodes*: *rpb1*: MW218133; *rpb2*: JF741084; *tef1*: JF740757.  

*pseudotonkinense Fusarium* (Sand.-Den. & Crous) O'Donnell *et al.*, Index Fungorum 440: 3. 2020.

***Neocosmospora pseudotonkinensis*** Sand.-Den. & Crous, Persoonia 43: 159. 2019.

*Holotypus*: CBS H-23997.

*Ex-type culture*: CBS 143038.

*Type locality*: **Netherlands**, Zuid-Holland Province, Leiden.

*Type substrate*: Cornea of *Homo sapiens*.

*Descriptions and illustrations*: See Sandoval-Denis *et al.* (2019).

*Diagnostic DNA barcodes*: *rpb2*: LR583867; *tef1*: LR583640.  

*pteridis Fusarium* Ellis & Everh., Proc. Acad. Nat. Sci. Philadelphia 45: 466. 1894.

***Septogloeum pteridis*** (Ellis & Everh.) Wollenw., Fusaria Autogr. Delin. 1: 446. 1916.

*Syntypes*: In BPI, BRU, CUP, FLAS, ILL, ISC, MICH, MSC, MU, NEB, NY, OSC, PH, PUL, WIS & WSP.

*Type locality*: **USA**, New Jersey, Gloucester, Newfield.

*Type substrate*: *Phyllachora flabella* on *Pteris aquilina*.  

*pucciniophilum Fusarium* Sacc. & P. Syd., Syll. Fung. 14: 1128. 1899.

*Replaced synonym*: *Fusarium parasiticum* Ellis & Kellerm., J. Mycol. 3 (11): 127. 1887, *nom. illegit.*, Art. 53.1.

(See ***Fusarium heterosporum***)

*Holotypus*: Kellerman & Swingle no. 1104 in NY.

*Type locality*: **USA**, Kansas, Manhattan.

*Type substrate*: Parasitic on *Puccinia seymeriae* on leaves of *Solidago macrophylla*.

*Note*: Synonym *fide*Wollenweber & Reinking (1935).  

*pulvinatum Fusarium* (Link) Nees, Syst. Pilze: 32. 1817.

*Basionym*: *Atractium pulvinatum* Link, Mag. Ges. Naturf. Freunde Berlin 8: 32. 1816.

*Holotypus*: Not located.

*Type locality*: **Poland**, Wrocław.

*Type substrate*: Hanging scrub branches.

*Notes*: Status unclear. Not treated by any of Wollenweber & Reinking (1935), Booth (1971), or Gerlach & Nirenberg (1982).  

*pulvinatum Fusarium* (Berk. & Broome) Sacc., Syll. Fung. 4: 699. 1886, *nom. illegit.*, Art. 53.1.

*Basionym*: *Fusisporium pulvinatum* Berk. & Broome, J. Linn. Soc., Bot. 14: 102. 1873 [1875].

(See ***Fusarium sambucinum***)

*Holotypus*: In K(M).

*Type locality*: **Sri Lanka**.

*Type substrate*: Bark.

*Note*: Synonym *fide*Wollenweber & Reinking (1935).  

*punctiforme Fusarium* Durieu & Mont., Expl. Sci. Algérie 1: 335. 1848.

(See ***Fusarium reticulatum***)

*Holotypus*: Not located.

*Type locality*: **Algeria**.

*Type substrate*: *Citrus aurantium*.

*Note*: Synonym *fide*Wollenweber & Reinking (1935).  

*purpurascens Fusarium* Maryani *et al.*, Stud. Mycol*.* 92: 160. 2018 [2019].

(see ***Fusarium odoratissimum***)

*Holotypus*: InaCC F886 (preserved as metabolically inactive culture).

*Ex-type culture*: InaCC F886.

*Type locality*: **Indonesia**, East Kalimantan, Kampung Salak Martadinata.

*Type substrate*: *Musa* var. Pisang Kepok*.*

*Descriptions and illustrations*: See Maryani *et al.* (2019a).

*Diagnostic DNA barcodes*: *rpb2*: LS479385; *tef1*: LS479827.  

*pusillum Fusarium* Wollenw., Fusaria Autogr. Delin. 2: 550. 1924.

(See *Fusarium dimerum*)

*Lectotypus* (*hic designatus*, MBT 10000727): **Germany**, *Solanum tuberosum*, 1919, H.W. Wollenweber, in Fusaria Autogr. Delin. 2: 550. 1924.

*Note*: As no holotype specimen could be located, an illustration accompanying the original protologue is designated here as lectotype.  

*putaminum Fusarium* (Thüm.) Sacc., Syll. Fung. 4: 703. 1886.

*Basionym*: *Fusisporium putaminum* Thüm., Oesterr. Bot. Z. 27: 272. 1877.

(See ***Fusarium lateritium***)

*Holotypus*: Not located.

*Type locality*: **Austria**, Klosterneuburg.

*Type substrate*: *Prunus domestica*.

*Note*: Synonyms *fide*Wollenweber & Reinking (1935).  

*putrefaciens Fusarium* Osterw., Mitth. Thurgauischen Naturf. Ges. 16: 123. 1904.

(See ***Fusarium avenaceum***)

*Lectotypus* (*hic designatus*, MBT 10000728): **Switzerland**, Zürich, fruit and seeds of *Pyrus* sp., 1904, collector unknown, in Osterwalder, Mitth. Thurgauischen Naturf. Ges. 16, tab. 2, figs 10–30.

*Notes*: Synonym *fide*Wollenweber & Reinking (1935). As no holotype specimen could be located, an illustration accompanying the original protologue is designated here as lectotype.  

*pyrinum Fusarium* Schwein., Trans. Amer. Philos. Soc., n.s. 4: 302. 1834, unavailable, see Art. F.3.4.

(See ***Fusarium lactis***)

*pyrinum Fusarium* (Fr.) Sacc., Syll. Fung. 4: 720. 1886.

*Basionym*: *Fusisporium pyrinum* Fr., Syst. Mycol. 3: 445. 1832, *nom. sanct.*

(See ***Fusarium avenaceum***)

*Holotypus*: Not located.

*Type locality*: **Sweden**.

*Type substrate*: Rotten fruit of *Pyrus communis*.

*Note*: Synonym *fide*Wollenweber & Reinking (1935).  

*pyrochroum Fusarium* (Desm.) Sacc., Michelia 1: 534. 1879.

***Calonectria pyrochroa*** (Desm.) Sacc., Michelia 1: 308. 1878.

*Basionym*: *Selenosporium pyrochroum* Desm., Ann. Sci. Nat., Bot., sér. 3, 14: 111. 1850.

*Synonyms*: *Nectria pyrochroa* Desm., Pl. Crypt. N. France, ed. 2: no. 372. 1856.

*Calonectria daldiniana* De Not., Comment. Soc. Crittog. Ital. 2: 477. 1867.

*Fusarium pyrochroum* var. *diatrypellicola* P. Syd., Mycoth. March., Cent. 41: no. 4063. 1893.

*Nectria abnormis* Henn., Hedwigia 36: 219. 1897.

*Holotypus*: In ?PAD or PC.

*Type locality*: **France**.

*Type substrate*: *Sambucus nigra*.  

*quercicola Fusarium* Oudem., Ned. Kruidk. Arch., sér. 3, 2: 777. 1902.

*Holotypus*: ?L.

*Type locality*: **Netherlands**, Noord-Holland Province, Bussum.

*Type substrate*: *Quercus rubra*.

*Note*: Not *Fusarium fide*Wollenweber & Reinking (1935).  

*quercinum Fusarium* O'Donnell *et al.*, Index Fungorum 440: 4. 2020.

***Neocosmospora quercicola*** Sand.-Den. & Crous, Persoonia 43: 159. 2019.

*Holotypus*: CBS H-23998.

*Ex-type culture*: CBS 141.90 = NRRL 22652.

*Type locality*: **Italy**.

*Type substrate*: *Quercus cerris*.

*Descriptions and illustrations*: See Sandoval-Denis *et al.* (2019).

*Diagnostic DNA barcodes*: *rpb1*: MW834247; *rpb2*: LR583869; *tef1*: DQ247634.  

*radicicola Fusarium* Wollenw., J. Agric. Res. 2: 257. 1914.

(See *Fusarium solani*)

*Lectotypus*: Plate XVI, fig. K, in Wollenweber (1914), designated in Sandoval-Denis *et al.* (2019).

*Lectotype locality*: **USA**, Washington.

*Lectotype substrate*: *Solanum tuberosum*.

*Note*: Synonym *fide*Wollenweber & Reinking (1935) & Sandoval-Denis *et al.* (2019).  

***ramigenum Fusarium*** O'Donnell & Nirenberg, Mycologia 90: 451. 1998.

*Holotypus*: B 70 0001687.

*Ex-type culture*: BBA 68592 = CBS 418.97 = DAOM 225137 = IMI 375343 = NRRL 25208.

*Type locality*: **USA**, California.

*Type substrate*: *Ficus carica.*

*Descriptions and illustrations*: See Nirenberg & O'Donnell (1998).

*Diagnostic DNA barcodes*: *rpb1*: KF466401; *rpb2*: KF466412; *tef1*: AF160267.  

*ramosum Fusarium* (Batista & H. Maia) O'Donnell *et al.*, Index Fungorum 440: 4. 2020.

*Basionym*: *Hyaloflorea ramosa* Bat. & H. Maia, Anais Soc. Biol. Pernambuco 13: 155. 1955.

*Synonyms*: *Neocosmospora ramosa* (Bat. & H. Maia) L. Lombard & Crous, Stud. Mycol. 80: 227. 2015.

(See *Fusarium lichenicola* C. Massal.)

*Holotypus*: IMUR 410.

*Ex-type culture*: CBS 509.63 = IMUR 410 = MUCL 8050.

*Type locality*: **Brazil**.

*Type substrate*: Air.

*Diagnostic DNA barcodes*: *rpb2*: LR583843; *tef1*: LR583618.

*Note*: Synonymies *fide*Sandoval-Denis & Crous (2018).  

*ramulicola Fusarium* Sawada, Special Publ. Coll. Agric. Natl. Taiwan Univ. 8: 228. 1959, *nom. inval*., Art. 39.1.

*Authentic material*: Not located.

*Original locality*: **Taiwan**.

*Original substrate*: Branches of *Citrus tankan f. koshotankan*.

*Note*: This name is invalid because of missing Latin diagnosis.  

*rectiphorum Fusarium* Samuels *et al.* (as ‘*rectiphorus*’), Mycologia 103: 1324. 2011.

***Neocosmospora rectiphora*** Samuels *et al.*, Mycologia 103: 1324. 2011.

*Neocosmospora bomiensis* Z.Q. Zeng & W.Y. Zhuang, Phytotaxa 319: 177. 2017.

*Holotypus*: BPI 881229.

*Ex-type culture*: CBS 125727 = FRC S-1831 = G.J.S. 02-89.

*Type locality*: **Sri Lanka**, Wagamba Province, vic. Kurunegala, Arangakele.

*Type substrate*: Bark*.*

*Descriptions and illustrations*: See Nalim *et al.* (2011).

*Diagnostic DNA barcodes*: *rpb1*: MW834249; *rpb2*: LR583871; *tef1*: LR583641.  

***redolens Fusarium*** Wollenw., Phytopathology 3: 29. 1913 and Ber. Deutsch. Bot. Ges. 31: 31. 1913.

*Synonyms*: *Fusarium oxysporum* var. *redolens* (Wollenw.) W.L. Gordon, Canad. J. Bot. 30: 238. 1952.

*Fusarium solani* var. *redolens* (Wollenw.) Bilaĭ, Fusarii (Biologija i sistematika): 288. 1955.

?*Fusarium retusum* Wellman, Phytopathology 33: 957. 1943.

*Holotypus*: Not located.

*Type locality*: **Unknown**.

*Type substrate*: *Pisum sativum*.

*Lectotypus* (*hic designatus*, MBT 10000729): **Unknown**, *Pisum sativum*, 1913, H.W. Wollenweber, in Phytopathology 3: 31, fig. E.

*Epitypus* (*hic designatus*, MBT 10000730): **Germany**, Berlin-Dahlem, vascular bundle of *Dianthus caryophyllus*, 16 May 1959, D. Hantschke & W. Gerlach, CBS 360.87 (preserved as metabolically inactive culture).

*Ex-epitype culture*: ATCC 16067 = BBA 9526 = CBS 248.61 = CBS 360.87 = DSM 62390 = NRRL 20426 = NRRL 25600.

*Descriptions and illustrations*: See Gerlach & Pag (1961), Gerlach & Nirenberg (1982) and Leslie & Summerell (2006).

*Diagnostic DNA barcodes*: *rpb1*: MT409433; *rpb2*: MT409443; *tef1*: MT409453.

*Notes*: As both protologue publications occurred more or less simultaneously for *F. redolens*, we select the illustration provided in Phytopathology as lectotype, since no holotype material could be located. Gerlach & Nirenberg (1983) considered CBS 248.61 (= CBS 360.87) a good representative of *F. redolens*, which was initially designated by Gerlach & Pag (1961) as representative of *F. redolens f. sp. dianthi.* Therefore, an epitype is designated here to provide taxonomic stability for this species.  

*regulare Fusarium* (Sand.-Den. & Crous) O'Donnell *et al.*, Index Fungorum 440: 4. 2020.

***Neocosmospora regularis*** Sand.-Den. & Crous, Persoonia 43: 162. 2019.

*Holotypus*: CBS H-23999.

*Ex-type culture*: CBS 230.34

*Type locality*: **Netherlands**, Zeeland Province, Zuid Beveland, near Kloetinge.

*Type substrate*: *Pisum sativum*.

*Descriptions and illustrations*: See Sandoval-Denis *et al.* (2019).

*Diagnostic DNA barcodes*: *rpb2*: LR583873; *tef1*: LR583643.  

*rekanum Fusarium* Lynn & Marinc., Antonie van Leeuwenhoek 113: 816. 2020.

***Neocosmospora rekana*** (Lynn & Marinc.) L. Lombard & Sand.-Den., ***comb. nov*.** MycoBank MB 837706.

*Basionym*: *Fusarium rekanum* Lynn & Marinc., Antonie van Leeuwenhoek 113: 816. 2020.

*Holotypus*: PREM 62333.

*Ex-type culture*: CMW 52862 = PPRI 27163.

*Type locality*: **Indonesia**, Sumatra, Riau, Pelalawan.

*Type substrate*: *Acacia crassicarpa* infested with *Euwallacea perbrevis*.

*Descriptions and illustrations*: See Lynn *et al.* (2020).

*Diagnostic DNA barcodes*: *rpb2*: MN249137, MN249108; *tef1*: MN249151.

*Note*: Based on the phylogenetic position of this species related to the ‘ambrosia’ clade as illustrated by Lynn *et al.* (2020), we provide a new combination in the genus *Neocosmospora*.  

***reticulatum Fusarium*** Mont., Ann. Sci. Nat., Bot., sér. 2, 20: 379. 1843.

*Synonyms*: ?*Fusarium leucoconium* Corda, Icon. Fung. 1: 4. 1837. (*fide* Wollenweber & Reinking 1935).

?*Fusarium punctiforme* Durieu & Mont., Expl. Sci. Algérie 1: 335. 1848.

*Fusisporium flavidum* Bonord., Bot. Zeitung (Berlin) 19: 194. 1861.

*Fusarium flavidum* (Bonord.) Sacc., Syll. Fung. 4: 698. 1886.

*Fusarium ampelodesmi* Fautrey & Roum., in Roumeguère, Rev. Mycol. (Toulouse) 13: 82. 1891.

*Fusarium epithele* McAlpine, *Fungus Diseases of Citrus trees in Australia*: 80. 1899.

*Fusarium orchidis* Petch, Ann. Roy. Bot. Gard. (Peradeniya) 6: 256. 1917.

*Fusarium negundinis* Sherb., in Hubert, J. Agric. Res. 26: 451. 1923.

*Fusarium reticulatum* var. *negundinis* (Sherb.) Wollenw., Z. Parasitenk. (Berlin) 3: 351. 1931.

*Fusarium heterosporum* var. *negundinis* (Sherb.) Raillo, Fungi of the Genus Fusarium: 217. 1950.

*Fusarium reticulatum* var. *medium* Wollenw., Z. Parasitenk. (Berlin) 3: 358. 1931.

*Lectotypus* (*hic designatus*, MBT 10000731): **France**, Nouvelle-Aquitaine, Saint-Sever, *Citrullus* sp., 1843, L. Dufour, in Montagne, Ann. Sci. Nat., Bot., 2 sér. 20: 379: pl. 16, fig. 3.

*Epitypus* (*hic designatus*, MBT 10000732): **Germany**, Rellingen/Holstein, bark lesion of *Sophora japonica*, Jun. 1976, R. Schwarz, CBS 473.76 (preserved as metabolically inactive).

*Ex-epitype culture*: BBA 63657 = CBS 473.76 = NRRL 20684.

*Descriptions and illustrations*: See Gerlach & Nirenberg (1982).

*Diagnostic DNA barcodes*: *rpb1*: MW928816; *tef1*: MW928841.

*Notes*: Gerlach & Nirenberg (1983) considered CBS 473.76 a good representative of *F. reticulatum*. As no holotype specimen could be located, an illustration is designated as lectotype here and an epitype is designated to provide taxonomic stability for this species.  

*retusum Fusarium* Wellman, Phytopathology 33: 957. 1943.

(See ***Fusarium oxysporum***)

*Holotypus*: Not located.

*Type locality*: **USA**, Indiana.

*Type substrate*: *Solanum lycopersicum*.  

*rhabdophorum Fusarium* Berk. & Broome, Ann. Mag. Nat. Hist., ser. 4, 17: 142. 1876.

*Holotypus*: In K(M).

*Type locality*: **UK**, Scotland, Forres.

*Type substrate*: Dead sticks.

*Notes*: Status unclear. Not *Fusarium fide*Wollenweber & Reinking (1935).  

*rhizochromatistes Fusarium* Sideris, Phytopathology 14: 212. 1924.

(See ***Fusarium oxysporum***)

*Lectotypus* (*hic designatus*, MBT 10000733): **USA**, California, Stockton, roots of *Allium cepa*, 1924, C.P. Sideris, in Phytopathology 14, pl. XI.

*Notes*: Synonym *fide*Wollenweber & Reinking (1935). No holotype specimen could be located and therefore an illustration was designated as lectotype.  

*rhizogenum Fusarium* Pound & Clem., Bot. Surv. Nebraska 3: 12. 1894.

(See *Fusarium candidum* Ehrenb*.*)

*Holotypus*: NEB0040548.

*Type locality*: **USA**, Lincoln.

*Type substrate*: Roots of *Malus domestica* seedlings.

*Note*: Synonym *fide*Wollenweber & Reinking (1935).  

*rhizogenum Fusarium* Aderh., Centralbl. Bacteriol. Parasitenk., 1. Abth., 6: 623. 1900, *nom. illegit.*, Art. 53.1.

(See *Fusarium aderholdii*)

*Authentic material*: Not located.

*Original locality*: **Germany**.

*Original substrate*: *Malus domestica*.

*Notes*: Synonym *fide*Wollenweber & Reinking (1935). The original publication could not be checked but Sorauer (1923) clearly stated that Aderhold only used the name *Fusarium rhizogenum* Pound & Clem. to describe a disease using the latter name.  

*rhizophilum Fusarium* Corda, Icon. Fung. 2: 3. 1838.

*Synonym*: *Fusisporium georginae* Klotzsch, Herb. Viv. Mycol., Cent. 2: 186. 1832, *nom. nud.*, Art. 38.1(a).

(See *Fusarium merismoides*)

*Lectotypus* (*hic designatus*, MBT 10000734): **Czech Republic**, Prague, roots of garden plants, 1838, A.C.J. Corda, in Icon. Fung. 2, Tab. VIII, fig. 15.

*Notes*: Synonym *fide*Wollenweber & Reinking (1935). No holotype specimen could be located and therefore an illustration is designated as lectotype.  

*rhizophorae Fusarium* (Dayar.) O'Donnell *et al.*, Index Fungorum 440: 4. 2020.

***Neocosmospora rhizophorae*** Dayar., Mycosphere 11: 112. 2020.

*Holotypus*: MFLU 17-2588.

*Ex-type culture*: MFLUCC 17-2461.

*Type locality*: **Thailand**, Krabi Province, Phang Nga.

*Type substrate*: Submerged wood of *Rhizophora*.

*Descriptions and illustrations*: See Dayarathne *et al.* (2020)  

*rhodellum Fusarium* McAlpine, Proc. Linn. Soc. New South Wales 24: 122. 1899.

*Lectotypus* (*hic designatus*, MBT 10000735): **Kerguelen Islands**, *Pringlea antiscorbutica*, 1899, *D. McAlpine*, in Proc. Linn. Soc. New South Wales 24: Pl. XIII, Fig. 7.

*Notes*: Not *Fusarium fide* I. Pascoe. No holotype specimen could be located and therefore an illustration is designated as lectotype.  

*rhoicola Fusarium* Fautrey, Rev. Mycol. (Toulouse) 17: 171. 1895.

(See ***Fusarium graminearum***)

*Holotypus*: ?PC.

*Type locality*: **France** via **USA**.

*Type substrate*: *Rhus toxicodendron*.

*Note*: Synonym *fide*Wollenweber & Reinking (1935).  

*ricini Fusarium* (Bérenger) Bizz., Fl. Ven. Critt. 1: 539. 1885.

*Basionym*: *Fusisporium ricini* Bérenger, Mem. Accad. Agric. Verona 44: 257. 1866.

(See ***Fusarium sambucinum***)

*Holotypus*: Not located.

*Type locality*: **Italy**.

*Type substrate*: *Ricinus communis*.

*Note*: Synonym *fide*Wollenweber & Reinking (1935).  

*rigidiusculum Fusarium* (Berk. & Broome) W.C. Snyder & H.N. Hansen, Amer. J. Bot. 32: 664. 1945.

*Basionym*: *Nectria rigidiuscula* Berk. & Broome, J. Linn. Soc., Bot. 14: 116. 1873 [1875].

(See *Fusarium colorans*)

*Holotypus*: ?K(M).

*Type locality*: ?**Sri Lanka**.

*Type substrate*: Bark.

*Note*: Synonym *fide*Wollenweber & Reinking (1935).  

*rimicola Fusarium* Sacc. (as ‘*rimicolum*’), Michelia 2: 297. 1881.

(See ***Fusarium lateritium***)

*Holotypus*: Not located.

*Type locality*: **Italy**, Padua.

*Type substrate*: *Erythrina crista-galli*.

*Note*: Synonym *fide*Wollenweber & Reinking (1935).  

*rimosum Fusarium* (Peck) Sacc., Syll. Fung. 4: 713. 1886.

*Basionym*: *Fusisporium rimosum* Peck, Rep. (Annual) New York State Mus. Nat. Hist. 30: 58. 1878.

(See *Fusarium merismoides*)

*Holotypus*: NYSf2609.

*Type locality*: **USA**, New York, Albany.

*Type substrate*: Cut ends of stalks of *Zea mays*.

*Note*: Synonym *fide*Wollenweber & Reinking (1935).  

*riograndense Fusarium* Dallé Rosa *et al.*, J. Mycol. Med. 28: 33. 2018.

***Neocosmospora riograndensis*** (Dallé Rosa *et al.*) Sand.-Den. & Crous, Persoonia 43: 165. 2019.

*Holotypus*: UFMG-CM F12570.

*Ex-type culture*: UFMG-CM F12570 = URM-7361.

*Type locality*: **Brazil**, Rio Grande do Sul, Porto Alegre, Hospital de Clínicas de Porto Alegre.

*Type substrate*: Nasal cavity of *Homo sapiens.*

*Descriptions and illustrations*: See Dallé Rosa *et al.* (2018).

*Diagnostic DNA barcodes*: *rpb2*: KX534003; *tef1*: KX534002.  

*robiniae Fusarium* Pass., Atti Reale Accad. Lincei, Rendiconti Cl. Sci. Fis., sér. 4, 7: 51. 1891.

(See ***Fusarium sarcochroum***)

*Holotypus*: ?PARMA.

*Type locality*: **Italy**, Padua.

*Type substrate*: *Robinia pseudoacacia*.

*Note*: Synonym *fide*Wollenweber & Reinking (1935).  

***robustum Fusarium*** Gerlach, Phytopathol. Z. 88: 36. 1977.

*Holotypus*: In B.

*Isotypus*: CBS H-629.

*Ex-type culture*: BBA 63667 = CBS 637.76 = FRC R-5821 = IMI 322102 = NRRL 13392.

*Type locality*: **Argentina**.

*Type substrate*: *Araucaria angustifolia.*

*Descriptions and illustrations*: See Gerlach (1977c).

*Diagnostic DNA barcodes*: *rpb2*: MW928831; *tef1*: MW928842.  

*roesleri Fusarium* Thüm., Pilze Weinst.: 51. 1878.

(See *Fusarium merismoides*)

*Lectotypus* (*hic designatus*, MBT 10000736): **Austria**, Klosterneuburg, *Vitis vinifera*, 1878, K.A.E.J. Thümen, in Pilze Weinst. Tab. 3, fig. 7.

*Notes*: Synonym *fide*Wollenweber & Reinking (1935). No holotype specimen could be located and therefore an illustration is designated as lectotype.  

*rollandianum Fusarium* Sacc., Syll. Fung. 11: 650. 1895.

*Replaced synonym*: *Fusarium cydoniae* Roum. & Fautrey, Rev. Mycol. (Toulouse) 14: 170. 1892, *nom. illegit.*, Art. 53.1, *non* Allescher 1892.

*Syntype*: ILL00220295 (Fautrey, Fungi Sel. Gall. Exs. No. 6120).

*Type locality*: **France**.

*Type substrate*: Fruit of *Cydonia vulgaris.*

*Notes*: Not *Fusarium fide*Wollenweber & Reinking (1935). Typification pending further study of the syntype lodged in ILL.  

*rosae Fusarium* (Preuss) Sacc., Syll. Fung. 4: 697. 1886.

*Basionym*: *Selenosporium rosae* Preuss, Linnaea 24: 150. 1851.

*Holotypus*: Not located; not preserved in B *fide*Holubová-Jechová *et al.* (1994).

*Type locality*: **Germany**, Hoyerswerda.

*Type substrate*: *Rosa* sp.

*Notes*: Status unclear. Not treated by any of Wollenweber & Reinking (1935), Booth (1971), or Gerlach & Nirenberg (1982).  

*roseobullatum Fusarium* Wollenw. (as ‘*roseo-bullatum*’), Fusaria Autogr. Delin. 1: 117. 1916.

*Basionym*: *Fusarium bullatum* var. *roseum* Sherb., Mem. Cornell Univ. Agric. Exp. Sta. 6: 201. 1915.

(See ***Fusarium equiseti***)

*Holotypus*: *?*CUP-007433.

*Type locality*: **USA**, Iowa.

*Type substrate*: *Solanum tuberosum.*

*Note*: Synonym *fide*Wollenweber & Reinking (1935).  

*roseolum Fusarium* (H.O. Stephens ex Berk. & Broome) Sacc., Syll. Fung. 4: 710. 1886.

*Basionym*: *Fusisporium roseolum* H.O. Stephens ex Berk. & Broome, Ann. Mag. Nat. Hist., ser. 2, 7: 178. 1851.

(See *Fusarium merismoides*)

*Holotypus*: ?K(M).

*Type locality*: **UK**, Bristol.

*Type substrate*: Decayed *Solanum tuberosum*.

*Note*: Synonyms *fide*Wollenweber & Reinking (1935).  

*roseum Fusarium* Link, Mag. Ges. Naturf. Freunde Berlin 3: 10. 1809, *nom. rej*.

(See ***Fusarium sambucinum***)

*Lectotypus*: In B, selected in Gams *et al.* (1997).

*Type locality*: **Germany**.

*Type substrate*: *Malvaceae*.

*Notes*: Gams *et al.* (1997) proposed that the name, *F. roseum* be rejected due to ambiguity surrounding the type of this species, with *F. sambucinum* taking preference. This proposal was accepted in 1999 (see Gams 1999).  

*rostratum Fusarium* Appel & Wollenw., Arbeiten Kaiserl. Biol. Anst. Land- Forstw. 8: 30. 1910 [1913].

(See ***Fusarium graminearum***)

*Lectotypus* (*hic designatus*, MBT 10000737): **Germany**, Berlin, *Triticum aestivum*, 1913, O.A. Appel & H.W. Wollenweber, in Arbeiten Kaiserl. Biol. Anst. Land- Forstw. 8: 30, Abb. 1, figs E1–E13.

*Notes*: Synonym *fide*Wollenweber & Reinking (1935). No holotype specimen could be located and therefore an illustration is designated as lectotype.  

*roumeguerei Fusarium* Sacc. (as ‘*roumegueri*’), Syll. Fung. 4: 702. 1886, *nom. illegit.*, Art. 52.1.

*Replaced synonym*: *Fusarium insidiosum* Roum., Michelia 2 (6): 132. 1880.

(See ***Fusarium lateritium***)

*Type material*: See *Fusarium insidiosum*.

*Note*: Synonym *fide*Wollenweber & Reinking (1935).  

*ruberrimum Fusarium* Delacr., Bull. Soc. Mycol. France 6: 139. 1890.

(See ***Fusarium avenaceum***)

*Holotypus*: ?PC.

*Type locality*: **France**, Paris.

*Type substrate*: *Onobrychis viciifolia*.

*Note*: Synonym *fide*Wollenweber & Reinking (1935).  

*rubi Fusarium* (G. Winter) Berl. & Voglino, Add. Syll. Fung. 1–4: 391. 1886.

*Basionym*: *Fusisporium rubi* G. Winter, in Rabenh., Fungi Eur. Extraeur Exs., Ed. Nov., Ser. Sec., Cent. 13 (resp. 33): 3280. 1885.

*Synonym*: *Ramularia rubi* (G. Winter) Wollenw., Fusaria Autogr. Delin. 1: 470. 1916.

*Cercosporella rubi* (G. Winter) Plakidas, J. Agricultural Research 54: 275. 1937.

*Syntypes*: In BPI, CHRB, CUP, F, HAL, ISC, LSUM, MSC, MU, NEB & PH (Fungi Eur. Extraeur. Exs. no. 3280).

*Type locality*: **USA**, Illinois, Cobden

*Type substrate*: *Rubus villosus*

*Note*: Status unclear *fide*Braun (1998).  

*rubicolor Fusarium* Berk. & Broome, Trans. Linn. Soc. London, Bot. 2: 68. 1883.

*Holotypus*: ?K(M).

*Type locality*: **Australia**, Queensland, Brisbane.

*Type substrate*: Leaves of *Eucalyptus* sp.

*Note*: Not *Fusarium fide*Wollenweber & Reinking (1935).  

*rubiginosum Fusarium* Appel & Wollenw., Arbeiten Kaiserl. Biol. Anst. Land- Forstw. 8: 108. 1910 [1913].

(See ***Fusarium culmorum***)

*Lectotypus (hic designatus*, MBT 10000738):**Germany**, *Solanum tuberosum*, 1913, O.A. Appel & H.W. Wollenweber, in Arbeiten Kaiserl. Biol. Anst. Land- Forstw. 8: Tab. I, figs 31–48.

*Notes*: Synonym *fide*Wollenweber & Reinking (1935). No holotype specimen could be located and therefore an illustration is designated as lectotype.  

*rubrum Fusarium* Parav., Ann. Mycol. 16: 311. 1918.

(See ***Fusarium lactis***)

*Lectotypus* (*hic designatus*, MBT 10000739): **Germany**, core of *Malus domestica* fruit, 1918, E. Paravicini, in Ann. Mycol. 16, pl. 4, figs 23–33.

*Notes*: Synonym *fide*Wollenweber & Reinking (1935). No holotype specimen could be located and therefore an illustration is designated as lectotype.  

*rusci Fusarium* (Sacc.) O'Donnell & Geiser, Phytopathology 103: 404. 2013.

*Basionym*: *Fusarium roseum* var. *rusci* Sacc., Michelia 2: 294. 1881.

*Synonyms*: *Trichofusarium rusci* (Sacc.) Bubák, Bull. Herb. Boissier, sér. 2, 6: 488. 1906.

*Pycnofusarium rusci* D. Hawksw. & Punith., Trans. Brit. Mycol. Soc. 61: 63. 1973.

*Syntype*: BPI 453152.

*Type locality*: **Italy**, Selva.

*Type substrate*: *Ruscus aculeatus*.

*Notes*: Examination of the syntype (BPI 453152) revealed that this species does not belong to the genus *Fusarium*, having a myrothecium-like morphology. Also see notes under *Nothofusarium devonianum*.  

*russianum Fusarium* Manns, Bull. North Dakota Agric. Exp. Sta. 259: 34. 1932.

(See ***Fusarium acuminatum***)

*Holotypus*: Not located.

*Type locality*: **USA**, North Dakota.

*Type substrate*: *Linum usitatissimum*.

*Note*: Synonym *fide*Wollenweber & Reinking (1935).  

*ruticola Fusarium* Fautrey & Roum. (as ‘*rutaecola*’), Rev. Mycol. (Toulouse) 13: 82. 1891.

(See ***Fusarium avenaceum***)

*Syntype*: ?PC (Fungi Sel. Gall. Exs. No. 5686).

*Type locality*: **France**, Noidan.

*Type substrate*: *Ruta graveolens*.

*Note*: Synonym *fide*Wollenweber & Reinking (1935).  

*saccardoanum Fusarium* P. Syd., Syll. Fung. 14: 1128. 1899.

*Replaced synonym*: *Fusarium sclerodermatis* Peck, Rep. (Annual) Regents Univ. State New York New York State Mus. 43: 77. 1890, *nom. illegit.*, Art. 53.1, *non Fusarium sclerodermatis* Oudem. 1889.

(See ***Fusarium oxysporum***)

*Holotypus*: NYSf2731.

*Type locality*: **USA**, New York, Suffolk, Manor, Long Island.

*Type substrate*: *Scleroderma vulgaris*.

*Note*: Synonym *fide*Wollenweber & Reinking (1935).  

***sacchari Fusarium*** (E.J. Butler) W. Gams, Cephalosporium-artige Schimmelpilze: 218. 1971.

*Basionym*: *Cephalosporium sacchari* E.J. Butler, Mem. Dept. Agric. India, Bot. Ser. 6: 185. 1913.

*Synonyms*: *Fusarium neoceras* Wollenw. & Reinking, Phytopathology 15: 164. 1925.

*Gibberella sacchari* Summerell & J.F. Leslie, Mycologia 97: 719. 2005, *nom. illegit.*, Art. 53.1, *non Gibberella sacchari* Speg. 1896.

*Fusarium desaboruense* N. Maryani *et al.*, Persoonia 43: 59. 2019.

*Lectotypus*: In Mem. Dept. Agric. India, Bot. Ser. 6: 185, pl. II, figs 1–13. 1913, designated by Yilmaz *et al.* (2021).

*Epitypus*: CBS 223.76 (preserved as metabolically inactive culture), designated by Yilmaz *et al.* (2021).

*Ex-epitype culture*: BBA 63340 = CBS 223.76 = DAOM 225138 = IMI 202881 = NRRL 13999.

*Lectotype and epitype locality*: **India**.

Lectotype and epitype substrate: *Saccharum officinarum*.

*Descriptions and illustrations*: See Butler & Khan (1913), Gams (1971), Gerlach & Nirenberg (1982), Leslie *et al.* (2005) and Leslie & Summerell (2006).

*Diagnostic DNA barcodes*: *rpb1*: JX171466; *rpb2*: JX171580; *tef1*: AF160278.  

*salicicola Fusarium* Allesch. (as ‘*salicicolum*’), Ber. Bayer. Bot. Ges. 4: 39. 1896.

(See ***Fusarium avenaceum***)

*Holotypus*: In M.

*Type locality*: **Germany**, München, forest near Großhesselohe.

*Type substrate*: Dead branch of *Salix caprea*.

*Note*: Synonym *fide*Wollenweber & Reinking (1935).  

*salicinum Fusarium* Corda, Icon. Fung. 3: 33. 1839.

*Typus*: In PRM *fide*Pilat (1938).

*Type locality*: **Czech Republic**, near Prague.

*Type substrate*: Thin branches of *Salix* sp.

*Notes*: Not *Fusarium fide*Wollenweber & Reinking (1935). Lectotypification pending study of material lodged in PRM.  

*salicis Fusarium* Fuckel, Fungi Rhen. Exs., Suppl., Fasc. 7, no. 2110. 1868.

(See ***Fusarium lateritium***)

*Syntype*: S-F267709 (Fungi Rhen. Exs. no. 2110).

*Type locality*: **Germany**, Hessen, Münchau, near Hattenheim

*Type substrate*: Dry branches of *Salix triandra.*

*Notes*: Synonym *fide*Wollenweber & Reinking (1935). Typification pending further study of the syntype lodged in S.  

***salinense Fusarium*** Sand.-Den. *et al.*, Persoonia 40: 15. 2017 [2018].

*Holotypus*: CBS H-23019.

*Ex-type culture*: CBS 142420 = CPC 26973.

*Type locality*: **Italy**, Sicily, Messina, Leni.

*Type substrate*: Twigs of *Citrus sinensis*.

*Descriptions and illustrations*: See Sandoval-Denis *et al.* (2018a).

*Diagnostic DNA barcodes*: *rpb1*: LT746286; *rpb2*: LT746306; *tef1*: LT746193.  

*salmonicolor Fusarium* Berk. & M.A. Curtis, J. Linn. Soc., Bot. 10: 359. 1868 [1869].

*Synonym*: *Fusidium salmonicolor* (Berk. & M.A. Curtis) Wollenw., Fusaria Autogr. Delin. 1: 478. 1916.

*Holotypus*: In K(M).

*Type locality*: **Cuba**.

*Type substrate*: Dead twigs of unknown host.

*Note*s: Synonym *fide*Wollenweber & Reinking (1935). This taxon needs to be recombined into the genus *Neonectria* but requires further investigation.  

*samararum Fusarium* Allesch., Ber. Bayer. Bot. Ges. 4: 39. 1896.

(See ***Fusarium lateritium***)

*Holotypus*: In M.

*Type locality*: **Germany**, München, Starnberg.

*Type substrate*: Fallen fruits of *Fraxinus excelsior*.

*Note*: Synonym *fide*Wollenweber & Reinking (1935).  

***sambucinum Fusarium*** Fuckel, Fungi Rhen. Exs., Fasc. 3, no. 211. 1863, *nom. cons*.

*Synonyms*: *Fusarium roseum* Link, Mag. Ges. Naturf. Freunde Berlin 3: 10. 1809, *nom. rej*.

*Fusidium roseum* (Link) Link, Mag. Ges. Naturf. Freunde Berlin 8: 31. 1815 [1816].

*Gibberella rosea* (Link) W.C. Snyder & H.N. Hansen, Amer. J. Bot. 32: 664. 1945.

*Sphaeria pulicaris* Fr., Mykol. Hefte 2: 37. 1823.

*Gibbera pulicaris* (Fr.) Fr., Summa Veg. Scand. 2: 402. 1849.

*Botryosphaeria pulicaris* (Fr.) Ces. & De Not., Comment. Soc. Crittog. Ital. 1: 212. 1863.

*Nectria pulicaris* (Fr.) Tul. & C. Tul., Select. Fung. Carpol. 3: 63. 1865.

*Cucurbitaria pulicaris* (Fr.) Quél., Mém. Soc. Émul. Montbéliard, sér. 2, 5: 511. 1875.

*Gibberella pulicaris* (Fr.) Sacc., Michelia 1: 43. 1877.

*Fusarium sulphureum* Schltdl., Fl. Berol. 2: 139. 1824, *nom. rej*.

*Fusidium sulphureum* (Schltdl.) Link, in Willdenow, Sp. Pl. ed. 4, 6: 98. 1825.

*Fusarium discolor* var. *sulphureum* (Schltdl.) Appel & Wollenw., Arbeiten Kaiserl. Biol. Anst. Land- Forstw. 8: 115. 1910 [1913].

*Sphaeria cyanogena* Desm., Ann. Sci. Nat., Bot., sér. 3, 10: 352. 1848.

*Botryosphaeria cyanogena* (Desm.) Niessl, Verh. Naturf. Vereins Brünn 10: 197. 1872.

*Gibberella cyanogena* (Desm.) Sacc., Syll. Fung. 2: 555. 1883.

*Calonectria cyanogena* (Desm.) Lar.N. Vassiljeva, Nizshie Rasteniya, Griby i Mokhoobraznye

Dalnego Vostoka Rossii, Griby. Tom 4. Pirenomitsety i Lokuloaskomitsety: 169. 1998.

*Fusarium maydis* Kalchbr., Math. Term. Közlem. 3: 285. 1865, *nom. rej.*

*Fusisporium ricini* Bérenger, Mem. Accad. Agric. Verona 44: 257. 1866, *nom. rej.*

*Fusarium ricini* (Bérenger) Bizz., Fl. Ven. Critt. 1: 539. 1885.

*Fusarium subcarneum* P. Crouan & H. Crouan, Fl. Finistère: 14. 1867, *nom. rej.*

*Fusarium violaceum* P. Crouan & H. Crouan, Fl. Finistère: 14. 1867, *nom. illegit.*, Art. 53.1.

*Fusisporium pezizoideum* Berk. & M.A. Curtis, Grevillea 3: 147. 1875.

*Fusarium pezizoideum* (Berk. & M.A. Curtis) Sacc., Syll. Fung. 4: 711. 1886.

*Fusisporium pulvinatum* Berk. & Broome, J. Linn. Soc., Bot. 14: 102. 1873 [1875].

*Fusarium pulvinatum* (Berk. & Broome) Sacc., Syll. Fung. 4: 699. 1886, *nom. illegit.*, Art. 53.1.

*Fusarium roseum* var. *buxi* Sacc., Michelia 2: 294. 1881.

*Fusarium roseum* var. *calystegiae* Sacc., Michelia 2: 294. 1881.

*Fusarium roseum* var. *cucubali-bacciferi* Sacc., Michelia 2: 295. 1881.

*Fusarium roseum* var. *dulcamarae* Sacc., Michelia 2: 295. 1881.

*Fusarium roseum* var. *filicis* Sacc., Michelia 2: 295. 1881.

*Fusarium roseum* var. *fraxini* Therry, Cryptog. Lyonn.: 5717. 1881.

*Fusarium roseum* var. *helianti* Sacc., Michelia 2: 295. 1881.

*Fusarium roseum* var. *maydis* Sacc., Michelia 2: 295. 1881.

*Fusarium roseum* var. *phytolaccae* Sacc., Michelia 2: 294. 1881.

*Fusarium roseum* var. *rosae* Sacc., Michelia 2: 295. 1881.

*Fusarium roseum* var. *vitalbae* Sacc., Michelia 2: 294. 1881.

*Fusarium granulare* Kalchbr., Crypt. Austro-Afric., no. 1068. 1874.

*Fusarium roseum* var. *dracaenae* Roum., Fungi Sel. Gall. Exs., Cent. 19: 1869. 1882.

*Fusisporium tenuissimum* Peck, Rep. (Annual) New York State Mus. Nat. Hist. 34: 48. 1883.

*Fusarium tenuissimum* (Peck) Sacc., Syll. Fung. 4: 711. 1886.

*Fusisporium hordei* Wm.G. Sm., Diseases of field and garden crops, chiefly as are caused by fungi: 212. 1884.

*Fusarium hordei* (Wm.G. Sm.) Sacc., Syll. Fung. 11: 652. 1895.

*Gibberella pulicaris f. robiniae* P. Syd., Mycoth. March., Cent. 14: 1544. 1887.

*Fusarium tenellum* Sacc. & Briard, Rev. Mycol. (Toulouse) 7: 212. 1885.

*Fusarium asparagi* Delacr., Bull. Soc. Mycol. France 6: 99. 1890, *nom. illegit.*, Art. 53.1.

*Fusarium delacroixii* Sacc., Syll. Fung. 10: 725. 1892.

*Fusarium fraxini* Allesch., Ber. Bot. Vereines Landshut 12: 130. 1892.

*Fusarium polymorphum* Matr., Rech. Dével. Mucéd.: 84. 1892.

*Fusarium roseum* var. *lonicerae* Allesch., Ber. Bayer. Bot. Ges. 5: 22. 1897.

*Fusarium roseum f. visci* Brunaud, Actes Soc. Linn. Bordeaux 52: 149. 1897.

*Fusarium pannosum* Massee, Bull. Misc. Inform. Kew 1898: 117. 1898.

*Gibberella pulicaris* var. *subtropica* Rehm, in Theissen, Ann. Mycol. 9: 63. 1911.

*Gibberella subtropica* (Rehm) Wollenw., Fusaria Autogr. Delin. 1: 38. 1916.

*Botryosphaeria subtropica* (Rehm) Weese, Sitzungsber. Akad. Wiss. Wien, Math.-Naturwiss. Cl., Abt. 1, 128: 708. 1919.

*Fusarium genevense* Dasz., Bull. Soc. Bot. Genève, sér. 2, 4: 305. 1912.

*Fusarium discolor* Appel & Wollenw., Arbeiten Kaiserl. Biol. Anst. Land- Forstw. 8: 114. 1913.

*Fusarium subpallidum* Sherb., Mem. Cornell Univ. Agric. Exp. Sta. 6: 230. 1915.

*Fusarium roseum* var. *phaseoli* Gonz. Frag., Trab. Mus. Nac. Cienc. Nat., Ser. Bot. 10: 173. 1916.

*Fusarium aridum* O.A. Pratt, J. Agric. Res. 13: 89. 1918.

*Fusarium elongatum* O.A. Pratt, J. Agric. Res. 13: 84. 1918, *nom. illegit.*, Art. 53.1.

*Fusarium roseum* var. *zeae* Cif., Bull. Soc. Bot. Ital. 1921: 73. 1921.

*Fusarium sambucinum* var. *medium* Wollenw., Z. Parasitenk. (Berlin) 3: 358. 1931.

*Fusarium sambucinum* f2 Wollenw., Z. Parasitenk. (Berlin) 3: 357. 1931.

*Fusarium sambucinum* f3 Wollenw., Z. Parasitenk. (Berlin) 3: 357. 1931.

*Fusarium sambucinum* f4 Wollenw., Z. Parasitenk. (Berlin) 3: 357. 1931.

*Fusarium sambucinum* f6 Wollenw., Z. Parasitenk. (Berlin) 3: 358. 1931.

*Gibberella pulicaris* var. *minor* Wollenw., Z. Parasitenk. (Berlin) 3: 356. 1931.

*Fusarium roseum f. phaseoli* N. Barros, Revista Inst. Colomb. Agropecu. 1: 80. 1966.

*Fusarium roseum f. compactum* Tivoli, Agronomie 8: 220. 1988, *nom. inval*., Arts. 35.1, 39.1.

*Fusarium roseum* var. *lavaterae-arboreae* Thüm., Mycoth. Univ. Cent. 11: no. 1084. 1878.

*Lectotypus*: G00266369.

*Type locality*: **Germany**, Hessen*.*

*Type substrate*: Dead branches of *Sambucus nigra.*

*Descriptions and illustrations*: See Wollenweber & Reinking (1935), Booth (1971), Gerlach & Nirenberg (1982), Nelson *et al.* (1983), and Leslie & Summerell (2006).

*Notes*: The taxonomy of *F. sambucinum*, the type species of the genus *Fusarium*, is confusing. Divergent species concepts have been derived from multiple taxonomic systems and the conflicting application of the older name *F. roseum* (Gams *et al.* 1997, Leslie & Summerell 2006). After examination of the type material, a proposal to conserve *F. sambucinum* against several earlier names was presented (Gams *et al.* 1997) and unanimously accepted by the committee for fungal taxonomy (Gams 1999). Further older valid synonymous names are in need to be rejected, notably *Sphaeria pulicaris* and *Sphaeria cyanogena*.  

*samoense Fusarium* Gehrm., Arbeiten Kaiserl. Biol. Anst. Land- Forstw. 9: 24. 1913.

(See ***Fusarium verticillioides***)

*Lectotypus* (*hic designatus*, MBT 10000740): **Samoa**, cortex of *Theobroma cacao*, 1913, K. Gehrmann, in Arbeiten Kaiserl. Biol. Anst. Land- Forstw. 9: Abb. 6, figs 1–3.

*Notes*: Synonym *fide*Wollenweber & Reinking (1935). No holotype specimen could be located and therefore an illustration is designated as lectotype.  

*sampaioi Fusarium* Gonz. Frag., Bol. Soc. Brot. 2: 50. 1924.

*Synonym: Illosporium corallinum* Roberge, in Desmazières, Pl. Crypt. N. France, ed. 1, Fasc. 32: no. 1551. 1847 (*pr. p. fide* Hawksworth 1979).

*Marchandiomyces corallinus* (Roberge) Diederich & D. Hawksw., Mycotaxon 37: 312. 1990 (*pr. p. fide* Diederich 1990).

*Aegerita physciae* Vouaux, Bull. Trimestriel Soc. Mycol. France 30: 314. 1914.

*Holotypus*: Not indicated. Several syntypes *fide*Hawksworth (1979).

*Type locality*: **Portugal**, near Gaia, Alto da Bandeira; and near Tabuaço*.*

*Type substrate*: Lichen thallus (on *Lasallia pustulata*, *Parmelia saxatilis*, *P. soredians* and *P. exasperata*; *Physcia semipinnata*, *P. tenella*, *Phaeophyscia orbicularis* and *Physconia grisea*)*.*

*Notes*: Hawksworth (1979), after examination of a syntype, concluded that the *Fusarium* name should be rejected since the studied material was based on discordant elements. Nevertheless, examination of all available syntypes is required to confirm these observations or otherwise, to fix the use of this name by lectotypification.  

*samuelsii Fusarium* (Sand.-Den. & Crous) O'Donnell *et al.*, Index Fungorum 440: 4. 2020.

***Neocosmospora samuelsii*** Sand.-Den. & Crous, Persoonia 43: 165. 2019.

*Holotypus*: CBS H-24001.

*Ex-type culture*: CBS 114067 = G.J.S. 89-70.

*Type locality*: **Guyana**, Mount Wokomung, on ridge leading NW toward summit, 0.5–1 h walk from Base Camp.

*Type substrate*: Bark.

*Descriptions and illustrations*: See Sandoval-Denis *et al.* (2019).

*Diagnostic DNA barcodes*: *rpb1*: MW834252; *rpb2*: LR583874; *tef1*: LR583644.  

***sangayamense Fusarium*** Maryani *et al.*, Stud. Mycol. 92: 187. 2018 [2019].

*Holotypus*: InaCC F960 (preserved as metabolically inactive culture).

*Ex-type culture*: InaCC F960.

*Type locality*: **Indonesia**, South Kalimantan, Kota Baru, Sengayam.

*Type substrate*: Pseudostem of *Musa* var. Pisang Kepok.

*Descriptions and illustrations*: See Maryani *et al.* (2019a).

*Diagnostic DNA barcodes*: *rpb1*: LS479537; *rpb2*: LS479283; *tef1*: LS479732.  

*sanguineum Fusarium* Sherb., Mem. Cornell Univ. Agric. Exp. Sta. 6: 193. 1915.

(See ***Fusarium acuminatum***)

*Typus*: ?CUP-007444.

*Type locality*: **USA**, New York, Ithaca

*Type substrate*: *Solanum tuberosum.*

*Notes*: Synonym *fide*Wollenweber & Reinking (1935). Lectotypification pending study of material lodged in CUP.  

*sapindophilum Fusarium* Speg., Anales Mus. Nac. Hist. Nat. Buenos Aires 6: 351. 1898 [1899].

*Synonym*: *Cercoseptoria sapindophila* (Speg.) Cif., Mycopathol. Mycol. Appl. 6: 26. 1951.

*Holotypus*: In LPS (Fungi Argent. n.v.c. no. 868).

*Type locality*: **Argentina**, near Tucumán.

*Type substrate*: Living leaves of unknown climbing *Sapindaceae*.

*Note*: Synonym *fide*Wollenweber & Reinking (1935).  

***sarcochroum Fusarium*** (Desm.) Sacc., Michelia 1: 534. 1879.

*Basionym*: *Selenosporium sarcochroum* Desm., Ann. Sci. Nat., Bot., sér. 3, 14: 112. 1850.

*Synonyms*: *Fusarium diplosporum* Cooke & Ellis, Grevillea 7: 38. 1878.

*Fusarium desciscens* Oudem., Ned. Kruidk. Arch., sér. 2, 5: 515. 1889.

*Fusarium robiniae* Pass., Atti Reale Accad. Lincei, Rendiconti Cl. Sci. Fis., sér. 4, 7: 51. 1891.

*Fusarium sarcochroum* var. *robiniae* (Pass.) Wollenw., Z. Parasitenk. (Berlin) 3: 388. 1931.*Fusarium sarcochroum f*. *polygalae-myrtifoliae* Henn., Verh. Bot. Vereins Prov. Brandenburg 40: 174. 1898 [1899].

*Fusarium sarcochroum* var. *casei* Loubière, Rech. Mucédinées: 53. 1924.

*Gibberella pseudopulicaris* Wollenw., Z. Parasitenk. (Berlin) 3: 387. 1931.

*Neotypus* (*hic designatus*, MBT 10000741): **Switzerland**, *Viscum album*, 1977, W. Gerlach, CBS 745.79 (preserved as metabolically inactive culture).

*Ex-neotype culture*: BBA 63714 = CBS 745.79 = NRRL 20472.

*Descriptions and illustrations*: See Wollenweber & Reinking (1935), Raillo (1950), Bilaĭ (1955), Gerlach & Nirenberg (1982).

*Diagnostic DNA barcodes*: *rpb1*: JX171472; *rpb2*: JX171586; *tef1*: JABEXW010000634.

*Notes*: No type material could be located. Therefore, CBS 745.79 is designated as neotype here. Both Gerlach & Nirenberg (1982) and O'Donnell *et al.* (2013) considered this isolate an authentic representation of this species.  

*schawrowii Fusarium* Speschnew (as ‘*schawrovi*‘), Arbeiten Kaukas. Stat. Seidenzucht 10: 1906.

(See ***Fusarium lateritium***)

*Holotypus*: Not located.

*Type locality*: **Turkey**, Anatolia.

*Type substrate*: Branch of *Morus* sp.

*Note*: Synonym *fide*Wollenweber & Reinking (1935).  

*schiedermayeri Fusarium* (Thüm.) Sacc., Syll. Fung. 4: 712. 1886.

*Basionym*: *Fusisporium schiedermayeri* Thüm., Fungi Austr. Exs. Cent. 1: no. 78. 1871.

(See ***Fusarium avenaceum***)

*Syntypus*: In HAL.

*Type locality*: **Austria**, Linz.

*Type substrate*: Ovaries of *Luzula pilosa*, in association with *Ustilago luzulae*.

*Note*: Synonyms *fide*Wollenweber & Reinking (1935).  

*schnablianum Fusarium* Allesch., Hedwigia 34: 289. 1895.

(See ***Fusarium avenaceum***)

*Holotypus*: In M.

*Type locality*: **Germany**, Großhesselohe, near München.

*Type substrate*: Decorticated branch of *Acer pseudoplatanus*.

*Note*: Synonym *fide*Wollenweber & Reinking (1935).  

*schribauxii Fusarium* Delacr., Bull. Soc. Mycol. France 6: 99. 1890.

(See ***Fusarium culmorum***)

*Holotypus*: ?PC.

*Type locality*: **France**.

*Type substrate*: Seeds of *Triticum sativum*, in association with *Trichothecium roseum*.

*Note*: Synonym *fide*Wollenweber & Reinking (1935).  

*schweinitzii Fusarium* Ellis & Harkn., Bull. Torrey Bot. Club 8: 27. 1881.

***Colletotrichum crassipes*** (Speg.) Arx, Verh. Kon. Akad. Wetensch., Afd. Natuurk., Sect. 2, 51: 77. 1957.

*Basionym*: *Gloeosporium crassipes* Speg., Rivista Vitic. Enol. 2: 405. 1878.

*Syntypes*: In CHRB, CUP, ILL, MICH, MU, NEB, NYS, PH, PUL & WIS (Ellis, N. Amer. Fungi no. 539).

*Type locality*: **USA**, New Jersey, Newfield.

*Type substrate*: *Vitis* sp. vine

*Note*: Synonym *fide*Wollenweber & Reinking (1935).  

***scirpi Fusarium*** Lambotte & Fautrey, Fautrey, Fungi Sel. Gall. Exs. no. 6540. 1893.

*Synonyms*: ?*Fusoma helminthosporii* Corda, Icon. Fung. 1: 7. 1837.

?*Fusoma filiferum* Preuss, Linnaea 25: 73. 1852.

?*Fusarium filiferum* (Preuss) Wollenw., Fusaria Autogr. Delin. 1: 220. 1916.

?*Fusarium scirpi* var. *filiferum* (Preuss) Wollenw., Fusaria Autogr. Delin. 3: 936. 1930.

?*Fusisporium chenopodinum* Thüm., Mycoth. Univ., Cent. 14: no. 1378. 1879.

?*Fusarium chenopodinum* (Thüm.) Sacc., Syll. Fung. 4: 701. 1886.

?*Fusarium aloes* Kalchbr. & Cooke (as ‘*aloës*’), Grevillea 9: 23. 1880.

?*Fusarium osteophilum* Speg., Anales Soc. Ci. Argent. 10: 60. 1880.

?*Fusisporium mucophytum* W.G. Sm., Gard. Chron. n.s. 22: 245. 1884.

?*Fusarium mucophytum* (W.G. Sm.) Massee, Brit. Fung.-Fl. 3: 483. 1893.

*Fusarium equiseticola* Allesch., Hedwigia 34: 289. 1895.

*Fusarium sclerotium* Wollenw., Ber. Deutsch. Bot. Ges. 31: 301. 1913.

*Fusarium caudatum* Wollenw., J. Agric. Res. 2: 262. 1914.

*Fusarium sclerodermatis* var. *lycoperdonis* Picb., Bull. Ecol. Sup. Agron., Brno, R.C.S. Fac. Silvicult. 13: 27. 1929.

*Fusarium scirpi* var. *comma* Wollenw., Fusaria Autogr. Delin. 3: 922. 1930.

*Fusarium scirpi* var. *nigrantum* F.T. Benn. (as ‘*nigrans*’), Ann. Appl. Biol. 19: 26. 1932.

*Fusarium scirpi* var. *pallens* F.T. Benn., Ann. Appl. Biol. 19: 21. 1932.

*Lectotypus* (*hic designatus*, MBT 10000742): **France**, *Schoenoplectus lacustris* (= *Scirpus lacustris*), 1893, F. Fautrey, ILL00220730 (Fautrey, Fungi Sel. Gall. Exs. No. 6540).

*Epitypus* (*hic designatus*, MBT 10000743): **Australia**, New South Wales, near Broken Hill, pasture soil, 1981, P.E. Nelson, CBS H-24069.

*Ex-epitype culture*: CBS 447.84 = FRC R-6252 = NRRL 36478.

*Descriptions and illustrations*: See Wollenweber (1916–1935), Wollenweber & Reinking (1935), Burgess *et al.* (1985) and Leslie & Summerell (2006).

*Diagnostic DNA barcodes*: *rpb2*: GQ505832; *tef1*: GQ505654.

*Notes*: The epitypification of *Fusarium scirpi* by Xia *et al.* (2019) was not Code compliant as the holo- or lectotype was not correctly indicated (Art. 9.9). Here, the lectotype is clearly indicated, making the epitypification valid.  

*sclerodermatis Fusarium* Oudem., Ned. Kruidk. Arch., sér. 2, 5: 516. 1889.

(See ***Fusarium torulosum***)

*Holotypus*: ?L.

*Type locality*: **Netherlands**, Zuid-Holland Province, Scheveningen.

*Type substrate*: Rotten *Scleroderma vulgaris*.

*Note*: Synonym *fide*Nirenberg (1995).  

*sclerodermatis Fusarium* Peck, Rep. (Annual) Regents Univ. State New York New York State Mus. 43: 77. 1890, *nom. illegit.*, Art. 53.1.

(See ***Fusarium oxysporum***)

*Authentic material*: NYSf2731.

*Original locality*: **USA**, New York, Suffolk.

*Original substrate*: Peridium of *Scleroderma vulgaris*.

*Notes*: A later homonym of *F. sclerodermatis* Oudem. Saccardo (1892) published *F. peckii* as a replacement name which was again an illegitimate homonym; the taxon was later synonymised with *F. oxysporum* var. *aurantiacum* (Wollenweber & Reinking 1935).  

*sclerostromaton Fusarium* Sideris, Phytopathology 14: 213. 1924.

(See ***Fusarium oxysporum***)

*Holotypus*: Not located.

*Type locality*: **USA**, California, Delta, near Stockton.

*Type substrate*: Roots of *Allium* sp. with symptoms of pink root disease.

*Note*: Synonym *fide*Wollenweber & Reinking (1935).  

*sclerotioides Fusarium* Sherb., Mem. Cornell Univ. Agric. Exp. Sta. 6: 214. 1915.

(See ***Fusarium oxysporum***)

*Typus*: ?BPI 452971.

*Type locality*: **USA**, New York, Ithaca.

*Type substrate*: *Solanum tuberosum.*

*Notes*: Synonym *fide*Wollenweber & Reinking (1935). Typification pending further study of the specimen lodged in BPI.  

*sclerotioides Fusarium* (Höhn.) Samuels & Rossman, Mycological Papers 164: 23. 1991, *nom. illegit.*, Art. 53.1

*Basionym*: *Stagonopsis sclerotioides* Höhn., in Penther & Zederbauer, Ann. K. K. Naturhist. Hofmus. 20: 368. 1905.

(See *Fusarium kurdicum*)

*Holotypus*: FH00965353.

*Type locality*: **Turkey**, near Erciyes Dağı.

*Type substrate*: Thin twigs of *Astragalus* sp.  

*sclerotium Fusarium* Wollenw., Ber. Deutsch. Bot. Ges. 31: 30. 1913.

(See ***Fusarium scirpi***)

*Holotypus*: Not located.

*Type locality*: **USA**.

*Type substrate*: *Citrullus vulgaris* and *Lycopersicon esculentum*.

*Note*: Synonym *fide*Nirenberg (1995).  

*scolecoides Fusarium* Sacc. & Ellis, Miscellanea Mycologia 2: 18. 1885.

(See *Fusarium ciliatum*)

*Holotypus*: In PAD.

*Type locality*: **USA**, Pennsylvania, Bethlehem.

*Type substrate*: Branch of *Robinia* sp.

*Note*: Synonym *fide*Wollenweber & Reinking (1935).  

*secalis Fusarium* Fée, Mém. Soc. Mus. Hist. Nat. Strassbourg 3: 35. 1843.

(See ***Fusarium heterosporum***)

*Holotypus*: Not located.

*Type locality*: **France**.

*Type substrate*: Spikes of *Secale cereale*.

*Note*: Synonym *fide*Wollenweber & Reinking (1935).  

*secalis Fusarium* Jacz., Bull. Trimestriel Soc. Mycol. France 28: 346. 1912, *nom. illegit.*, Art. 53.1.

(See *Fusarium nivale*)

*Authentic material*: Not located.

*Original locality*: **Russia**, near Moscow.

*Original substrate*: Grain of *Secale* sp.

*Note*: Synonym *fide*Wollenweber & Reinking (1935).  

***secorum Fusarium*** Secor *et al.*, Fungal Biology 118: 767. 2014.

*Holotypus*: BPI 892692.

*Ex-type culture*: NRRL 62593.

*Type locality*: **USA**, Minnesota, Sabin.

*Type substrate*: Root of *Beta vulgaris*.

*Descriptions and illustrations*: See Secor *et al.* (2014).

*Diagnostic DNA barcodes*: *rpb1*: JABEEM010001657; *rpb2*: JABEEM010001483; *tef1*: KJ189225.  

*sedimenticola Fusarium* M.M. Wang *et al.*, Botanica Marina 63: 174. 2020.

(See *Fusarium keratoplasticum*)

*Holotypus*: HAMS 248044.

*Ex-type culture*: CGMCC 3.19499 = LC12845.

*Type locality*: **China**, South-West Indian Ocean.

*Type substrate*: Deep-sea sediments.

*Descriptions and illustrations*: See Jones *et al.* (2020).

*Diagnostic DNA barcodes*: *rpb2*: MK190729; *tef1*: MK190727.

*Notes*: *Fusarium sedimenticola* was recently introduced by Jones *et al.* (2020) in the FSSC (=*Neocosmospora*) isolated from deep-sea sediment in the Indian Ocean. However, based on comparisons of both protologues and sequences using a larger sampling of *N. keratoplastica* isolates (results not shown), we consider *F. sedimenticola* a synonym under *N. keratoplastica*.  

*seemenianum Fusarium* Henn., in Seemen, Allg. Bot. Z. Syst. 2: 83. 1896.

(See ***Fusarium avenaceum***)

*Holotypus*: B 70 0100194.

*Type locality*: **Germany**, Borkum.

*Type substrate*: Leaves of *Platanthera bifolia* var. *robusta*.

*Note*: Synonym *fide*Wollenweber & Reinking (1935).  

*semitectum Fusarium* Berk. & Ravenel, Grevillea 3: 98. 1875.

(See ***Fusarium incarnatum***)

*Holotypus*: ?K(M).

*Type locality*: **USA**, Pennsylvania, Philadelphia.

*Type substrate*: Petioles of *Musa* sp.

*serjaniae Fusarium* Syd. & P. Syd., Beibl. Hedwigia 40: (2). 1901.

*Synonym*: *Cercospora serjaniae* (Syd. & P. Syd.) Wollenw., Z. Parasitenk. (Berlin) 3: 496. 1931.

*Holotypus*: S-F45658.

*Type locality*: **Mexico**, Puebla, Tehuacán.

*Type substrate*: Leaves of *Serjania racemosa*.

*Notes*: Status unclear. Not *Fusarium fide*Wollenweber & Reinking (1935) and not *Cercospora fide* Crous & Braun (2003).  

***serpentinum Fusarium*** J.W. Xia *et al.*, Persoonia 43: 217. 2019.

*Holotypus*: CBS H-24070.

*Ex-type culture*: BBA 62209 = CBS 119880 = MRC 1813.

*Type locality*: **Unknown**.

*Type substrate*: Unknown.

*Descriptions and illustrations*: See Xia *et al.* (2019).

*Diagnostic DNA barcodes*: *rpb2*: MN170432; *tef1*: MN170499.  

*setosum Fusarium* Nirenberg & Samuels, Canad. J. Bot. 67: 3372. 1989.

***Setofusarium setosum*** (Samuels & Nirenberg) Sand.-Den. & Crous, Stud. Mycol. 98 (no. 100116): 75. 2021.

*Synonym*: *Nectria setofusarii* Samuels & Nirenberg (as ‘*setofusariae*’), Canad. J. Bot. 67: 3372. 1989.

*Holotypus*: NY00927992.

*Type locality*: **French Guiana**, Piste de Saint-Elie, Km 16 on road between Sinnamary and St. Elie, ORSTOM research area, "ECEREX".

*Type substrate*: Bark of living liana.

*Epitypus*: CBS H-24723, designated in this study.

*Ex-epitype culture*: CBS 635.92 = G.J.S. 88-12 = NRRL 36526.

*Epitype locality*: **French Guiana**, Cayenne, 15 km from Remise, trail to Vidal-old farm, secondary forest.

*Epitype substrate*: Bark.

*Descriptions and illustrations*: See Samuels & Nirenberg (1989).

*Diagnostic DNA barcodes*: *rpb1*: JX171539; *rpb2*: JX171651; *tef1*: MW834294.  

***sibiricum Fusarium*** Gagkaeva *et al.*, Int. J. Food Microbiol. 147: 64. 2011.

*Holotypus*: LEP 12652.

*Ex-type culture*: MFG 11013 = NRRL 53430.

*Type locality*: **Russia**, Khabarovsk.

*Type substrate*: Grain of *Avena sativa*.

*Descriptions and illustrations*: See Yli-Mattila *et al.* (2011).

*Diagnostic DNA barcodes*: *rpb1*: MW233302; *rpb2*: HQ154472; *tef1*: HM744684.  

***siculi Fusarium*** Sand.-Den. *et al.*, Persoonia 40: 17. 2017 [2018].

*Holotypus*: CBS H-23021.

*Ex-type culture*: CBS 142422 = CPC 27188.

*Type locality*: **Italy**, Sicily, Catania, Paternó.

*Type substrate*: *Citrus sinensis*.

*Descriptions and illustrations*: See Sandoval-Denis *et al.* (2018a).

*Diagnostic DNA barcodes*: *rpb1*: LT746299; *rpb2*: LT746327; *tef1*: LT746214.  

*silvicola Fusarium* (Sand.-Den. & Crous) O'Donnell *et al.*, Index Fungorum 440: 4. 2020.

***Neocosmospora silvicola*** Sand.-Den. & Crous, Persoonia 43: 167. 2019.

*Synonyms*: *Fusarium solani f. robiniae* Matuo & Y. Sakurai, Ann. Phytophathol. Soc. Japan 30: 35. 1965.

*Hypomyces solani f*. *robiniae* Matuo & Y. Sakurai, Ann. Phytophathol. Soc. Japan 30: 35. 1965.

*Nectria solani f. robiniae* (Matuo & Y. Sakurai) G.R.W. Arnold, Z. Pilzk. 37: 193. 1972.

*Holotypus*: CBS H-24002.

*Ex-type culture*: CBS 123846 = G.J.S. 04-147.

*Type locality*: **USA**, Tennessee, Great Smoky Mountains National Park.

*Type substrate*: Fallen trunk of *Liriodendron tulipifera*.

*Descriptions and illustrations*: See Sandoval-Denis *et al.* (2019).

*Diagnostic DNA barcodes*: *rpb1*: MW834254; *rpb2*: LR583876; *tef1*: LR583646.  

***sinense Fusarium*** Z.H. Zhao & G.Z. Lu (as ‘*sinensis*’), Mycologia 100: 747. 2008.

*Holotypus*: IBE 000007.

*Ex-type culture*: CBS 122710.

*Type locality*: **China**, Shandong Province, Jinan.

*Type substrate*: Seed of *Triticum aestivum*.

*Descriptions and illustrations*: See Zhao & Lu (2008).

*Diagnostic DNA barcode: tef1*: EF531235.  

*socium Fusarium* Sacc., Atti Ist. Veneto Sci. Lett. Arti, sér. 6, 2: 450. 1884.

(See ***Fusarium expansum***)

*Holotypus*: Not located.

*Type locality*: **France**, Troyes.

*Type substrate*: Cortex of *Carpinus* sp. in association with *Stilbospora* sp. and *Nectria stilbosporae*.

*Note*: Synonym *fide*Wollenweber & Reinking (1935).  

*solani Fusarium* (Mart.) Sacc., Michelia 2: 296. 1881.

***Neocosmospora solani*** (Mart.) L. Lombard & Crous, Stud. Mycol. 80: 228. 2015.

*Basionym*: *Fusisporium solani* Mart., *Die Kartoffel-Epidemie der letzten Jahre oder die Stockfäule und Räude der Kartoffeln*: 20. 1842.

*Synonyms*: *Fusisporium solani-tuberosi* Desm., Ann. Sci. Nat., Bot., sér. 3, 3: 359. 1845.

*Fusarium solani-tuberosi* (Desm.) Sacc., Syll. Fung. 4: 189. 1886.

*Pionnotes solani-tuberosi* (Desm.) Sacc., Syll. Fung. 4: 727. 1886.

*Fusisporium rhizophilum* var. *solani-tuberosi* (Desm.) Westend., Bull. Acad. Roy. Sci. Belgique, Cl. Sci. 18(2): 413. 1852.

*Fusisporium candidum* Bonord., Handb. Allg. Mykol.: 96. 1851, *nom. illegit.*, Art. 53.1, *non Fusarium candidum* (Link) Sacc. 1886

*Fusarium commutatum* Sacc., Syll. Fung. 4: 710. 1886.

*Fusarium allii-sativi* Allesch., Ber. Bot. Vereines Landshut 12: 131. 1892.

*Hymenula affinis* (Fautrey & Lambotte) Wollenw., Fusaria Autogr. Delin. 1: 484. 1916 [*pr. p. fide* Booth (1971)].

*Pionnotes viridis* Lechmere, Compt. Rend. Hebd. Séances Acad. Sci. 155: 178. 1912.

*Fusarium viride* (Lechmere) Wollenw., Fusaria Autogr. Delin. 1: 418. 1916.

*Fusarium radicicola* Wollenw., J. Agric. Res. 2: 257. 1914.

*Fusarium javanicum* var. *radicicola* (Wollenw.) Wollenw., Z. Parasitenk. (Berlin) 3: 286. 1931.

*Fusarium solani f*. *radicicola* (Wollenw.) W.C. Snyder & H.N. Hansen, Amer. J. Bot. 28: 740. 1941.

*Fusarium eumartii* C.W. Carp., J. Agric. Res. 5: 204. 1915.

*Fusarium solani* var. *eumartii* (C.W. Carp.) Wollenw., Z. Parasitenk. (Berlin) 3: 452. 1931.

*Fusarium solani f. eumartii* (C.W. Carp.) W.C. Snyder & H.N. Hansen, Amer. J. Bot. 28: 740. 1941.

*Fusarium malli* Taubenh., Bull. Texas Agric. Exp. Sta. 273: 25. 1921.

*Fusarium alluviale* Wollenw. & Reinking, Phytopathology 15: 167. 1925.

*Fusarium aduncisporum* Weimer & Harter, J. Agric. Res. 32: 312. 1926.

*Fusarium solani* var. *aduncisporum* (Weimer & Harter) Wollenw., Fusaria Autogr. Delin. 3: 1035. 1930.

*Neocosmospora rubicola* L. Lombard & Crous, Stud. Mycol. 80: 227. 2015.

*Lectotypus*: Illustration tab. III, fig. 29 in von Martius (1842), designated in Schroers *et al.* (2016).

*Epitypus*: CBS H-22335, designated in Schroers *et al.* (2016).

*Ex-epitype culture*: CBS 140079 = FRC S-2364 = NRRL 66304.

*Epitype locality*: **Slovenia**, Doljenska, Radohova.

*Epitype substrate*: Rotten tuber of *Solanum tuberosum*.

*Descriptions and illustrations*: See Wollenweber & Reinking (1935), Leslie & Summerell (2006), and Schroers *et al.* (2016).

*Diagnostic DNA barcodes*: *rpb1*: MW218134; *rpb2*: KT313623; *tef1*: KT313611.  

*solani-melongenae Fusarium* O'Donnell *et al.*, Index Fungorum 440: 4. 2020.

***Neocosmospora ipomoeae*** (Halst.) L. Lombard & Crous, Stud. Mycol. 80: 227. 2015.

*Basionym*: *Nectria ipomoeae* Halst., Rep. (Annual) New Jersey Agric. Exp. Sta. 12: 281. 1891.

*Synonyms*: *Cucurbitaria ipomoeae* (Halst.) Kuntze, Revis. Gen. Pl. 3: 461. 1898.

*Creonectria ipomoeae* (Halst.) Seaver, N. Amer. Fl. 3: 22. 1910.

*Hypomyces ipomoeae* (Halst.) Wollenw., Phytopathology 3: 34. 1913.

*Haematonectria ipomoeae* (Halst.) Samuels & Nirenberg, Stud. Mycol. 42: 136. 1999.

*Nectria ipomoeae f*. *ipomoeae* Halst., Rep. (Annual) New Jersey Agric. Exp. Sta. 12: 281. 1891.

*Nectria ipomoeae* var. *ipomoeae* Halst., Rep. (Annual) New Jersey Agric. Exp. Sta. 12: 281. 1891.

*Hypomyces ipomoeae* var. *ipomoeae* (Halst.) Wollenw., Phytopathology 3: 34. 1913.

*Hypomyces ipomoeae* var. *major* Wollenw., Fusaria Autogr. Delin. 3: 826. 1930.

?*Fusarium striatum* Sherb., Cornell Univ. Agric. Exp. Sta. Mem. 6: 255. 1915

?*Fusarium solani* var. *striatum* (Sherb.) Wollenw., Z. Parasitenk. (Berlin) 3: 451. 1931.

*Holotypus*: BPI 552416.

*Type locality*: **USA**, New Jersey, Mickleton.

*Type substrate*: *Solanum melongena*.

*Note*: This species requires epitypification from the type locality and host.  

*solani-tuberosi Fusarium* (Desm.) Sacc., Syll. Fung. 4: 189. 1886.

*Basionym*: *Fusisporium solani-tuberosi* Desm., Ann. Sci. Nat., Bot., sér. 3, 3: 359. 1845.

(See *Fusarium solani*)

*Holotypus*: ?PC.

*Type locality*: **France**.

*Type substrate*: Rotten tuber of *Solanum tuberosum*.

*Note*: Synonyms *fide*Wollenweber & Reinking (1935).  

*sophorae Fusarium* Allesch., Beibl. Hedwigia 36: (164). 1897.

(See ***Fusarium lateritium***)

*Holotypus*: In M.

*Type locality*: **Germany**, Berlin, Späth'sche Baumschulen.

*Type substrate*: *Sophora japonica*.

*Note*: Synonym *fide*Wollenweber & Reinking (1935).  

*sorghi Fusarium* Henn., Ann. Mus. Congo Belge, Bot., sér. 5, 2: 105. 1907.

(See ***Fusarium avenaceum***)

*Syntype*: Vanderyst 171 in B *fide*Hein (1988).

*Type locality*: **Democratic Republic of the Congo**, Kisantu.

*Type substrate*: Spikelet of *Sorghum vulgare* (= *Sorghum bicolor*).

*Note*: Synonym *fide*Wollenweber & Reinking (1935).  

***sororula Fusarium*** Herron *et al.*, Stud. Mycol. 80: 146. 2015.

*Holotypus*: PREM 60903.

*Ex-type culture*: CBS 137242 = CMW 40578.

*Type locality*: **Colombia**, Risaralda, Angela Maria (Santa Rosa).

*Type substrate*: Stem cankers of *Pinus patula*.

*Descriptions and illustrations*: See Herron *et al.* (2015).

*Notes*: Comparisons of recently generated sequences from the living ex-type culture (CBS 137242 = CMW 40578) of *F. sororula* indicate a strain transposition or contamination by another *Fusarium* species. Therefore, this species needs to be recollected from the type locality and substrate or sequences need to be generated from the holotype specimen.  

*spartinae Fusarium* Ellis & Everh., J. Mycol. 8: 14. 1902.

***Septogloeum spartinae*** (Ellis & Everh.) Wollenw. & Reinking, *Fusarien*: 336. 1935.

*Holotypus*: NY (*fide* Index Fungorum).

*Type locality*: **USA**, California, Pacific Grove.

*Type substrate*: Leaves of *Spartina stricta*.

*Note*: Synonym *fide*Wollenweber & Reinking (1935).  

***spartum Fusarium*** S. Gargouri *et al.*, Mycologia 112: 799. 2020.

*Holotypus*: BPI 911207.

*Ex-type culture*: NRRL 66896.

*Type locality*: **Tunisia**, Kasserine Governorate.

*Type substrate*: Rhizosphere of *Macrochloa tenacissima*.

*Descriptions and illustrations*: See Gargouri *et al.* (2020).

*Diagnostic DNA barcodes*: *rpb1*: MT409439; *rpb2*: MT409449; *tef1*: MT409459.  

*spathulatum Fusarium* (Sand.-Den. & Crous) O'Donnell *et al.*, Index Fungorum 440: 4. 2020.

***Neocosmospora spathulata*** Sand.-Den. & Crous, Persoonia 43: 171. 2019.

*Holotypus*: CBS H-24003.

*Ex-type culture*: CBS 145474 = NRRL 28541 = UTHSC 98-1305.

*Type locality*: **USA**, New England.

*Type substrate*: Synovial fluid from *Homo sapiens*.

*Descriptions and illustrations*: See Sandoval-Denis *et al.* (2019).

*Diagnostic DNA barcodes*: *rpb1*: MW218137; *rpb2*: EU329542; *tef1*: DQ246882.  

*speiranthae Fusarium* Henn. (as ‘*speiranthis*’), Verh. Bot. Vereins Prov. Brandenburg 40: 174. 1898.

***Colletotrichum dematium*** (Pers.) Grove, J. Bot. 56: 341. 1918.

*Basionym*: *Sphaeria dematium* Pers., Syn. Meth. Fung.: 88. 1801.

*Synonyms*: *Exosporium dematium* (Pers.) Link, in Willdenow, Sp. pl., Ed. 4, 6: 122. 1825.

*Vermicularia dematium* (Pers.) Fr., Syst. Mycol. 3: 255. 1829.

*Lasiella dematium* (Pers.) Quél., Mém. Soc. Émul. Montbéliard sér. 2, 5: 518. 1875.

*Gloeosporium speiranthae* (Henn.) Wollenw., Fusaria Autogr. Delin. 1: 500. 1916.

*Holotypus*: In B *fide*Hein (1988).

*Type locality*: **Germany**, Berlin, botanical garden.

*Type substrate*: Leaves of *Speirantha convallarioides*.

*Notes*: Wollenweber (1916) studied and illustrated authentic material of this species, recombining it in *Gloeosporium*. The shape of the conidia is similar to species in the *Colletotrichum dematium* species complex. However, the conidia are slightly broader than those of the ex-type strain of *C. dematium* (3.5–4.5 *vs* 3–4 μm, Damm *et al.* 2009); the synonymy needs to be confirmed. Publication data cited in name repositories (Allg. Bot. Z. Syst. 2: 83. 1896.) are incorrect and instead refer to the protologue of *F. seemenianum* (syn. *F. avenaceum*), an unrelated taxon.  

*speiseri Fusarium* Lindau, Rabenh. Krypt.-Fl. Ed. 2, 1(9): 580. 1909.

(See ***Fusarium avenaceum***).

*Holotypus*: B 70 0100195.

*Type locality*: **Poland**, Karthaus, Nýdek.

*Type substrate*: Dead *Auchenorrhyncha* (cicada).

*Note*: Synonym *fide*Wollenweber & Reinking (1935).  

*spermogoniopsis Fusarium* Jul. Müll., Ber. Deutsch. Bot. Ges. 3: 394. 1885.

***Hymenella spermogoniopsis*** (Jul. Müll.) L. Lombard & Sand.-Den., ***comb. nov***. MycoBank MB 837721.

*Basionym*: *Fusarium spermogoniopsis* Jul. Müll., Ber. Deutsch. Bot. Ges. 3: 394. 1885.

*Synonym*: *Hymenula spermogoniopsis* (Jul. Müll.) Wollenw., Fusaria Autogr. Delin. 1: 483. 1916.

Syntypes: ?B 70 0100196, B 70 0100197 & B 700100198.

*Type locality*: **Germany**

*Type substrate*: Sporocarps of *Phragmidium subcorticium* (= *Phragmidium mucronatum*) and on the uredo- and teliospores of *Phragmidium rubi* (= *Phragmidium barclayi*).

*Note*s: Wollenweber (1916) provided a new combination for *F. spermogoniopsis* in the genus *Hymenula*. However, the generic name *Hymenella* (1822) predates the generic name *Hymenula* (1828) and therefore we provide a new combination in the latter genus.  

*sphaeriae Fusarium* Fuckel, Fungi Rhen. Exs., Fasc. 3, no. 212. 1863.

(See *Fusarium clematidis*)

*Lectotypus*: G00111017, designated in Gräfenhan *et al.* (2011).

*Lectotype locality*: **Germany**, Hessen, Reichartshausen near Oestrich-Winkel.

*Lectotype substrate*: Parasitic on *Leptosphaeria* (*Sphaeria*) *dioica*, on *Urtica dioica*.  

*sphaeriiforme Fusarium* Sacc. (as ‘*sphaeriaeforme*’), Syll. Fung. 10: 723. 1892.

*Replaced synonym*: *Fusarium celtidis* Pass., Atti Reale Accad. Lincei, Rendiconti Cl. Sci. Fis., sér. 4, 7: 51. 1891, *nom. illegit.*, Art. 53.1.

(See *Fusarium melanochlorum*)

*Holotypus*: ?PARMA.

*Type locality*: **Italy**, Parma, Vigheffio.

*Type substrate*: Dead branches of *Celtis australis*.

*Note*: Synonyms *fide*Wollenweber & Reinking (1935).  

*sphaeroideum Fusarium* Pass., Atti Reale Accad. Lincei, Rendiconti Cl. Sci. Fis., sér. 4, 4: 105. 1888.

(See ***Fusarium lateritium***)

*Holotypus*: ?PARMA.

*Type locality*: **Italy**, Parma.

*Type substrate*: Branch of *Ficus carica*.

*Note*: Synonym *fide*Wollenweber & Reinking (1935).  

*sphaerosporum Fusarium* Q.T. Chen & X.H. Fu, Acta Mycol. Sin., Suppl. 1: 331. 1987.

***Neocosmospora sphaerospora*** (Q.T. Chen & X.H. Fu) Sand.-Den. & Crous, Persoonia 43: 173. 2019.

*Holotypus*: HMAS 43749.

*Ex-type culture*: NF 5840.

*Type locality*: **China**, Guangdong Province, Maoming.

*Type substrate*: Water from underground pipes of oilfield.

*Descriptions and illustrations*: See Chen *et al.* (1987).

*spinaciae Fusarium* Hungerf., Phytopathology 13: 209. 1923.

(See ***Fusarium oxysporum***)

*Lectotypus* (*hic designatus*, MBT 10000744): **USA**, Idaho, roots of *Spinacia oleracea*, 1923, C.W. Hungerford, in Phytopathology 13: 208, fig. 4.

*Notes*: Synonym *fide*Booth (1971). No holotype specimen could be located and therefore an illustration was designated as lectotype.  

***spinosum Fusarium*** L. Lombard *et al.*, Fungal Syst. Evol. 4: 195. 2019.

*Holotypus*: CBS H-24020.

*Ex-type culture*: CBS 122438.

*Type locality*: **Brazil**.

*Type substrate*: Galia melon imported into the Netherlands.

*Descriptions and illustrations*: See Lombard *et al.* (2019a).

*Diagnostic DNA barcodes*: *rpb1*: MN120729; *rpb2*: MN120747; *tef1*: MN120768.  

*spinulosum Fusarium* (Pfenning) O'Donnell *et al.*, Index Fungorum 440: 4. 2020.

***Neocosmospora spinulosa*** Pfenning, Sydowia 47: 66. 1995.

*Holotypus*: CBS H-5452a.

*Ex-type culture*: CBS 321.93.

*Type locality*: **Brazil**, Pará, Capitão Poço.

*Type substrate*: Soil under *Theobroma cacao*.

*Descriptions and illustrations*: See Pfenning (1995).

*splendens Fusarium* Matuo & Takah. Kobay., Trans. Mycol. Soc. Japan 2(4): 13. 1960, *nom. inval.*, Art. 39.1.

(See *Fusarium matuoi*)

*Authentic material*: Not located.

*Original locality*: **Japan**.

*Original substrate*: Twigs of *Albizzia julibrissin*.

*Descriptions and illustrations*: See Matuo & Kobayashi (1960) and Hosoya & Tubaki (2004).  

***sporodochiale Fusarium*** L. Lombard & Crous, Fungal Syst. Evol. 4: 196. 2019.

*Holotypus*: CBS H-12681.

*Ex-type culture*: ATCC 14167 = CBS 220.61 = MUCL 8047 = NRRL 20842.

*Type locality*: **South Africa**, Gauteng Province, Johannesburg.

*Type substrate*: Soil.

*Descriptions and illustrations*: See Lombard *et al.* (2019a).

*Diagnostic DNA barcodes*: *rpb1*: MN120731; *rpb2*: MN120749; *tef1*: MN120770.  

*sporotrichiella Fusarium* Bilaĭ, Yadovitye griby na zerne khlebnykh zlakov: 86. 1953, *nom. inval.*, Art. 39.1.

(See ***Fusarium sporotrichioides***)

*Authentic material*: Not located.

*Original locality*: **Ukraine**.

*Original substrate*: Unknown.

*Descriptions and illustrations*: See Bilaĭ (1955).

*Notes*: This taxon was published as a new name for all the taxa in section *Sporotrichiella*. However, it is invalid as no type and Latin diagnosis were provided. Synonym *fide*Gerlach & Nirenberg (1982).  

***sporotrichioides Fusarium*** Sherb., Mem. Cornell Univ. Agric. Exp. Sta. 6: 183. 1915.

*Synonyms*: *Fusarium sporotrichiella* var. *sporotrichioides* (Sherb.) Bilaĭ, *Yadovitye griby na zerne khlebnykh zlakov (Poisonous fungi on cereal* see*d),* Kiev: 87. 1953, *nom*. *inval*., Art. 39.1.

*Fusarium sporotrichiella* Bilaĭ, *Yadovitye griby na zerne khlebnykh zlakov (Poisonous fungi on cereal* see*d)*, Kiev: 86. 1953, *nom*. *inval*., Art. 39.1.

*Fusarium sporotrichioides* var. *minus* Wollenw., Fusaria Autogr. Delin. 3: 886. 1930.

*Fusarium sporotrichioides* subsp. *minus* (Wollenw.) Raillo, Fungi of the Genus Fusarium: 196. 1950.

*Lectotypus* (*hic designatus*, MBT 10000745): **USA**, New York, rotten tubers of *Solanum tuberosum*, together with *F. solani* and *F. oxysporum*, 1915, C.D. Sherbakoff, in Mem. Cornell Univ. Agric. Exp. Sta. 6: 184, fig. 22.

*Notes*: This economically important species requires epitypification from the type locality and substrate. No holotype specimen could be located and therefore an illustration was designated as lectotype.  

*staphyleae Fusarium* Samuels & Rogerson, Brittonia 36: 84. 1984.

***Geejayessia atrofusca*** (Schwein.) Schroers & Gräfenhan, Stud. Mycol. 68: 126. 2011.

*Basionym*: *Sphaeria atrofusca* Schwein., Trans. Amer. Philos. Soc., n.s. 4: 206. 1832.

*Synonyms*: *Valsaria atrofusca* (Schwein.) Cooke ex Sacc., Syll. Fung. 9: 759. 1891.

*Nectria atrofusca* (Schwein.) Ellis & Everh., N. Amer. Pyren.: 99. 1892.

*Pseudodiplodia atrofusca* (Schwein.) Starbäck, Bih. Kongl. Svenska Vetensk.-Akad. Handl. 19: 94. 1894.

*Cucurbitaria atrofusca* (Schwein.) Kuntze, Revis. Gen. Pl. 3: 460. 1898.

*Creonectria atrofusca* (Schwein.) Seaver, Mycologia 1: 186. 1909.

*Holotypus*: In NY.

*Ex-type culture*: ATCC 66906 = CBS 502.94 = IMI 345891 = NRRL 22120.

*Type locality*: **USA**, Massachusetts, Berkshire, south of Ashley Falls, Bartholomew's Cobble.

*Type substrate*: Branches of *Staphylea trifolia*.

*Descriptions and illustrations*: See Samuels & Rogerson (1984) and Schroers *et al.* (2011).  

*stercicola Fusarium* Šišić *et al.*, Antonie van Leeuwenhoek 111: 1793. 2018.

***Neocosmospora stercicola*** (Šišić *et al.*) Sand.-Den. & Crous, Persoonia 43: 173. 2019.

*Synonyms*: *Fusarium martii* var. *viride* Sherb., Mem. Cornell Univ. Agric. Exp. Sta. 6: 247. 1915.

*Fusarium solani* var. *martii* ‘f. 1’ Wollenw., Z. Parasitenk. (Berlin) 3: 290. 1931.

*Fusarium witzenhausenense* Šišić *et al.*, Antonie van Leeuwenhoek 111: 1795. 2018.

*Fusarium xiangyunense* F. Zhang *et al.* (as ‘*xiangyunensis*’), Phytotaxa 450: 278. 2020. *nom. inval.*, Art. 40.8.

*Holotypus*: CBS H-23352.

*Ex-type culture*: CBS 142481 = DSM 106211 = FS 89.

*Type locality*: **Germany**, Niedersachsen, Hannover.

*Type substrate*: Compost yard waste plant debris.

*Descriptions and illustrations*: See Šišić *et al.* (2018a).

*Diagnostic DNA barcodes*: *rpb1*: MW834255; *rpb2*: LR583887; *tef1*: LR583658.  

*stercorarium Fusarium* Rostr., Meddel. Grønland 18: 74. 1894.

*Holotypus*: C-F-92401

*Type locality*: **Greenland**, Vestfjord.

*Type substrate*: Dung of *Rangifer tarandus* (reindeer).

*Notes*: Status unclear. Not *Fusarium fide*Wollenweber & Reinking (1935).  

*stercoris Fusarium* Fuckel, Fungi Rhen. Exs., Suppl., Fasc. 5: no. 1921. 1867 [and Jahrb. Nassauischen Vereins Naturk. 23–24: 369. 1870].

(See ***Fusarium avenaceum***)

*Lectotypus* (*hic designatus*, MBT 10000746): **Germany**, Hessen, Oestrich-Winkel, soil next to *Peziza stercoraria*, date unknown, K.W.G.L. Fuckel, Fungi Rhen. Exs., Suppl., Fasc. 5: no. 1921 in HAL.

*Notes*: Synonym *fide*Wollenweber & Reinking (1935). No holotype specimen could be located and therefore the exsiccate lodged in HAL is designated as lectotype.  

***sterilihyphosum Fusarium*** Britz *et al.*, Mycologia 94: 726. 2002.

*Holotypus*: PREM 57302.

*Ex-type culture*: NRRL 25623.

*Type locality*: **South Africa**, Limpopo Province, Tzaneen, Letsitele area.

*Type substrate*: Malformed inflorescence of *Mangifera indica*.

*Descriptions and illustrations*: See Britz *et al.* (2002) and Leslie & Summerell (2006).

*Diagnostic DNA barcodes*: *rpb1*: MN193925; *rpb2*: MN193897; *tef1*: MN193869.  

*sticticum Fusarium* Berk. & M.A. Curtis, in Berkeley, Grevillea 3: 99. 1875.

(See ***Fusarium lateritium***)

*Holotypus*: ?K(M).

*Type locality*: **USA**, South Carolina.

*Type substrate*: Twigs of *Prunus persica*.

*Note*: Synonym *fide*Wollenweber & Reinking (1935).  

*stictoides Fusarium* Durieu & Mont., Explor. Sci. Algérie 1: 334. 1848.

(See ***Fusarium graminearum***)

*Holotypus*: ?PC.

*Type locality*: **Algeria**.

*Type substrate*: Branch of flowering *Agave* sp.

*Note*: Synonym *fide*Wollenweber & Reinking (1935).  

*stilbaster Fusarium* (Link) Link, Sp. pl., Ed. 4, 6: 106. 1825.

***Atractium stilbaster*** Link, Mag. Ges. Naturf. Freunde, Berlin 3: 10. 1809.

*Synonyms*: *Atractium fuscum* Sacc., Syll. Fung. 2: 514. 1883.

*Stilbella fusca* (Sacc.) Seifert, Stud. Mycol. 27: 77. 1985.

*Atractium flavoviride* Sacc., Syll. Fung. 2: 514. 1883.

*Stilbum madidum* Peck, Rep. (Annual) New York State Mus. Nat. Hist. 46: 115. 1894.

*Didymostilbe eichleriana* Bres. & Sacc., Atti Congr. Bot. Palermo: 59. 1903.

*Didymostilbe obovoidea* Matsush., Icon. Microfung. Matsush. lect.: 60. 1975.

*Lectotypus*: Illustration published in Mag. Ges. Naturf. Freunde, Berlin 3, tab. I, fig. 11, designated in Gräfenhan *et al.* (2011).

*Epitypus*: CBS 410.67 (preserved as metabolically inactive culture), designated in Gräfenhan *et al.* (2011).

*Ex-epitype culture*: CBS 410.67.

*Epitype locality*: **Germany**, Bayerischer Wald, Rachelseewand.

*Epitype substrate*: Bark.

*Descriptions and illustrations*: See Seifert (1985) and Gräfenhan *et al.* (2011).

*Diagnostic DNA barcodes*: *rpb1*: KM232206; *tef1*: KM231920.  

***stilboides Fusarium*** Wollenw., Fusaria Autogr. Delin. 2: 615. 1924.

*Synonyms*: *Fusarium lateritium* var. *stilboides* (Wollenw.) Bilaĭ, Fusarii (Biologija i sistematika): 266. 1955, *nom. inval*., Art. 41.5.

*Fusarium lateritium* var. *stilboides* (Wollenw.) Bilaĭ, Mikrobiol. Zhurn. 49: 6. 1987.

*Fusarium lateritium* var. *longum* Wollenw., Fusaria Autogr. Delin. 1: 385. 1916.

*Fusarium fructigenum* var. *minus* Wollenw., Z. Parasitenk. (Berlin) 3: 386. 1931.

*Fusarium stilboides* var. *minus* (Wollenw.) Wollenw., Z. Parasitenk. (Berlin) 3: 333. 1931.

*Fusarium stilboides* ‘f. 1’ Raillo, Fungi of the Genus Fusarium: 271. 1950.

*Gibberella stilboides* W.L. Gordon ex C. Booth, The Genus Fusarium: 119. 1971.

*Lectotypus* (*hic designatus*, MBT 10000747): **Philippines**, Los Baños, living twigs of *Citrus* sp., invaded by coccids, 1917, O.A. Reinking, in Fusaria Autogr. Delin. 2: 615.

*Epitypus* (*hic designatus*, MBT 10000748): **Cook Islands**, *Citrus* sp., Sep. 1978, G.F. Laundon, CBS 746.79 (preserved as metabolically inactive culture).

*Ex-epitype culture*: BBA 63887 = CBS 746.79 = ICMP 10624 = NRRL 25485.

*Descriptions and illustrations*: See Wollenweber (1924, 1930), Wollenweber & Reinking (1935), Doidge (1938), Raillo (1950), Booth (1971), and Gerlach & Nirenberg (1982).

*Diagnostic DNA barcodes*: *rpb1*: MW928817; *rpb2*: MW928832; *tef1*: MW928843.

*Note*: No holotype specimen could be located and therefore an illustration was designated as lectotype.  

*stillatum Fusarium* De Not. ex Sacc., in Berlese & Voglino, Syll. Fung., Addit. I–IV: 390. 1886.

***Myxosporium stillatum*** (De Not. ex Sacc.) Wollenw., Fusaria Autogr. Delin. 1: 490. 1916.

*Lectotypus* (*hic designatus*, MBT 10000749): **Italy**, Valle Intrasca, at the bridge on Possaccio, dried stems of *Genista tinctoria*, 1862, G. de Notaris, S-F45664 [Baglietto, Cesati & Notaris, Erb. Critt. Ital. Ser. I no. 148 (1148)].

*Notes*: Synonym *fide*Wollenweber & Reinking (1935). No holotype specimen could be located and therefore the exsiccate lodged in S is designated as lectotype.  

*stoveri Fusarium* C. Booth, The Genus Fusarium: 37. 1971.

***Microdochium stoveri*** (C. Booth) Samuels & I.C. Hallett, Trans. Brit. Mycol. Soc. 81: 481. 1983.

*Basionym*: *Micronectriella stoveri* C. Booth, Mycol. Pap. 94: 3. 1964.

*Synonym*: *Monographella stoveri* (C. Booth) Samuels & I.C. Hallett, Trans. Brit. Mycol. Soc. 81: 473. 1983.

*Holotypus*: IMI 92905.

*Type locality*: **Honduras**.

*Type substrate*: Leaf of *Musa* sp.

*Descriptions and illustrations*: See Booth (1964, 1971), Gerlach & Nirenberg (1982) and Samuels & Hallet (1983).  

*striatum Fusarium* Sherb., Mem. Cornell Univ. Agric. Exp. Sta. 6: 255. 1915.

(See *Fusarium solani-melongenae*)

*Typus*: ?CUP-007460.

*Type locality*: **USA**, Colorado

*Type substrate*: *Solanum tuberosum.*

*Notes*: Synonym *fide*Nirenberg & Brielmaiers-Liebetanz, 1996 and Sandoval-Denis *et al.* (2019). Lectotypification pending study of material lodged in CUP.  

*strobilinum Fusarium* Corda, Icon. Fung. 1: 4. 1837.

***Sirococcus conigenus*** (Pers.) P.F. Cannon & Minter, Taxon 32: 577. 1983.

*Basionym*: *Hysterium conigenum* Pers., Ann. Bot. (Usteri) 15: 30. 1795.

*Synonyms*: *Hypoderma conigenum* (Pers.) DC., Fl. Franç., ed. 3, 2: 305. 1805.

*Hypodermopsis conigena* (Pers.) Kuntze, Revis. Gen. Pl. 3: 487. 1898.

*Discella conigena* (Pers.) Höhn., Mitt. Bot. Inst. T. H. Wien 6: 120. 1929.

*Ascochyta strobilina* (Corda) Wollenw., Fusaria Autogr. Delin. 1: 505. 1916.

*Sphaeria strobilina* Holl & J.C. Schmidt, Deutschl. Schwämme, Erste Lieferung: 4. 1815, *nom. inval*., Art. 38.1(a).

*Sphaeria strobilina* Holle & J.C. Schmidt ex Fr., Syst. Mycol. 2: 495. 1823.

*Dichaena strobilina* (Holle & J.C. Schmidt ex Fr.) Fr., Summa Veg. Scand. 2: 403. 1849.

*Sporonema strobilinum* Desm., Ann. Sci. Nat., Bot., sér. 3, 18: 368. 1852.

*Plenodomus strobilinus* (Desm.) Höhn., Sitzungsber. Kaiserl. Akad. Wiss. Wien, Math.-Naturwiss. Cl., Abt. 1, 119: 647. 1910.

*Discella strobilina* (Desm.) Died., Krypt.-Fl. Brandenburg 9: 752. 1914.

*Sirococcus strobilinus* (Desm.) Petr., Sydowia 1: 155. 1947, *nom. illegit.*, Art. 53.1.

*Sirococcus strobilinus* Preuss, Linnaea 26: 716. 1855.

*Phoma conigena* P. Karst., Rev. Mycol. (Toulouse) 7: 106. 1885.

*Septoria parasitica* R. Hartig, Z. Forst- Jagdwesen 1890: 1. 1890.

*Diplodina parasitica* (R. Hartig) Prill., Maladies des Plantes Agricoles 2: fig. 365. 1897.

*Ascochyta parasitica* Fautrey, Rev. Mycol. (Toulouse) 13: 79. 1891.

*Ascochyta piniperda* Lindau, Nat. Pflanzenfam., Teil. I, 1: 367. 1900.

*Phoma conigena* var. *abieticola* Sacc., Ann. Mycol. 3: 233. 1905.

*Typus*: In PRM *fide*Pilat (1938).

*Type locality*: **Czech Republic**, near Liberec (Reichenberg).

*Type substrate*: Rotten cone scales of *Pinus* sp.

*Note*: Typification pending study of material lodged in PRM.  

*stromaticola Fusarium* Henn., Bot. Jahrb. Syst. 28: 280. 1900.

***Dialonectria volutella*** (Ellis & Everh.) L. Lombard & Sand.-Den., ***comb. nov*.** MycoBank MB 837722.

*Basionym*: *Fusarium volutella* Ellis & Everh., Proc. Acad. Nat. Sci. Philadelphia 43: 93. 1891.

*Synonyms*: *Fusarium aquaeductuum* var. *medium* Wollenw., Fusaria Autogr. Delin. 3: 844. 1930.

*Fusarium aquaeductuum* subsp. *medium* (Wollenw.) Raillo, Fungi of the Genus Fusarium: 278. 1950.

*Dialonectria ullevolea* Seifert & Gräfenhan, Stud. Mycol. 68: 97. 2011.

*Holotypus*: In B *fide*Hein (1988).

*Type locality*: **Japan**, Tokyo.

*Type substrate*: Old stroma of *Dothideaceae*, on *Bambusa* sp. branches with *Zythia stromaticola*.

*Note*s: Synonym *fide*Wollenweber & Reinking (1935) and Gräfenhan *et al.* (2011). The older epithet ‘*volutella*’ (1891) supersedes the epithet ‘*ullevolea*’ (2011) and, therefore, a new combination is provided.  

*stromaticum Fusarium* Delacr., Bull. Soc. Mycol. France 9: 186. 1893.

(See ***Fusarium heterosporum***)

*Holotypus*: ?PC.

*Type locality*: **France**, overseas department of Mayotte, Mayotte islands.

*Type substrate*: Seeds of unknown *Poaceae* (= *Gramineae*).

*Note*: Synonym *fide*Wollenweber & Reinking (1935).  

*subcarneum Fusarium* P. Crouan & H. Crouan, Fl. Finistère: 14. 1867, *nom. rej*.

(See ***Fusarium sambucinum***)

*Authentic material*: ?PC.

*Original locality*: **France**, Brittany, Finistère, marshes.

*Original substrate*: Twigs and dead leaves of *Ulex* sp.

*Note*: Synonym *fide*Wollenweber & Reinking (1935).  

*subcorticale Fusarium* Oudem., Ned. Kruidk. Arch., sér. 3, 3: 135. 1898.

(See *Fusarium buxicola*)

*Holotypus*: ?L.

*Type locality*: **Netherlands**, Zuid-Holland Province, Zorgvliet.

*Type substrate*: *Buxus sempervirens*.

*Note*: Synonym *fide*Wollenweber & Reinking (1935).  

***subglutinans Fusarium*** (Wollenw. & Reinking) P.E. Nelson *et al.*, *Fusarium* species. An illustrated manual for identification: 135. 1983.

*Basionym*: *Fusarium moniliforme* var. *subglutinans* Wollenw. & Reinking, Phytopathology 15: 163. 1925.

*Synonyms*: *Fusarium moniliforme f. subglutinans* (Wollenw. & Reinking) C. Moreau, Rev. Mycol. (Paris) 17: 23. 1952.

*Fusarium sacchari* var. *subglutinans* (Wollenw. & Reinking) Nirenberg, Mitt. Biol. Bundesanst. Land- Forstw. 169: 53. 1976.

*Gibberella fujikuroi* var. *subglutinans* (Wollenw. & Reinking) E.T. Edwards, Agric. Gaz. New South Wales 44: 895. 1933 (Art. F.8.1, *Note* 2, Exs. 2).

*Gibberella subglutinans* (Wollenw. & Reinking) P.E. Nelson *et al.*, Fusarium species. An illustrated manual for identification (University Park): 135. 1983.

*Neotypus*: CBS 747.97 (preserved as metabolically inactive culture), designated by Yilmaz *et al.* (2021).

*Ex-neotype culture*: BBA 62451 = CBS 747.97 = DAOM 225141 = FRC M-36 = MRC 8554 = NRRL 22016 = NRRL 22114.

*Neotype locality*: **USA**, Illinois, Saint Elmo.

*Neotype substrate*: *Zea mays*.

*Descriptions and illustrations*: See Booth (1971), Nirenberg (1976, 1981), Nelson *et al.* (1983), Pascoe (1990), Leslie & Summerell (2006).

*Diagnostic DNA barcodes*: *rpb1*: JX171486; *rpb2*: JX171599; *tef1*: HM057336.  

***sublunatum Fusarium*** Reinking, Zentralbl. Bakteriol., 2. Abt. 89: 510. 1934.

*Synonyms*: *Fusarium elongatum* Reinking, Zentralbl. Bakteriol. Parasitenk., Abt. 2, 89: 511. 1934, *nom. illegit.*, Art. 53.1.

*Fusarium sambucinum* var. *sublunatum* (Reinking) Bilaĭ, Mikrobiol. Zhurn. 49: 6. 1987, *nom. inval*., Art. 41.4, *Note* 1.

*Lectotypus* (*hic designatus*, MBT 10000750): **Costa Rica**, Limon, soil in *Musa sapientum* plantation, 1933, O.A. Reinking, CBS 189.34 (preserved as metabolically inactive culture).

*Ex-type culture*: BBA 62431 = CBS 189.34 = DSM 62431 = NRRL 13384 = NRRL 20840.

*Descriptions and illustrations*: See Gerlach & Nirenberg (1982).

*Diagnostic DNA barcodes*: *rpb1*: JX171451; *rpb2*: KX302935; *tef1*: KX302919.

*Notes*: No holotype specimen could be located for *F. sublunatum* and therefore the metabolically inactive culture CBS 189.34 (= IMB 5238), which represents the ex-type culture (Gerlach & Nirenberg 1982), is designated as lectotype.  

*subnivale Fusarium* Höhn., in Penther & Zederbauer, Ann. K. K. Naturhist. Hofmus. 20: 369. 1905.

(See *Fusarium dimerum*)

*Holotypus*: FH00965354.

*Type locality*: **Turkey**, Anatolia.

*Type substrate*: Stems and leaves of decayed *Astragalus* sp.

*Note*: Synonym *fide*Wollenweber & Reinking (1935).  

*subpallidum Fusarium* Sherb., Mem. Cornell Univ. Agric. Exp. Sta. Mem. 6: 230. 1915.

(See ***Fusarium sambucinum***)

Typus: ?CUP-007480.

*Type locality*: **USA**, Louisiana, Edgerton.

*Type substrate*: *Solanum tuberosum.*

*Notes*: Synonym *fide*Wollenweber & Reinking (1935). Lectotypification pending study of material lodged in CUP.  

*subtectum Fusarium* Roberge ex Desm., Pl. Crypt. N. France, ed. 1, Fasc. 29, no. 1428. 1845.

***Rhodesia subtecta*** (Roberge ex Desm.) Grove, British Stem- and Leaf-Fungi (Coelomycetes) 2: 205. 1937.

*Synonyms*: *Myxosporina subtecta* (Roberge ex Desm.) Höhn., in Weese, Ber. Deutsch. Bot. Ges. 37: 155. 1919, *nom. inval*., Art. 35.1.

*Myxosporina subtecta* (Roberge ex Desm.) Höhn., Mitt. Bot. Inst. Tech. Hochsch. Wien 4: 74. 1927.

*Hainesia subtecta* (Roberge ex Desm.) Grove, J. Bot. 70: 4. 1932.

*Hymenula psammae* Oudem., Ned. Kruidk. Arch., sér. 3, 1: 533. 1898. (*fide* Wollenweber & Reinking 1935).

*Syntypes*: Pl. Crypt. N. France no. 1428 in ?BRU, PC & PH.

*Type locality*: **France**.

*Type substrate*: Dead leaves of *Arundo arenaria*.  

***subtropicale Fusarium*** C. Pereira *et al.*, Mycologia 110: 864. 2018.

*Holotypus*: BPI 910644.

*Ex-type culture*: CBS 144706 = NRRL 66764.

*Type locality*: **Brazil**, Paraná State, Guarapuava.

*Type substrate*: *Hordeum vulgare*.

*Descriptions and illustrations*: See Pereira *et al.* (2018).

*Diagnostic DNA barcodes*: *rpb1*: MH706972; *rpb2*: MH706973; *tef1*: MH706974.  

*subulatum Fusarium* Appel & Wollenw., Arbeiten Kaiserl. Biol. Anst. Land- Forstw. 8: 131. 1913.

*Replaced synonym*: *Fusarium roseum* var. *lupini-albi* Sacc., Michelia 2: 295. 1881.

(See ***Fusarium avenaceum***)

*Holotypus*: Not located.

*Type locality*: **Italy**, Selva.

*Type substrate*: *Lupinus albus*.

*Note*: Synonyms *fide*Wollenweber & Reinking (1935).  

*subviolaceum Fusarium* Roum. & Fautrey, Fungi Sel. Gall. Exs. no. 6022. 1892.

(See ***Fusarium avenaceum***)

*Syntype*: ILL0020193 (Fungi Sel. Gall. Exs. no. 6022).

*Type locality*: **France**, Jardin de Noidan.

*Type substrate*: Dry stems of *Asparagus officinalis.*

*Note*: Synonym *fide*Wollenweber & Reinking (1935).  

***succisae Fusarium*** Schröt. ex Sacc., Syll. Fung. 10: 724. 1892.

*Synonym*: *Fusisporium succisae* J. Schröt., Hedwigia 13: 180. 1874, *nom. inval*., Art. 36.1(a).

*Lectotypus*: ILL00076313 (Thümen, Mycoth. Univ. no. 675), designated by Yilmaz *et al.* (2021).

*Lectotype locality*: **Germany**, Bavaria, Borussia.

*Lectotype substrate*: *Succisa pratensis.*

*Epitypus*: IMI 202876, designated by Yilmaz *et al.* (2021).

*Ex-epitype culture*: BBA 12287 = BBA 63627 = CBS 219.76 = DAOM 225142 = IMI 202876 = IMI 375347 = NRRL 13613.

*Epitype locality*: **Germany**.

*Epitype substrate*: *Succisa pratensis.*

*Descriptions and illustrations*: See Nirenberg (1976), Gerlach & Nirenberg (1982).

*Diagnostic DNA barcodes*: *rpb1*: LT996207; *rpb2*: LT970764.  

***sudanense Fusarium*** S.A. Ahmed *et al.*, Antonie van Leeuwenhoek 110: 826. 2017.

*Holotypus*: CBS H-22547.

*Ex-type culture*: CBS 454.97.

*Type locality*: **Sudan**.

*Type substrate*: Plant debris of *Striga hermonthica*.

*Descriptions and illustrations*: See Moussa *et al.* (2017).

*Diagnostic DNA barcodes*: *rpb1*: LT996208; *rpb2*: LT996155; *tef1*: KU711697.  

***sulawesiense Fusarium*** Maryani *et al.* (as ‘*sulawense*’), Persoonia 43: 65. 2019.

*Holotypus*: InaCC F940 (preserved as metabolically inactive culture).

*Ex-type culture*: InaCC F940.

*Type locality*: **Indonesia**, South Sulawesi, Bone, Kecamatan Bengo, Desa Selli.

*Type substrate*: Infected pseudostem of *Musa acuminata* var. Pisang Cere (AAA).

*Descriptions and illustrations*: See Maryani *et al.* (2019b).

*Diagnostic DNA barcodes*: *rpb2*: LS479855; *tef1*: LS479443.  

*sulphureum Fusarium* Schltdl., Fl. Berol. 2: 139. 1824, *nom. rej*.

(See ***Fusarium sambucinum***)

*Holotypus*: HAL 1613 F.

*Type locality*: **Germany**, Berlin.

*Type substrate*: Rotting tuber of *Solanum tuberosum*.

*Note*: Synonym *fide*Wollenweber & Reinking (1935).  

*suttonianum Fusarium* (Sand.-Den. & Crous) O'Donnell *et al.*, Index Fungorum 440: 4. 2020.

***Neocosmospora suttoniana*** Sand.-Den. & Crous, Persoonia 41: 123. 2018.

*Holotypus*: CBS H-23224.

*Ex-type culture*: CBS 143214 = FRC S-1423 = NRRL 32858.

*Type locality*: **USA**, Louisiana.

*Type substrate*: *Homo sapiens*.

*Descriptions and illustrations*: See Sandoval-Denis & Crous (2018).

*Diagnostic DNA barcodes*: *rpb1*: MW218138; *rpb2*: EU329630; *tef1*: DQ247163.  

*tabacinum Fusarium* (J.F.H. Beyma) W. Gams, Persoonia 5: 179. 1968.

*Basionym*: *Cephalosporium tabacinum* J.F.H. Beyma, Zentralbl. Bakteriol. 2. Abt. 89: 240. 1933.

***Plectosphaerella cucumerina*** (Lindf.) W. Gams, in Domsch & Gams, *Fungi in Agricultural Soils*: 160. 1972.

*Basionym*: *Venturia cucumerina* Lindf., Meddn Centralanst. Försksv. Jordbruksomr. Bot. Avd. 17: 7. 1919.

*Synonyms*: *Monographella cucumerina* (Lindf.) Arx, Trans. Brit. Mycol. Soc. 83: 374. 1984.

*Microdochium tabacinum* (J.F.H. Beyma) Arx, Trans. Brit. Mycol. Soc. 83: 374. 1984.

*Plectosporium tabacinum* (J.F.H. Beyma) M.E. Palm, W. Gams & Nirenberg, Mycologia 87: 399. 1995.

*Plectosphaerella cucumeris* Kleb., Phytopathol. Z. 1: 43. 1929.

*Micronectriella cucumeris* (Kleb.) C. Booth, The Genus Fusarium: 39. 1971.

*Cephalosporium ciferrii* Verona, *Studio sulle cause microbiche che danneggiano la carta ed i libri*: 30. 1939.

*Cephalosporiopsis imperfecta* M. Moreau & Moreau, Rev. Mycol. (Paris) 6: 67. 1941, *nom. inval*., Art. 39.1.

*Neotypus*: CBS H-7656, designated in Palm *et al.* (1995).

*Ex-neotype culture*: CBS 137.33 = MUCL 9701 = NRRL 22455*.*

*Neotype locality*: **UK**, England, Bristol.

*Neotype substrate*: Stems of *Nicotiana tabacum*.

*Descriptions and illustrations*: See Domsch *et al.* (2007), Carlucci *et al.* (2012), Giraldo & Crous (2019)*.*  

*tabacivorum Fusarium* Delacr., Ann. Inst. Natl. Agron., ser. 2, 5: 207. 1906.

(See ***Fusarium oxysporum***)

*Holotypus*: ?PC.

*Type locality*: **France**, Périgueux, Razac-sur-l'Isle*.*

*Type substrate*: *Nicotiana tabacum.*

*Note*: Synonym *fide*Wollenweber & Reinking (1935).  

***tanahbumbuense Fusarium*** Maryani *et al.*, Persoonia 43: 63. 2019.

*Holotypus*: InaCC F965 (preserved as metabolically inactive culture).

*Ex-type culture*: InaCC F965.

*Type locality*: **Indonesia**, South Kalimantan, Tanah Bumbu, Kecamatan Kusan Hilir, Desa Betung.

*Type substrate*: Pseudostem of *Musa* var. Pisang Hawa.

*Descriptions and illustrations*: See Maryani *et al.* (2019b).

*Diagnostic DNA barcodes*: *rpb1*: LS479877; *rpb2*: LS479863; *tef1*: LS479448.  

***tardichlamydosporum Fusarium*** Maryani *et al.*, Stud. Mycol. 92: 181. 2018 [2019].

*Holotypus*: InaCC F958 (preserved as metabolically inactive culture).

*Ex-type culture*: InaCC F958.

*Type locality*: **Indonesia**, East Nusa Tenggara, Sikka Flores, Desa Kota Uneng Kecamatan Alok.

*Type substrate*: Pseudostem of *Musa acuminata* var. Pisang Barangan.

*Descriptions and illustrations*: See Maryani *et al.* (2019a).

*Diagnostic DNA barcodes*: *rpb1*: LS479534; *rpb2*: LS479280; *tef1*: LS479729.  

***tardicrescens Fusarium*** Maryani *et al.*, Persoonia 43: 69. 2019.

*Synonym*: *Fusarium tardicrescens* Maryani *et al.*, Stud. Mycol. 92: 185. 2018 [2019], *nom. inval*., Art. 40.7.

*Holotypus*: CBS 102024 (preserved as metabolically inactive culture).

*Ex-type culture*: CBS 102024 = NRRL 36113.

*Type locality*: **Malawi**, Karonga, Misuku Hills.

*Type substrate*: *Musa sapientum* cv. Harare.

*Descriptions and illustrations*: See Maryani *et al.* (2019b).

*Diagnostic DNA barcodes*: *rpb1*: LS479474; *rpb2*: LS479217; *tef1*: LS479665.  

*tasmaniense Fusarium* (McAlpine) Rossman (as ‘*tasmanica*’), Mycol. Pap. 150: 54. 1983.

*Basionym*: *Microcera tasmaniensis* McAlpine, J. Dept. Agric. Victoria 2: 647. 1904.

*Synonyms*: *Discofusarium tasmaniense* (McAlpine) Petch, Trans. Brit. Mycol. Soc. 7: 143, 165. 1921.

*Microcera myrtilaspis* McAlpine, J. Dept. Agric. Victoria 2: 647. 1904.

*Calonectria coccidophaga* Petch, Trans. Brit. Mycol. Soc. 7: 161. 1921.

*Nectria coccidophaga* (Petch) Rossman, Mycotaxon 8: 499. 1979.

*Holotypus*: VPRI 2744.

*Type locality*: **Australia**, Tasmania.

*Type substrate*: Parasitic on *Aspidiotus* sp. (scale) on *Eucalyptus* bark.

*Descriptions and illustrations*: See Rossman (1983).

*Notes*: Status unclear. Rossman (1983) studied the specimen in K(M) and recombined the asexual morph name in *Fusarium*, which is not supported by the features of the sexual-morph. This species most likely belongs to *Microcera* as originally specified by McAlpine (1904).  

***temperatum Fusarium*** Scaufl. & Munaut, Mycologia 103: 593. 2011.

*Holotypus*: MUCL 52463-H.

*Ex-type culture*: MUCL 52463.

*Type locality*: **Belgium**, Waals-Brabant Province, Chastre.

*Type substrate*: *Zea mays*.

*Descriptions and illustrations*: See Scauflaire *et al.* (2011).

*Diagnostic DNA barcode*: *tef1*: KM487197.  

*tenellum Fusarium* Sacc. & Briard, Rev. Mycol. (Toulouse) 7: 212. 1885.

(See ***Fusarium sambucinum***)

*Holotypus*: Not located.

*Type locality*: **France**, Troyes.

*Type substrate*: Rotten stem of *Brassica oleracea*.

*Note*: Synonym *fide*Wollenweber & Reinking (1935).  

*tenue Fusarium* Corda, Icon. Fung. 1: 3. 1837.

(See ***Fusarium avenaceum***)

*Typus*: In PRM *fide*Pilat (1938).

*Type locality*: **Czech Republic**, near Prague.

*Type substrate*: Rotting stem of an unidentified host.

*Notes*: Synonym *fide*Wollenweber & Reinking (1935). Lectotypification pending study of material lodged in PRM.  

*tenuicristatum Fusarium* (S. Ueda & Udagawa) O'Donnell *et al.*, Index Fungorum 440: 4. 2020.

*Basionym*: *Neocosmospora tenuicristata* S. Ueda & Udagawa, Mycotaxon 16: 387. 1983.

*Synonym*: *Acremonium tenuicristatum* S. Ueda & Udagawa, Mycotaxon 16: 387. 1983.

*Holotypus*: NHL 2911.

*Type locality*: **Japan**, Nagasaki.

*Type substrate*: Marine sludge.

*Descriptions and illustrations*: See Ueda & Udagawa (1983).

*Notes*: Status unclear. See Sandoval-Denis *et al.* (2019).  

*tenuissimum Fusarium* (Peck) Sacc., Syll. Fung. 4: 711. 1886.

*Basionym*: *Fusisporium tenuissimum* Peck, Rep. (Annual) New York State Mus. Nat. Hist. 34: 48. 1883. 1881.

(See ***Fusarium sambucinum***)

*Holotypus*: NYSf3163.

*Type locality*: **USA**, New York, Schenectady.

*Type substrate*: Dead stem of unidentified host.

*Note*: Synonyms *fide*Wollenweber & Reinking (1935).  

*tenuistipes Fusarium* Sacc., Atti Mem. Reale Accad. Sci. Lett. Arti, Padova 33: 195. 1917.

(See ***Fusarium incarnatum***)

*Holotypus*: In PAD.

*Type locality*: **Unknown**.

*Type substrate*: *Pennisetum spicatum*.

*Note*: Synonym *fide*Wollenweber & Reinking (1935).  

*terrestre Fusarium* Manns, Bull. North Dakota Agric. Exp. Sta.: no. 259. 1932.

(See ***Fusarium equiseti***)

*Holotypus*: Not located.

*Type locality*: **USA**, North Dakota.

*Type substrate*: Soil.

*Note*: Synonym *fide*Wollenweber & Reinking (1935).  

***terricola Fusarium*** Al-Hatmi *et al.*, Antonie van Leeuwenhoek 110: 826. 2017.

*Holotypus*: CBS H-22548.

*Ex-type culture*: CBS 483.94.

*Type locality*: **Australia**, Queensland.

*Type substrate*: Desert soil.

*Descriptions and illustrations*: See Moussa *et al.* (2017).

*Diagnostic DNA barcodes*: *rpb1*: LT996209; *rpb2*: LT996156; *tef1*: KU711698.  

***thapsinum Fusarium*** Klittich *et al.*, Mycologia 89: 644. 1997.

*Synonym*: *Gibberella thapsina* Klittich *et al.*, Mycologia 89: 643. 1997.

*Holotypus*: BPI 737885.

*Ex-type culture*: ATCC 200522 = CBS 777.96 = FRC M-6564.

*Type locality*: **USA**, Kansas.

*Type substrate*: Stalk of *Sorghum* sp.

*Descriptions and illustrations*: See Klittich *et al.* (1997).

*Diagnostic DNA barcodes*: *rpb1*: MW928818; *rpb2*: MW928833; *tef1*: MW928844.  

*theobromae Fusarium* Appel & Strunk, Centralbl. Bacteriol., 2. Abth., 11: 635. 1904.

***Neocosmospora theobromae*** (Appel & Strunk) Sand.-Den. & Crous, Persoonia 43: 174. 2019.

*Synonyms*: *Fusarium javanicum* var. *theobromae* (Appel & Strunk) Wollenw., Z. Parasitenk. (Berlin) 3: 483. 1931.

*Neotypus*: BPI 453072, designated in Sandoval-Denis *et al.* (2019).

*Type locality*: **Cameroon**, Victoria.

*Type substrate*: Fruits and seeds of *Theobroma cacao*.

*Descriptions and illustrations*: See Sandoval-Denis *et al.* (2019).

*Diagnostic DNA barcode*: *tef1*: LR583660.

*Notes*: This *Fusarium* name was recently resurrected, neotypified, and transferred to *Neocosmospora* by Sandoval-Denis *et al.* (2019). DNA barcodes were generated from the neotype specimen; however, fresh collections are needed for epitypification.  

*theobromae Fusarium* M.L. Lutz, Bull. Soc. Bot. France 53: L. 1907 [1906], *nom. illegit.*, Art. 53.1.

***Diplocladium theobromae*** Sacc. & Trotter, Syll. Fung. 22: 1309. 1913.

*Authentic material*: Not located.

*Original locality*: **Democratic Republic of São Tomé and Príncipe**.

*Original substrate*: Fermented beans of *Theobroma cacao*.

*Note*: Originally erroneously assigned to the genus *Fusarium*.  

*thevetiae Fusarium* Tassi, Atti Reale Accad. Fisiocrit. Siena, sér. 4, 8: 238. 1897.

*Holotypus*: ?SIENA.

*Type locality*: **India**.

*Type substrate*: *Thevetia venenifera*.

*Notes*: Status unclear. A doubtful species *fide*Wollenweber & Reinking (1935).  

*thuemenii Fusarium* Sacc., Syll. Fung. 4: 722. 1886.

*Replaced synonym*: *Fusarium parasiticum* Thüm., Nuovo Giorn. Bot. Ital. 12: 198. 1880, *nom. illegit.*, Art. 53.1.

(See ***Fusarium oxysporum***)

*Holotypus*: Not located.

*Type locality*: **Russia**, Orenburg.

*Type substrate*: Rotten branches of *Betula verrucosa* (= *Betula pendula*).

*Note*: Synonym *fide*Wollenweber & Reinking (1935).  

***tjaetaba Fusarium*** T.T.H. Vu *et al.*, Fungal Diversity 77: 361. 2015 [2016].

*Holotypus*: RBG 5361.

*Ex-type culture*: FRL14350 = NRRL 66243 = RBG 5361.

*Type locality*: **Australia**, Northern Territory, Litchfield National Park.

*Type substrate*: *Sorghum interjectum*.

*Descriptions and illustrations*: See Laurence *et al.* (2016).

*Diagnostic DNA barcodes*: *rpb1*: KP083267; *rpb2*: KP083275; *tef1*: KP083263.  

***tjaynera Fusarium*** J.L. Walsh *et al.*, Fungal Diversity 77: 361. 2015 [2016].

*Holotypus*: RBG 5367.

*Ex-type culture*: NRRL 66246 = RBG 5367.

*Type locality*: **Australia**, Northern Territory, Litchfield National Park.

*Type substrate*: *Triodia microstachya*.

*Descriptions and illustrations*: See Laurence *et al.* (2016).

*Diagnostic DNA barcodes*: *rpb1*: KP083268; *rpb2*: KP083279; *tef1*: EF107152.  

*tomentosum Fusarium* Berk. & M.A. Curtis, J. Linn. Soc., Bot. 10: 359. 1868 [1869].

*Holotypus*: In K(M).

*Type locality*: **Cuba**.

*Type substrate*: Dead sticks.

*Notes*: Status unclear. Not *Fusarium fide*Wollenweber & Reinking (1935).  

*tonkinense Fusarium* (Bugnic.) O'Donnell *et al.*, Index Fungorum 440: 4. 2020.

***Neocosmospora tonkinensis*** (Bugnic.) Sand.-Den. & Crous, Persoonia 41: 126. 2018.

*Basionym*: *Cylindrocarpon tonkinense* Bugnic., Encyclop. Mycol.11: 181. 1939.

*Synonym*: *Fusarium ershadii* M. Papizadeh *et al.*, Europ. J. Pl. Pathol. 151: 693. 2018, *nom. illegit.*, Art. 52.1.

*Holotypus*: IMI 113868.

*Ex-type culture*: CBS 115.40 = IMI 113868.

*Type locality*: **Vietnam**, Tonkin.

*Type substrate*: *Musa sapientum*.

*Diagnostic DNA barcodes*: *rpb1*: MW218140; *rpb2*: LT960564; *tef1*: LT906672.  

***torreyae Fusarium*** T. Aoki *et al.*, Mycologia 105: 314. 2013.

*Holotypus*: BPI 884050.

*Ex-type culture*: CBS 133858 = MAFF 243468 = NRRL 54151.

*Type locality*: **USA**, Florida, Liberty County, Torreya State Park, Aspalaga Tract.

*Type substrate*: Stem tissue of diseased *Torreya taxifolia*.

*Descriptions and illustrations*: See Aoki *et al.* (2013).

*Diagnostic DNA barcodes*: *rpb1*: MW928819; *rpb2*: MW928834; *tef1*: MW928845.  

*tortuosum Fusarium* Thüm. & Pass., Pilze Weinst.: 51. 1878.

***Neofabraea vagabunda*** (Desm.) P.R. Johnst., IMA Fungus 5: 103. 2014.

*Basionym*: *Phlyctema vagabunda* Desm., Ann. Sci. Nat., Bot., sér. 3, 8: 16. 1847.

*Synonyms*: *Rhabdospora vagabunda* (Desm.) Zerov, Viznachnik gribiv Ukraini. T. 3. Nezaversheni gribi: 501. 1971, *nom. inval*., Art. 41.1.

*Rhabdospora vagabunda* (Desm.) R.S. Mathur*, Coelomycetes of India*: 234. 1979.

*Gloeosporium tortuosum* (Thüm. & Pass.) Sacc., Michelia 2: 117. 1880.

*Myxosporium tortuosum* (Thüm. & Pass.) Allesch., Rabenh. Krypt.-Fl., Ed. 2, 1(7): 534. 1903.

?*Fusarium obtusatum* Corda, Icon. Fung. 1: 3. 1837.

*Fusarium bipunctatum* Preuss, Linnaea 25: 741. 1852.

*Lituaria riessii* Schulzer, Verh. K. K. Zool.-Bot. Ges. Wien 21: 1241. 1871.

*Gloeosporium riessii* (Schulzer) Schulzer & Sacc., Hedwigia 23: 110. 1884.

*Gloeosporium tineum* Sacc., Michelia 1: 219. 1878.

*Gloeosporium frigidum* Sacc., Michelia 2: 168. 1880.

*Cylindrosporium frigidum* (Sacc.) Vassiljevsky, Fungi Imperfecti Parasitici 2: 515. 1950.

*Gloeosporium pyrenoides* Sacc. & Malbr., in Saccardo, Michelia 2: 633. 1882.

*Gloeosporium phillyreae* Pass., Atti Reale Accad. Lincei, Rendiconti Cl. Sci. Fis., sér. 4, 4: 103. 1888.

*Gloeosporium allantosporum* Fautrey, Rev. Mycol. (Toulouse) 14: 97. 1892.

*Gloeosporium allantoideum* Peck, Rep. (Annual) Regents Univ. State New York New York State Mus. 45: 81. 1893.

*Gloeosporium alutaceum* Sacc., Malpighia 11: 317. 1897.

*Allantozythia alutacea* (Sacc.) Höhn., Ann. Mycol. 22: 203. 1924.

*Phlyctema alutacea* (Sacc.) Petr., Ann. Mycol. 27: 370. 1929.

*Fusarium japonicum* Allesch., Beibl. Hedwigia 36: (164). 1897.

*Gloeosporium unedonis* Traverso, R.C. Congr. Bot. Palermo, 1902: 3 (extr.). 1902.

*Trichoseptoria fructigena* Maubl., Bull. Trimestriel Soc. Mycol. France 21: 95. 1905.

*Gloeosporium beguinotii* Sacc., in Potebnia, Ann. Mycol. 5: 20. 1907.

*Cylindrosporium olivae* Petri, Ann. Mycol. 5: 324. 1907.

*Gloeosporium olivae* (Petri) Foschi, Ann. Sperim. Agrar, n.s. 9: 911. 1955.

*Gloeosporium album* Osterw., Centralbl. Bacteriol. Parasitenk., 2. Abth., 18: 826. 1907.

*Gloeosporium diervillae* Grove, J. Bot. 60: 145. 1922.

*Pezicula alba* E.J. Guthrie, Trans. Brit. Mycol. Soc. 42: 504. 1959.

*Neofabraea alba* (E.J. Guthrie) Verkley, Stud. Mycol. 44: 125. 1999.

*Holotypus*: ?PARMA.

*Type locality*: **Italy**, Parma.

*Type substrate*: Dry twigs of *Vitis vinifera*.

*Note*: Synonyms *fide*Wollenweber & Reinking (1935).  

*torulosum Fusarium* (Berk. & M.A. Curtis) Gruyter & J.H.M. Schneid., Jaarb. Plantenziektenkundige Dienst, Wageningen 1989/1990, no. 168: 135. 1991, *nom. inval*., Art. 41.4.

*Basionym*: *Fusidium torulosum* Berk. & M.A. Curtis, Grevillea 3: 112. 1875.

(See ***Fusarium torulosum*** (Berk. & M.A. Curtis) Nirenberg)  

***torulosum Fusarium*** (Berk. & M.A. Curtis) Nirenberg, Mycopathologia 129: 136. 1995.

*Basionym*: *Fusidium torulosum* Berk. & M.A. Curtis, Grevillea 3: 112. 1875.

*Synonyms*: *Fusoma torulosum* (Berk. & M.A. Curtis) Sacc., Syll. Fung. 4: 220. 1886.

*Fusarium torulosum* (Berk. & M.A. Curtis) Gruyter & J.H.M. Schneid., Jaarboek. Plantenziektenkundige Dienst. Wageningen 1989/1990 no. 168: 135. 1991, *nom. inval*., Art. 41.4.

*Fusarium sclerodermatis* Oudem., Nederl. Kruidk. Arch. ser. 2, 5: 516. 1889.

*Fusarium sambucinum* var. *coeruleum* Wollenw., Ann. Mycol. 15: 55. 1917.

?*Gibberella pulicaris* var. *minor* Wollenw., Z. Parasitenk. (Berlin) 3: 356. 1931.

*Syntype*: ?Car Inf. no. 6034. in K(M).

*Type locality*: **USA**, Pennsylvania, Michener.

*Type substrate*: Decaying *Brassica* stalks or *Pinus*.

*Descriptions and illustrations*: See Nirenberg (1995).  

***toxicum Fusarium*** L. Lombard & J.W. Xia, Persoonia 43: 220. 2019.

*Holotypus*: CBS H-24071.

*Ex-type culture*: CBS 406.86 = FRC R-8507 = IMI 309347 = NRRL 25796.

*Type locality*: **Germany**, Berlin.

*Type substrate*: Soil.

*Descriptions and illustrations*: See Xia *et al.* (2019).

*Diagnostic DNA barcodes*: *rpb2*: MN170441; *tef1*: MN170508.  

*tracheiphilum Fusarium* (E.F. Sm.) Wollenw., Phytopathology 3: 29. 1913.

*Basionym*: *Neocosmospora vasinfecta* var. *tracheiphila* E.F. Sm., Bull. Div. Veg. Physiol. Pathol. U.S.D.A. 17: 45. 1899.

(See *Fusarium neocosmosporiellum*)

Syntypes: IN BPI, F, FLAS, ISC, MICH, PUL, UC & WSP.

*Type locality*: **USA**, South Carolina, James Island.

*Type substrate*: Dead stem of *Vigna sinensis.*

*Note*: Published as a new name for the sporodochial morph found on the authentic material of *N. vasinfecta* var. *tracheiphila*.  

*translucens Fusarium* Berk. & Broome, Ann. Mag. Nat. Hist., ser. 4, 17: 141. 1876.

*Holotypus*: ?K(M).

*Type locality*: **UK**, Scotland, Glamis.

*Type substrate*: Wood.

*Notes*: Status unclear. Not *Fusarium fide*Wollenweber & Reinking (1935).  

***transvaalense Fusarium*** Sand.-Den. *et al.*, MycoKeys 34: 82. 2018.

*Holotypus*: CBS H-23497.

*Ex-type culture*: CBS 144211.

*Type locality*: **South Africa**, Kruger National Park, Skukuza, Granite Supersite.

*Type substrate*: Rhizosphere of *Sida cordifolia*.

*Descriptions and illustrations*: See Sandoval-Denis *et al.* (2018b).

*Diagnostic DNA barcodes*: *rpb1*: LT996210; *rpb2*: LT996157; *tef1*: LT996099.  

*tremelloides Fusarium* Grev., Scott. Crypt. Fl. 1: 10. 1822.

***Calloria tremelloides*** (Grev.) L. Lombard, ***comb. nov*.** MycoBank MB 837723.

*Basionym*: *Fusarium tremelloides* Grev., Scott. Crypt. Fl. 1: 10. 1822.

*Synonyms*: *Peziza fusarioides* Berk., Mag. Zool. Bot. 1: 46. 1837.

*Calloria fusarioides* (Berk.) Fr., Summa Veg. Scand. 2: 359. 1849.

*Callorina fusarioides* (Berk.) Korf, Phytologia 21: 203. 1971.

*Peziza neglecta* Lib., Pl. Crypt. Arduenna Fasc. 1: no. 29. 1830.

*Calloria neglecta* (Lib.) B. Hein, Beih. Willdenowia 9: 54. 1976.

*Holotypus*: Not located.

*Type locality*: **UK**, Scotland, near Edinburg.

*Type substrate*: Dead stems of *Urtica dioica.*

*Notes*: Synonyms *fide*Wollenweber & Reinking (1935). As the epithet of *F. tremelloides* (1822) takes priority above the epithet of *C. neglecta* (1830), a new combination is introduced here.  

*trichothecioides Fusarium* Wollenw., J. Wash. Acad. Sci. 2: 147. 1912.

*Synonyms*: *Fusarium sambucinum* var. *trichothecioides* (Wollenw.) Bilaĭ, Fusarii (Biologija i sistematika): 268. 1955, *nom. inval*., Art. 41.1.

*Fusarium tuberivorum* Wilcox & G.K. Link, Res. Bull. Nebraska Agric. Exp. Sta. 1: 48. 1913.

*Lectotypus* (*hic designatus*, MBT 10000751): **USA**, rotten tuber of *Solanum tuberosum*, Aug. 1912, H.W. Wollenweber, in J. Wash. Acad. Sci. 2: 150, figs A–F.

*Descriptions and illustrations*: See Booth (1971) and Gerlach & Nirenberg (1982).

*Notes*: A putative synonym of *F. sulphureum* (Gordon 1959, Subramanian 1971, Gerlach & Nirenberg 1982) or *F. sambucinum* (Nelson *et al.* 1983, Nirenberg 1995). The taxonomy of this potato pathogen has not yet been resolved. As no holotype specimen was preserved (Gerlach & Nirenberg 1982), the figures accompanying the original protologue are designated as lectotype here.  

***tricinctum Fusarium*** (Corda) Sacc., Syll. Fung. 4: 700. 1886.

*Basionym*: *Selenosporium tricinctum* Corda, Icon. Fung. 2: 7. 1838.

*Synonyms*: *Fusarium sporotrichioides* var. *tricinctum* (Corda) Raillo, Fungi of the Genus Fusarium: 197. 1950.

*Fusarium sporotrichiella* var. *tricinctum* (Corda) Bilaĭ, Yadovitye griby na zerne khlebnykh zlakov. Kiev: 87. 1953, *nom. inval*., Art. 39.1.

*Fusarium sporotrichiella* var. *tricinctum* (Corda) Bilaĭ, Mikrobiol. Zhurn. 49: 7. 1987, *nom. inval*., Art. 35.1.

?*Vermicularia subeffigurata* γ *helianthi* Schwein., Trans. Amer. Philos. Soc., n.s. 4: 228. 1832 [1834].

?*Fusarium helianthi* (Schwein.) Wollenw., Fusaria Autogr. Delin. 2: 555. 1924.

*Fusarium muentzii* Delacr. (as ‘*müntzii*’), Bull. Soc. Mycol. France 8: 192. 1892.

*Fusarium citriforme* Jamal., Valt. Maatalousk. Julk. 123: 11. 1943.

*Gibberella tricincta* El-Gholl *et al.*, Canad. J. Bot. 56: 2206. 1978.

*Lectotypus*: PRM 155623 (designated in Holubová-Jechová *et al.* 1994).

*Type locality*: **Czech Republic**, near Prague, Chuchle, Vyskočilka.

*Type substrate*: Stem of *Umbelliferae*.

*Epitypus*: In PRM, designated in Holubová-Jechová *et al.* (1994).

*Ex-epitype culture*: BBA 64485 = CBS 393.93 = NRRL 25481.

*Epitype locality*: **Germany**, Berlin.

*Epitype substrate*: Culm base of *Triticum aestivum*.

*Descriptions and illustrations*: See Holubová-Jechová *et al.* (1994) and Leslie & Summerell (2006).

*Diagnostic DNA barcodes*: *rpb1*: JX171516; *rpb2*: JX171629; *tef1*: MH582379.  

*trifolii Fusarium* Jacz., Jahrb. Pflanzenkrankh. Russlands. VII-VIII: Abt. 6. 1917.

(See ***Fusarium oxysporum***)

*Holotypus*: Not located.

*Type locality*: **Russia**, St. Petersburg.

Type substrate: Root crown of *Trifolium* sp.

*Note*: Synonym *fide*Wollenweber & Reinking (1935).  

***triseptatum Fusarium*** L. Lombard & Crous, Persoonia 43: 34. 2018 [2019].

*Holotypus*: CBS H-23622.

*Ex-type culture*: CBS 258.50 = NRRL 36389.

*Type locality*: **USA**.

*Type substrate*: *Ipomoea batatas*.

*Descriptions and illustrations*: See Lombard *et al.* (2019b).

*Diagnostic DNA barcodes*: *rpb1*: MW928820; *rpb2*: MH484873; *tef1*: MH484964.  

*tritici Fusarium* Liebman bis, Tidsskr. Landoekon., n.s., 2: 515. 1840.

(See ***Fusarium avenaceum***)

*Lectotypus* (*hic designates*, MBT 10000752): **Denmark**, *Triticum* sp., in Tidsskr. Landoekon., n.s., 2: figs B, 1, 2.

*Notes*: Synonymy *fide*Rostrup (1894). No holotype specimen could be located and therefore an illustration is designated as lectotype.  

*tritici Fusarium* Erikss., Fungi Paras. Scand. Exs. no. 400. 1891, *nom. illegit.*, Art. 53.1.

(See *Fusarium nivale*)

*Authentic material*: CHRB-F-0007556.

*Original locality*: **Sweden**, Stockholm.

*Original substrate*: *Triticum durum.*

*Note*: Synonym *fide*Wollenweber & Reinking (1935).  

*truncatum Fusarium* Sherb., Mem. Cornell Univ. Agric. Exp. Sta. 6: 155. 1915.

(See ***Fusarium avenaceum***)

*Typus*: ?CUP-007429.

*Type locality*: **USA**, New York.

*Type substrate*: *Solanum tuberosum.*

*Note*: Synonym *fide*Wollenweber & Reinking (1935). Lectotypification pending study of the material lodged in CUP.  

*tuaranense Fusarium* T. Aoki *et al.*, Mycologia 111: 926. 2019.

***Neocosmospora tuaranensis*** (T. Aoki *et al.*) L. Lombard & Sand.-Den., ***comb. nov*.** MycoBank MB 837724.

*Basionym*: *Fusarium tuaranense* T. Aoki *et al.*, Mycologia 111: 926. 2019.

*Holotypus*: BPI 910971.

*Ex-type culture*: ATCC 16563 = MAFF 246842 = NRRL 22231.

*Type locality*: **Malaysia**, Sabah State, Tuaran.

*Type substrate*: *Hevea brasiliensis* damaged by an unknown ambrosia beetle*.*

*Descriptions and illustrations*: See Aoki *et al.* (2019).

*Diagnostic DNA barcodes*: *rpb1*: KC691600; *rpb2*: KC691660, KC691631; *tef1*: KC691542.

*Note*: A new combination is provided in the genus *Neocosmospora* based on the phylogenetic relationship and morphology of this species (Aoki *et al.* 2019).  

*tubercularioides Fusarium* (Corda) Sacc., Syll. Fung. 4: 697. 1886.

*Basionym*: *Selenosporium tubercularioides* Corda, Icon. Fung. 1: 7. 1837.

(See ***Fusarium avenaceum***)

*Typus*: PRM 155625.

*Type locality*: **Czech Republic**, Liberec, Hamrštejn (as ‘Sudetenland, Reichenberg, Hammerstein’).

*Type substrate*: Dead branches of *Rubus idaeus*.

*Descriptions and illustrations*: See Holubová-Jechová *et al.* (1994).

*Note*: Synonym *fide*Wollenweber & Reinking (1935). Lectotypification pending study of the material lodged in PRM.  

*tuberis Fusarium* Preuss, Linnaea 24: 148. 1851.

*Holotypus*: In B *fide* Jülich (1974).

*Type locality*: **Germany**, Hoyerswerda.

*Type substrate*: Tuber of *Dahlia* sp.

*Note*: Status unclear. Not *Fusarium fide*Wollenweber & Reinking (1935).  

*tuberivorum Fusarium* Wilcox & G.K. Link, Res. Bull. Nebraska Agric. Exp. Sta. 1: 48. 1913.

(See *Fusarium trichothecioides*)

*Lectotypus* (*hic designates*, MBT 10000753): **USA**, Nebraska, *Solanum tuberosum*, in Res. Bull. Nebraska Agric. Exp. Sta. 1, Pl. 24.

*Notes*: Synonym *fide*Wollenweber & Reinking (1935). No holotype specimen could be located and therefore an illustration is designated as lectotype.  

*tucumaniae Fusarium* T. Aoki *et al.*, Mycologia 95: 664. 2003.

(See *Fusarium azukiicola*)

*Holotypus*: BPI 841955.

*Ex-type culture*: MAFF 238418 = MJ-172 = NRRL 31096.

*Type locality*: **Argentina**, Tucumán, San Agustin.

*Type substrate*: *Glycine max*.

*Descriptions and illustrations*: See Aoki *et al.* (2003).

*Diagnostic DNA barcodes*: *rpb1*: MAED01000445; *rpb2*: EU329557; *tef1*: GU170636.  

***tumidum Fusarium*** Sherb., Phytopathology 18: 148. 1928.

*Synonym*: *Gibberella tumida* P.G. Broadh. & P.R. Johnst., Mycol. Res. 98: 730. 1994.

*Syntypes*: Krieger, *Fungi Saxon.* Exs. no. 2499 in BPI & HAL.

*Type locality*: **Germany**.

*Type substrate*: Heads of *Sarothamnus scoparius.*

*Note*: Typification pending further study of the syntypes.  

***tupiense Fusarium*** C.S. Lima *et al.*, Mycologia 104: 1414. 2012.

*Holotypus*: CMB-UB 22068.

*Ex-type culture*: CML 262 = CMM 3655 = KSU 16195 = NRRL 53984.

*Type locality*: **Brazil**, Minas Gerais, Lavras.

*Type substrate*: Diseased tissue of *Mangifera indica*.

*Descriptions and illustrations*: See Lima *et al.* (2012).

*Diagnostic DNA barcodes*: *rpb1*: LR792583; *rpb2*: LR792619; *tef1*: GU737404.  

***udum Fusarium*** E.J. Butler, Mem. Dept. Agric. India, Bot. Ser. 2(9): 54. 1910.

*Synonyms*: *Fusarium oxysporum f. sp. udum* (E.J. Butler) W.C. Snyder & H.N. Hansen, Amer. J. Bot. 24: 66. 1940.

*Fusarium butleri* Wollenw., Phytopathology 3: 38. 1913, *nom. illegit.*, Art. 52.1.

*Fusarium lateritium* var. *uncinatum* (Wollenw.) Wollenw., Z. Parasitenk. (Berlin) 3: 375. 1931.

*Fusarium vasinfectum* var. *crotalariae* Kulkarni, Indian J. Agric. Sci. 4: 994. 1934.

*Fusarium udum f. sp. crotalariae* (Kulkarni) Subram., The Genus Fusarium: 114. 1971.

*Fusarium udum* var. *cajani* Padwick, Indian J. Agric. Sci. 10: 878. 1940.

*Fusarium lateritium f. cajani* (Padwick) W.L. Gordon, Canad. J. Bot. 30: 232. 1952.

*Fusarium udum* var. *crotalariae* Padwick, Indian J. Agric. Sci. 10: 877. 1940.

*Fusarium lateritium f*. *crotalariae* (Padwick) W.L. Gordon, Canad. J. Bot. 30: 232. 1952.

*Gibberella indica* B. Rai & R.S. Upadhyay, Mycologia 74: 343. 1982.

*Lectotypus*: Butler (1910), Pl. IV, fig. 4, designated in Pfenning *et al.* (2019).

*Epitypus*: UB23905, designated in Pfenning *et al.* (2019).

*Ex-epitype culture*: BBA 65058 = CML 3238 = NRRL 25199.

*Type locality*: **India**.

*Type substrate*: *Cajanus cajan*.

*Descriptions and illustrations*: See Wollenweber & Reinking (1935), Booth (1971), Subramanian (1971), Booth (1978), Gerlach & Nirenberg (1982) and Pfenning *et al.* (2019).

*Diagnostic DNA barcodes*: *rpb2*: KY498875; *tef1*: MK639096.  

*udum Fusarium* (Berk.) Wollenw., Phytopathology 3: 38. 1913, *nom. illegit.*, Art. 53.1.

*Basionym*: *Fusisporium udum* Berk., Ann. Mag. Nat. Hist. 6: 438. 1841.

(See *Fusarium merismoides*)

*Holotypus*: ?K(M).

*Type locality*: **UK**, King's Cliffe.

*Type substrate*: Unidentified tree.

*Note*: Synonyms *fide*Wollenweber & Reinking (1935).  

*ulmi Fusarium* P. Crouan & H. Crouan, Fl. Finistère: 14. 1867.

(See *Fusarium candidum* (Link) Sacc.)

*Holotypus*: ?PC.

*Type locality*: **France**, Finistère, edge of a stream.

*Type substrate*: Roots of *Ulmus* sp.

*Note*: Synonym *fide*Wollenweber & Reinking (1935).  

*ulmicola Fusarium* Dearn. & House, Circ. New York Stat. Mus. 24: 60. 1940, *nom. inval*., Art. 39.1.

*Authentic material*: NYSf3256.

*Original locality*: **USA**, New York, Albany, Ravena.

*Original substrate*: Dead branches of *Ulmus thomasii*.

*Notes*: Lacks a Latin diagnosis. Requires further investigation to confirm its taxonomic affiliation.  

*uncinatum Fusarium* Wollenw., Ann. Mycol. 15: 54. 1917.

(See ***Fusarium udum***)

*Holotypus*: Not located.

*Type locality*: **India**, Dehli, Pusa.

*Type substrate*: Dried stem of *Cajanus indicus*.

*Note*: Synonym *fide*Wollenweber & Reinking (1935) and Gerlach & Nirenberg (1982).  

*uniseptatum Fusarium* Höhn., Ann. Mycol. 1: 409. 1903.

*Synonyms*: *Cylindrocarpon uniseptatum* (Höhn.) Wollenw., Fusaria Autogr. Delin. 2: 646. 1924.

*Ramularia uniseptata* (Höhn.) Wollenw., Fusaria Autogr. Delin. 2: 646. 1924.

*Holotypus*: Not located.

*Type locality*: **Austria**, Vienna.

*Type substrate*: Rotten *Gleditsia triacanthos*.

*Notes*: Status unclear. Not *Fusarium fide*Wollenweber & Reinking (1935) and not *Ramularia fide* Braun (1998).  

*uredinicola Fusarium* Jul. Müll., Ber. Deutsch. Bot. Ges. 3: 395. 1885.

(See ***Fusarium avenaceum***)

*Holotypus*: Not located.

*Type locality*: **Germany**.

*Type substrate*: Aecidium of *Phragmidium subcorticium* (= *Phragmidium mucronatum*) and *Phragmidium rubi* (= *Phragmidium barclayi*).

*Note*: Synonym *fide*Wollenweber & Reinking (1935).  

*uredinicola Fusarium* Pat. & Gaillard, Bull. Soc. Mycol. France 4: 127. 1888, *nom. illegit.*, Art. 53.1.

*Synonym: Fusarium patouillardii* Sacc. (as ‘*patouillardi*’), Syll. Fung. 10: 729. 1892.

*Authentic material*: Not located.

*Original locality*: **Venezuela**, Caracas.

*Original substrate*: Parasitic on the bottom of spots of *Puccinia pallidissima*, between the perithecia of *Darluca filum* parasitised by the *Puccinia* sp.

*Notes*: Status unclear. Not *Fusarium fide*Wollenweber & Reinking (1935).  

*uredinicola Fusarium* Petch, Ann. Roy. Bot. Gard. (Peradeniya) 6: 256. 1917, *nom. illegit.*, Art. 53.1.

*Authentic material*: PDA 4731.

*Original locality*: **Sri Lanka**, Hakgala.

*Original substrate*: Parasitic on *Uredo microglossa* on leaves of *Microglossa zeylanica*.

*Notes*: Status unclear. A probable synonym of *F. solani* var. *minus* (syn. *Neocosmospora brevicona*) according to Wollenweber & Reinking (1935).  

*uredinophilum Fusarium* Speg. (as ‘*urediniphilum*’), Anales Mus. Nac. Hist. Nat. Buenos Aires 31: 445. 1922.

*Holotypus*: In LPS (Fungi Parag. pp. 93–94, no. 262).

*Type locality*: **Paraguay**, near Puerto Sajonia.

*Type substrate*: Parasitic on the acervuli of *Uredo cyclotrauma*, on leaving leaves of *Pithecellobium cauliflorum*.

*Notes*: Status unclear. Not treated by any of Wollenweber & Reinking (1935), Booth (1971), or Gerlach & Nirenberg (1982).  

*uredinum Fusarium* Ellis & Everh., N. Amer. Fungi, Ser. II, no. 2799. 1890, *nom. inval*., Art. 38.1(a).

***Ramularia uredinis*** (W. Voss) Sacc., Syll. Fung. 4: 199. 1886.

*Basionym*: *Cylindrosporium uredinis* W. Voss, Verh. Zool.-Bot. Ges. Wien 29: 684. 1879.

*Synonym*: *Ramularia nambuana* Henn., Hedwigia 43: 146. 1904.

*Authentic material*: NY00928692.

*Original locality*: **USA**, Wisconsin, Racine.

*Original substrate*: Parasitic on uredinia of *Melampsora salicina*, on leaf of *Salix* sp.

*Notes*: Wollenweber & Reinking (1935) considered *F. uredinum* a synonym of *Cladosporium herbarum*. It is quite possible that this common saprobic *Cladosporium* species also occurred on uredinia in N. Am. Fungi 2799, but it can be ruled out that Ellis & Everhard confused this dematiaceous hyphomycete characterised by having long conidiophores with thickened and darkened conidiogeneous loci and large catenate conidia with a colourless *Fusarium*. Davis (1915) found *Ramularia uredinis*, a common mucedinacous hyphomycete on *Melampsora* spp. on *Populus* and *Salix*, in material authentic for this name. This is undoubtedly correct.  

*urticearum Fusarium* (Corda) Sacc., Syll. Fung. 4: 698. 1886.

*Basionym*: *Selenosporium urticearum* Corda, Icon. Fung. 2: 7. 1838.

(See ***Fusarium lateritium***)

*Lectotypus* (*hic designatus*, MBT 10000754): **Czech Republic**, Prague, dead branches of *Ficus elastica* and *Morus nigra*, 1838. A.C.J. Corda, in Icon. Fung. 2, Tab. 9, fig. 30.

*Notes*: Synonym *fide*Wollenweber & Reinking (1935). No holotype specimen could be located and therefore an illustration is designated as lectotype.  

***ussurianum Fusarium*** T. Aoki *et al.*, Mycologia 101: 847. 2009.

*Holotypus*: BPI 878845.

*Ex-type culture*: CBS 123752 = NRRL 45681 = TG-2662/0.

*Type locality*: **Russia**, Ussuriysk, Primorsky krai (Far East territory), agricultural field near the city Ussuriysk.

*Type substrate*: Seed of *Avena sativa*.

*Descriptions and illustrations*: See Yli-Mattila *et al.* (2009).

*Diagnostic DNA barcodes*: *rpb1*: KM361648; *rpb2*: KM361666; *tef1*: FJ240301.  

*ustilaginis Fusarium* Kellerm. & Swingle, Rep. (Annual) Kansas Agric. Exp. Sta. 2: 285. 1890 [1889].

(See ***Fusarium avenaceum***)

*Lectotypus* (*hic designatus*, MBT 10000755): **USA**, Kansas, Manhattan, on *Ustilago avenae*, on *Avena sativa*, 1890, W.A. Kellerman & W.T. Swingle, in Rep. (Annual) Kansas Agric. Exp. Sta. 2, pl. IX, figs 1–13.

*Note*: Synonym *fide*Wollenweber & Reinking (1935).  

*ustilaginis Fusarium* Rostr., Bot. Foren. Festskr. 54: 137. 1890, *nom. illegit.*, Art. 53.1.

(See *Fusarium nivale*)

*Authentic material*: C-F-125286.

*Original locality*: **Denmark**, Jutland, near Viborg.

*Original substrate*: Parasitic on *Ustilago grandis* on *Phragmites communis*.

*Note*: Synonym *fide*Wollenweber & Reinking (1935).  

*vanettenii Fusarium* O'Donnell *et al.*, Index Fungorum 440: 5. 2020.

*Basionym*: *Fusarium martii* var. *pisi* F.R. Jones, J. Agric. Res. 26: 459. 1923.

(See *Fusarium pisi*)  

*vasinfectum Fusarium* G.F. Atk., Bull. Alabama Agric. Exp. Sta. 41: 28. 1892.

(See ***Fusarium oxysporum***)

*Holotypus*: ?CUP-A-(0100)#1.

*Type locality*: **USA**, Alabama, Montgomery, Mathews.

*Type substrate*: *Gossypium herbaceum.*

*Note*: Synonym *fide*Wollenweber & Reinking (1935).  

***venenatum Fusarium*** Nirenberg, Mycopathologia 129: 136. 1995.

*Misapplied names*: *Fusarium sambucinum* var. *coeruleum* Wollenw. *sensu* Booth, The Genus Fusarium: 171–172. 1971.

*Fusarium sambucinum* var. *coeruleum* Wollenw. *sensu* Gerlach & Nirenberg, Mitt. Biol. Bundesanst. Land.- Forstw. 209: 213–216. 1982.

*Holotypus*: CBS 458.93 (preserved as metabolically inactive culture).

*Ex-type culture*: BBA 64537 = CBS 458.93 = NRRL 26228.

*Type locality*: **Austria**.

*Type substrate*: Culm of *Triticum aestivum*.

*Descriptions and illustrations*: See Nirenberg (1995).

*Diagnostic DNA barcodes*: *rpb2*: KM232382; *tef1*: KM231942.  

*venerorum Fusarium* Dounin & Goldmacher, Index of the plant diseases in the U.S. 5: 284–298. 1927.

(See ***Fusarium avenaceum***)

*Holotypus*: Not located.

*Type locality*: **Unknown**.

*Type substrate*: Unknown.

*Note*: Synonym *fide*Wollenweber & Reinking (1935).  

*venezuelense Fusarium* O'Donnell *et al.*, Index Fungorum 440: 5. 2020.

***Neocosmospora robusta*** Sand.-Den. & Crous, Persoonia 43: 165. 2019, *non Fusarium robustum* Gerlach 1977.

*Holotypus*: CBS H-24000.

*Ex-type culture*: BBA 65682 = CBS 145473 = NRRL 22395.

*Type locality*: **Venezuela**.

*Type substrate*: Bark.

*Descriptions and illustrations*: See Sandoval-Denis *et al.* (2019).

*Diagnostic DNA barcodes*: *rpb1*: MW834251; *rpb2*: EU329507; *tef1*: AF178341.  

*ventricosum Fusarium* Appel & Wollenw., Phytopathology 3: 32. 1913.

***Rectifusarium ventricosum*** (Appel & Wollenw.) L. Lombard & Crous, Stud. Mycol. 80: 229. 2015.

*Synonyms*: *Fusarium solani* var. *ventricosum* (Appel & Wollenw.) Joffe, Pl. & Soil 38: 440. 1973.

*Fusarium cuneiforme* Sherb., Mem. Cornell Univ. Agric. Exp. Sta. 6: 129. 1915.

*Hypomyces solani* Reinke & Berth., Untersuch. Bot. Lab. Univ. Göttingen 1: 27. 1879.

*Hyponectria solani* (Reinke & Berth.) Petch, J. Bot. 75. 220. 1937.

*Nectriopsis solani* (Reinke & Berth.) C. Booth, Mycol. Pap. 74: 8. 1960.

*Nectria ventricosa* Booth, The Genus Fusarium: 55. 1971.

*Holotypus*: B 70 0021849.

*Epitypus*: CBS H-21947, designated in Lombard *et al.* (2015).

*Ex-epitype culture*: BBA 62452 = CBS 748.79 = NRRL 20846 = NRRL 22113.

*Type locality*: **Germany**, Berlin.

*Type substrate*: Tuber of *Solanum tuberosum*.

*Descriptions and illustrations*: See Wollenweber (1917), Booth (1971) and Lombard *et al.* (2015).

*Diagnostic DNA barcodes*: *rpb*1: JX171484; *rpb*2: JX171597; *tef1*: KM231924.

*Notes*: Contrary to Wollenweber & Reinking (1935), Booth (1971) considered this species as different from *F. argillaceum*, which was later confirmed by Lombard *et al.* (2015). The same authors designated an epitype for this taxon and transferred it to the genus *Rectifusarium* as *R. ventricosum*.  

*veratri Fusarium* (Allesch.) Höhn., in Kabát & Bubák, Fungi Imperf. Exs. No. 349. 1906.

***Gloeosporium veratri*** (Allesch.) Höhn., Mitt. Bot. Inst. Tech. Hochsch. Wien 4: 112. 1927.

*Basionym*: *Fusoma veratri* Allesch., Ber. Bayer. Bot. Ges. 2: 19. 1892.

*Synonym*: *Septogloeum veratri* (Allesch.) Wollenw., Fusaria Autogr. Delin. 1: 439. 1916.

*Holotypus*: ?M.

*Type locality*: **Germany**, Bavaria, Oberammergau.

*Type substrate*: Leaves of *Veratrum lobelianum.*

*Notes*: This species produces acervuli and 1-septate conidia with truncate basal cells. Therefore, it was transferred to *Gloesporium* (*Helotiales*, *Dermataceae*).  

*verrucosum Fusarium* (Pat.) O'Donnell & Geiser, Phytopathology 103: 404. 2013.

***Albonectria verrucosa*** (Pat.) Rossman & Samuels, Stud. Mycol. 42: 108. 1999.

*Basionym*: *Calonectria verrucosa* Pat., Bull. Soc. Mycol. France 11: 228. 1895.

*Synonym*: *Nectria astromata* Rossman, Mycotaxon 8: 550. 1979, *non N. verrucosa* (Schwein.) Sacc.

*Holotypus*: In FH *fide*Rossman *et al.* (1999).

*Type locality*: **Ecuador**, San Jorge.

*Type substrate*: *Chusquea* sp.

*Descriptions and illustrations*: See Rossman (1983) and Rossman *et al.* (1999).

*Notes*: Although recently recombined in *Fusarium* (Geiser *et al.* 2013), the taxonomy of this species is uncertain. With 5–9(–13)-septate ascospores, this species cannot be a member of *Fusarium s. str*., and the identity of the isolates included in recent phylogenetic estimates (CBS 102163, originally identified as *F. concolor* and NRRL 22566) cannot be confirmed at this stage.  

*versicolor Fusarium* Sacc., Syll. Fung. 16: 1099. 1902.

(See ***Fusarium culmorum***)

*Holotypus*: In PAD.

*Type locality*: **France**, Côte-d’Or.

*Type substrate*: Cortex of *Cucurbita* sp.

*Note*: Synonym *fide*Wollenweber & Reinking (1935).  

*versiforme Fusarium* Kabát & Bubák, Hedwigia 44: 358. 1905.

*Holotypus*: BPI 453128.

*Type locality*: **Czech Republic**, Bohemia, Turnov.

*Type substrate*: Living leaves of *Hosta sieboldii* (syn. *Hosta albomarginata*).

*Notes*: Status unclear. Not *Fusarium fide*Wollenweber & Reinking (1935).  

***verticillioides Fusarium*** (Sacc.) Nirenberg, Mitt. Biol. Bundesanst. Land- Forstw. 169: 26. 1976.

*Basionym*: *Oospora verticillioides* Sacc., Fung. Ital., Fasc. 17–28: pl. 879. 1881.

*Synonyms*: *Alysidium verticillioides* (Sacc.) Kuntze, Revis. Gen. Pl. 3: 442. 1898.

*Fusarium moniliforme* J. Sheld., Annual Rep. Nebraska Agric. Exp. Sta. 17: 23. 1904.

*Gibberella moniliformis* Wineland, J. Agric. Res. 28: 909. 1924.

*Lectotypus*: Pl. 879 in Saccardo, Fung. Ital. (1881), designated by Yilmaz *et al.* (2021).

*Epitypus*: CBS 218.76 (preserved as metabolically inactive culture), designated by Yilmaz *et al.* (2021).

*Ex-epitype culture*: BBA 11782 = CBS 218.76 = DSM 62264 = IMI 202875 = NRRL 13993.

*Epitype locality*: **Germany**.

*Epitype substrate*: *Zea mays.*

*Descriptions and illustrations*: See Nirenberg (1976, 1981), Gerlach & Nirenberg (1982) and Leslie & Summerell (2006).

*Diagnostic DNA barcodes*: *rpb1*: MW402638; *rpb2*: MW928835; *tef1*: KF499582.  

***veterinarium Fusarium*** L. Lombard & Crous, Persoonia 43: 35. 2018 [2019].

*Holotypus*: CBS H-23623.

*Ex-type culture*: CBS 109898 = NRRL 36153.

*Type locality*: **Netherlands**.

*Type substrate*: Peritoneum of *Selachimorpha* (shark).

*Descriptions and illustrations*: See Lombard *et al.* (2019b).

*Diagnostic DNA barcodes*: *rpb2*: MH484899; *tef1*: MH484990.  

*victoriae Fusarium* Henn., in herb., *fide* Wollenweber, Fusaria Autogr. Delin. 1: 66. 1916.

***Macronectria jungneri*** (Henn.) C. Salgado & P. Chaverri, Fungal Diversity 80: 448. 2016. *Basionym*: *Nectria jungneri* Henn., Bot. Jahrb. Syst. 22: 75. 1895.

*Synonyms*: *Nectria eustoma* Penz. & Sacc., Malpighia 11: 509. 1898.

*Nectria leucocoma* Starbäck, Bih. Kongl. Svenska Vetensk.-Akad. Handl. 25: 28. 1899.

*Nectria cinereopapillata* Henn. & E. Nyman, Monsunia 1: 161. 1900.

*Nectria striatospora* Zimm., Centralbl. Bakteriol. Abt. 1, 7: 105. 1901.

*Cylindrocarpon victoriae* Wollenw., Z. Parasitenk. (Berlin) 1: 161. 1928.

*Nectria azureo-ostiolata* Yoshim. Doi, Mem. Nat. Sci. Mus. Tokyo 10: 23. 1977.

*Authentic material*: In B *fide* Wollenweber, Fusaria Autogr. Delin. 1: 66. 1916.

*Original locality*: **Cameroon**.

*Original substrate*: Trunk of an unknown tree.  

*vinosum Fusarium* Massee, Brit. Fung.-Fl. 3: 479. 1893.

(See ***Fusarium flocciferum***)

*Holotypus*: ?K(M).

*Type locality*: **UK**.

*Type substrate*: Decaying mast manufactured from *Fagus sylvatica*.

*Note*: Synonym *fide*Wollenweber & Reinking (1935).  

*vinosum Fusarium* Greco, Origine des Tumeurs (Etiologie du Cancer. *etc*.) et Observations de Mycoses (Blastomycoses. *etc*.) Argentines (Buenos Aires): 670. 1916, *nom. illegit.*, Art. 53.1.

*Authentic material*: Not located.

*Original locality*: **Argentina**.

*Original substrate*: *Homo sapiens*.

*Note*: A late homonym of *F. vinosum* Massee.  

*violaceum Fusarium* P. Crouan & H. Crouan, Fl. Finistère: 14. 1867, *nom. illegit.*, Art. 53.1.

(See ***Fusarium sambucinum***)

*Authentic material*: ?PC.

*Original locality*: **France**, Brittany, Finistère, marshes.

*Original substrate*: Bark of unknown tree.

*Notes*: An illegitimate homonym of *F. violaceum*Fuckel (1863). Synonym *fide*Gams *et al.* (1997).  

*violaceum Fusarium* Fuckel, Fungi Rhen. Exs. No. 209. 1863.

(See *Fusarium caeruleum*)

*Syntypes*: In BPI, F, HAL, MICH, S & WSP (Fuckel, Fungi Rhen. Exs. No. 209).

*Type locality*: **Germany**, Hessen, Oestrich.

*Type substrate*: *Solanum tuberosum.*

*Note*: Synonym *fide*Wollenweber & Reinking (1935) and Booth (1971).  

*violae Fusarium* F.A. Wolf, Mycologia 2: 21. 1910.

(See ***Fusarium oxysporum***)

*Holotypus*: Not located.

*Type locality*: **USA**, Nebraska, Lincoln.

*Type substrate*: Stems and roots of *Viola tricolor*.

*Note*: Synonym *fide*Wollenweber & Reinking (1935).  

*virguliforme Fusarium* O'Donnell & T. Aoki, Mycologia 95: 667. 2003.

(See *Fusarium azukicola*)

*Holotypus*: BPI 841956.

*Ex-type culture*: MAFF 238553 = NRRL 31041 = Shuxian Li # 95.

*Type locality*: **USA**, Illinois.

*Type substrate*: *Glycine max*.

*Descriptions and illustrations*: See Aoki *et al.* (2003).

*Diagnostic DNA barcodes*: *rpb1*: JX171530; *rpb2*: JX171643; *tef1*: AY220193.  

*viride Fusarium* (Lechmere) Wollenw., Fusaria Autogr. Delin. 1: 418. 1916.

*Basionym*: *Pionnotes viridis* Lechmere, Compt. Rend. Hebd. Séances Acad. Sci. 155: 178. 1912.

(See *Fusarium solani*)

*Holotypus*: Not located.

*Type locality*: **Ivory Coast**.

*Type substrate*: Undetermined wood.

*Note*: Synonyms *fide*Wollenweber & Reinking (1935).  

*viticola Fusarium* Thüm. (as ‘*viticolum*’), Pilze Weinst.: 52. 1878.

*Synonym*: *Fusarium herbarum* var. *viticola* (Thüm.) Wollenw., Fusaria Autogr. Delin. 3: 898. 1930.

(See ***Fusarium avenaceum***)

*Lectotypus* (*hic designatus*, MBT 10000756): **Italy**, Liguria, Genoa, Rapallo, dry twigs of *Vitis vinifera*, Jul. 1876, G. Passerini, in Thümen, Pilze Weinst. 1878: pl. 3, fig. 3.

*Notes*: Synonyms *fide*Wollenweber & Reinking (1935). No holotype specimen could be located and therefore an illustration is designated as lectotype.  

*vogelii Fusarium* Henn., Z. Pflanzenkrankh. 12: 16. 1902.

*Synonyms*: *Septosporium curvatum* Rabenh. & A. Braun, Krankh. Pfl.: 14. 1854.

*Septoria curvata* (Rabenh. & A. Braun) Sacc., Syll. Fung. 3: 484. 1884.

*Cercospora curvata* (Rabenh. & A. Braun) Wollenw., Fusaria Autogr. Delin. 1: 451. 1916.

*Holotypus*: In B (Kabát & Bubák, Fungi Imp. Exs. 248) *fide* Hein (1988).

*Type locality*: **Poland**, Dąbroszyn (former Tamsel)*.*

*Type substrate*: Leaf of *Robinia pseudoacacia.*

*Notes*: Status unclear. Neither *Fusarium fide*Wollenweber & Reinking (1935) nor *Cercospora fide* Chupp (1954).  

***volatile Fusarium*** Al-Hatmi *et al.*, Fungal Syst. Evol. 4: 174. 2019.

*Holotypus*: CBS H-24004.

*Ex-type culture*: CBS 143874.

*Type locality*: **French Guiana**, Cayenne.

*Type substrate*: Bronchoalveolar lavage effusion from *Homo sapiens* with lung infection.

*Descriptions and illustrations*: See Al-Hatmi *et al.* (2019).

*Diagnostic DNA barcodes*: *rpb2*: LR596006; *tef1*: LR596007.  

*volutella Fusarium* Ellis & Everh., Proc. Acad. Nat. Sci. Philadelphia 43: 93. 1891.

(See *Fusarium stromaticum*)

*Holotypus*: Langlois 1505 in NY *fide* Index Fungorum.

*Type locality*: **USA**, Louisiana, Saint Martinsville.

*Type substrate*: Dead twigs of *Nekemias arborea* (syn. *Ampelopsis arborea*).

*Note*: Synonym *fide*Wollenweber & Reinking (1935) and Gräfenhan *et al.* (2011).  

***vorosii Fusarium*** B. Tóth *et al.*, Fungal Genet. Biol. 44: 1202. 2007.

*Holotypus*: BPI 871658.

*Ex-type culture*: NRRL 37605.

*Type locality*: **Hungary**, Pest, Ipolydamásd.

*Type substrate*: Spikelet of *Triticum aestivum*.

*Descriptions and illustrations*: See Starkey *et al.* (2007).

*Diagnostic DNA barcodes*: *rpb1*: KM361647; *rpb2*: KM361665; *tef1*: DQ459745.  

*waltergamsii Fusarium* O'Donnell *et al.*, Index Fungorum 440: 5. 2020.

***Neocosmospora gamsii*** Sand.-Den. & Crous, Persoonia 41: 116. 2018.

*Holotypus*: CBS H-23226.

*Ex-type culture*: CBS 143207 = NRRL 32323 = UTHSC 99-250.

*Type locality*: **USA**, Pennsylvania.

*Type substrate*: Bronchoalveolar lavage fluid from *Homo sapiens*.

*Descriptions and illustrations*: See Sandoval-Denis & Crous (2018).

*Diagnostic DNA barcodes*: *rpb1*: MW834223; *rpb2*: KM361665; *tef1*: DQ246951.  

***werrikimbe Fusarium*** J.L. Walsh, L.W. Burgess, E.C.Y. Liew & B.A. Summerell, ***sp. nov*.** MycoBank MB 837725.

*Synonym*: *Fusarium werrikimbe* J.L. Walsh, L.W. Burgess, E.C.Y. Liew & B.A. Summerell, Fungal Diversity 44: 155. 2010, *nom. inval.*, Art. 40.7.

*Etymology*: In reference to Werrikimbe National Park, the geographic origin of the isolates first recognised as belonging to this species.

For diagnosis see Walsh *et al.*, Fungal Diversity 44: 155. 2010.

*Holotypus*: CBS 125535 (preserved as metabolically inactive culture).

*Ex-type culture*: CBS 125535 = F19350 = RBG 5332.

*Type locality*: **Australia**, New South Wales, Werrikimbe National Park.

*Type substrate*: *Sorghum leiocladum*.

*Descriptions and illustrations*: See Walsh *et al.* (2010).

*Diagnostic DNA barcodes*: *rpb1*: MW928821; *rpb2*: MN534304; *tef1*: MW928846.

*Notes*: Walsh *et al.* (2010) did not indicate the holotype for *F. werrikimbe*, rendering the name invalid (Art. 40.7). Here we validate the name.  

*willkommii Fusarium* Lindau, Rabenh. Krypt.-Fl. ed. 2, 1(9): 551. 1910.

*Replaced synonym*: *Fusarium candidum* Sacc. & D. Sacc., Syll. Fung. 18: 674. 1906, *nom. illegit.*, Art. 53.1, *non Fusarium candidum* Ehrenb. 1818.

*Lectotypus* (*hic designatus*, MBT 10000757): **Germany**, Saxony, *Fagus sylvatica*, 1866, M. Willkomm, in Die mikroskopischen Feinde des Waldes 1, Tab. VI, figs 11–12.

*Notes*: Lindau's description of *F. willkommii* was based on Willkomm's (1866: 103) description and illustration under the name *Fusidium candidum* Link as well as Saccardo's (*l.c*.) description under *Fusarium candidum*. Therefore, the illustration by Willkomm (1866) is designated as lectotype.  

*witzenhausenense Fusarium* Šišić *et al.*, Antonie van Leeuwenhoek 111: 1795. 2018.

(See *Fusarium stercicola*)

*Holotypus*: CBS H-23351.

*Ex-type culture*: CBS 142480 = DSM 106212.

*Type locality*: **Germany**, Hessen, Witzenhausen, Neu-Eichenberg.

*Type substrate*: Branch of *Hibiscus* sp.

*Descriptions and illustrations*: See Šišić *et al.* (2018a).

*Diagnostic DNA barcodes*: *rpb1*: MG237865; *rpb2*: LR583886; *tef1*: KY556525.  

*wolgense Fusarium* Rodigin, Trudy Bashkir. Sel’. Khoz. Inst. 3: 101. 1942.

*Holotypus*: Not located.

*Type locality*: **Russia**, Volgograd (formerly Stalingrad).

*Type substrate*: Fruit of *Citrullus lanatus* (syn. *Citrullus vulgaris*).

*Notes*: Status unclear. Not treated by either of Booth (1971) and Gerlach & Nirenberg (1982).  

*wollenweberi Fusarium* Raillo, Fungi of the Genus Fusarium: 189. 1950, *nom. illegit.*, Art. 52.1.

(See ***Fusarium anthophilum***)

*Authentic material*: Not located.

*Original locality*: **Azerbaijan**.

*Original substrate*: Seeds and stems of *Gossypium* sp.

*Descriptions and illustrations*: See Raillo (1950).

*Notes*: *Fusarium wollenweberi* was published as a new combination, but no basionym was indicated. As a *nomen novum*, it can only be based on *F. anthophilum*, the only cited name, which is a valid name. Therefore, *F. wollenweberi* would be illegitimate (*nom. superfl*., Art. 52.1.). Additionally, the condition for the introduction of a new species is also not met as a Latin diagnosis, necessary in 1950, is lacking.  

*xiangyunense Fusarium* F. Zhang *et al.* (as ‘*xiangyunensis*’), Phytotaxa 450: 278. 2020, *nom. inval*., Art. 40.8.

(See *Fusarium stercicola*)

*Authentic material*: DLU11-1, School of Agriculture and Biology, Dali University, China.

*Authentic culture*: CGMCC 3.19676.

*Original locality*: **China**, Yunnan, Xiangyun, Dali, Da-bo-na hot-spring.

*Original substrate*: Waterlogged soil.

*Descriptions and illustrations*: See Zhang *et al.* (2020).

*Diagnostic DNA barcodes*: *rpb1*: MH999281; *tef1*: MH992629.

*Note*: Based on phylogenetic and morphological evidence provided by Zhang *et al.* (2020), this invalid name (Art. 40.8) belongs to the genus *Neocosmospora* and is a synonym of *N. stercicola*.  

***xylarioides Fusarium*** Steyaert, Bull. Soc. Roy. Bot. Belgique 80: 42. 1948.

*Synonyms*: *Gibberella xylarioides* (Steyaert) R. Heim & Saccas, Rev. Mycol. (Paris) 15 (Suppl. Colon.): 97. 1950.

*Fusarium oxysporum f*. *xylarioides* (Steyaert) Delassus, Bull. sci. Minist. Colon., Sect. Agric. trop. 5: 347. 1954.

*Lectotypus* (*hic designatus*, MBT 10000758): **Central African Republic**, Bangui, trunk of *Coffea excelsa*, 1939, H. Frédéric, in Steyaert, Bull. Soc. Roy. Bot. Belgique 80, pl. I, fig. 8.

*Epitypus* (*hic designatus*, MBT 10001275): **Ivory Coast**, on trunk of *Coffea* sp., Feb. 1951, C. & M. Moreau, CBS 258.52 (preserved as metabolically inactive culture).

*Ex-epitype culture*: CBS 258.52 = NRRL 25486.

*Descriptions and illustrations*: See Steyaert (1948), Booth (1971), Gerlach & Nirenberg (1982) and Geiser *et al.* (2005).

*Diagnostic DNA barcodes*: *rpb1*: JX171517; *rpb2*: JX171630; *tef1*: AY707136.

*Notes*: A lectotype is designated here based on an illustration provided by Steyaert (1948) accompanying the original protologue. All attempts to locate the holotype specimen lodged at the Université de Bangui (BANG), Central African Republic, as indicated by Steyaert (1948), failed. In addition, an epitype (CBS 258.52) is designated here to provide taxonomic stability for this important species.  

***xyrophilum Fusarium*** I. Laraba *et al.*, Mycologia 112: 45. 2019 [2020].

*Holotypus*: BPI 910919.

*Ex-type culture*: FRC M-8921 = NRRL 62721.

*Type locality*: **Guyana**, Cuyuni-Mazaruni, Kamakusa Mountain.

*Type substrate*: *Xyris surinamensis*.

*Descriptions and illustrations*: See Laraba *et al.* (2020).

*Diagnostic DNA barcodes*: *rpb1*: MN193933; *rpb2*: MN193905; *tef1*: MN193877.  

*yamamotoi Fusarium* O'Donnell *et al.*, Index Fungorum 440: 5. 2020.

*Replaced synonym*: *Nectria elegans* W. Yamam. & Maeda, Hyogo Univ. Agric. ser. Agric. Biol. 3: 15. 1957, *non Fusarium elegans* Appel & Wollenw. 1910.

***Neocosmospora elegans*** (W. Yamam. & Maeda) Sand.-Den. & Crous, Persoonia 43: 127. 2019.

*Lectotypus*: Figs 1–9, page 16, in Yamamoto *et al.* (1957), designated in Sandoval-Denis *et al.* (2019).

*Epitypus*: CBS H-23980, designated in Sandoval-Denis *et al.* (2019).

*Ex-epitype culture*: ATCC 42366 = CBS 144396 = MAFF 238541 = NRRL 22277 = SUF XV-1.

*Type locality*: **Japan**.

*Type substrate*: Twigs and trunks of *Zanthoxylum piperitum*.

*Descriptions and illustrations*: See Sandoval-Denis *et al.* (2019).

*Diagnostic DNA barcodes*: *rpb1*: MW218113; *rpb*2: FJ240380; *tef1*: AF178336.  

*yuccae Fusarium* Cooke, Grevillea 7: 34. 1878, *nom. inval*., Art. 36.1(a).

(See ***Fusarium lateritium***)

*Authentic material*: BPI 453149.

*Original locality*: **USA**, South Carolina, Aiken.

*Original substrate*: *Yucca aloifolia*.

*Note*: Synonym *fide*Wollenweber & Reinking (1935).  

***zanthoxyli Fusarium*** X. Zhou *et al.*, Mycologia 108: 675. 2016.

*Holotypus*: HMNWAFU XZ-Fyzs133-20130408

*Ex-type culture*: CBS 140838 = NRRL 66285.

*Type locality*: **China**, Shaanxi, Tongchuan, Yaozhou, Sunyuan.

*Type substrate*: *Zanthoxylum bungeanum*.

*Descriptions and illustrations*: See Zhou *et al.* (2016).

*Diagnostic DNA barcodes*: *rpb1*: KM520383; *rpb2*: KM236763; *tef1*: KM236703.  

*zavianum Fusarium* (Sacc.) Sacc., Syll. Fung. 4: 709. 1886.

*Basionym*: *Fusisporium zavianum* Sacc., Michelia 1: 83. 1877.

(See ***Fusarium lateritium***)

*Holotypus*: In PAD.

*Type locality*: **Italy**, Vittorio.

*Type substrate*: *Vitis vinifera*.

*Note*: Synonyms *fide*Wollenweber & Reinking (1935).  

*zeae Fusarium* (Westend.) Sacc., Syll. Fung. 4: 713. 1886.

*Basionym*: *Fusisporium zeae* Westend., Bull. Acad. Roy. Sci. Belgique, Cl. Sci. 18: 414. 1852. (*non Fusisporium zeae* Roum., Rev. Mycol. (Toulouse) 6: 163. 1884).

(See ***Fusarium avenaceum***)

*Holotypus*: BR5020141668483.

*Type locality*: **Belgium**, Kortrijk railway station.

*Type substrate*: Rotting stalks of *Zea mays*.

*Note*: Synonyms *fide*Wollenweber & Reinking (1935).  

*zealandicum Fusarium* Nirenberg & Samuels, Canad. J. Bot. 78: 1483. 2000.

***Geejayessia zealandica*** (Cooke) Schroers, Stud. Mycol. 68: 133. 2011.

*Basionym*: *Nectria zealandica* Cooke, Grevillea 8: 65. 1879.

*Synonyms*: *Cucurbitaria zelandica* (Cooke) Kuntze, Revis. Gen. Pl. 3: 462. 1898.

*Cosmospora zealandica* (Cooke) Samuels & Nirenberg, Canad. J. Bot. 78: 1483. 2000.

*Holotypus*: BPI 747915*.*

*Ex-type culture*: BBA 64792 = CBS 111.93.

*Type locality*: **New Zealand**, Auckland, Waitakere Ranges Regional Park, Cascades Kauri.

*Type substrate*: Bark of *Hoheria populnea.*

*Descriptions and illustrations*: See Nirenberg & Samuels (2000).

*Diagnostic DNA barcodes*: *rpb2*: HM626684; *tef1*: HQ728148.  

*ziziphinum Fusarium* Pass., Erb. Critt. Ital. ser. 2 no. 1084. 1881.

(See ***Fusarium lateritium***)

*Syntype*: F 982523 ( Erb. Critt. Ital. no. 1048).

*Type locality*: **Italy**.

*Type substrate*: Twigs of *Ziziphus sinensis* (syn. *Ziziphus jujuba*).

*Note*: Synonym *fide*Wollenweber & Reinking (1935).  

*zonatum Fusarium* (Sherb.) Wollenw., Fusaria Autogr. Delin. 1: 392. 1916.

*Basionym*: *Fusarium lutulatum* var. *zonatum* Sherb., Mem. Cornell Univ. Agric. Exp. Sta. 6: 214. 1915.

(See ***Fusarium oxysporum***)

*Typus*: ?CUP-007453.

*Type locality*: **USA**, New York, Ithaca.

*Type substrate*: *Solanum tuberosum.*

*Notes*: Synonym *fide*Wollenweber & Reinking (1935). Lectotypification pending study of the material lodged in CUP.  

*zygopetali Fusarium* Delacr., Bull. Soc. Mycol. France 13: 103. 1897.

*Holotypus*: ?PC.

*Type locality*: **France**, Paris, Luxembourg gardens.

*Type substrate*: Leaves of *Zygopetalum maculatum* (syn. *Zygopetalum mackayi*).

*Notes*: Status unclear. Not *Fusarium fide*Wollenweber & Reinking (1935).

## Conclusions

The present study is the first to provide an up-to-date morphological, biochemical, and phylogenetic overview of the 20 fusarioid genera that are presently recognised in *Nectriaceae*. Morphological species recognition frequently fails to distinguish fusarioid taxa that have been described based on genealogical concordance phylogenetic species recognition (GCPSR *sensu* Taylor *et al.* 2000). To address this issue, we have established a new database, Fusarioid-ID, with accurate names for species and genera of fusarioid taxa. Although the phylogenetically most informative genes remain *tef1*, *rpb1* and *rpb2*, additional markers such as *act1*, *CaM*, *tub2*, ITS and LSU are also incorporated. These genetic fragments can be amplified by PCR and sequenced using the primers indicated in Table 2. In the future, new species and other phylogenetically informative orthologous genes, will be added to resolve isolates at species and genus level. Researchers interested in obtaining reference strains should contact the Westerdijk Fungal Biodiversity Institute (https://wi.knaw.nl/page/Collection), which houses a large collection of phylogenetically diverse fusarioid taxa.

As we have shown here, the phylogenetically derived argument that species under the node F1 should be considered members of “*Fusarium*” is not practical, as this circumscription would lead to a genus without apparent synapomorphies, as lineages outside the genus would also share its characteristics. However, the F3 node (corresponding to *Fusarium s. str.*) is resolved by all genetic markers so far analysed (*e.g.*, see Geiser *et al.* 2021) and delineates the morphologically, ecologically, and biochemically well-delineated genus *Fusarium*.

*Fusarium s. str.* does not have different sexual morphs, other than *Gibberella*. Fusarioid genera are not only morphologically distinct, but as we have shown in this study, correlate to different monophyletic groups and also differ in their biology and mycotoxin profiles.

One of the reasons for the desire to classify any species producing conidia with foot-shaped basal cells into a single genus could be that plant pathologists and clinicians typically isolate conidia or obtain cultures from vegetative mycelium that inhabits their specimens. Also, Wollenweber and his successors may have primarily worked with vegetatively proliferating materials, although it was also Wollenweber (1924, 1926) who produced the first general synopsis of holomorphs in the *Hypocreales*. However, mainly Joan M. Dingley (1951, 1957), Colin Booth (1959), and especially Gary J. Samuels (Samuels 1976a, b, 1978, 1988, Samuels *et al.* 1991) significantly changed our points of view by systematically isolating ascospores obtained from ascomata, of which a vast majority were not gathered in agricultural fields but from woody or herbaceous substrata in forests of pantropical, species-rich regions. The result of their taxonomic considerations was an infrageneric subgrouping system in *Nectria* that was based on sexual and asexual connections. The classification of species according to morphological similarities in sexual morphs allowed understanding patterns of asexual characteristics that are unique for the sexually defined subgroups and eventually correlating sexual groupings with Wollenweber's section system. The diversity of nectria-like species Samuels looked at is huge and was eventually interpreted on the level of families, within which numerous genera were recognised or newly described (Rossman *et al.* 1999) with infrageneric, informal species groups of *Nectria* accepted at the genus level (*e.g.*, see Chaverri *et al.* 2011 and subsequent studies). Applying the generic level to the numerous nectria-like subgroups producing fusarioid conidia is therefore another small but unavoidable step towards a taxonomic system that allows distinguishing natural diversity above the species level based on morphologically and phylogenetically well-defined units.

When Colin Booth delivered his Presidential address to the British Mycological Society in 1977, he chose the title “Do you believe in genera?”. He addressed this topic based on his interpretation of *Nectriaceae* (Booth 1978). Booth subsequently showed that several “groups” of species formed fusarioid asexual morphs, namely *Gibberella* (now *Fusarium s. str.*), *Haematonectria* (now *Neocosmospora*), *Nectria episphaeria* (now *Cosmosporella* and *Dialonectria*), and *Calonectria rigidiuscula* (now *Albonectria*). Booth concluded that the “fusarium morphs” reflected “terms of convenience” rather than genealogical relationships. In moving to the one fungus = one name nomenclature (Hawksworth *et al.* 2011, Wingfield *et al.* 2012), *Fusarium s. str*. was chosen over *Gibberella* (Gräfenhan *et al.* 2011, Schroers *et al.* 2011, Rossman *et al.* 2013). As the genus *Fusarium* was thus clearly well-defined, other *Nectriaceae* lineages with a fusarium-like morphology were recognised (Gräfenhan *et al.* 2011, Schroers *et al.* 2011, Lombard *et al.* 2015, Lechat & Fournier 2015). As we have shown here, taxa are constantly being newly collected and added to the phylogeny of *Nectriaceae*. The only stable option forward is to apply and use the genus name *Fusarium* (= *Gibberella*) as more precisely defined based on its own monophyletic node as presented here (F3), supported by morphology, biochemistry, and biology.

## Disclaimer

The present paper represents a separate initiative to Geiser *et al.* (NSF 1655980): A phylogenetic revisionary monograph of the genus *Fusarium*.

## Figures and Tables

**Fig. S:**
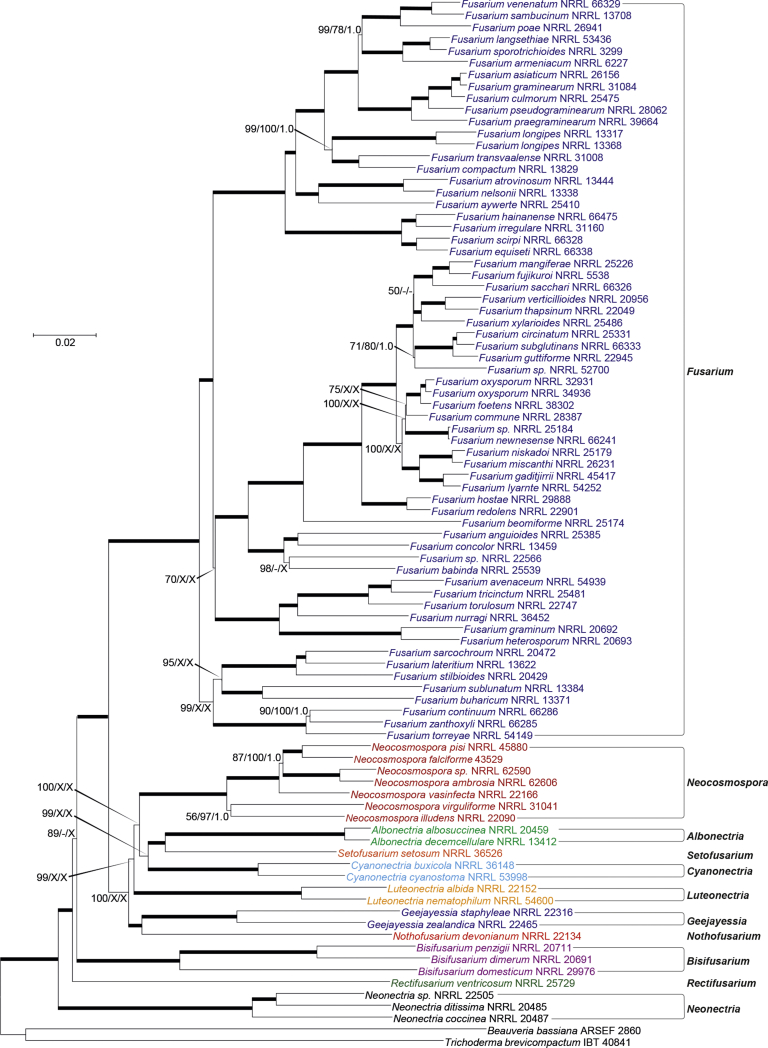
3Phylogenetic reconstruction from a re-analysis of the dataset presented by Geiser *et al.* (2021) based on Minimum Evolution, with values on the branches representing support in Minimum Evolution, Maximum Likelihood, and Bayesian Inference, respectively. An X represents a conflicting topology in the particular analysis, while a – (dash) sign means lack of support for the presented or an alternate topology. The scale bar represents the amount of substitution per site. ARSEF: Collection of entomopathogenic fungal cultures, Agricultural Research Service (ARS), US Department of Agriculture (USDA), Ithaca, NY, USA. NRRL: ARS, National Center for Agricultural Utilization Research, USDA, Peoria, IL, USA. IBT: Culture Collection of Fungi, BioCentrum, Technical University of Denmark, Lyngby Denmark.

**Fig. S:**
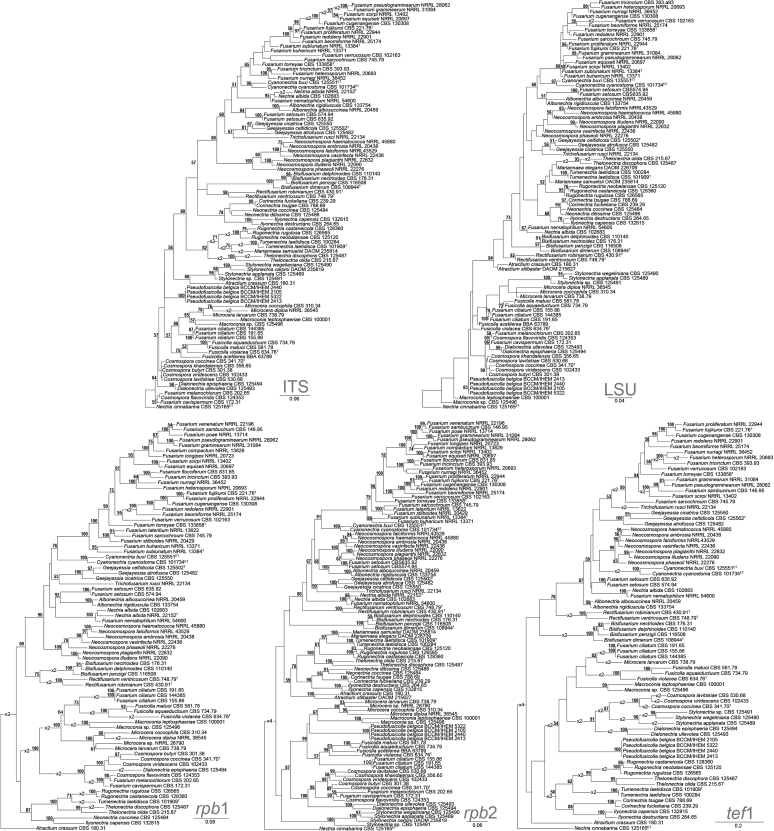
4RAxML single gene phylogenies.
